# GLASSgo – Automated and Reliable Detection of sRNA Homologs From a Single Input Sequence

**DOI:** 10.3389/fgene.2018.00124

**Published:** 2018-04-17

**Authors:** Steffen C. Lott, Richard A. Schäfer, Martin Mann, Rolf Backofen, Wolfgang R. Hess, Björn Voß, Jens Georg

**Affiliations:** ^1^Genetics and Experimental Bioinformatics, Faculty of Biology, University of Freiburg, Freiburg, Germany; ^2^Institute of Biochemical Engineering, University of Stuttgart, Stuttgart, Germany; ^3^Bioinformatics Group, Faculty of Computer Science, University of Freiburg, Freiburg, Germany; ^4^Forest Growth and Dendroecology, Institute of Forest Sciences, University of Freiburg, Freiburg, Germany; ^5^ZBSA Center for Biological Systems Analysis, University of Freiburg, Freiburg, Germany; ^6^BIOSS Centre for Biological Signalling Studies, Cluster of Excellence, University of Freiburg, Freiburg, Germany; ^7^Center for Non-coding RNA in Technology and Health, University of Copenhagen, Frederiksberg, Denmark; ^8^Freiburg Institute for Advanced Studies, University of Freiburg, Freiburg, Germany

**Keywords:** sRNA, prediction, homology search, ncRNA, bacteria, Rfam, comparative genomics, graph-based clustering

## Abstract

Bacterial small RNAs (sRNAs) are important post-transcriptional regulators of gene expression. The functional and evolutionary characterization of sRNAs requires the identification of homologs, which is frequently challenging due to their heterogeneity, short length and partly, little sequence conservation. We developed the GLobal Automatic Small RNA Search go (GLASSgo) algorithm to identify sRNA homologs in complex genomic databases starting from a single sequence. GLASSgo combines an iterative BLAST strategy with pairwise identity filtering and a graph-based clustering method that utilizes RNA secondary structure information. We tested the specificity, sensitivity and runtime of GLASSgo, BLAST and the combination RNAlien/cmsearch in a typical use case scenario on 40 bacterial sRNA families. The sensitivity of the tested methods was similar, while the specificity of GLASSgo and RNAlien/cmsearch was significantly higher than that of BLAST. GLASSgo was on average ∼87 times faster than RNAlien/cmsearch, and only ∼7.5 times slower than BLAST, which shows that GLASSgo optimizes the trade-off between speed and accuracy in the task of finding sRNA homologs. GLASSgo is fully automated, whereas BLAST often recovers only parts of homologs and RNAlien/cmsearch requires extensive additional bioinformatic work to get a comprehensive set of homologs. GLASSgo is available as an easy-to-use web server to find homologous sRNAs in large databases.

## Introduction

Small regulatory RNAs (sRNAs) are important regulators of gene expression in bacteria (Wagner and Romby, 2015). In recent years, the number of identified bacterial sRNAs has increased dramatically, but their functional characterization is lagging behind, requiring the development of novel technologies and approaches (Barquist et al., 2015). An initial step in the validation of candidate sRNAs from computational predictions or transcriptomic analyses is to test for their evolutionary conservation. A phylogenetically conserved sRNA is, e.g., more likely to be of functional importance. Moreover, comparative computational tools for the prediction of sRNA targets (Wright et al., 2013, 2014), for the calculation of a potentially conserved secondary structure (Bernhart et al., 2008; Katoh and Toh, 2008; Smith et al., 2010) and for the prediction of small open reading frames of potential μ-proteins or dual function sRNAs (Washietl et al., 2011) require the input of multiple members of an sRNA family. In high-throughput dRNA-seq/RNA-seq experiments, the comparative approach can be also used to predict potential sRNAs from scratch (Lott et al., 2017).

Homologous sRNAs are conserved both at the sequence and the structure level. For each set of homologous sRNAs it is unclear how much additional information can be gained when considering the secondary structures besides their primary sequences. However, the importance of the secondary structure as a feature increases when the overall sequence conservation drops (Nawrocki and Eddy, 2013a). Hence, methods for the prediction of sRNA homologs (reviewed in reference Freyhult et al., 2006) are based on sequence comparison or the combination of sequence and structure information. Tools like, e.g., BLASTn (Altschul et al., 1990) or profile hidden Markov models (Madera and Gough, 2002; Lindgreen et al., 2014) use the primary sequence for predictions. Probabilistic models (covariance models, CM) as implemented in Infernal (Nawrocki and Eddy, 2013b) utilize the conserved sequence and secondary structure of an RNA family, thereby gaining additional information to detect homologs with low sequence conservation. However, the construction of CMs requires a structurally annotated single sequence or a multiple sequence alignment. The Rfam database (Nawrocki et al., 2015) contains pre-built models for a limited set of currently approximately 500 sRNA families, which can be used to scan (meta) genome or transcriptome data with Infernal. Therefore, a common strategy to systematically find homologs of a newly detected sRNA from a so far undescribed family starts with searching databases for homologous sequences, e.g., by BLASTn, followed by manual curation of the candidate list and generation of a multiple sequence/structure alignment. These information are used to build a CM (Reinkensmeier et al., 2011; Lagares et al., 2016) with which additional homologs can be searched with high specificity. Each of these steps either requires extensive manual action, or a solid background in bioinformatics. These facts represent a high entry barrier for non-expert users (Menzel et al., 2009). The tool RNAlien (Eggenhofer et al., 2016) has automated the first step of this approach and allows unsupervised CM construction on a web server. Despite this advance, the user is still left with the task to use the CM to search for new homologs in sequence databases. While this sounds trivial, it actually requires the user to download sequence databases, install the Infernal software and run the tool using the command line. All these steps represent substantial barriers for experimental biologists. To solve these problems, we developed a fully automated workflow that combines the speed of BLAST, a high specificity, as known for CM-based approaches, with an easy access for wet lab users. Our tool GLASSgo is based on an iterative BLASTn search strategy with pairwise identity filtering. To account for the conservation of RNA secondary structures, we additionally developed a fast, graph-based, clustering method called Londen. Within a few minutes, GLASSgo automatically generates sets of conserved sRNAs that need no, or only little manual curation. These sets are ideally suited for comparative genomics analyses, the description of new RNA families (for submission to Rfam) and to study their evolution and phylogenetic distribution. GLASSgo is accessible via an easy-to-use web server^1^. Furthermore, the source code of GLASSgo is available at GitHub^2^ and a Docker image at DockerHub^3^.

## Materials and Methods

### Sequence-Based Search

BLASTn (version 2.2.30+) and the NCBI ‘nt’ database (from November 08, 2015) were used for sequence similarity searches. Hits with an *E*-value lower or equal to a predefined threshold are considered for the further analyses. The BLAST algorithm often returns local hits that are shorter than the original query sequence, therefore we apply an asymmetric sequence extension. It computes the ranges of the missing parts with respect to the homologous part in the query sequence and extracts the information from e.g. the NCBI ‘nt’ database via the blastdbcmd tool from the NCBI BLAST+ suite. Based on a global sequence alignment (Gotoh, 1982), the pairwise identity of each hit to the query sequence was computed. For performing a pairwise sequence comparison, the “pairwise2.align.globalms” function from the Biopython module pairwise2 was used with the parameter setting *match = 2, mismatch = -1, gapOpen = -0.5* and *gapExtend = -0.1*. For our benchmark, we restricted the BLAST search to the phylum of the respective input sequence and used an E-value of 1.

### Sequence Based Homolog Classification and Sequence Selection for Iterative BLAST Search

After the BLAST search the sequences are classified according to their global pairwise sequence identity to the query (PI). BLASTn requires ∼60-65% sequence identity to identify a significant similarity for typical sRNAs (Nawrocki and Eddy, 2013a). We decided to choose a conservative threshold for true homolog classification based on sequence similarity independent of sRNA length and database size. Thus hits with a *PI > 70%* are considered “true homologs,” those with a PI between the lower PI threshold (default: 52%) and 70% are considered “candidate homologs.” The “candidate homologs” are evaluated by a secondary structure based clustering as described below. To increase the sensitivity of GLASSgo, selected hits with a PI between 65 and 80% are used for re-blasting. From hits with a PI in the intervals (65, 70) and (70, 80) we select up to 45 each and from the interval (80, 100) we take up to 10 hits. This set forms the query for a new round of BLAST searching, sequence extension and PI based filtering. The repeated blasting potentially generates duplicated sequences that are collapsed prior to the next steps.

### Sequence Based Pre-clustering as Input for Structural Analysis

The input sequences were roughly pre-grouped based on their pairwise sequence identity calculated with the Clustal Omega k-tuple method (Sievers et al., 2011). Only one representative sequence (selection described below) of each group is subsequently analyzed with RNApdist (Bonhoeffer et al., 1993; Hofacker et al., 1994; Lorenz et al., 2011) and used for the clustering by Londen (described below). This step reduces the runtime and prevents the existence of too similar sequences, which could result in relatively short branches and subsequently in a strict small cutting radius. This could interfere with the automatic scaling procedure of Londen. As shown in **Supplementary Figure S3** we first select the most distant sequence to the query as representative for later processing. All sequences that have a similarity *≥85%* to this representative sequence were grouped and excluded from the similarity matrix prior to the next iteration. From the remaining sequences we again select the sequence with the lowest PI to the query and collect its neighborhood with a similarity *≥85%*. This procedure is repeated until no sequence remains. In contrast to, e.g., single linkage clustering on a pairwise identity matrix this approach prevents chaining, i.e., the linkage of very dissimilar sequences due to a single intermediate observation. If one of the representatives is classified as structurally similar to a positive sequence (i.e., the query or a true positive hit) by Londen (see next paragraph), all respective group members are classified as structurally similar. While not extensively screened, all sequence based similarity thresholds were roughly optimized based on the 40 benchmark sRNAs.

### Structure Based Clustering (Londen)

**FIGURE 1:**
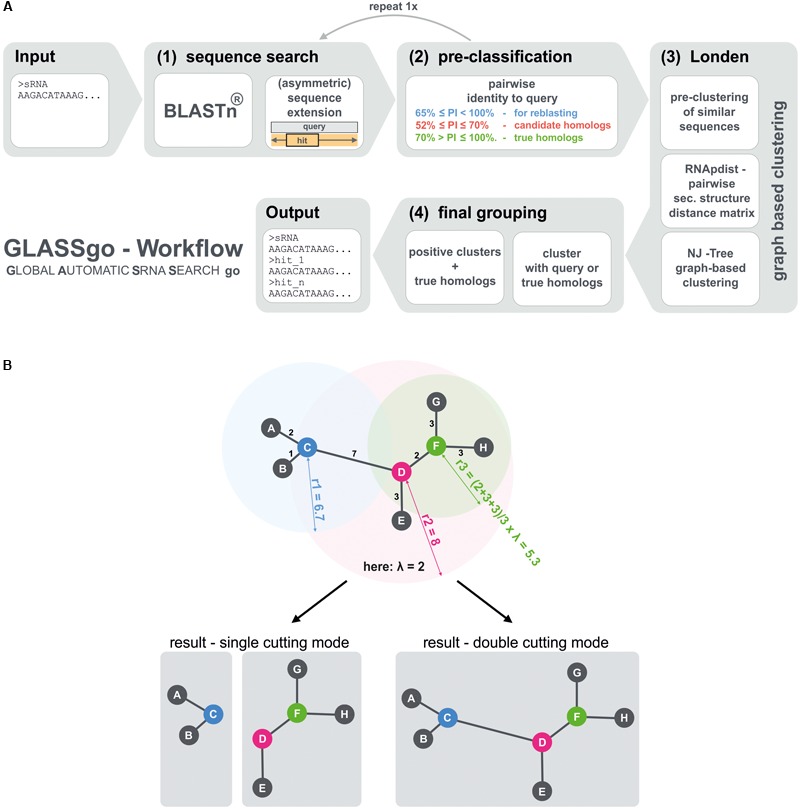
**(A)** Main steps of the GLASSgo workflow. **(B)** Illustrates the function of the tree based structural filter Londen. The leaves of the tree represent the predicted homologs (gray circles) and internal nodes (blue, pink, and green circles) represent respective hypothetical common ancestors. Londen decides for each internal node if the node and the corresponding leaves should be divided from the initial tree to build an independent tree/cluster, or if the node should stay connected to the adjacent internal node(s). Therefore the mean of the length of all edges connected to a specific internal node is calculated and multiplied with the stringency factor λ (In this example λ = 2) to get the cutting radius. If an adjacent edge is longer than the cutting radius, the edge is cut. In consequence a higher stringency factor leads to less stringent cutting because the cutting radius will be higher. The stringency factor is automatically calculated in the range of based on the input sequence length. Londen features two cutting modes. The more stringent “single cutting mode” cuts between two inner nodes if the cutting radius of at least one of both nodes indicates a separation. In the “double cutting mode” both cutting radiuses are needed to support a separation.

For the representative sequences from the above described sequence based pre-grouping algorithm we applied RNApdist (Bonhoeffer et al., 1993; Hofacker et al., 1994; Lorenz et al., 2011) (version 2.2.5) to compute a pairwise secondary structure distance matrix, which is used to perform structure-based clustering. Sequences in a cluster that contains at least one true homolog or the input sequence itself, will be defined as true homologs. For the clustering, we introduced a new approach that is inspired by graph-based clustering (Zahn, 1971). Graph-based clustering works on a minimum spanning tree and generates a clustering by removing inconsistent edges, i.e., edges, whose length is significantly larger than the length of neighboring edges. It can be considered as an improvement of single-linkage clustering that prevents chaining. Instead of a minimum spanning tree as in the original graph-based clustering, we relied on neighbor-joining (NJ) to generate a spanning tree. NJ was shown to produce biologically correct trees in several studies and outperforms other distance-based methods (Saito and Imanishi, 1989; Huelsenbeck, 1995). Thus, we first calculate a cluster tree using NJ based on the structure distance matrix that was calculated with RNApdist. Then we identify inconsistent edges as follows: Let $H_{i}^{\neg e}$ be the set of edges connected to node *i* excluding edge *e*, and L^e^ the length of edge *e*. For an edge *e* that connects nodes *i* and *j*, we compute the mean length *M* of the respectively connected edges, i.e., $M_{i}^{e}=\frac{\sum_{k\in H_{i}^{\neg e}}L^{k}}{\left|H_{i}^{\neg e}\right|}$ and $M_{j}^{e}=\frac{\sum_{k\in H_{j}^{\neg e}}L^{k}}{\left|H_{j}^{\neg e}\right|}$ Londen supports two cutting methods, called single cut and double cut mode. In single cut mode, edge *e* is removed if $L^{e}>\lambda \cdot M_{i}^{e}\;or\;L^{e}>\lambda \cdot M_{j}^{e}$. In double cut mode, edge *e* is removed, if $L^{e}>\lambda \cdot M_{i}^{e}\;and\;L^{e}>\lambda \cdot M_{j}^{e}$. The factor λ controls the stringency of the procedure. Londen inspects all edges of the NJ tree, resulting in a, possibly unary, set of trees (the cutting procedure is visualized in **Figure 1B**). From this we select the trees (clusters) that contain the query sequence and/or or a true positive hit and report all enclosed sequences. Through the GLASSgo web server, the user can manually change λ (called “Manual value for filtering” in the interval [0, 3], default value 2); otherwise, GLASSgo adjusts λ automatically based on the following strategy: We estimate the sequence diversity by the ratio *r = α/β*, where *α* is the number of sequences with pairwise identity *PI* ≥ 60% and *β* the number of sequences with *PI* < 60% and at least 2. If *r < 1* (single-cut mode) *λ = min*(0.29^∗^log_10_(L) + *r*, 2.4), else (*r* ≥ 1, double-cut mode) = min(0.01^∗^log_10_(*L*) + *r*, 2.4), where *L* is the sequence length. In case of a manually set *λ*, GLASSgo uses the double-cut mode. By changing the parameter (-l, –londen_mode), it can be switched from the double-cut mode to the single-cut mode (not provided on the web server version). The used Londen parameters were optimized on the 40 benchmark RNAs. To evaluate the adjusted parameters, we tested GLASSgo on 15 additional sRNA families and compared the results to BLAST. For this new set GLASSgo reached a total true positive (TP) number of 3,777 [69 false positives (FPs), a mean positive predictive value (PPV = TPs/(TPs + FPs)] of 0.986 and a minimum PPV of 0.79. The data for each family are available in **Supplementary Datasheet S2**.

### Test Case Design

For the benchmark, we selected 40 Rfam sRNA representatives from various bacterial phyla and published evidence of actual transcription. The sRNAs cover a sequence length range of 61–429 nucleotides. The input sequences for the benchmark were the first entries in the respective Rfam seeds of the benchmark sRNA families. The selected 40 Rfam families are: RF00034, RF00035, RF00057, RF00111, RF00117, RF00444, RF00503, RF01389, RF01391, RF01395, RF01399, RF01402, RF01408, RF01460, RF01471, RF01472, RF01476, RF01477, RF01493, RF01675, RF01783, RF01820, RF01828, RF02050, RF02072, RF02099, RF02237, RF02238, RF02243, RF02273, RF02353, RF02376, RF02377, RF02378, RF02405, RF02417, RF02452, RF02502, RF02503, and RF02677. The used sequences for each family are available in **Supplementary Datasheet S1**. The 15 Families used to evaluate the adjusted parameters were: RF00039, RF00079, RF00083, RF00166, RF00195, RF00378, RF00616, RF01116, RF01416, RF01701, RF01808, RF01816, RF02394, RF02396, and RF02552.

### cmsearch

The tool cmsearch comes with Infernal [v1.1.2 (July 2016)] and allows to search covariance models against a given sequence database (Nawrocki and Eddy, 2013b). To make the results of cmsearch comparable to those of GLASSgo and BLAST, we used the same phylum-specific databases. Default parameter settings were used and only significant hits (as indicated by cmsearch) were considered for further analyses. The cmsearch tool also finds fragments shorter than the sequences that led to the CM model. For the benchmark analysis, all hits with a sequence length <70% of the original input were discarded.

### cmscan

The tool cmscan comes with Infernal [v1.1.2 (July 2016)] and allows to search a sequence against its related covariance model. cmscan is used to evaluate all predicted sequences coming from GLASSgo, RNAlien/cmsearch as well as BLAST vanilla. The classified sRNA homologs are available in **Supplementary Datasheet S1**.

### RNAlien

We created a docker container (v1.12.6) based on openSUSE 42.2 Leap and installed RNAlien^4^ version 1.3.7. Furthermore, we installed the following dependencies: RNAz (v2.1) (Gruber et al., 2009), ViennaRNA Package (v2.3.4) (Lorenz et al., 2011), Infernal (v1.1.2) (Nawrocki and Eddy, 2013b), RNAcode (v0.3) (Washietl et al., 2011) and LocARNA (v1.9.2) (Smith et al., 2010).

### Synteny Analysis

For the synteny analysis, amino acid sequences of all protein-coding genes overlapping a window 3 kb up- and downstream of the sRNA locus were extracted from the respective Genbank files and clustered with CD-HIT (Huang et al., 2010) using a sequence identity threshold of 0.4 and a word size of 2. Furthermore, for clustering, the alignment needs to cover at least 60% of the longer sequence. A visualization of the gene neighborhood of all RF00111 homologs predicted by GLASSgo is given in the **Supplementary Datasheet S3**.

### Pairwise Diversity Analysis as Proxy for Sensitivity and Complexity

The pairwise diversity for each Rfam family is computed by taking all predicted sequences per family into account that are classified as true positives by cmscan. We calculated the pairwise similarity for each detected TP homolog to the respective query. This is done for the GLASSgo results, the RNAlien/cmsearch results and the extended BLAST results. For performing a pairwise sequence comparison, the same software package and parameters, as described for “Sequence based search,” were used.

### Runtime Analysis

The 40 selected Rfam sRNA representatives from the benchmark were used to analyze the runtime of GLASSgo, RNAlien/cmsearch and BLAST. All three algorithms are successively applied on the same dedicated infrastructure and the runtime (single core) was measured using the Linux time command. Calculations were carried out on a dual-CPU system (Intel Xeon Broadwell E5-2680 v4, 2.40GHz) with 128GB DDR4 SDRAM running on 2400MHz. Bandwidth of the internet connection has been measured (speedtest-cli v1.0.6)^5^ at 45/75Mbit/s (Download/Upload) against servers at Swift Systems (ID: 246) located 60 km north of the NCBI (Bethesda, MD, United States).

### Taxonomic Tree

In order to get an overview of the taxonomic distribution of the detected GLASSgo hits, the GLASSgo web server provides an interactive taxonomic tree of the organisms harboring a predicted sRNA homolog. Therefore, the taxonomic identifier of each GLASSgo hit is used to compute the taxonomic path up to the root node. Multiple occurring paths are merged and counted. Finally, the tree is transferred into JSON format and visualized via a JavaScript application.

## Results

### GLASSgo Workflow

The GLASSgo workflow consists of 4 major steps (**Figure 1A**). The first step is a sequence search with BLAST, providing a set of putative homologs of the input sRNA. Second the sequences are classified based on their pairwise identity to the input sequence (PI). Hits with a PI > 70% are directly taken as true homologs due to their high sequence conservation. Hits with a PI between 70% and an adjustable lower PI threshold (default 52%) are considered candidate homologs and further analyzed based on their secondary structure in a tree based clustering approach in step three. BLAST hits with a PI below the threshold are not considered any further. In step four, positive structural clusters are filtered and the corresponding sequences as well as their true homologs are returned in a FASTA file as homologs of the input sRNA. The taxonomic distribution of the respective organisms is furthermore visualized in an interactive tree (provided on the web server version).

The main new contribution of GLASSgo to the field of sRNA homolog prediction is the application of a tree/graph-based clustering approach called “Londen” to group sRNAs with a similar secondary structure (**Figure 1B**). To this end we compute the distances between the putative homologs based on their RNA secondary structure ensembles using RNApdist (Hofacker et al., 1994). The resulting pairwise distance matrix is used to construct a neighbor joining tree, which is clipped by an auto-adaptive procedure to generate clusters of sRNAs with similar secondary structures. In order to decide which clusters likely contain true homologs of the query sRNA we use the true positive hits and the query sRNA as references. Each cluster containing at least one of these reference sequences is considered as positive cluster and all candidate hits within these positive clusters are considered as true homologous sRNAs. A more detailed visualization of the workflow is given in **Supplementary Figure S1**.

### Benchmark With Known sRNAs

GLASSgo was tested on 40 sRNAs from the Rfam database (Nawrocki et al., 2015). The selected sRNAs are 61 to 429 nucleotides long and originate from various taxa. Only sRNAs with published evidence for their actual transcription were considered. We compared the performance of GLASSgo in terms of specificity, sensitivity and runtime with BLAST and a combination of RNAlien and cmsearch (Nawrocki and Eddy, 2013b; Eggenhofer et al., 2016). For BLAST alone we used the same parameters as in the BLAST step of GLASSgo (*E*-value = 1, taxon specific databases). To test the RNAlien/cmsearch combination we first built models for all sRNA families with RNAlien and then used these models to scan the same taxon specific databases with cmsearch that were used for GLASSgo as well as BLAST. Only hits that covered at least 70% of the query sequence and were classified as “significant” by cmsearch were considered further. Detailed benchmark results are given in **Supplementary Datasheet S2**, a summarized overview is given in **Table 1**.

**Table 1:** Overview of benchmark results.

		GLASSgo	RNAlien/cmsearch	BLAST
True positives (TP)	Mean	229.4	244.1	216.9
	Median	83.0	82.5	117.0
	Maximum	1434	1417	1446
False positives (FP)	Mean	2.13	0.1	354.4
	Median	0.0	0.0	27.5
	Maximum	18	1	7042
PPV	Mean	0.99	1.00	0.67
	Median	1.00	1.00	0.78
	Maximum	1.00	1.00	1.00
	Minimum	0.85	0.98	0.01
Run time [s]	Mean	191	12308	26
	Median	92	10660	12
	Maximum	2409	26258	275
	Minimum	4.8	4039	1.55

#### Sensitivity and Specificity

**FIGURE 2:**
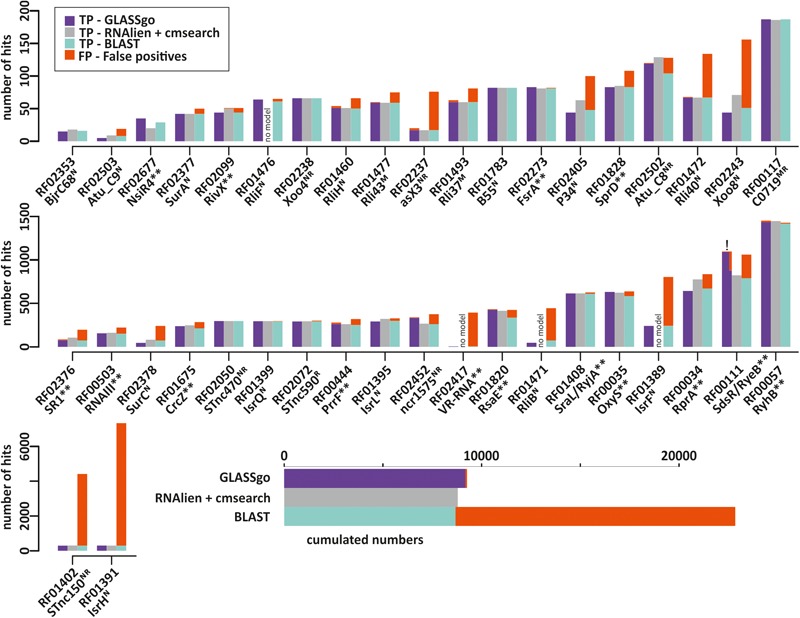
Number of true positive (TP) and false positive (FP) hits per Rfam sRNA family detected by GLASSgo, RNAlien/cmsearch and BLAST. FP hits are accumulated on top of the bars and displayed in red. Families where RNAlien detected no homologs are indicated by “no model.” The inlay in the lower part shows the cumulated TP and FP numbers for all 40 families. The name of the sRNA as used in literature is given next to the Rfam ID of the respective sRNA family. The superscript letters indicate the verification method for the respective sRNA (N, Northern Blot; R, RNAseq; M, Microarray). Functionally characterized sRNAs are indicated by two asterisks (^∗∗^). For the RF00111 GLASSgo prediction (marked by “!”) the number of TPs and FPs is given both based on the cmscan result and the refined result including the synteny analysis.

**FIGURE 3:**
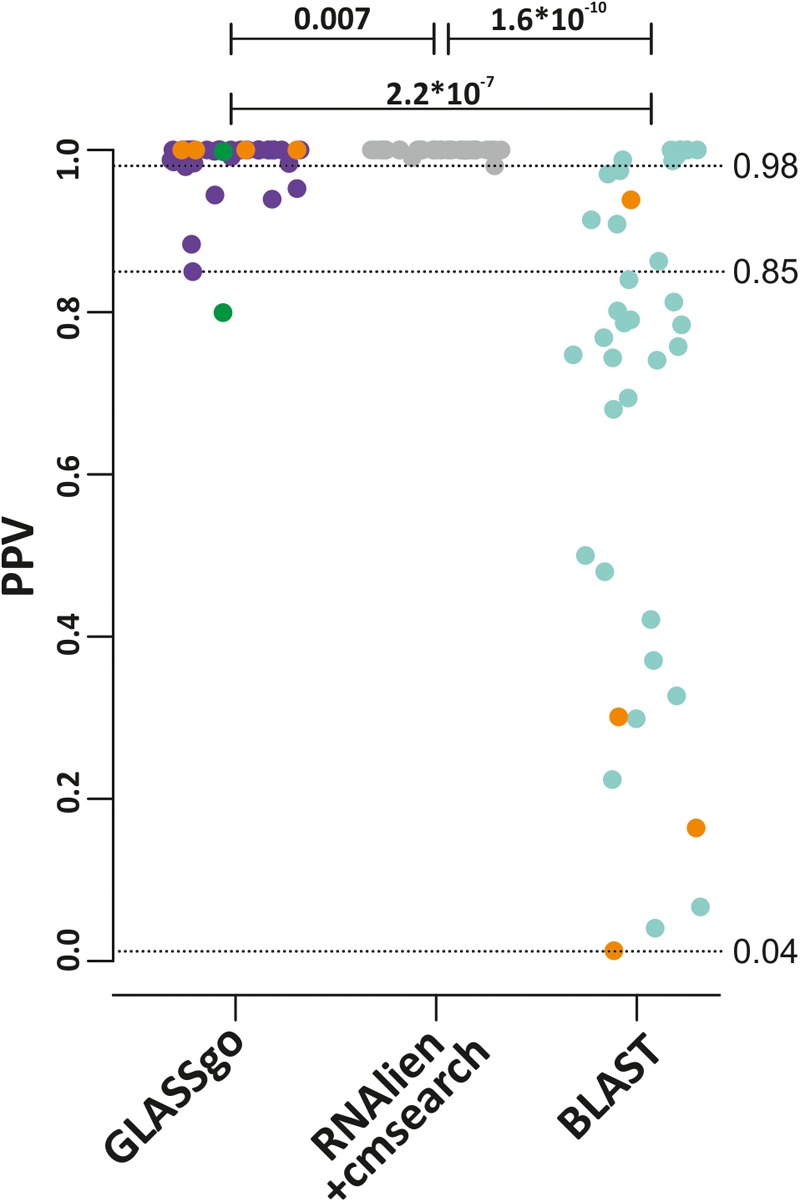
Positive predictive values (PPV) for all predictions from GLASSgo, RNAlien/cmsearch and BLAST. The p-value of a Kruskal–Wallis test for a significant difference between two distributions is shown if ≤ 0.05. The lowest PPV for each method is indicated by a broken horizontal line and the respective value. The four sRNA families which are not present in the RNAlien/cmsearch prediction are colored in orange. The PPV of the GLASSgo RF00111 prediction based on cmscan alone and with the additional synteny analysis are given as green dots.

In order to calculate sensitivity and specificity it is necessary to know the numbers of true positives (TPs) and false positives (FPs) as well as false negative predictions (FNs). This would require knowledge about the number of true sRNA homologs for each Rfam family in the NCBI database. However, since we benchmarked on real life data, the actual “biological truth” is unknown. In order to get a realistic measure of TPs and FPs we used the respective CMs from Rfam to evaluate all predicted candidates with cmscan. The quantity and the sequence diversity of the detected homologs was then used as a proxy for the sensitivity. GLASSgo detected in total 9,182 TP homologs (79 FPs). Initially, cmscan classified 292 sRNA homologs as FPs, but as described below cmscan missed 213 TPs for RF00111/SdsR which could be verified by gene neighborhood analysis. RNAlien/cmsearch detected 8,788 TP (4 FP) homologs and BLAST found 8,674 TPs (14,175 FPs). RNAlien could not build CM models for 4 sRNA families corresponding to 10% of the benchmark. The hits per Rfam family are given in **Supplementary Datasheet S2** and displayed in **Figure 2**. GLASSgo and RNAlien/cmsearch had a high specificity (**Figure 3**) with an average positive predictive value (PPV) of 0.986 and 0.999, respectively. The respective worst performing families in GLASSgo (PPV = 0.85) and RNAlien/cmsearch (PPV = 0.98) showed no dramatic decrease in specificity. BLAST had a mean PPV of 0.692 and 22/40 families with a PPV below 0.8. The FASTA files with all GLASSgo, RNAlien/cmsearch and BLAST predictions are available in **Supplementary Datasheet S1**.

#### RF00111 – SdsR

**FIGURE 4:**
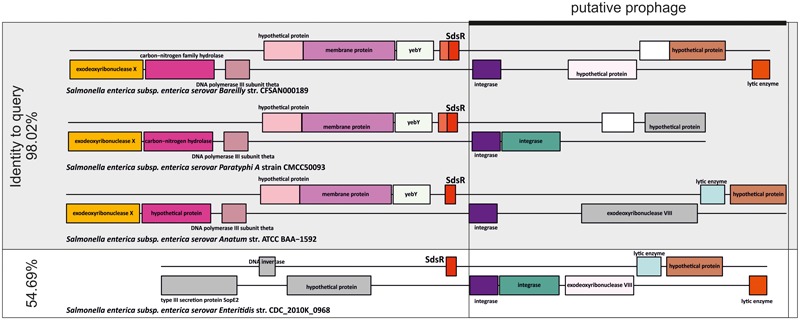
Synteny analysis of three homologs of RF00111/SdsR that are classified as TPs and a homolog that was classified as FP by cmscan. The TPs have a sequence identity of 98.02% to the query sequence and the supposedly FP has a sequence identity of 54.69% in comparison to the query. All 4 predicted homologs are located in vicinity to a putative prophage (black box). Homologous genes have matching background colors. Genes without homologs are colored gray.

There was only one benchmark sRNA family where the specificity of GLASSgo seemingly dropped below 0.8. Based on the cmscan analysis, GLASSgo detected 877 TPs and 220 FPs for RF00111 (RyeB or SdsR). In order to analyze the reason for this high number of FPs we inspected the gene neighborhood (synteny) of the predicted homologs. A reasonable sequence conservation together with a conserved synteny is a strong indicator that the sequences are indeed related and originate from a common ancestor, as has been shown for MmgR or SgrS (Horler and Vanderpool, 2009; Lagares et al., 2016). Initially, SdsR was described to have a conserved synteny with the *yebY* gene at the 5′ site and no conserved synteny at the 3′ site (Fröhlich et al., 2012). In some strains, e.g., *Salmonella enterica* ATCC BAA-1592 (SdsR homolog has 98% identity to query) a prophage or prophage fragment appears at the 3′ side of SdsR. The predicted homologs from 213/220 of the FP classified homologs are located in vicinity of a homologous prophage, as shown in **Figure 4**. In a likely scenario, the phage transferred a part of SdsR to another genomic location. A multiple alignment of predicted homologs with high and low sequence identity to the query shows that the putatively phage-transferred SdsR homologs have a high conservation at the 3′ end and a low conservation in the 5′ part. This indicates that only a part of SdsR was transferred by the phage (**Supplementary Figure S2**). Interestingly, some of these homologs are classified as TPs by cmscan with a high *E*-value (e.g., *S. enterica* ATCC 9150, *E*-value: 4.7e-05), but only one nucleotide difference causes an *E*-value below the threshold (e.g., *S. enterica* EC20120697, E-value: 0.013) (**Supplementary Figure S2**). In conclusion, these predicted homologs are very likely descendants of a true SdsR homolog and hence no FPs. This likely also applies to some of the SdsR homologs predicted by BLAST. However, the original 5′ part including the promoter sequences are likely missing and it is unclear if these sequences are transcribed. In consequence, the actual PPV for the GLASSgo prediction of the RF00111 family is 0.993.

#### Diversity of TP sRNA Homologs

**FIGURE 5:**
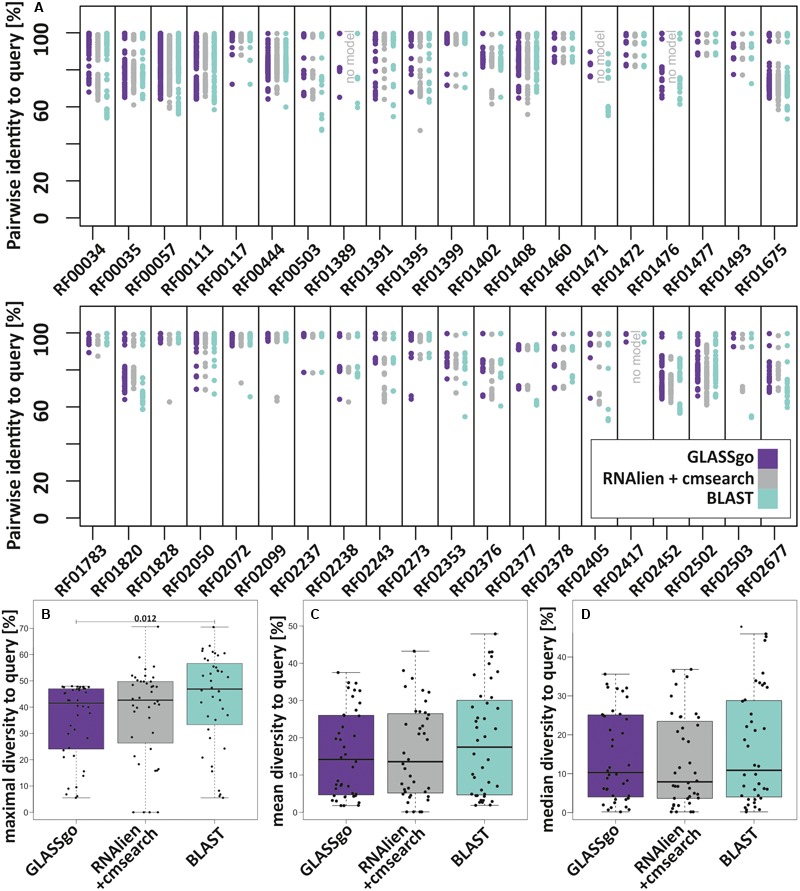
Diversity of the homologs of the 40 benchmark sRNA families in the GLASSgo, RNAlien/cmsearch and BLAST predictions. **(A)** Diversity displayed as the maximum pairwise diversity to the query (mPD) mPD = 100–(thelowestPI[]foreachsRNAfamily). **(B)** Boxplots of the per family mPDs. **(C)** Boxplots of the mean per family diversities of all TP homologs to the respective query. **(D)** Boxplots of the median per family diversities of all TP homologs to the respective query. The *p*-Value of a Kruskal–Wallis test for a significant difference between two distributions in the panels **(B–D)** is shown if ≤ 0.05. The respective values for each family are given in **Supplementary Datasheet S2**.

All tools detected considerable numbers of true homologs. We analyzed if these high numbers are only due to “trivial” hits with nearly perfect sequence identity to the input or if they truly reflect a high sensitivity. Possible methods to investigate the diversity would address their taxonomic distribution or the sequence diversity of the TP hits. For the sake of simplicity, we calculated for all benchmark families the pairwise sequence identities for each TP homolog to the respective query (**Figure 5A**). The lower the observed pairwise sequence identities, the more diverse the analyzed sequences. Thus, we defined the maximum pairwise diversity (mPD) to the query, i.e., *mPD = 100%-min*(*PI*[%]). Using only the mPD as measure, GLASSgo results were more diverse than those of RNAlien/cmsearch in 11 cases and less diverse in 29 cases. Compared to BLAST, GLASSgo results were more diverse in 6 cases, equally diverse in 6 cases and less diverse in 28 cases. BLAST results were more diverse than RNAlien/cmsearch results in 25 cases and less diverse in 15 cases. **Figure 5B** shows that the mPD of GLASSgo is capped at 48%, due to the default PI threshold of 52%. On a closer look, **Figure 5A** shows that the mPD was often dominated by single highly diverse sequences (e.g., RF01395, RF01828, and RF2072). In order to get a better measure of the overall diversity within the detected homologs, we additionally calculated the per family mean diversity (**Figure 5C**) and median diversity (**Figure 5D**). Again, BLAST results showed the highest overall diversity, while GLASSgo and RNAlien/cmsearch performed equally good with slightly higher medians for the GLASSgo results. The diversity values for each family are available in **Supplementary Datasheet S2**.

#### Runtime

**FIGURE 6:**
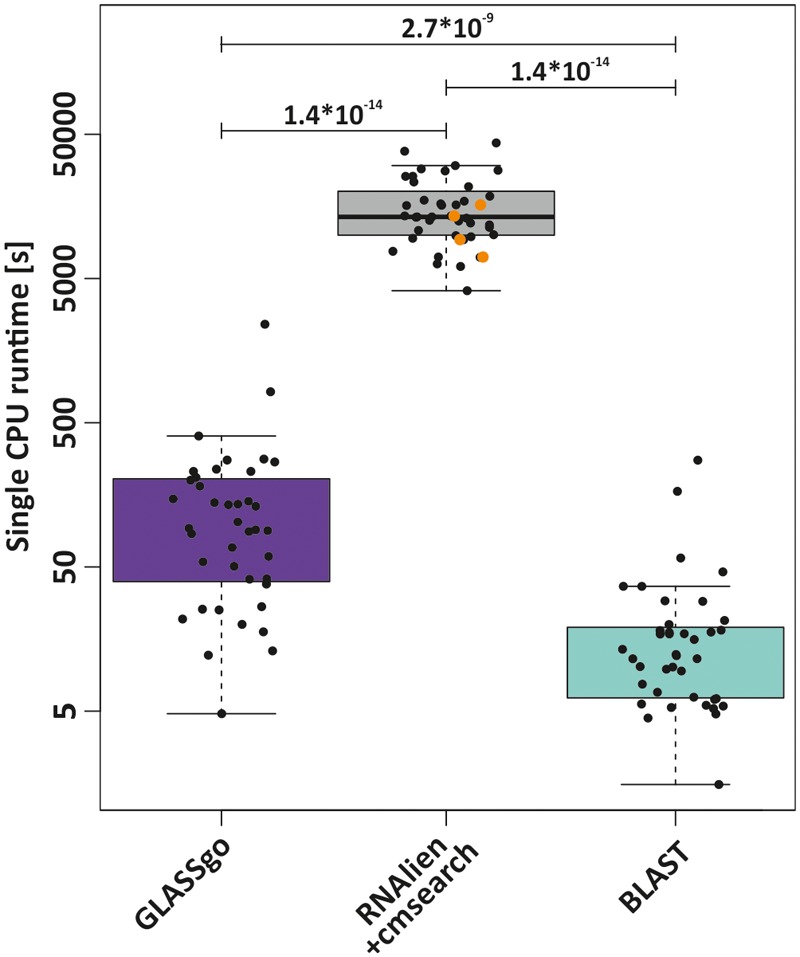
Comparison of the runtimes of GLASSgo, RNAlien/cmsearch and BLAST for the 40 benchmark predictions. RNAlien could not build a covariance model for four families, for these families only the runtime of RNAlien is plotted (orange dots). The *p*-value of a Kruskal–Wallis test for a significant difference between two distributions is shown if ≤ 0.05.

GLASSgo is designed for large-scale analyses of hundreds of sRNAs, e.g., detected in transcriptomic studies, which renders its runtime a critical factor. We compared the job execution times for each of the 40 Rfam queries on a single CPU for GLASSgo, RNAlien/cmsearch and BLAST. The mean and median execution times for GLASSgo were 191.1 and 91.6 s, respectively, while the maximum runtime was 2,409 seconds. The runtime of RNAlien in combination with cmsearch was roughly two orders of magnitude higher with mean, median and maximum execution times of 16,760 (87.7 times slower than GLASSgo), 13,380 (146.1 times slower than GLASSgo) and 43,590 s, respectively. The bulk of the runtime is due to the CM construction by RNAlien. BLAST was the fastest method with mean, median and maximum execution times of 25.7 (7.4 times faster than GLASSgo), 12.2 (7.5 times faster than GLASSgo) and 275 s, respectively (**Figure 6**). The time for the sequence fetching of the BLAST hits is included in this analysis. The individual runtimes per sRNA family are given in **Supplementary Datasheet S2**.

### The Web Server

GLASSgo is provided as an easy-to-use web server^6^. In default mode, with the parameter settings used in this benchmark, the only required input is the sequence of an sRNA of interest. However, there are several options for customization to optimize the homolog prediction for an individual sRNA. It is recommended to limit the BLAST search to a specific taxonomic group (e.g., Bacteria or Proteobacteria). Sensitivity and specificity can be modulated by lowering or increasing the E-value and the lower PI thresholds. The structural filtering (Londen) can be switched off to increase sensitivity. In the default mode, the filter is automatically adjusted to the length of the input sRNA as well as the ratio of the sequence numbers with a *PI ≥ 60%* and those *<60%* related to the query. For a short sRNA with less information at sequence level, the Londen filter is stricter than for a longer sRNA. In custom mode, the structural-filtering value can be relaxed (higher values) or tightened (lower values). The main output of GLASSgo is a FASTA file with all predicted homologs. Additionally, the phylogenetic distribution of the hits is visualized along a taxonomic tree.

## Discussion

The search for homologs is often the first step in the functional characterization of an sRNA. A phylogenetically conserved sRNA is more likely to be of functional importance and less likely to be a sequencing artifact or the result of noisy transcription. GLASSgo allows to discover homologous sRNA sequences from scratch. We compared GLASSgo with two existing homolog prediction approaches, BLAST and the RNAlien/cmsearch combination. BLAST is fast and implemented on a well-established web server that can be easily used by non-experts to find homologous (nucleotide) sequences of all kinds. That makes it an obvious starting point also for sRNA homolog prediction. BLAST was the fastest approach detecting an equal total number of TPs compared to the other two approaches. However, BLAST produced also by far the most FPs and had the by far lowest specificity. The combination of RNAlien and cmsearch reached the highest specificity with all PPVs above 0.98. Considering the whole distribution of diversities for all families, both RNAlien/cmsearch and GLASSgo performed equally well. A major drawback was that RNAlien could not built CMs for 4 families representing 10% of the benchmark sRNAs and hence no homologs could be predicted. Due to the complex workflow, RNAlien/cmsearch has a roughly two orders of magnitude higher runtime than GLASSgo, which hinders large-scale surveys as well as quick analyses for an sRNA of immediate interest. Furthermore, extensive additional bioinformatic work is necessary to use the CMs from RNAlien with cmsearch, which restricts its usefulness for non-expert users.

One of the main goals of GLASSgo was to ease the accessibility for non-expert users. Therefore, we provide a web server which is easy to use and directly displays the predicted homologs ready for downstream analyses. We showed that GLASSgo is only 7.5 times slower than BLAST and has a high specificity which is only slightly lower than the specificity of RNAlien/cmsearch. Furthermore, GLASSgo is as sensitive as the RNAlien/cmsearch combination. The GLASSgo workflow is highly flexible and can be easily adapted by the user. If desired, the sensitivity of GLASSgo can be enhanced without affecting the specificity by re-running the tool with a TP that has a relatively low pairwise identity to the query (<70%) as new input sequence. In cases where highest sensitivity is of interest and specificity is less important, the structural filtering of GLASSgo can be switched off. The BLAST output is then filtered only by the sequence identity to the query, with the additional benefit of the asymmetric sequence extension and the iterative search procedure. To enhance the usability, the synteny analysis presented in this paper (**Figure 4**) as well as direct transfer of the GLASSgo output to the CopraRNA target prediction tool (Wright et al., 2013, 2014) is planned to be implemented in the next version of the web server. It is also worth investigating how the graph-based clustering approach of GLASSgo performs for other classes of structured non-coding RNAs like, e.g., miRNA precursors, riboswitches, or CRISPR repeats, where the information content at sequence level is lower. We are convinced that GLASSgo will boost the high-throughput functional classification of sRNAs. This is of high interest for diverse disciplines, ranging from fundamental research to biotechnology and medicine.

## Author Contributions

BV, SL, WH, and JG designed the study. SL implemented the tool. MM implemented the webserver. RS implemented the taxonomy visualization. SL and RS performed the benchmark. BV, SL, and JG analyzed the data. JG did the synteny analysis. SL and JG prepared the figures. All the authors were involved in writing and quality control of the manuscript.

## Conflict of Interest Statement

The authors declare that the research was conducted in the absence of any commercial or financial relationships that could be construed as a potential conflict of interest.

## Footnotes

1 http://rna.informatik.uni-freiburg.de/GLASSgo/

2 https://github.com/lotts/GLASSgo_GI-Version

3 https://hub.docker.com/r/lotts/glassgo_gi_version/

4 https://github.com/eggzilla/RNAlien

5 https://pypi.python.org/pypi/speedtest-cli/

6 http://rna.informatik.uni-freiburg.de/GLASSgo/

## Supplementary Material

FIGURE S1 The GLASSgo algorithm. First, GLASSgo conducts a low stringency BLASTn (default *E*-value = 1) search with a single sRNA sequence query against the NCBI nucleotide collection (step 1). The BLAST search can be restricted to specific taxonomic domains or phyla. Short local BLAST hits are extended, based on the pairwise local BLAST alignment to match the length of the query (step 2). Hits with a global pairwise sequence identity (PI) to the query that is greater or equal to a given threshold (default PI: 52%) are considered for the following steps (3). Next, the sequences are grouped based on their PI (step 4). Hits with a *PI > 70%* are directly stored and considered as true positives (TPs). Sequences with a PI between the lower PI threshold and 70% are considered as candidate homologs and analyzed together with the true positive hits by the structure-based filter module via graph-based clustering. The true positive hits serve as references for positive clusters. Hits between 65 and 80% are candidates for re-blasting, to increase the sensitivity of GLASSgo. A set of up to 100 hits is selected from these candidates (step 5). From hits with a PI in the intervals (65, 70) and (70, 80) we select up to 45 each and from the interval (80, 100) we take up to 10 hits. This set forms the query for a new round of BLAST searching, sequence extension and PI based filtering (steps 1–4). The repeated blasting with up to 100 new queries potentially generates a high number of duplicated sequences, which are removed (step 6) before the sequences are pre-clustered based on their pairwise sequence identity (step 7) to generate sequence similarity based clusters (cluster_seq_) (step 8). This step reduces the runtime and prevents the existence of too similar leaves in the resulting tree, which could interfere with the automatic scaling procedure of Londen. One representative of each cluster_seq_ is used to compute pairwise distances based on the RNA secondary structure ensembles using RNApdist (Hofacker et al., 1994) (step 9). The resulting distance matrix is used by Londen to construct a neighbor joining tree (NJ), which is clipped by an auto-adaptive procedure to generate structure based clusters (cluster_struc_). All cluster_struc_ that include the input sequence or a true positive hit are considered as positive clusters and retained (steps 10 + 11). If a representative of a cluster_seq_ is part of a positive cluster_struc_, all other members of this cluster_seq_ are automatically considered as positives (step 12). Sequences confirmed by Londen and true positive hits are merged together and constitute the result of GLASSgo (step 13). The output is a FASTA file with all predicted homologs and a taxonomic tree of the respective organisms.

FIGURE S2 Mafft multiple sequence alignment of selected RF00111 homologs including partial promoter sequences. The sequence of RF00111/SdsR is boxed in red. Homologs with are likely transferred by a phage are boxed in black. Sequence “a” is classified as TP by cmscan. Sequence “b” with only one base difference to sequence “a” is classified as FP by cmscan.

FIGURE S3 Visualization of the sequence based pre-clustering of the BLAST hits prior to the structure based clustering.

DATASHEET S1 This zip folder contains four subfolders ‘main_test_cases-0_raw_data’, ‘main_test_cases-1_cm_evaluated_raw_data’, ‘additional_test_cases-0_raw_data’ and ‘additional_test_cases-1_cm_evaluated_raw_data’. In each folder with the prefix ‘main_test_cases’, there are three files, one represents the BLAST analysis, one the GLASSgo analysis and one the RNAlien analysis. Instead, the folders with the prefix ‘additional_test_cases’ contain only one file. All files have the same underlying data format. Each sub result starts with its relating RFAM ID, e.g., ‘#RF00111’ followed by the data in FASTA format. The first entry after the given RFAM ID represents the query sequence. This sequence was used to perform the search/prediction and at the end of an FASTA header, the taxonomic ID was introduced for the BLAST, GLASSgo as well as the additional test cases. For the GLASSgo, RNAlien/CMscan as well as the additional test cases, the pairwise sequence identity related to the query sequence is available, e.g., ‘p.c.VAL:98.02%’. All files in the ‘^∗^-0_raw_data’ folder are unfiltered and represents the search/prediction results, containing false as well as true positives. The files in folder ‘^∗^-1_cm_evaluated_raw_data’ were evaluated with cmscan and each sequence was either classified as TP (CM-MODEL_TRUE) or as FP (CM-MODEL_FALSE).

DATASHEET S2 (Sheet 1) Benchmark results of the three compared approaches for each of the 40 sRNA families. The number of true positives (TPs) and false positives (FPs) is given with regard to our verification pipeline for GLASSgo, RNAlien/cmsearch and BLAST. The specificity is expressed as the positive predictive value (PPV). The run-time for each analysis on a single CPU is given in seconds (time). For RNAlien/cmsearch, the run-times for the CM generation by RNAlien (time Alien) and for the library scan with cmsearch (time cms) are given separately and combined (time comb.) We used the pairwise diversity of all detected TP homologs to the query as a proxy for the sensitivity of the tools. Therefore, we calculated the maximal pairwise diversity to the query sequence (mPD), the mean pairwise diversity to the query sequence (mean PD) and the median pairwise diversity to the query sequence (median PD). (Sheet 2) Results of the GLASSgo sRNA homolog prediction for 15 additional sRNA families which were used to test the adjusted parameters of GLASSgo.

DATASHEET S3 Synteny analysis of all homologs of RF00111/SdsR that are predicted by GLASSgo. All genome regions are centered around the predicted RF00111/SdsR homolog drawn in red. Homologous genes have matching background colors. Genes without homologs are colored gray and genes without amino acid sequence information in the genbank file (e.g., pseudo-genes) are white.

## References

[B1] Altschul S. F. Gish W. Miller W. Myers E. W. Lipman D. J. (1990). Basic local alignment search tool. *J. Mol. Biol.* 215 403–410. 10.1016/S0022-2836(05)80360-2 2231712

[B2] Barquist L. Lars B. Jörg V. (2015). Accelerating discovery and functional analysis of small RNAs with new technologies. *Annu. Rev. Genet.* 49 367–394. 10.1146/annurev-genet-112414-054804 26473381

[B3] Bernhart S. H. Hofacker I. L. Will S. Gruber A. R. Stadler P. F. (2008). RNAalifold: improved consensus structure prediction for RNA alignments. *BMC Bioinformatics* 9:474. 10.1186/1471-2105-9-474 19014431 PMC2621365

[B4] Bonhoeffer S. McCaskill J. S. Stadler P. F. Schuster P. (1993). RNA multi-structure landscapes. A study based on temperature dependent partition functions. *Eur. Biophys. J.* 22 13–24. 7685689 10.1007/BF00205808

[B5] Eggenhofer F. Hofacker I. L. Höner Zu Siederdissen C. (2016). RNAlien - Unsupervised RNA family model construction. *Nucleic Acids Res.* 44 8433–8441. 10.1093/nar/gkw558 27330139 PMC5041467

[B6] Freyhult E. K. Bollback J. P. Gardner P. P. (2006). Exploring genomic dark matter: a critical assessment of the performance of homology search methods on noncoding RNA. *Genome Res.* 17 117–125. 10.1101/gr.5890907 17151342 PMC1716261

[B7] Fröhlich K. S. Papenfort K. Berger A. A. Vogel J. (2012). A conserved RpoS-dependent small RNA controls the synthesis of major porin OmpD. *Nucleic Acids Res.* 40 3623–3640. 10.1093/nar/gkr1156 22180532 PMC3333887

[B8] Gotoh O. (1982). An improved algorithm for matching biological sequences. *J. Mol. Biol.* 162 705–708. 10.1016/0022-2836(82)90398-9 7166760

[B9] Gruber A. R. Findeiß S. Washietl S. Hofacker I. L. Stadler P. F. (2009). RNAz 2.0: improved noncoding RNA detection. *Pac. Symp. Biocomput.* 15 69–79. 10.1142/9789814295291_0009 19908359

[B10] Hofacker I. L. Fontana W. Stadler P. F. Bonhoeffer L. S. Tacker M. Schuster P. (1994). Fast folding and comparison of RNA secondary structures. *Monatsh. Chem. Chem. Mon.* 125 167–188. 10.1007/BF00818163

[B11] Horler R. S. P. Vanderpool C. K. (2009). Homologs of the small RNA SgrS are broadly distributed in enteric bacteria but have diverged in size and sequence. *Nucleic Acids Res.* 37 5465–5476. 10.1093/nar/gkp501 19531735 PMC2760817

[B12] Huang Y. Niu B. Gao Y. Fu L. Li W. (2010). CD-HIT Suite: a web server for clustering and comparing biological sequences. *Bioinformatics* 26 680–682. 10.1093/bioinformatics/btq003 20053844 PMC2828112

[B13] Huelsenbeck J. P. (1995). Performance of phylogenetic methods in simulation. *Syst. Biol.* 44 17–48. 10.1093/sysbio/44.1.17

[B14] Katoh K. Toh H. (2008). Improved accuracy of multiple ncRNA alignment by incorporating structural information into a MAFFT-based framework. *BMC Bioinformatics* 9:212. 10.1186/1471-2105-9-212 18439255 PMC2387179

[B15] Lagares A. Jr. Roux I. Valverde C. (2016). Phylogenetic distribution and evolutionary pattern of an α-proteobacterial small RNA gene that controls polyhydroxybutyrate accumulation in *Sinorhizobium meliloti*. *Mol. Phylogenet. Evol.* 99 182–193. 10.1016/j.ympev.2016.03.026 27033949

[B16] Lindgreen S. Umu S. U. Lai A. S.-W. Eldai H. Liu W. McGimpsey S. (2014). Robust identification of noncoding RNA from transcriptomes requires phylogenetically-informed sampling. *PLoS Comput. Biol.* 10:e1003907. 10.1371/journal.pcbi.1003907 25357249 PMC4214555

[B17] Lorenz R. Ronny L. Bernhart S. H. zu Siederdissen C. H. Hakim T. Christoph F. (2011). ViennaRNA package 2.0. *Algorithms Mol. Biol.* 6:26. 10.1186/1748-7188-6-26 22115189 PMC3319429

[B18] Lott S. C. Wolfien M. Riege K. Bagnacani A. Wolkenhauer O. Hoffmann S. (2017). Customized workflow development and data modularization concepts for RNA-sequencing and metatranscriptome experiments. *J. Biotechnol.* 261 85–96. 10.1016/j.jbiotec.2017.06.1203 28676233

[B19] Madera M. Gough J. (2002). A comparison of profile hidden Markov model procedures for remote homology detection. *Nucleic Acids Res.* 30 4321–4328. 10.1093/nar/gkf544 12364612 PMC140544

[B20] Menzel P. Gorodkin J. Stadler P. F. (2009). The tedious task of finding homologous noncoding RNA genes. *RNA* 15 2075–2082. 10.1261/rna.1556009 19861422 PMC2779685

[B21] Nawrocki E. P. Burge S. W. Bateman A. Daub J. Eberhardt R. Y. Eddy S. R. (2015). Rfam 12.0: updates to the RNA families database. *Nucleic Acids Res.* 43 D130–D137. 10.1093/nar/gku1063 25392425 PMC4383904

[B22] Nawrocki E. P. Eddy S. R. (2013a). Computational identification of functional RNA homologs in metagenomic data. *RNA Biol.* 10 1170–1179. 10.4161/rna.25038 23722291 PMC3849165

[B23] Nawrocki E. P. Eddy S. R. (2013b). Infernal 1.1: 100-fold faster RNA homology searches. *Bioinformatics* 29 2933–2935. 10.1093/bioinformatics/btt509 24008419 PMC3810854

[B24] Reinkensmeier J. Schlüter J.-P. Giegerich R. Becker A. (2011). Conservation and occurrence of trans-encoded sRNAs in the Rhizobiales. *Genes* 2 925–956. 10.3390/genes2040925 24710299 PMC3927594

[B25] Saito T. Imanishi N. (1989). Relative efficiencies of the Fitch-Margoliash, maximum-parsimony, maximum-likelihood, minimum-evolution, and neighbor-joining methods of phylogenetic tree construction in obtaining the correct tree. *Mol. Biol. Evol.* 6 514–525. 10.1093/oxfordjournals.molbev.a040572

[B26] Sievers F. Wilm A. Dineen D. Gibson T. J. Karplus K. Li W. (2011). Fast, scalable generation of high-quality protein multiple sequence alignments using Clustal Omega. *Mol. Syst. Biol.* 7:539. 10.1038/msb.2011.75 21988835 PMC3261699

[B27] Smith C. Heyne S. Richter A. S. Will S. Backofen R. (2010). Freiburg RNA Tools: a web server integrating INTARNA, EXPARNA and LOCARNA. *Nucleic Acids Res.* 38 W373–W377. 10.1093/nar/gkq316 20444875 PMC2896085

[B28] Wagner E. G. H. Romby P. (2015). Small RNAs in bacteria and archaea: who they are, what they do, and how they do it. *Adv. Genet.* 90 133–208. 10.1016/bs.adgen.2015.05.001 26296935

[B29] Washietl S. Findeiss S. Müller S. A. Kalkhof S. von Bergen M. Hofacker I. L. (2011). RNAcode: robust discrimination of coding and noncoding regions in comparative sequence data. *RNA* 17 578–594. 10.1261/rna.2536111 21357752 PMC3062170

[B30] Wright P. R. Georg J. Mann M. Sorescu D. A. Richter A. S. Lott S. (2014). CopraRNA and IntaRNA: predicting small RNA targets, networks and interaction domains. *Nucleic Acids Res.* 42 W119–W123. 10.1093/nar/gku359 24838564 PMC4086077

[B31] Wright P. R. Richter A. S. Papenfort K. Mann M. Vogel J. Hess W. R. (2013). Comparative genomics boosts target prediction for bacterial small RNAs. *Proc. Natl. Acad. Sci. U.S.A.* 110 E3487–E3496. 10.1073/pnas.1303248110 23980183 PMC3773804

[B32] Zahn C. T. (1971). Graph-theoretical methods for detecting and describing gestalt clusters. *IEEE Trans. Comput.* C20 68–86. 10.1109/T-C.1971.223083

