# Supplementary figures and images for: GLASSgo – Automated and Reliable Detection of sRNA Homologs From a Single Input Sequence
Source: Front Genet. 2018 Apr 17;9:124. doi: 10.3389/fgene.2018.00124 (PMC5913331; doi:10.3389/fgene.2018.00124)

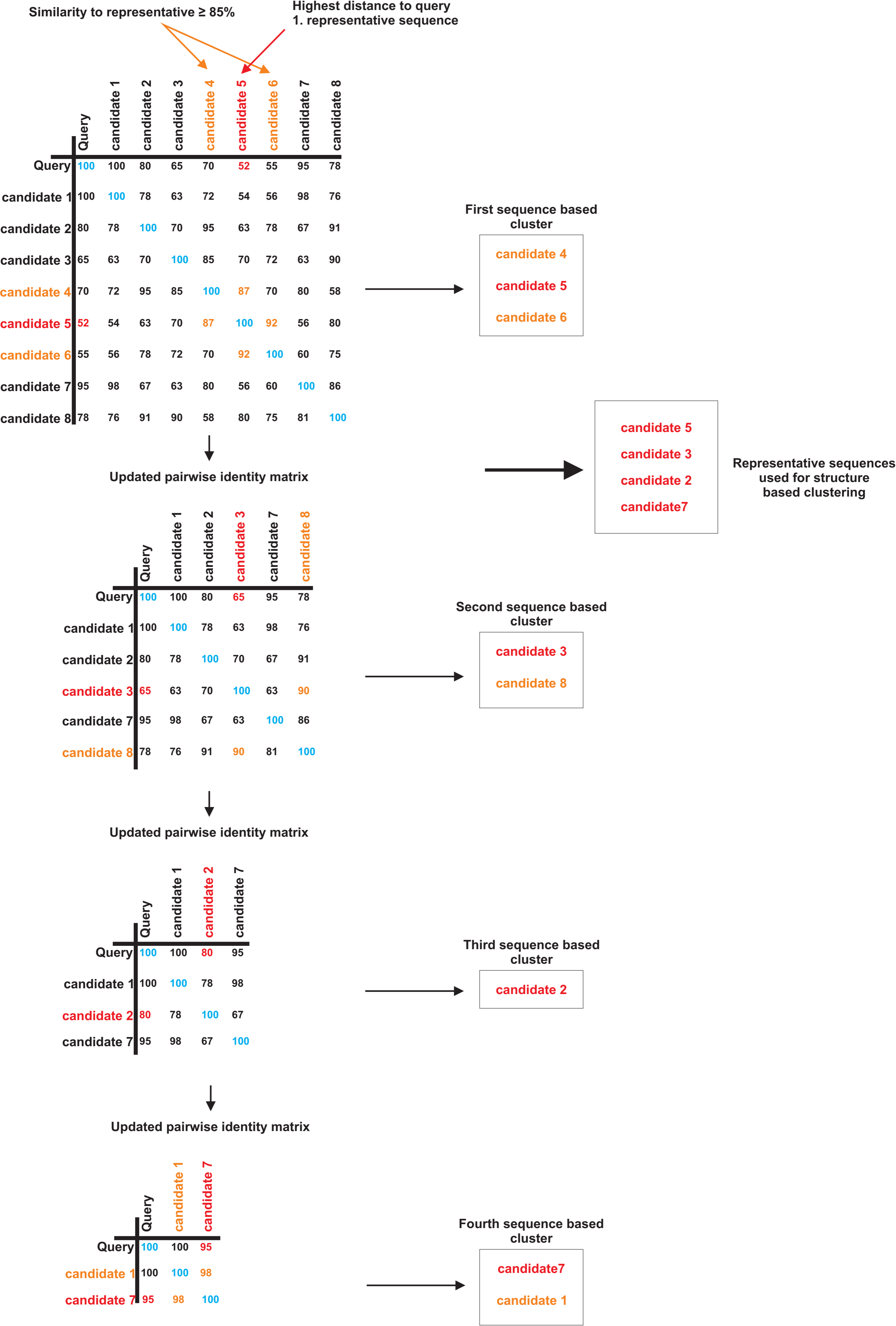

Supplement: FIGURE S3 — Visualization of the sequence based pre-clustering of the BLAST hits prior to the structure based clustering. [file Image_3.PDF]
