# Supplementary material for: GLASSgo – Automated and Reliable Detection of sRNA Homologs From a Single Input Sequence
Source: Front Genet. 2018 Apr 17;9:124. doi: 10.3389/fgene.2018.00124 (PMC5913331; doi:10.3389/fgene.2018.00124)
Supplement: DATASHEET S3 — Synteny analysis of all homologs of RF00111/SdsR that are predicted by GLASSgo. All genome regions are centered around the predicted RF00111/SdsR homolog drawn in red. Homologous genes have matching background colors. Genes without homologs are colored gray and genes without amino acid sequence information in the genbank file (e.g., pseudo-genes) are white. [file Data_Sheet_3.PDF]

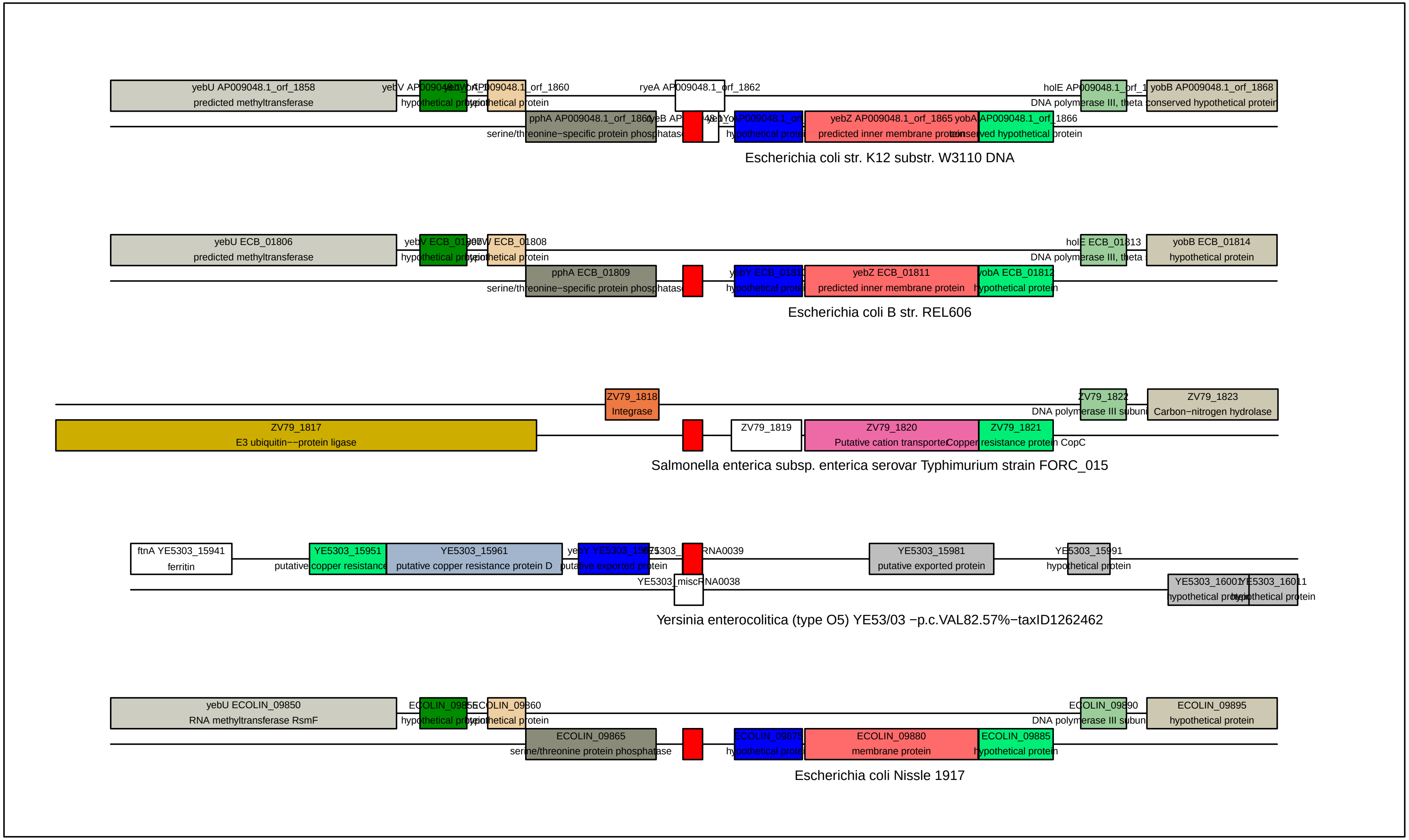
yebU AP009048.1_orf_1858
predicted methyltransferase
ryeA AP009048.1_orf_1862
pphA AP009048.1_orf_1861
serine/threonine−specific protein phosphatase
yebZ AP009048.1_orf_1865
predicted inner membrane protein
conserved hypothetical protein
yobB AP009048.1_orf_1868
Escherichia coli str. K12 substr. W3110 DNA
yebU ECB_01806
predicted methyltransferase
pphA ECB_01809
yebZ ECB_01811
predicted inner membrane protein
yobA ECB_01812
hypothetical protein
yobB ECB_01814
Escherichia coli B str. REL606
ZV79_1818
Integrase
ZV79_1822
ZV79_1823
Carbon−nitrogen hydrolase
ZV79_1817
E3 ubiquitin−−protein ligase
ZV79_1819
ZV79_1820
Putative cation transporter
ZV79_1821
Copper resistance protein CopC
Salmonella enterica subsp. enterica serovar Typhimurium strain FORC_015
ftnA YE5303_15941
ferritin
YE5303_15951
YE5303_15961
putative copper resistance protein D
putative exported protein
YE5303_15981
putative exported protein
YE5303_15991
hypothetical protein
YE5303_miscRNA0038
Yersinia enterocolitica (type O5) YE53/03 −p.c.VAL82.57%−taxID1262462
yebU ECOLIN_09850
RNA methyltransferase RsmF
ECOLIN_09865
serine/threonine protein phosphatase
ECOLIN_09880
membrane protein
ECOLIN_09885
hypothetical protein
ECOLIN_09895
hypothetical protein
Escherichia coli Nissle 1917

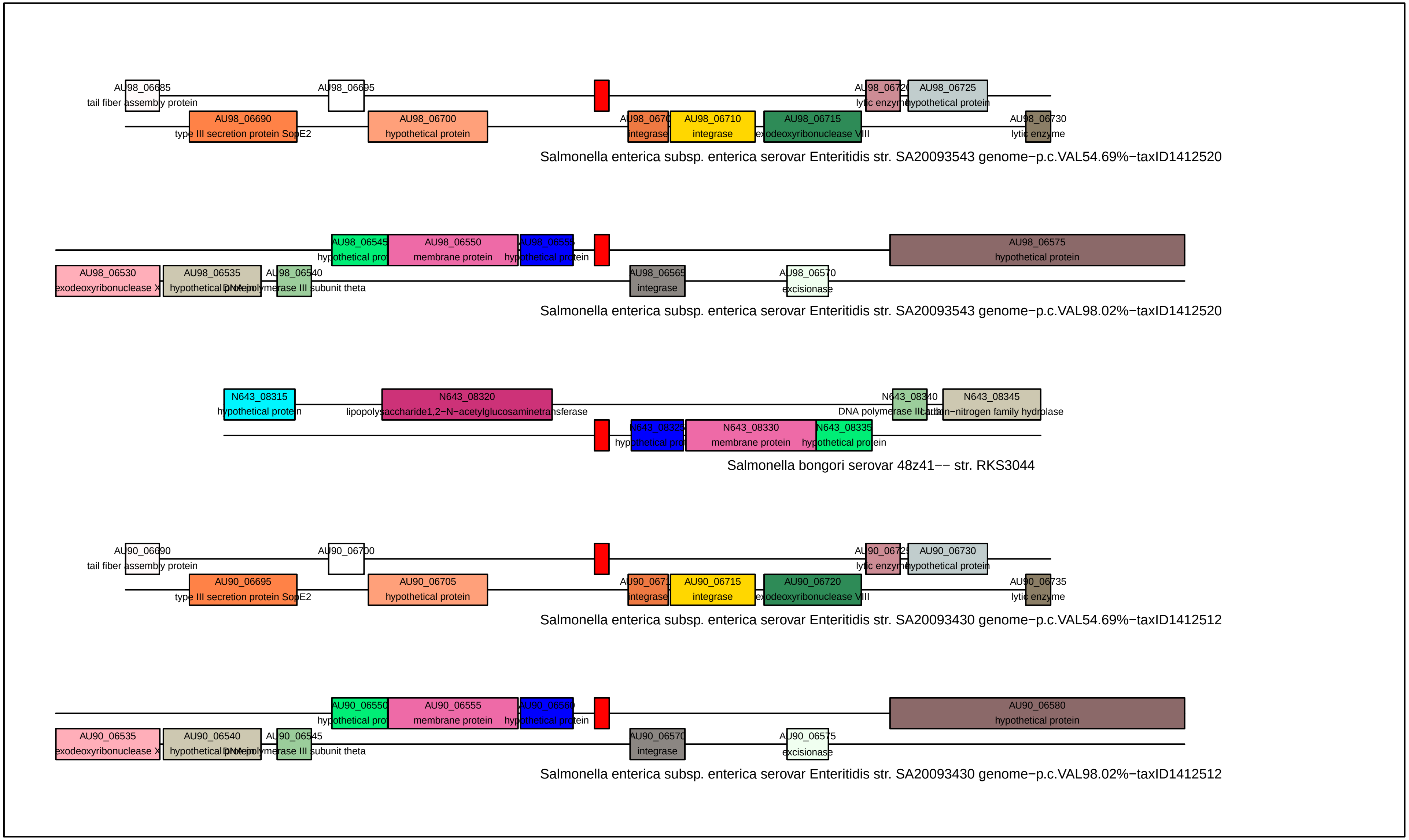

AU98_06685
tail fiber assembly protein
AU98_06695
AU98_06690
type III secretion protein SopE2
AU98_06700
hypothetical protein
integrase
AU98_06710
integrase
AU98_06715
exodeoxyribonuclease VIII
lytic enzyme
AU98_06725
hypothetical protein
AU98_06730
lytic enzyme
Salmonella enterica subsp. enterica serovar Enteritidis str. SA20093543 genome−p.c.VAL54.69%−taxID1412520
AU98_06545
AU98_06550
membrane protein
hypothetical protein
AU98_06575
hypothetical protein
AU98_06530
exodeoxyribonuclease X
AU98_06535
AU98_06540
DNA polymerase III subunit theta
AU98_06565
integrase
AU98_06570
excisionase
Salmonella enterica subsp. enterica serovar Enteritidis str. SA20093543 genome−p.c.VAL98.02%−taxID1412520
N643_08315
hypothetical protein
N643_08320
lipopolysaccharide1,2−N−acetylglucosaminetransferase
N643_08340
N643_08345
N643_08330
membrane protein
N643_08335
hypothetical protein
Salmonella bongori serovar 48z41−− str. RKS3044
AU90_06690
tail fiber assembly protein
AU90_06700
AU90_06695
type III secretion protein SopE2
AU90_06705
hypothetical protein
integrase
AU90_06715
integrase
AU90_06720
exodeoxyribonuclease VIII
lytic enzyme
AU90_06730
hypothetical protein
AU90_06735
lytic enzyme
Salmonella enterica subsp. enterica serovar Enteritidis str. SA20093430 genome−p.c.VAL54.69%−taxID1412512
AU90_06550
AU90_06555
membrane protein
hypothetical protein
AU90_06580
hypothetical protein
AU90_06535
exodeoxyribonuclease X
AU90_06540
AU90_06545
DNA polymerase III subunit theta
AU90_06570
integrase
AU90_06575
excisionase
Salmonella enterica subsp. enterica serovar Enteritidis str. SA20093430 genome−p.c.VAL98.02%−taxID1412512

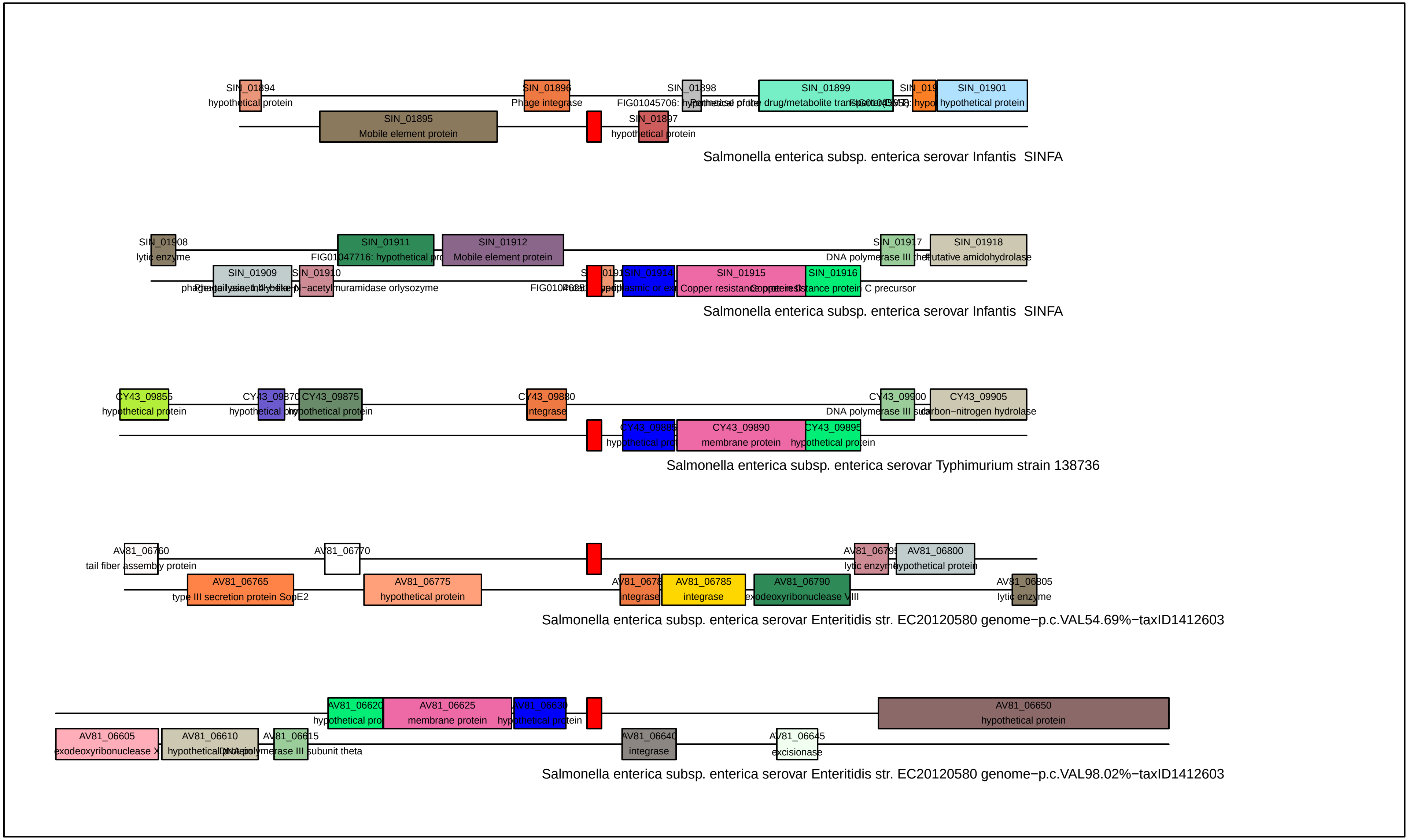

SIN_01894
hypothetical protein
SIN_01895
Mobile element protein
SIN_01896
Phage integrase
SIN_01897
hypothetical protein
SIN_01898
SIN_01899
SIN_01901
hypothetical protein
Salmonella enterica subsp. enterica serovar Infantis SINFA
SIN_01908
lytic enzyme
SIN_01909
SIN_01910
N−acetylmuramidase orlysozyme
SIN_01911
SIN_01912
Mobile element protein
SIN_01914
SIN_01915
SIN_01916
C precursor
SIN_01917
DNA polymerase III
SIN_01918
Putative amidohydrolase
Salmonella enterica subsp. enterica serovar Infantis SINFA
CY43_09855
hypothetical protein
CY43_09875
hypothetical protein
CY43_09880
integrase
CY43_09890
membrane protein
CY43_09895
hypothetical protein
CY43_09900
DNA polymerase III
CY43_09905
carbon−nitrogen hydrolase
Salmonella enterica subsp. enterica serovar Typhimurium strain 138736
AV81_06760
tail fiber assembly protein
AV81_06765
type III secretion protein SopE2
AV81_06770
AV81_06775
hypothetical protein
AV81_06785
integrase
AV81_06790
exodeoxyribonuclease VIII
AV81_06800
hypothetical protein
AV81_06805
lytic enzyme
Salmonella enterica subsp. enterica serovar Enteritidis str. EC20120580 genome−p.c.VAL54.69%−taxID1412603
AV81_06605
exodeoxyribonuclease X
AV81_06610
AV81_06615
subunit theta
AV81_06620
AV81_06625
membrane protein
AV81_06640
integrase
AV81_06645
excisionase
AV81_06650
hypothetical protein
Salmonella enterica subsp. enterica serovar Enteritidis str. EC20120580 genome−p.c.VAL98.02%−taxID1412603

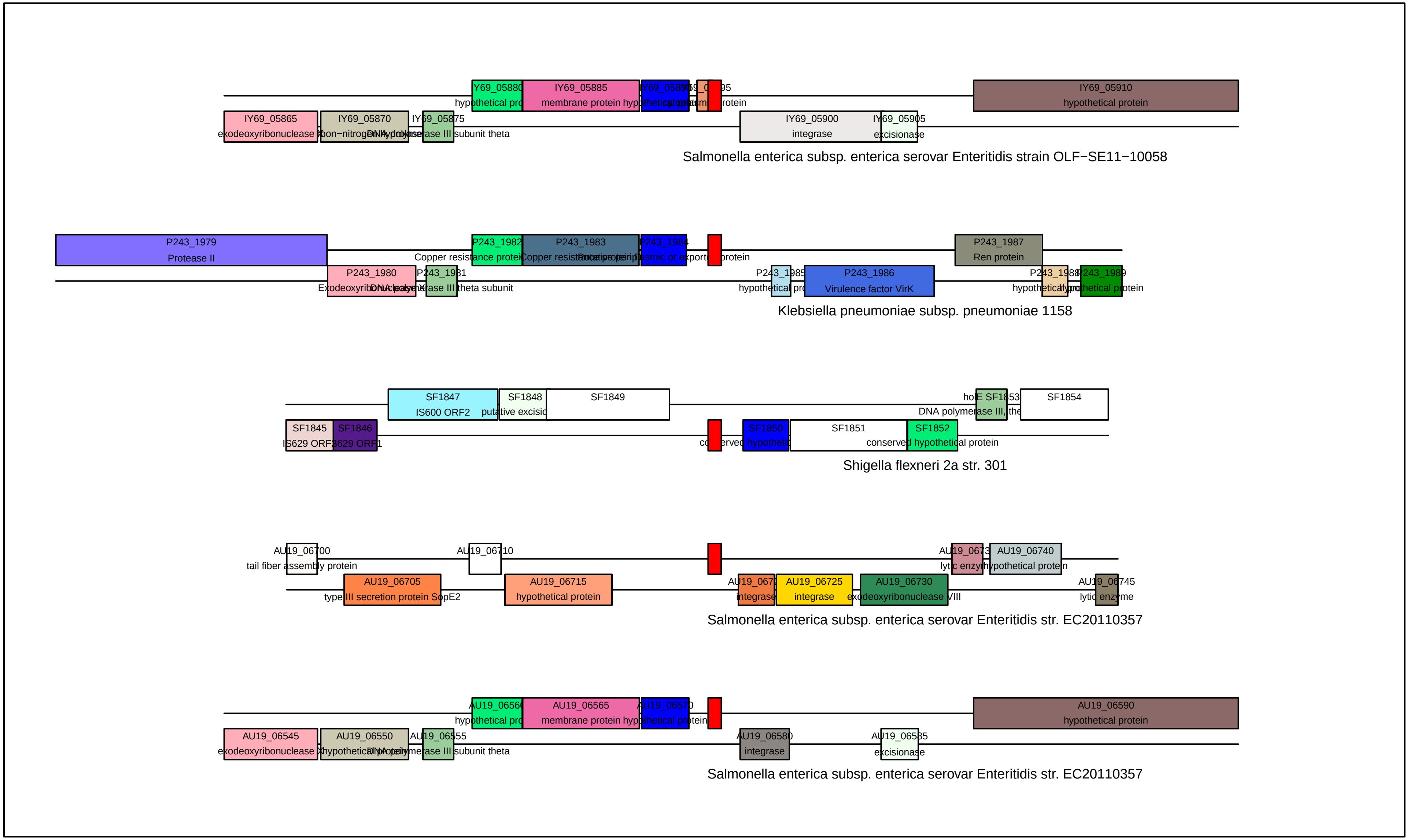

IY69_05865
exodeoxyribonuclease X
IY69_05870
IY69_05875
subunit theta
hypothetical protein
IY69_05885
membrane protein
IY69_05900
integrase
IY69_05905
excisionase
IY69_05910
hypothetical protein
Salmonella enterica subsp. enterica serovar Enteritidis strain OLF−SE11−10058
P243_1979
Protease II
P243_1980
P243_1981
theta subunit
P243_1982
P243_1983
P243_1984
P243_1985
P243_1986
Virulence factor VirK
P243_1987
Ren protein
P243_1988
P243_1989
hypothetical protein
Klebsiella pneumoniae subsp. pneumoniae 1158
SF1845
SF1846
SF1847
IS600 ORF2
SF1848
SF1849
SF1850
SF1851
SF1852
conserved hypothetical protein
SF1853
SF1854
Shigella flexneri 2a str. 301
AU19_06700
tail fiber assembly protein
AU19_06705
type III secretion protein SopE2
AU19_06710
AU19_06715
hypothetical protein
AU19_06725
integrase
AU19_06730
exodeoxyribonuclease VIII
AU19_06740
hypothetical protein
AU19_06745
lytic enzyme
Salmonella enterica subsp. enterica serovar Enteritidis str. EC20110357
AU19_06545
exodeoxyribonuclease X
AU19_06550
AU19_06555
subunit theta
AU19_06565
membrane protein
hypothetical protein
AU19_06580
integrase
AU19_06585
excisionase
AU19_06590
hypothetical protein
Salmonella enterica subsp. enterica serovar Enteritidis str. EC20110357

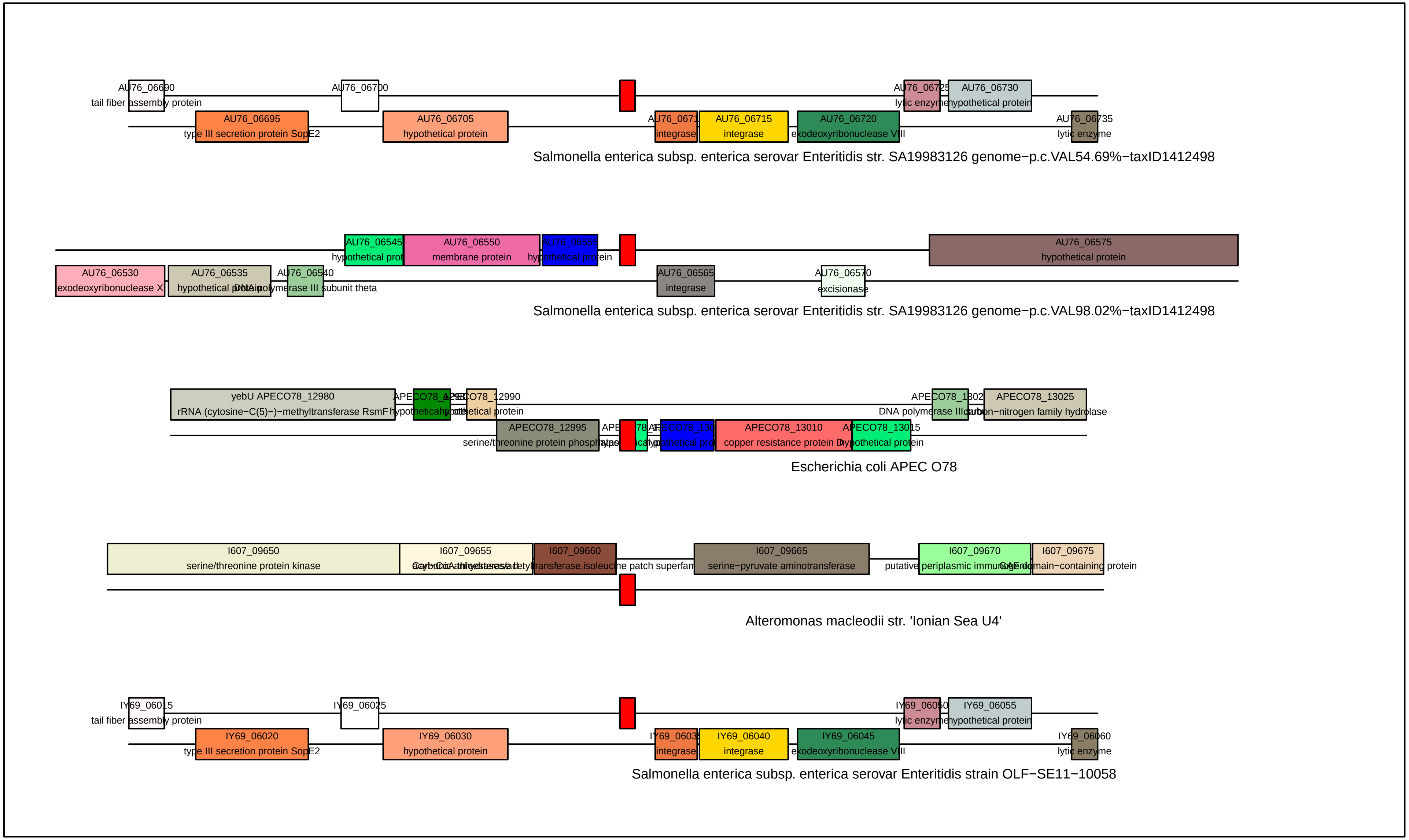

AU76_06690
tail fiber assembly protein
AU76_06700
AU76_06695
type III secretion protein SopE2
AU76_06705
hypothetical protein
integrase
AU76_06715
integrase
AU76_06720
exodeoxyribonuclease VIII
AU76_06725
lytic enzyme
AU76_06730
hypothetical protein
AU76_06735
lytic enzyme
Salmonella enterica subsp. enterica serovar Enteritidis str. SA19983126 genome−p.c.VAL54.69%−taxID1412498
AU76_06545
AU76_06550
membrane protein
hypothetical protein
AU76_06575
hypothetical protein
AU76_06530
exodeoxyribonuclease X
AU76_06535
hypothetical protein
DNA polymerase III subunit theta
AU76_06540
AU76_06565
integrase
AU76_06570
excisionase
Salmonella enterica subsp. enterica serovar Enteritidis str. SA19983126 genome−p.c.VAL98.02%−taxID1412498
yebU APECO78_12980
rRNA (cytosine−C(5)−)−methyltransferase RsmF
APECO78_12990
hypothetical protein
APECO78_12995
serine/threonine protein phosphatase
APECO78_13010
copper resistance protein D
APECO78_13015
hypothetical protein
DNA polymerase III
APECO78_13025
carbon−nitrogen family hydrolase
Escherichia coli APEC O78
I607_09650
serine/threonine protein kinase
I607_09655
I607_09660
I607_09665
serine−pyruvate aminotransferase
I607_09670
putative periplasmic immunogenic
I607_09675
domain−containing protein
Alteromonas macleodii str. 'Ionian Sea U4'
IY69_06015
tail fiber assembly protein
IY69_06025
IY69_06020
type III secretion protein SopE2
IY69_06030
hypothetical protein
integrase
IY69_06040
integrase
IY69_06045
exodeoxyribonuclease VIII
IY69_06050
lytic enzyme
IY69_06055
hypothetical protein
IY69_06060
lytic enzyme
Salmonella enterica subsp. enterica serovar Enteritidis strain OLF−SE11−10058

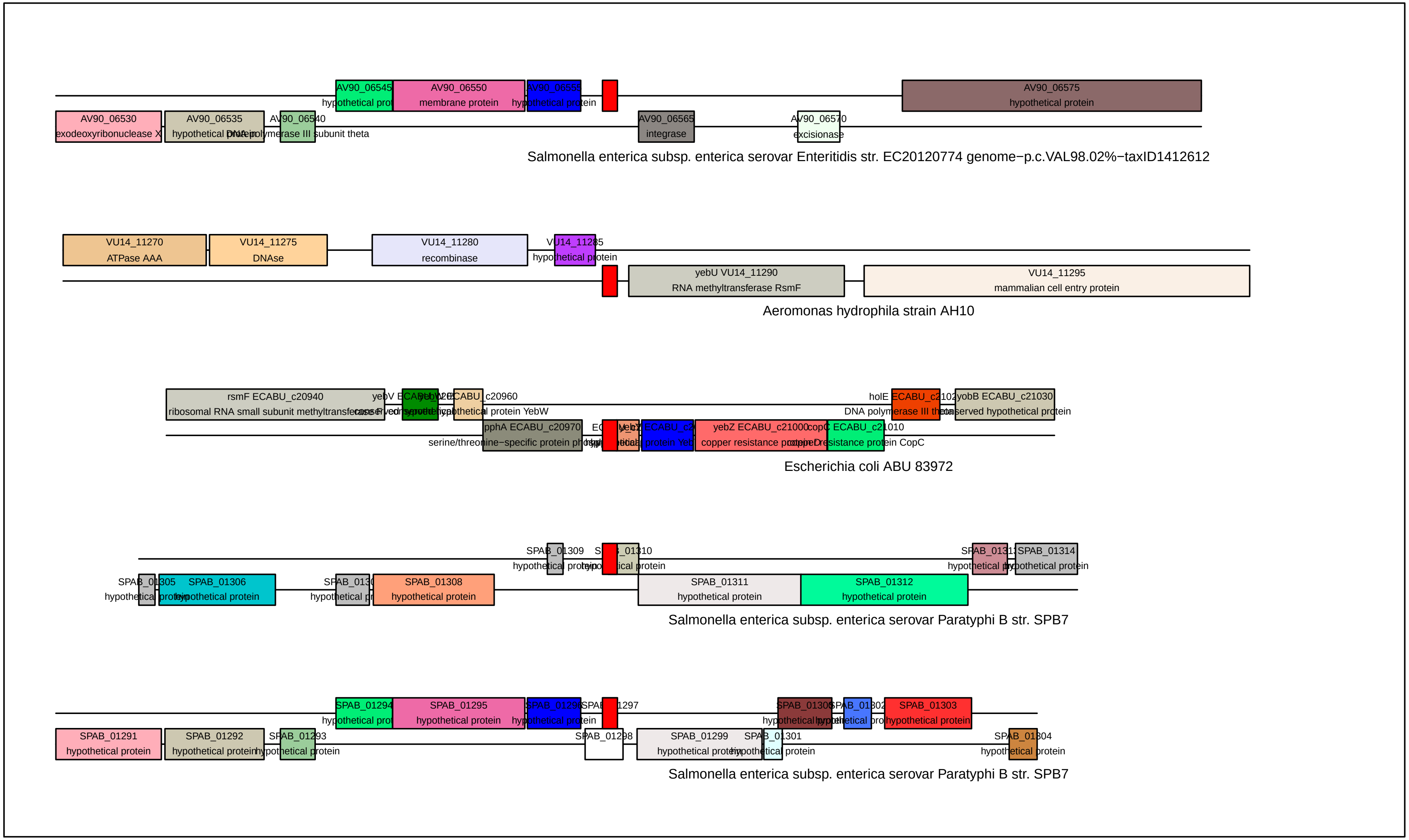
AV90_06545
AV90_06550
membrane protein
AV90_06575
hypothetical protein
AV90_06530
exodeoxyribonuclease X
AV90_06535
AV90_06540
DNA polymerase III subunit theta
AV90_06565
integrase
AV90_06570
excisionase
Salmonella enterica subsp. enterica serovar Enteritidis str. EC20120774 genome−p.c.VAL98.02%−taxID1412612
VU14_11270
ATPase AAA
VU14_11275
DNAse
VU14_11280
recombinase
VU14_11285
hypothetical protein
yebU VU14_11290
RNA methyltransferase RsmF
VU14_11295
mammalian cell entry protein
Aeromonas hydrophila strain AH10
rsmF ECABU_c20940
ribosomal RNA small subunit methyltransferase F
hypothetical protein YebW
holE ECABU_c2102
DNA polymerase III theta
yobB ECABU_c21030
conserved hypothetical protein
pphA ECABU_c20970
serine/threonine−specific protein phosphatase
yebZ ECABU_c21000
copper resistance protein D
copC ECABU_c21010
copper resistance protein CopC
Escherichia coli ABU 83972
SPAB_01309
SPAB_01310
hypothetical protein
SPAB_01314
SPAB_01305
SPAB_01306
hypothetical protein
SPAB_01308
hypothetical protein
SPAB_01311
hypothetical protein
SPAB_01312
hypothetical protein
Salmonella enterica subsp. enterica serovar Paratyphi B str. SPB7
SPAB_01294
SPAB_01295
hypothetical protein
SPAB_01297
SPAB_01300
SPAB_01302
SPAB_01303
hypothetical protein
SPAB_01291
hypothetical protein
SPAB_01292
hypothetical protein
SPAB_01293
hypothetical protein
SPAB_01298
SPAB_01299
SPAB_01301
hypothetical protein
SPAB_01304
hypothetical protein
Salmonella enterica subsp. enterica serovar Paratyphi B str. SPB7

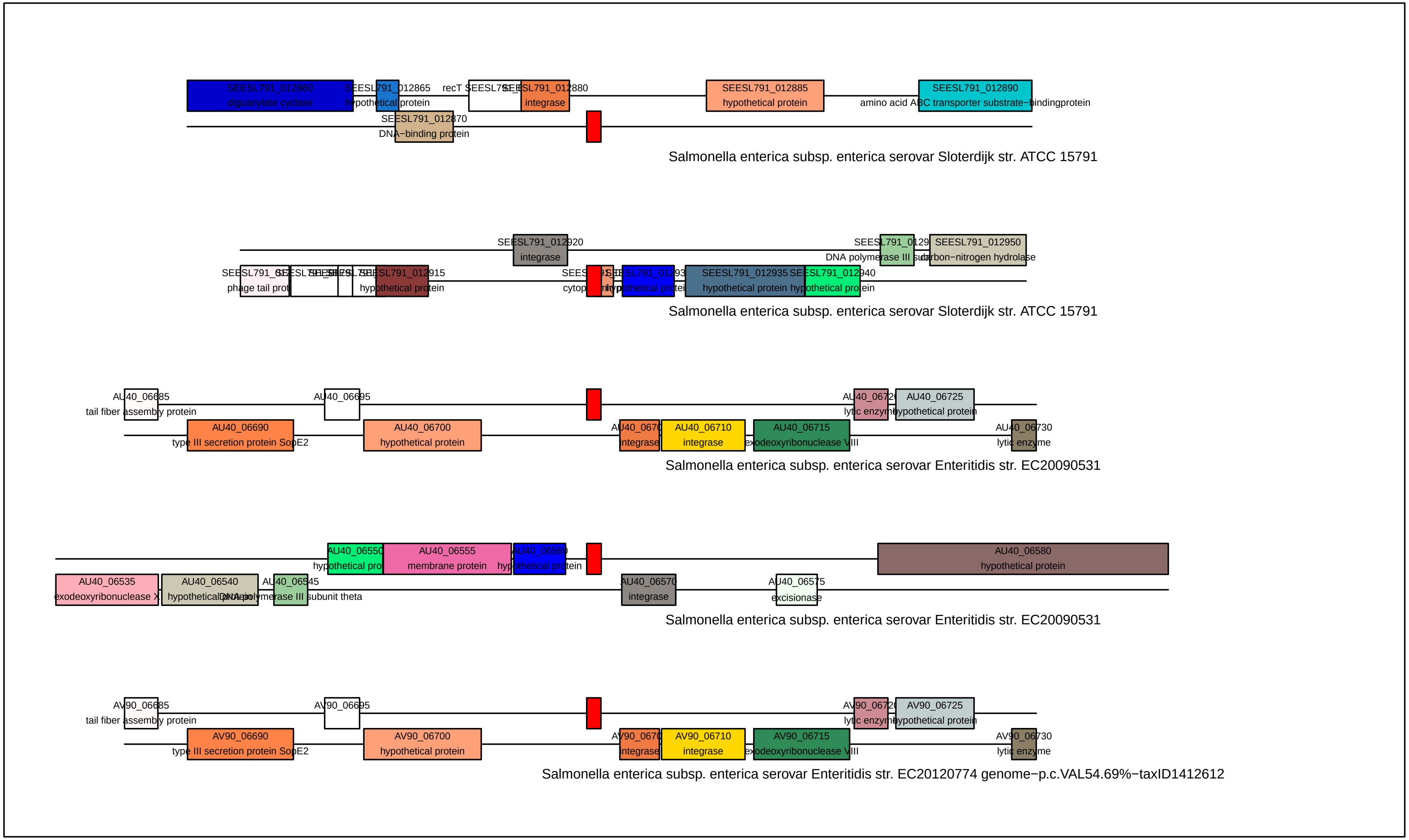

SEESL791_012860
diguanylate cyclase
SEESL791_012865
hypothetical protein
recT
SEESL791_012880
integrase
SEESL791_012885
hypothetical protein
SEESL791_012890
amino acid ABC transporter substrate−bindingprotein
SEESL791_012870
DNA−binding protein
Salmonella enterica subsp. enterica serovar Sloterdijk str. ATCC 15791
SEESL791_012920
integrase
SEESL791_012950
carbon−nitrogen hydrolase
DNA polymerase III
SEESL791_012915
hypothetical protein
phage tail prot
SEESL791_012935
hypothetical protein
SEESL791_012940
hypothetical protein
Salmonella enterica subsp. enterica serovar Sloterdijk str. ATCC 15791
AU40_06685
tail fiber assembly protein
AU40_06695
AU40_06725
hypothetical protein
AU40_06690
type III secretion protein SopE2
AU40_06700
hypothetical protein
integrase
AU40_06710
integrase
AU40_06715
exodeoxyribonuclease VIII
AU40_06730
lytic enzyme
Salmonella enterica subsp. enterica serovar Enteritidis str. EC20090531
membrane protein
AU40_06555
AU40_06580
hypothetical protein
AU40_06535
exodeoxyribonuclease X
AU40_06540
AU40_06570
integrase
AU40_06575
excisionase
subunit theta
Salmonella enterica subsp. enterica serovar Enteritidis str. EC20090531
AV90_06685
tail fiber assembly protein
AV90_06695
AV90_06725
hypothetical protein
AV90_06690
type III secretion protein SopE2
AV90_06700
hypothetical protein
integrase
AV90_06710
integrase
AV90_06715
exodeoxyribonuclease VIII
AV90_06730
lytic enzyme
Salmonella enterica subsp. enterica serovar Enteritidis str. EC20120774 genome−p.c.VAL54.69%−taxID1412612

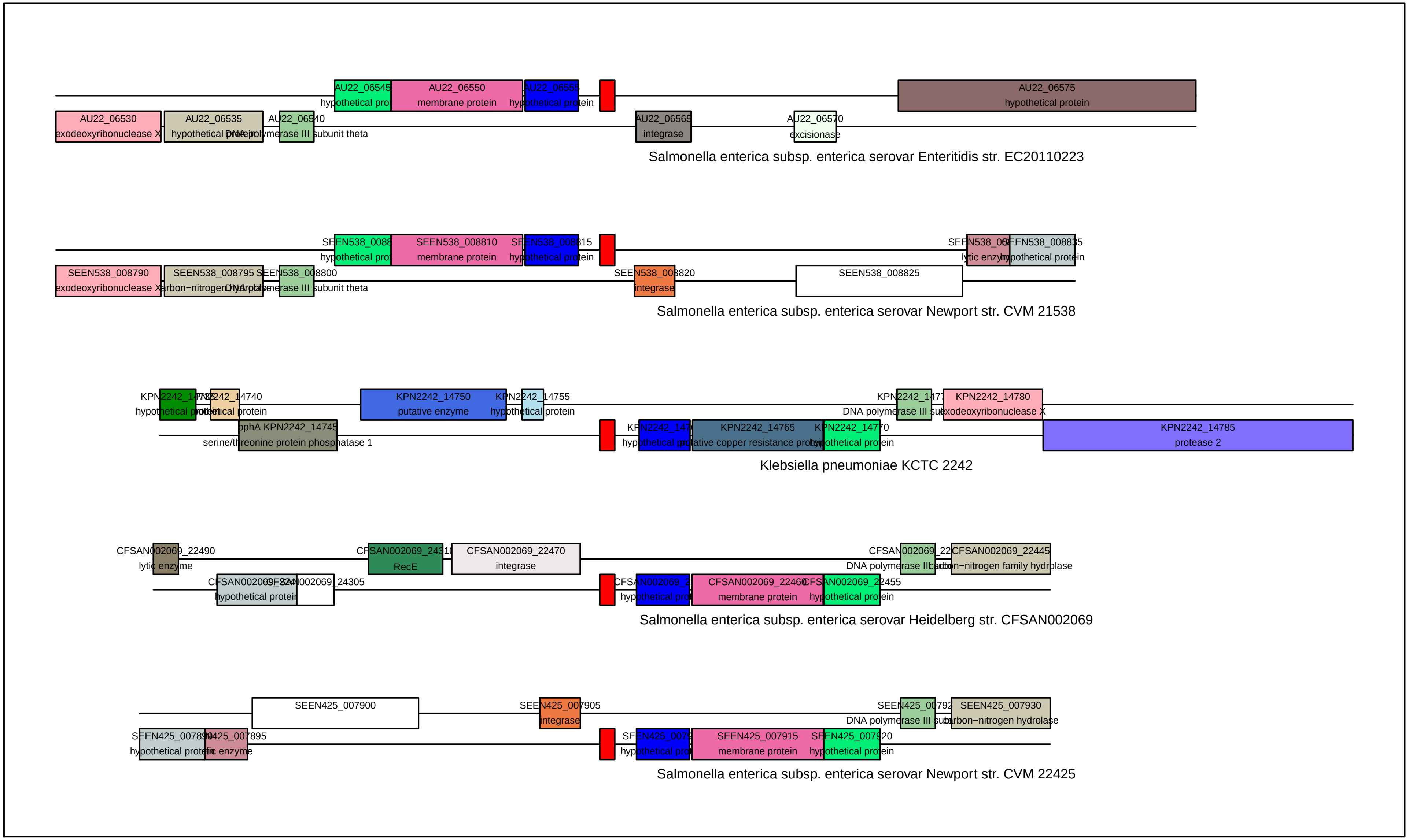

AU22_06550
membrane protein
AU22_06575
hypothetical protein
AU22_06530
exodeoxyribonuclease X
AU22_06535
AU22_06565
integrase
AU22_06570
excisionase
Salmonella enterica subsp. enterica serovar Enteritidis str. EC20110223
SEEN538_008810
membrane protein
SEEN538_008790
exodeoxyribonuclease X
SEEN538_008795
SEEN538_008825
Salmonella enterica subsp. enterica serovar Newport str. CVM 21538
KPN2242_14750
putative enzyme
KPN2242_14780
exodeoxyribonuclease X
KPN2242_14765
KPN2242_14785
protease 2
Klebsiella pneumoniae KCTC 2242
CFSAN002069_22470
integrase
RecE
CFSAN002069_22445
carbon−nitrogen family hydrolase
CFSAN002069_24305
membrane protein
Salmonella enterica subsp. enterica serovar Heidelberg str. CFSAN002069
SEEN425_007900
SEEN425_007930
carbon−nitrogen hydrolase
SEEN425_007915
membrane protein
Salmonella enterica subsp. enterica serovar Newport str. CVM 22425

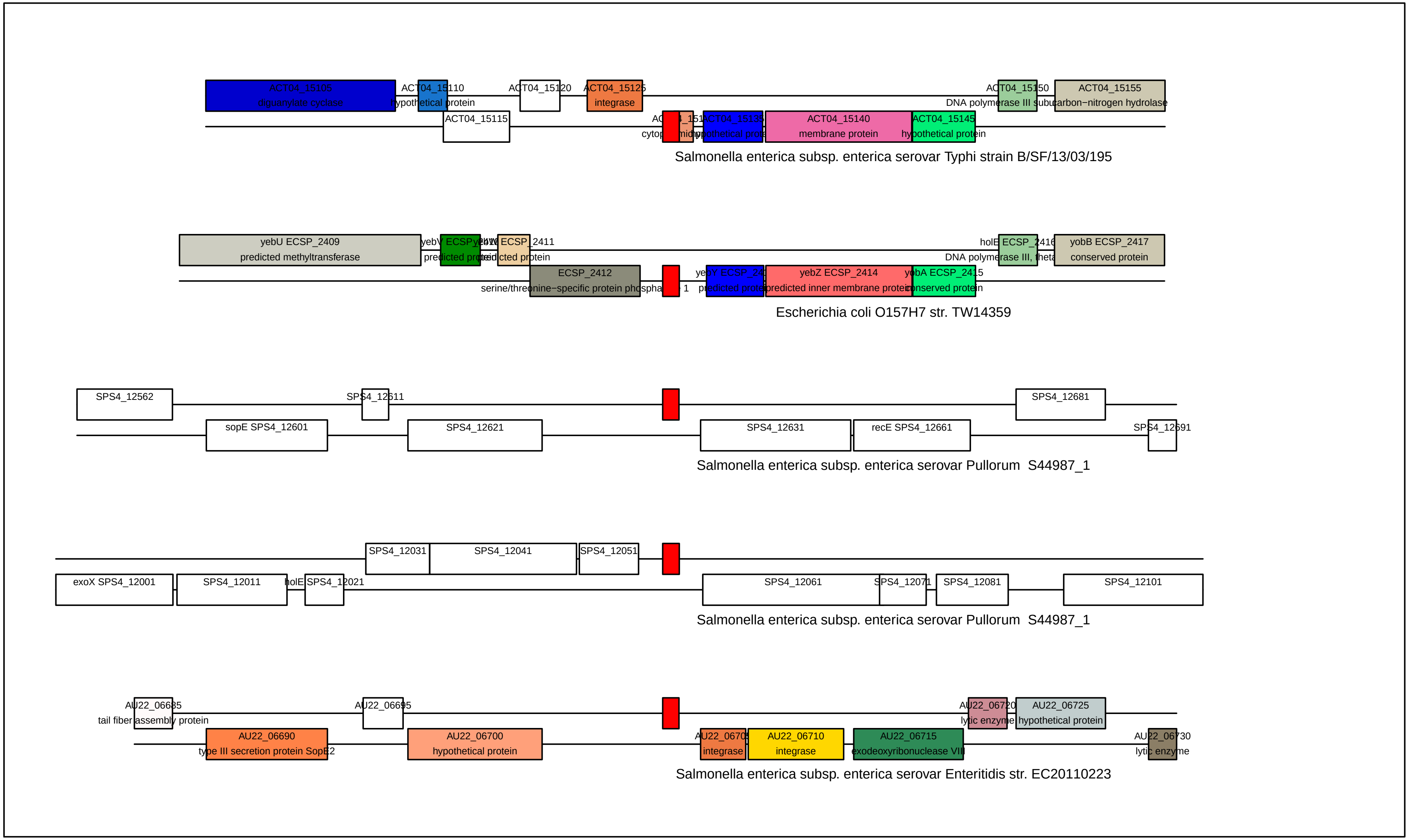

ACT04_15105
diguanylate cyclase
ACT04_15110
hypothetical protein
ACT04_15115
ACT04_15120
ACT04_15125
integrase
ACT04_15135
ACT04_15140
membrane protein
ACT04_15145
hypothetical protein
ACT04_15150
DNA polymerase III subunit
ACT04_15155
carbon−nitrogen hydrolase
Salmonella enterica subsp. enterica serovar Typhi strain B/SF/13/03/195
yebU ECSP_2409
predicted methyltransferase
ECSP_2411
predicted protein
ECSP_2412
serine/threonine−specific protein phosphatase 1
yebZ ECSP_2414
predicted inner membrane protein
yobA ECSP_2415
conserved protein
holE ECSP_2416
DNA polymerase III, theta
yobB ECSP_2417
conserved protein
Escherichia coli O157H7 str. TW14359
SPS4_12562
SPS4_12611
sopE SPS4_12601
SPS4_12621
SPS4_12631
recE SPS4_12661
SPS4_12681
SPS4_12691
Salmonella enterica subsp. enterica serovar Pullorum S44987_1
SPS4_12031
SPS4_12041
SPS4_12051
exoX SPS4_12001
SPS4_12011
holE SPS4_12021
SPS4_12061
SPS4_12071
SPS4_12081
SPS4_12101
Salmonella enterica subsp. enterica serovar Pullorum S44987_1
AU22_06685
tail fiber assembly protein
AU22_06695
AU22_06690
type III secretion protein SopE2
AU22_06700
hypothetical protein
integrase
AU22_06710
integrase
AU22_06715
exodeoxyribonuclease VIII
AU22_06720
lytic enzyme
AU22_06725
hypothetical protein
AU22_06730
lytic enzyme
Salmonella enterica subsp. enterica serovar Enteritidis str. EC20110223

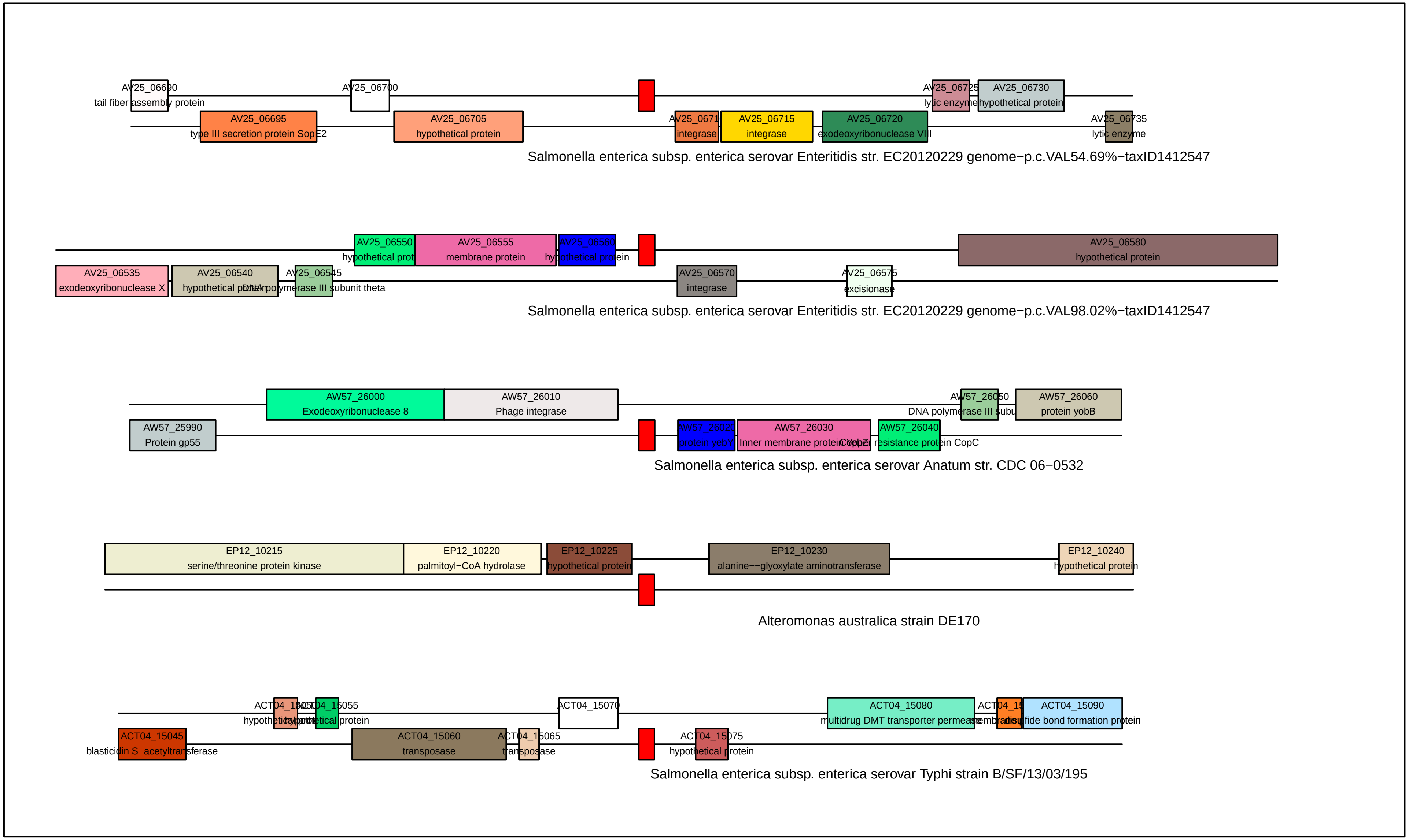

AV25_06690
tail fiber assembly protein
AV25_06700
AV25_06725
lytic enzyme
AV25_06730
hypothetical protein
AV25_06695
type III secretion protein SopE2
AV25_06705
hypothetical protein
integrase
AV25_06715
integrase
AV25_06720
exodeoxyribonuclease VIII
AV25_06735
lytic enzyme
Salmonella enterica subsp. enterica serovar Enteritidis str. EC20120229 genome−p.c.VAL54.69%−taxID1412547
AV25_06550
AV25_06555
membrane protein
AV25_06560
hypothetical protein
AV25_06580
hypothetical protein
AV25_06535
exodeoxyribonuclease X
AV25_06540
AV25_06545
DNA polymerase III subunit theta
AV25_06570
integrase
AV25_06575
excisionase
Salmonella enterica subsp. enterica serovar Enteritidis str. EC20120229 genome−p.c.VAL98.02%−taxID1412547
AW57_26000
Exodeoxyribonuclease 8
AW57_26010
Phage integrase
AW57_26050
AW57_26060
protein yobB
AW57_25990
Protein gp55
AW57_26020
protein yebY
AW57_26030
Inner membrane protein YebZ
AW57_26040
resistance protein CopC
Salmonella enterica subsp. enterica serovar Anatum str. CDC 06−0532
EP12_10215
serine/threonine protein kinase
EP12_10220
palmitoyl−CoA hydrolase
EP12_10225
hypothetical protein
EP12_10230
alanine−−glyoxylate aminotransferase
EP12_10240
hypothetical protein
Alteromonas australica strain DE170
ACT04_15055
hypothetical protein
ACT04_15070
ACT04_15080
multidrug DMT transporter permease
ACT04_15090
disulfide bond formation protein
ACT04_15045
blasticidin S−acetyltransferase
ACT04_15060
transposase
ACT04_15065
transposase
ACT04_15075
hypothetical protein
Salmonella enterica subsp. enterica serovar Typhi strain B/SF/13/03/195

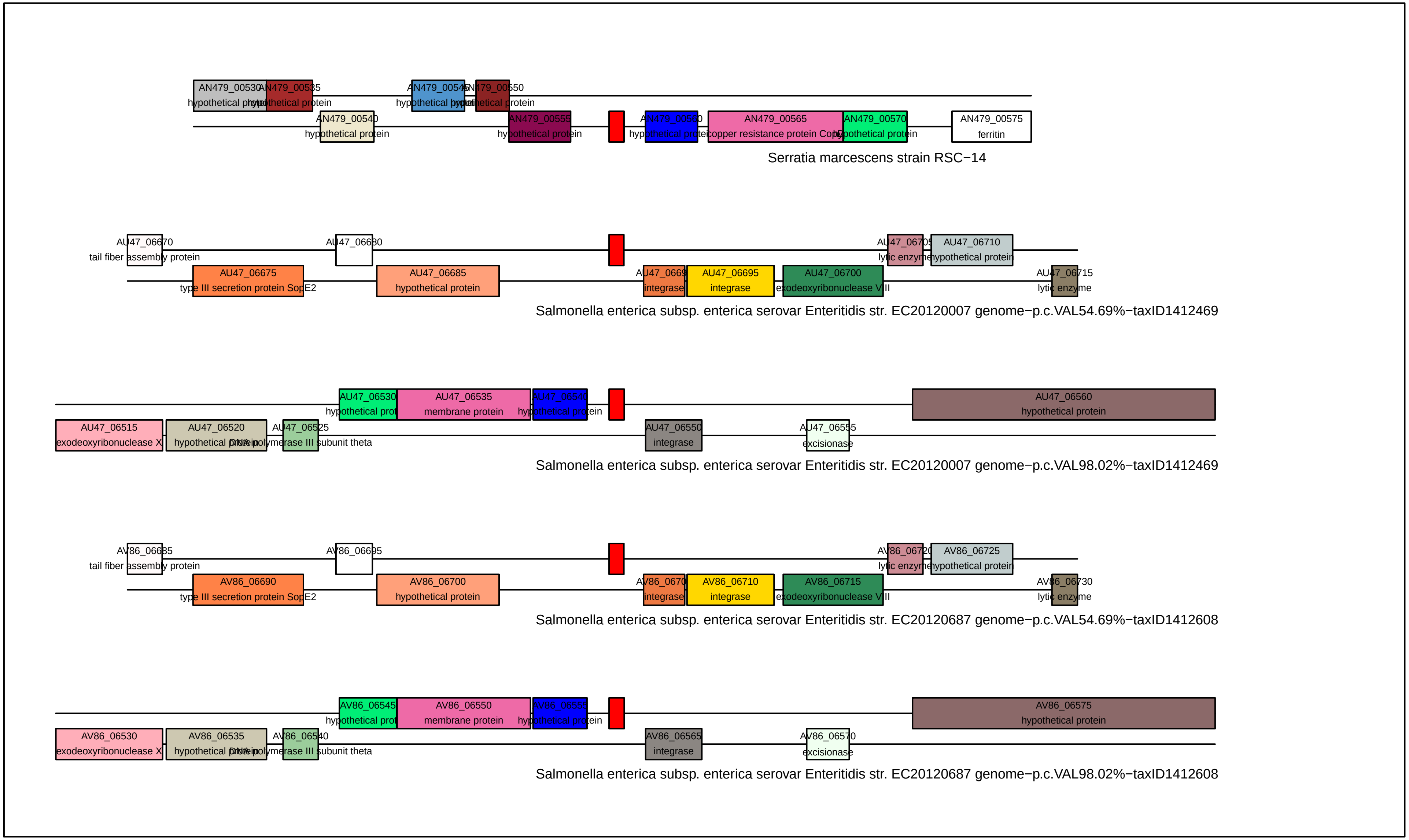

Serratia marcescens strain RSC−14
AN479_00530
AN479_00535
AN479_00540
AN479_00545
AN479_00550
AN479_00555
AN479_00560
AN479_00565
copper resistance protein CopD
AN479_00570
AN479_00575
ferritin
hypothetical protein
Salmonella enterica subsp. enterica serovar Enteritidis str. EC20120007 genome−p.c.VAL54.69%−taxID1412469
AU47_06670
tail fiber assembly protein
AU47_06675
type III secretion protein SopE2
AU47_06680
AU47_06685
integrase
AU47_06695
AU47_06700
exodeoxyribonuclease VIII
AU47_06710
AU47_06715
lytic enzyme
Salmonella enterica subsp. enterica serovar Enteritidis str. EC20120007 genome−p.c.VAL98.02%−taxID1412469
AU47_06515
exodeoxyribonuclease X
AU47_06520
AU47_06525
DNA polymerase III subunit theta
AU47_06530
AU47_06535
membrane protein
AU47_06540
AU47_06550
AU47_06555
excisionase
AU47_06560
Salmonella enterica subsp. enterica serovar Enteritidis str. EC20120687 genome−p.c.VAL54.69%−taxID1412608
AV86_06685
AV86_06690
AV86_06695
AV86_06700
AV86_06710
AV86_06715
AV86_06725
AV86_06730
Salmonella enterica subsp. enterica serovar Enteritidis str. EC20120687 genome−p.c.VAL98.02%−taxID1412608
AV86_06530
AV86_06535
AV86_06540
AV86_06545
AV86_06550
AV86_06555
AV86_06565
AV86_06570
AV86_06575

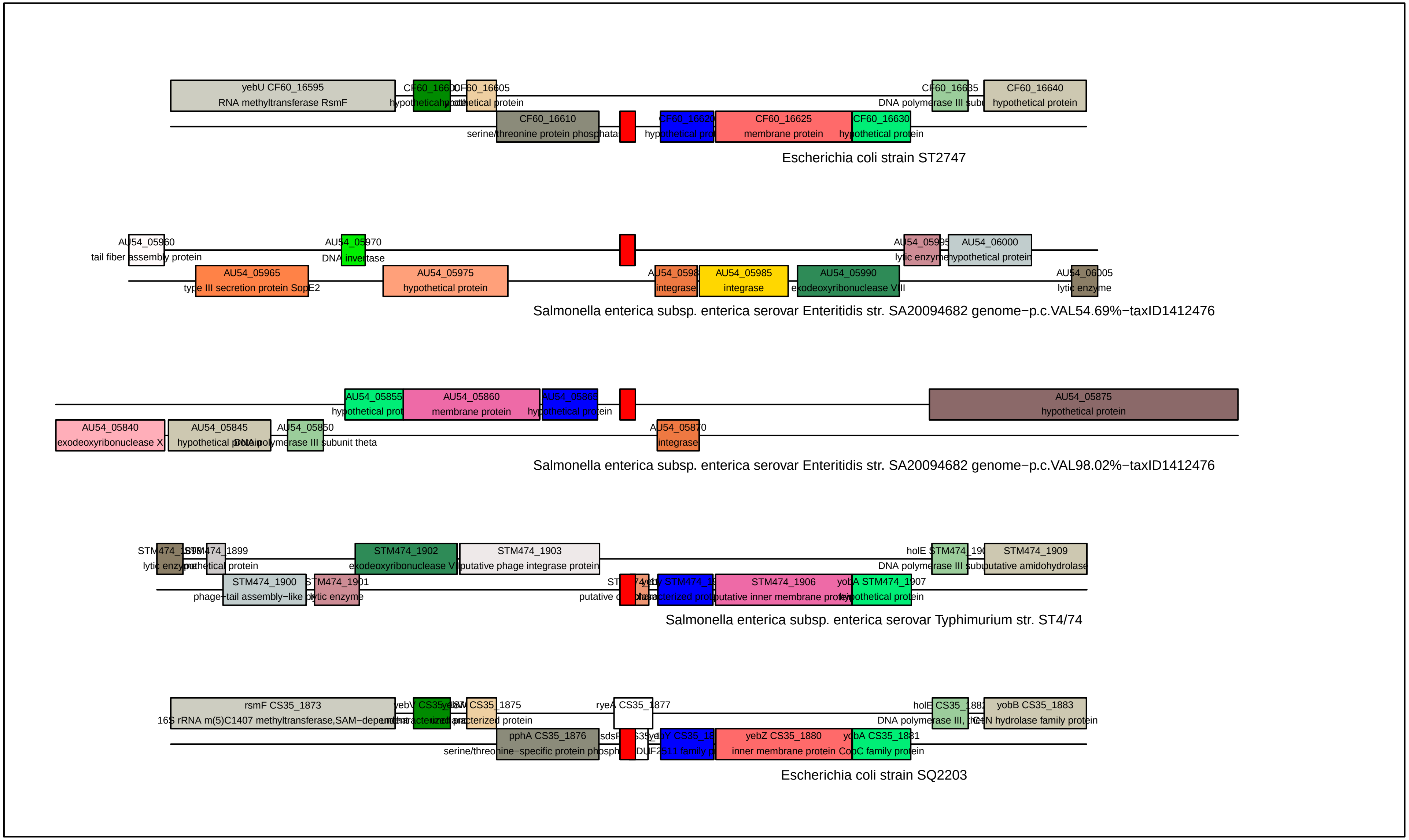

yebU CF60_16595
RNA methyltransferase RsmF
CF60_16605
CF60_16635
CF60_16640
hypothetical protein
DNA polymerase III
CF60_16610
serine/threonine protein phosphatase
CF60_16625
membrane protein
CF60_16630
hypothetical protein
Escherichia coli strain ST2747
AU54_05960
tail fiber assembly protein
AU54_05970
DNA invertase
AU54_06000
hypothetical protein
AU54_05965
type III secretion protein SopE2
AU54_05975
hypothetical protein
AU54_05985
integrase
AU54_05990
exodeoxyribonuclease VIII
AU54_06005
lytic enzyme
Salmonella enterica subsp. enterica serovar Enteritidis str. SA20094682 genome−p.c.VAL54.69%−taxID1412476
AU54_05860
membrane protein
AU54_05875
hypothetical protein
AU54_05840
exodeoxyribonuclease X
AU54_05845
AU54_05850
DNA polymerase III subunit theta
AU54_05870
integrase
Salmonella enterica subsp. enterica serovar Enteritidis str. SA20094682 genome−p.c.VAL98.02%−taxID1412476
STM474_1899
STM474_1902
exodeoxyribonuclease VIII
STM474_1903
putative phage integrase protein
STM474_1909
putative amidohydrolase
STM474_1900
phage−tail assembly−like protein
STM474_1901
lytic enzyme
STM474_1906
putative inner membrane protein
STM474_1907
hypothetical protein
Salmonella enterica subsp. enterica serovar Typhimurium str. ST4/74
rsmF CS35_1873
16S rRNA m(5)C1407 methyltransferase,SAM−dependent
CS35_1875
ryeA CS35_1877
yobB CS35_1883
hydrolase family protein
pphA CS35_1876
serine/threonine−specific protein phosphatase
yebZ CS35_1880
inner membrane protein CopC family protein
yobA CS35_1881
Escherichia coli strain SQ2203

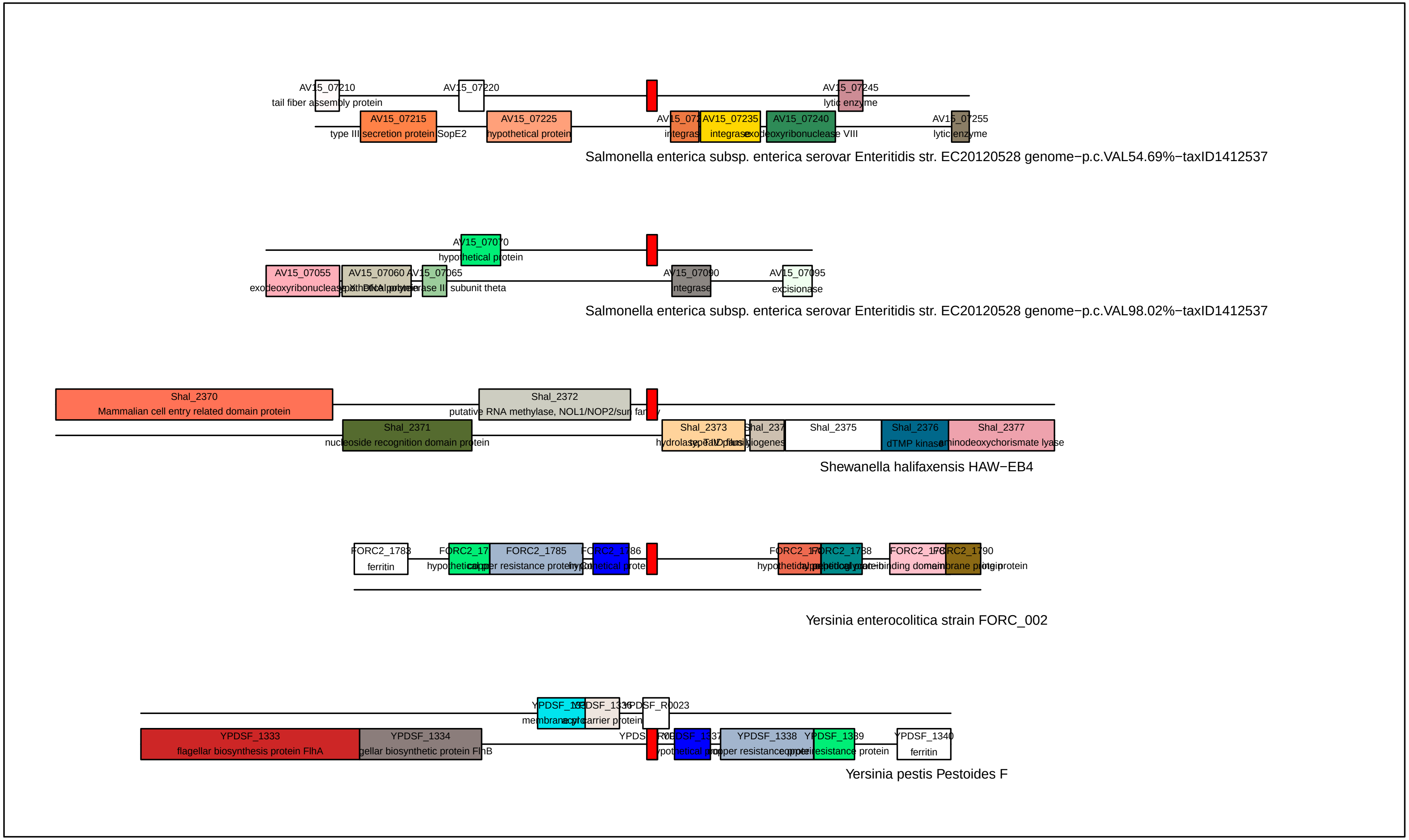

AV15_07210
tail fiber assembly protein
AV15_07220
AV15_07245
lytic enzyme
AV15_07215
type III secretion protein SopE2
AV15_07225
hypothetical protein
AV15_07235
integrase
AV15_07240
exodeoxyribonuclease VIII
AV15_07255
lytic enzyme
Salmonella enterica subsp. enterica serovar Enteritidis str. EC20120528 genome−p.c.VAL54.69%−taxID1412537
AV15_07070
hypothetical protein
AV15_07055
AV15_07060
AV15_07065
subunit theta
AV15_07090
integrase
AV15_07095
excisionase
Salmonella enterica subsp. enterica serovar Enteritidis str. EC20120528 genome−p.c.VAL98.02%−taxID1412537
Shal_2370
Mammalian cell entry related domain protein
Shal_2372
Shal_2371
nucleoside recognition domain protein
Shal_2373
Shal_2375
Shal_2376
dTMP kinase
Shal_2377
Shewanella halifaxensis HAW−EB4
FORC2_1783
ferritin
FORC2_1785
FORC2_1790
Yersinia enterocolitica strain FORC_002
YPDSF_R0023
YPDSF_1333
flagellar biosynthesis protein FlhA
YPDSF_1334
YPDSF_1338
YPDSF_1339
YPDSF_1340
ferritin
Yersinia pestis Pestoides F

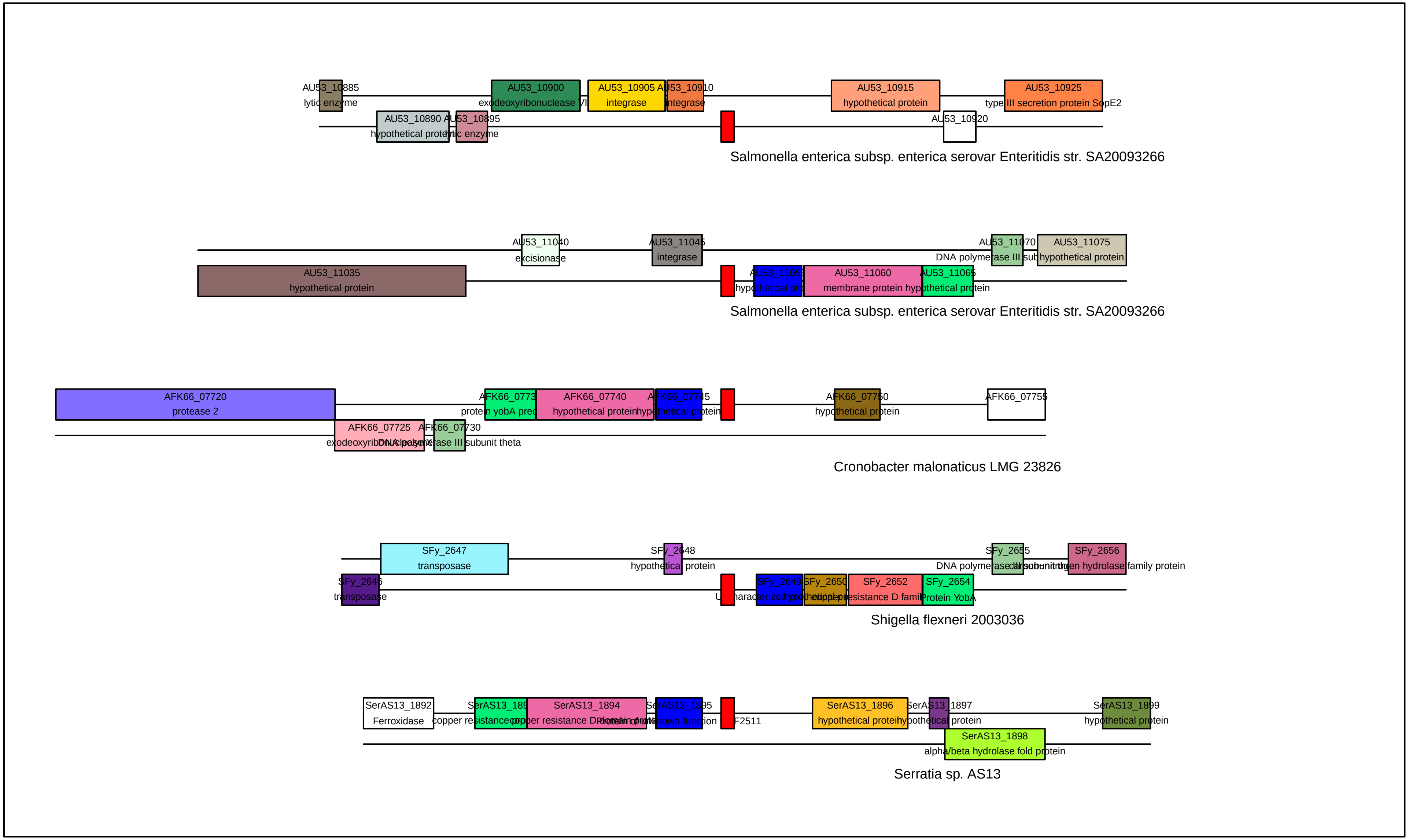
AU53_10885
lytic enzyme
AU53_10900
exodeoxyribonuclease
AU53_10905
integrase
AU53_10910
integrase
AU53_10915
hypothetical protein
AU53_10925
type III secretion protein SopE2
AU53_10890
hypothetical protein
AU53_10895
lytic enzyme
AU53_10920
Salmonella enterica subsp. enterica serovar Enteritidis str. SA20093266
AU53_11040
excisionase
AU53_11045
integrase
AU53_11070
AU53_11075
hypothetical protein
AU53_11035
hypothetical protein
AU53_11060
membrane protein
AU53_11065
hypothetical protein
Salmonella enterica subsp. enterica serovar Enteritidis str. SA20093266
AFK66_07720
protease 2
AFK66_07740
hypothetical protein
AFK66_07750
hypothetical protein
AFK66_07755
AFK66_07725
AFK66_07730
subunit theta
Cronobacter malonaticus LMG 23826
SFy_2647
transposase
SFy_2648
hypothetical protein
SFy_2655
SFy_2656
family protein
SFy_2646
transposase
SFy_2650
SFy_2652
SFy_2654
Protein YobA
Shigella flexneri 2003036
SerAS13_1892
Ferroxidase
SerAS13_1894
SerAS13_1895
SerAS13_1896
hypothetical protein
SerAS13_1897
SerAS13_1899
hypothetical protein
SerAS13_1898
alpha/beta hydrolase fold protein
Serratia sp. AS13

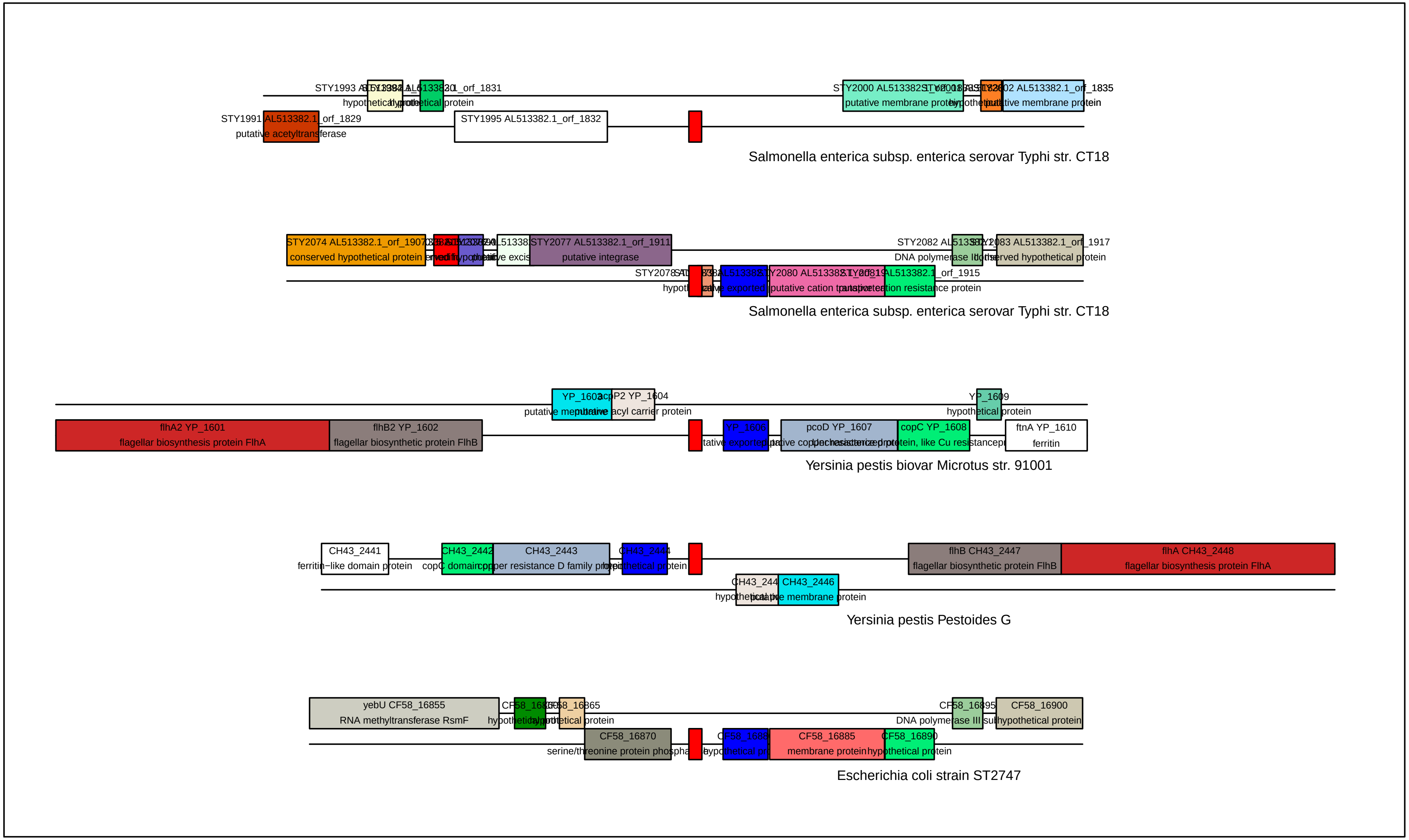
STY1991 AL513382.1_orf_1829
putative acetyltransferase
STY1995 AL513382.1_orf_1832
STY2002 AL513382.1_orf_1835
putative membrane protein
Salmonella enterica subsp. enterica serovar Typhi str. CT18
STY2074 AL513382.1_orf_1907
conserved hypothetical protein
STY2077 AL513382.1_orf_1911
putative integrase
STY2083 AL513382.1_orf_1917
DNA polymerase III
conserved hypothetical protein
AL513382.1_orf_1915
putative cation transporter
resistance protein
Salmonella enterica subsp. enterica serovar Typhi str. CT18
YP_1603
acpP2 YP_1604
putative acyl carrier protein
YP_1609
hypothetical protein
flhA2 YP_1601
flagellar biosynthesis protein FlhA
flhB2 YP_1602
flagellar biosynthetic protein FlhB
YP_1606
pcoD YP_1607
copC YP_1608
ftnA YP_1610
ferritin
Yersinia pestis biovar Microtus str. 91001
CH43_2441
ferritin−like domain protein
CH43_2442
CH43_2443
copper resistance D family protein
CH43_2444
hypothetical protein
flhB CH43_2447
flagellar biosynthetic protein FlhB
flhA CH43_2448
flagellar biosynthesis protein FlhA
CH43_2446
putative membrane protein
Yersinia pestis Pestoides G
yebU CF58_16855
RNA methyltransferase RsmF
CF58_16865
hypothetical protein
CF58_16895
DNA polymerase III
CF58_16900
hypothetical protein
CF58_16870
serine/threonine protein phospha
CF58_16885
membrane protein
CF58_16890
hypothetical protein
Escherichia coli strain ST2747

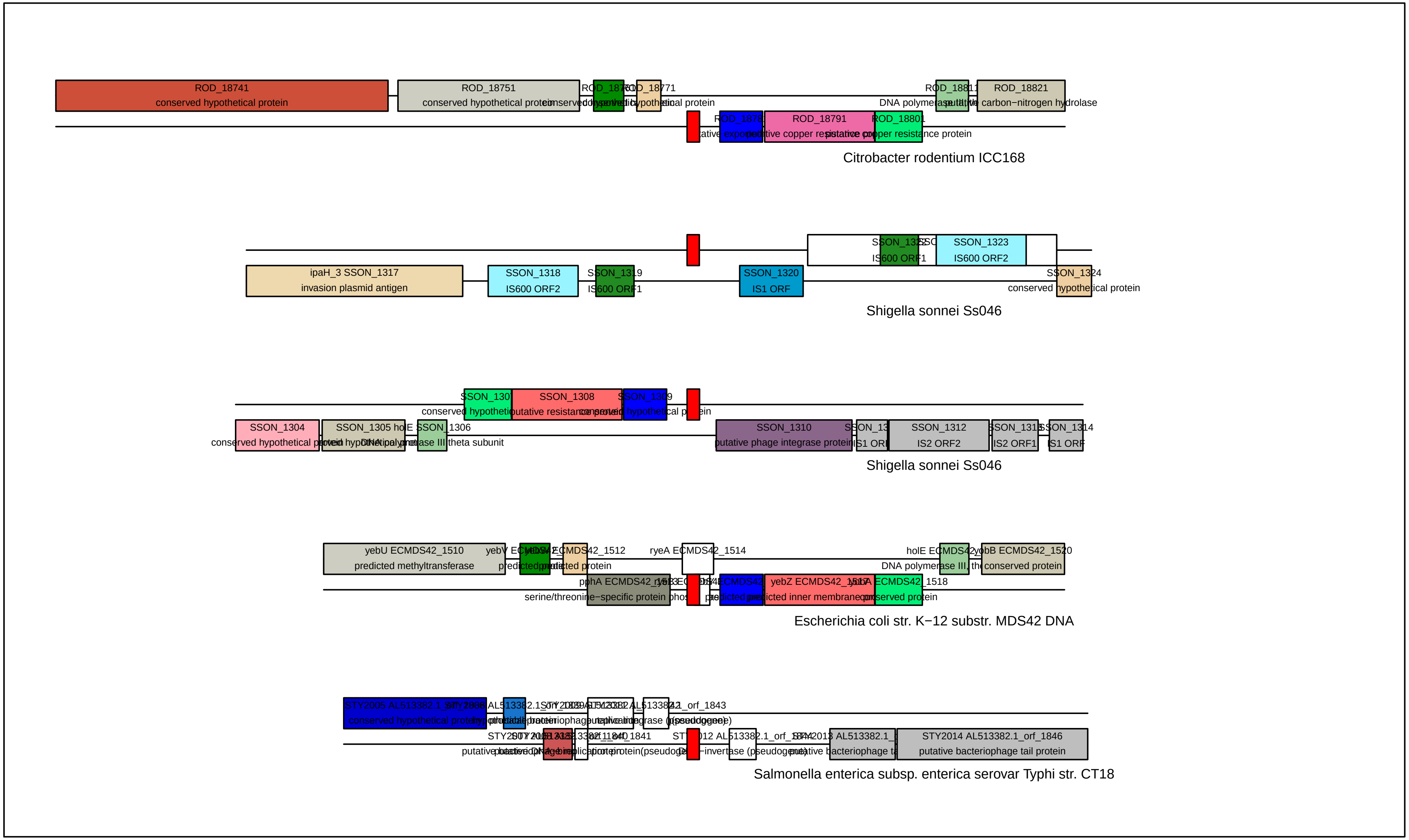
ROD_18741
conserved hypothetical protein
ROD_18751
ROD_18771
ROD_18791
ROD_18801
ROD_18821
carbon−nitrogen hydrolase
Citrobacter rodentium ICC168
SSON_1323
IS600 ORF2
ipaH_3 SSON_1317
invasion plasmid antigen
SSON_1318
IS600 ORF2
SSON_1320
IS1 ORF
SSON_1324
Shigella sonnei Ss046
SSON_1308
SSON_1304
SSON_1310
putative phage integrase protein
SSON_1312
IS2 ORF2
SSON_1314
Shigella sonnei Ss046
yebU ECMDS42_1510
predicted methyltransferase
ryeA ECMDS42_1514
DNA polymerase III
yobB ECMDS42_1520
pphA
serine/threonine−specific protein
Escherichia coli str. K−12 substr. MDS42 DNA
STY2014 AL513382.1_orf_1846
putative bacteriophage tail protein
Salmonella enterica subsp. enterica serovar Typhi str. CT18

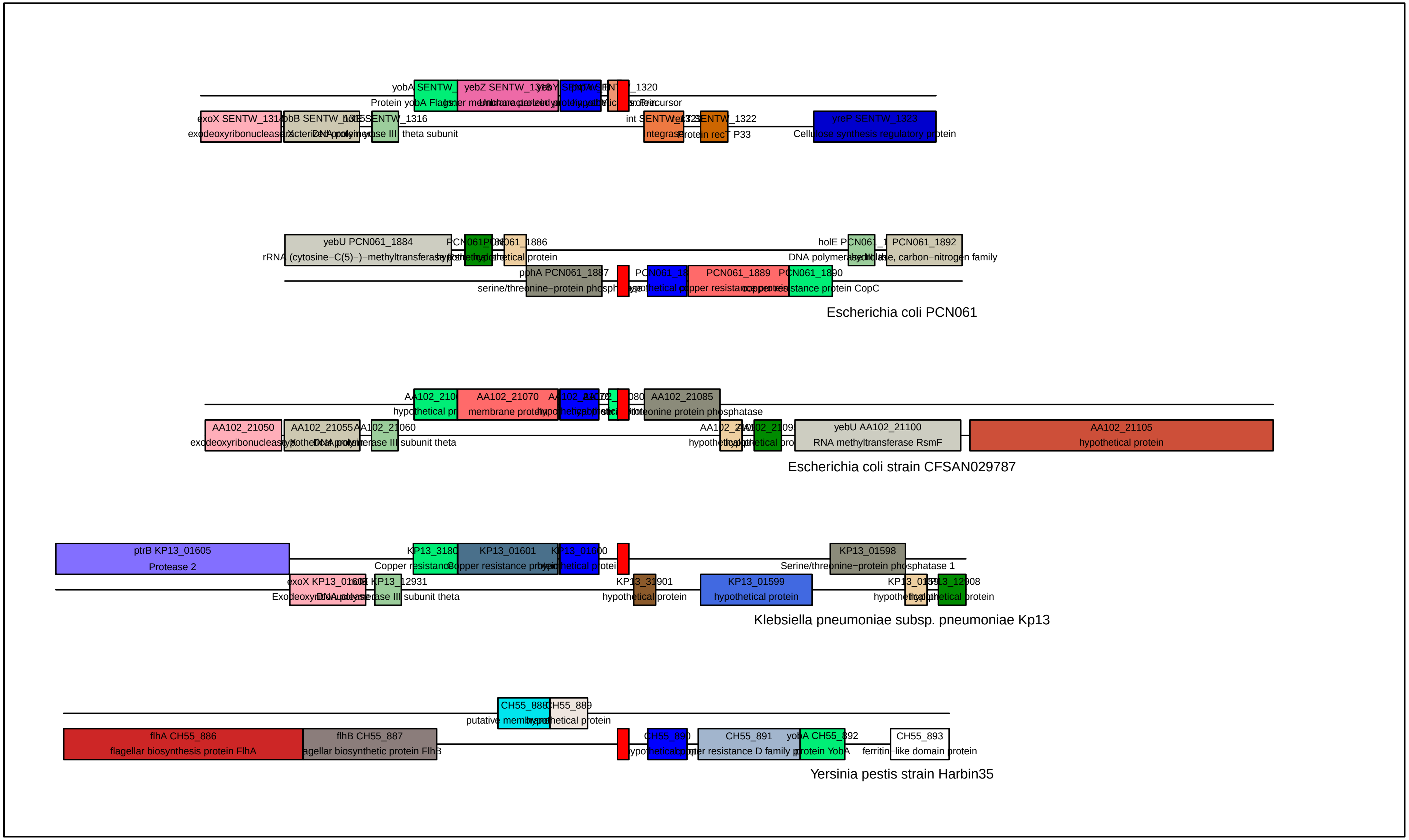

exoX SENTW_1314
exodeoxyribonuclease X
yebZ SENTW_1318
Inner membrane protein
int SENTW_1321
integrase
SENTW_1322
Protein recT
P33
yeeP SENTW_1323
Cellulose synthesis regulatory protein
theta subunit
yebU PCN061_1884
rRNA (cytosine−C(5)−)−methyltransferase
PCN061_1886
hypothetical protein
holE
PCN061_1892
DNA polymerase III
hydrolase, carbon−nitrogen family
pphA PCN061_1887
serine/threonine−protein phosphatase
PCN061_1889
PCN061_1890
copper resistance protein CopC
Escherichia coli PCN061
AA102_21070
membrane protein
AA102_21085
threonine protein phosphatase
AA102_21050
exodeoxyribonuclease X
AA102_21055
AA102_21060
subunit theta
yebU AA102_21100
RNA methyltransferase RsmF
AA102_21105
hypothetical protein
Escherichia coli strain CFSAN029787
ptrB KP13_01605
Protease 2
KP13_01601
Copper resistance protein
KP13_01598
Serine/threonine−protein phosphatase 1
exoX
Exodeoxyribonuclease
KP13_12931
subunit theta
KP13_31901
hypothetical protein
KP13_01599
hypothetical protein
Klebsiella pneumoniae subsp. pneumoniae Kp13
CH55_888
CH55_889
hypothetical protein
flhA CH55_886
flagellar biosynthesis protein FlhA
flhB CH55_887
CH55_890
CH55_891
copper resistance D family protein
yobA CH55_892
protein YobA
CH55_893
ferritin−like domain protein
Yersinia pestis strain Harbin35

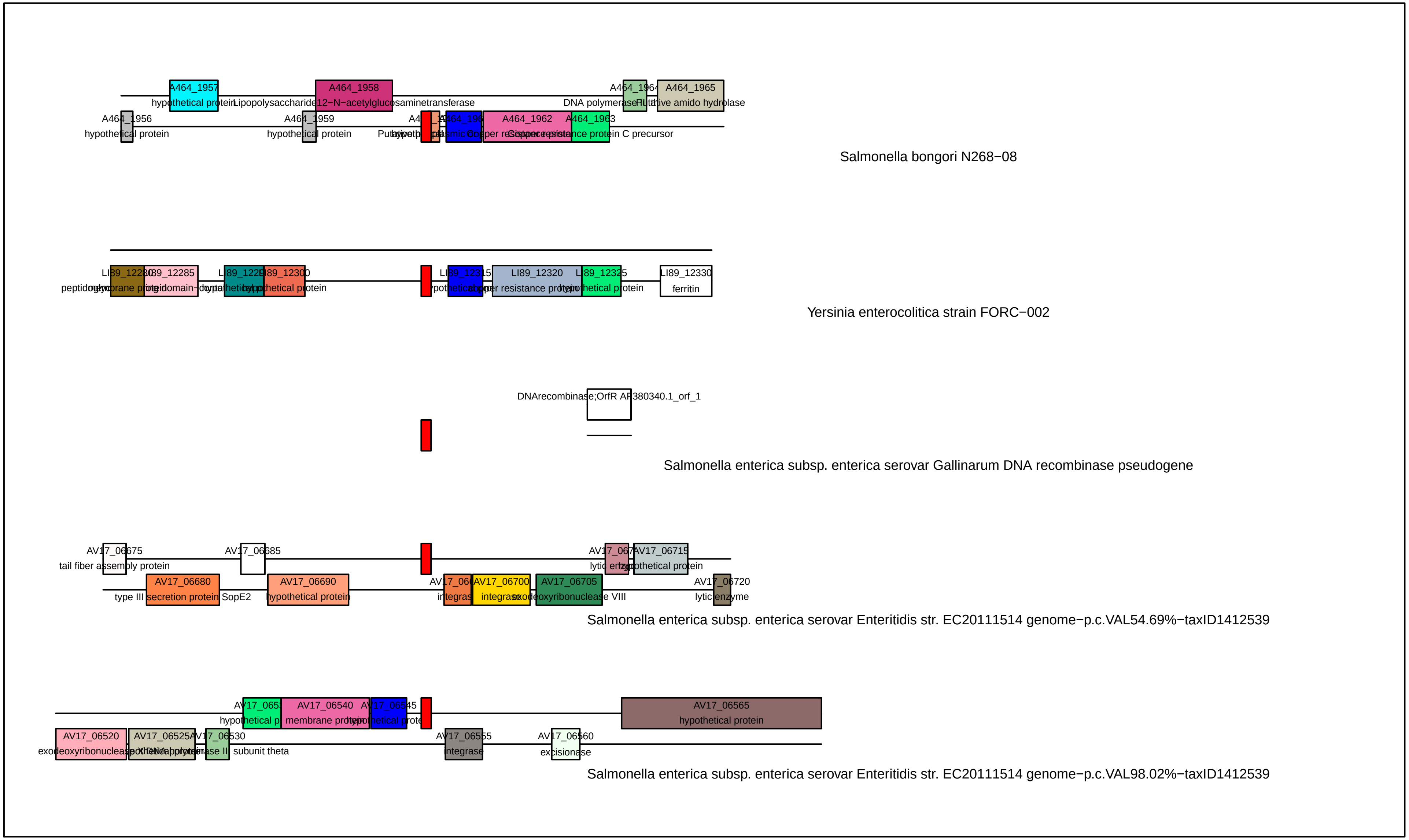

Salmonella bongori N268−08
Yersinia enterocolitica strain FORC−002
Salmonella enterica subsp. enterica serovar Gallinarum DNA recombinase pseudogene
Salmonella enterica subsp. enterica serovar Enteritidis str. EC20111514 genome−p.c.VAL54.69%−taxID1412539
Salmonella enterica subsp. enterica serovar Enteritidis str. EC20111514 genome−p.c.VAL98.02%−taxID1412539
A464_1957
A464_1958
A464_1965
Putative amido hydrolase
A464_1956
hypothetical protein
A464_1959
A464_1962
A464_1963
LI89_12285
LI89_12300
LI89_12320
LI89_12325
LI89_12330
ferritin
DNArecombinase;OrfR AF380340.1_orf_1
AV17_06675
tail fiber assembly protein
AV17_06685
AV17_06715
AV17_06680
AV17_06690
AV17_06700
AV17_06705
AV17_06720
lytic enzyme
AV17_06540
AV17_06545
AV17_06565
AV17_06520
AV17_06530
subunit theta
AV17_06555
integrase
AV17_06560
excisionase

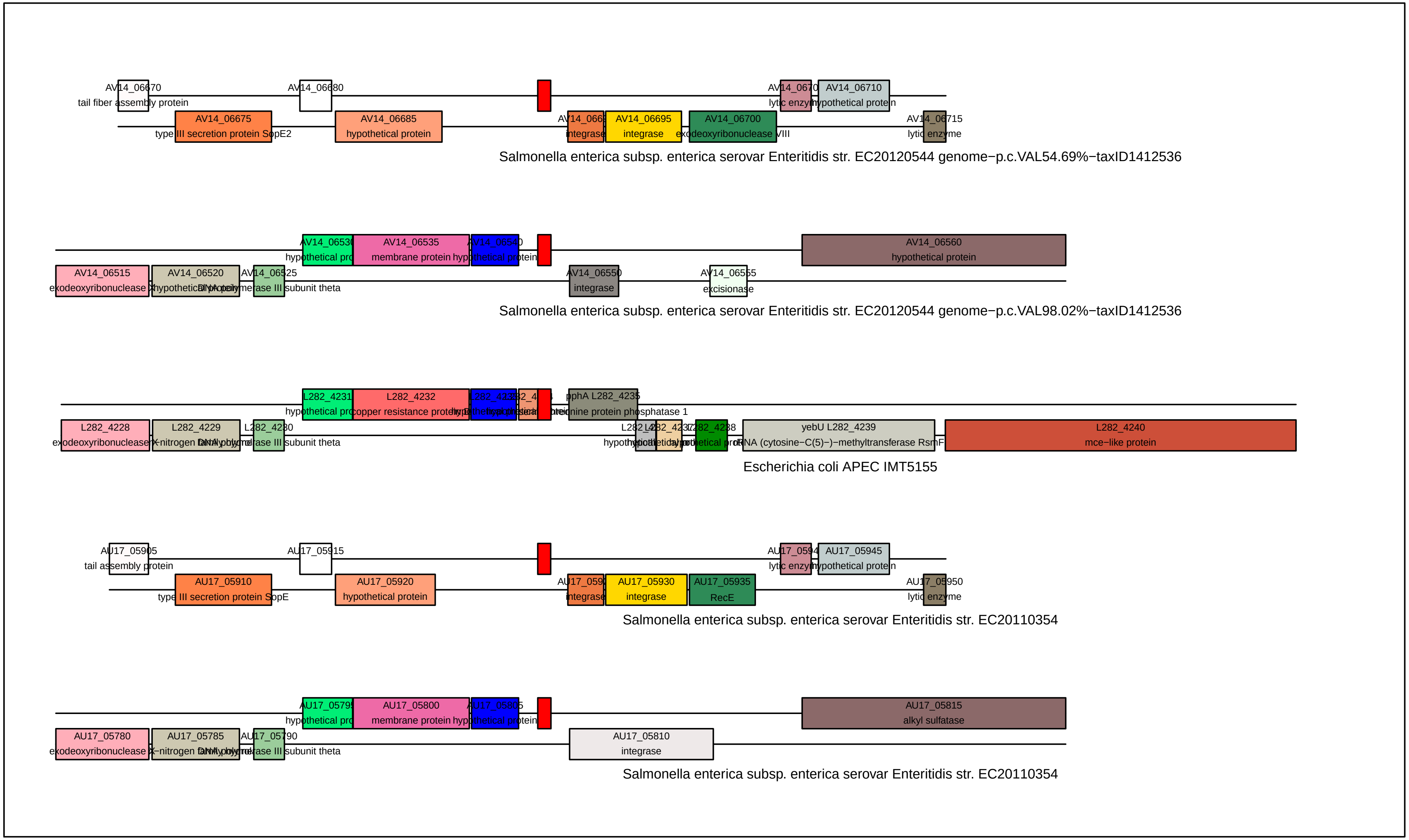
AV14_06670
tail fiber assembly protein
AV14_06680
AV14_06710
hypothetical protein
AV14_06675
type III secretion protein SopE2
AV14_06685
hypothetical protein
integrase
AV14_06695
integrase
AV14_06700
exodeoxyribonuclease VIII
AV14_06715
lytic enzyme
Salmonella enterica subsp. enterica serovar Enteritidis str. EC20120544 genome−p.c.VAL54.69%−taxID1412536
AV14_06535
membrane protein
AV14_06560
hypothetical protein
AV14_06515
exodeoxyribonuclease X
AV14_06520
AV14_06525
subunit theta
AV14_06550
integrase
AV14_06555
excisionase
Salmonella enterica subsp. enterica serovar Enteritidis str. EC20120544 genome−p.c.VAL98.02%−taxID1412536
L282_4231
L282_4232
copper resistance protein
pphA L282_4235
L282_4228
exodeoxyribonuclease X
L282_4229
L282_4230
subunit theta
yebU L282_4239
L282_4240
mce−like protein
Escherichia coli APEC IMT5155
AU17_05905
tail assembly protein
AU17_05915
AU17_05945
hypothetical protein
AU17_05910
type III secretion protein SopE
AU17_05920
hypothetical protein
integrase
AU17_05930
integrase
AU17_05935
RecE
AU17_05950
lytic enzyme
Salmonella enterica subsp. enterica serovar Enteritidis str. EC20110354
AU17_05800
membrane protein
AU17_05815
alkyl sulfatase
AU17_05780
exodeoxyribonuclease X
AU17_05785
subunit theta
AU17_05810
integrase
Salmonella enterica subsp. enterica serovar Enteritidis str. EC20110354

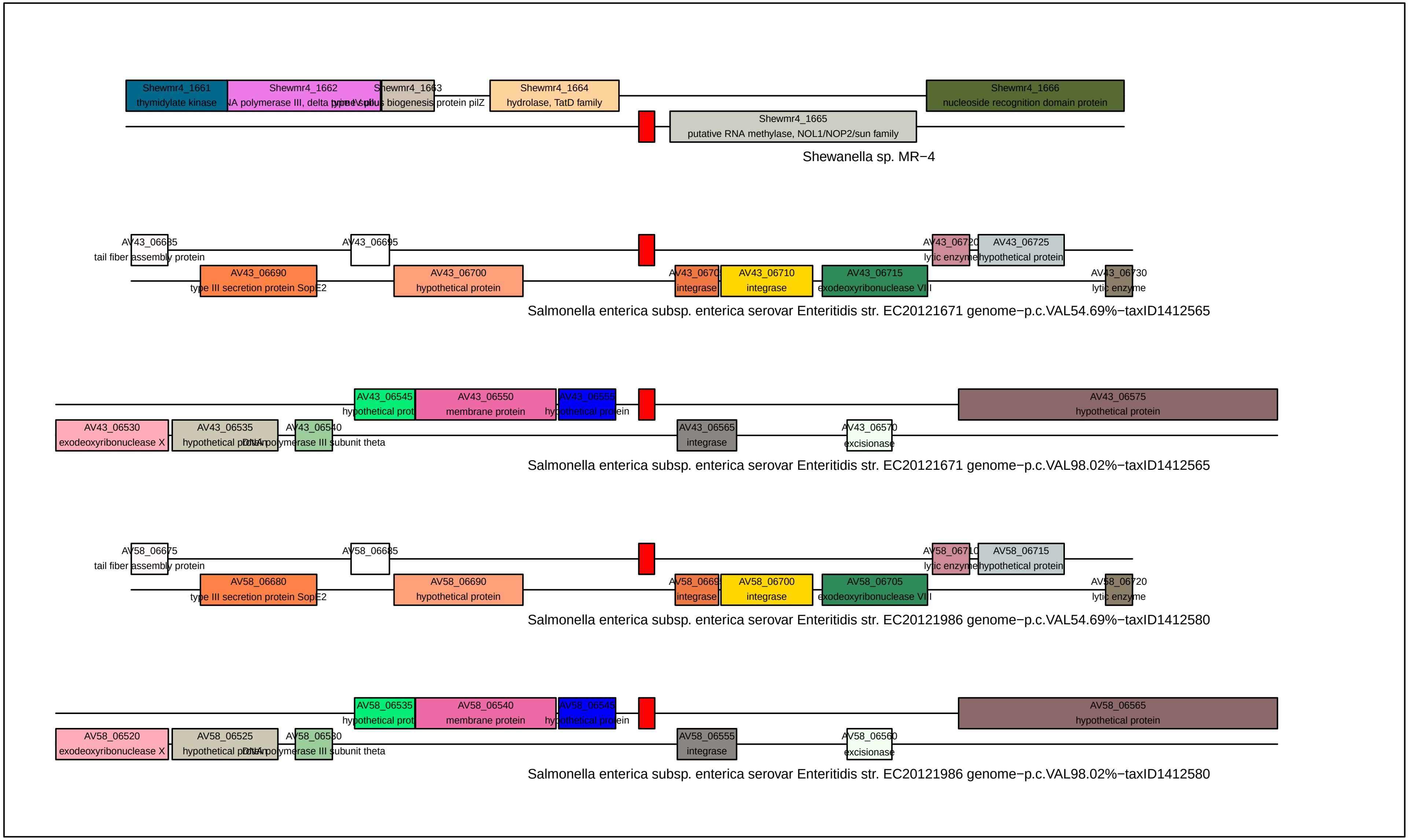

Shewmr4_1661
thymidylate kinase
Shewmr4_1662
Shewmr4_1663
Shewmr4_1664
hydrolase, TatD family
Shewmr4_1666
nucleoside recognition domain protein
Shewmr4_1665
putative RNA methylase, NOL1/NOP2/sun family
Shewanella sp. MR−4
AV43_06685
tail fiber assembly protein
AV43_06695
AV43_06725
hypothetical protein
AV43_06690
type III secretion protein SopE2
AV43_06700
hypothetical protein
integrase
AV43_06710
integrase
AV43_06715
exodeoxyribonuclease VIII
AV43_06730
lytic enzyme
Salmonella enterica subsp. enterica serovar Enteritidis str. EC20121671 genome−p.c.VAL54.69%−taxID1412565
AV43_06545
AV43_06550
membrane protein
AV43_06555
AV43_06575
hypothetical protein
AV43_06530
exodeoxyribonuclease X
AV43_06535
AV43_06540
AV43_06565
integrase
AV43_06570
excisionase
Salmonella enterica subsp. enterica serovar Enteritidis str. EC20121671 genome−p.c.VAL98.02%−taxID1412565
AV58_06675
tail fiber assembly protein
AV58_06685
AV58_06715
hypothetical protein
AV58_06680
type III secretion protein SopE2
AV58_06690
hypothetical protein
integrase
AV58_06700
integrase
AV58_06705
exodeoxyribonuclease VIII
AV58_06720
lytic enzyme
Salmonella enterica subsp. enterica serovar Enteritidis str. EC20121986 genome−p.c.VAL54.69%−taxID1412580
AV58_06535
AV58_06540
membrane protein
AV58_06545
AV58_06565
hypothetical protein
AV58_06520
exodeoxyribonuclease X
AV58_06525
AV58_06530
AV58_06555
integrase
AV58_06560
excisionase
Salmonella enterica subsp. enterica serovar Enteritidis str. EC20121986 genome−p.c.VAL98.02%−taxID1412580

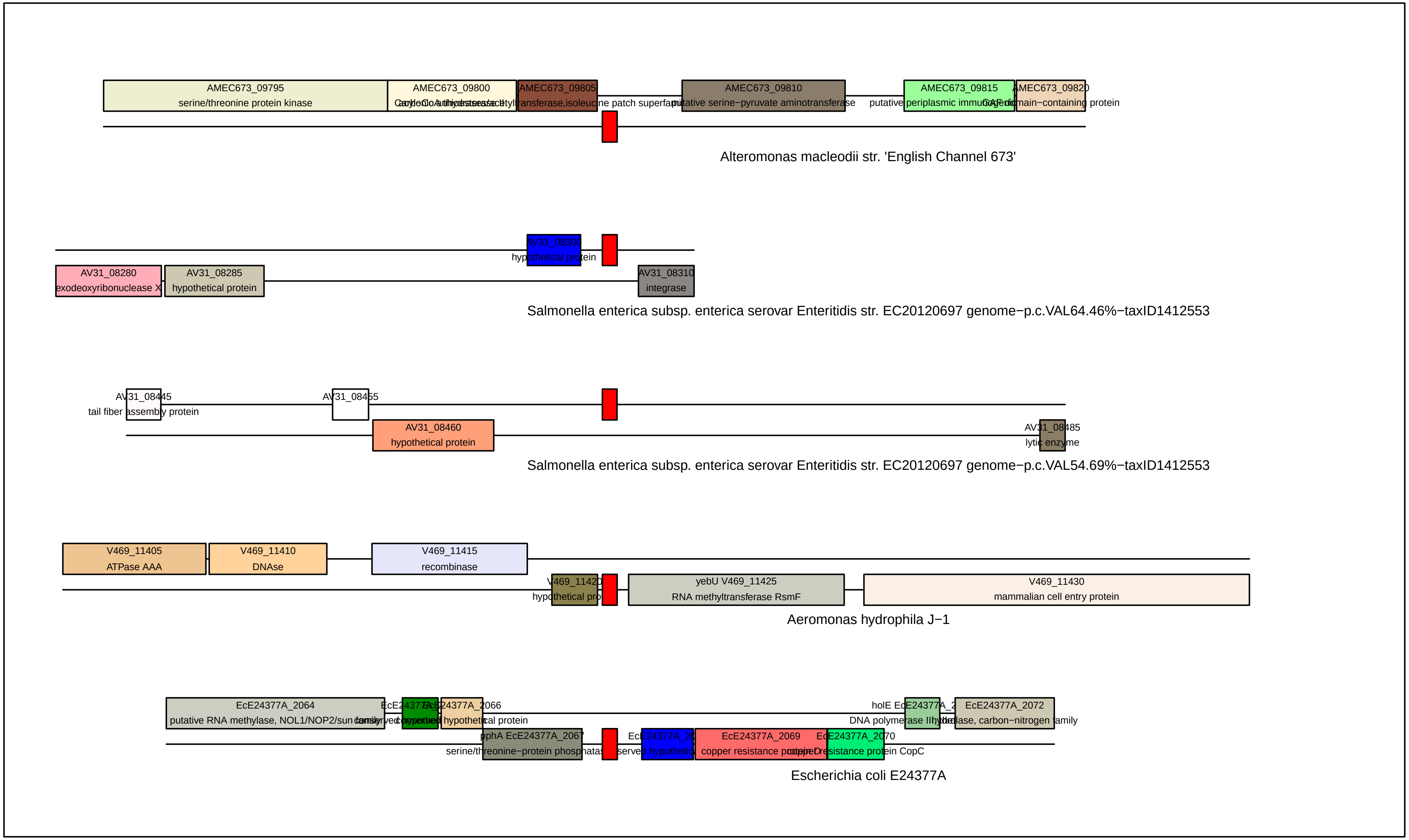

AMEC673_09795
serine/threonine protein kinase
AMEC673_09800
AMEC673_09805
AMEC673_09810
putative serine−pyruvate aminotransferase
AMEC673_09815
AMEC673_09820
Alteromonas macleodii str. 'English Channel 673'
AV31_08280
exodeoxyribonuclease X
AV31_08285
hypothetical protein
AV31_08310
integrase
Salmonella enterica subsp. enterica serovar Enteritidis str. EC20120697 genome−p.c.VAL64.46%−taxID1412553
AV31_08445
tail fiber assembly protein
AV31_08460
hypothetical protein
AV31_08485
lytic enzyme
Salmonella enterica subsp. enterica serovar Enteritidis str. EC20120697 genome−p.c.VAL54.69%−taxID1412553
V469_11405
ATPase AAA
V469_11410
DNAse
V469_11415
recombinase
yebU V469_11425
RNA methyltransferase RsmF
V469_11430
mammalian cell entry protein
Aeromonas hydrophila J−1
EcE24377A_2064
pphA EcE24377A_2067
EcE24377A_2069
EcE24377A_2070
EcE24377A_2072
Escherichia coli E24377A

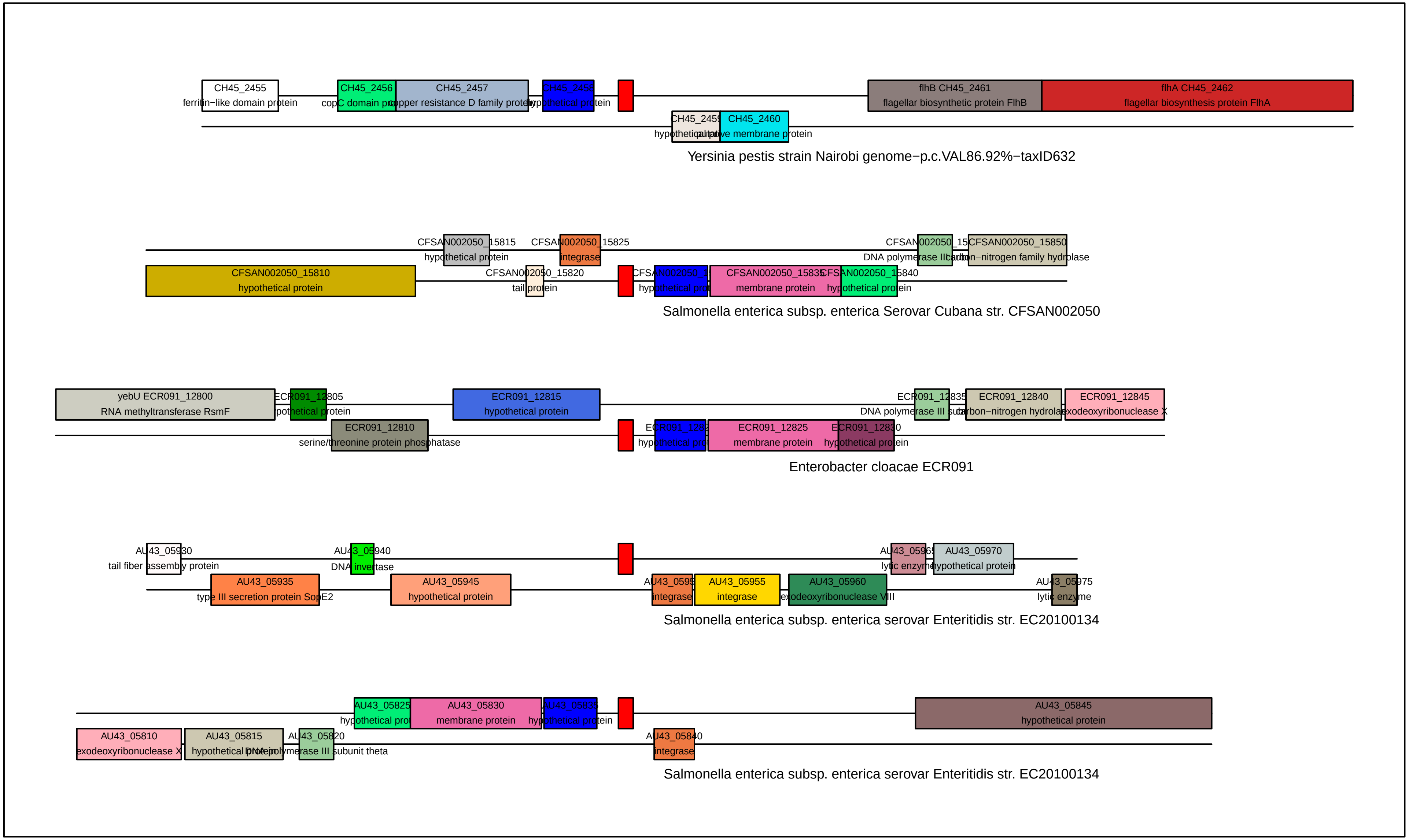

CH45_2455
ferritin−like domain protein
CH45_2456
CH45_2457
copper resistance D family protein
flhB CH45_2461
flagellar biosynthetic protein FlhB
flhA CH45_2462
flagellar biosynthesis protein FlhA
CH45_2460
putative membrane protein
Yersinia pestis strain Nairobi genome−p.c.VAL86.92%−taxID632
CFSAN002050_15815
hypothetical protein
CFSAN002050_15825
integrase
CFSAN002050_15850
carbon−nitrogen family hydrolase
CFSAN002050_15810
hypothetical protein
CFSAN002050_15820
tail protein
membrane protein
CFSAN002050_15840
hypothetical protein
Salmonella enterica subsp. enterica Serovar Cubana str. CFSAN002050
yebU ECR091_12800
RNA methyltransferase RsmF
ECR091_12805
ECR091_12815
hypothetical protein
ECR091_12840
ECR091_12845
ECR091_12810
serine/threonine protein phosphatase
ECR091_12825
membrane protein
ECR091_12830
hypothetical protein
Enterobacter cloacae ECR091
AU43_05930
tail fiber assembly protein
AU43_05940
DNA invertase
AU43_05970
hypothetical protein
AU43_05935
type III secretion protein SopE2
AU43_05945
hypothetical protein
AU43_05955
integrase
AU43_05960
exodeoxyribonuclease VIII
AU43_05975
lytic enzyme
Salmonella enterica subsp. enterica serovar Enteritidis str. EC20100134
AU43_05825
AU43_05830
membrane protein
AU43_05845
hypothetical protein
AU43_05810
exodeoxyribonuclease X
AU43_05815
AU43_05820
AU43_05840
integrase
Salmonella enterica subsp. enterica serovar Enteritidis str. EC20100134

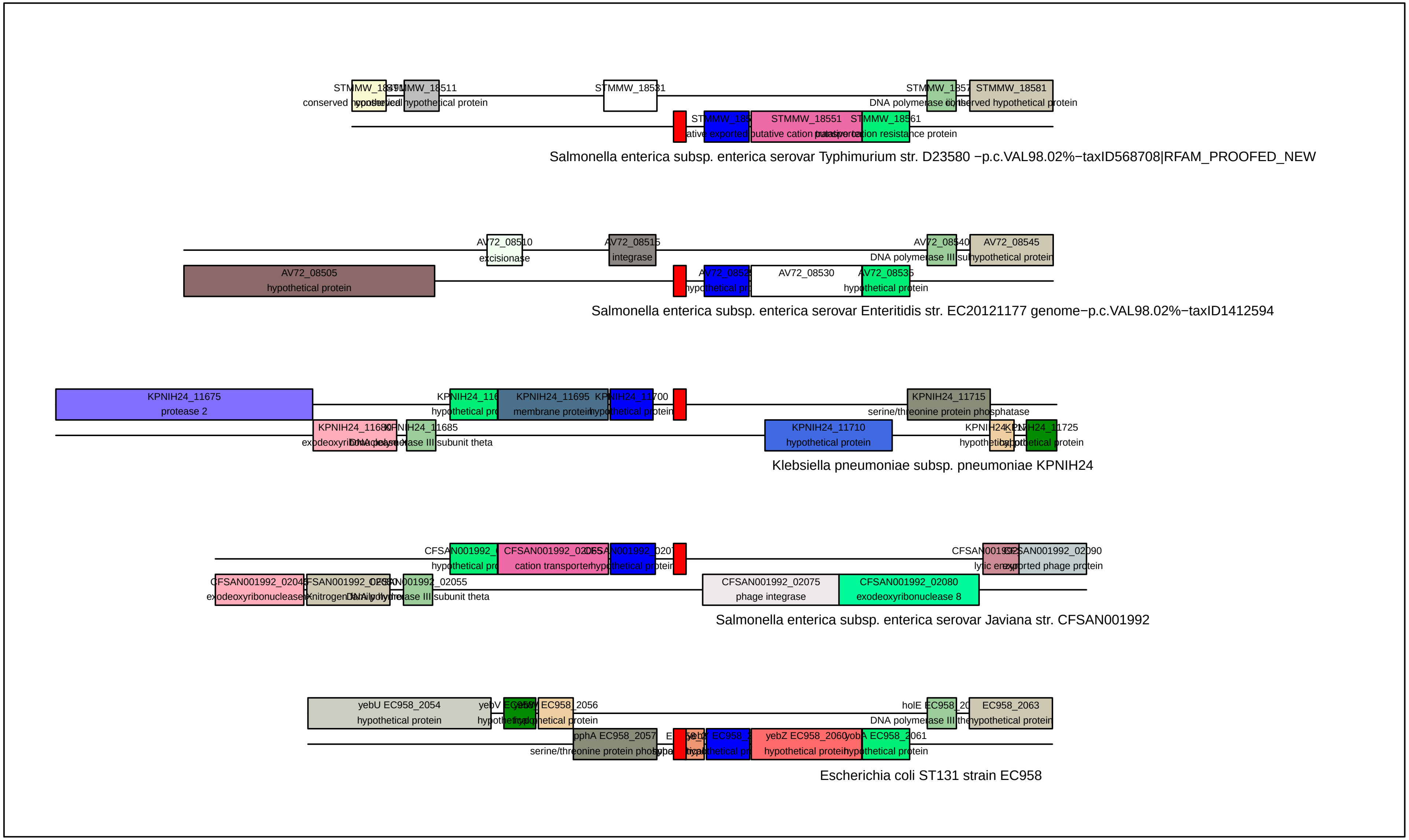
STMMW_18511
STMMW_18531
STMMW_18551
STMMW_18561
STMMW_18581
conserved hypothetical protein
DNA polymerase
Salmonella enterica subsp. enterica serovar Typhimurium str. D23580 −p.c.VAL98.02%−taxID568708|RFAM_PROOFED_NEW
AV72_08505
hypothetical protein
AV72_08510
excisionase
AV72_08515
integrase
AV72_08530
AV72_08535
AV72_08540
AV72_08545
hypothetical protein
Salmonella enterica subsp. enterica serovar Enteritidis str. EC20121177 genome−p.c.VAL98.02%−taxID1412594
KPNIH24_11675
protease 2
KPNIH24_11685
subunit theta
KPNIH24_11695
membrane protein
KPNIH24_11700
KPNIH24_11710
hypothetical protein
KPNIH24_11715
serine/threonine protein phosphatase
KPNIH24_11725
Klebsiella pneumoniae subsp. pneumoniae KPNIH24
CFSAN001992_02055
subunit theta
cation transporter
CFSAN001992_02075
phage integrase
CFSAN001992_02080
exodeoxyribonuclease 8
CFSAN001992_02090
exported phage protein
Salmonella enterica subsp. enterica serovar Javiana str. CFSAN001992
yebU EC958_2054
hypothetical protein
EC958_2056
pphA EC958_2057
yebZ EC958_2060
hypothetical protein
EC958_2061
EC958_2063
Escherichia coli ST131 strain EC958

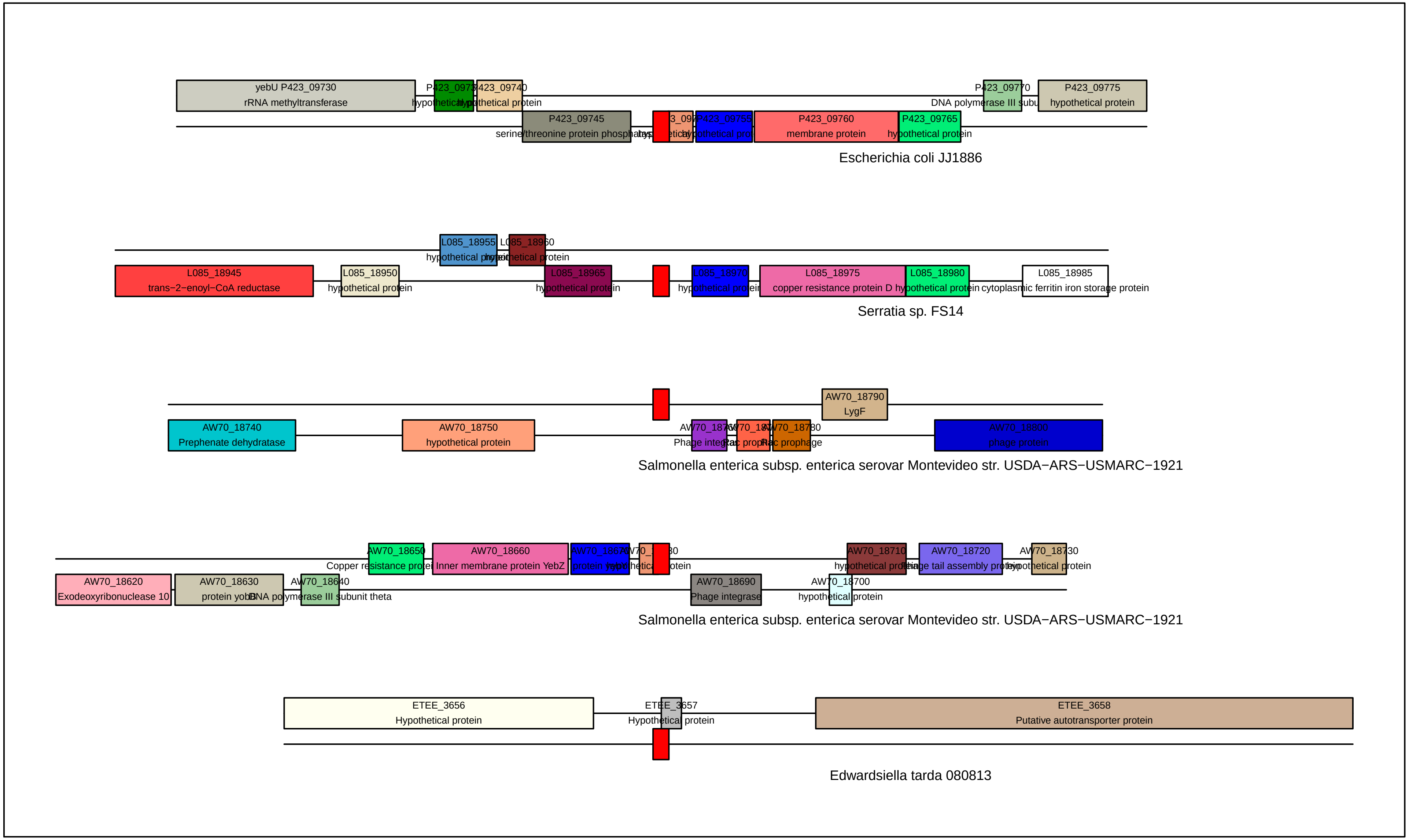

yebU P423_09730
rRNA methyltransferase
P423_09775
hypothetical protein
P423_09745
P423_09755
P423_09760
membrane protein
P423_09765
hypothetical protein
DNA polymerase III
Escherichia coli JJ1886
L085_18955
L085_18945
trans−2−enoyl−CoA reductase
L085_18950
hypothetical protein
L085_18965
hypothetical protein
L085_18970
L085_18975
copper resistance protein D
L085_18980
hypothetical protein
L085_18985
cytoplasmic ferritin iron storage protein
Serratia sp. FS14
AW70_18790
LygF
AW70_18740
Prephenate dehydratase
AW70_18750
hypothetical protein
AW70_18800
phage protein
Salmonella enterica subsp. enterica serovar Montevideo str. USDA−ARS−USMARC−1921
AW70_18650
AW70_18660
Inner membrane protein YebZ
AW70_18710
AW70_18720
AW70_18730
AW70_18620
Exodeoxyribonuclease 10
AW70_18630
AW70_18690
Phage integrase
AW70_18700
hypothetical protein
Salmonella enterica subsp. enterica serovar Montevideo str. USDA−ARS−USMARC−1921
ETEE_3656
Hypothetical protein
ETEE_3658
Putative autotransporter protein
Edwardsiella tarda 080813

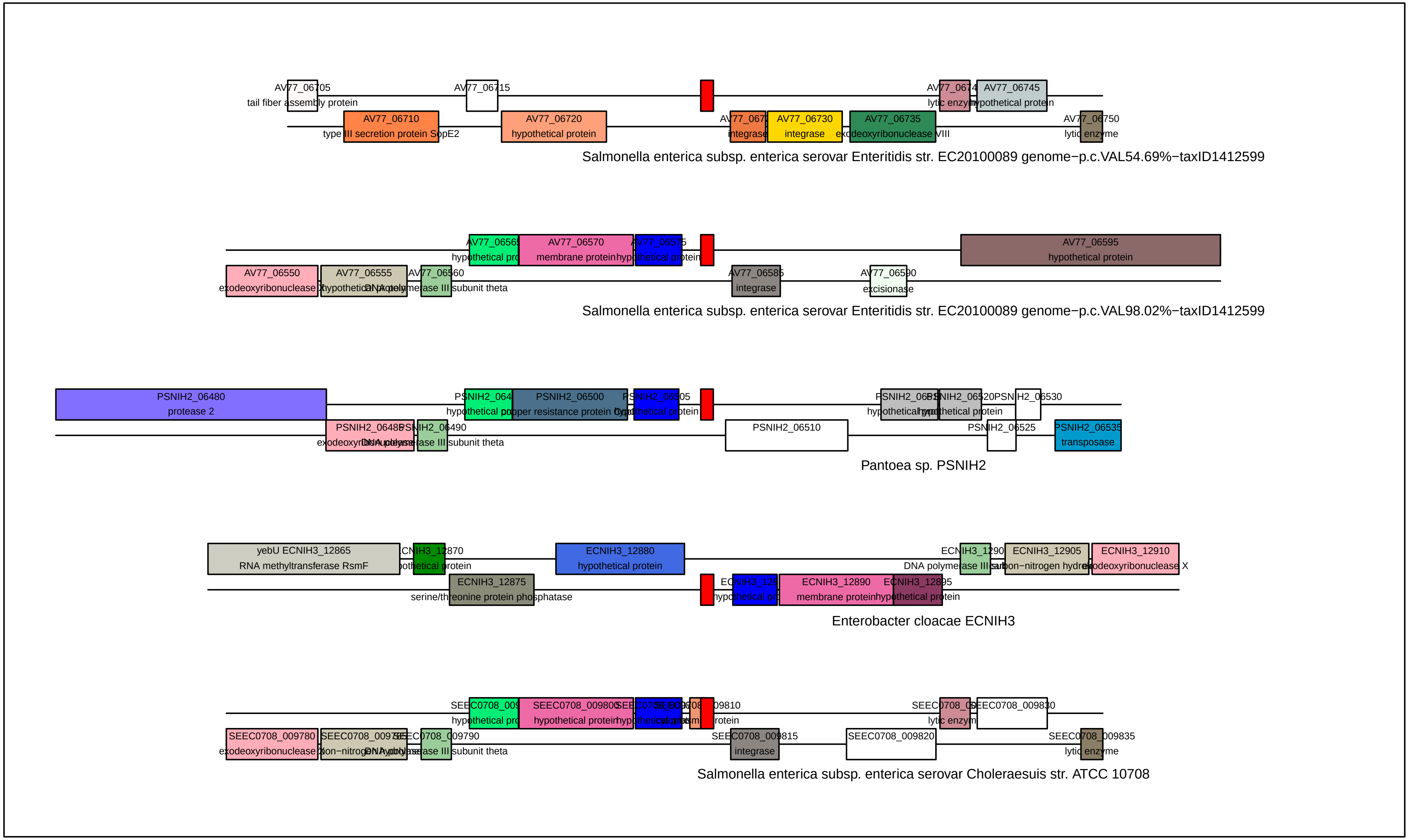
AV77_06705
tail fiber assembly protein
AV77_06715
AV77_06745
hypothetical protein
AV77_06710
type III secretion protein SopE2
AV77_06720
hypothetical protein
AV77_06730
integrase
AV77_06735
exodeoxyribonuclease VIII
AV77_06750
lytic enzyme
Salmonella enterica subsp. enterica serovar Enteritidis str. EC20100089 genome−p.c.VAL54.69%−taxID1412599
AV77_06570
membrane protein
AV77_06595
hypothetical protein
AV77_06550
exodeoxyribonuclease X
AV77_06555
AV77_06560
DNA polymerase III subunit theta
AV77_06585
integrase
AV77_06590
excisionase
Salmonella enterica subsp. enterica serovar Enteritidis str. EC20100089 genome−p.c.VAL98.02%−taxID1412599
PSNIH2_06480
protease 2
PSNIH2_06500
copper resistance protein
PSNIH2_06490
DNA polymerase III subunit theta
PSNIH2_06510
PSNIH2_06530
PSNIH2_06525
PSNIH2_06535
transposase
Pantoea sp. PSNIH2
yebU ECNIH3_12865
RNA methyltransferase RsmF
ECNIH3_12870
ECNIH3_12880
hypothetical protein
ECNIH3_12905
ECNIH3_12910
exodeoxyribonuclease X
ECNIH3_12875
serine/threonine protein phosphatase
ECNIH3_12890
membrane protein
ECNIH3_12895
hypothetical protein
Enterobacter cloacae ECNIH3
SEEC0708_009800
hypothetical protein
SEEC0708_009830
SEEC0708_009780
exodeoxyribonuclease X
SEEC0708_009790
DNA polymerase III subunit theta
SEEC0708_009815
integrase
SEEC0708_009820
SEEC0708_009835
lytic enzyme
Salmonella enterica subsp. enterica serovar Choleraesuis str. ATCC 10708

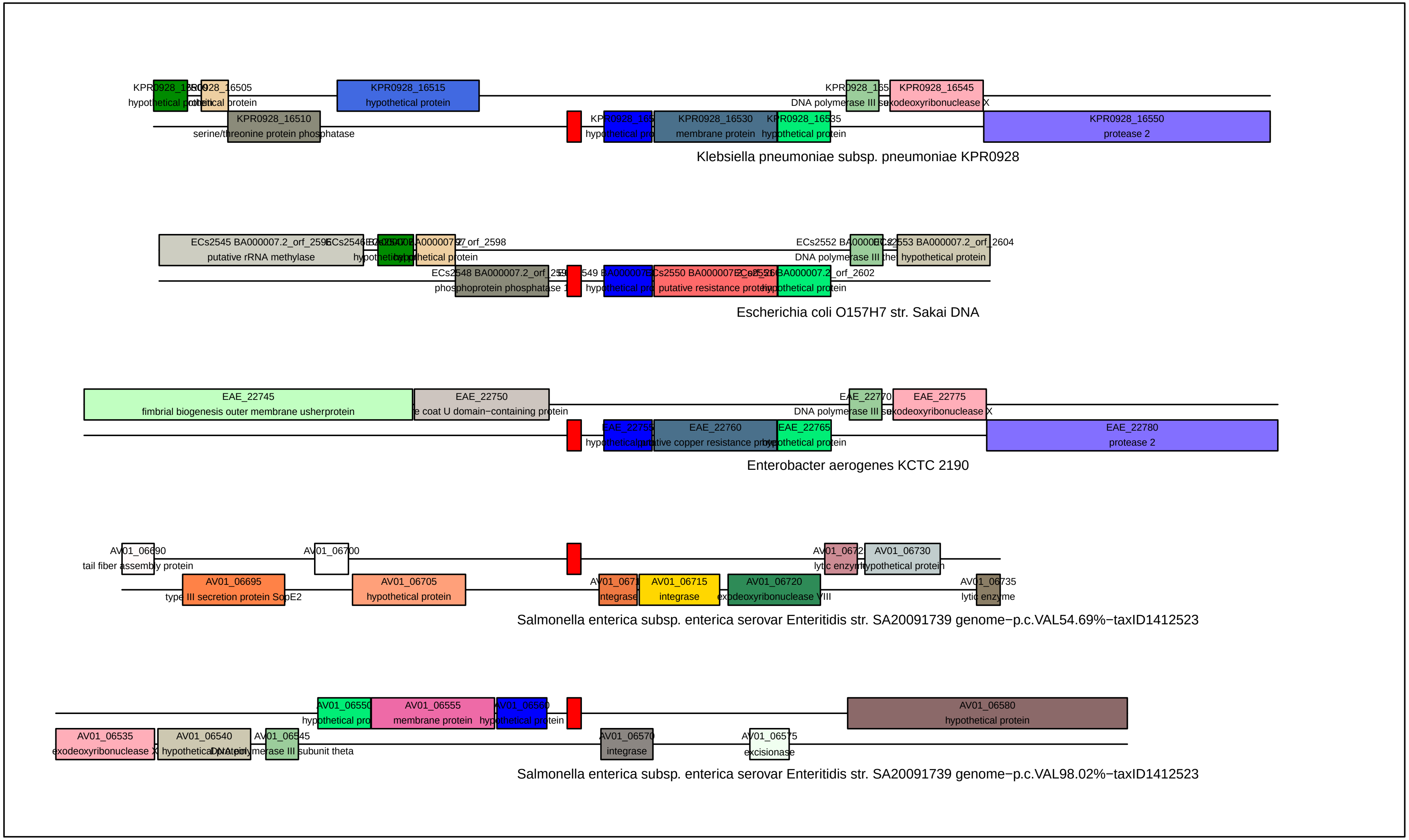

KPR0928_16505
KPR0928_16515
hypothetical protein
KPR0928_16510
serine/threonine protein phosphatase
KPR0928_16530
membrane protein
KPR0928_16535
hypothetical protein
KPR0928_16545
exodeoxyribonuclease X
KPR0928_16550
protease 2
Klebsiella pneumoniae subsp. pneumoniae KPR0928
putative rRNA methylase
ECs2548 BA000007.2_orf_259
phosphoprotein phosphatase 1
putative resistance protein
hypothetical protein
ECs2553 BA000007.2_orf_2604
Escherichia coli O157H7 str. Sakai DNA
EAE_22745
fimbrial biogenesis outer membrane usherprotein
EAE_22750
EAE_22760
EAE_22765
EAE_22770
EAE_22775
exodeoxyribonuclease X
EAE_22780
protease 2
Enterobacter aerogenes KCTC 2190
AV01_06690
tail fiber assembly protein
AV01_06700
AV01_06695
type III secretion protein SopE2
AV01_06705
hypothetical protein
AV01_06715
integrase
AV01_06720
exodeoxyribonuclease VIII
AV01_06730
hypothetical protein
AV01_06735
lytic enzyme
Salmonella enterica subsp. enterica serovar Enteritidis str. SA20091739 genome−p.c.VAL54.69%−taxID1412523
AV01_06550
AV01_06555
membrane protein
AV01_06560
hypothetical protein
AV01_06580
hypothetical protein
AV01_06535
exodeoxyribonuclease X
AV01_06540
AV01_06545
subunit theta
AV01_06570
integrase
AV01_06575
excisionase
Salmonella enterica subsp. enterica serovar Enteritidis str. SA20091739 genome−p.c.VAL98.02%−taxID1412523

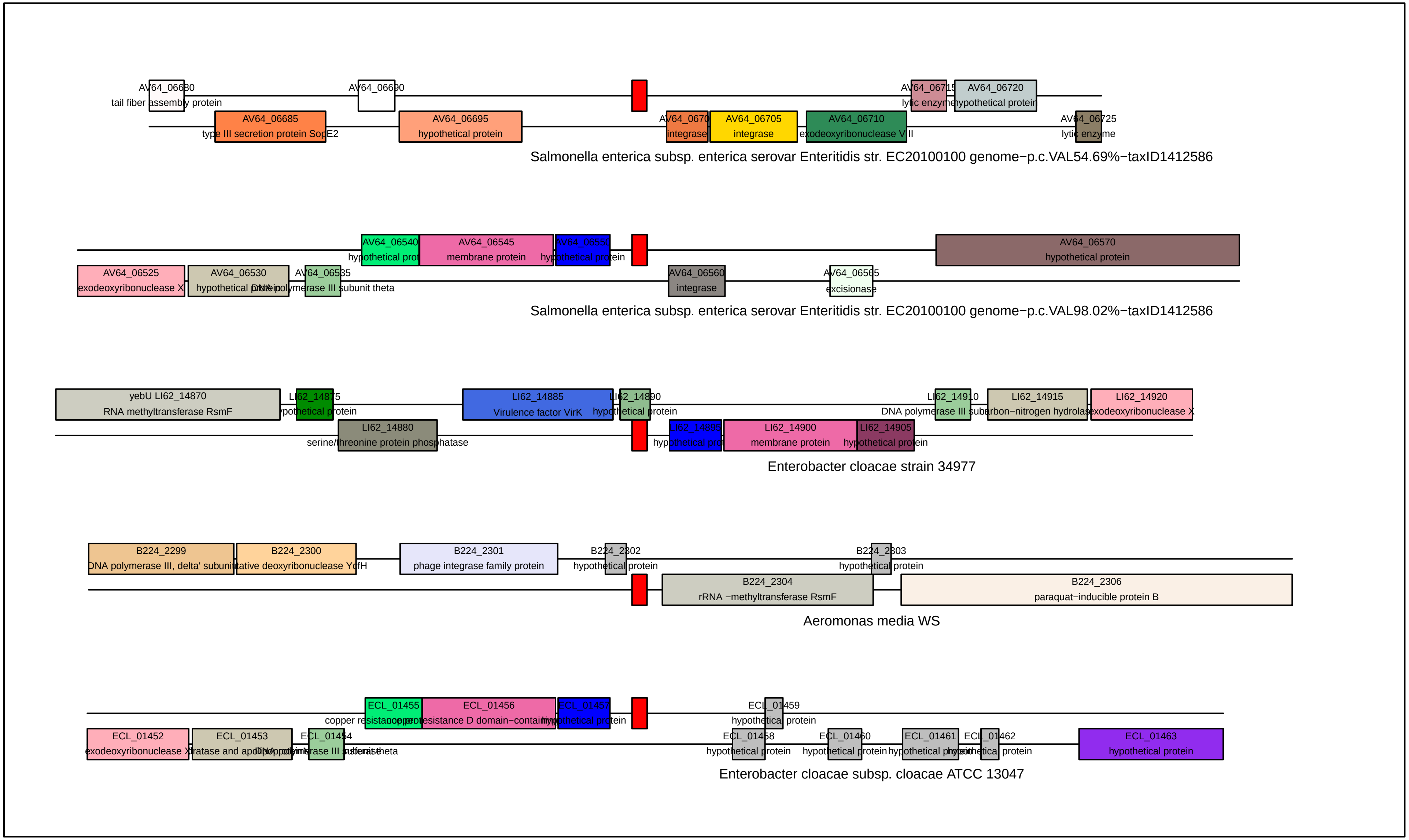

Salmonella enterica subsp. enterica serovar Enteritidis str. EC20100100 genome−p.c.VAL54.69%−taxID1412586
Salmonella enterica subsp. enterica serovar Enteritidis str. EC20100100 genome−p.c.VAL98.02%−taxID1412586
Enterobacter cloacae strain 34977
Aeromonas media WS
Enterobacter cloacae subsp. cloacae ATCC 13047

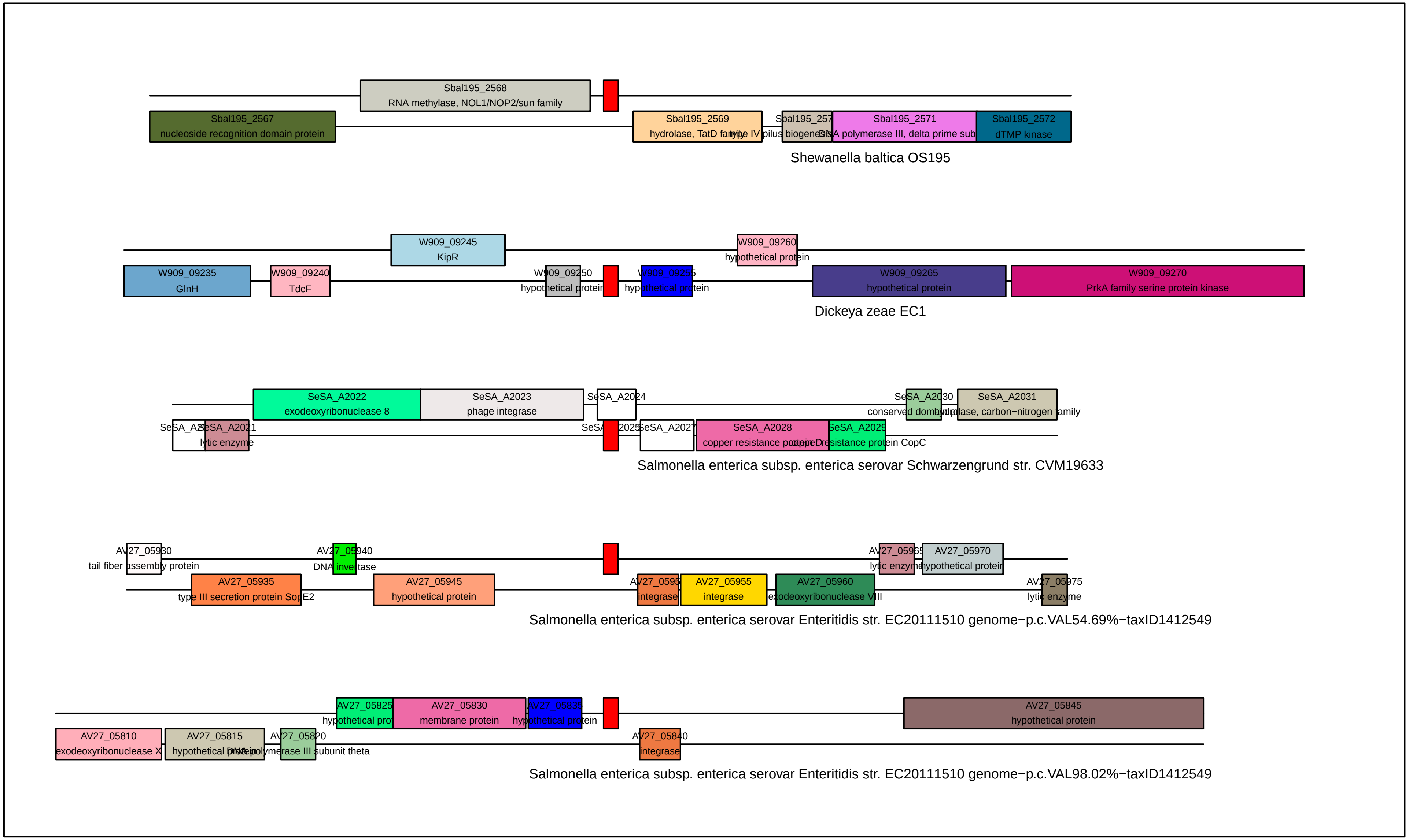

Sbal195_2568
RNA methylase, NOL1/NOP2/sun family
Sbal195_2567
nucleoside recognition domain protein
Sbal195_2569
hydrolase, TatD family
Sbal195_2571
Sbal195_2572
dTMP kinase
Shewanella baltica OS195
W909_09245
KipR
W909_09260
hypothetical protein
W909_09235
GlnH
W909_09240
TdcF
W909_09250
hypothetical protein
W909_09265
hypothetical protein
W909_09270
PrkA family serine protein kinase
Dickeya zeae EC1
SeSA_A2022
exodeoxyribonuclease 8
SeSA_A2023
phage integrase
SeSA_A2024
SeSA_A2030
SeSA_A2031
SeSA_A2021
lytic enzyme
SeSA_A2028
copper resistance protein
SeSA_A2029
Salmonella enterica subsp. enterica serovar Schwarzengrund str. CVM19633
AV27_05930
tail fiber assembly protein
AV27_05940
DNA invertase
AV27_05965
lytic enzyme
AV27_05970
hypothetical protein
AV27_05935
type III secretion protein SopE2
AV27_05945
hypothetical protein
integrase
AV27_05955
integrase
AV27_05960
exodeoxyribonuclease VIII
AV27_05975
lytic enzyme
Salmonella enterica subsp. enterica serovar Enteritidis str. EC20111510 genome−p.c.VAL54.69%−taxID1412549
AV27_05825
AV27_05830
membrane protein
AV27_05845
hypothetical protein
AV27_05810
exodeoxyribonuclease X
AV27_05815
AV27_05820
AV27_05840
integrase
Salmonella enterica subsp. enterica serovar Enteritidis str. EC20111510 genome−p.c.VAL98.02%−taxID1412549

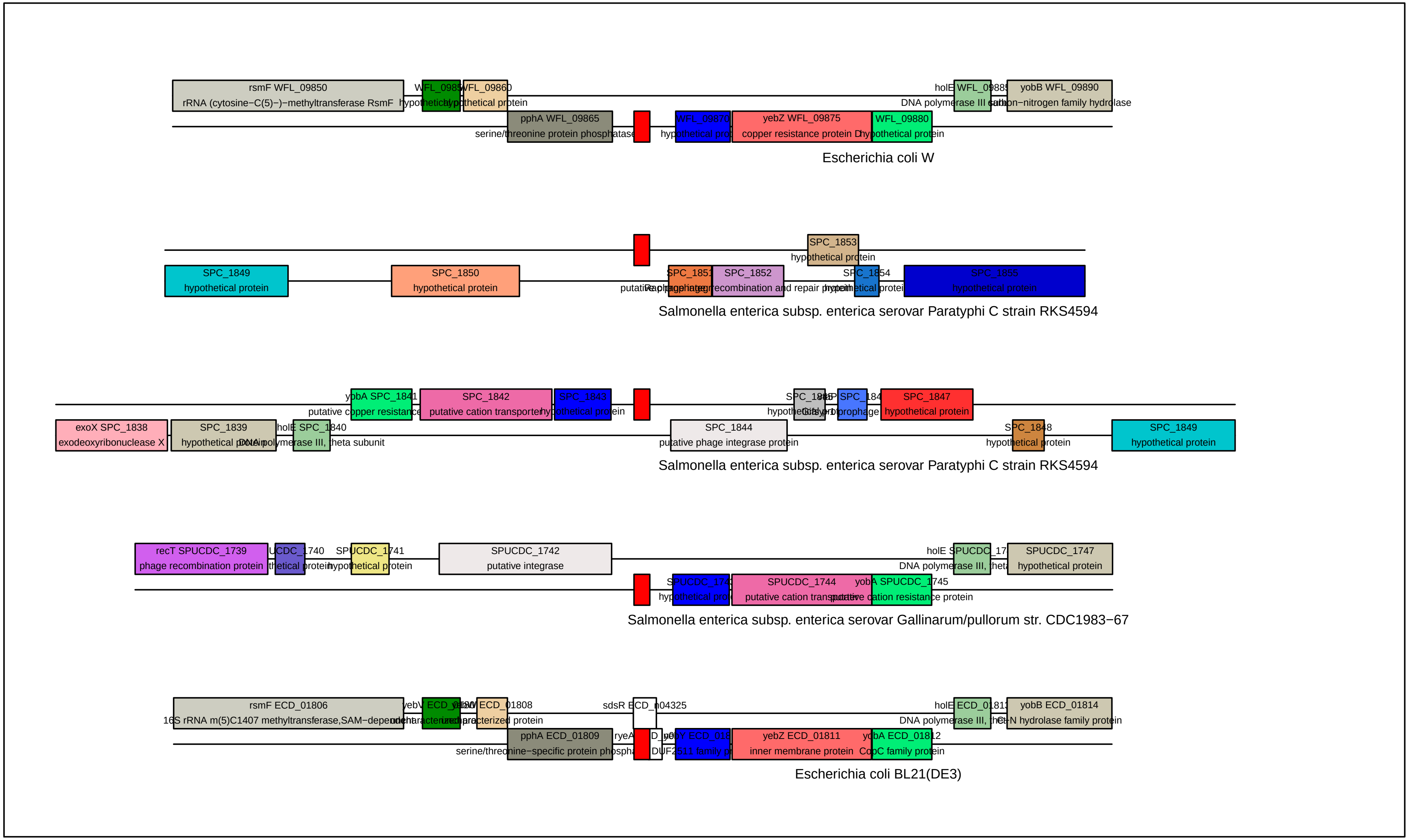

rsmF WFL_09850
rRNA (cytosine−C(5)−)−methyltransferase RsmF
pphA WFL_09865
serine/threonine protein phosphatase
yebZ WFL_09875
copper resistance protein D
WFL_09880
yobB WFL_09890
Escherichia coli W
SPC_1853
SPC_1849
hypothetical protein
SPC_1850
hypothetical protein
SPC_1852
SPC_1855
hypothetical protein
Salmonella enterica subsp. enterica serovar Paratyphi C strain RKS4594
SPC_1842
putative cation transporter
SPC_1843
SPC_1847
hypothetical protein
exoX SPC_1838
exodeoxyribonuclease X
SPC_1839
SPC_1844
putative phage integrase protein
SPC_1849
hypothetical protein
Salmonella enterica subsp. enterica serovar Paratyphi C strain RKS4594
recT SPUCDC_1739
phage recombination protein
SPUCDC_1742
putative integrase
SPUCDC_1747
hypothetical protein
SPUCDC_1744
Salmonella enterica subsp. enterica serovar Gallinarum/pullorum str. CDC1983−67
rsmF ECD_01806
pphA ECD_01809
sdsR ECD_r04325
yebZ ECD_01811
inner membrane protein
yobB ECD_01814
C−N hydrolase family protein
Escherichia coli BL21(DE3)

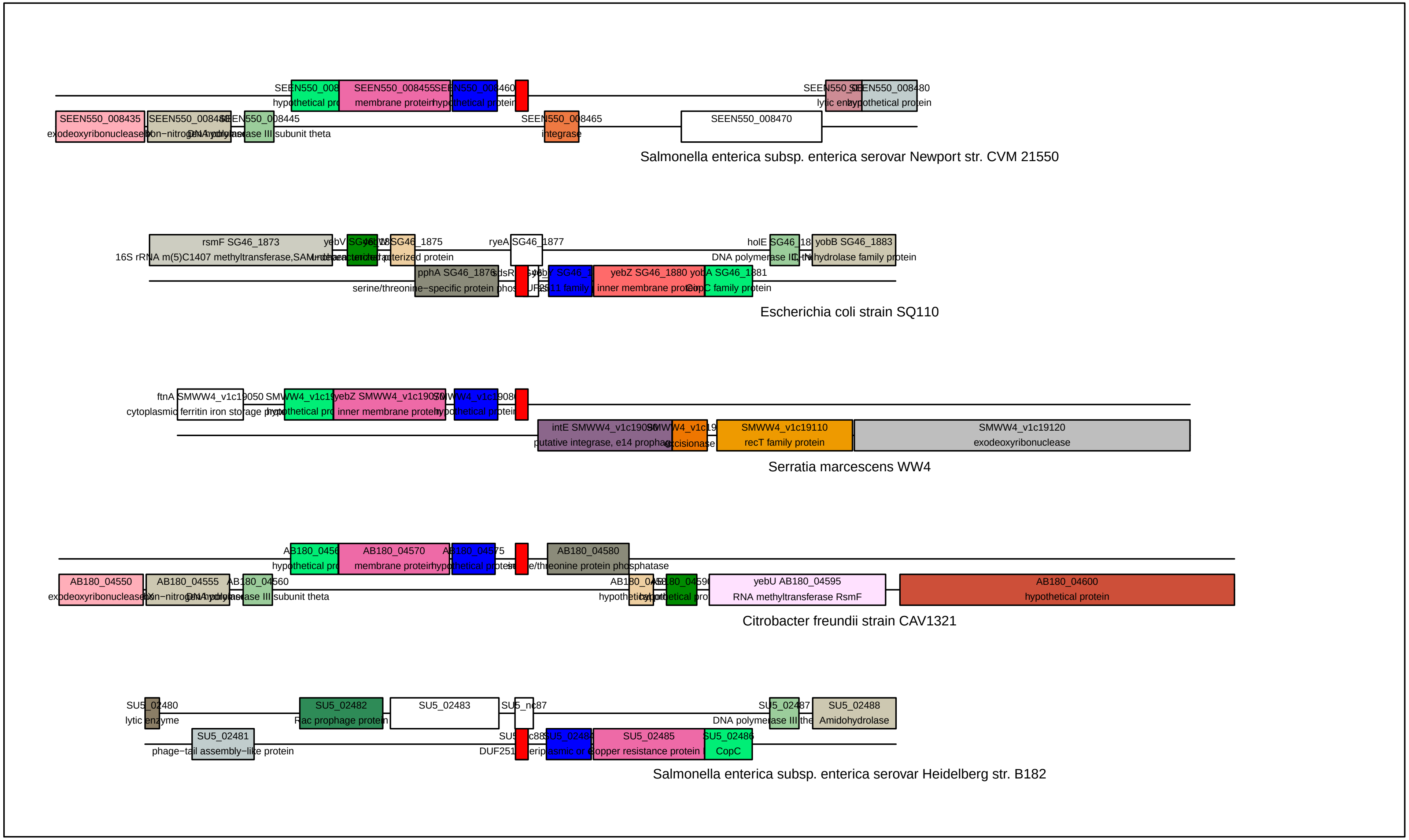

Salmonella enterica subsp. enterica serovar Newport str. CVM 21550
SEEN550_008435
SEEN550_008465
integrase
SEEN550_008470
Escherichia coli strain SQ110
rsmF SG46_1873
yobB SG46_1883
Serratia marcescens WW4
SMWW4_v1c19110
recT family protein
SMWW4_v1c19120
exodeoxyribonuclease
Citrobacter freundii strain CAV1321
AB180_04550
AB180_04570
AB180_04580
yebU AB180_04595
RNA methyltransferase RsmF
AB180_04600
hypothetical protein
Salmonella enterica subsp. enterica serovar Heidelberg str. B182
SU5_02482
Rac prophage protein
SU5_02483
SU5_02481
SU5_02485
SU5_02488
Amidohydrolase

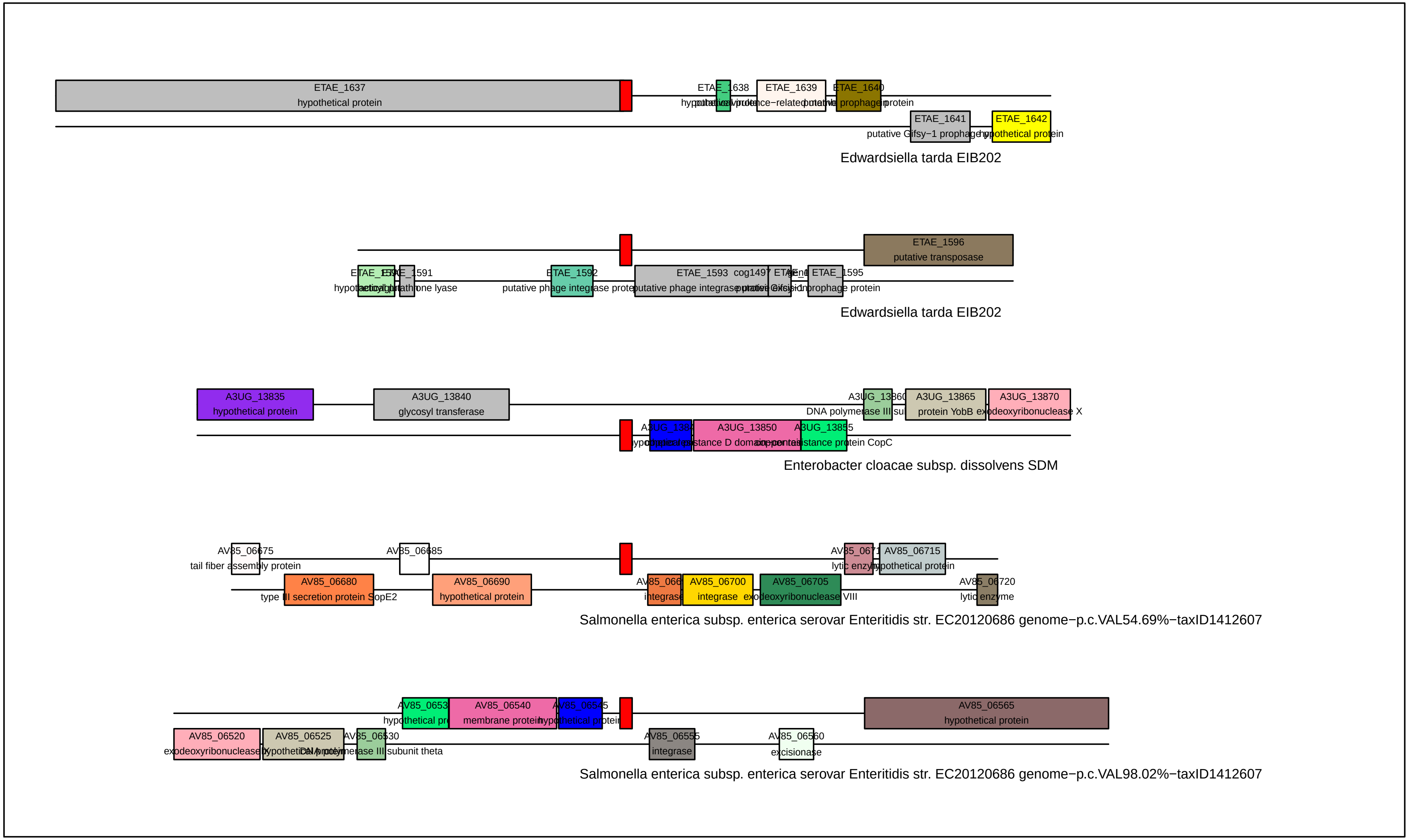

ETAE_1637
hypothetical protein
ETAE_1639
ETAE_1641
ETAE_1642
hypothetical protein
Edwardsiella tarda EIB202
ETAE_1596
putative transposase
ETAE_1592
ETAE_1593
ETAE_1595
Edwardsiella tarda EIB202
A3UG_13835
hypothetical protein
A3UG_13840
glycosyl transferase
A3UG_13865
protein YobB
A3UG_13870
exodeoxyribonuclease X
A3UG_13850
Enterobacter cloacae subsp. dissolvens SDM
AV85_06675
tail fiber assembly protein
AV85_06685
AV85_06680
type III secretion protein SopE2
AV85_06690
hypothetical protein
AV85_06700
integrase
AV85_06705
exodeoxyribonuclease VIII
AV85_06715
hypothetical protein
AV85_06720
lytic enzyme
Salmonella enterica subsp. enterica serovar Enteritidis str. EC20120686 genome−p.c.VAL54.69%−taxID1412607
AV85_06540
membrane protein
AV85_06565
hypothetical protein
AV85_06520
exodeoxyribonuclease
AV85_06525
AV85_06530
AV85_06555
integrase
AV85_06560
excisionase
Salmonella enterica subsp. enterica serovar Enteritidis str. EC20120686 genome−p.c.VAL98.02%−taxID1412607

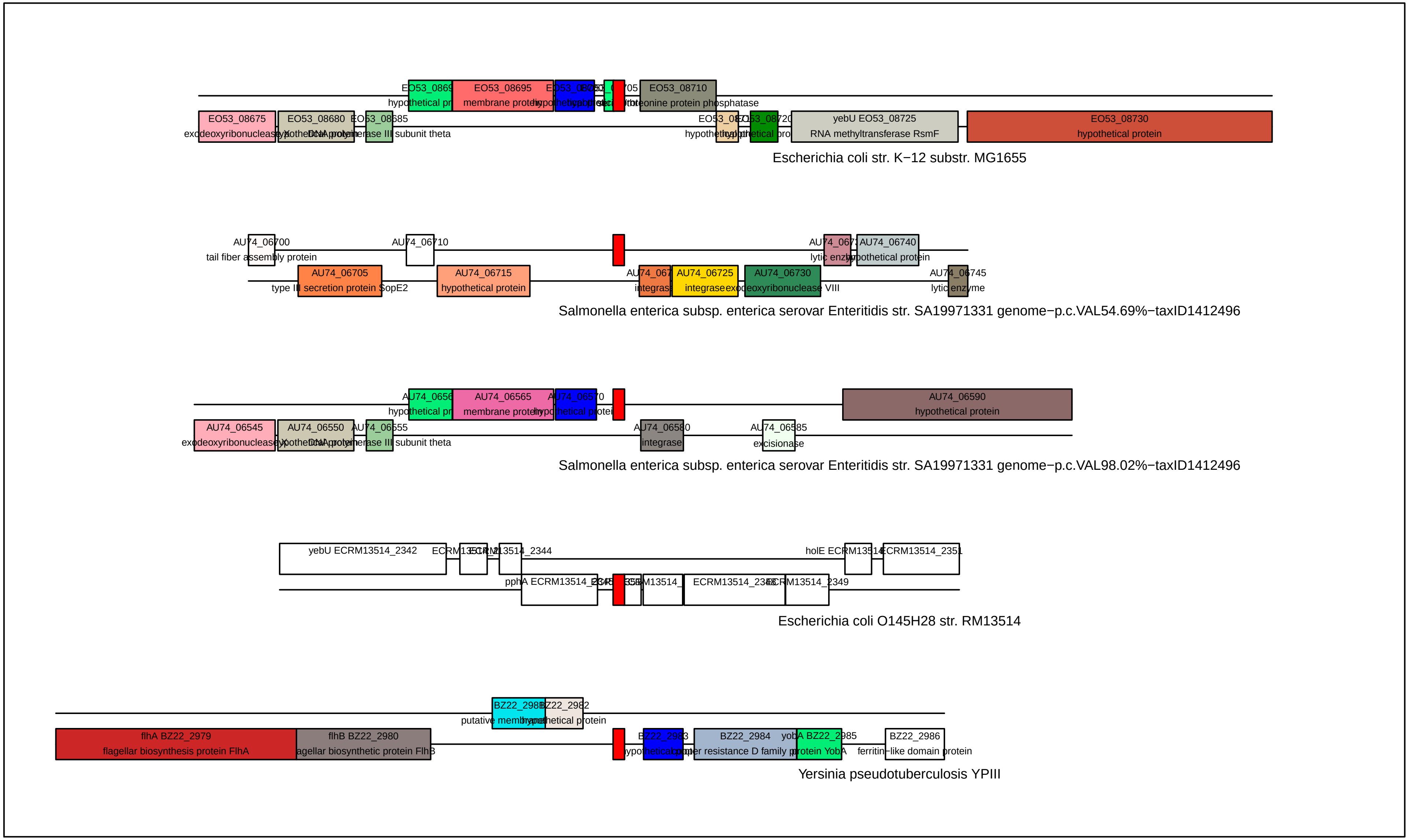
EO53_08695
membrane protein
EO53_08710
threonine protein phosphatase
EO53_08675
EO53_08680
EO53_08685
subunit theta
yebU EO53_08725
RNA methyltransferase RsmF
EO53_08730
hypothetical protein
Escherichia coli str. K−12 substr. MG1655
AU74_06700
tail fiber assembly protein
AU74_06710
AU74_06740
hypothetical protein
AU74_06705
type III secretion protein SopE2
AU74_06715
hypothetical protein
AU74_06725
integrase
AU74_06730
exodeoxyribonuclease VIII
AU74_06745
lytic enzyme
Salmonella enterica subsp. enterica serovar Enteritidis str. SA19971331 genome−p.c.VAL54.69%−taxID1412496
AU74_06565
membrane protein
AU74_06570
AU74_06590
hypothetical protein
AU74_06545
AU74_06550
AU74_06555
subunit theta
AU74_06580
integrase
AU74_06585
excisionase
Salmonella enterica subsp. enterica serovar Enteritidis str. SA19971331 genome−p.c.VAL98.02%−taxID1412496
yebU ECRM13514_2342
ECRM13514_2351
pphA ECRM13514
ECRM13514_2349
Escherichia coli O145H28 str. RM13514
BZ22_2982
hypothetical protein
flhA BZ22_2979
flagellar biosynthesis protein FlhA
flhB BZ22_2980
BZ22_2984
BZ22_2986
ferritin−like domain protein
Yersinia pseudotuberculosis YPIII

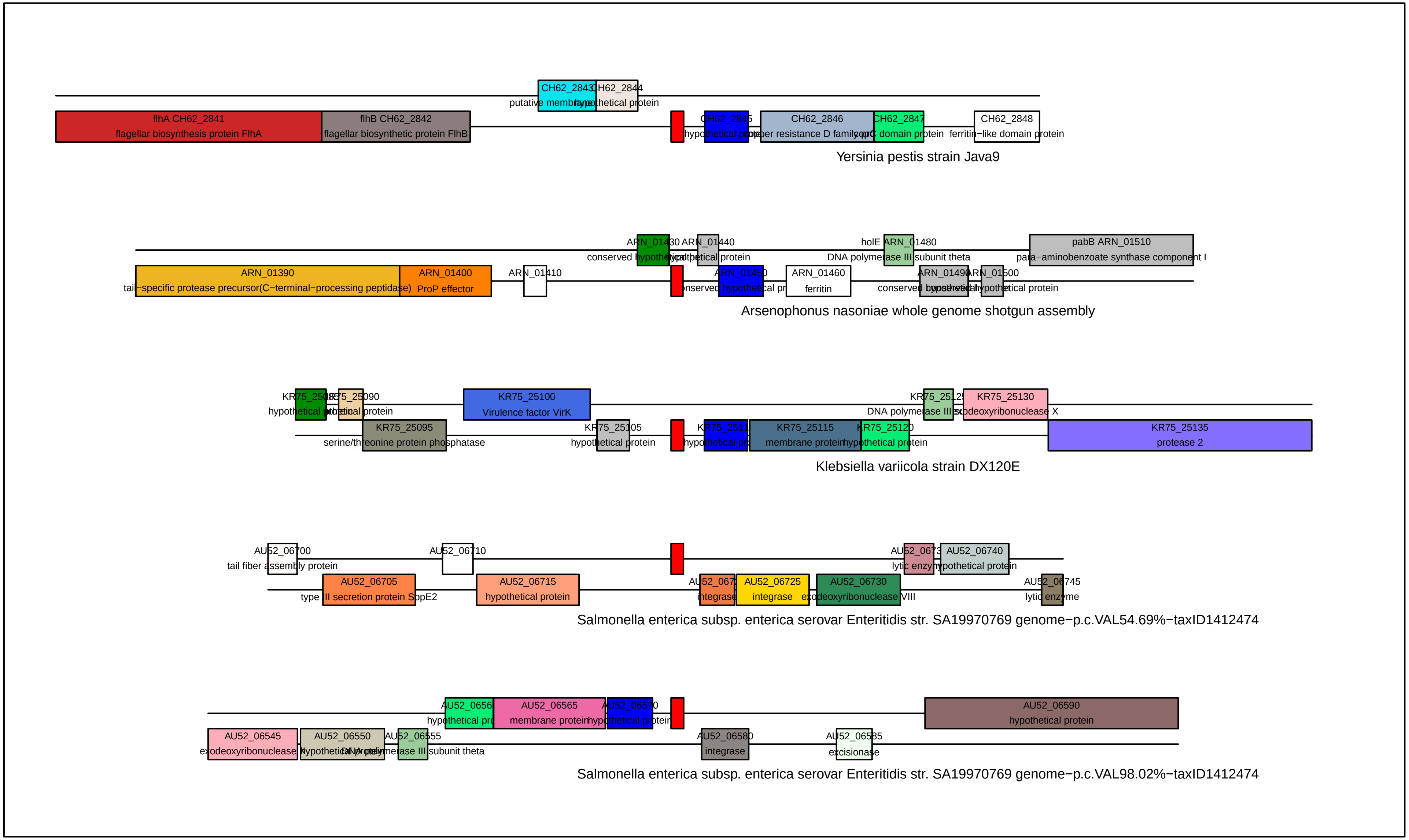
flhA CH62_2841
flagellar biosynthesis protein FlhA
flhB CH62_2842
flagellar biosynthetic protein FlhB
CH62_2844
CH62_2846
CH62_2847
CH62_2848
ferritin−like domain protein
Yersinia pestis strain Java9
ARN_01390
tail−specific protease precursor(C−terminal−processing peptidase)
ARN_01400
ProP effector
ARN_01410
ARN_01460
ferritin
holE ARN_01480
DNA polymerase III subunit theta
pabB ARN_01510
para−aminobenzoate synthase component I
Arsenophonus nasoniae whole genome shotgun assembly
KR75_25100
Virulence factor VirK
KR75_25095
serine/threonine protein phosphatase
KR75_25105
hypothetical protein
KR75_25115
membrane protein
KR75_25120
KR75_25130
exodeoxyribonuclease X
KR75_25135
protease 2
Klebsiella variicola strain DX120E
AU52_06700
tail fiber assembly protein
AU52_06705
type III secretion protein SopE2
AU52_06710
AU52_06715
hypothetical protein
AU52_06725
integrase
AU52_06730
exodeoxyribonuclease VIII
AU52_06740
hypothetical protein
AU52_06745
lytic enzyme
Salmonella enterica subsp. enterica serovar Enteritidis str. SA19970769 genome−p.c.VAL54.69%−taxID1412474
AU52_06545
exodeoxyribonuclease X
AU52_06550
AU52_06555
DNA polymerase III subunit theta
AU52_06565
membrane protein
AU52_06580
integrase
AU52_06585
excisionase
AU52_06590
hypothetical protein
Salmonella enterica subsp. enterica serovar Enteritidis str. SA19970769 genome−p.c.VAL98.02%−taxID1412474

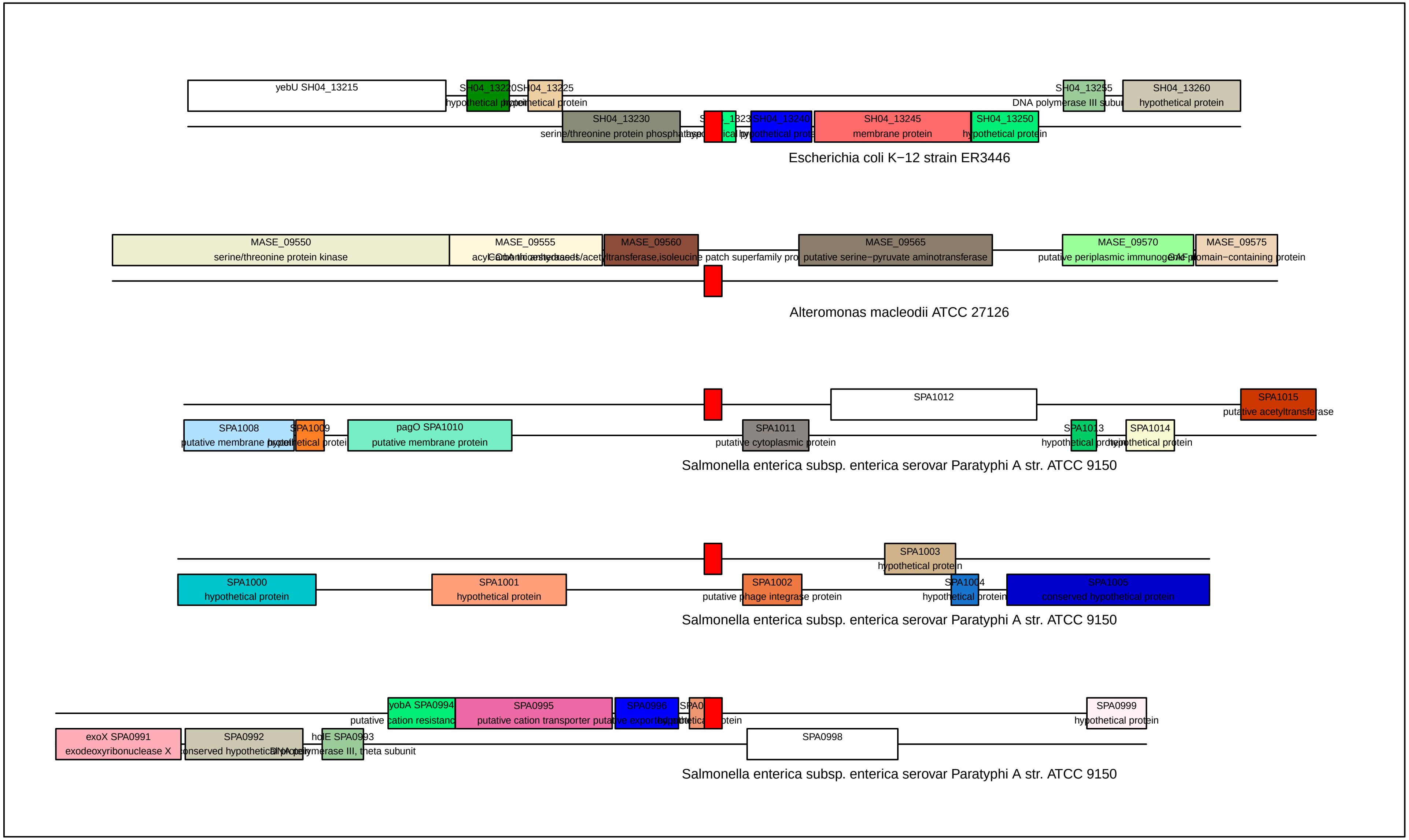
yebU SH04_13215
SH04_13230
serine/threonine protein phosphatase
SH04_13245
membrane protein
SH04_13250
hypothetical protein
DNA polymerase III subun
SH04_13260
hypothetical protein
Escherichia coli K−12 strain ER3446
MASE_09550
serine/threonine protein kinase
MASE_09555
MASE_09560
MASE_09565
putative serine−pyruvate aminotransferase
MASE_09570
MASE_09575
Alteromonas macleodii ATCC 27126
SPA1008
SPA1009
pagO SPA1010
putative membrane protein
SPA1011
putative cytoplasmic protein
SPA1012
SPA1013
SPA1014
SPA1015
putative acetyltransferase
Salmonella enterica subsp. enterica serovar Paratyphi A str. ATCC 9150
SPA1000
hypothetical protein
SPA1001
hypothetical protein
SPA1002
putative phage integrase protein
SPA1003
hypothetical protein
SPA1004
SPA1005
conserved hypothetical protein
Salmonella enterica subsp. enterica serovar Paratyphi A str. ATCC 9150
exoX SPA0991
exodeoxyribonuclease X
SPA0992
holE SPA0993
DNA polymerase III, theta subunit
yobA SPA0994
SPA0995
putative cation transporter
SPA0996
SPA0998
SPA0999
hypothetical protein
Salmonella enterica subsp. enterica serovar Paratyphi A str. ATCC 9150

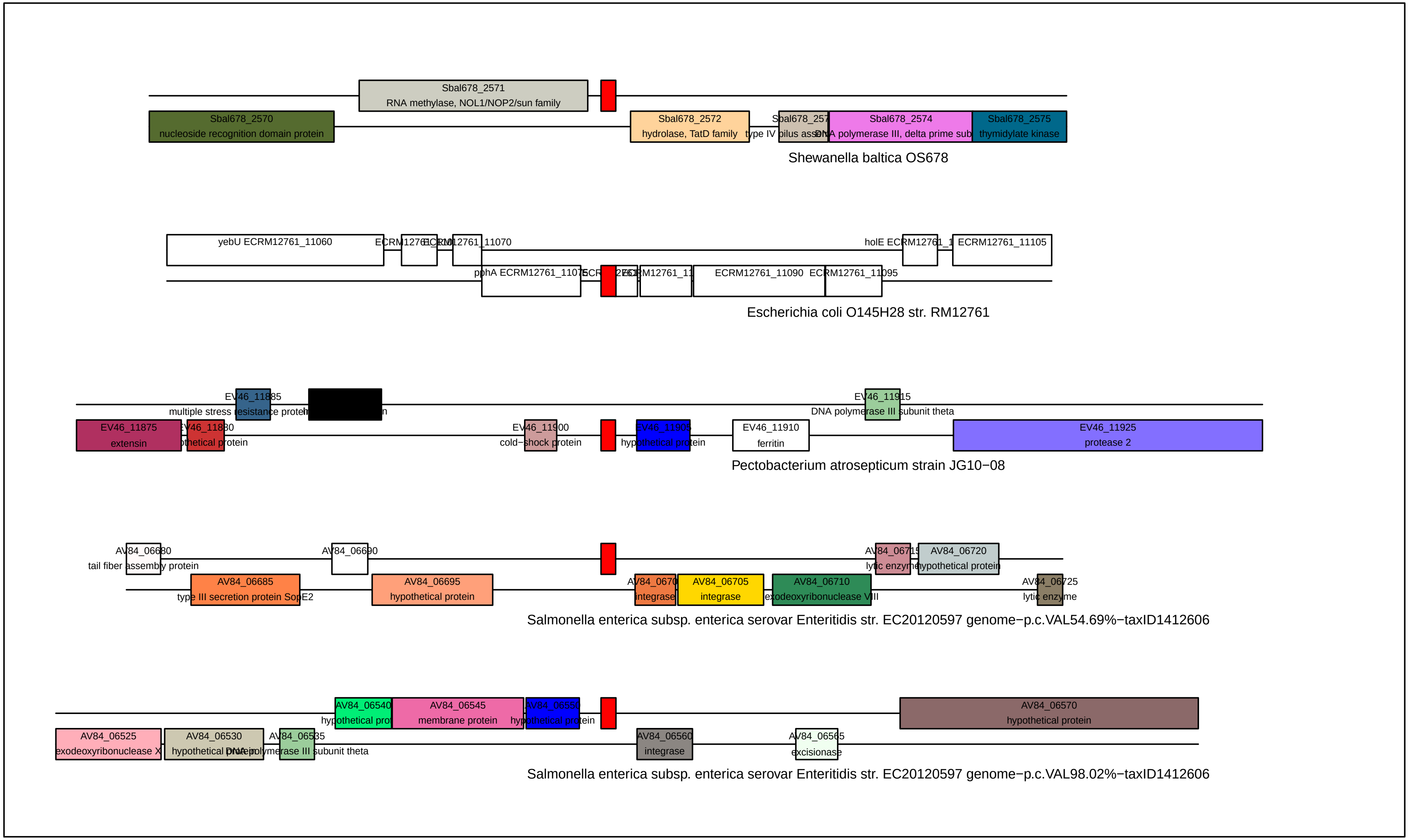
Sbal678_2571
RNA methylase, NOL1/NOP2/sun family
Sbal678_2570
nucleoside recognition domain protein
Sbal678_2572
hydrolase, TatD family
Sbal678_2574
Sbal678_2575
thymidylate kinase
Shewanella baltica OS678
yebU ECRM12761_11060
ECRM12761_11105
ECRM12761_11090
Escherichia coli O145H28 str. RM12761
multiple stress resistance protein
EV46_11875
extensin
ferritin
EV46_11910
DNA polymerase III subunit theta
EV46_11925
protease 2
cold−shock protein
Pectobacterium atrosepticum strain JG10−08
tail fiber assembly protein
AV84_06685
type III secretion protein SopE2
AV84_06695
hypothetical protein
AV84_06705
integrase
AV84_06710
exodeoxyribonuclease VIII
AV84_06720
Salmonella enterica subsp. enterica serovar Enteritidis str. EC20120597 genome−p.c.VAL54.69%−taxID1412606
AV84_06545
membrane protein
AV84_06570
hypothetical protein
AV84_06525
exodeoxyribonuclease X
AV84_06530
AV84_06560
integrase
excisionase
Salmonella enterica subsp. enterica serovar Enteritidis str. EC20120597 genome−p.c.VAL98.02%−taxID1412606

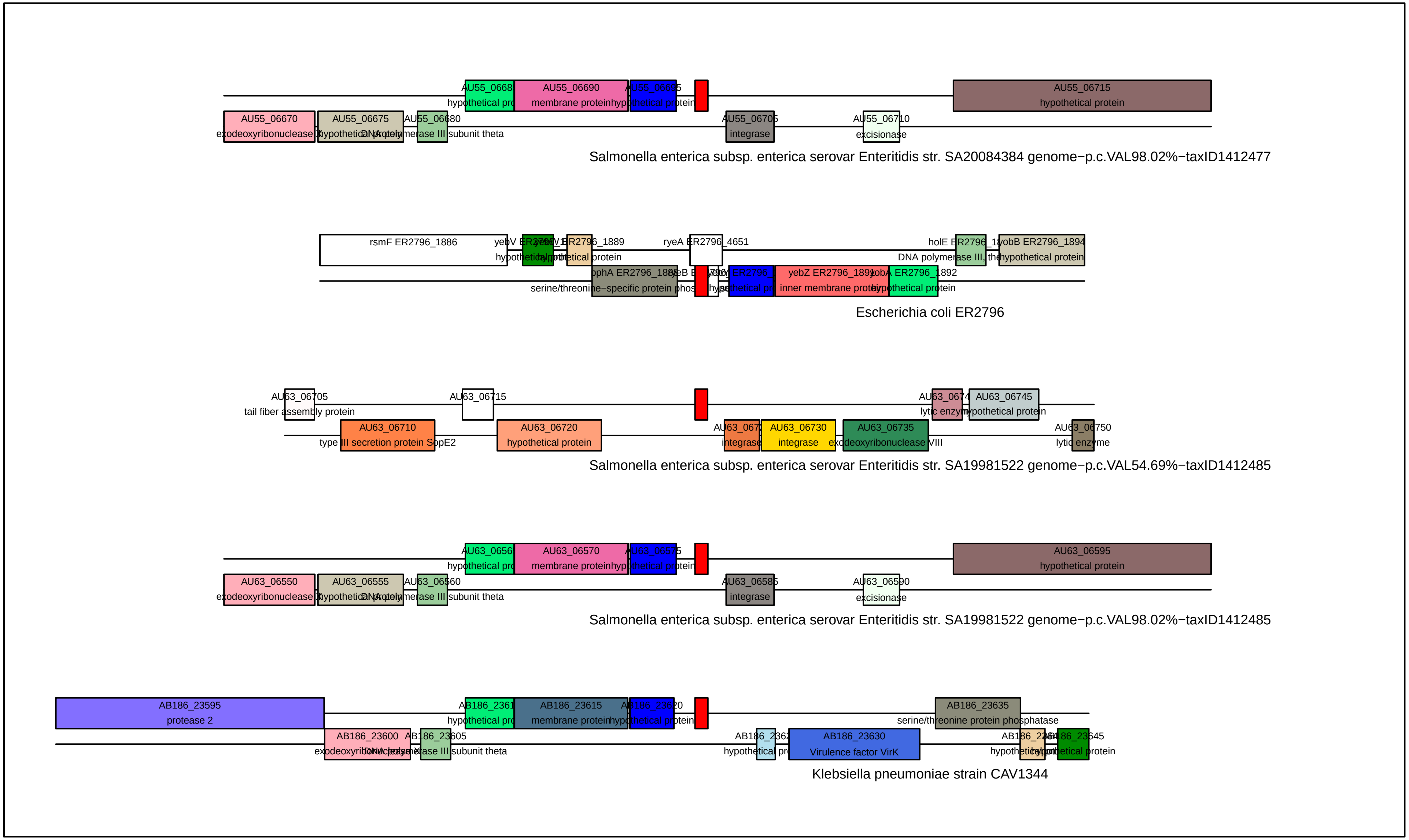
AU55_06690
membrane protein
AU55_06715
hypothetical protein
AU55_06670
AU55_06675
AU55_06705
integrase
excisionase
Salmonella enterica subsp. enterica serovar Enteritidis str. SA20084384 genome−p.c.VAL98.02%−taxID1412477
rsmF ER2796_1886
ryeA ER2796_4651
holE ER2796_1
yobB ER2796_1894
hypothetical protein
yebZ ER2796_1891
inner membrane protein
serine/threonine−specific protein phos
Escherichia coli ER2796
AU63_06705
tail fiber assembly protein
AU63_06715
AU63_06745
AU63_06710
type III secretion protein SopE2
AU63_06720
hypothetical protein
AU63_06730
integrase
AU63_06735
AU63_06750
lytic enzyme
Salmonella enterica subsp. enterica serovar Enteritidis str. SA19981522 genome−p.c.VAL54.69%−taxID1412485
AU63_06570
membrane protein
AU63_06595
hypothetical protein
AU63_06550
AU63_06555
AU63_06585
integrase
AU63_06590
excisionase
Salmonella enterica subsp. enterica serovar Enteritidis str. SA19981522 genome−p.c.VAL98.02%−taxID1412485
AB186_23595
protease 2
AB186_23615
membrane protein
AB186_23635
serine/threonine protein phosphatase
AB186_23600
AB186_23605
AB186_23630
Virulence factor VirK
Klebsiella pneumoniae strain CAV1344

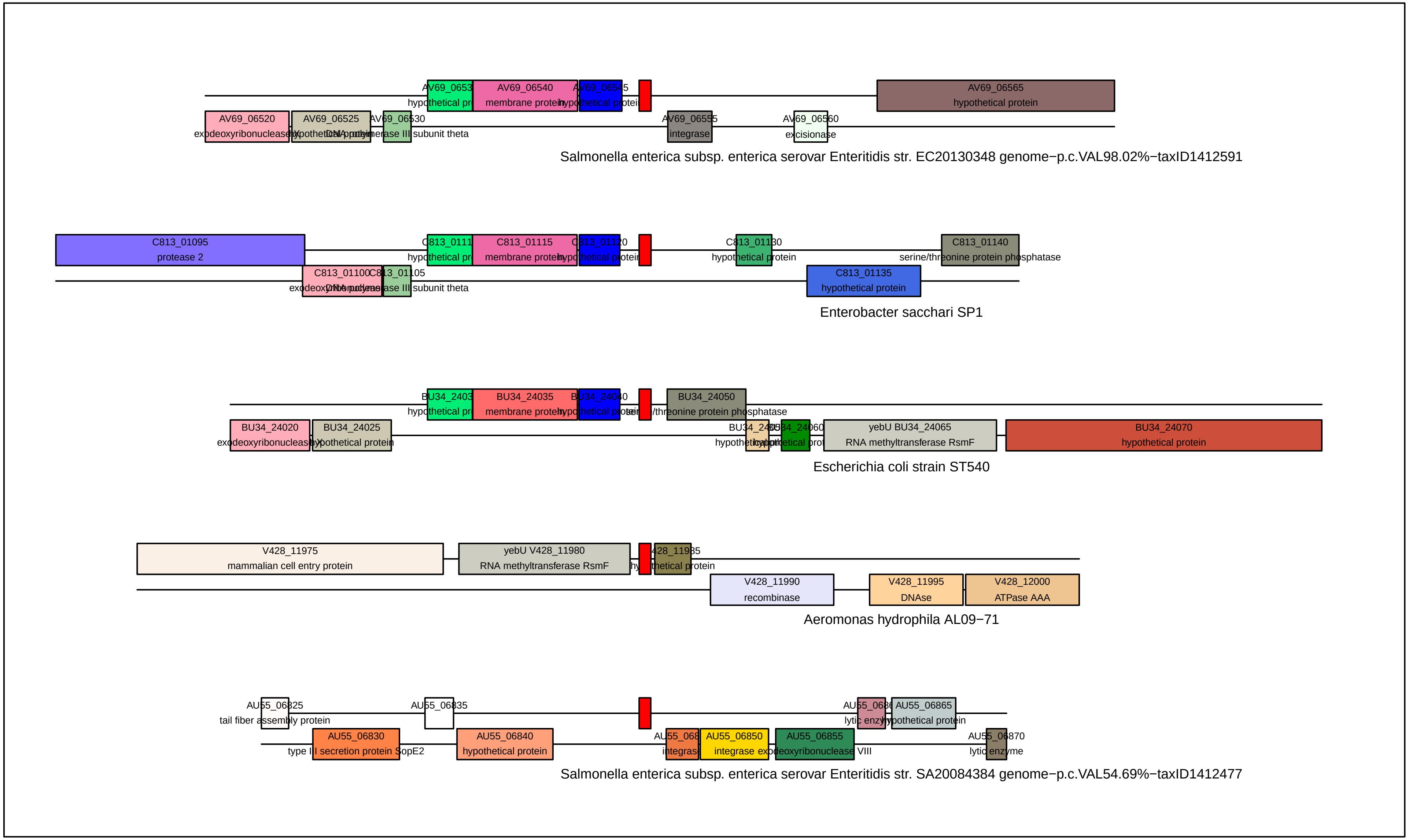
AV69_06540
membrane protein
AV69_06565
hypothetical protein
AV69_06520
AV69_06525
AV69_06555
integrase
AV69_06560
excisionase
Salmonella enterica subsp. enterica serovar Enteritidis str. EC20130348 genome−p.c.VAL98.02%−taxID1412591
C813_01095
protease 2
C813_01115
membrane protein
C813_01140
serine/threonine protein phosphatase
C813_01135
hypothetical protein
Enterobacter sacchari SP1
BU34_24035
membrane protein
BU34_24050
BU34_24020
BU34_24025
hypothetical protein
yebU BU34_24065
RNA methyltransferase RsmF
BU34_24070
hypothetical protein
Escherichia coli strain ST540
V428_11975
mammalian cell entry protein
yebU V428_11980
RNA methyltransferase RsmF
V428_11990
recombinase
V428_11995
DNAse
V428_12000
ATPase AAA
Aeromonas hydrophila AL09−71
tail fiber assembly protein
AU55_06830
AU55_06840
hypothetical protein
AU55_06850
AU55_06855
AU55_06865
Salmonella enterica subsp. enterica serovar Enteritidis str. SA20084384 genome−p.c.VAL54.69%−taxID1412477

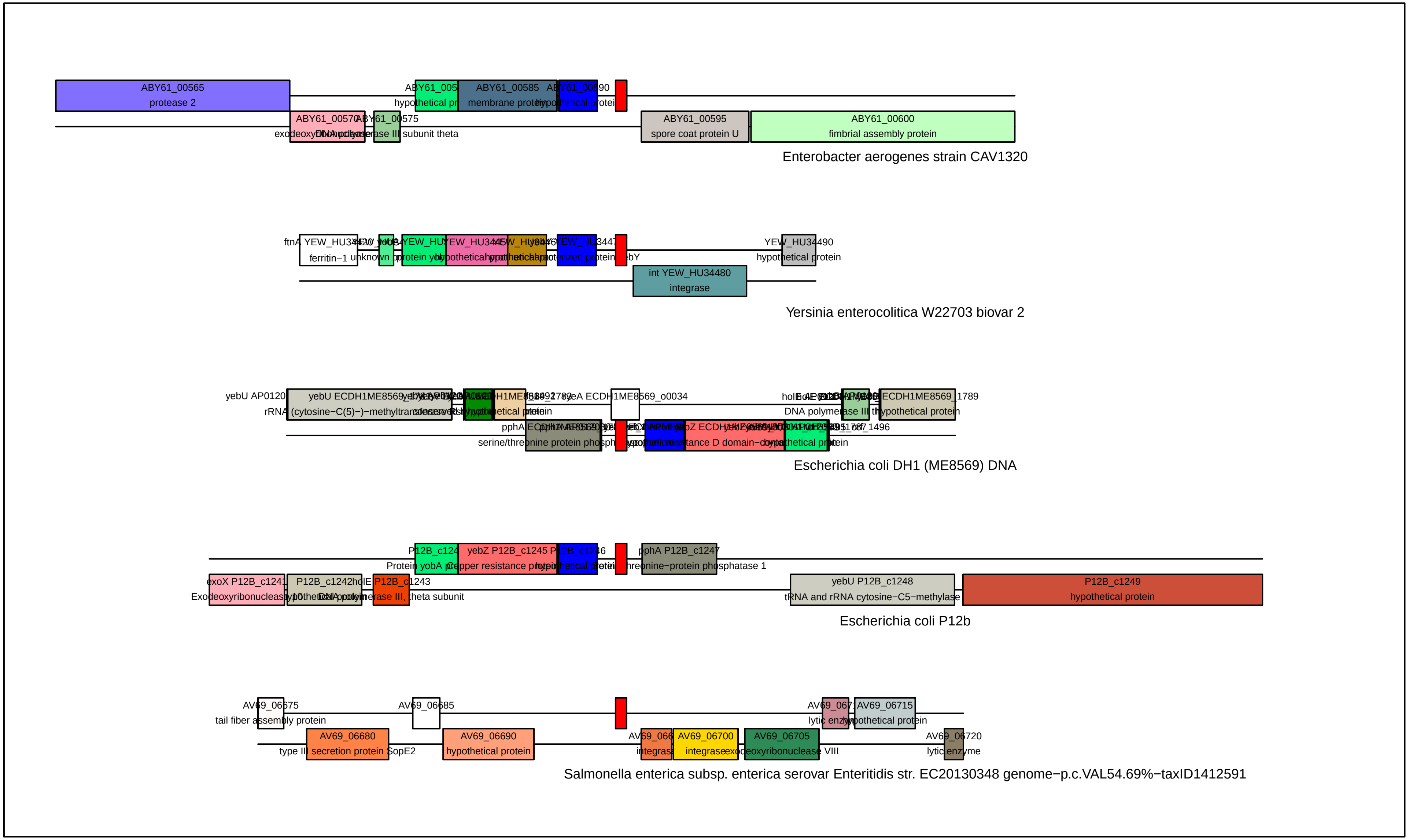

ABY61_00565
protease 2
ABY61_00585
membrane protein
ABY61_00595
spore coat protein U
ABY61_00600
fimbrial assembly protein
ABY61_00575
subunit theta
Enterobacter aerogenes strain CAV1320
ftnA
ferritin−1
YEW_HU34490
hypothetical protein
int YEW_HU34480
integrase
Yersinia enterocolitica W22703 biovar 2
yebU AP0120
rRNA (cytosine−C(5)−)−methyltransferase RsmF
ECDH1ME8569_o0034
ECDH1ME8569_1789
hypothetical protein
DNA polymerase III
serine/threonine protein phosphatase
Escherichia coli DH1 (ME8569) DNA
yebZ P12B_c1245
pphA P12B_c1247
exoX P12B_c1241
Exodeoxyribonuclease
P12B_c1243
yebU P12B_c1248
tRNA and rRNA cytosine−C5−methylase
P12B_c1249
hypothetical protein
Escherichia coli P12b
AV69_06675
tail fiber assembly protein
AV69_06685
AV69_06680
type III secretion protein SopE2
AV69_06690
hypothetical protein
AV69_06700
integrase
AV69_06705
exodeoxyribonuclease VIII
AV69_06715
hypothetical protein
AV69_06720
lytic enzyme
Salmonella enterica subsp. enterica serovar Enteritidis str. EC20130348 genome−p.c.VAL54.69%−taxID1412591

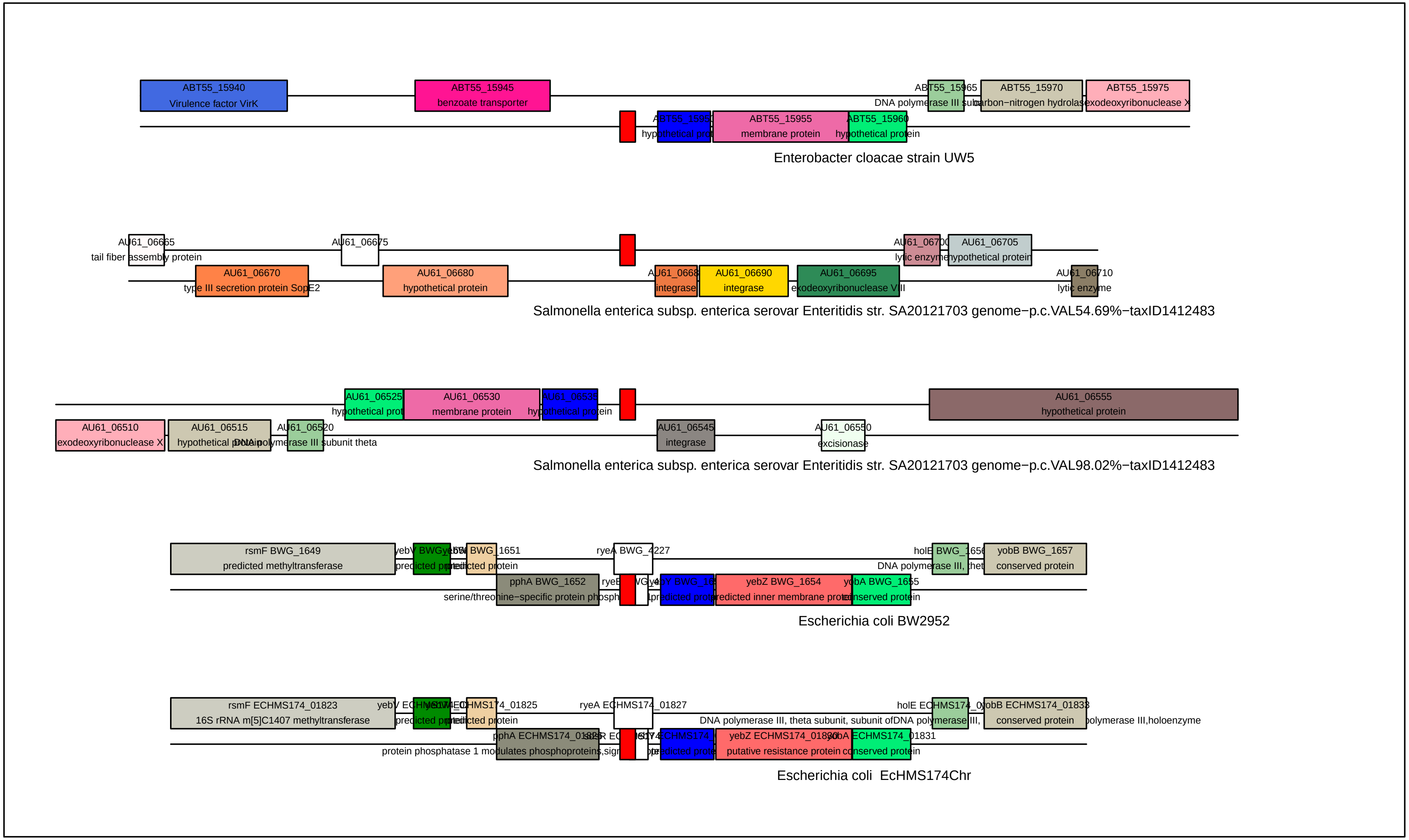
ABT55_15940
Virulence factor VirK
ABT55_15945
benzoate transporter
ABT55_15965
DNA polymerase III
ABT55_15970
ABT55_15975
exodeoxyribonuclease X
ABT55_15955
membrane protein
ABT55_15960
hypothetical protein
Enterobacter cloacae strain UW5
AU61_06665
tail fiber assembly protein
AU61_06675
AU61_06705
hypothetical protein
AU61_06670
type III secretion protein SopE2
AU61_06680
hypothetical protein
integrase
AU61_06690
integrase
AU61_06695
exodeoxyribonuclease VIII
AU61_06710
lytic enzyme
Salmonella enterica subsp. enterica serovar Enteritidis str. SA20121703 genome−p.c.VAL54.69%−taxID1412483
AU61_06525
AU61_06530
membrane protein
AU61_06555
hypothetical protein
AU61_06510
exodeoxyribonuclease X
AU61_06515
AU61_06520
subunit theta
AU61_06545
integrase
AU61_06550
excisionase
Salmonella enterica subsp. enterica serovar Enteritidis str. SA20121703 genome−p.c.VAL98.02%−taxID1412483
rsmF BWG_1649
predicted methyltransferase
predicted protein
ryeA BWG_4227
yobB BWG_1657
conserved protein
pphA BWG_1652
yebZ BWG_1654
conserved protein
Escherichia coli BW2952
rsmF ECHMS174_01823
16S rRNA m[5]C1407 methyltransferase
ryeA ECHMS174_01827
conserved protein
putative resistance protein
conserved protein
Escherichia coli EcHMS174Chr

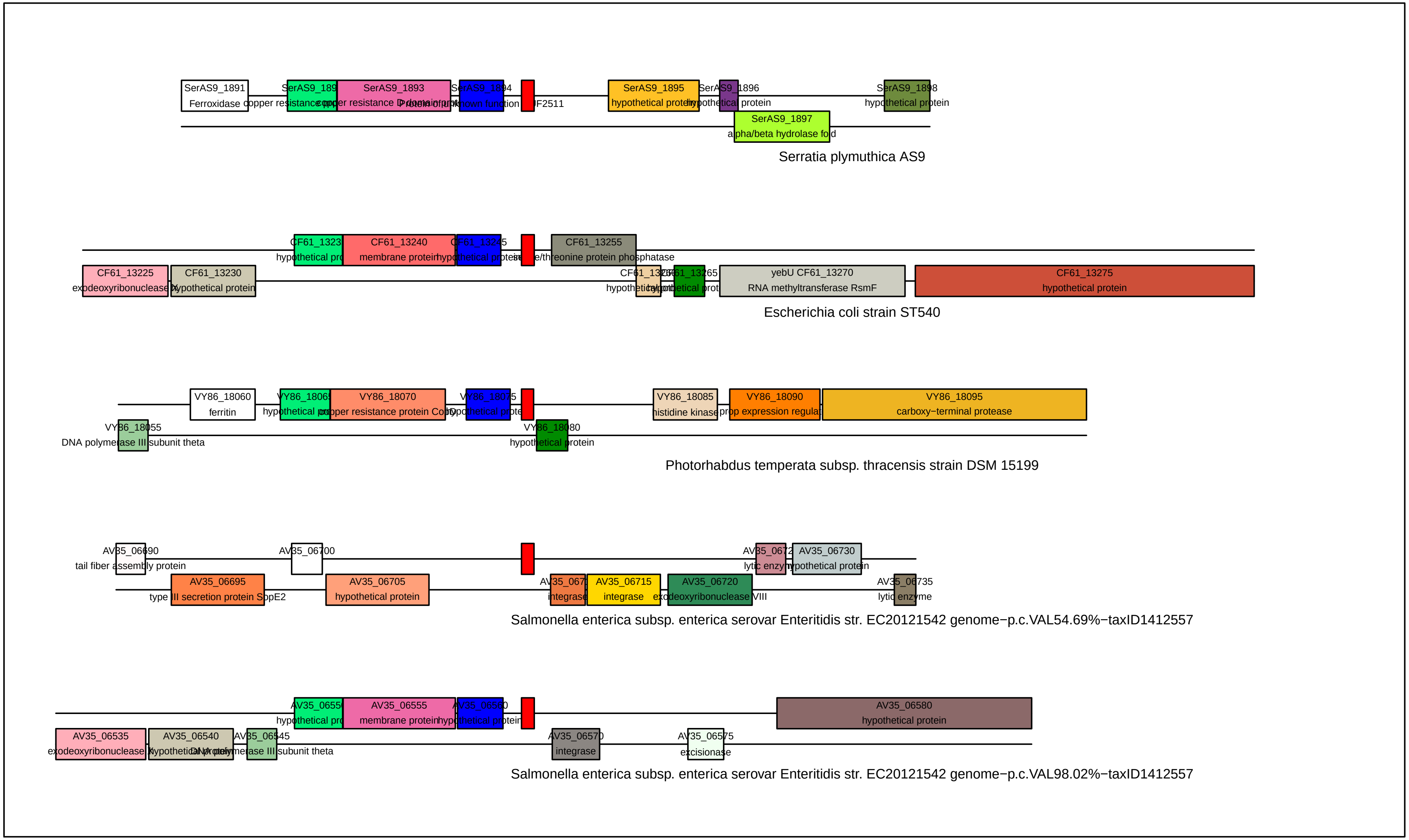

SerAS9_1891
Ferroxidase
SerAS9_1893
SerAS9_1895
hypothetical protein
SerAS9_1897
alpha/beta hydrolase fold
SerAS9_1898
hypothetical protein
Serratia plymuthica AS9
CF61_13240
membrane protein
CF61_13255
CF61_13225
CF61_13230
hypothetical protein
yebU CF61_13270
RNA methyltransferase RsmF
CF61_13275
hypothetical protein
Escherichia coli strain ST540
VY86_18060
ferritin
VY86_18070
VY86_18085
VY86_18090
VY86_18095
carboxy−terminal protease
VY86_18055
DNA polymerase III subunit theta
VY86_18080
hypothetical protein
Photorhabdus temperata subsp. thracensis strain DSM 15199
AV35_06690
tail fiber assembly protein
AV35_06700
AV35_06730
AV35_06695
type III secretion protein SopE2
AV35_06705
hypothetical protein
AV35_06715
integrase
AV35_06720
exodeoxyribonuclease VIII
AV35_06735
lytic enzyme
Salmonella enterica subsp. enterica serovar Enteritidis str. EC20121542 genome−p.c.VAL54.69%−taxID1412557
AV35_06555
membrane protein
AV35_06580
hypothetical protein
AV35_06535
AV35_06540
AV35_06545
DNA polymerase III subunit theta
AV35_06570
integrase
AV35_06575
excisionase
Salmonella enterica subsp. enterica serovar Enteritidis str. EC20121542 genome−p.c.VAL98.02%−taxID1412557

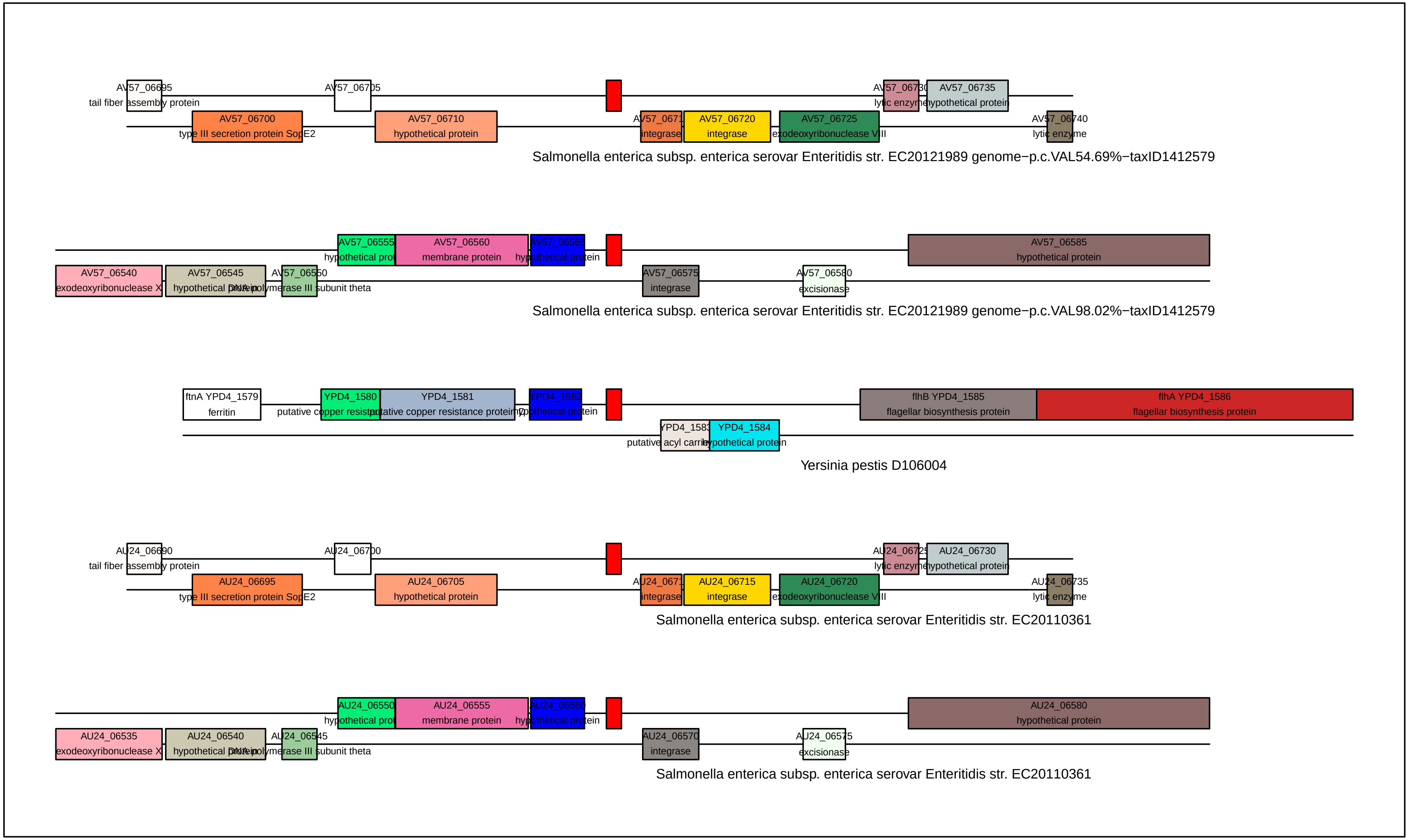

Salmonella enterica subsp. enterica serovar Enteritidis str. EC20121989 genome−p.c.VAL54.69%−taxID1412579
Salmonella enterica subsp. enterica serovar Enteritidis str. EC20121989 genome−p.c.VAL98.02%−taxID1412579
Yersinia pestis D106004
Salmonella enterica subsp. enterica serovar Enteritidis str. EC20110361
Salmonella enterica subsp. enterica serovar Enteritidis str. EC20110361

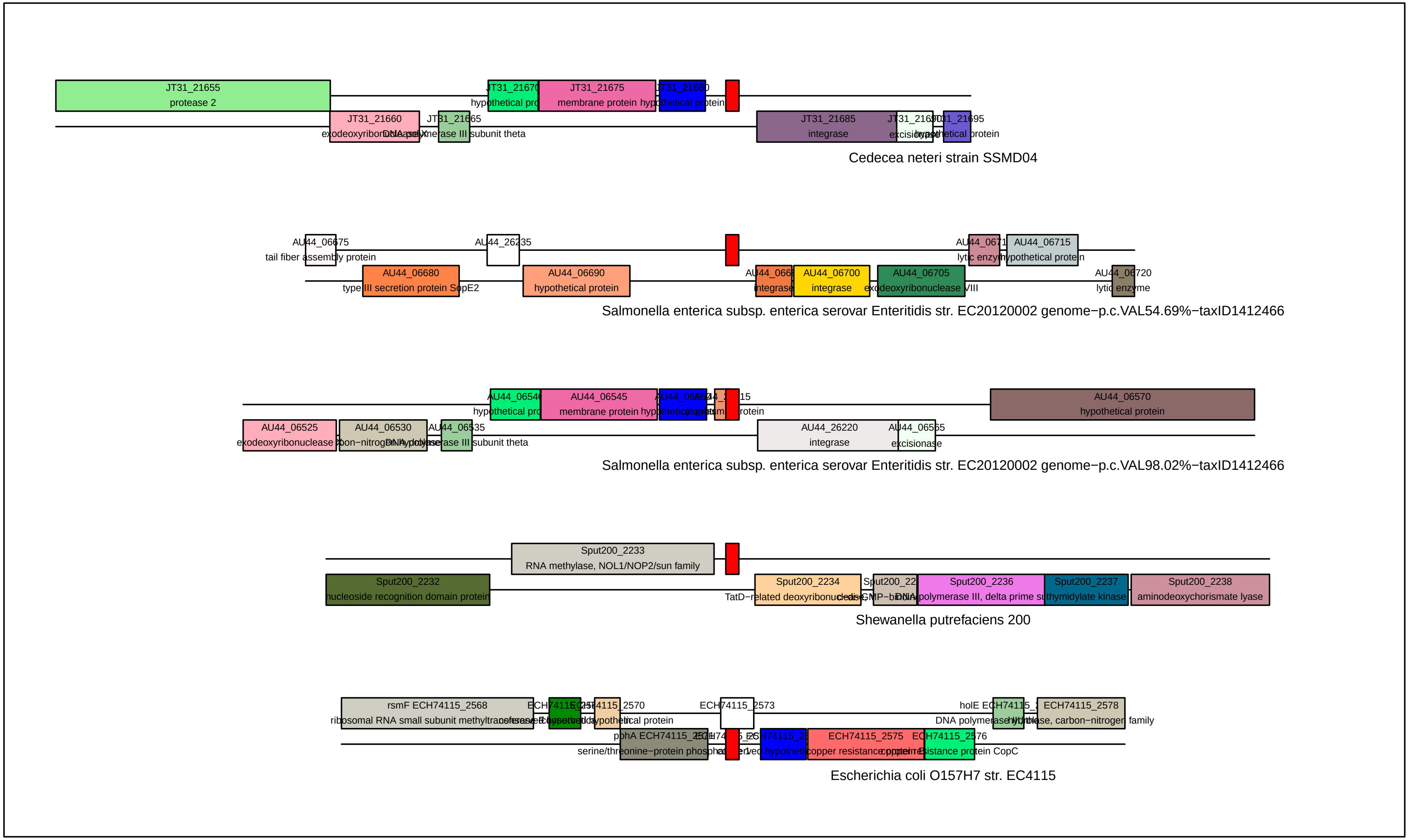

JT31_21655
protease 2
JT31_21660
JT31_21665
JT31_21675
membrane protein
JT31_21685
integrase
hypothetical protein
Cedecea neteri strain SSMD04
tail fiber assembly protein
AU44_06680
type III secretion protein SopE2
AU44_06690
hypothetical protein
AU44_06700
integrase
AU44_06705
exodeoxyribonuclease VIII
AU44_06715
AU44_06720
lytic enzyme
Salmonella enterica subsp. enterica serovar Enteritidis str. EC20120002 genome−p.c.VAL54.69%−taxID1412466
AU44_06525
exodeoxyribonuclease
AU44_06530
AU44_06545
membrane protein
AU44_26220
integrase
excisionase
AU44_06570
hypothetical protein
Salmonella enterica subsp. enterica serovar Enteritidis str. EC20120002 genome−p.c.VAL98.02%−taxID1412466
Sput200_2232
nucleoside recognition domain protein
Sput200_2233
RNA methylase, NOL1/NOP2/sun family
Sput200_2234
Sput200_2236
Sput200_2237
thymidylate kinase
Sput200_2238
aminodeoxychorismate lyase
Shewanella putrefaciens 200
rsmF ECH74115_2568
ECH74115_2573
ECH74115_2575
ECH74115_2578
Escherichia coli O157H7 str. EC4115

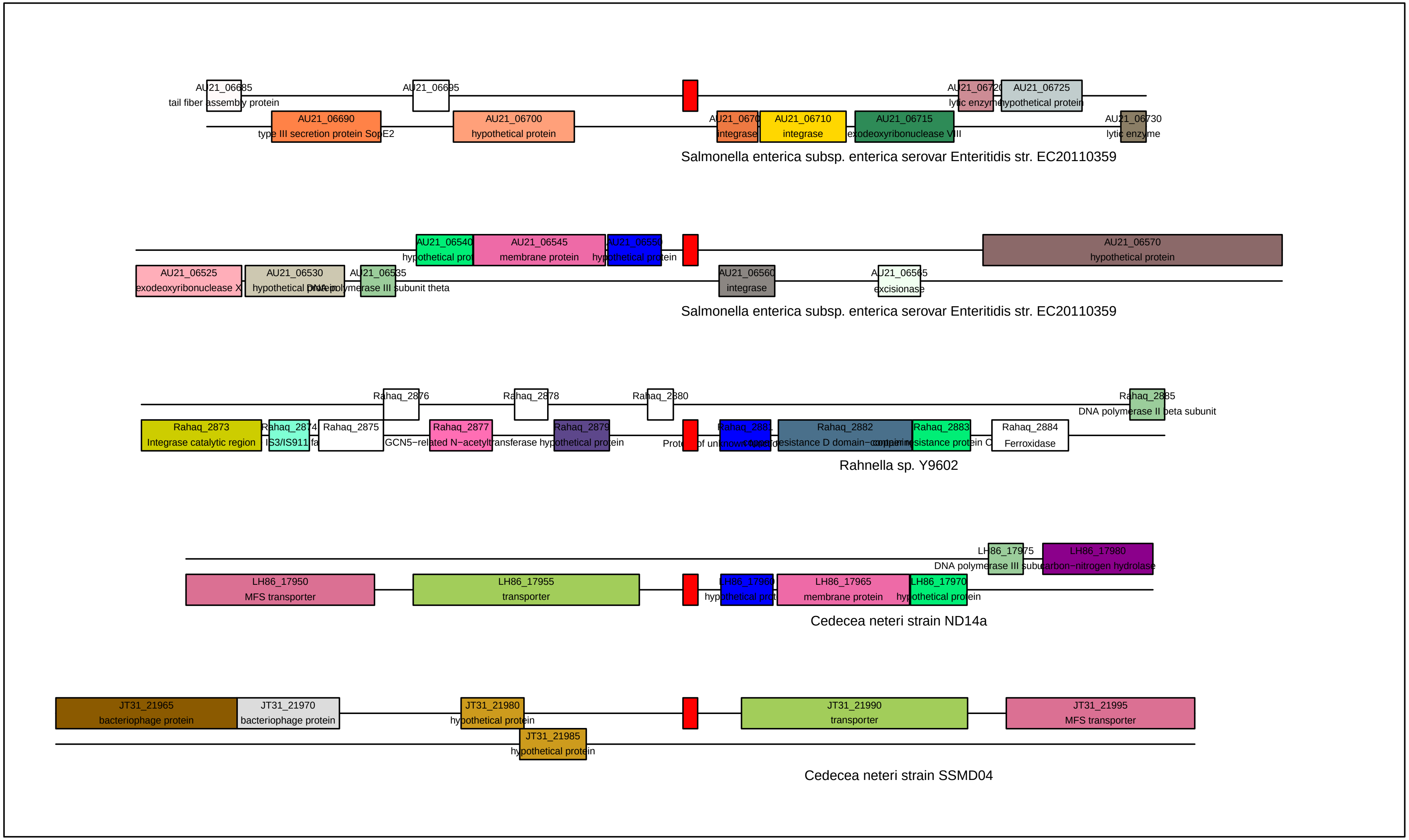

AU21_06685
tail fiber assembly protein
AU21_06695
AU21_06690
type III secretion protein SopE2
AU21_06700
hypothetical protein
AU21_06710
integrase
AU21_06715
exodeoxyribonuclease VIII
AU21_06725
hypothetical protein
AU21_06730
lytic enzyme
Salmonella enterica subsp. enterica serovar Enteritidis str. EC20110359
AU21_06545
membrane protein
AU21_06570
hypothetical protein
AU21_06525
exodeoxyribonuclease X
AU21_06530
AU21_06560
integrase
excisionase
Salmonella enterica subsp. enterica serovar Enteritidis str. EC20110359
Rahaq_2876
Rahaq_2878
Rahaq_2885
DNA polymerase II beta subunit
Rahaq_2873
Integrase catalytic region
Rahaq_2875
Rahaq_2877
Rahaq_2882
Rahaq_2883
Rahaq_2884
Ferroxidase
Rahnella sp. Y9602
LH86_17980
LH86_17950
MFS transporter
LH86_17955
transporter
LH86_17965
membrane protein
Cedecea neteri strain ND14a
JT31_21965
bacteriophage protein
JT31_21970
bacteriophage protein
JT31_21980
hypothetical protein
JT31_21990
transporter
JT31_21995
MFS transporter
JT31_21985
hypothetical protein
Cedecea neteri strain SSMD04

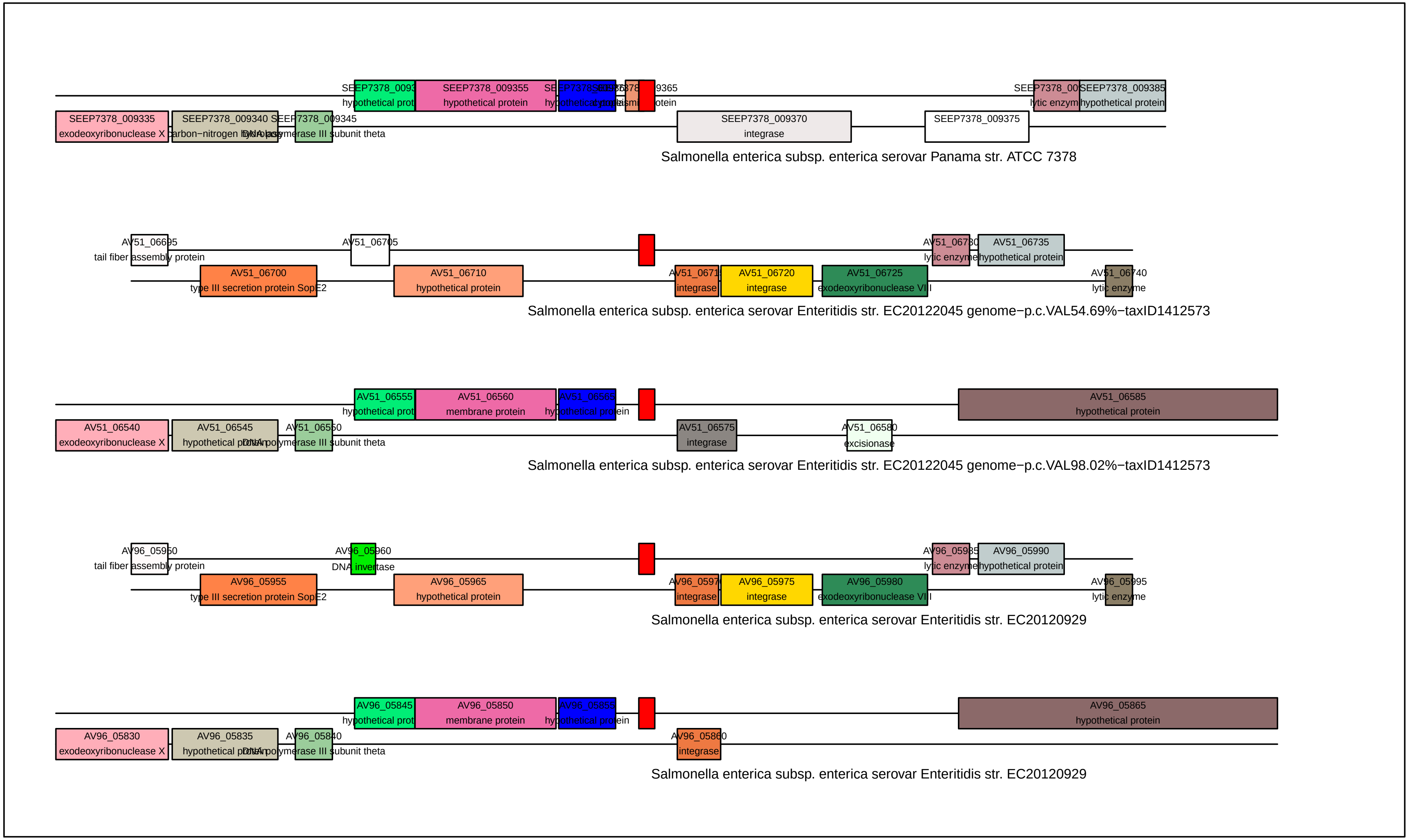
SEEP7378_009355
hypothetical protein
SEEP7378_009335
exodeoxyribonuclease X
SEEP7378_009340
SEEP7378_009345
DNA polymerase III subunit theta
SEEP7378_009370
integrase
SEEP7378_009375
SEEP7378_009385
hypothetical protein
lytic enzym
Salmonella enterica subsp. enterica serovar Panama str. ATCC 7378
AV51_06695
tail fiber assembly protein
AV51_06705
AV51_06735
hypothetical protein
lytic enzyme
AV51_06700
type III secretion protein SopE2
AV51_06710
hypothetical protein
integrase
AV51_06720
integrase
AV51_06725
exodeoxyribonuclease VIII
AV51_06740
lytic enzyme
Salmonella enterica subsp. enterica serovar Enteritidis str. EC20122045 genome−p.c.VAL54.69%−taxID1412573
AV51_06555
AV51_06560
membrane protein
AV51_06585
hypothetical protein
AV51_06540
exodeoxyribonuclease X
AV51_06545
AV51_06550
DNA polymerase III subunit theta
AV51_06575
integrase
AV51_06580
excisionase
Salmonella enterica subsp. enterica serovar Enteritidis str. EC20122045 genome−p.c.VAL98.02%−taxID1412573
AV96_05950
tail fiber assembly protein
AV96_05960
DNA invertase
AV96_05990
hypothetical protein
lytic enzyme
AV96_05955
type III secretion protein SopE2
AV96_05965
hypothetical protein
integrase
AV96_05975
integrase
AV96_05980
exodeoxyribonuclease VIII
AV96_05995
lytic enzyme
Salmonella enterica subsp. enterica serovar Enteritidis str. EC20120929
AV96_05845
AV96_05850
membrane protein
AV96_05855
AV96_05865
hypothetical protein
AV96_05830
exodeoxyribonuclease X
AV96_05835
AV96_05840
DNA polymerase III subunit theta
AV96_05860
integrase
Salmonella enterica subsp. enterica serovar Enteritidis str. EC20120929

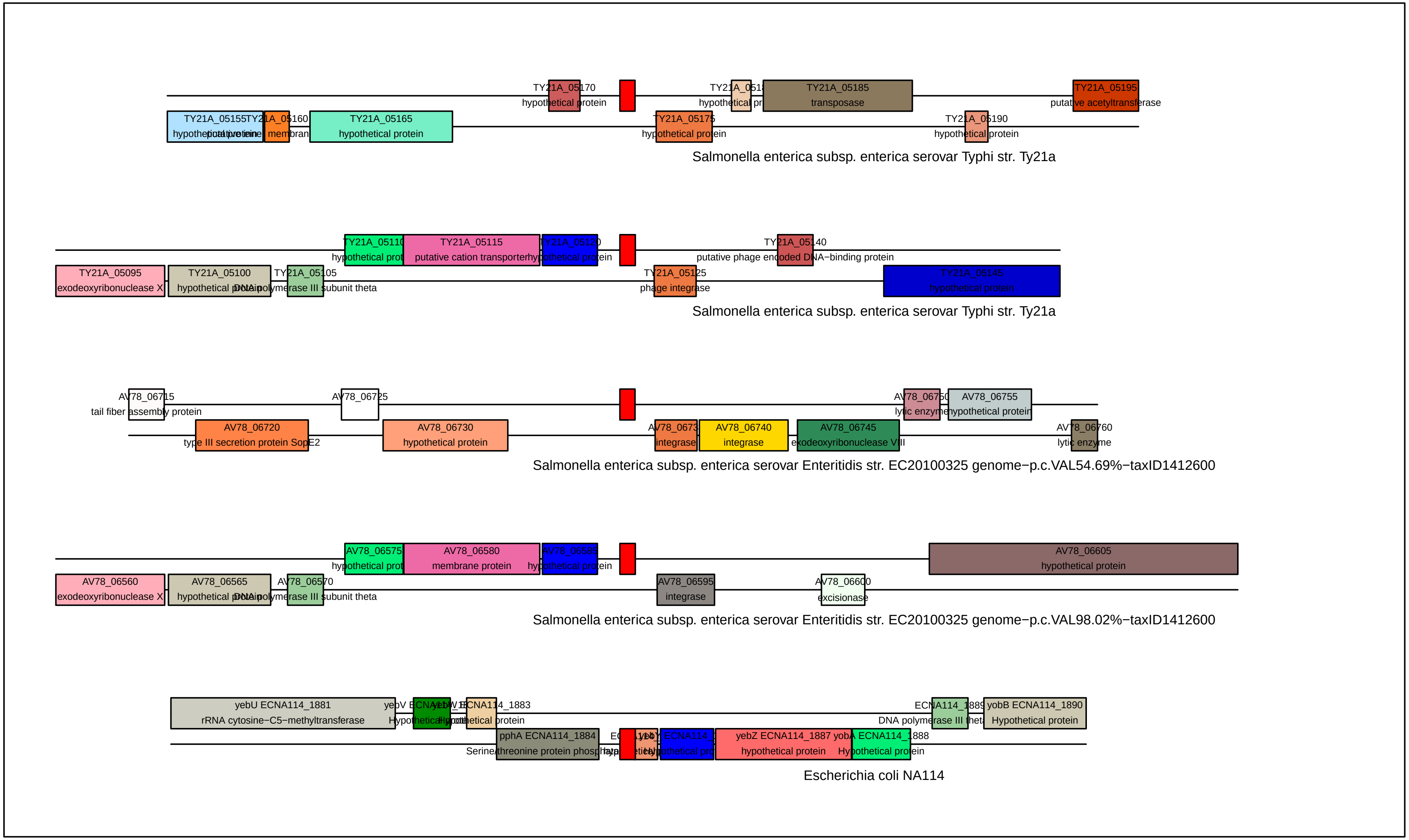

TY21A_05170
hypothetical protein
TY21A_05185
transposase
TY21A_05195
putative acetyltransferase
TY21A_05165
hypothetical protein
TY21A_05175
hypothetical protein
TY21A_05190
hypothetical protein
Salmonella enterica subsp. enterica serovar Typhi str. Ty21a
TY21A_05115
putative cation transporter
TY21A_05140
putative phage encoded DNA−binding protein
TY21A_05095
exodeoxyribonuclease X
TY21A_05100
TY21A_05105
TY21A_05125
phage integrase
TY21A_05145
hypothetical protein
Salmonella enterica subsp. enterica serovar Typhi str. Ty21a
AV78_06715
tail fiber assembly protein
AV78_06725
AV78_06755
hypothetical protein
AV78_06720
type III secretion protein SopE2
AV78_06730
hypothetical protein
AV78_06740
integrase
AV78_06745
exodeoxyribonuclease VIII
AV78_06760
lytic enzyme
Salmonella enterica subsp. enterica serovar Enteritidis str. EC20100325 genome−p.c.VAL54.69%−taxID1412600
AV78_06575
AV78_06580
membrane protein
AV78_06605
hypothetical protein
AV78_06560
exodeoxyribonuclease X
AV78_06565
AV78_06570
AV78_06595
integrase
AV78_06600
excisionase
Salmonella enterica subsp. enterica serovar Enteritidis str. EC20100325 genome−p.c.VAL98.02%−taxID1412600
yebU ECNA114_1881
rRNA cytosine−C5−methyltransferase
yobB ECNA114_1890
Hypothetical protein
pphA ECNA114_1884
yebZ ECNA114_1887
hypothetical protein
Escherichia coli NA114

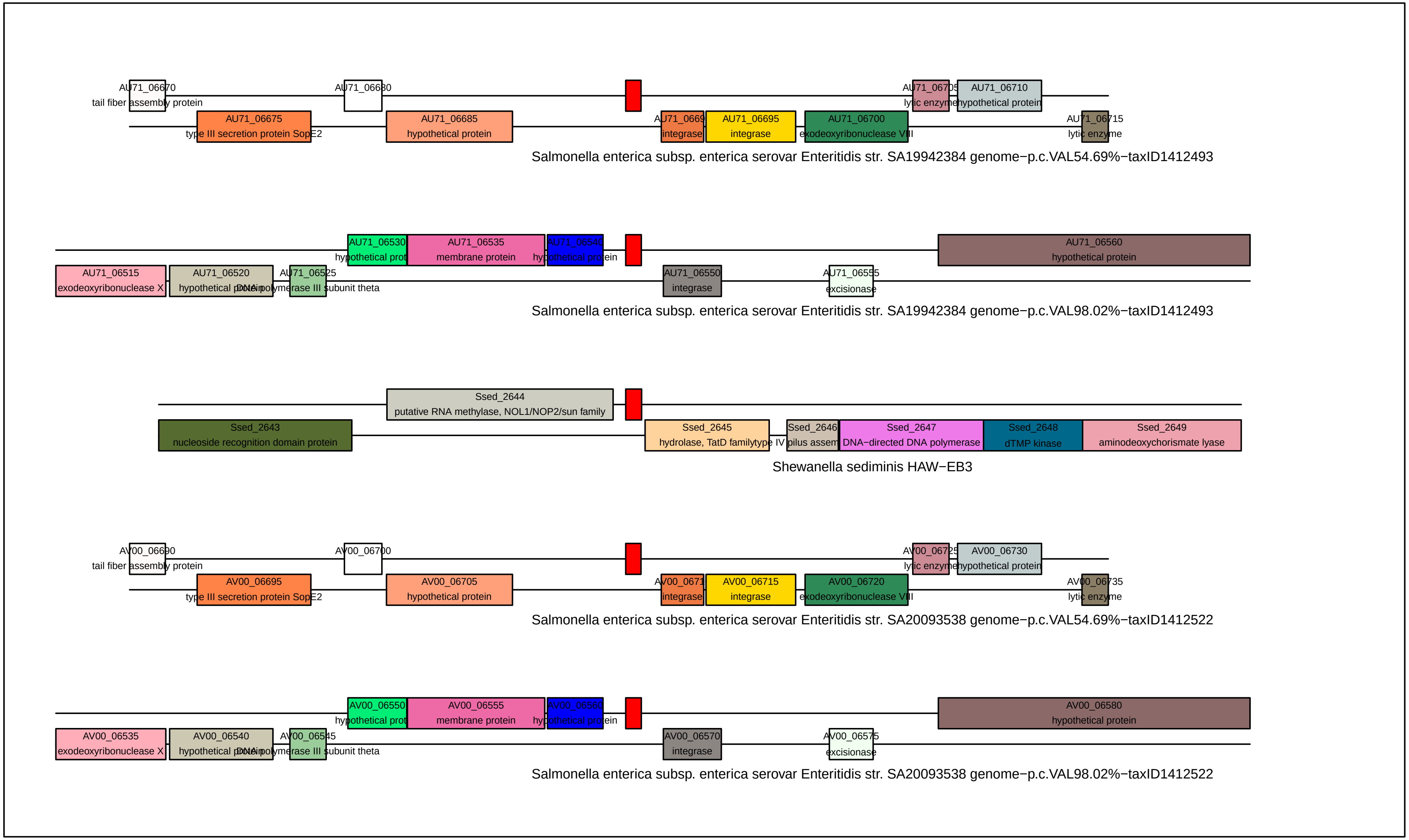
AU71_06670
tail fiber assembly protein
AU71_06680
AU71_06705
lytic enzyme
AU71_06710
hypothetical protein
AU71_06675
type III secretion protein SopE2
AU71_06685
hypothetical protein
AU71_0669
integrase
AU71_06695
integrase
AU71_06700
exodeoxyribonuclease VIII
AU71_06715
lytic enzyme
Salmonella enterica subsp. enterica serovar Enteritidis str. SA19942384 genome−p.c.VAL54.69%−taxID1412493
AU71_06530
hypothetical prot
AU71_06535
membrane protein
AU71_06540
hypothetical protein
AU71_06560
hypothetical protein
AU71_06515
exodeoxyribonuclease X
AU71_06520
hypothetical protein
AU71_06525
DNA polymerase III subunit theta
AU71_06550
integrase
AU71_06555
excisionase
Salmonella enterica subsp. enterica serovar Enteritidis str. SA19942384 genome−p.c.VAL98.02%−taxID1412493
Ssed_2644
putative RNA methylase, NOL1/NOP2/sun family
Ssed_2643
nucleoside recognition domain protein
Ssed_2645
hydrolase, TatD family
type IV
Ssed_2646
pilus assem
Ssed_2647
DNA−directed DNA polymerase
Ssed_2648
dTMP kinase
Ssed_2649
aminodeoxychorismate lyase
Shewanella sediminis HAW−EB3
AV00_06690
tail fiber assembly protein
AV00_06700
AV00_06725
lytic enzyme
AV00_06730
hypothetical protein
AV00_06695
type III secretion protein SopE2
AV00_06705
hypothetical protein
AV00_0671
integrase
AV00_06715
integrase
AV00_06720
exodeoxyribonuclease VIII
AV00_06735
lytic enzyme
Salmonella enterica subsp. enterica serovar Enteritidis str. SA20093538 genome−p.c.VAL54.69%−taxID1412522
AV00_06550
hypothetical prot
AV00_06555
membrane protein
AV00_06560
hypothetical protein
AV00_06580
hypothetical protein
AV00_06535
exodeoxyribonuclease X
AV00_06540
hypothetical protein
AV00_06545
DNA polymerase III subunit theta
AV00_06570
integrase
AV00_06575
excisionase
Salmonella enterica subsp. enterica serovar Enteritidis str. SA20093538 genome−p.c.VAL98.02%−taxID1412522

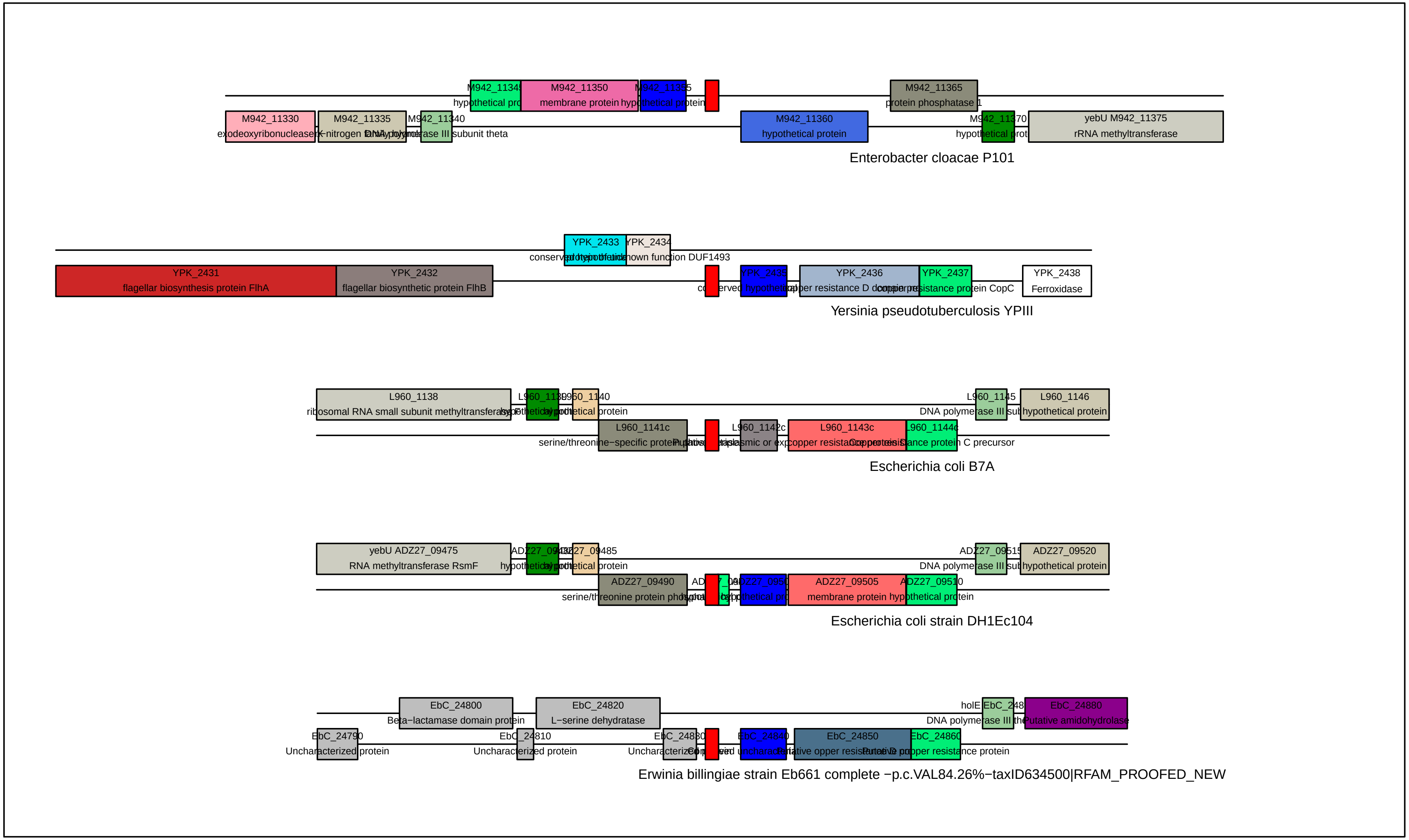

M942_11350
membrane protein
M942_11365
protein phosphatase 1
M942_11330
M942_11335
M942_11340
subunit theta
M942_11360
hypothetical protein
yebU M942_11375
rRNA methyltransferase
Enterobacter cloacae P101
YPK_2433
YPK_2434
own function DUF1493
YPK_2431
flagellar biosynthesis protein FlhA
YPK_2432
flagellar biosynthetic protein FlhB
YPK_2436
YPK_2437
istance protein CopC
YPK_2438
Ferroxidase
Yersinia pseudotuberculosis YPIII
L960_1138
ribosomal RNA small subunit methyltransfera
L960_1145
L960_1146
hypothetical protein
L960_1141c
L960_1143c
ance protein C precursor
Escherichia coli B7A
yebU ADZ27_09475
RNA methyltransferase RsmF
ADZ27_09485
ADZ27_09520
hypothetical protein
ADZ27_09490
ADZ27_09505
membrane protein
ADZ27_09510
Escherichia coli strain DH1Ec104
EbC_24800
Beta−lactamase domain protein
EbC_24820
L−serine dehydratase
EbC_24880
Putative amidohydrolase
EbC_24790
Uncharacterized protein
EbC_24810
Uncharacterized protein
EbC_24850
EbC_24860
pper resistance protein
Erwinia billingiae strain Eb661 complete −p.c.VAL84.26%−taxID634500|RFAM_PROOFED_NEW

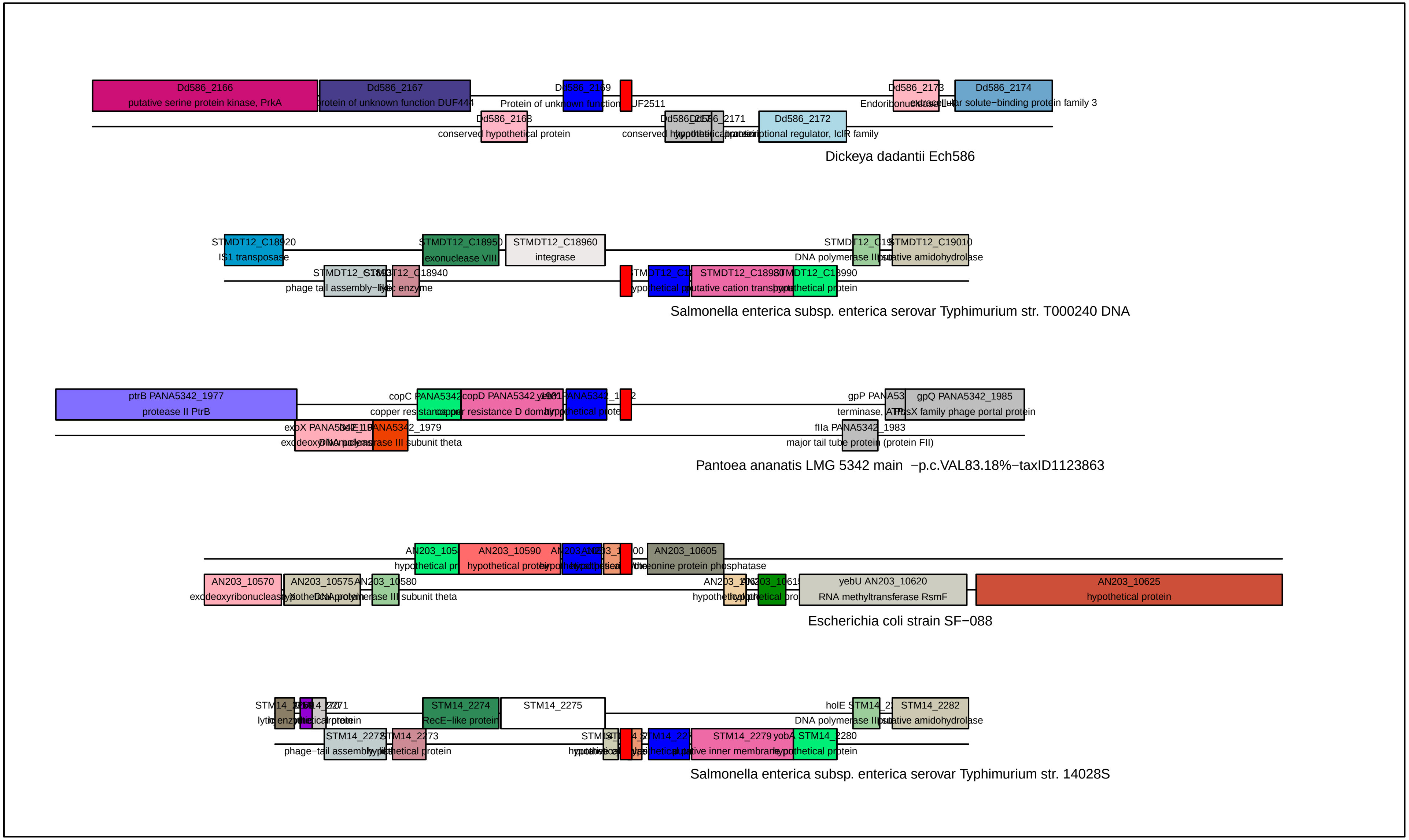

Dd586_2166
putative serine protein kinase, PrkA
Dd586_2167
Dd586_2168
conserved hypothetical protein
Dd586_2171
Dd586_2172
Dd586_2174
Dickeya dadantii Ech586
STMDT12_C18920
IS1 transposase
STMDT12_C18960
integrase
exonuclease VIII
STMDT12_C19010
putative amidohydrolase
Salmonella enterica subsp. enterica serovar Typhimurium str. T000240 DNA
ptrB PANA5342_1977
protease II PtrB
gpQ PANA5342_1985
fIIa PANA5342_1983
major tail tube protein (protein FII)
Pantoea ananatis LMG 5342 main −p.c.VAL83.18%−taxID1123863
AN203_10590
AN203_10605
AN203_10570
AN203_10580
yebU AN203_10620
RNA methyltransferase RsmF
AN203_10625
hypothetical protein
Escherichia coli strain SF−088
STM14_2274
RecE−like protein
STM14_2275
STM14_2279
STM14_2280
STM14_2282
putative amidohydrolase
Salmonella enterica subsp. enterica serovar Typhimurium str. 14028S

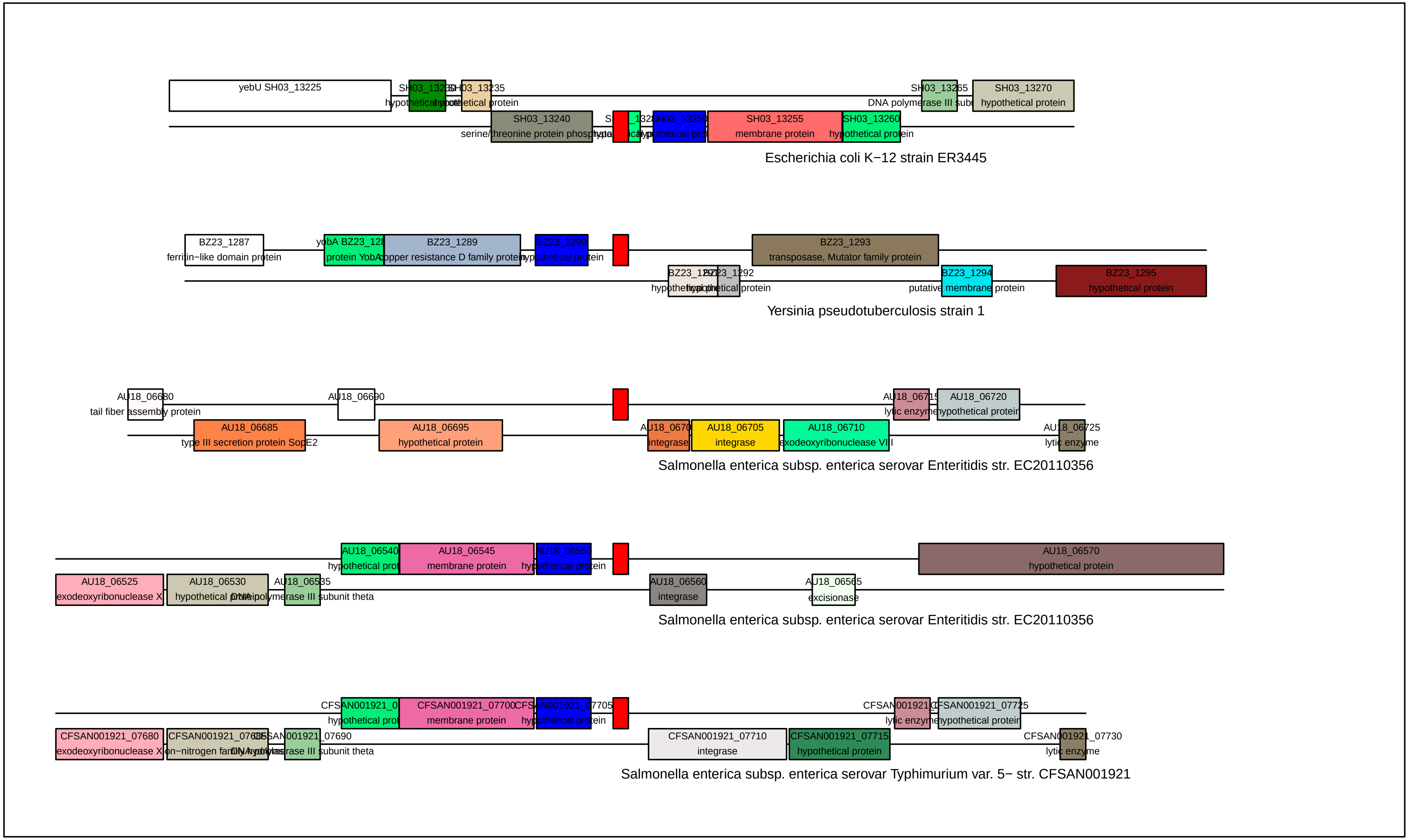

yebU SH03_13225
SH03_13235
SH03_13265
DNA polymerase III
SH03_13270
hypothetical protein
SH03_13240
serine/threonine protein phosph
SH03_13255
membrane protein
SH03_13260
hypothetical protein
Escherichia coli K−12 strain ER3445
BZ23_1287
ferritin−like domain protein
BZ23_1289
copper resistance D family protein
BZ23_1290
hypothetical protein
BZ23_1293
transposase, Mutator family protein
BZ23_1292
BZ23_1294
putative membrane protein
BZ23_1295
hypothetical protein
Yersinia pseudotuberculosis strain 1
AU18_06680
tail fiber assembly protein
AU18_06690
AU18_06720
hypothetical protein
lytic enzyme
AU18_06685
type III secretion protein SopE2
AU18_06695
hypothetical protein
integrase
AU18_06705
integrase
AU18_06710
exodeoxyribonuclease VIII
AU18_06725
lytic enzyme
Salmonella enterica subsp. enterica serovar Enteritidis str. EC20110356
AU18_06540
AU18_06545
membrane protein
AU18_06570
hypothetical protein
AU18_06525
AU18_06530
AU18_06535
subunit theta
AU18_06560
integrase
AU18_06565
excisionase
Salmonella enterica subsp. enterica serovar Enteritidis str. EC20110356
CFSAN001921_07700
membrane protein
CFSAN001921_07725
hypothetical protein
lytic enzyme
CFSAN001921_07680
CFSAN001921_07690
subunit theta
CFSAN001921_07710
integrase
CFSAN001921_07715
hypothetical protein
CFSAN001921_07730
lytic enzyme
Salmonella enterica subsp. enterica serovar Typhimurium var. 5− str. CFSAN001921

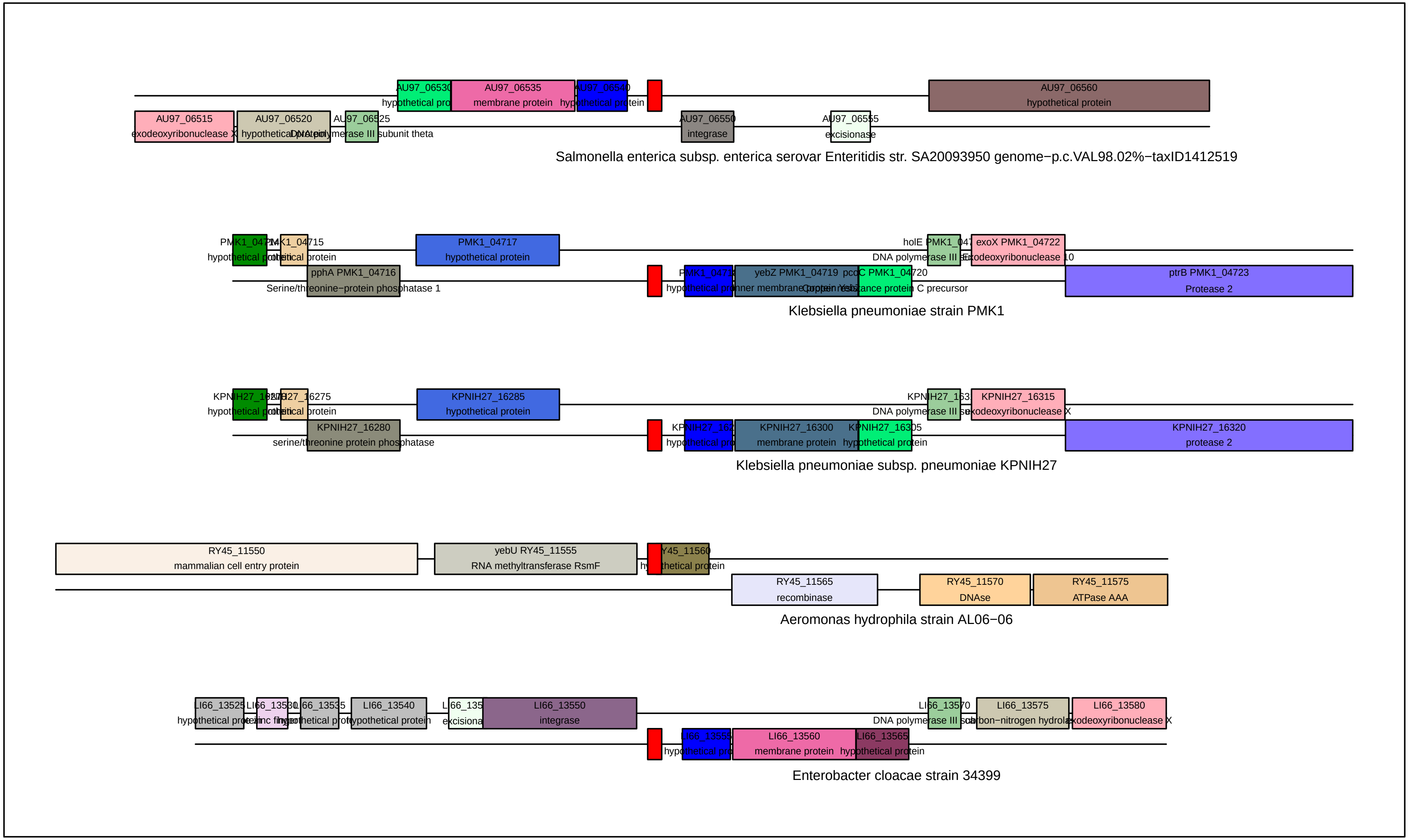

AU97_06535
membrane protein
AU97_06560
hypothetical protein
AU97_06515
AU97_06520
AU97_06550
integrase
excisionase
Salmonella enterica subsp. enterica serovar Enteritidis str. SA20093950 genome−p.c.VAL98.02%−taxID1412519
PMK1_04717
hypothetical protein
exoX PMK1_04722
pphA PMK1_04716
Serine/threonine−protein phosphatase 1
yebZ PMK1_04719
ptrB PMK1_04723
Protease 2
Klebsiella pneumoniae strain PMK1
KPNIH27_16285
hypothetical protein
KPNIH27_16315
KPNIH27_16280
serine/threonine protein phosphatase
KPNIH27_16300
membrane protein
KPNIH27_16320
protease 2
Klebsiella pneumoniae subsp. pneumoniae KPNIH27
RY45_11550
mammalian cell entry protein
yebU RY45_11555
RNA methyltransferase RsmF
RY45_11565
recombinase
RY45_11570
DNAse
RY45_11575
ATPase AAA
Aeromonas hydrophila strain AL06−06
LI66_13540
hypothetical protein
LI66_13550
integrase
LI66_13575
LI66_13580
LI66_13560
membrane protein
Enterobacter cloacae strain 34399

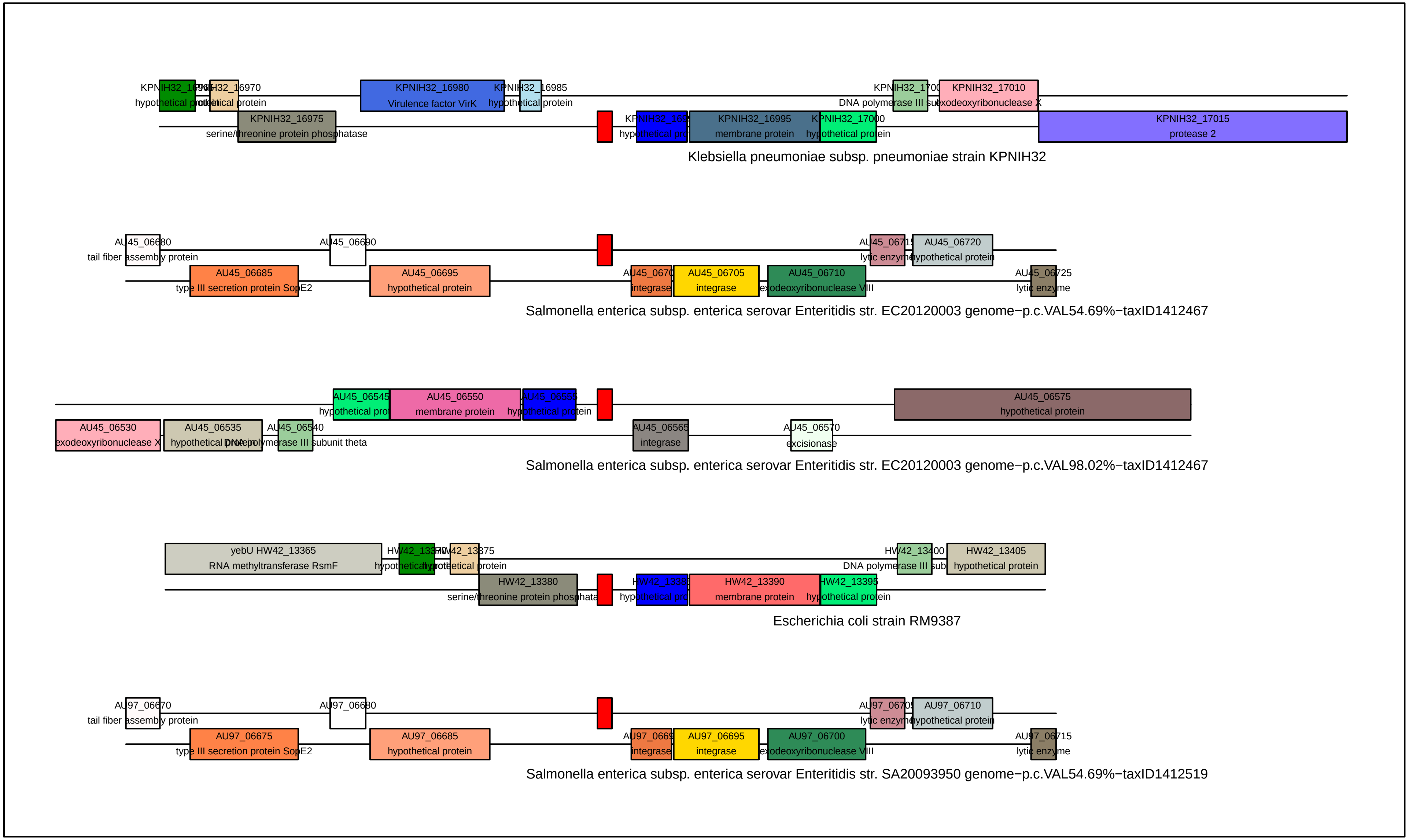

KPNIH32_16970
hypothetical protein
KPNIH32_16975
serine/threonine protein phosphatase
KPNIH32_16980
Virulence factor VirK
KPNIH32_16985
hypothetical protein
KPNIH32_16995
membrane protein
KPNIH32_17000
hypothetical protein
DNA polymerase III
KPNIH32_17010
exodeoxyribonuclease X
KPNIH32_17015
protease 2
Klebsiella pneumoniae subsp. pneumoniae strain KPNIH32
AU45_06680
tail fiber assembly protein
AU45_06690
AU45_06685
type III secretion protein SopE2
AU45_06695
hypothetical protein
integrase
AU45_06705
integrase
AU45_06710
exodeoxyribonuclease VIII
lytic enzyme
AU45_06720
hypothetical protein
AU45_06725
lytic enzyme
Salmonella enterica subsp. enterica serovar Enteritidis str. EC20120003 genome−p.c.VAL54.69%−taxID1412467
AU45_06545
AU45_06550
membrane protein
AU45_06575
hypothetical protein
AU45_06530
exodeoxyribonuclease X
AU45_06535
AU45_06540
subunit theta
AU45_06565
integrase
AU45_06570
excisionase
Salmonella enterica subsp. enterica serovar Enteritidis str. EC20120003 genome−p.c.VAL98.02%−taxID1412467
yebU HW42_13365
RNA methyltransferase RsmF
HW42_13375
HW42_13400
HW42_13405
hypothetical protein
HW42_13380
HW42_13390
membrane protein
hypothetical protein
Escherichia coli strain RM9387
AU97_06670
tail fiber assembly protein
AU97_06680
AU97_06675
type III secretion protein SopE2
AU97_06685
hypothetical protein
integrase
AU97_06695
integrase
AU97_06700
exodeoxyribonuclease VIII
lytic enzyme
AU97_06710
hypothetical protein
AU97_06715
lytic enzyme
Salmonella enterica subsp. enterica serovar Enteritidis str. SA20093950 genome−p.c.VAL54.69%−taxID1412519

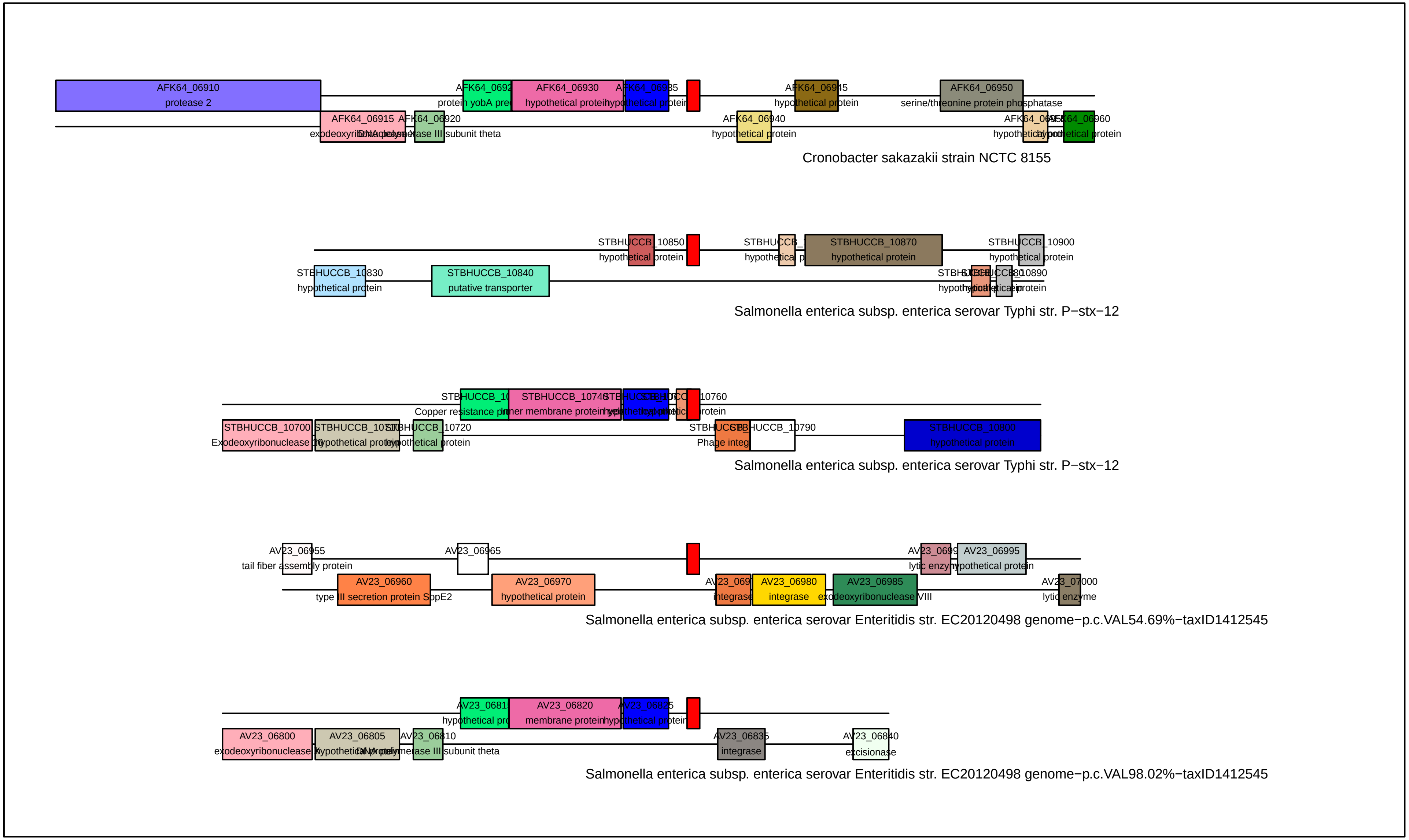

AFK64_06910
protease 2
AFK64_06930
hypothetical protein
AFK64_06945
hypothetical protein
AFK64_06950
serine/threonine protein phosphatase
AFK64_06915
AFK64_06920
AFK64_06940
hypothetical protein
AFK64_06960
Cronobacter sakazakii strain NCTC 8155
STBHUCCB_10850
hypothetical protein
STBHUCCB_10870
hypothetical protein
STBHUCCB_10900
hypothetical protein
STBHUCCB_10830
hypothetical protein
STBHUCCB_10840
putative transporter
Salmonella enterica subsp. enterica serovar Typhi str. P−stx−12
Copper resistance
inner membrane protein
STBHUCCB_10700
Exodeoxyribonuclease
STBHUCCB_10720
hypothetical protein
STBHUCCB_10790
STBHUCCB_10800
hypothetical protein
Salmonella enterica subsp. enterica serovar Typhi str. P−stx−12
AV23_06955
tail fiber assembly protein
AV23_06965
AV23_06995
hypothetical protein
AV23_06960
type III secretion protein SopE2
AV23_06970
hypothetical protein
AV23_06980
integrase
AV23_06985
exodeoxyribonuclease VIII
AV23_07000
lytic enzyme
Salmonella enterica subsp. enterica serovar Enteritidis str. EC20120498 genome−p.c.VAL54.69%−taxID1412545
AV23_06820
membrane protein
AV23_06800
exodeoxyribonuclease
AV23_06805
AV23_06810
subunit theta
AV23_06835
integrase
AV23_06840
excisionase
Salmonella enterica subsp. enterica serovar Enteritidis str. EC20120498 genome−p.c.VAL98.02%−taxID1412545

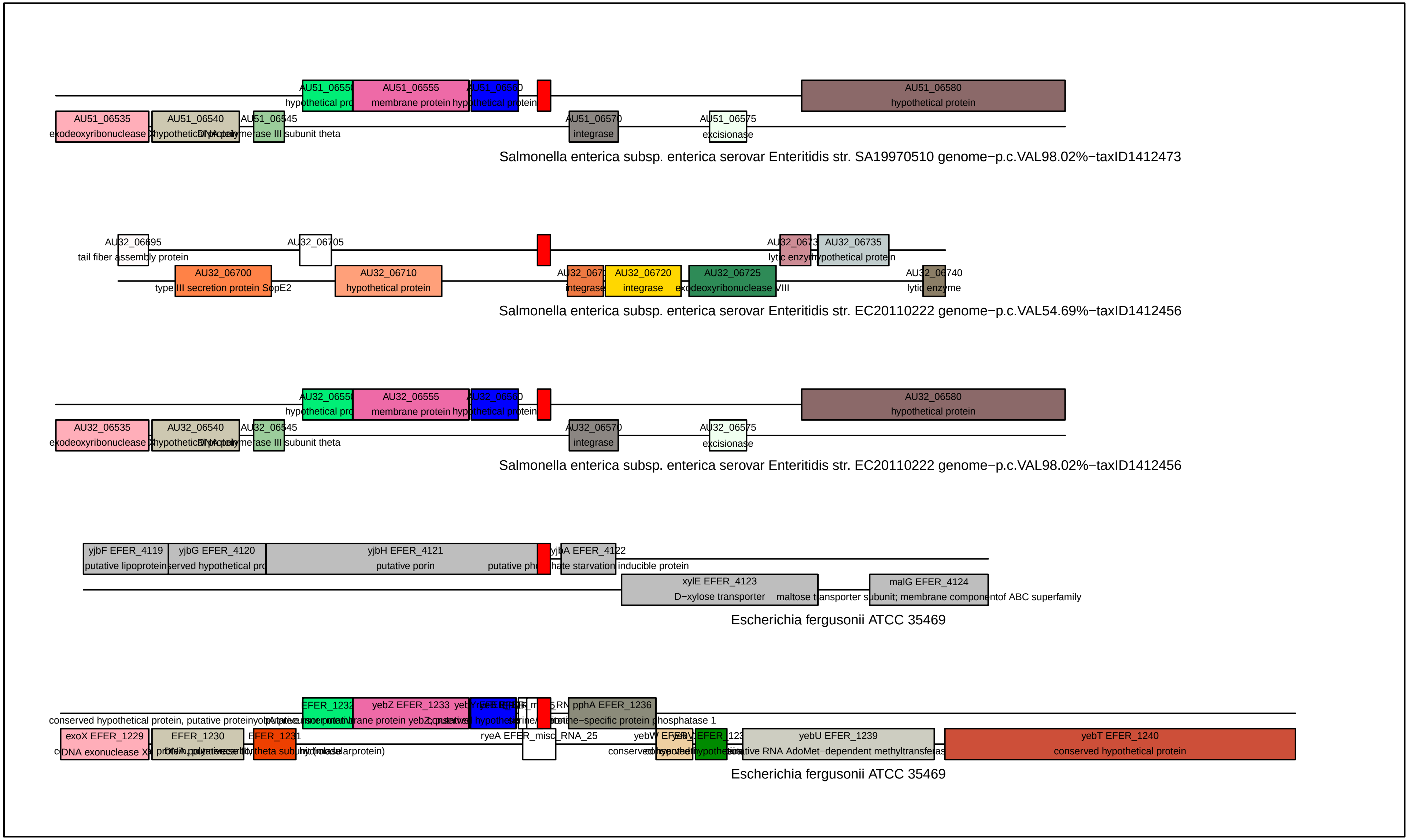
AU51_06580
hypothetical protein
AU51_06555
membrane protein
AU51_06535
exodeoxyribonuclease
AU51_06540
AU51_06545
subunit theta
AU51_06570
integrase
AU51_06575
excisionase
Salmonella enterica subsp. enterica serovar Enteritidis str. SA19970510 genome−p.c.VAL98.02%−taxID1412473
AU32_06695
tail fiber assembly protein
AU32_06705
AU32_06735
AU32_06700
type III secretion protein SopE2
AU32_06710
hypothetical protein
AU32_06720
integrase
AU32_06725
exodeoxyribonuclease VIII
AU32_06740
lytic enzyme
Salmonella enterica subsp. enterica serovar Enteritidis str. EC20110222 genome−p.c.VAL54.69%−taxID1412456
AU32_06580
hypothetical protein
AU32_06555
membrane protein
AU32_06535
exodeoxyribonuclease
AU32_06540
AU32_06545
subunit theta
AU32_06570
integrase
AU32_06575
excisionase
Salmonella enterica subsp. enterica serovar Enteritidis str. EC20110222 genome−p.c.VAL98.02%−taxID1412456
yjbF EFER_4119
putative lipoprotein
yjbG EFER_4120
yjbH EFER_4121
putative porin
yjbA EFER_4122
inducible protein
xylE EFER_4123
D−xylose transporter
malG EFER_4124
maltose transporter
tof ABC superfamily
Escherichia fergusonii ATCC 35469
yebZ EFER_1233
pphA EFER_1236
conserved hypothetical protein, putative protein
phosphatase 1
exoX EFER_1229
EFER_1230
ryeA EFER_misc_RNA_25
yebU EFER_1239
yebT EFER_1240
conserved hypothetical protein
Escherichia fergusonii ATCC 35469

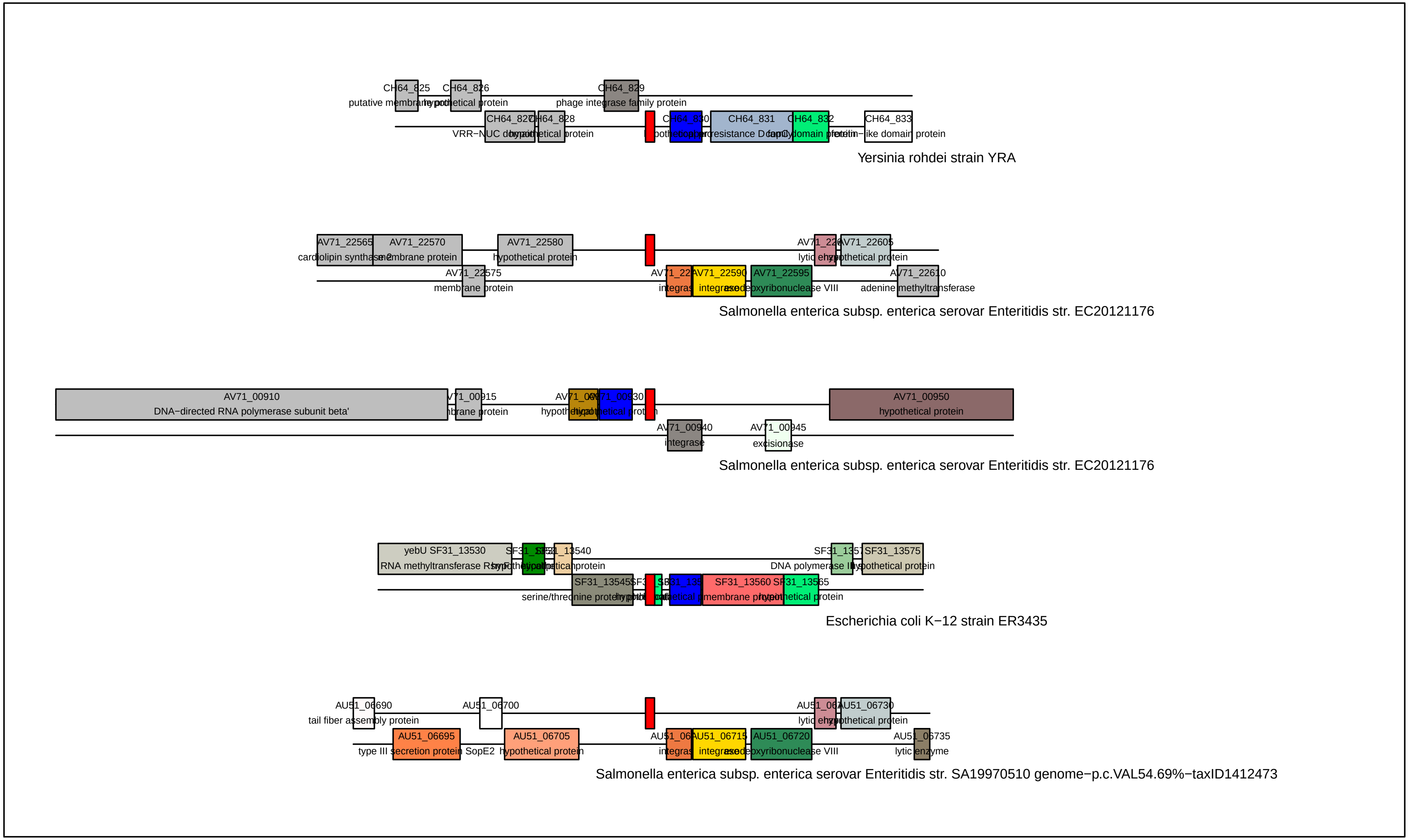
CH64_825
CH64_826
CH64_829
phage integrase family protein
CH64_831
CH64_832
CH64_833
Yersinia rohdei strain YRA
AV71_22565
AV71_22570
AV71_22580
hypothetical protein
AV71_22575
membrane protein
AV71_22590
AV71_22595
AV71_22605
AV71_22610
adenine methyltransferase
Salmonella enterica subsp. enterica serovar Enteritidis str. EC20121176
AV71_00910
DNA−directed RNA polymerase subunit beta'
AV71_00950
hypothetical protein
AV71_00940
integrase
AV71_00945
excisionase
Salmonella enterica subsp. enterica serovar Enteritidis str. EC20121176
yebU SF31_13530
SF31_13540
SF31_13575
SF31_13545
SF31_13560
SF31_13565
Escherichia coli K−12 strain ER3435
AU51_06690
AU51_06700
AU51_06730
AU51_06695
AU51_06705
AU51_06715
AU51_06720
AU51_06735
type III secretion protein SopE2
hypothetical protein
Salmonella enterica subsp. enterica serovar Enteritidis str. SA19970510 genome−p.c.VAL54.69%−taxID1412473

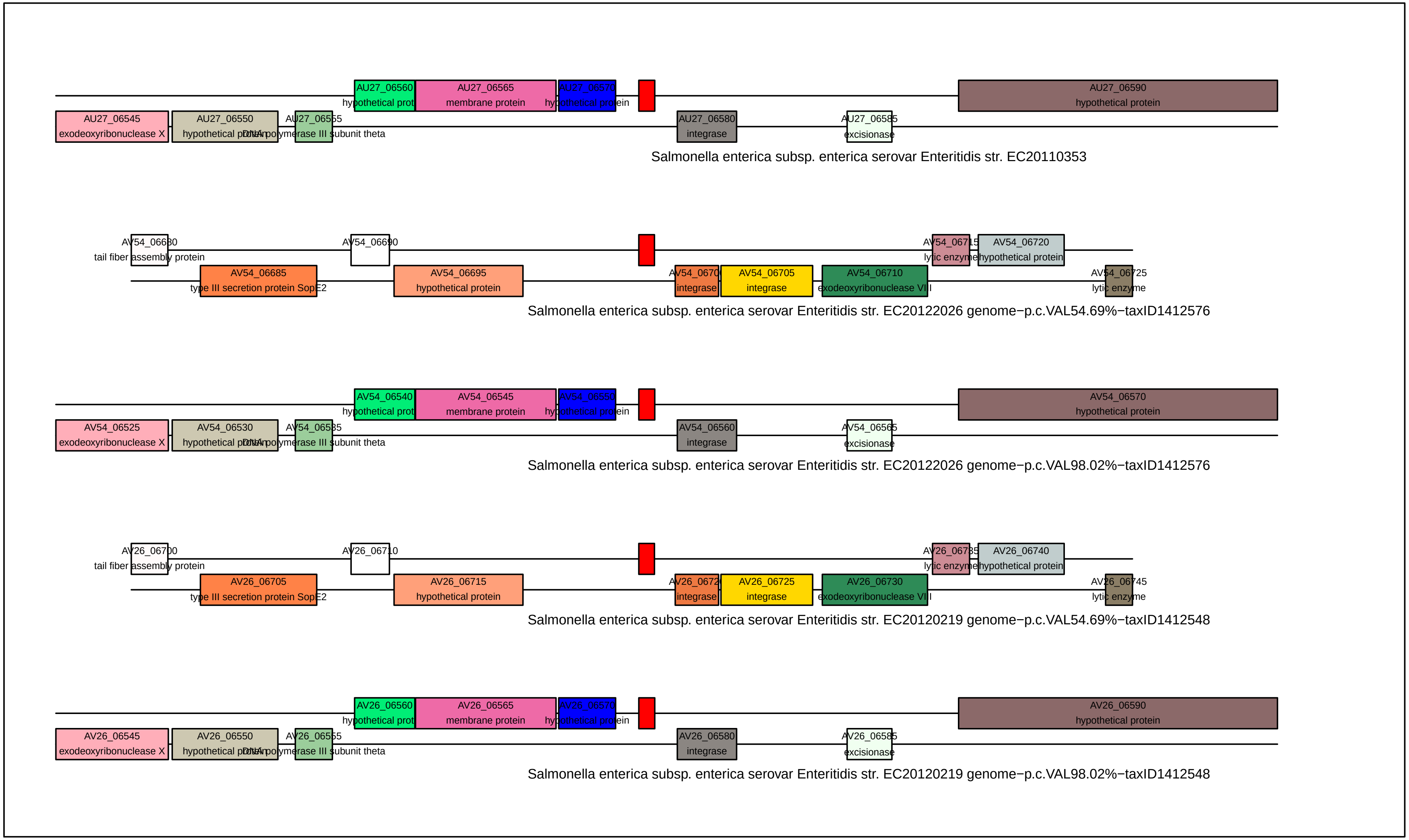
AU27_06560
hypothetical prot
AU27_06565
membrane protein
AU27_06570
hypothetical protein
AU27_06590
hypothetical protein
AU27_06545
exodeoxyribonuclease X
AU27_06550
hypothetical protein
AU27_06555
DNA polymerase III subunit theta
AU27_06580
integrase
AU27_06585
excisionase
Salmonella enterica subsp. enterica serovar Enteritidis str. EC20110353
AV54_06680
tail fiber assembly protein
AV54_06690
AV54_06715
lytic enzyme
AV54_06720
hypothetical protein
AV54_06685
type III secretion protein SopE2
AV54_06695
hypothetical protein
AV54_06700
integrase
AV54_06705
integrase
AV54_06710
exodeoxyribonuclease VIII
AV54_06725
lytic enzyme
Salmonella enterica subsp. enterica serovar Enteritidis str. EC20122026 genome−p.c.VAL54.69%−taxID1412576
AV54_06540
hypothetical prot
AV54_06545
membrane protein
AV54_06550
hypothetical protein
AV54_06570
hypothetical protein
AV54_06525
exodeoxyribonuclease X
AV54_06530
hypothetical protein
AV54_06535
DNA polymerase III subunit theta
AV54_06560
integrase
AV54_06565
excisionase
Salmonella enterica subsp. enterica serovar Enteritidis str. EC20122026 genome−p.c.VAL98.02%−taxID1412576
AV26_06700
tail fiber assembly protein
AV26_06710
AV26_06735
lytic enzyme
AV26_06740
hypothetical protein
AV26_06705
type III secretion protein SopE2
AV26_06715
hypothetical protein
AV26_06720
integrase
AV26_06725
integrase
AV26_06730
exodeoxyribonuclease VIII
AV26_06745
lytic enzyme
Salmonella enterica subsp. enterica serovar Enteritidis str. EC20120219 genome−p.c.VAL54.69%−taxID1412548
AV26_06560
hypothetical prot
AV26_06565
membrane protein
AV26_06570
hypothetical protein
AV26_06590
hypothetical protein
AV26_06545
exodeoxyribonuclease X
AV26_06550
hypothetical protein
AV26_06555
DNA polymerase III subunit theta
AV26_06580
integrase
AV26_06585
excisionase
Salmonella enterica subsp. enterica serovar Enteritidis str. EC20120219 genome−p.c.VAL98.02%−taxID1412548

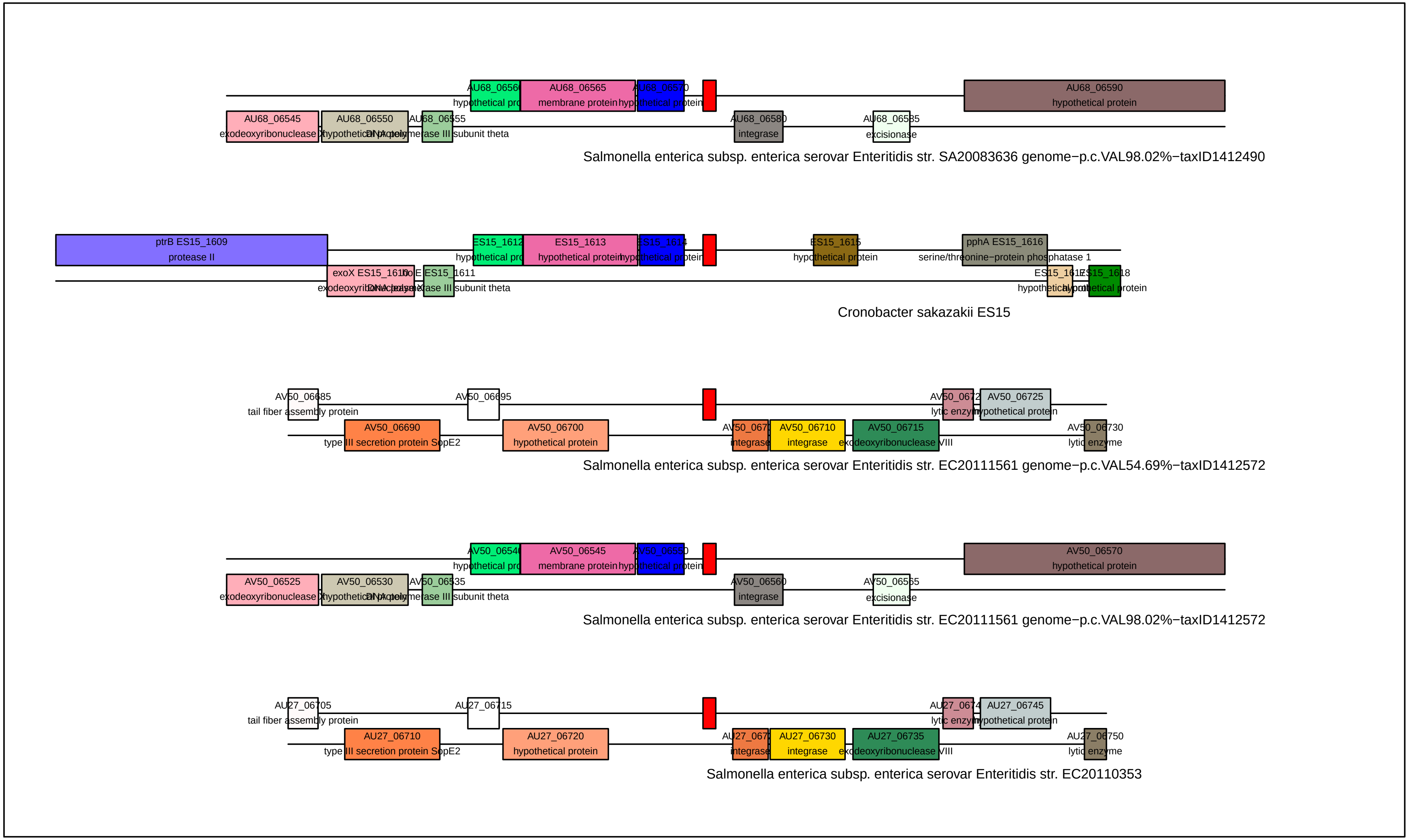

AU68_06565
membrane protein
AU68_06590
hypothetical protein
AU68_06545
AU68_06550
AU68_06580
integrase
excisionase
subunit theta
Salmonella enterica subsp. enterica serovar Enteritidis str. SA20083636 genome−p.c.VAL98.02%−taxID1412490
ptrB ES15_1609
protease II
ES15_1613
hypothetical protein
ES15_1615
pphA ES15_1616
serine/threonine−protein phosphatase 1
subunit theta
Cronobacter sakazakii ES15
AV50_06685
tail fiber assembly protein
AV50_06695
AV50_06725
AV50_06690
type III secretion protein SopE2
AV50_06700
hypothetical protein
AV50_06710
integrase
AV50_06715
exodeoxyribonuclease VIII
AV50_06730
lytic enzyme
Salmonella enterica subsp. enterica serovar Enteritidis str. EC20111561 genome−p.c.VAL54.69%−taxID1412572
AV50_06545
membrane protein
AV50_06570
hypothetical protein
AV50_06525
AV50_06530
AV50_06560
integrase
AV50_06565
excisionase
subunit theta
Salmonella enterica subsp. enterica serovar Enteritidis str. EC20111561 genome−p.c.VAL98.02%−taxID1412572
AU27_06705
tail fiber assembly protein
AU27_06715
AU27_06745
AU27_06710
type III secretion protein SopE2
AU27_06720
hypothetical protein
AU27_06730
integrase
AU27_06735
exodeoxyribonuclease VIII
AU27_06750
lytic enzyme
Salmonella enterica subsp. enterica serovar Enteritidis str. EC20110353

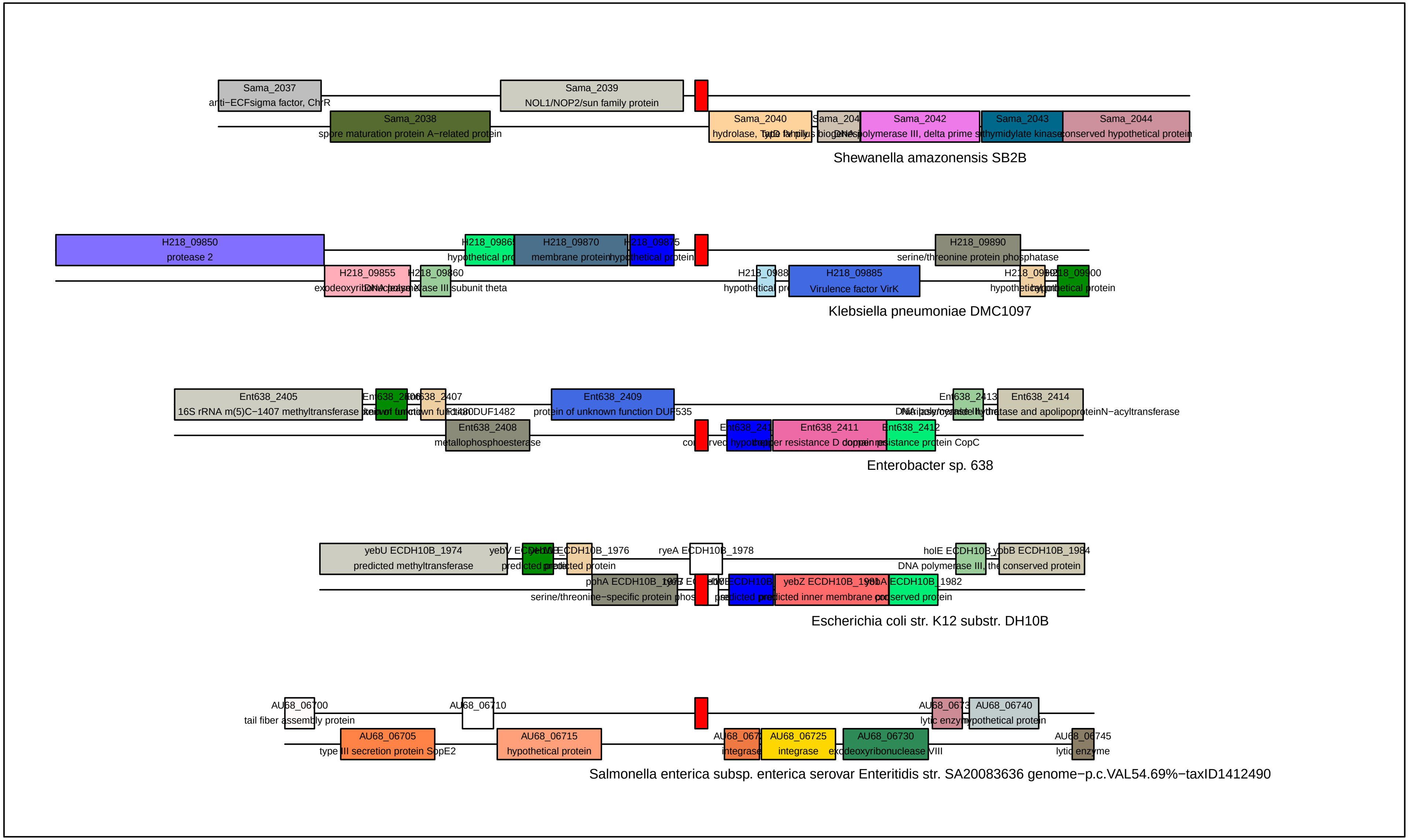

Sama_2037
Sama_2039
NOL1/NOP2/sun family protein
Sama_2038
Sama_2040
Sama_2042
Sama_2043
Sama_2044
Shewanella amazonensis SB2B
H218_09850
protease 2
H218_09855
H218_09860
H218_09870
H218_09885
Virulence factor VirK
H218_09890
serine/threonine protein phosphatase
Klebsiella pneumoniae DMC1097
Ent638_2405
16S rRNA m(5)C−1407 methyltransferase
Ent638_2407
Ent638_2408
metallophosphoesterase
Ent638_2409
Ent638_2411
Ent638_2412
Ent638_2414
Enterobacter sp. 638
yebU ECDH10B_1974
predicted methyltransferase
Escherichia coli str. K12 substr. DH10B
AU68_06705
AU68_06710
AU68_06715
hypothetical protein
AU68_06725
integrase
AU68_06730
AU68_06740
AU68_06745
lytic enzyme
Salmonella enterica subsp. enterica serovar Enteritidis str. SA20083636 genome−p.c.VAL54.69%−taxID1412490

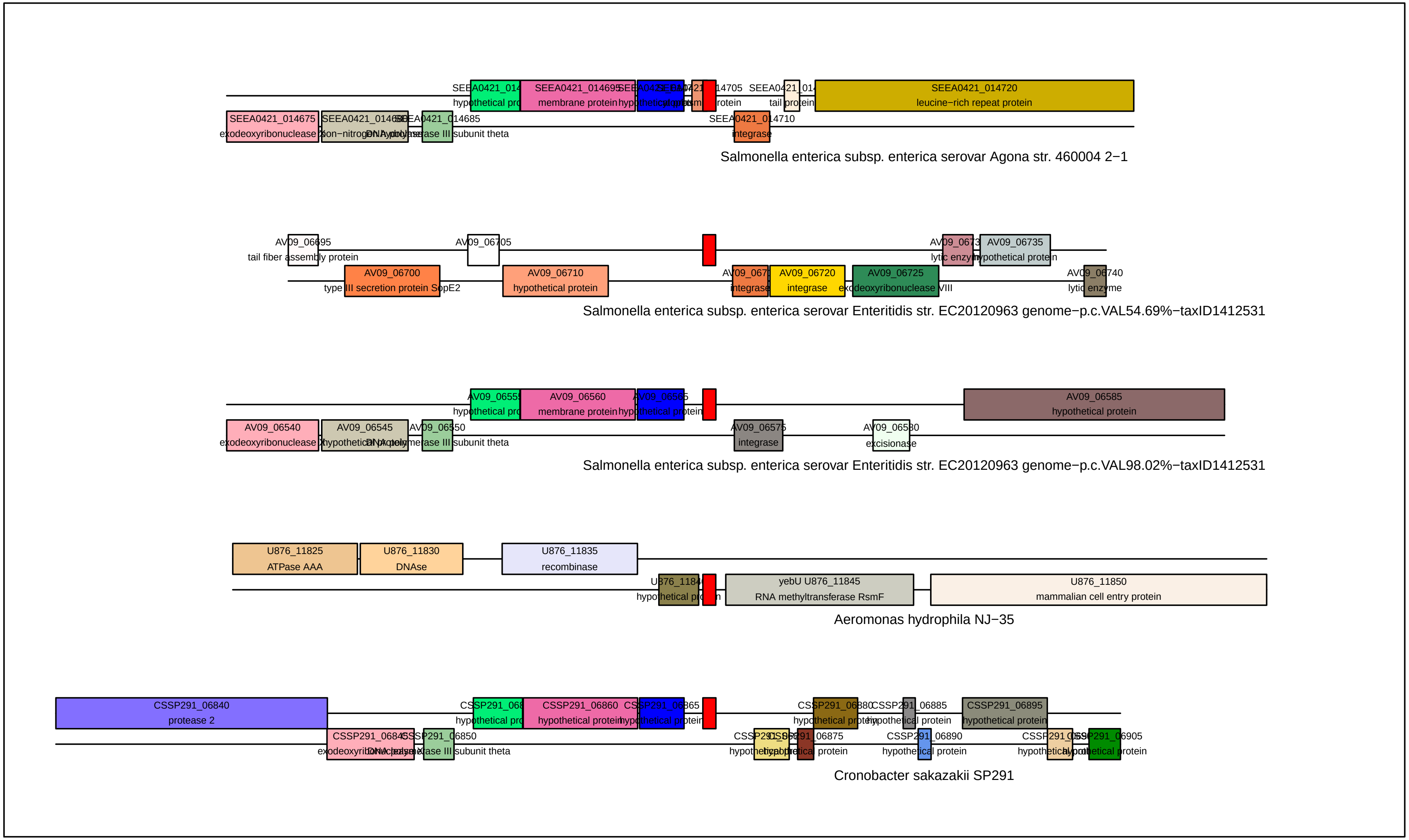
SEEA0421_014695
membrane protein
SEEA0421_014720
leucine−rich repeat protein
SEEA0421_014675
exodeoxyribonuclease
SEEA0421_014685
subunit theta
SEEA0421_014710
integrase
tail protein
Salmonella enterica subsp. enterica serovar Agona str. 460004 2−1
AV09_06695
tail fiber assembly protein
AV09_06705
AV09_06735
hypothetical protein
AV09_06700
type III secretion protein SopE2
AV09_06710
hypothetical protein
AV09_06720
integrase
AV09_06725
exodeoxyribonuclease VIII
AV09_06740
lytic enzyme
Salmonella enterica subsp. enterica serovar Enteritidis str. EC20120963 genome−p.c.VAL54.69%−taxID1412531
AV09_06560
membrane protein
AV09_06585
hypothetical protein
AV09_06540
exodeoxyribonuclease
AV09_06545
AV09_06550
subunit theta
AV09_06575
integrase
AV09_06580
excisionase
Salmonella enterica subsp. enterica serovar Enteritidis str. EC20120963 genome−p.c.VAL98.02%−taxID1412531
U876_11825
ATPase AAA
U876_11830
DNAse
U876_11835
recombinase
yebU U876_11845
RNA methyltransferase RsmF
U876_11850
mammalian cell entry protein
Aeromonas hydrophila NJ−35
CSSP291_06840
protease 2
CSSP291_06860
hypothetical protein
CSSP291_06885
hypothetical protein
CSSP291_06895
hypothetical protein
CSSP291_06850
subunit theta
CSSP291_06875
CSSP291_06890
hypothetical protein
CSSP291_06905
Cronobacter sakazakii SP291

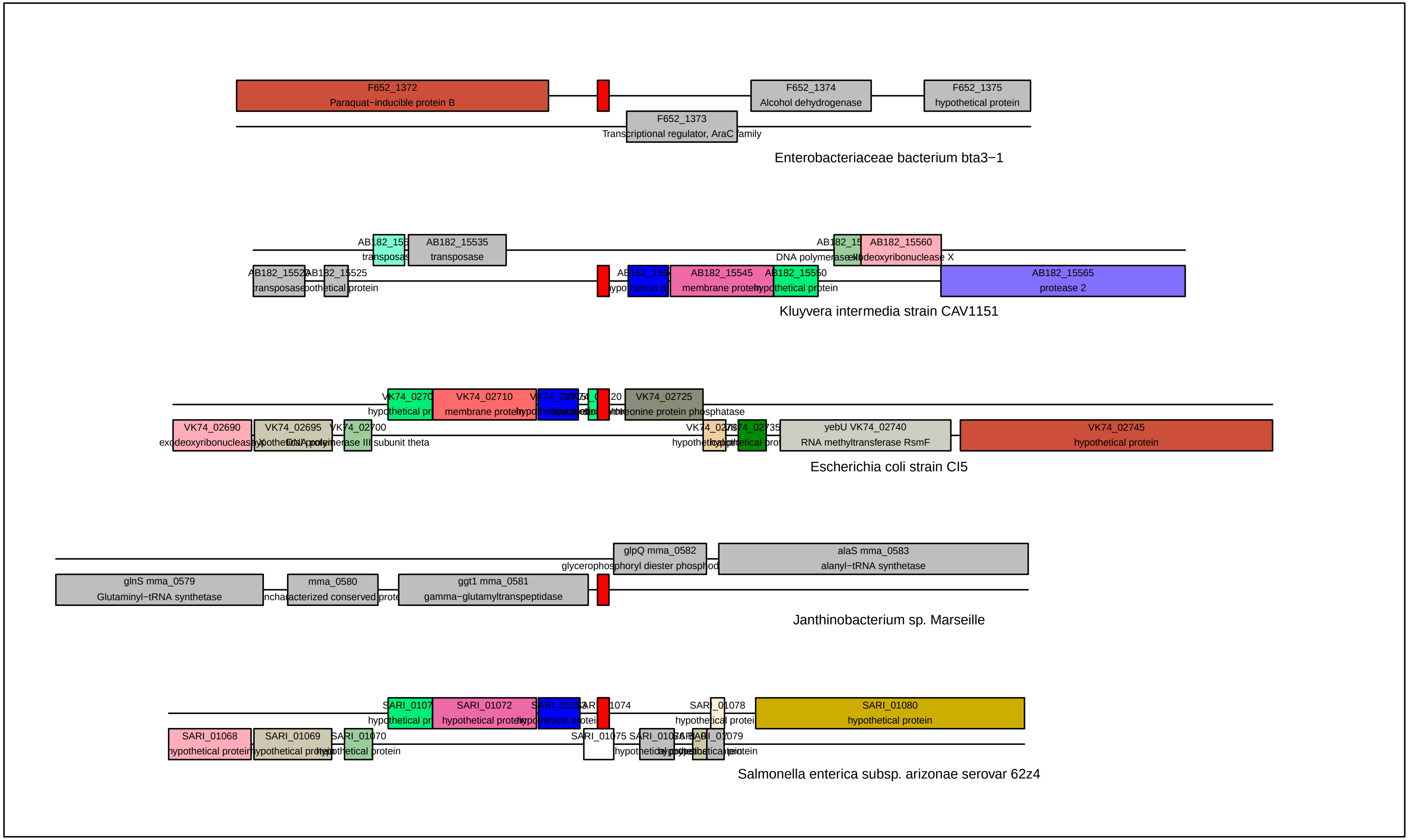
F652_1372
Paraquat−inducible protein B
F652_1374
Alcohol dehydrogenase
F652_1375
hypothetical protein
F652_1373
Transcriptional regulator, AraC family
Enterobacteriaceae bacterium bta3−1
AB182_15535
transposase
AB182_15560
exodeoxyribonuclease X
DNA polymerase
AB182_15545
membrane protein
AB182_15565
protease 2
AB182_15525
hypothetical protein
Kluyvera intermedia strain CAV1151
VK74_02710
membrane protein
VK74_02725
threonine protein phosphatase
VK74_02690
VK74_02695
VK74_02700
DNA polymerase III subunit theta
yebU VK74_02740
RNA methyltransferase RsmF
VK74_02745
hypothetical protein
Escherichia coli strain CI5
glpQ mma_0582
alaS mma_0583
alanyl−tRNA synthetase
glnS mma_0579
Glutaminyl−tRNA synthetase
mma_0580
ggt1 mma_0581
gamma−glutamyltranspeptidase
Janthinobacterium sp. Marseille
SARI_01072
hypothetical protein
SARI_01080
hypothetical protein
SARI_01068
SARI_01069
SARI_01070
SARI_01075
SARI_01078
Salmonella enterica subsp. arizonae serovar 62z4

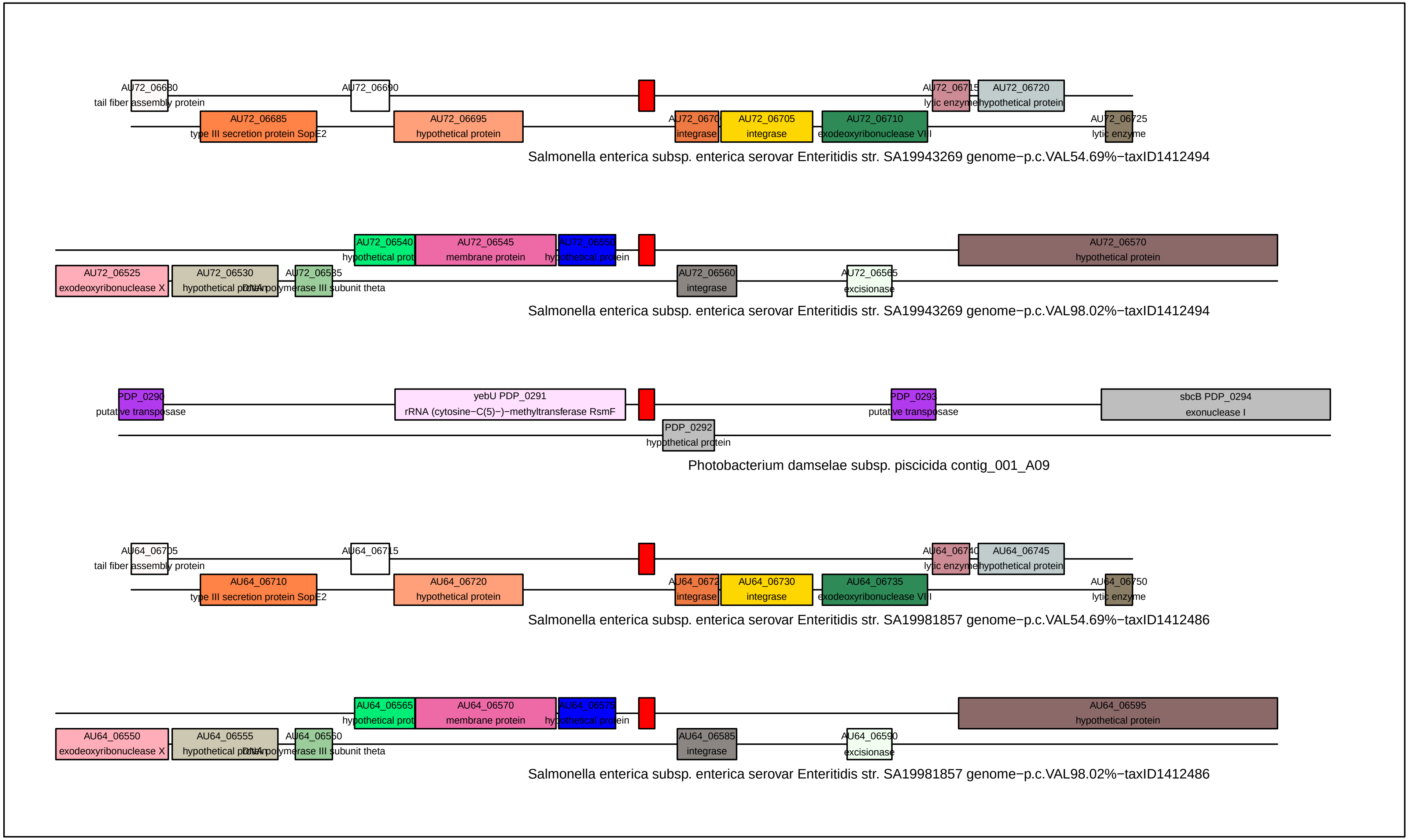

AU72_06680
tail fiber assembly protein
AU72_06690
AU72_06715
lytic enzyme
AU72_06720
hypothetical protein
AU72_06685
type III secretion protein SopE2
AU72_06695
hypothetical protein
AU72_0670
integrase
AU72_06705
integrase
AU72_06710
exodeoxyribonuclease VIII
AU72_06725
lytic enzyme
Salmonella enterica subsp. enterica serovar Enteritidis str. SA19943269 genome−p.c.VAL54.69%−taxID1412494
AU72_06540
hypothetical prot
AU72_06545
membrane protein
AU72_06550
hypothetical protein
AU72_06570
hypothetical protein
AU72_06525
exodeoxyribonuclease X
AU72_06530
hypothetical protein
AU72_06535
DNA polymerase III subunit theta
AU72_06560
integrase
AU72_06565
excisionase
Salmonella enterica subsp. enterica serovar Enteritidis str. SA19943269 genome−p.c.VAL98.02%−taxID1412494
PDP_0290
putative transposase
yebU PDP_0291
rRNA (cytosine−C(5)−)−methyltransferase RsmF
PDP_0293
putative transposase
sbcB PDP_0294
exonuclease I
PDP_0292
hypothetical protein
Photobacterium damselae subsp. piscicida contig_001_A09
AU64_06705
tail fiber assembly protein
AU64_06715
AU64_06740
lytic enzyme
AU64_06745
hypothetical protein
AU64_06710
type III secretion protein SopE2
AU64_06720
hypothetical protein
AU64_0672
integrase
AU64_06730
integrase
AU64_06735
exodeoxyribonuclease VIII
AU64_06750
lytic enzyme
Salmonella enterica subsp. enterica serovar Enteritidis str. SA19981857 genome−p.c.VAL54.69%−taxID1412486
AU64_06565
hypothetical prot
AU64_06570
membrane protein
AU64_06575
hypothetical protein
AU64_06595
hypothetical protein
AU64_06550
exodeoxyribonuclease X
AU64_06555
hypothetical protein
AU64_06560
DNA polymerase III subunit theta
AU64_06585
integrase
AU64_06590
excisionase
Salmonella enterica subsp. enterica serovar Enteritidis str. SA19981857 genome−p.c.VAL98.02%−taxID1412486

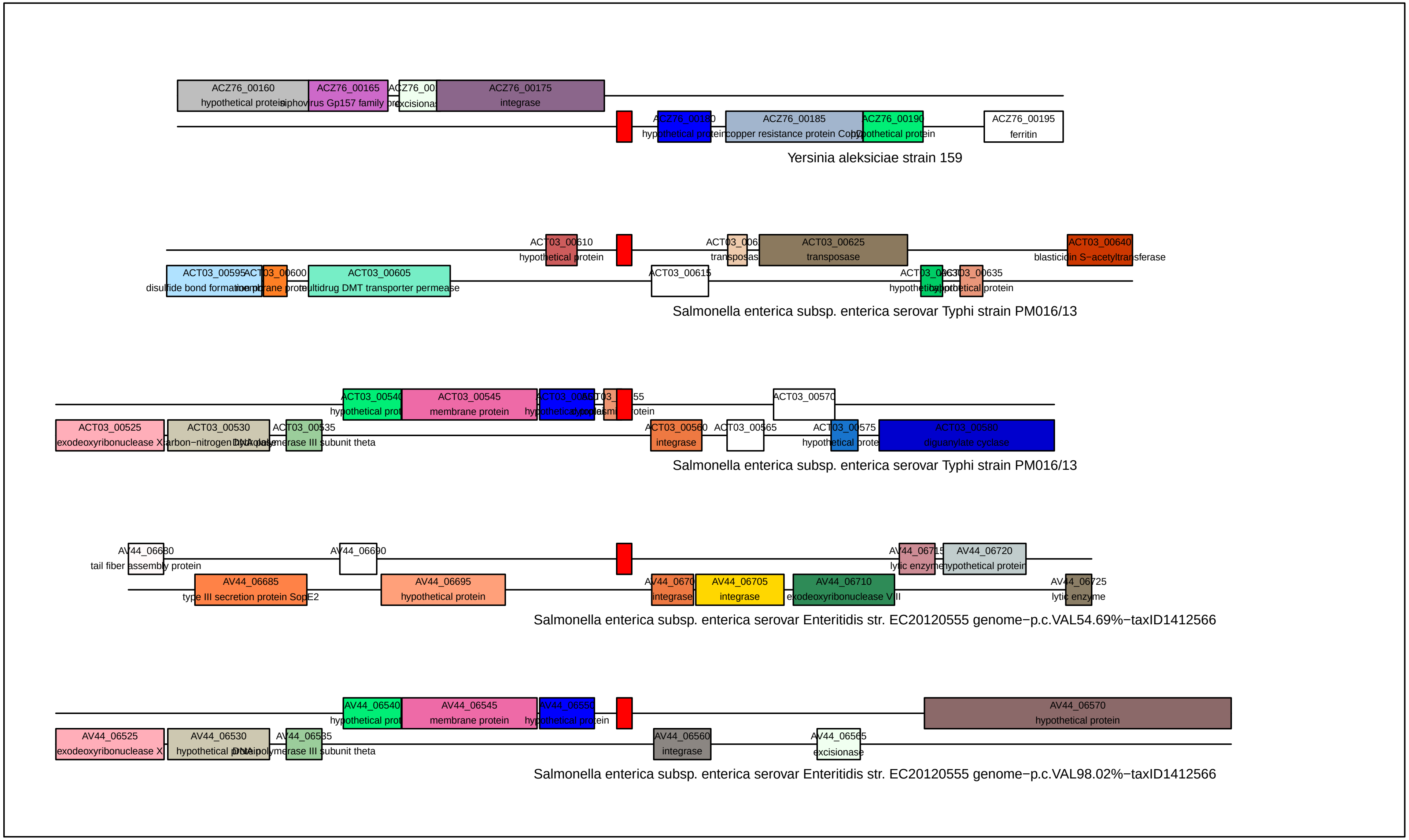

ACZ76_00160
hypothetical protein
ACZ76_00165
ACZ76_00175
integrase
ACZ76_00185
copper resistance protein CopD
ACZ76_00190
ACZ76_00195
ferritin
Yersinia aleksiciae strain 159
ACT03_00610
hypothetical protein
ACT03_00625
transposase
ACT03_00640
blasticidin S−acetyltransferase
ACT03_00595
ACT03_00600
ACT03_00605
multidrug DMT transporter permease
ACT03_00615
ACT03_00635
hypothetical protein
Salmonella enterica subsp. enterica serovar Typhi strain PM016/13
ACT03_00540
ACT03_00545
membrane protein
ACT03_00570
ACT03_00525
exodeoxyribonuclease X
ACT03_00530
ACT03_00535
DNA polymerase III subunit theta
ACT03_00560
integrase
ACT03_00565
ACT03_00575
ACT03_00580
diguanylate cyclase
Salmonella enterica subsp. enterica serovar Typhi strain PM016/13
AV44_06680
tail fiber assembly protein
AV44_06690
AV44_06715
lytic enzyme
AV44_06720
hypothetical protein
AV44_06685
type III secretion protein SopE2
AV44_06695
hypothetical protein
AV44_06705
integrase
AV44_06710
exodeoxyribonuclease VIII
AV44_06725
lytic enzyme
Salmonella enterica subsp. enterica serovar Enteritidis str. EC20120555 genome−p.c.VAL54.69%−taxID1412566
AV44_06540
AV44_06545
membrane protein
AV44_06570
hypothetical protein
AV44_06525
exodeoxyribonuclease X
AV44_06530
hypothetical protein
AV44_06535
DNA polymerase III subunit theta
AV44_06560
integrase
AV44_06565
excisionase
Salmonella enterica subsp. enterica serovar Enteritidis str. EC20120555 genome−p.c.VAL98.02%−taxID1412566

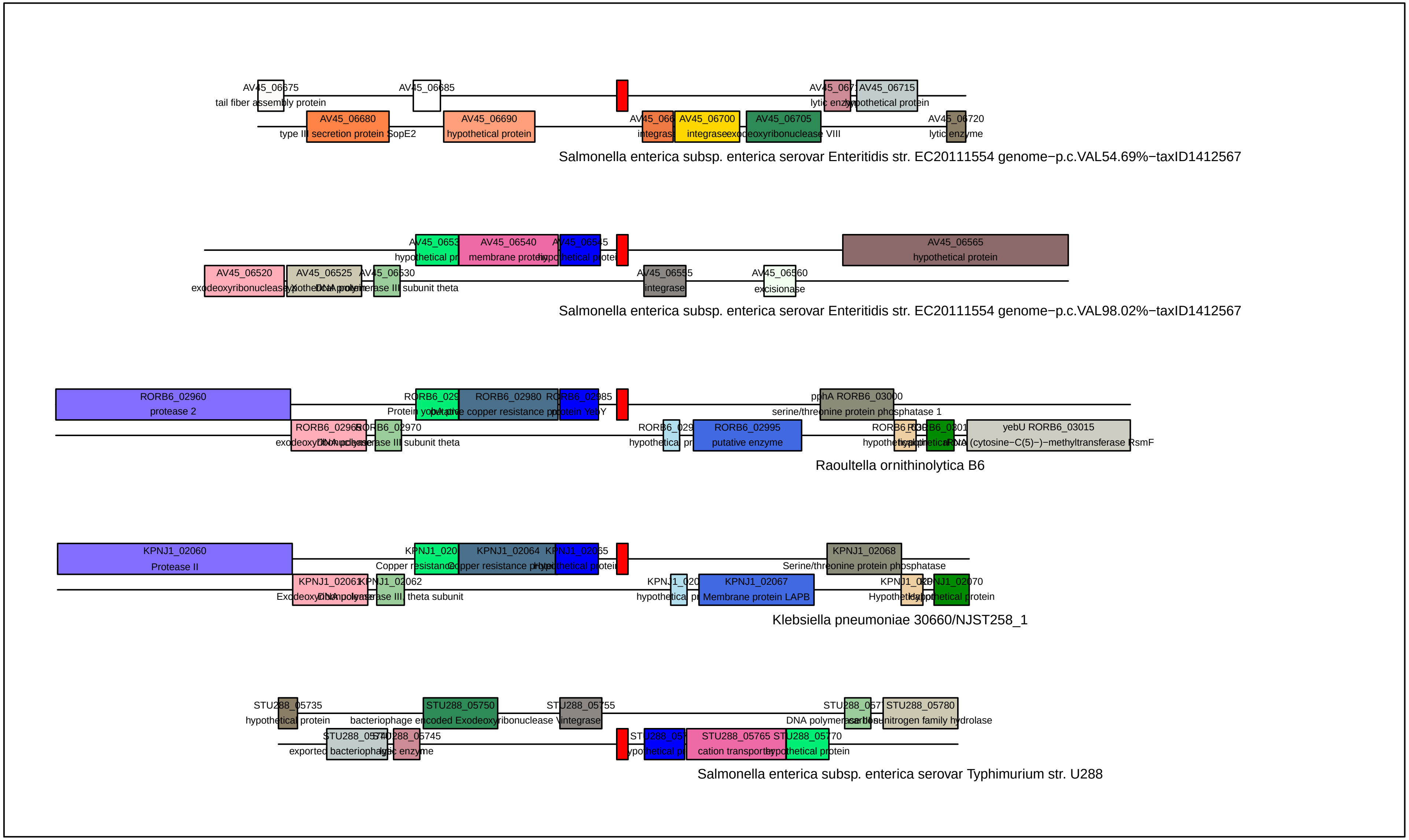

AV45_06675
tail fiber assembly protein
AV45_06685
AV45_06715
hypothetical protein
AV45_06680
type III secretion protein SopE2
AV45_06690
hypothetical protein
AV45_06700
integrase
AV45_06705
exodeoxyribonuclease VIII
AV45_06720
lytic enzyme
Salmonella enterica subsp. enterica serovar Enteritidis str. EC20111554 genome−p.c.VAL54.69%−taxID1412567
AV45_06540
membrane protein
AV45_06545
AV45_06565
hypothetical protein
AV45_06520
exodeoxyribonuclease
AV45_06525
AV45_06530
DNA polymerase III subunit theta
AV45_06555
integrase
AV45_06560
excisionase
Salmonella enterica subsp. enterica serovar Enteritidis str. EC20111554 genome−p.c.VAL98.02%−taxID1412567
RORB6_02960
protease 2
RORB6_02980
putative copper resistance protein
RORB6_02985
pphA RORB6_03000
serine/threonine protein phosphatase 1
RORB6_02965
RORB6_02970
DNA polymerase III subunit theta
RORB6_02995
putative enzyme
yebU RORB6_03015
rRNA (cytosine−C(5)−)−methyltransferase RsmF
Raoultella ornithinolytica B6
KPNJ1_02060
Protease II
KPNJ1_02064
Copper resistance protein
KPNJ1_02065
KPNJ1_02068
Serine/threonine protein phosphatase
KPNJ1_02061
KPNJ1_02062
DNA polymerase III, theta subunit
KPNJ1_02067
Membrane protein LAPB
KPNJ1_02070
Hypothetical protein
Klebsiella pneumoniae 30660/NJST258_1
STU288_05735
hypothetical protein
STU288_05750
bacteriophage encoded Exodeoxyribonuclease V
STU288_05755
integrase
STU288_05780
carbon−nitrogen family hydrolase
STU288_05745
lytic enzyme
STU288_05765
cation transporter
STU288_05770
hypothetical protein
Salmonella enterica subsp. enterica serovar Typhimurium str. U288

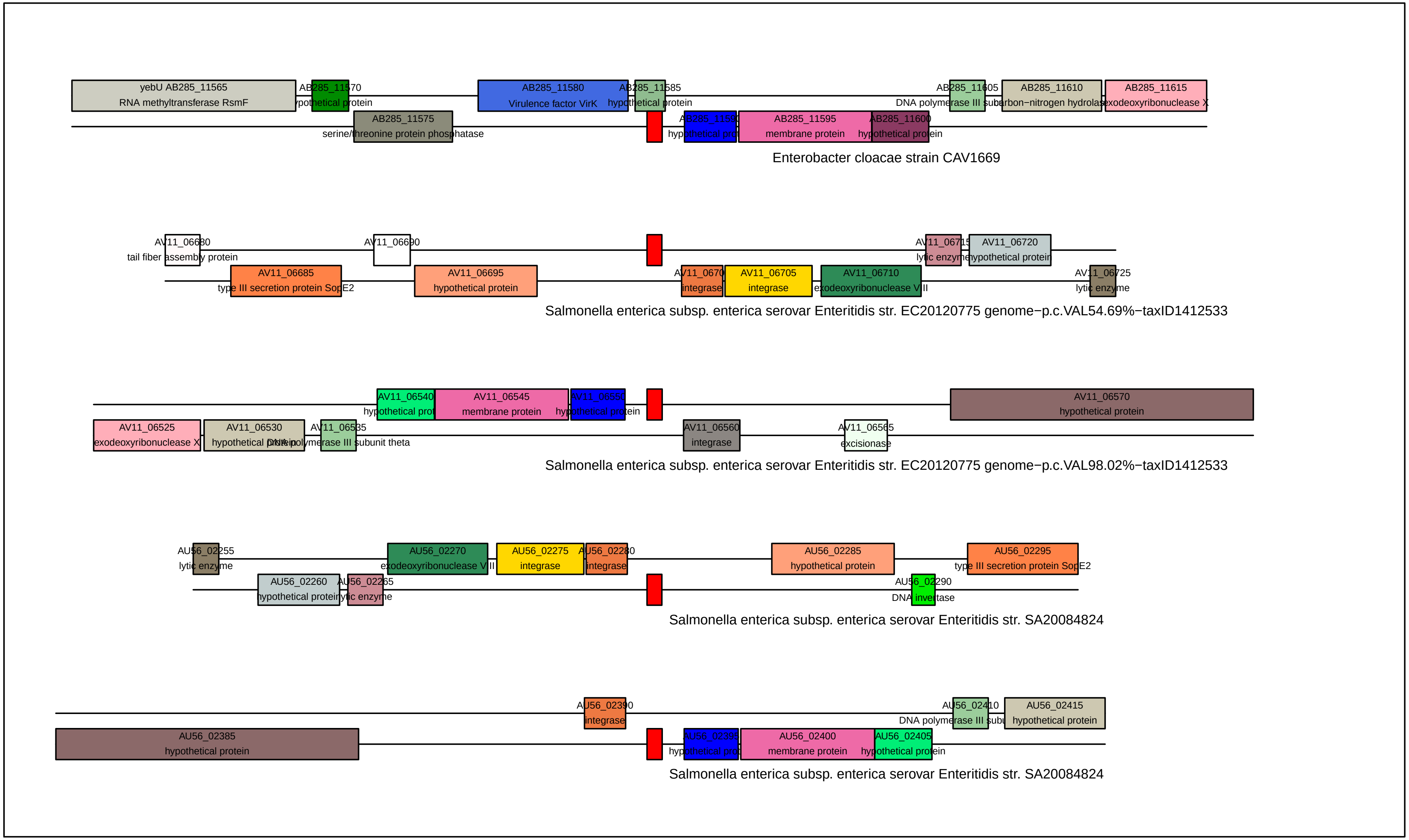

yebU AB285_11565
RNA methyltransferase RsmF
AB285_11570
hypothetical protein
AB285_11575
serine/threonine protein phosphatase
AB285_11580
Virulence factor VirK
AB285_11585
hypothetical protein
AB285_11590
hypothetical protein
AB285_11595
membrane protein
AB285_11600
hypothetical protein
AB285_11605
DNA polymerase III subunit
AB285_11610
carbon−nitrogen hydrolase
AB285_11615
exodeoxyribonuclease X
Enterobacter cloacae strain CAV1669
AV11_06680
tail fiber assembly protein
AV11_06685
type III secretion protein SopE2
AV11_06690
AV11_06695
hypothetical protein
AV11_06700
integrase
AV11_06705
integrase
AV11_06710
exodeoxyribonuclease VIII
AV11_06715
lytic enzyme
AV11_06720
hypothetical protein
AV11_06725
lytic enzyme
Salmonella enterica subsp. enterica serovar Enteritidis str. EC20120775 genome−p.c.VAL54.69%−taxID1412533
AV11_06525
exodeoxyribonuclease X
AV11_06530
hypothetical protein
AV11_06535
DNA polymerase III subunit theta
AV11_06540
hypothetical protein
AV11_06545
membrane protein
AV11_06550
hypothetical protein
AV11_06560
integrase
AV11_06565
excisionase
AV11_06570
hypothetical protein
Salmonella enterica subsp. enterica serovar Enteritidis str. EC20120775 genome−p.c.VAL98.02%−taxID1412533
AU56_02255
lytic enzyme
AU56_02260
hypothetical protein
AU56_02265
lytic enzyme
AU56_02270
exodeoxyribonuclease VIII
AU56_02275
integrase
AU56_02280
integrase
AU56_02285
hypothetical protein
AU56_02290
DNA invertase
AU56_02295
type III secretion protein SopE2
Salmonella enterica subsp. enterica serovar Enteritidis str. SA20084824
AU56_02385
hypothetical protein
AU56_02390
integrase
AU56_02395
hypothetical protein
AU56_02400
membrane protein
AU56_02405
hypothetical protein
AU56_02410
DNA polymerase III subunit
AU56_02415
hypothetical protein
Salmonella enterica subsp. enterica serovar Enteritidis str. SA20084824

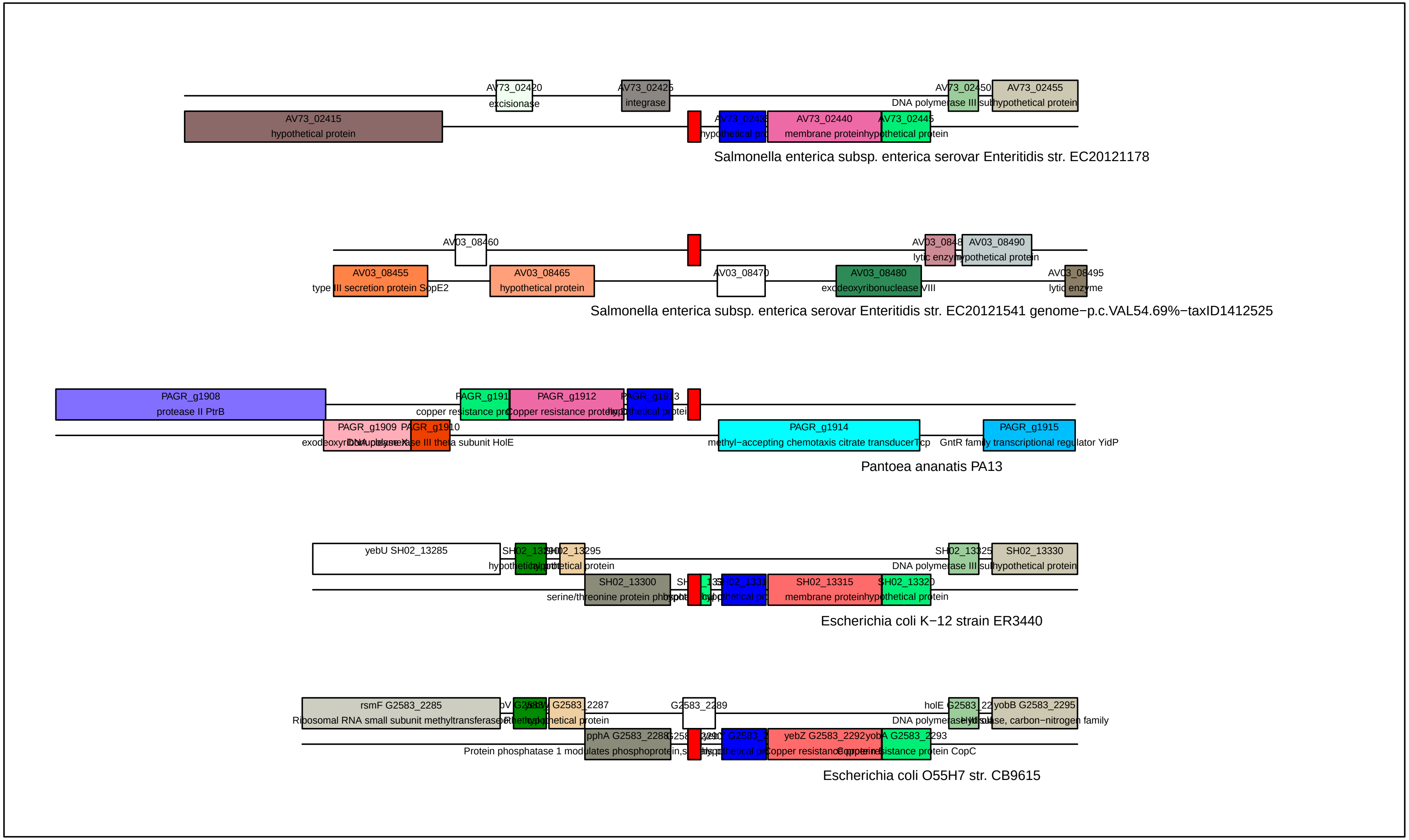
AV73_02420
excisionase
AV73_02425
integrase
AV73_02450
AV73_02455
hypothetical protein
AV73_02415
hypothetical protein
AV73_02440
membrane protein
AV73_02445
Salmonella enterica subsp. enterica serovar Enteritidis str. EC20121178
AV03_08460
AV03_08490
hypothetical protein
AV03_08455
type III secretion protein SopE2
AV03_08465
hypothetical protein
AV03_08470
AV03_08480
exodeoxyribonuclease VIII
AV03_08495
lytic enzyme
Salmonella enterica subsp. enterica serovar Enteritidis str. EC20121541 genome−p.c.VAL54.69%−taxID1412525
PAGR_g1908
protease II PtrB
PAGR_g1912
PAGR_g1909
PAGR_g1910
PAGR_g1914
methyl−accepting chemotaxis citrate transducer Tcp
PAGR_g1915
GntR family transcriptional regulator YidP
Pantoea ananatis PA13
yebU SH02_13285
SH02_13295
SH02_13325
SH02_13330
hypothetical protein
SH02_13300
SH02_13315
membrane protein
SH02_13320
Escherichia coli K−12 strain ER3440
rsmF G2583_2285
G2583_2287
G2583_2289
yobB G2583_2295
pphA G2583_2288
yebZ G2583_2292
G2583_2293
Escherichia coli O55H7 str. CB9615

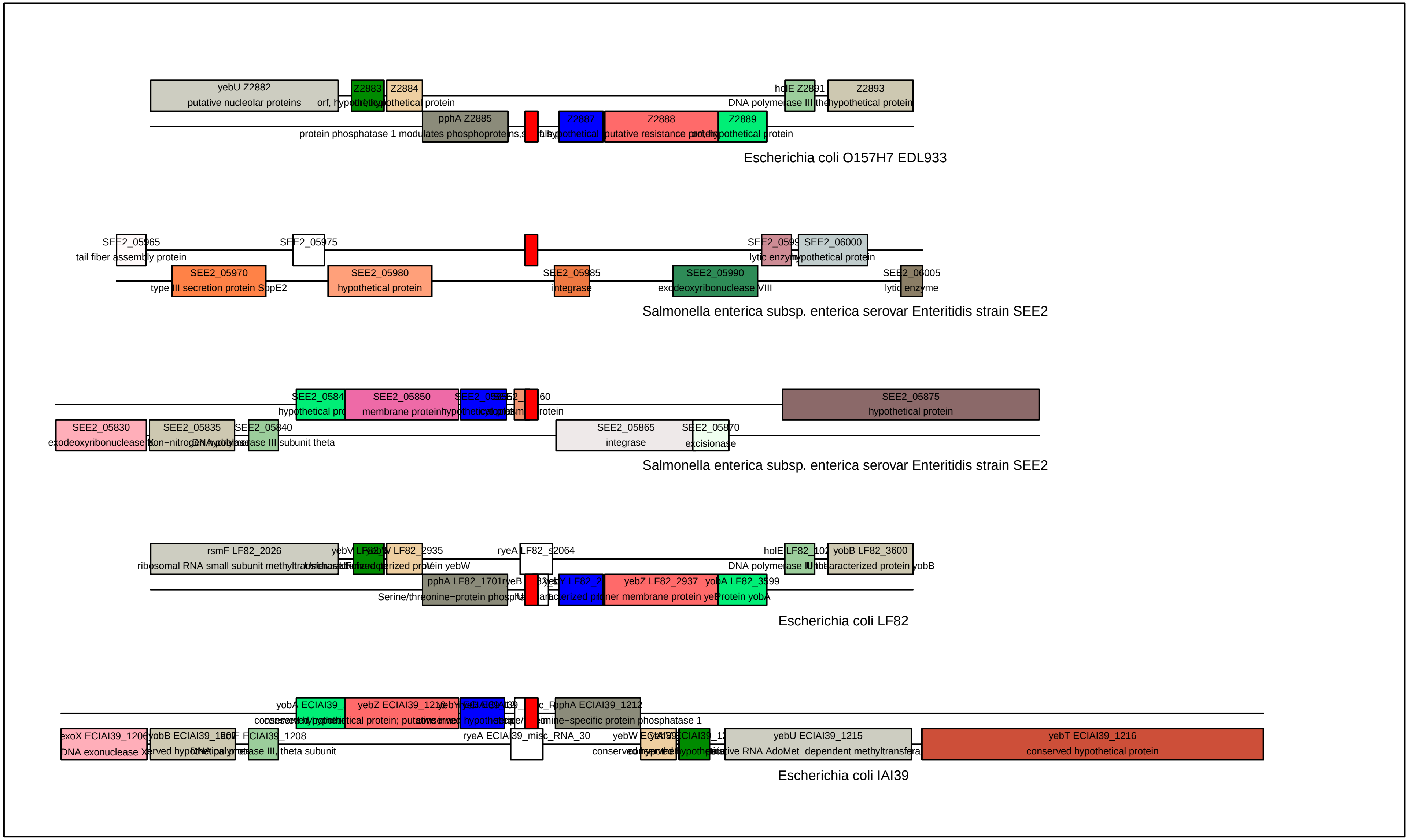

yebU Z2882
putative nucleolar proteins
Z2883
Z2884
hypothetical protein
holE Z2891
DNA polymerase III
Z2893
hypothetical protein
pphA Z2885
protein phosphatase 1 modulates phosphoproteins
Z2887
Z2888
putative resistance protein
Z2889
hypothetical protein
Escherichia coli O157H7 EDL933
SEE2_05965
tail fiber assembly protein
SEE2_05975
SEE2_06000
hypothetical protein
SEE2_05970
type III secretion protein SopE2
SEE2_05980
hypothetical protein
SEE2_05985
integrase
SEE2_05990
exodeoxyribonuclease VIII
SEE2_06005
lytic enzyme
Salmonella enterica subsp. enterica serovar Enteritidis strain SEE2
SEE2_05850
membrane protein
SEE2_05875
hypothetical protein
SEE2_05830
exodeoxyribonuclease X
SEE2_05835
SEE2_05840
subunit theta
SEE2_05865
integrase
SEE2_05870
excisionase
Salmonella enterica subsp. enterica serovar Enteritidis strain SEE2
rsmF LF82_2026
ryeA LF82_s2064
yobB LF82_3600
pphA LF82_1701
yebZ LF82_2937
Escherichia coli LF82
yobA ECIAI39_
yebZ ECIAI39_121
pphA ECIAI39_1212
phosphatase 1
exoX ECIAI39_1206
DNA exonuclease X
theta subunit
ryeA ECIAI39_misc_RNA_30
yebU ECIAI39_1215
yebT ECIAI39_1216
conserved hypothetical protein
Escherichia coli IAI39

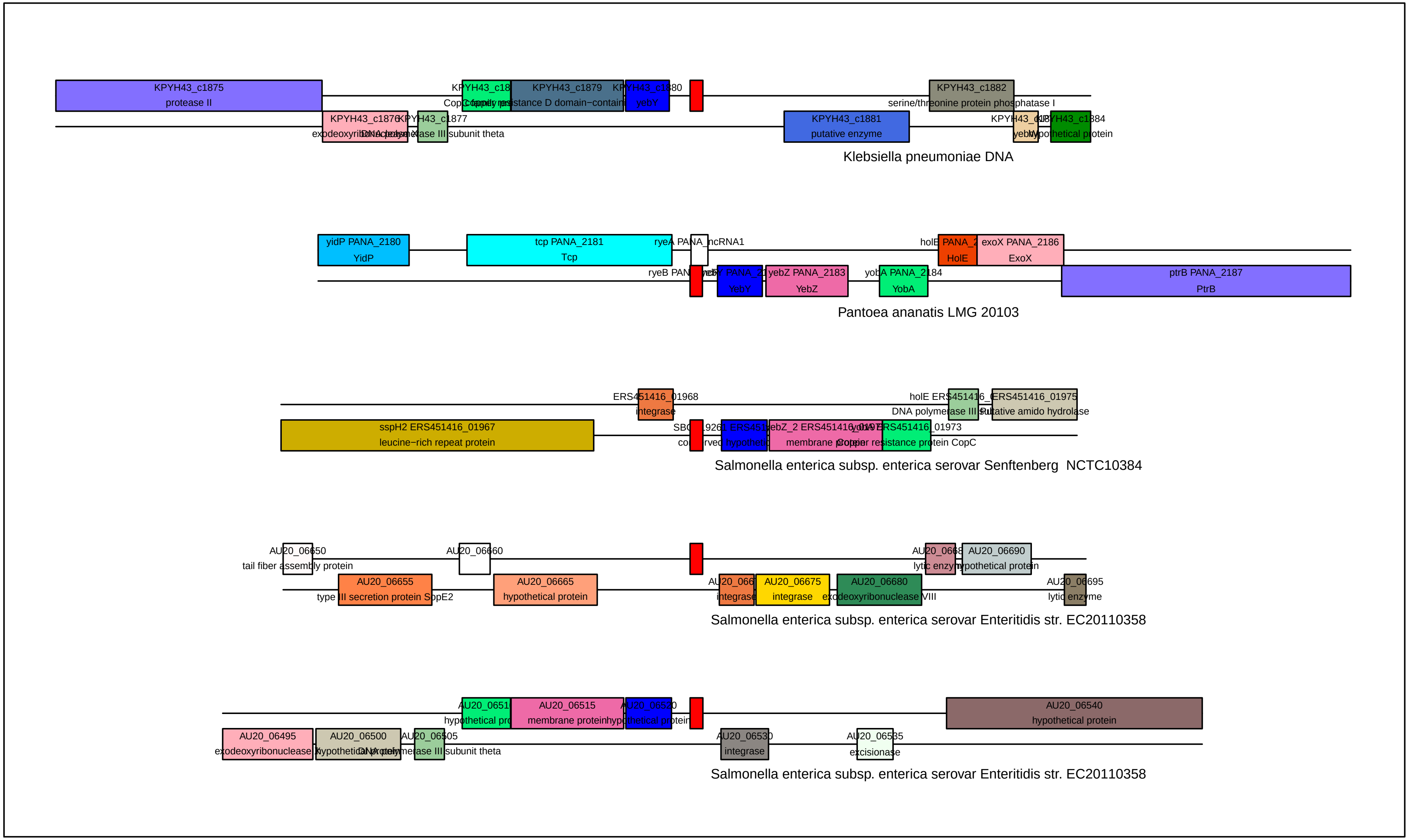

KPYH43_c1875
protease II
KPYH43_c1879
KPYH43_c1882
serine/threonine protein phosphatase I
KPYH43_c1877
subunit theta
KPYH43_c1881
putative enzyme
Klebsiella pneumoniae DNA
yidP PANA_2180
YidP
tcp PANA_2181
Tcp
ryeA PANA_ncRNA1
exoX PANA_2186
ExoX
HolE
yebZ PANA_2183
YebZ
YobA
ptrB PANA_2187
PtrB
Pantoea ananatis LMG 20103
ERS451416_01975
integrase
sspH2 ERS451416_01967
leucine−rich repeat protein
Salmonella enterica subsp. enterica serovar Senftenberg NCTC10384
AU20_06690
hypothetical protein
AU20_06655
type III secretion protein SopE2
AU20_06665
hypothetical protein
AU20_06675
integrase
AU20_06680
exodeoxyribonuclease VIII
Salmonella enterica subsp. enterica serovar Enteritidis str. EC20110358
AU20_06515
AU20_06540
hypothetical protein
AU20_06495
AU20_06500
AU20_06530
integrase
excisionase
Salmonella enterica subsp. enterica serovar Enteritidis str. EC20110358

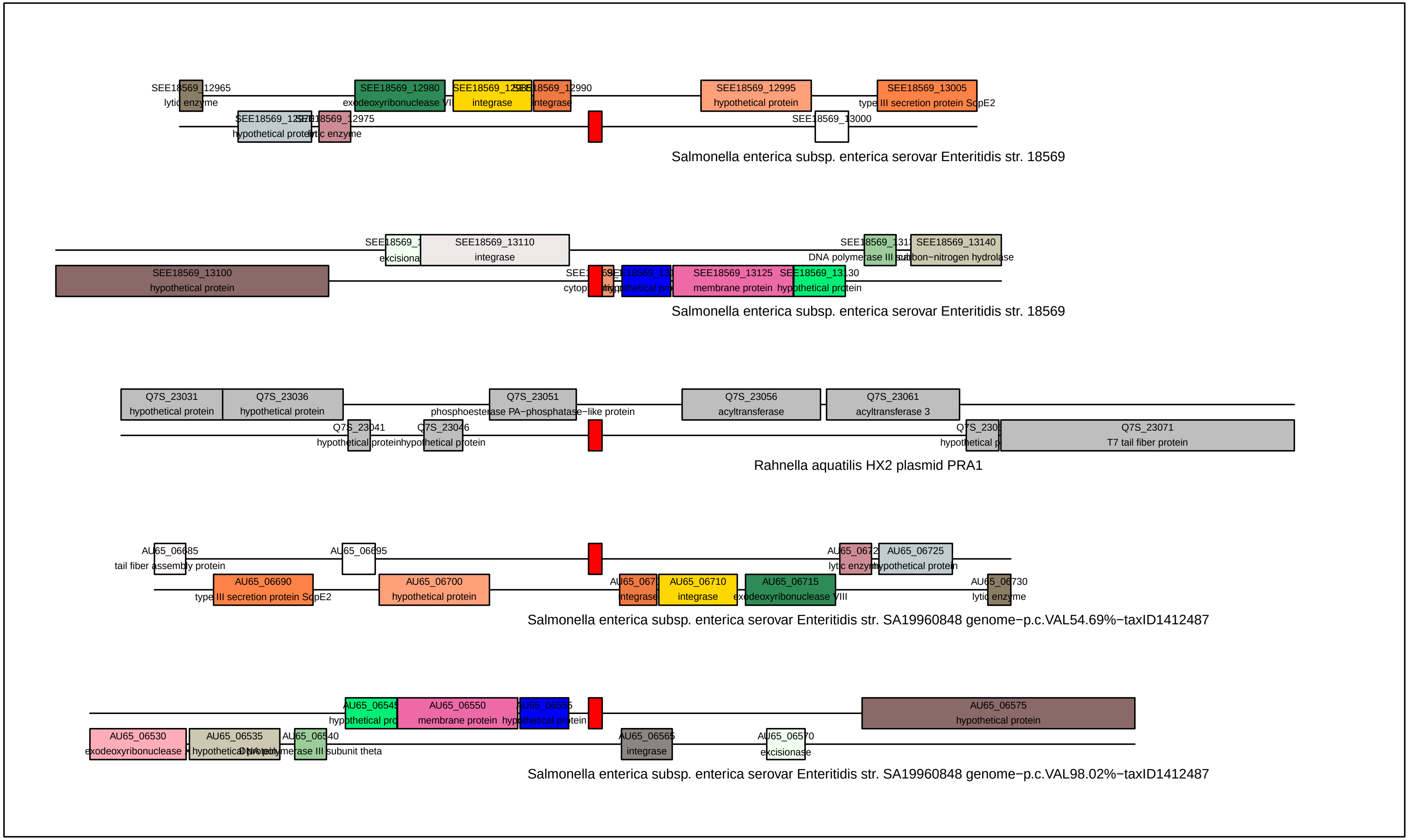

SEE18569_12965
lytic enzyme
SEE18569_12980
SEE18569_12990
integrase
SEE18569_12995
hypothetical protein
SEE18569_13005
type III secretion protein SopE2
SEE18569_12975
SEE18569_13000
Salmonella enterica subsp. enterica serovar Enteritidis str. 18569
SEE18569_13110
integrase
SEE18569_13140
carbon−nitrogen hydrolase
SEE18569_13100
hypothetical protein
SEE18569_13125
membrane protein
Salmonella enterica subsp. enterica serovar Enteritidis str. 18569
Q7S_23031
hypothetical protein
Q7S_23036
hypothetical protein
Q7S_23051
phosphoesterase PA−phosphatase−like protein
Q7S_23056
acyltransferase
Q7S_23061
acyltransferase 3
Q7S_23041
Q7S_23046
hypothetical protein
Q7S_23071
T7 tail fiber protein
Rahnella aquatilis HX2 plasmid PRA1
AU65_06685
tail fiber assembly protein
AU65_06695
AU65_06725
AU65_06690
type III secretion protein SopE2
AU65_06700
hypothetical protein
AU65_06710
integrase
AU65_06715
exodeoxyribonuclease VIII
AU65_06730
lytic enzyme
Salmonella enterica subsp. enterica serovar Enteritidis str. SA19960848 genome−p.c.VAL54.69%−taxID1412487
AU65_06550
membrane protein
AU65_06575
hypothetical protein
AU65_06530
exodeoxyribonuclease X
AU65_06535
AU65_06540
DNA polymerase III subunit theta
AU65_06565
integrase
AU65_06570
excisionase
Salmonella enterica subsp. enterica serovar Enteritidis str. SA19960848 genome−p.c.VAL98.02%−taxID1412487

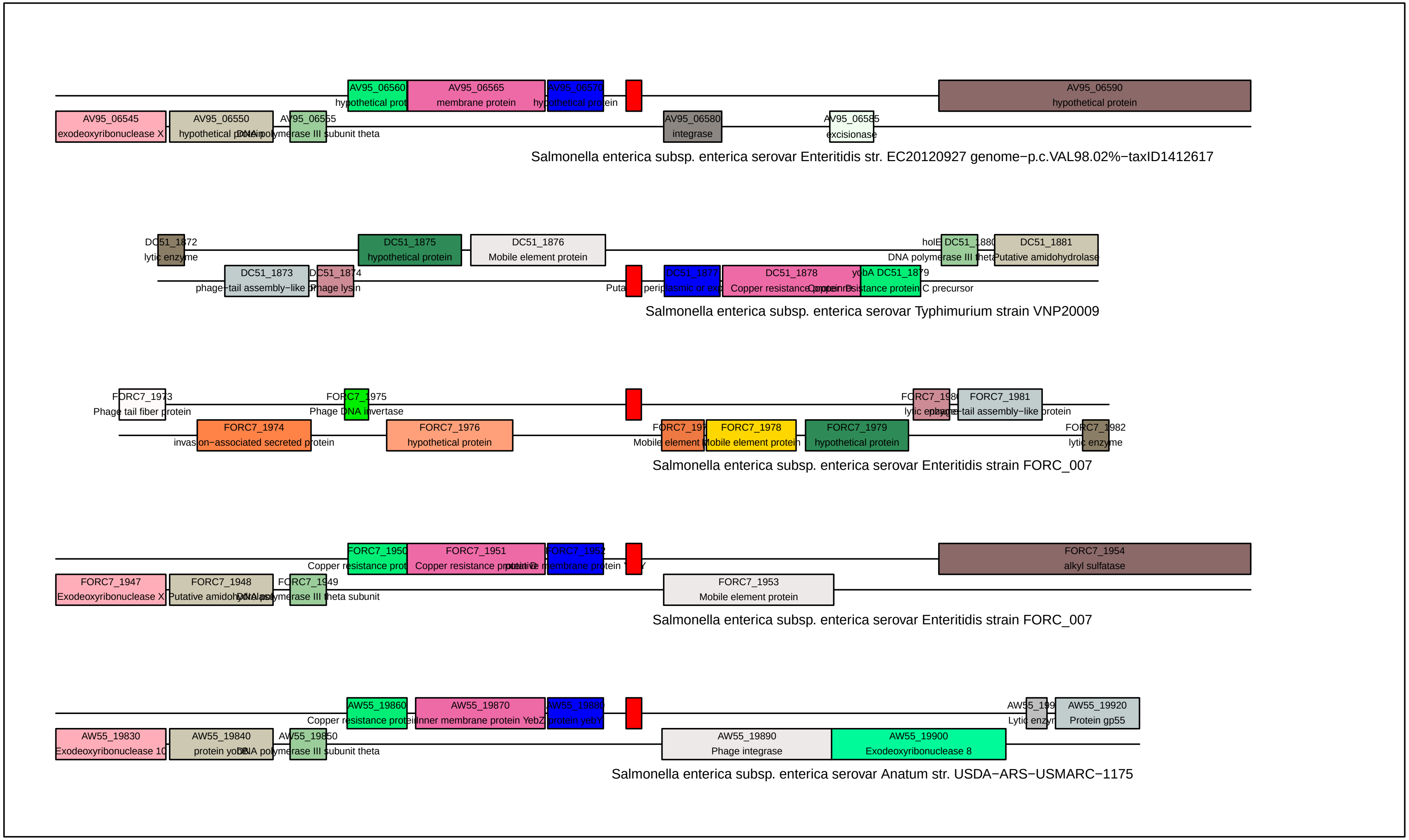

AV95_06545
exodeoxyribonuclease X
AV95_06550
hypothetical protein
DNA polymerase III subunit theta
AV95_06560
AV95_06565
membrane protein
AV95_06580
integrase
AV95_06585
excisionase
AV95_06590
hypothetical protein
Salmonella enterica subsp. enterica serovar Enteritidis str. EC20120927 genome−p.c.VAL98.02%−taxID1412617
DC51_1872
lytic enzyme
DC51_1873
DC51_1874
Phage lysin
DC51_1875
hypothetical protein
DC51_1876
Mobile element protein
DC51_1878
DC51_1879
DC51_1881
Putative amidohydrolase
C precursor
Salmonella enterica subsp. enterica serovar Typhimurium strain VNP20009
FORC7_1973
Phage tail fiber protein
FORC7_1974
invasion−associated secreted protein
FORC7_1975
Phage DNA invertase
FORC7_1976
hypothetical protein
FORC7_1978
Mobile element protein
FORC7_1979
hypothetical protein
FORC7_1981
phage−tail assembly−like protein
FORC7_1982
lytic enzyme
Salmonella enterica subsp. enterica serovar Enteritidis strain FORC_007
FORC7_1947
Exodeoxyribonuclease X
FORC7_1948
FORC7_1949
theta subunit
FORC7_1950
FORC7_1951
FORC7_1952
FORC7_1953
Mobile element protein
FORC7_1954
alkyl sulfatase
Salmonella enterica subsp. enterica serovar Enteritidis strain FORC_007
AW55_19830
AW55_19840
AW55_19850
DNA polymerase III subunit theta
AW55_19860
AW55_19870
Inner membrane protein YebZ
AW55_19890
Phage integrase
AW55_19900
Exodeoxyribonuclease 8
AW55_19920
Protein gp55
Salmonella enterica subsp. enterica serovar Anatum str. USDA−ARS−USMARC−1175

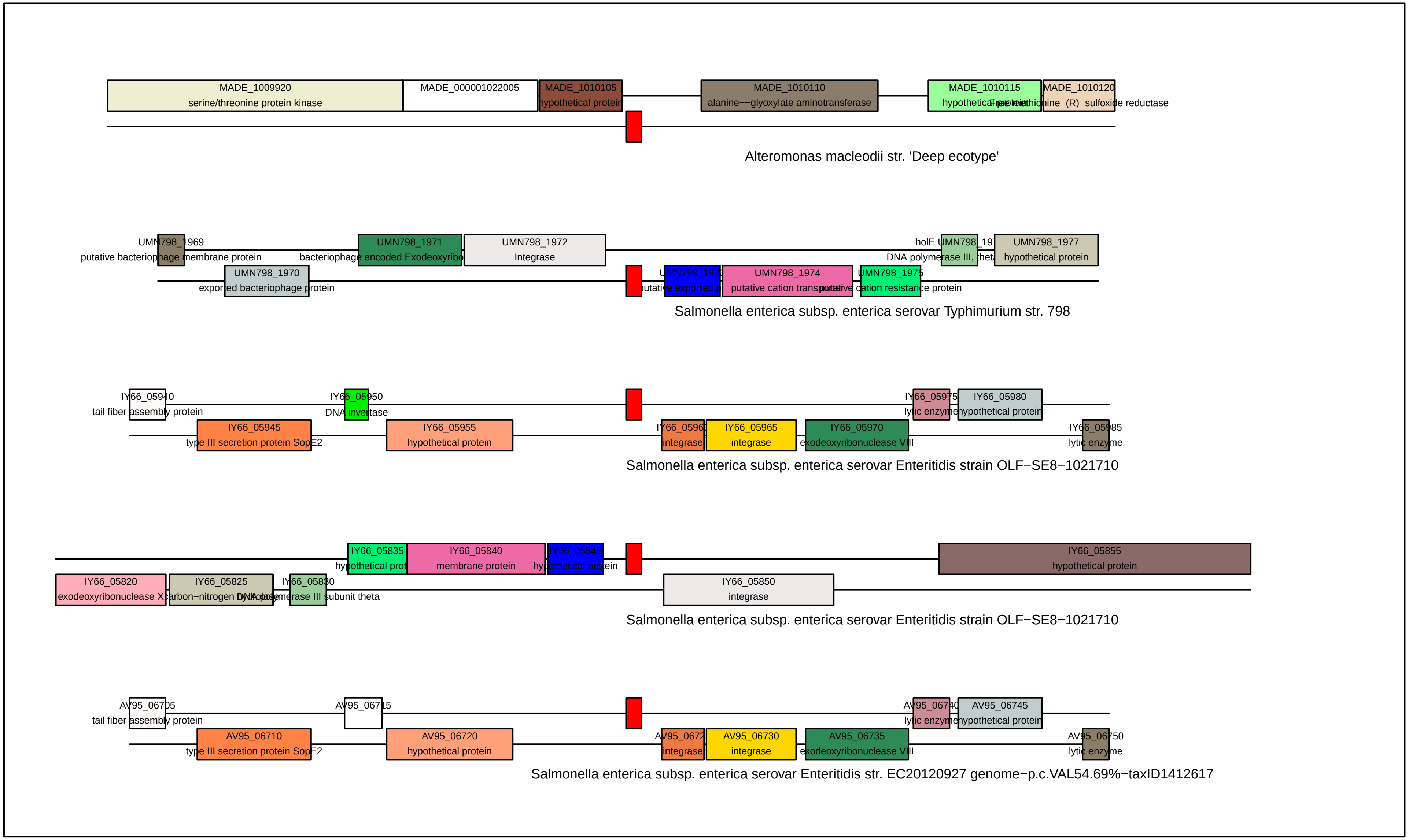
MADE_1009920
serine/threonine protein kinase
MADE_000001022005
MADE_1010105
hypothetical protein
MADE_1010110
alanine−−glyoxylate aminotransferase
MADE_1010115
MADE_1010120
Alteromonas macleodii str. 'Deep ecotype'
UMN798_1969
putative bacteriophage membrane protein
UMN798_1971
UMN798_1972
Integrase
UMN798_1977
hypothetical protein
holE
UMN798_1970
exported bacteriophage protein
putative cation resistance protein
Salmonella enterica subsp. enterica serovar Typhimurium str. 798
IY66_05940
tail fiber assembly protein
IY66_05950
DNA invertase
IY66_05980
hypothetical protein
IY66_05945
type III secretion protein SopE2
IY66_05955
hypothetical protein
IY66_05965
integrase
IY66_05970
exodeoxyribonuclease VIII
IY66_05985
lytic enzyme
Salmonella enterica subsp. enterica serovar Enteritidis strain OLF−SE8−1021710
IY66_05835
IY66_05840
membrane protein
IY66_05855
hypothetical protein
IY66_05820
IY66_05825
IY66_05850
integrase
Salmonella enterica subsp. enterica serovar Enteritidis strain OLF−SE8−1021710
AV95_06705
tail fiber assembly protein
AV95_06715
AV95_06745
hypothetical protein
AV95_06710
type III secretion protein SopE2
AV95_06720
hypothetical protein
AV95_06730
integrase
AV95_06735
exodeoxyribonuclease VIII
AV95_06750
lytic enzyme
Salmonella enterica subsp. enterica serovar Enteritidis str. EC20120927 genome−p.c.VAL54.69%−taxID1412617

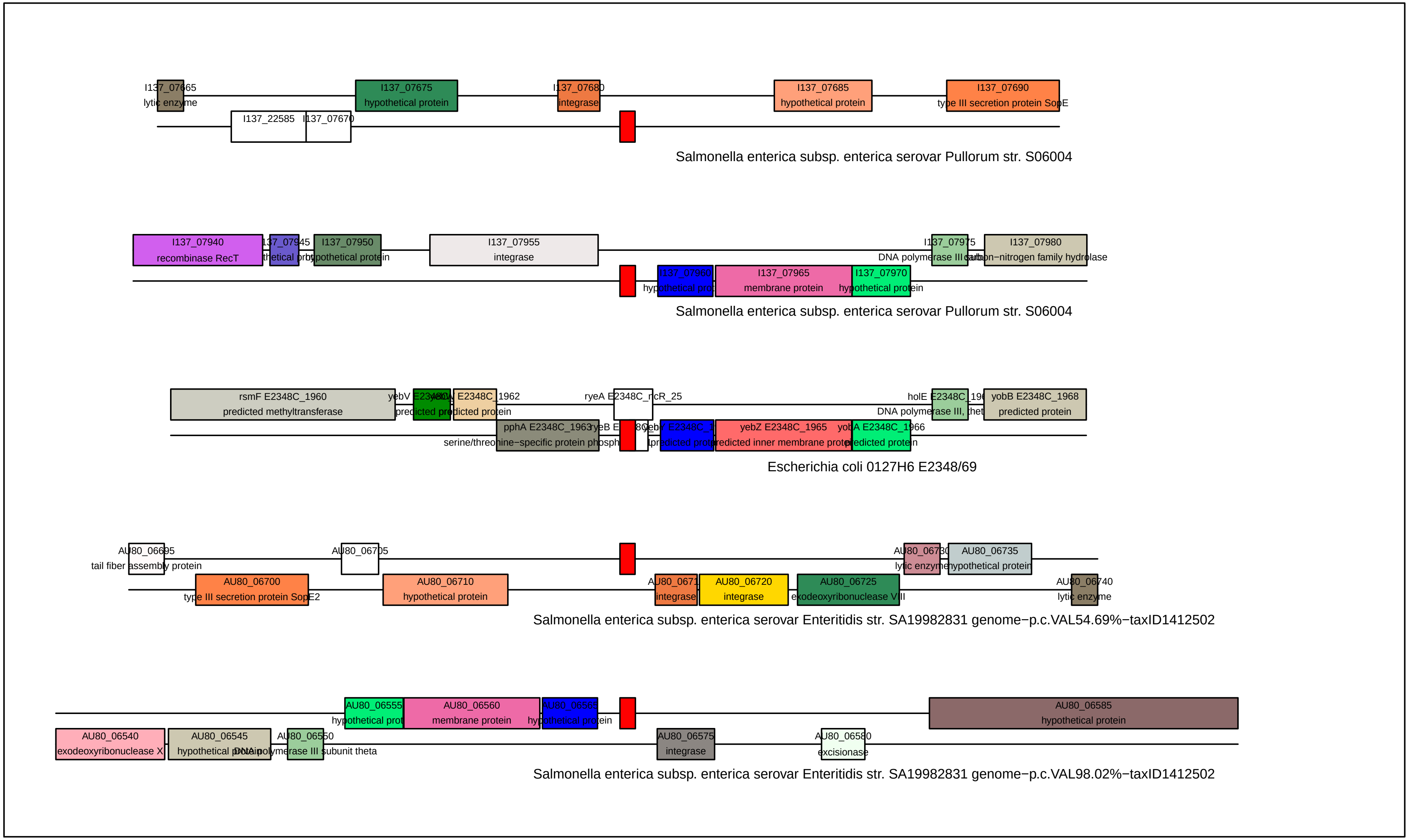

I137_07665
lytic enzyme
I137_07675
hypothetical protein
I137_07680
integrase
I137_07685
hypothetical protein
I137_07690
type III secretion protein SopE
I137_22585
I137_07670
Salmonella enterica subsp. enterica serovar Pullorum str. S06004
I137_07940
recombinase RecT
I137_07945
I137_07950
hypothetical protein
I137_07955
integrase
I137_07975
DNA polymerase III
I137_07980
carbon−nitrogen family hydrolase
I137_07965
membrane protein
I137_07970
hypothetical protein
Salmonella enterica subsp. enterica serovar Pullorum str. S06004
rsmF E2348C_1960
predicted methyltransferase
E2348C_1962
predicted protein
ryeA E2348C_ncR_25
holE
DNA polymerase III
yobB E2348C_1968
predicted protein
pphA E2348C_1963
yebZ E2348C_1965
predicted inner membrane protein
E2348C_1966
predicted protein
Escherichia coli 0127H6 E2348/69
AU80_06695
tail fiber assembly protein
AU80_06705
AU80_06730
lytic enzyme
AU80_06735
hypothetical protein
AU80_06700
type III secretion protein SopE2
AU80_06710
hypothetical protein
integrase
AU80_06720
integrase
AU80_06725
exodeoxyribonuclease VIII
AU80_06740
lytic enzyme
Salmonella enterica subsp. enterica serovar Enteritidis str. SA19982831 genome−p.c.VAL54.69%−taxID1412502
AU80_06555
AU80_06560
membrane protein
hypothetical protein
AU80_06585
hypothetical protein
AU80_06540
exodeoxyribonuclease X
AU80_06545
hypothetical protein
AU80_06550
DNA polymerase III subunit theta
AU80_06575
integrase
AU80_06580
excisionase
Salmonella enterica subsp. enterica serovar Enteritidis str. SA19982831 genome−p.c.VAL98.02%−taxID1412502

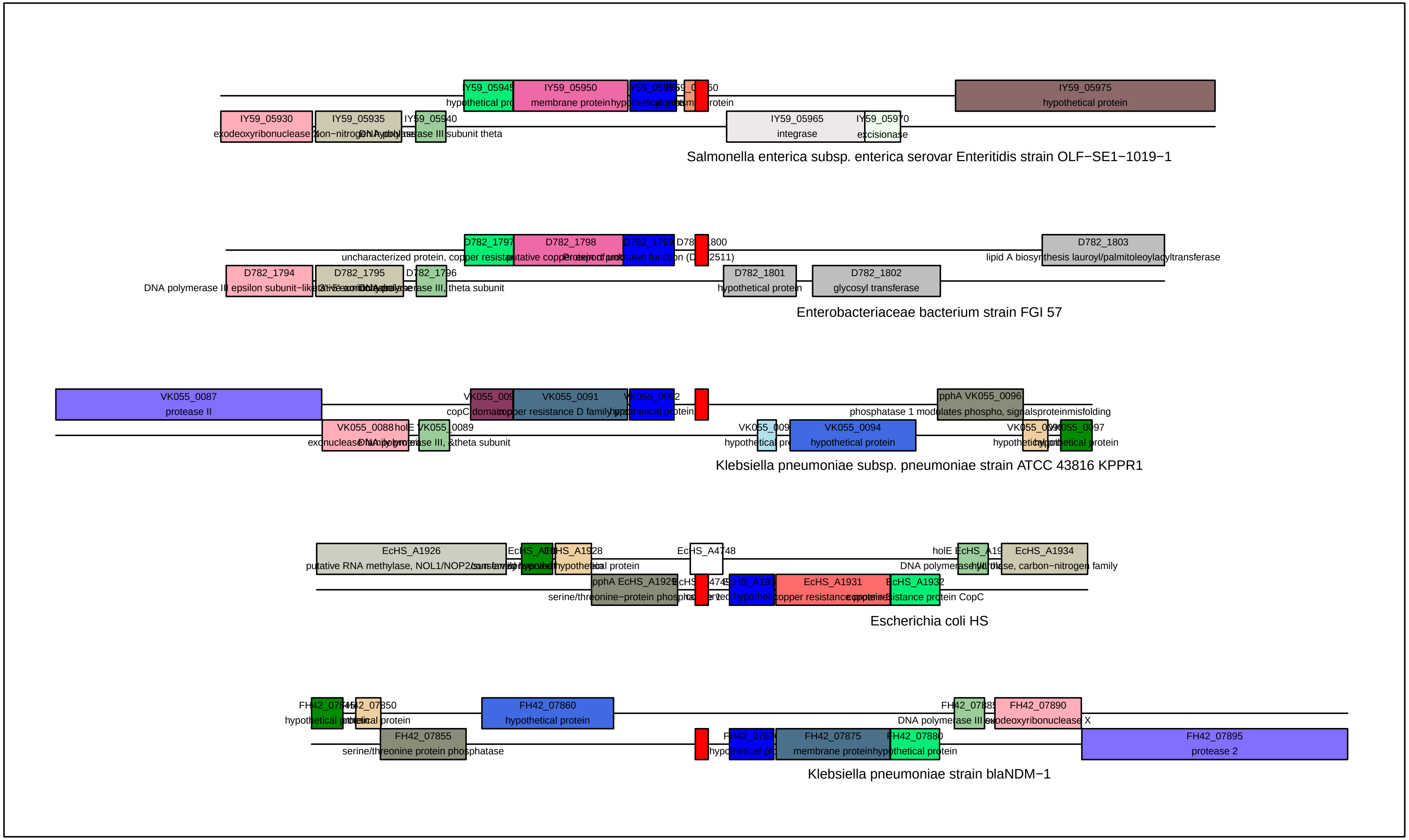
IY59_05945
IY59_05950
membrane protein
IY59_05975
hypothetical protein
IY59_05930
exodeoxyribonuclease X
IY59_05935
IY59_05940
subunit theta
IY59_05965
integrase
IY59_05970
excisionase
Salmonella enterica subsp. enterica serovar Enteritidis strain OLF−SE1−1019−1
D782_1797
D782_1798
uncharacterized protein, copper resistance
D782_1803
lipid A biosynthesis lauroyl/palmitoleoyl acyltransferase
D782_1794
D782_1795
D782_1796
DNA polymerase III, theta subunit
D782_1801
hypothetical protein
D782_1802
glycosyl transferase
Enterobacteriaceae bacterium strain FGI 57
VK055_0087
protease II
VK055_0091
copper resistance D family
hypothetical protein
pphA VK055_0096
phosphatase 1 modulates phospho, signals protein misfolding
VK055_0088
holE
VK055_0089
DNA polymerase III, theta subunit
VK055_0094
hypothetical protein
VK055_0097
hypothetical protein
Klebsiella pneumoniae subsp. pneumoniae strain ATCC 43816 KPPR1
EcHS_A1926
putative RNA methylase, NOL1/NOP2/sun family
EcHS_A1928
hypothetical protein
EcHS_A4748
holE
EcHS_A1934
DNA polymerase III
carbon−nitrogen family
pphA EcHS_A1929
serine/threonine−protein phosphatase
EcHS_A1931
copper resistance protein
EcHS_A1932
copper resistance protein CopC
Escherichia coli HS
FH42_07850
hypothetical protein
FH42_07860
hypothetical protein
FH42_07890
DNA polymerase III
exodeoxyribonuclease X
FH42_07855
serine/threonine protein phosphatase
FH42_07875
membrane protein
FH42_07880
hypothetical protein
FH42_07895
protease 2
Klebsiella pneumoniae strain blaNDM−1

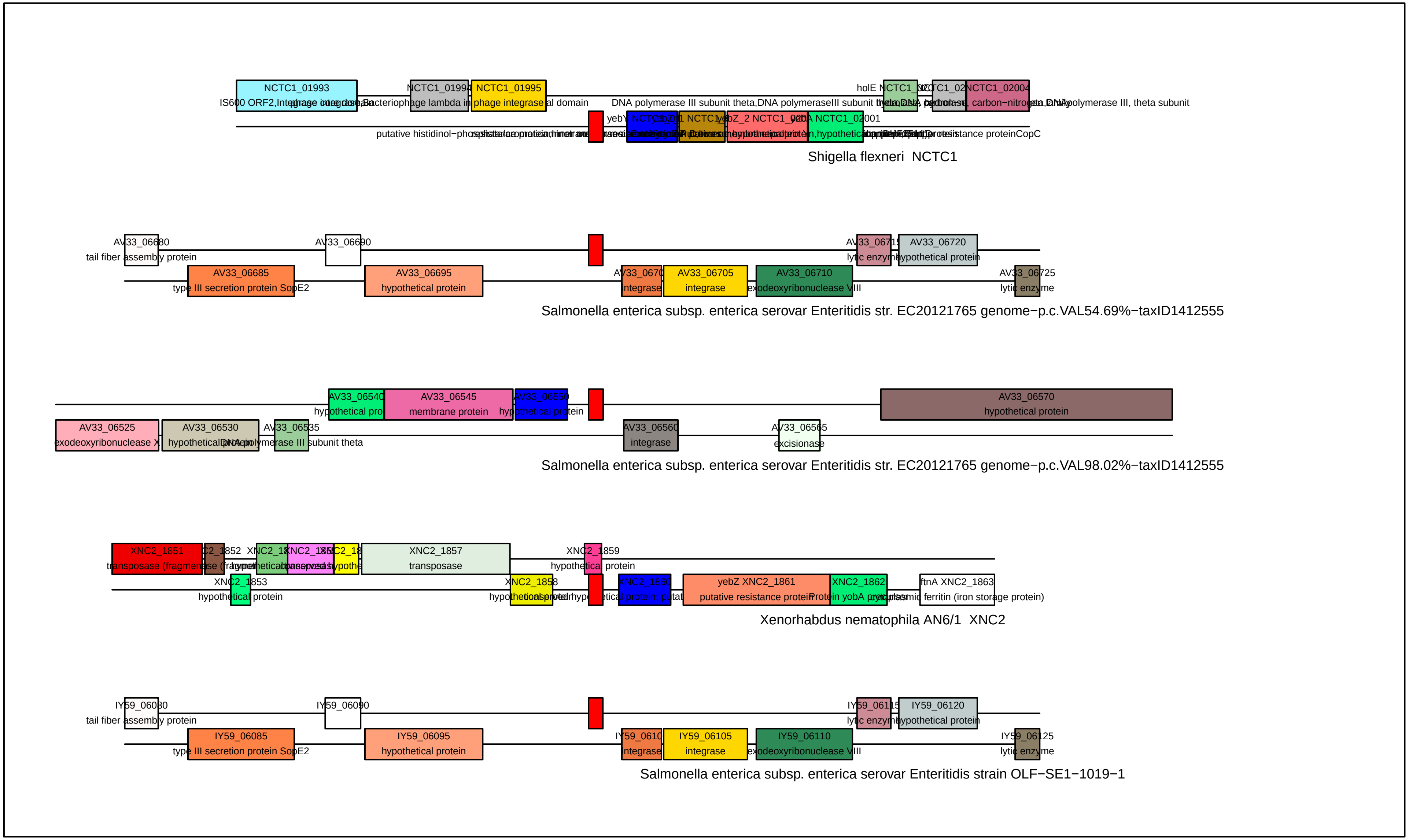

NCTC1_01993
IS600 ORF2
NCTC1_01995
phage integrase
DNA polymerase III subunit theta
holE
NCTC1_02004
putative histidinol−phosphatase
yebY
yebZ_2
NCTC1_02001
Shigella flexneri NCTC1
AV33_06680
tail fiber assembly protein
AV33_06690
AV33_06720
hypothetical protein
lytic enzyme
AV33_06685
type III secretion protein SopE2
AV33_06695
hypothetical protein
integrase
AV33_06705
integrase
AV33_06710
exodeoxyribonuclease VIII
AV33_06725
lytic enzyme
Salmonella enterica subsp. enterica serovar Enteritidis str. EC20121765 genome−p.c.VAL54.69%−taxID1412555
AV33_06540
AV33_06545
membrane protein
AV33_06550
hypothetical protein
AV33_06570
hypothetical protein
AV33_06525
exodeoxyribonuclease X
AV33_06530
AV33_06535
DNA polymerase III subunit theta
AV33_06560
integrase
AV33_06565
excisionase
Salmonella enterica subsp. enterica serovar Enteritidis str. EC20121765 genome−p.c.VAL98.02%−taxID1412555
XNC2_1851
XNC2_1857
transposase
XNC2_1859
hypothetical protein
XNC2_1853
hypothetical protein
XNC2_1858
yebZ XNC2_1861
putative resistance protein
XNC2_1862
ftnA XNC2_1863
ferritin (iron storage protein)
Xenorhabdus nematophila AN6/1 XNC2
IY59_06080
tail fiber assembly protein
IY59_06090
IY59_06120
hypothetical protein
lytic enzyme
IY59_06085
type III secretion protein SopE2
IY59_06095
hypothetical protein
integrase
IY59_06105
integrase
IY59_06110
exodeoxyribonuclease VIII
IY59_06125
lytic enzyme
Salmonella enterica subsp. enterica serovar Enteritidis strain OLF−SE1−1019−1

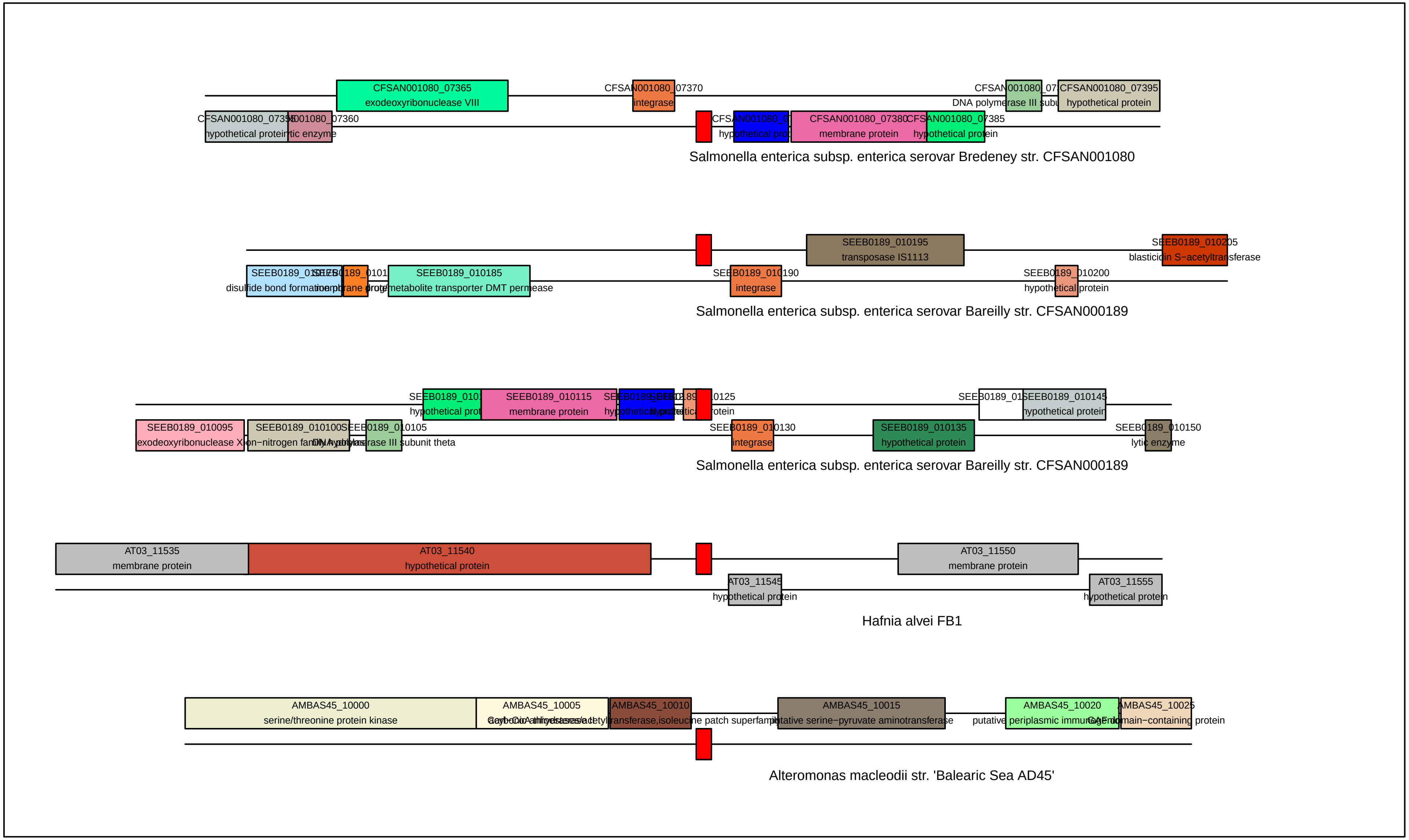

CFSAN001080_07365
exodeoxyribonuclease VIII
CFSAN001080_07395
hypothetical protein
CFSAN001080_07380
membrane protein
Salmonella enterica subsp. enterica serovar Bredeney str. CFSAN001080
SEEB0189_010195
transposase IS1113
SEEB0189_010185
metabolite transporter DMT permease
SEEB0189_010190
integrase
SEEB0189_010200
hypothetical protein
Salmonella enterica subsp. enterica serovar Bareilly str. CFSAN000189
SEEB0189_010115
membrane protein
SEEB0189_010095
exodeoxyribonuclease X
SEEB0189_010130
integrase
SEEB0189_010135
hypothetical protein
SEEB0189_010145
hypothetical protein
SEEB0189_010150
lytic enzyme
Salmonella enterica subsp. enterica serovar Bareilly str. CFSAN000189
AT03_11535
membrane protein
AT03_11540
hypothetical protein
AT03_11550
membrane protein
AT03_11545
hypothetical protein
AT03_11555
hypothetical protein
Hafnia alvei FB1
AMBAS45_10000
serine/threonine protein kinase
AMBAS45_10005
AMBAS45_10010
AMBAS45_10015
putative serine−pyruvate aminotransferase
AMBAS45_10020
AMBAS45_10025
Alteromonas macleodii str. 'Balearic Sea AD45'

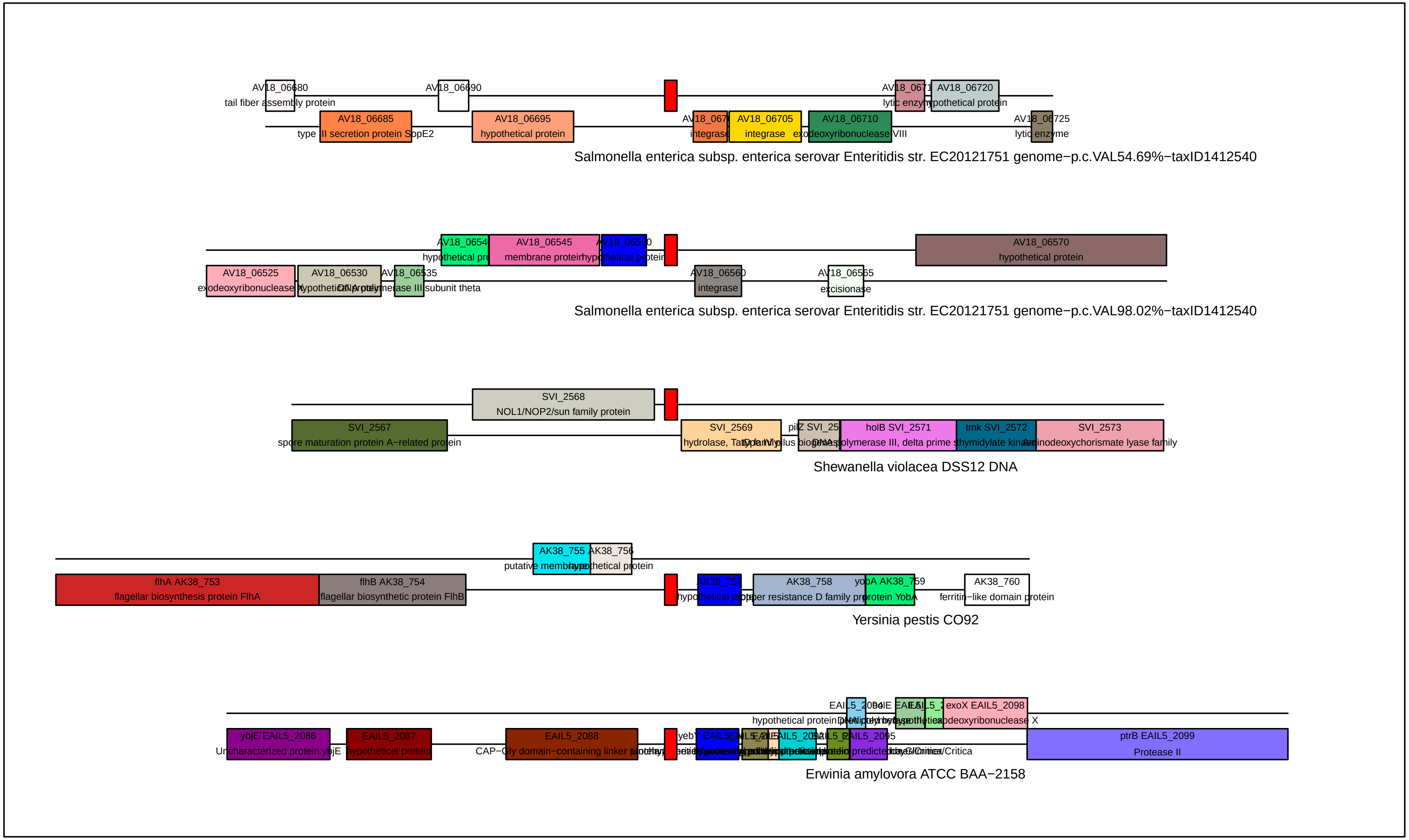

AV18_06680
tail fiber assembly protein
AV18_06690
AV18_06720
hypothetical protein
AV18_06685
type III secretion protein SopE2
AV18_06695
hypothetical protein
AV18_06705
integrase
AV18_06710
exodeoxyribonuclease VIII
AV18_06725
lytic enzyme
Salmonella enterica subsp. enterica serovar Enteritidis str. EC20121751 genome−p.c.VAL54.69%−taxID1412540
AV18_06545
AV18_06570
hypothetical protein
AV18_06525
exodeoxyribonuclease X
AV18_06530
AV18_06535
DNA polymerase III subunit theta
AV18_06560
integrase
AV18_06565
excisionase
Salmonella enterica subsp. enterica serovar Enteritidis str. EC20121751 genome−p.c.VAL98.02%−taxID1412540
SVI_2568
NOL1/NOP2/sun family protein
SVI_2567
spore maturation protein A−related protein
SVI_2569
holB SVI_2571
tmk SVI_2572
thymidylate kinase
SVI_2573
aminodeoxychorismate lyase family
Shewanella violacea DSS12 DNA
AK38_755
AK38_756
hypothetical protein
flhA AK38_753
flagellar biosynthesis protein FlhA
flhB AK38_754
flagellar biosynthetic protein FlhB
AK38_758
yobA AK38_759
AK38_760
ferritin−like domain protein
Yersinia pestis CO92
exoX EAIL5_2098
ybjE EAIL5_2086
Uncharacterized protein ybjE
EAIL5_2087
hypothetical protein
EAIL5_2088
CAP−Gly domain−containing linker protein
ptrB EAIL5_2099
Protease II
Erwinia amylovora ATCC BAA−2158

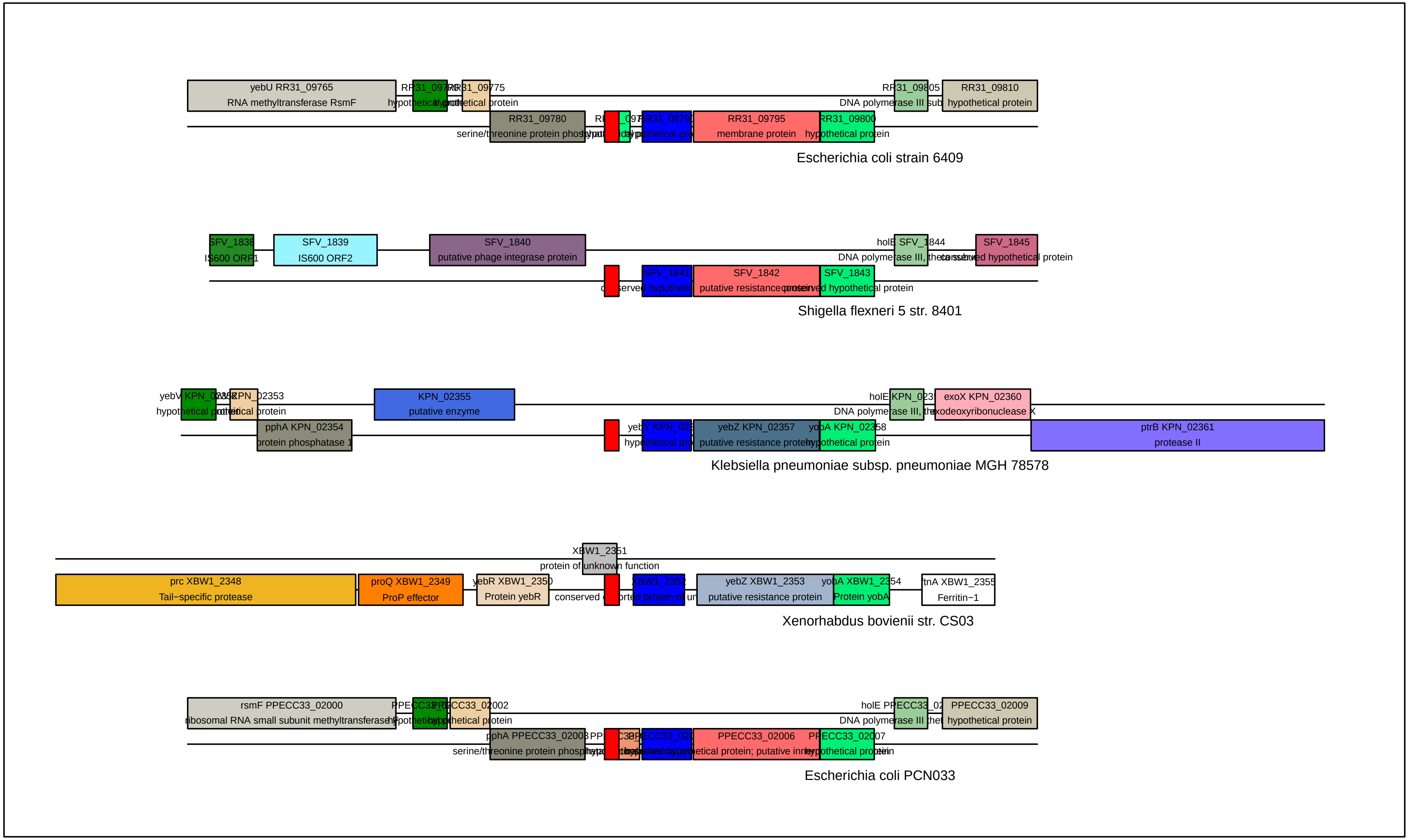

yebU RR31_09765
RNA methyltransferase RsmF
RR31_09775
RR31_09780
serine/threonine protein phosp
RR31_09795
membrane protein
RR31_09800
hypothetical protein
RR31_09805
DNA polymerase III
RR31_09810
hypothetical protein
Escherichia coli strain 6409
SFV_1839
IS600 ORF2
SFV_1840
putative phage integrase protein
SFV_1842
putative resistance protein
SFV_1843
conserved hypothetical protein
holE SFV_1844
SFV_1845
Shigella flexneri 5 str. 8401
KPN_02353
KPN_02355
putative enzyme
pphA KPN_02354
protein phosphatase 1
yebZ KPN_02357
putative resistance protein
KPN_02358
hypothetical protein
exoX KPN_02360
exodeoxyribonuclease X
ptrB KPN_02361
protease II
Klebsiella pneumoniae subsp. pneumoniae MGH 78578
XBW1_2351
protein of unknown function
prc XBW1_2348
Tail−specific protease
proQ XBW1_2349
ProP effector
yebR XBW1_2350
Protein yebR
yebZ XBW1_2353
putative resistance protein
yobA XBW1_2354
Protein yobA
ftnA XBW1_2355
Ferritin−1
Xenorhabdus bovienii str. CS03
rsmF PPECC33_02000
ribosomal RNA small subunit methyltransferase F
PPECC33_02002
pphA PPECC33_02003
serine/threonine protein phosphatase
PPECC33_02006
PPECC33_02007
hypothetical protein
PPECC33_02009
hypothetical protein
DNA polymerase III
Escherichia coli PCN033

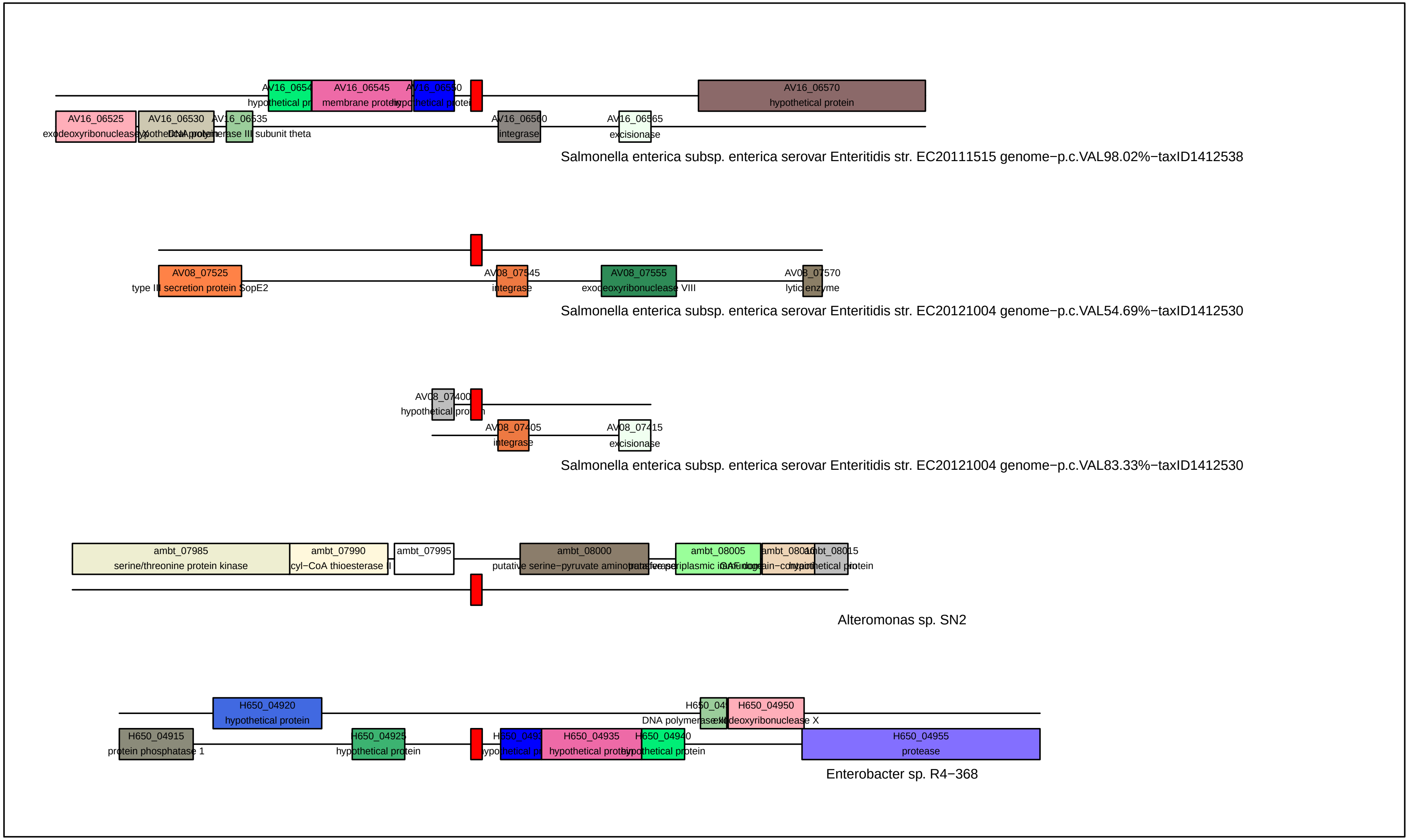
AV16_06545
membrane protein
AV16_06570
hypothetical protein
AV16_06525
exodeoxyribonuclease X
AV16_06530
AV16_06560
integrase
AV16_06565
excisionase
Salmonella enterica subsp. enterica serovar Enteritidis str. EC20111515 genome−p.c.VAL98.02%−taxID1412538
AV08_07525
type III secretion protein SopE2
AV08_07545
integrase
AV08_07555
exodeoxyribonuclease VIII
AV08_07570
lytic enzyme
Salmonella enterica subsp. enterica serovar Enteritidis str. EC20121004 genome−p.c.VAL54.69%−taxID1412530
AV08_07400
AV08_07405
integrase
AV08_07415
excisionase
Salmonella enterica subsp. enterica serovar Enteritidis str. EC20121004 genome−p.c.VAL83.33%−taxID1412530
ambt_07985
serine/threonine protein kinase
ambt_07990
ambt_07995
ambt_08000
ambt_08005
ambt_08015
Alteromonas sp. SN2
H650_04920
hypothetical protein
H650_04950
H650_04915
protein phosphatase 1
H650_04925
hypothetical protein
H650_04935
H650_04940
H650_04955
protease
Enterobacter sp. R4−368

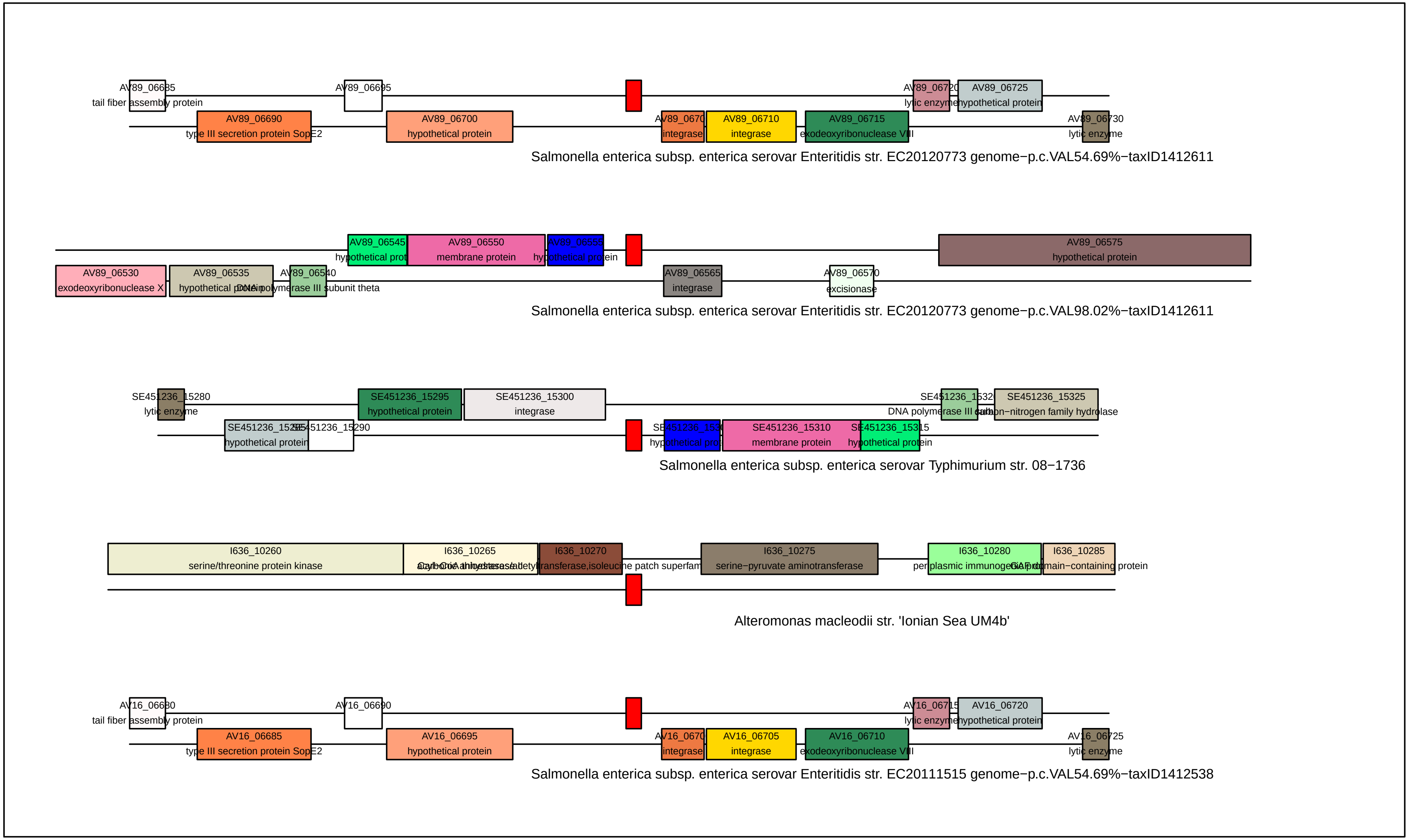

AV89_06685
tail fiber assembly protein
AV89_06695
AV89_06720
lytic enzyme
AV89_06725
hypothetical protein
AV89_06690
type III secretion protein SopE2
AV89_06700
hypothetical protein
integrase
AV89_06710
integrase
AV89_06715
exodeoxyribonuclease VIII
AV89_06730
lytic enzyme
Salmonella enterica subsp. enterica serovar Enteritidis str. EC20120773 genome−p.c.VAL54.69%−taxID1412611
AV89_06545
AV89_06550
membrane protein
hypothetical protein
AV89_06575
hypothetical protein
AV89_06530
exodeoxyribonuclease X
AV89_06535
AV89_06540
DNA polymerase III subunit theta
AV89_06565
integrase
AV89_06570
excisionase
Salmonella enterica subsp. enterica serovar Enteritidis str. EC20120773 genome−p.c.VAL98.02%−taxID1412611
SE451236_15280
lytic enzyme
SE451236_15295
hypothetical protein
SE451236_15300
integrase
DNA polymerase III
SE451236_15325
hypothetical protein
SE451236_15290
SE451236_15310
membrane protein
SE451236_15315
hypothetical protein
Salmonella enterica subsp. enterica serovar Typhimurium str. 08−1736
I636_10260
serine/threonine protein kinase
I636_10265
I636_10270
I636_10275
serine−pyruvate aminotransferase
I636_10280
I636_10285
Alteromonas macleodii str. 'Ionian Sea UM4b'
AV16_06680
tail fiber assembly protein
AV16_06690
lytic enzyme
AV16_06720
hypothetical protein
AV16_06685
type III secretion protein SopE2
AV16_06695
hypothetical protein
integrase
AV16_06705
integrase
AV16_06710
exodeoxyribonuclease VIII
lytic enzyme
Salmonella enterica subsp. enterica serovar Enteritidis str. EC20111515 genome−p.c.VAL54.69%−taxID1412538

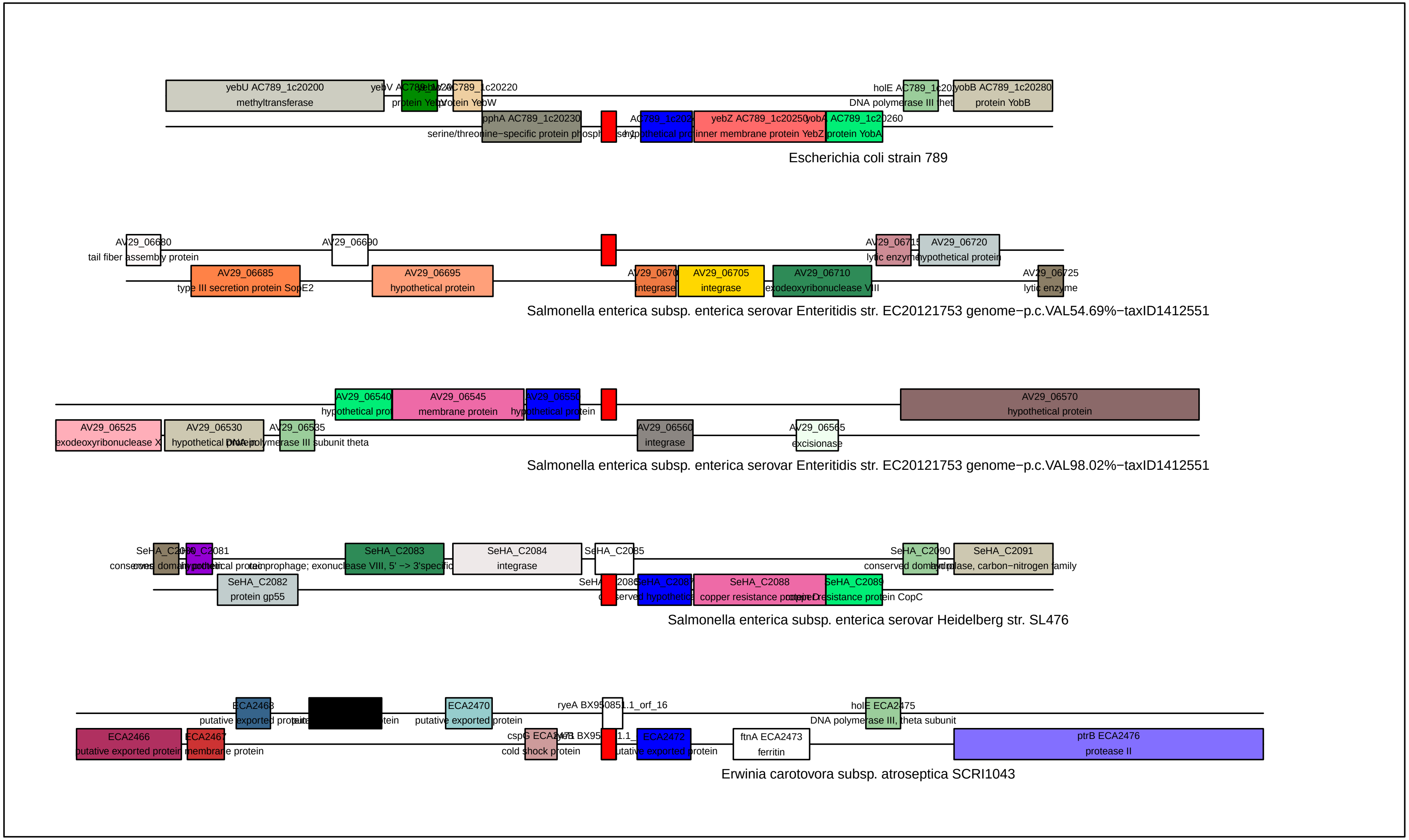

yebU AC789_1c20200
methyltransferase
holE
DNA polymerase III
yobB AC789_1c20280
protein YobB
serine/threonine−specific protein phosph
yebZ AC789_1c20250
inner membrane protein YebZ
protein YobA
Escherichia coli strain 789
AV29_06680
tail fiber assembly protein
AV29_06690
AV29_06720
hypothetical protein
AV29_06685
type III secretion protein SopE2
AV29_06695
hypothetical protein
AV29_06705
integrase
AV29_06710
exodeoxyribonuclease VIII
AV29_06725
lytic enzyme
Salmonella enterica subsp. enterica serovar Enteritidis str. EC20121753 genome−p.c.VAL54.69%−taxID1412551
AV29_06545
membrane protein
AV29_06570
hypothetical protein
AV29_06525
exodeoxyribonuclease X
AV29_06530
AV29_06560
integrase
AV29_06565
excisionase
Salmonella enterica subsp. enterica serovar Enteritidis str. EC20121753 genome−p.c.VAL98.02%−taxID1412551
SeHA_C2083
SeHA_C2084
integrase
SeHA_C2085
SeHA_C2091
SeHA_C2082
protein gp55
SeHA_C2088
SeHA_C2089
Salmonella enterica subsp. enterica serovar Heidelberg str. SL476
ECA2470
putative exported protein
ryeA BX950851.1_orf_16
holE ECA2475
DNA polymerase III, theta subunit
ECA2466
putative exported protein
ECA2467
membrane protein
cold shock protein
ECA2472
ftnA ECA2473
ferritin
ptrB ECA2476
protease II
Erwinia carotovora subsp. atroseptica SCRI1043

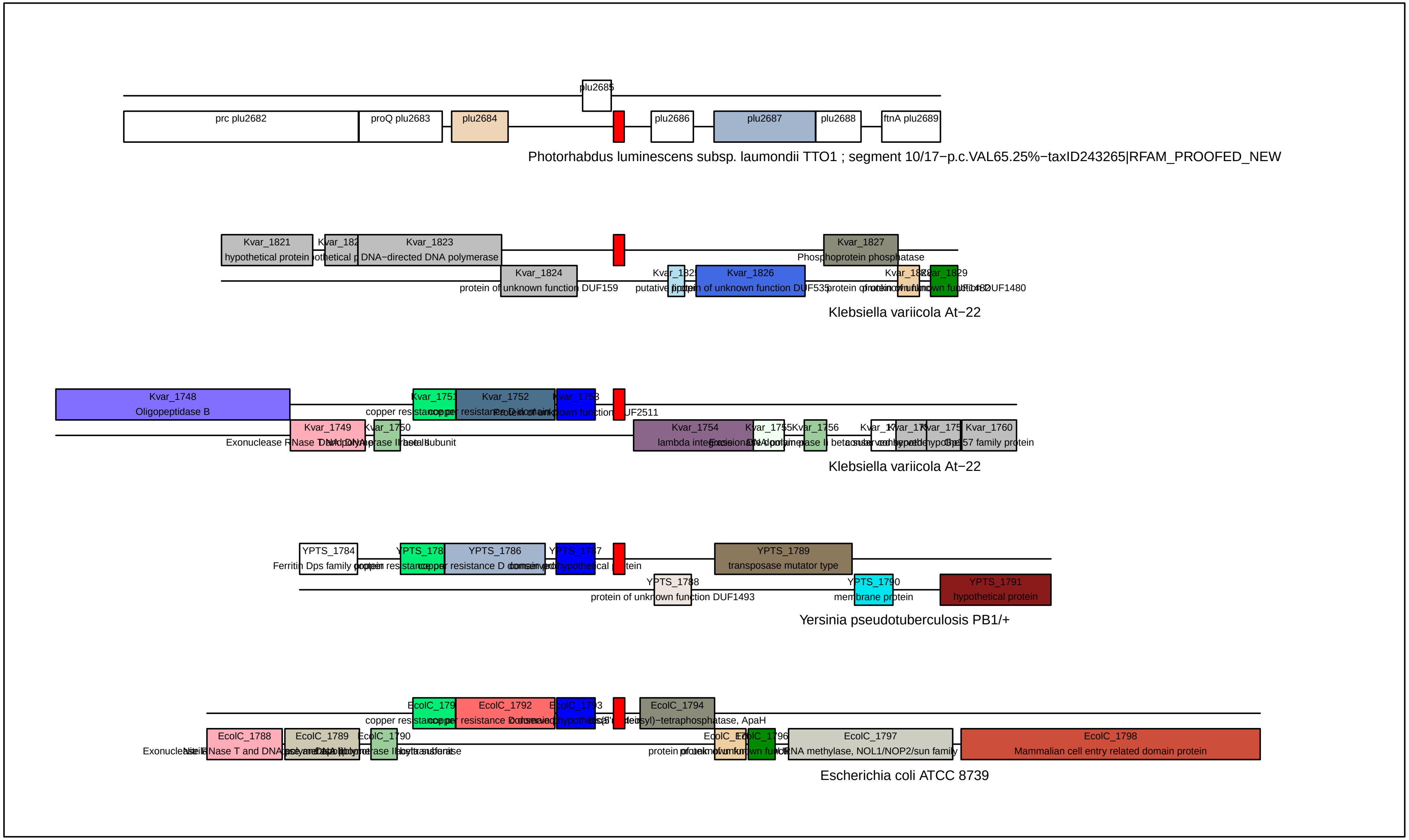

plu2685
prc plu2682
proQ plu2683
plu2684
plu2686
plu2687
plu2688
ftnA plu2689
Photorhabdus luminescens subsp. laumondii TTO1 ; segment 10/17−p.c.VAL65.25%−taxID243265|RFAM_PROOFED_NEW
Kvar_1821
hypothetical protein
Kvar_1823
DNA−directed DNA polymerase
Kvar_1827
Phosphoprotein phosphatase
Kvar_1824
protein of unknown function DUF159
Kvar_1826
protein of unknown function DUF535
Klebsiella variicola At−22
Kvar_1748
Oligopeptidase B
Kvar_1752
Kvar_1749
Kvar_1754
Kvar_1760
Klebsiella variicola At−22
YPTS_1784
YPTS_1786
YPTS_1789
transposase mutator type
YPTS_1788
protein of unknown function DUF1493
YPTS_1790
membrane protein
YPTS_1791
hypothetical protein
Yersinia pseudotuberculosis PB1/+
EcolC_1792
EcolC_1794
EcolC_1788
EcolC_1789
EcolC_1797
RNA methylase, NOL1/NOP2/sun family
EcolC_1798
Mammalian cell entry related domain protein
Escherichia coli ATCC 8739

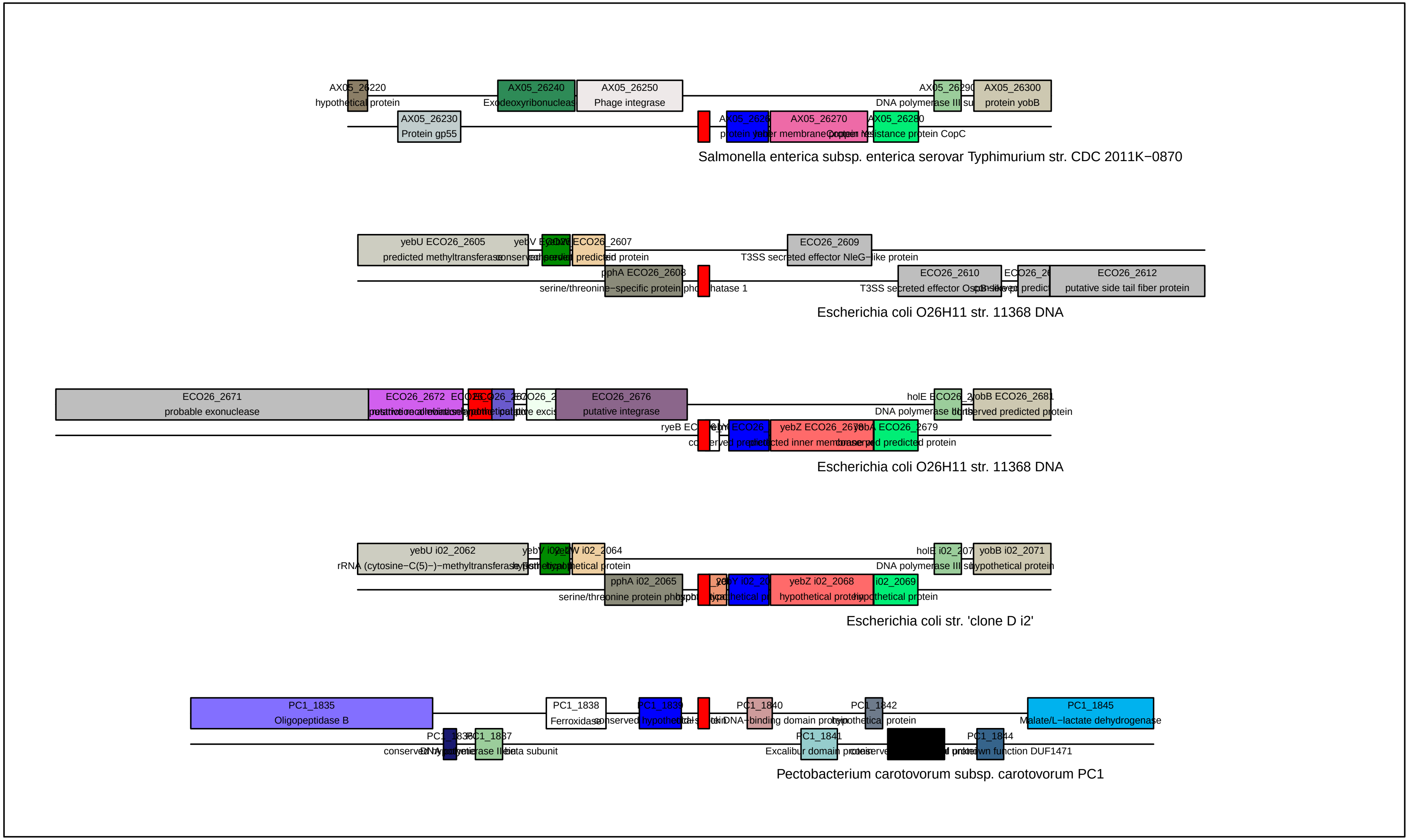

AX05_26220
hypothetical protein
AX05_26240
AX05_26250
Phage integrase
AX05_26300
protein yobB
AX05_26230
Protein gp55
AX05_26270
Salmonella enterica subsp. enterica serovar Typhimurium str. CDC 2011K−0870
yebU ECO26_2605
predicted methyltransferase
ECO26_2609
T3SS secreted effector NleG−like protein
pphA ECO26_2608
serine/threonine−specific protein phosphatase 1
ECO26_2610
ECO26_2612
putative side tail fiber protein
Escherichia coli O26H11 str. 11368 DNA
ECO26_2671
probable exonuclease
ECO26_2672
ECO26_2676
putative integrase
Escherichia coli O26H11 str. 11368 DNA
yebU i02_2062
rRNA (cytosine−C(5)−)−methyltransferase
yobB i02_2071
hypothetical protein
pphA i02_2065
yebZ i02_2068
Escherichia coli str. 'clone D i2'
PC1_1835
Oligopeptidase B
PC1_1838
Ferroxidase
PC1_1840
PC1_1842
hypothetical protein
PC1_1845
Malate/L−lactate dehydrogenase
PC1_1837
PC1_1841
Excalibur domain protein
PC1_1844
Pectobacterium carotovorum subsp. carotovorum PC1

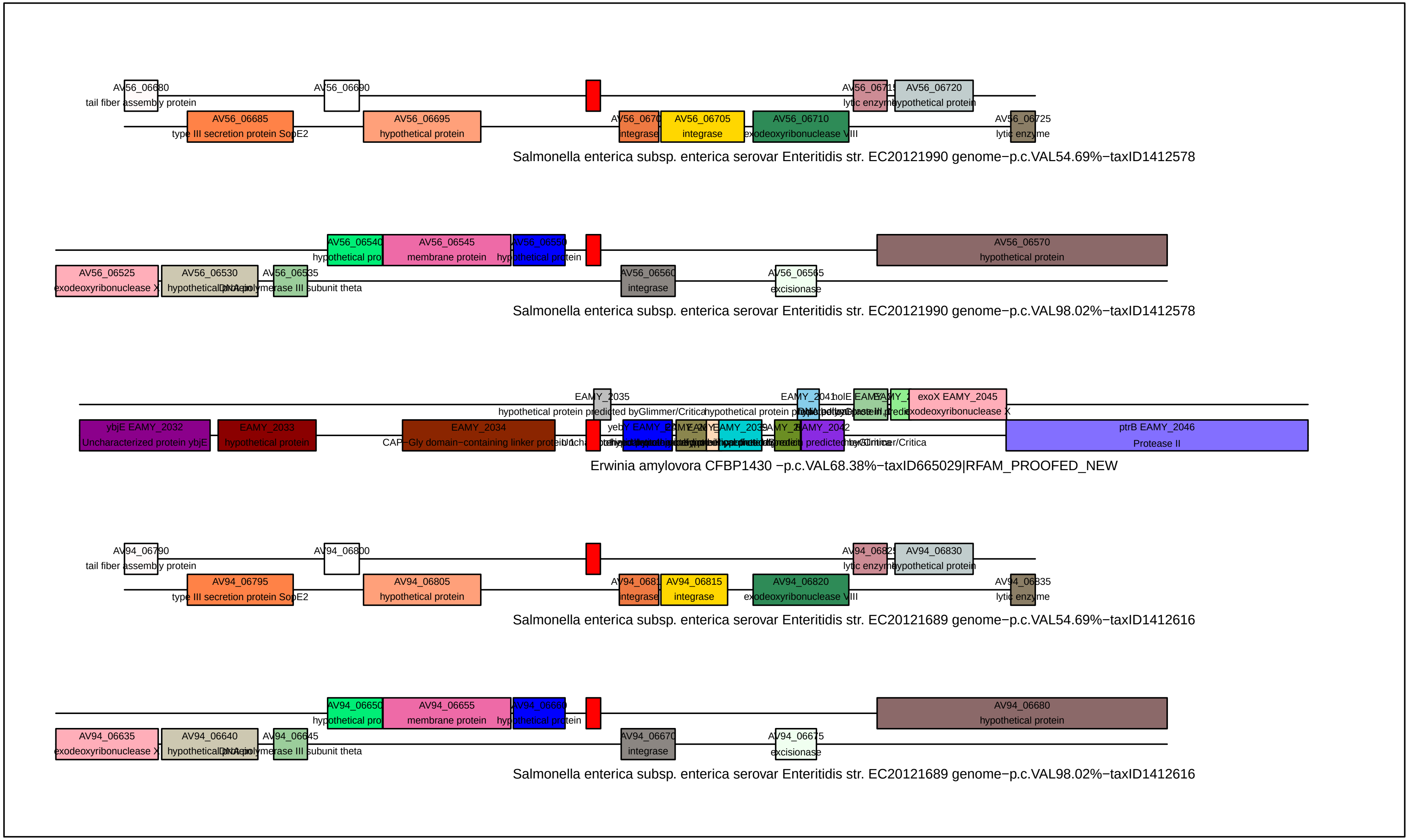
AV56_06680
tail fiber assembly protein
AV56_06690
AV56_06685
type III secretion protein SopE2
AV56_06695
hypothetical protein
integrase
AV56_06705
integrase
AV56_06710
exodeoxyribonuclease VIII
lytic enzyme
AV56_06720
hypothetical protein
AV56_06725
lytic enzyme
Salmonella enterica subsp. enterica serovar Enteritidis str. EC20121990 genome−p.c.VAL54.69%−taxID1412578
AV56_06540
AV56_06545
membrane protein
AV56_06570
hypothetical protein
AV56_06525
exodeoxyribonuclease X
AV56_06530
AV56_06535
AV56_06560
integrase
AV56_06565
excisionase
Salmonella enterica subsp. enterica serovar Enteritidis str. EC20121990 genome−p.c.VAL98.02%−taxID1412578
EAMY_2035
exoX EAMY_2045
exodeoxyribonuclease X
ybjE EAMY_2032
Uncharacterized protein ybjE
EAMY_2033
hypothetical protein
EAMY_2034
ptrB EAMY_2046
Protease II
Erwinia amylovora CFBP1430 −p.c.VAL68.38%−taxID665029|RFAM_PROOFED_NEW
AV94_06790
tail fiber assembly protein
AV94_06800
AV94_06795
type III secretion protein SopE2
AV94_06805
hypothetical protein
integrase
AV94_06815
integrase
AV94_06820
exodeoxyribonuclease VIII
lytic enzyme
AV94_06830
hypothetical protein
lytic enzyme
Salmonella enterica subsp. enterica serovar Enteritidis str. EC20121689 genome−p.c.VAL54.69%−taxID1412616
AV94_06650
AV94_06655
membrane protein
AV94_06680
hypothetical protein
AV94_06635
exodeoxyribonuclease X
AV94_06640
AV94_06645
AV94_06670
integrase
AV94_06675
excisionase
Salmonella enterica subsp. enterica serovar Enteritidis str. EC20121689 genome−p.c.VAL98.02%−taxID1412616

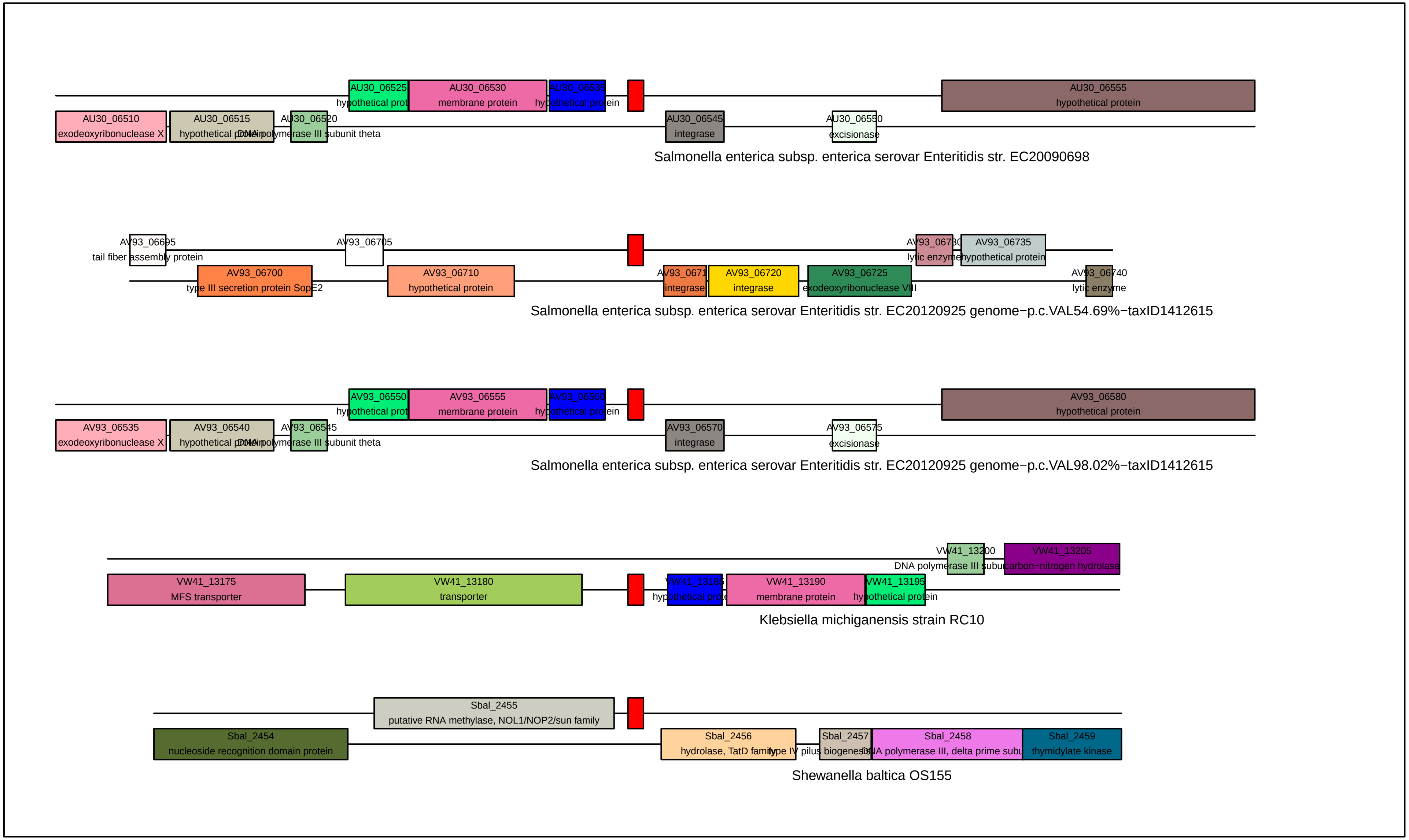

AU30_06525
AU30_06530
membrane protein
AU30_06555
hypothetical protein
AU30_06510
exodeoxyribonuclease X
AU30_06515
AU30_06545
integrase
excisionase
Salmonella enterica subsp. enterica serovar Enteritidis str. EC20090698
tail fiber assembly protein
AV93_06700
AV93_06710
hypothetical protein
AV93_06735
hypothetical protein
AV93_06720
integrase
AV93_06725
lytic enzyme
Salmonella enterica subsp. enterica serovar Enteritidis str. EC20120925 genome−p.c.VAL54.69%−taxID1412615
AV93_06550
AV93_06555
membrane protein
AV93_06580
hypothetical protein
AV93_06535
exodeoxyribonuclease X
AV93_06540
AV93_06570
integrase
excisionase
Salmonella enterica subsp. enterica serovar Enteritidis str. EC20120925 genome−p.c.VAL98.02%−taxID1412615
VW41_13205
VW41_13175
MFS transporter
VW41_13180
transporter
VW41_13190
membrane protein
VW41_13195
Klebsiella michiganensis strain RC10
Sbal_2455
putative RNA methylase, NOL1/NOP2/sun family
Sbal_2454
nucleoside recognition domain protein
Sbal_2456
Sbal_2457
Sbal_2458
Sbal_2459
thymidylate kinase
Shewanella baltica OS155

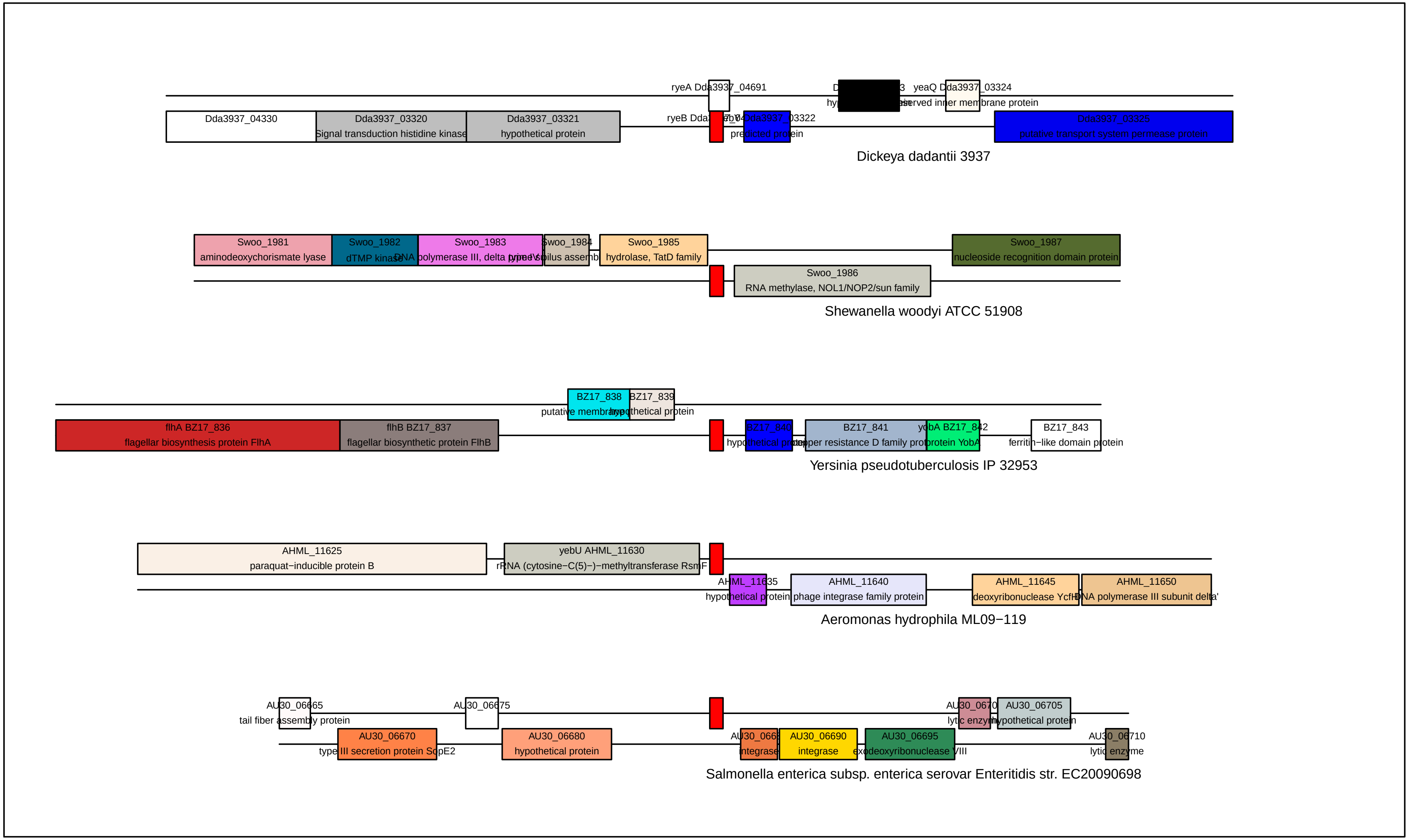
ryeA Dda3937_04691
yeaQ Dda3937_03324
conserved inner membrane protein
Dda3937_04330
Dda3937_03320
Signal transduction histidine kinase
Dda3937_03321
hypothetical protein
ryeB
Dda3937_03322
predicted protein
Dda3937_03325
putative transport system permease protein
Dickeya dadantii 3937
Swoo_1981
aminodeoxychorismate lyase
Swoo_1982
dTMP kinase
Swoo_1983
DNA polymerase III, delta prime
Swoo_1984
Swoo_1985
hydrolase, TatD family
Swoo_1987
nucleoside recognition domain protein
Swoo_1986
RNA methylase, NOL1/NOP2/sun family
Shewanella woodyi ATCC 51908
BZ17_838
putative membrane protein
BZ17_839
hypothetical protein
flhA BZ17_836
flagellar biosynthesis protein FlhA
flhB BZ17_837
flagellar biosynthetic protein FlhB
BZ17_840
hypothetical protein
BZ17_841
copper resistance D family protein
yobA BZ17_842
protein YobA
BZ17_843
ferritin−like domain protein
Yersinia pseudotuberculosis IP 32953
AHML_11625
paraquat−inducible protein B
yebU AHML_11630
rRNA (cytosine−C(5)−)−methyltransferase RsmF
AHML_11635
hypothetical protein
AHML_11640
phage integrase family protein
AHML_11645
deoxyribonuclease YcfH
AHML_11650
DNA polymerase III subunit delta'
Aeromonas hydrophila ML09−119
AU30_06665
tail fiber assembly protein
AU30_06675
AU30_06705
hypothetical protein
lytic enzyme
AU30_06670
type III secretion protein SopE2
AU30_06680
hypothetical protein
integrase
AU30_06690
integrase
AU30_06695
exodeoxyribonuclease VIII
AU30_06710
lytic enzyme
Salmonella enterica subsp. enterica serovar Enteritidis str. EC20090698

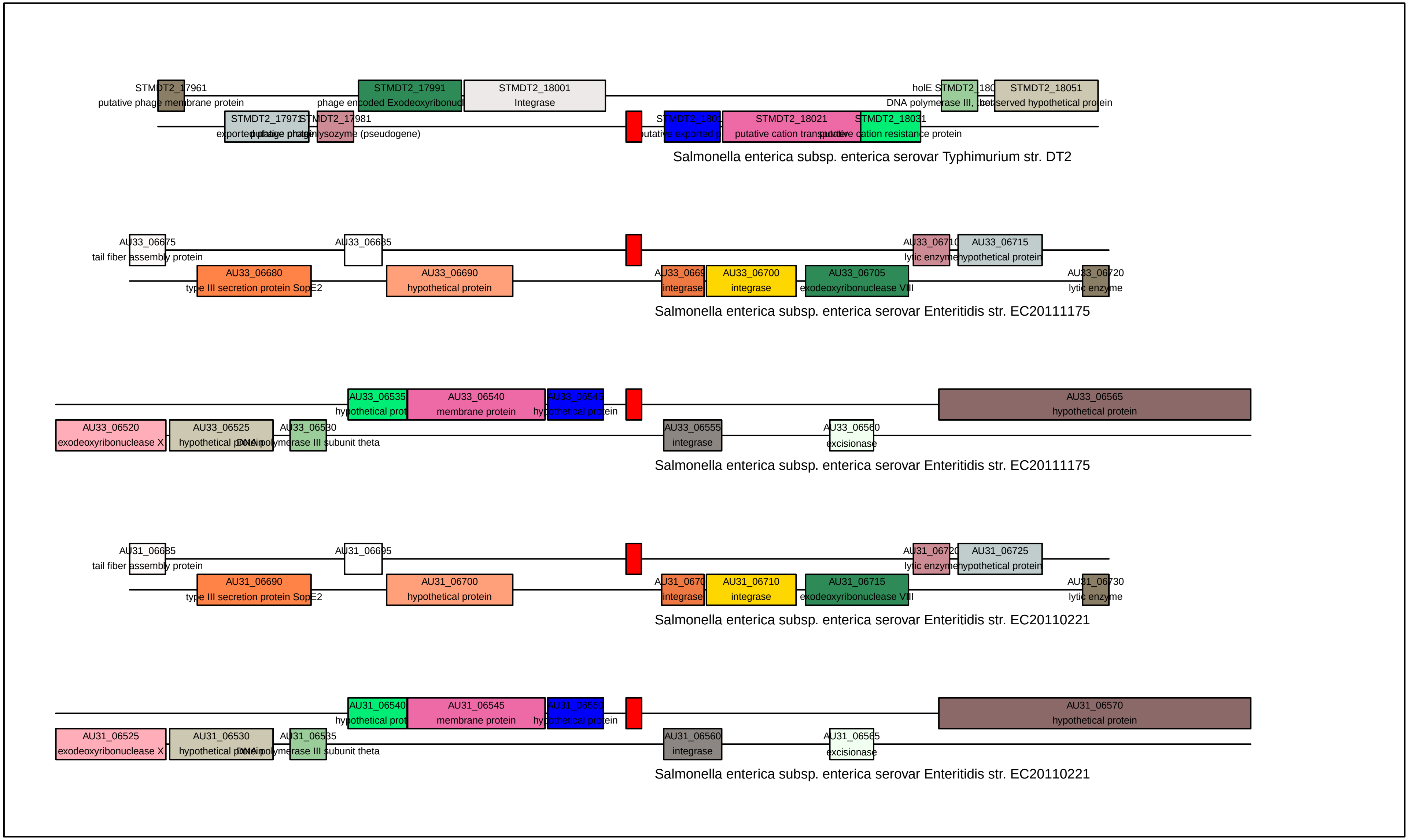

STMDT2_17961
putative phage membrane protein
STMDT2_17991
phage encoded Exodeoxyribonucl
STMDT2_18001
Integrase
holE
DNA polymerase III,
STMDT2_18051
conserved hypothetical protein
STMDT2_17971
STMDT2_17981
lysozyme (pseudogene)
putative exported
STMDT2_18021
putative cation transp
STMDT2_18031
putative cation resistance protein
Salmonella enterica subsp. enterica serovar Typhimurium str. DT2
AU33_06675
tail fiber assembly protein
AU33_06685
AU33_06715
hypothetical protein
lytic enzyme
AU33_06680
type III secretion protein SopE2
AU33_06690
hypothetical protein
integrase
AU33_06700
integrase
AU33_06705
exodeoxyribonuclease VIII
AU33_06720
lytic enzyme
Salmonella enterica subsp. enterica serovar Enteritidis str. EC20111175
AU33_06535
hypothetical prot
AU33_06540
membrane protein
hypothetical protein
AU33_06565
hypothetical protein
AU33_06520
exodeoxyribonuclease X
AU33_06525
hypothetical protein
AU33_06530
DNA polymerase III subunit theta
AU33_06555
integrase
AU33_06560
excisionase
Salmonella enterica subsp. enterica serovar Enteritidis str. EC20111175
AU31_06685
tail fiber assembly protein
AU31_06695
lytic enzyme
AU31_06725
hypothetical protein
AU31_06690
type III secretion protein SopE2
AU31_06700
hypothetical protein
integrase
AU31_06710
integrase
AU31_06715
exodeoxyribonuclease VIII
AU31_06730
lytic enzyme
Salmonella enterica subsp. enterica serovar Enteritidis str. EC20110221
AU31_06540
hypothetical prot
AU31_06545
membrane protein
hypothetical protein
AU31_06570
hypothetical protein
AU31_06525
exodeoxyribonuclease X
AU31_06530
hypothetical protein
AU31_06535
DNA polymerase III subunit theta
AU31_06560
integrase
AU31_06565
excisionase
Salmonella enterica subsp. enterica serovar Enteritidis str. EC20110221

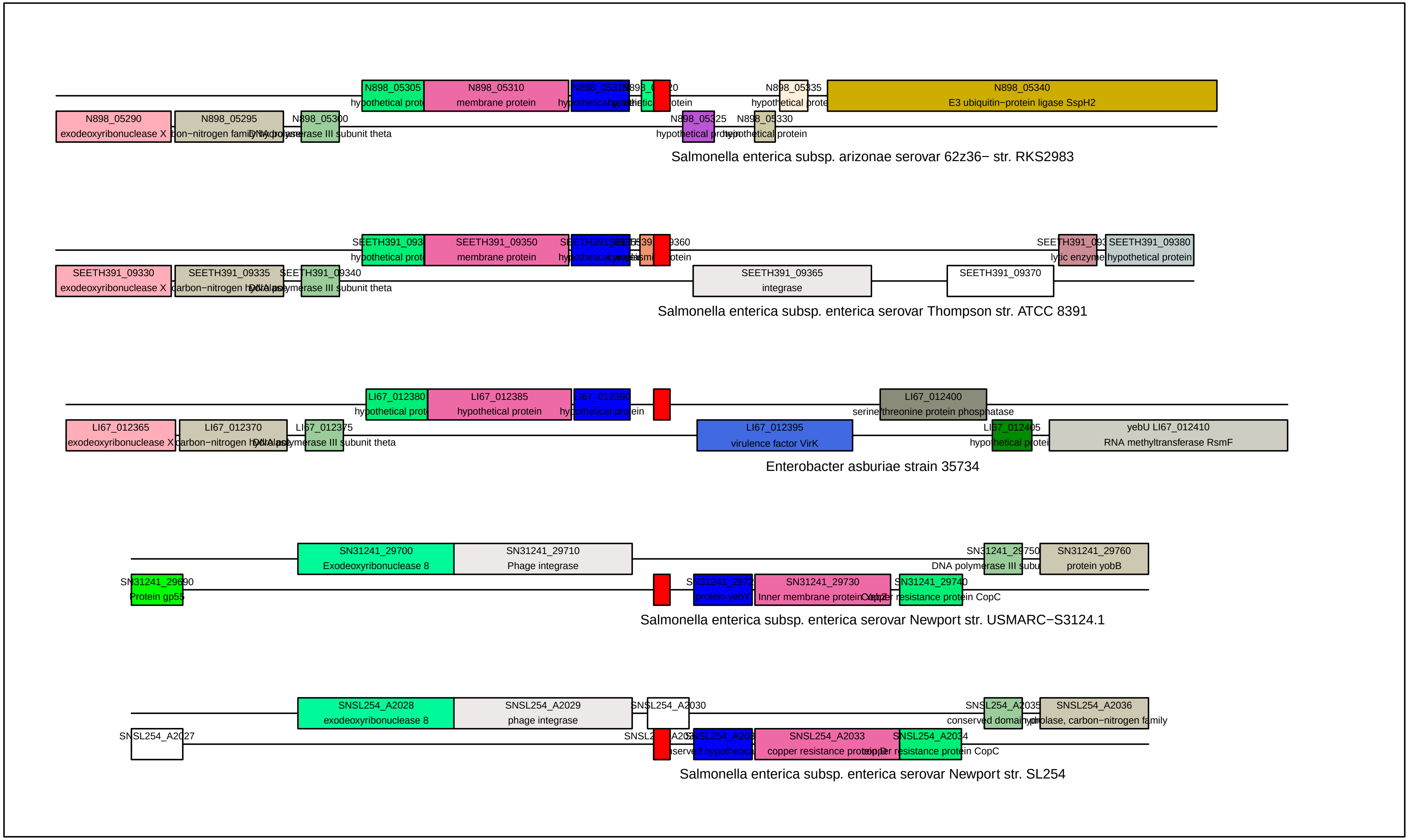
N898_05305
N898_05310
membrane protein
N898_05340
E3 ubiquitin−protein ligase SspH2
N898_05290
exodeoxyribonuclease X
N898_05295
N898_05300
Salmonella enterica subsp. arizonae serovar 62z36− str. RKS2983
SEETH391_09350
membrane protein
SEETH391_09380
hypothetical protein
SEETH391_09330
exodeoxyribonuclease X
SEETH391_09335
SEETH391_09365
integrase
SEETH391_09370
Salmonella enterica subsp. enterica serovar Thompson str. ATCC 8391
LI67_012380
LI67_012385
hypothetical protein
LI67_012400
serine/threonine protein phosphatase
LI67_012365
exodeoxyribonuclease X
LI67_012370
LI67_012395
virulence factor VirK
yebU LI67_012410
RNA methyltransferase RsmF
Enterobacter asburiae strain 35734
SN31241_29700
Exodeoxyribonuclease 8
SN31241_29710
Phage integrase
SN31241_29760
protein yobB
SN31241_29730
Salmonella enterica subsp. enterica serovar Newport str. USMARC−S3124.1
SNSL254_A2028
exodeoxyribonuclease 8
SNSL254_A2029
phage integrase
SNSL254_A2030
SNSL254_A2036
SNSL254_A2027
SNSL254_A2033
Salmonella enterica subsp. enterica serovar Newport str. SL254

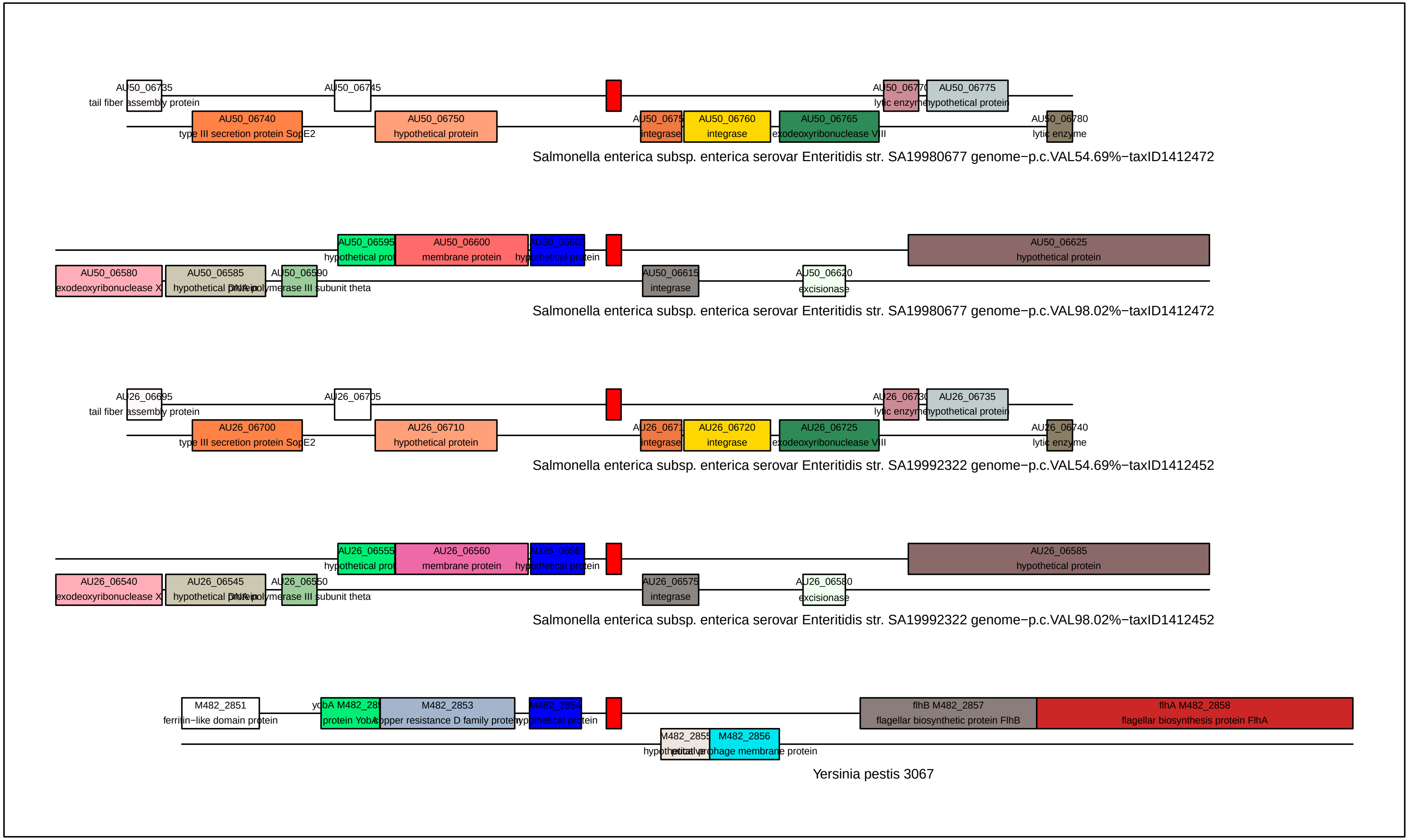
AU50_06735
tail fiber assembly protein
AU50_06745
AU50_06775
hypothetical protein
AU50_06740
type III secretion protein SopE2
AU50_06750
hypothetical protein
integrase
AU50_06760
integrase
AU50_06765
exodeoxyribonuclease VIII
lytic enzyme
Salmonella enterica subsp. enterica serovar Enteritidis str. SA19980677 genome−p.c.VAL54.69%−taxID1412472
AU50_06595
AU50_06600
membrane protein
AU50_06625
hypothetical protein
AU50_06580
exodeoxyribonuclease X
AU50_06585
AU50_06615
integrase
AU50_06620
excisionase
Salmonella enterica subsp. enterica serovar Enteritidis str. SA19980677 genome−p.c.VAL98.02%−taxID1412472
AU26_06695
tail fiber assembly protein
AU26_06705
AU26_06735
hypothetical protein
AU26_06700
type III secretion protein SopE2
AU26_06710
hypothetical protein
integrase
AU26_06720
integrase
AU26_06725
exodeoxyribonuclease VIII
lytic enzyme
Salmonella enterica subsp. enterica serovar Enteritidis str. SA19992322 genome−p.c.VAL54.69%−taxID1412452
AU26_06555
AU26_06560
membrane protein
AU26_06585
hypothetical protein
AU26_06540
exodeoxyribonuclease X
AU26_06545
AU26_06575
integrase
AU26_06580
excisionase
Salmonella enterica subsp. enterica serovar Enteritidis str. SA19992322 genome−p.c.VAL98.02%−taxID1412452
M482_2851
ferritin−like domain protein
M482_2853
flhB M482_2857
flagellar biosynthetic protein FlhB
flhA M482_2858
flagellar biosynthesis protein FlhA
M482_2856
Yersinia pestis 3067

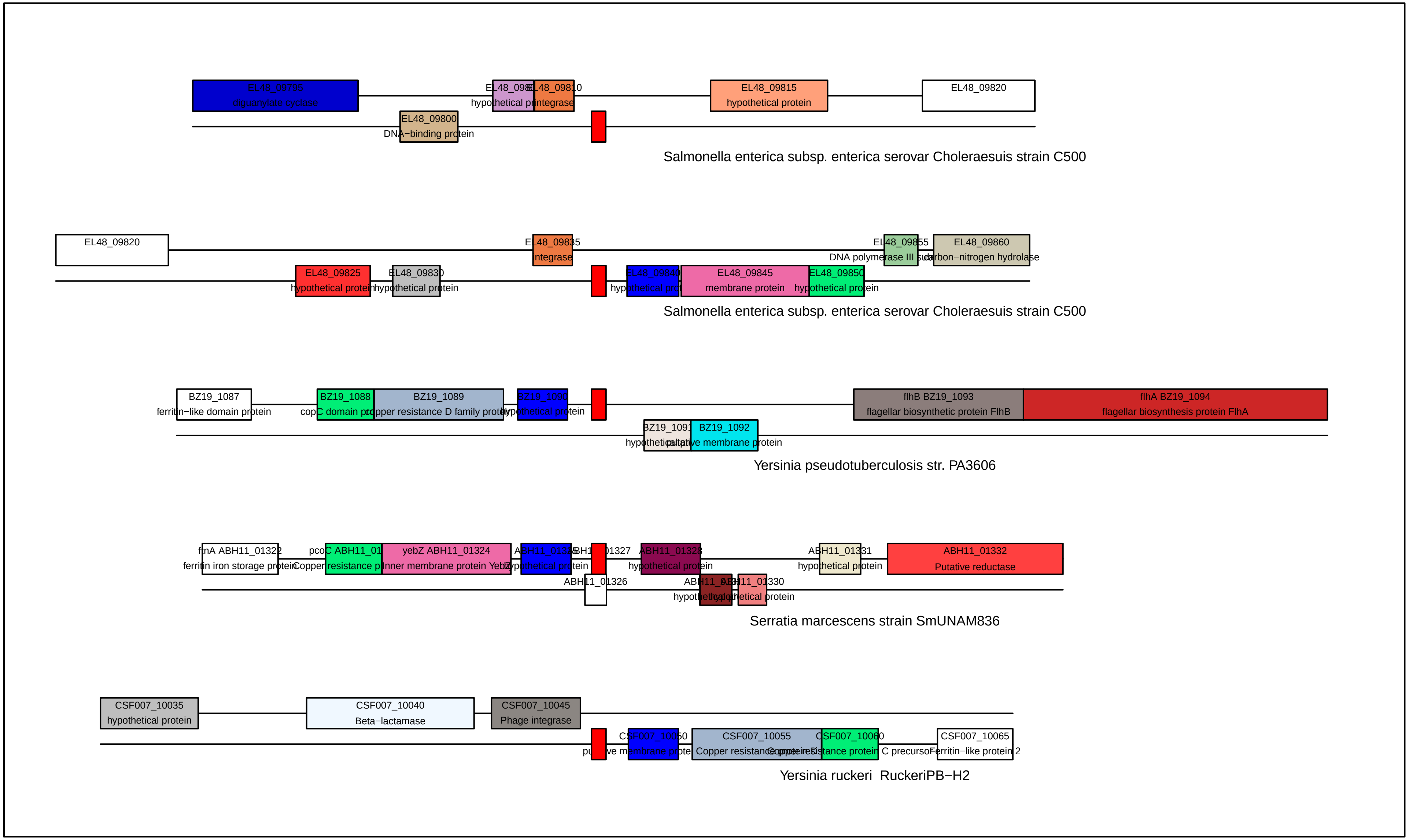

EL48_09795
diguanylate cyclase
EL48_09810
integrase
EL48_09815
hypothetical protein
EL48_09820
EL48_09800
DNA−binding protein
Salmonella enterica subsp. enterica serovar Choleraesuis strain C500
EL48_09820
EL48_09835
integrase
EL48_09860
carbon−nitrogen hydrolase
EL48_09825
hypothetical protein
EL48_09830
hypothetical protein
EL48_09845
membrane protein
EL48_09850
hypothetical protein
Salmonella enterica subsp. enterica serovar Choleraesuis strain C500
BZ19_1087
BZ19_1088
BZ19_1089
flhB BZ19_1093
flagellar biosynthetic protein FlhB
flhA BZ19_1094
flagellar biosynthesis protein FlhA
BZ19_1092
Yersinia pseudotuberculosis str. PA3606
yebZ ABH11_01324
ABH11_01326
ABH11_01331
hypothetical protein
ABH11_01332
Putative reductase
Serratia marcescens strain SmUNAM836
CSF007_10035
hypothetical protein
CSF007_10040
Beta−lactamase
CSF007_10045
Phage integrase
CSF007_10055
CSF007_10065
Yersinia ruckeri RuckeriPB−H2

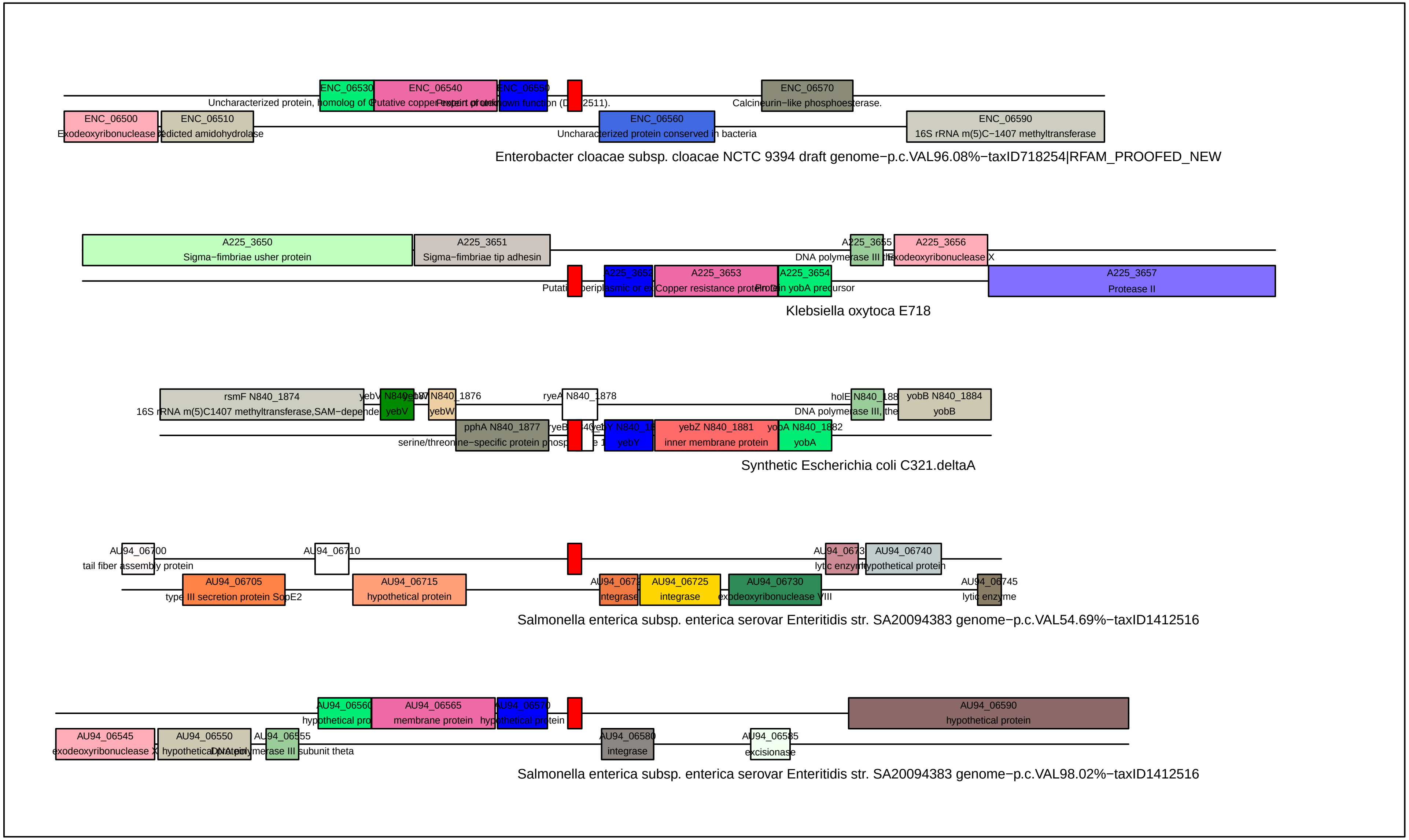
ENC_06530
ENC_06540
ENC_06550
ENC_06570
Calcineurin−like phosphoesterase.
ENC_06500
Exodeoxyribonuclease X
ENC_06510
ENC_06560
Uncharacterized protein conserved in bacteria
ENC_06590
16S rRNA m(5)C−1407 methyltransferase
Enterobacter cloacae subsp. cloacae NCTC 9394 draft genome−p.c.VAL96.08%−taxID718254|RFAM_PROOFED_NEW
A225_3650
Sigma−fimbriae usher protein
A225_3651
Sigma−fimbriae tip adhesin
A225_3655
A225_3656
Exodeoxyribonuclease X
A225_3653
A225_3654
A225_3657
Protease II
Klebsiella oxytoca E718
rsmF N840_1874
yebV
yebW
N840_1876
ryeA N840_1878
holE
yobB N840_1884
yobB
pphA N840_1877
yebZ N840_1881
inner membrane protein
yobA N840_1882
yobA
yebY
Synthetic Escherichia coli C321.deltaA
AU94_06700
tail fiber assembly protein
AU94_06710
AU94_06740
hypothetical protein
AU94_06705
type III secretion protein SopE2
AU94_06715
hypothetical protein
AU94_06725
integrase
AU94_06730
exodeoxyribonuclease VIII
AU94_06745
lytic enzyme
Salmonella enterica subsp. enterica serovar Enteritidis str. SA20094383 genome−p.c.VAL54.69%−taxID1412516
AU94_06565
membrane protein
AU94_06590
hypothetical protein
AU94_06545
exodeoxyribonuclease X
AU94_06550
AU94_06555
AU94_06580
integrase
AU94_06585
excisionase
Salmonella enterica subsp. enterica serovar Enteritidis str. SA20094383 genome−p.c.VAL98.02%−taxID1412516

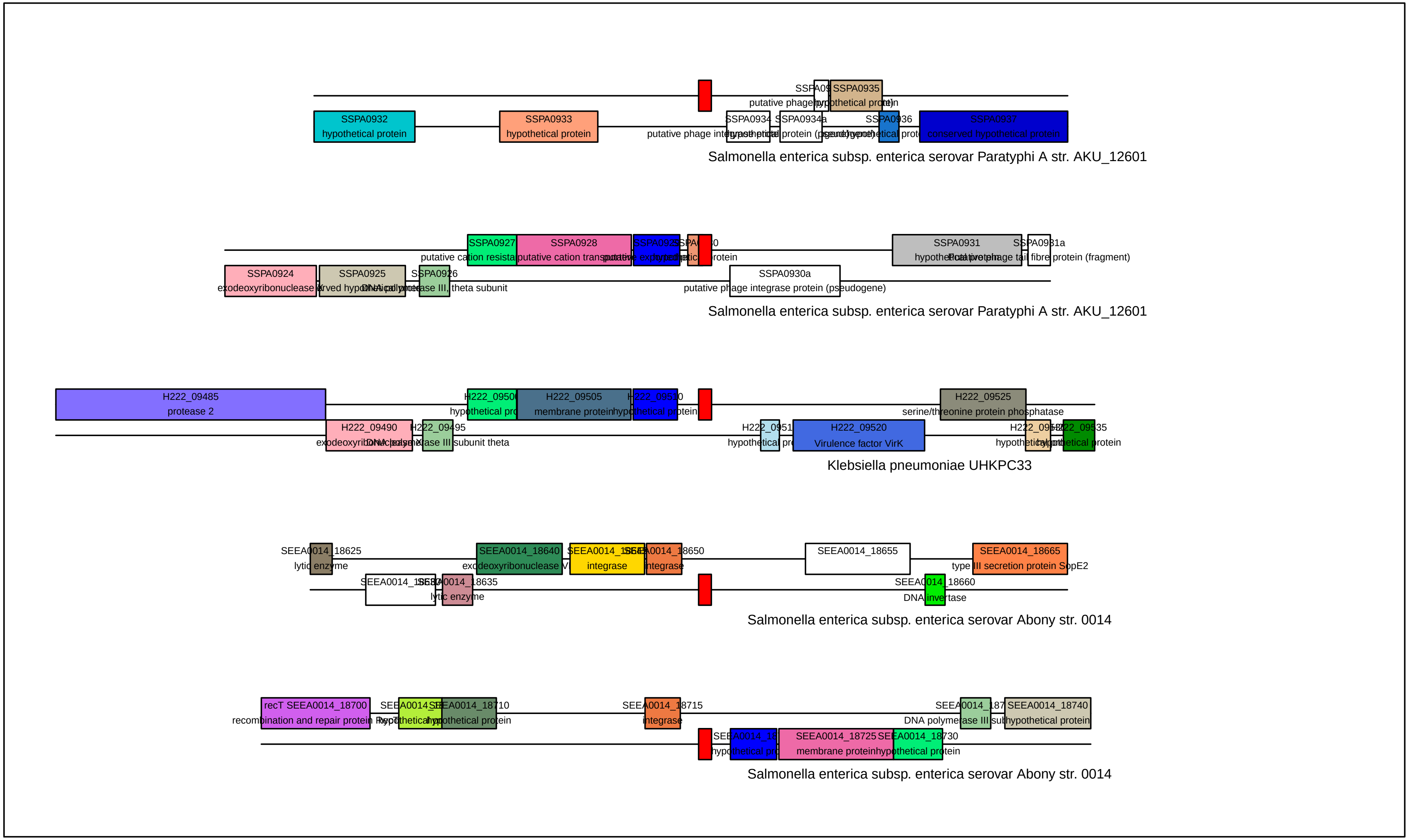

SSPA0935
hypothetical protein
SSPA0932
hypothetical protein
SSPA0933
hypothetical protein
SSPA0934
SSPA0934a
SSPA0936
SSPA0937
conserved hypothetical protein
Salmonella enterica subsp. enterica serovar Paratyphi A str. AKU_12601
SSPA0927
SSPA0928
putative cation transport protein
SSPA0931
SSPA0931a
putative phage tail fibre protein (fragment)
SSPA0924
SSPA0925
SSPA0926
theta subunit
SSPA0930a
putative phage integrase protein (pseudogene)
Salmonella enterica subsp. enterica serovar Paratyphi A str. AKU_12601
H222_09485
protease 2
H222_09505
membrane protein
H222_09525
serine/threonine protein phosphatase
H222_09490
H222_09495
subunit theta
H222_09520
Virulence factor VirK
H222_09535
Klebsiella pneumoniae UHKPC33
SEEA0014_18625
lytic enzyme
SEEA0014_18640
exodeoxyribonuclease
integrase
SEEA0014_18650
SEEA0014_18655
SEEA0014_18665
type III secretion protein SopE2
SEEA0014_18635
lytic enzyme
SEEA0014_18660
DNA invertase
Salmonella enterica subsp. enterica serovar Abony str. 0014
recT SEEA0014_18700
recombination and repair protein RecT
SEEA0014_18710
hypothetical protein
SEEA0014_18715
integrase
SEEA0014_18740
hypothetical protein
DNA polymerase III
SEEA0014_18725
membrane protein
SEEA0014_18730
hypothetical protein
Salmonella enterica subsp. enterica serovar Abony str. 0014

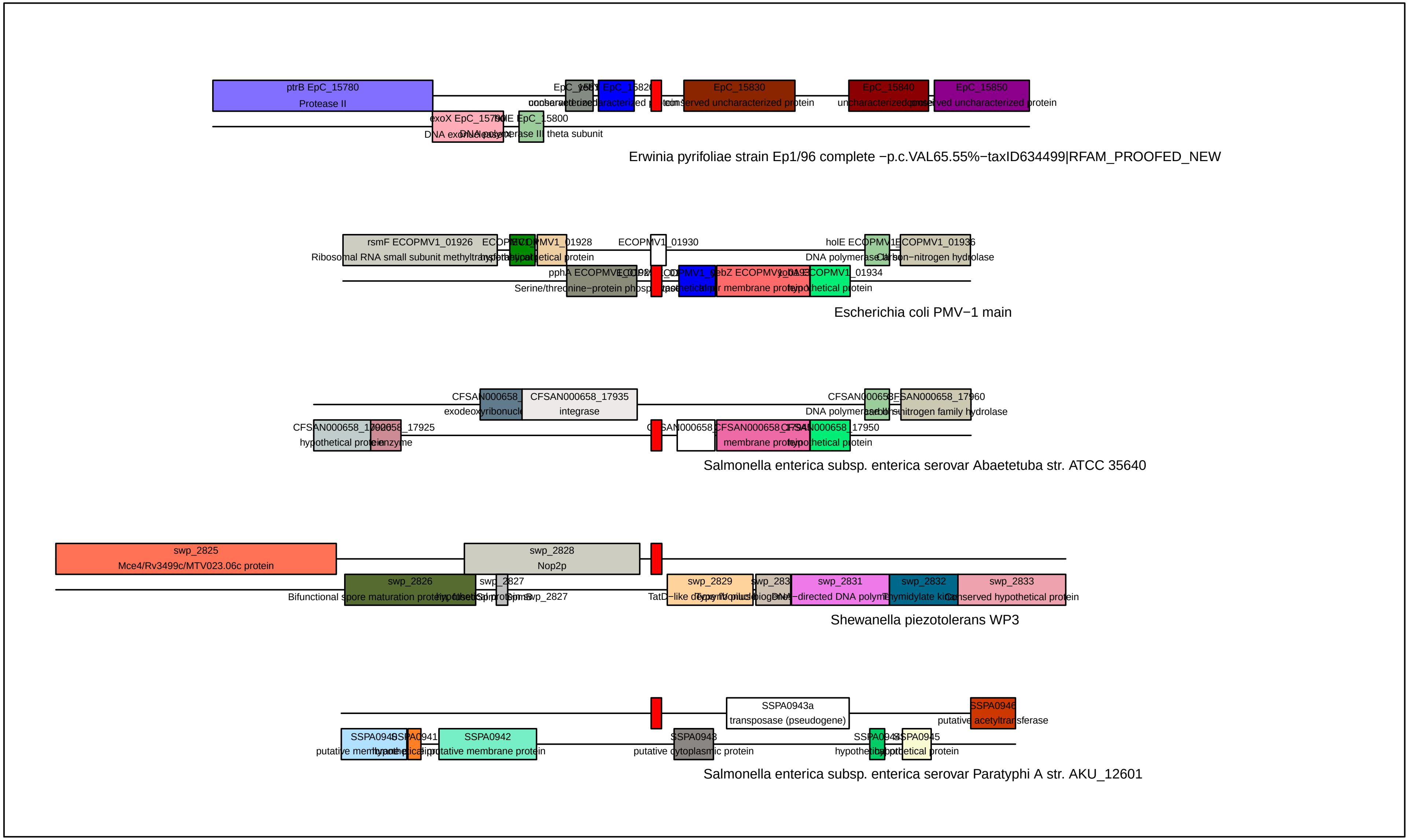

ptrB EpC_15780
Protease II
EpC_15830
EpC_15840
EpC_15850
conserved uncharacterized protein
exoX EpC_15790
holE EpC_15800
DNA polymerase III theta subunit
Erwinia pyrifoliae strain Ep1/96 complete −p.c.VAL65.55%−taxID634499|RFAM_PROOFED_NEW
rsmF ECOPMV1_01926
Ribosomal RNA small subunit methyltransferase F
ECOPMV1_01930
holE ECOPMV1
ECOPMV1_01936
Carbon−nitrogen hydrolase
ECOPMV1_01934
Serine/threonine−protein phosphatase
hypothetical protein
Escherichia coli PMV−1 main
CFSAN000658_17935
integrase
CFSAN000658_17960
DNA polymerase
CFSAN000658_17950
membrane protein
hypothetical protein
Salmonella enterica subsp. enterica serovar Abaetetuba str. ATCC 35640
swp_2825
Mce4/Rv3499c/MTV023.06c protein
swp_2828
Nop2p
swp_2826
swp_2827
swp_2829
swp_2831
swp_2832
swp_2833
Bifunctional spore maturation protein
Conserved hypothetical protein
Shewanella piezotolerans WP3
SSPA0943a
transposase (pseudogene)
SSPA0946
putative acetyltransferase
SSPA0942
putative membrane protein
putative cytoplasmic protein
SSPA0945
hypothetical protein
Salmonella enterica subsp. enterica serovar Paratyphi A str. AKU_12601

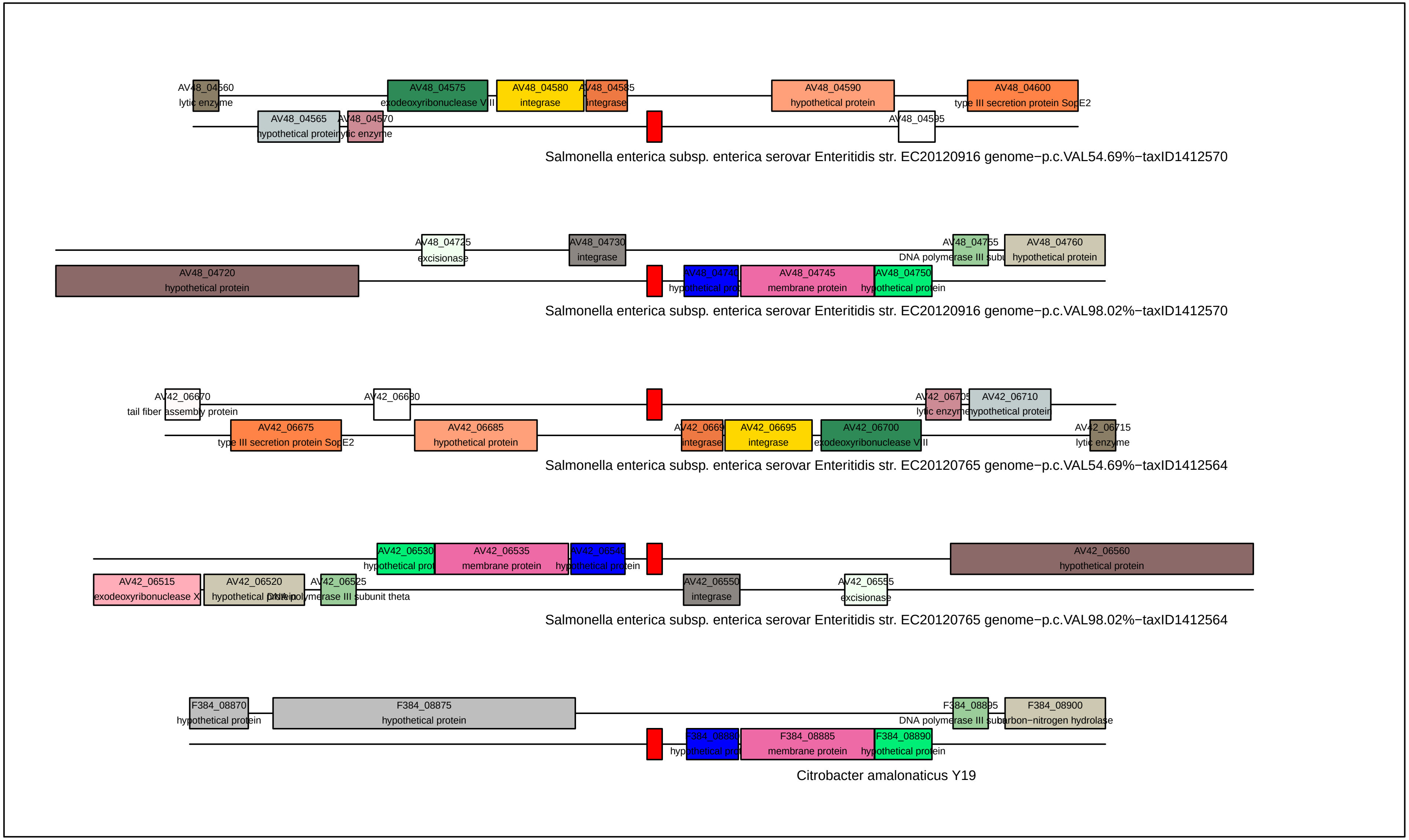
AV48_04575
AV48_04580
integrase
AV48_04590
hypothetical protein
AV48_04600
AV48_04565
AV48_04595
Salmonella enterica subsp. enterica serovar Enteritidis str. EC20120916 genome−p.c.VAL54.69%−taxID1412570
AV48_04725
excisionase
AV48_04730
integrase
AV48_04760
hypothetical protein
AV48_04720
hypothetical protein
AV48_04745
membrane protein
AV48_04750
Salmonella enterica subsp. enterica serovar Enteritidis str. EC20120916 genome−p.c.VAL98.02%−taxID1412570
AV42_06670
AV42_06680
AV42_06710
AV42_06675
AV42_06685
hypothetical protein
AV42_06695
integrase
AV42_06700
exodeoxyribonuclease VIII
Salmonella enterica subsp. enterica serovar Enteritidis str. EC20120765 genome−p.c.VAL54.69%−taxID1412564
AV42_06530
AV42_06535
membrane protein
AV42_06560
hypothetical protein
AV42_06515
AV42_06520
AV42_06525
AV42_06550
integrase
AV42_06555
excisionase
Salmonella enterica subsp. enterica serovar Enteritidis str. EC20120765 genome−p.c.VAL98.02%−taxID1412564
F384_08870
F384_08875
hypothetical protein
F384_08900
F384_08885
membrane protein
F384_08890
Citrobacter amalonaticus Y19

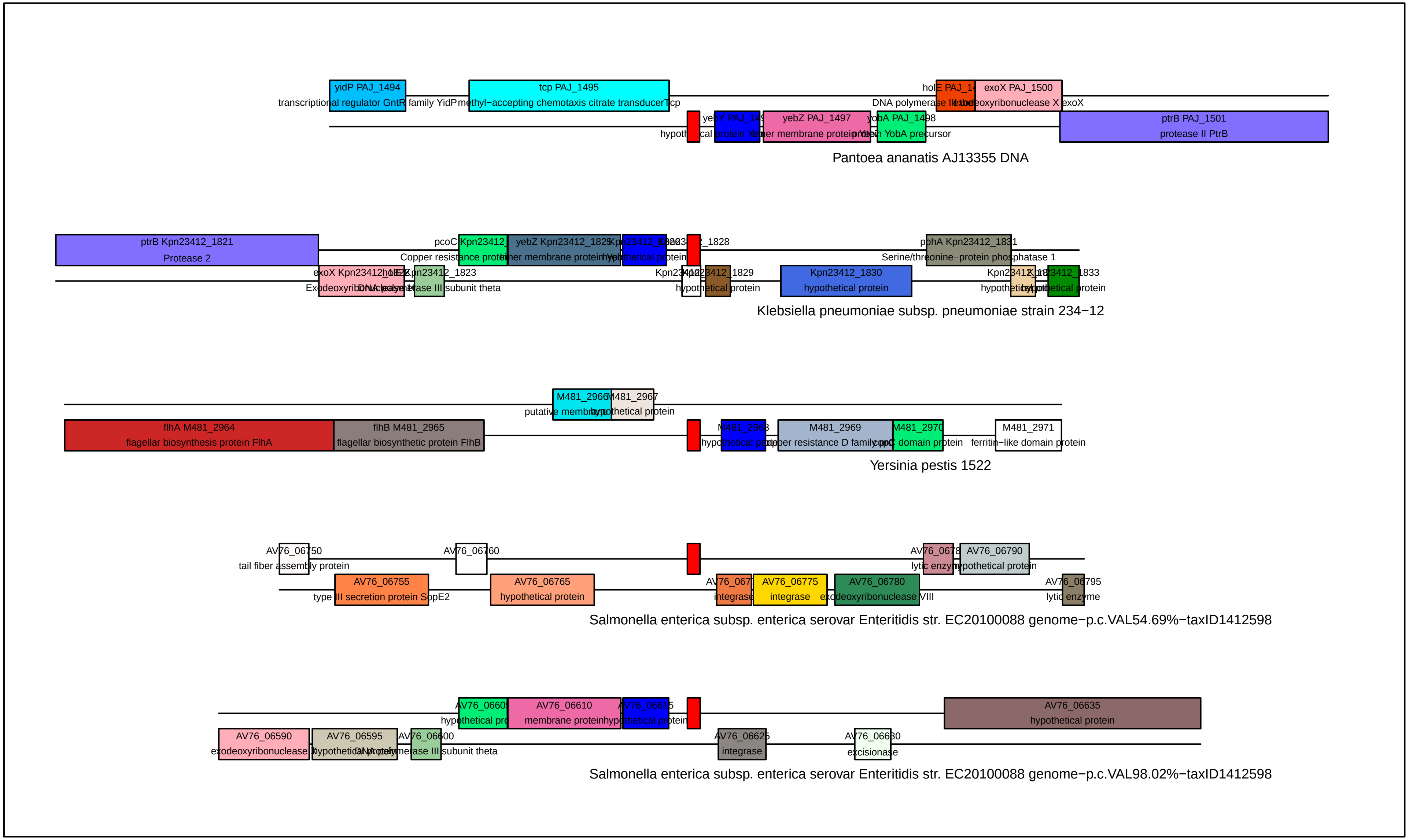
yidP PAJ_1494
transcriptional regulator GntR family YidP
tcp PAJ_1495
methyl−accepting chemotaxis citrate transducer Tcp
exoX PAJ_1500
exodeoxyribonuclease X exoX
yebZ PAJ_1497
yobA PAJ_1498
protein YobA precursor
ptrB PAJ_1501
protease II PtrB
Pantoea ananatis AJ13355 DNA
ptrB Kpn23412_1821
Protease 2
yebZ Kpn23412_1825
Kpn23412_1830
hypothetical protein
Serine/threonine−protein phosphatase 1
DNA polymerase III subunit theta
Klebsiella pneumoniae subsp. pneumoniae strain 234−12
M481_2966
M481_2967
hypothetical protein
flhA M481_2964
flagellar biosynthesis protein FlhA
flhB M481_2965
flagellar biosynthetic protein FlhB
M481_2969
M481_2970
M481_2971
ferritin−like domain protein
Yersinia pestis 1522
AV76_06750
tail fiber assembly protein
AV76_06760
AV76_06790
AV76_06755
type III secretion protein SopE2
AV76_06765
hypothetical protein
AV76_06775
integrase
AV76_06780
exodeoxyribonuclease VIII
AV76_06795
lytic enzyme
Salmonella enterica subsp. enterica serovar Enteritidis str. EC20100088 genome−p.c.VAL54.69%−taxID1412598
AV76_06610
AV76_06635
hypothetical protein
AV76_06590
AV76_06595
AV76_06625
integrase
excisionase
DNA polymerase III subunit theta
Salmonella enterica subsp. enterica serovar Enteritidis str. EC20100088 genome−p.c.VAL98.02%−taxID1412598

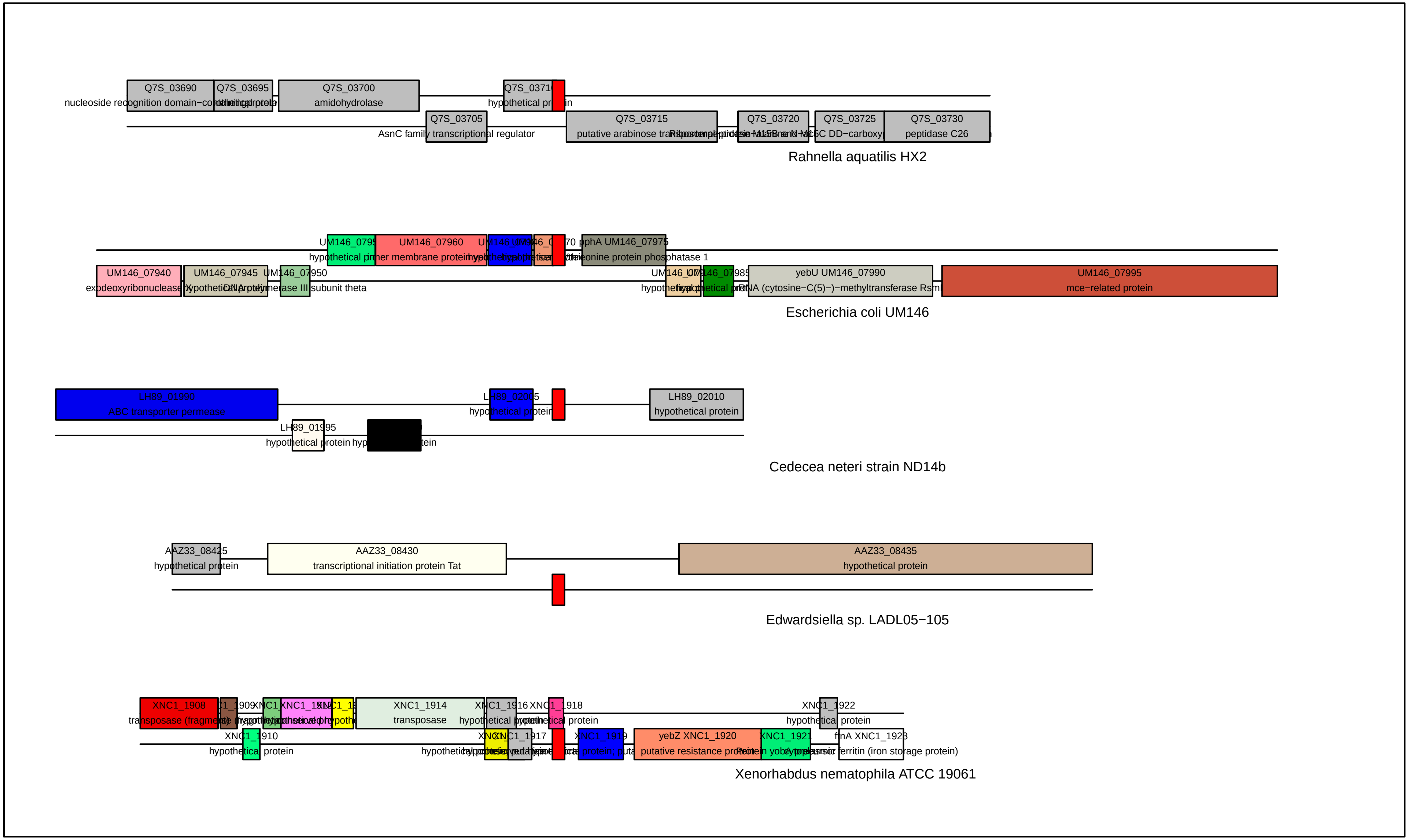
Q7S_03690
Q7S_03695
Q7S_03700
amidohydrolase
hypothetical protein
Q7S_03705
AsnC family transcriptional regulator
Q7S_03715
Q7S_03720
Q7S_03725
Q7S_03730
peptidase C26
Rahnella aquatilis HX2
UM146_07960
threonine protein phosphatase 1
UM146_07940
UM146_07945
DNA polymerase III subunit theta
yebU UM146_07990
UM146_07995
mce−related protein
Escherichia coli UM146
ABC transporter permease
hypothetical protein
LH89_02010
hypothetical protein
Cedecea neteri strain ND14b
AAZ33_08425
hypothetical protein
AAZ33_08430
transcriptional initiation protein Tat
AAZ33_08435
hypothetical protein
Edwardsiella sp. LADL05−105
XNC1_1908
XNC1_1910
hypothetical protein
XNC1_1914
transposase
XNC1_1917
XNC1_1918
hypothetical protein
yebZ XNC1_1920
XNC1_1921
XNC1_1922
hypothetical protein
ferritin (iron storage protein)
Xenorhabdus nematophila ATCC 19061

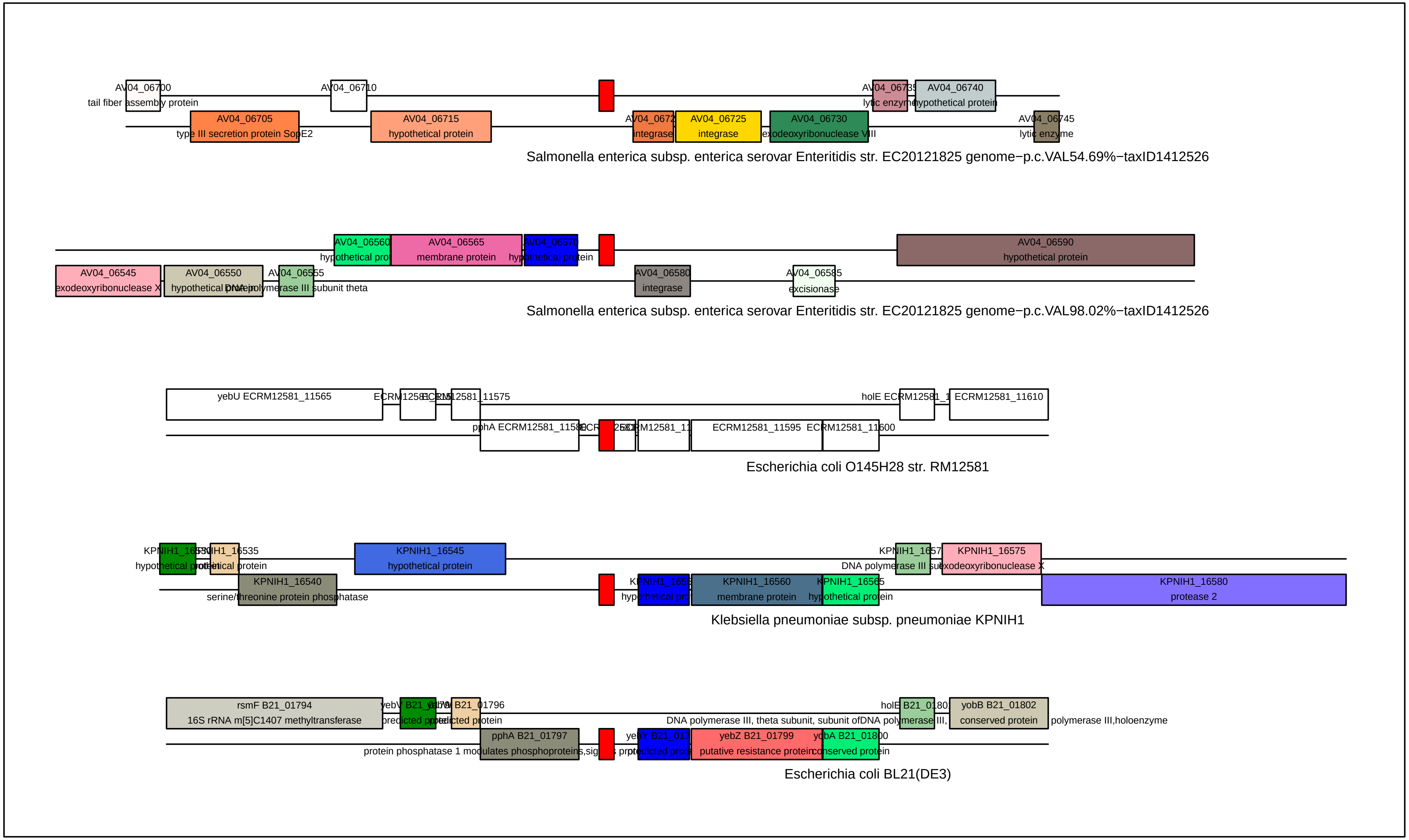
AV04_06700
tail fiber assembly protein
AV04_06710
AV04_06740
hypothetical protein
AV04_06705
type III secretion protein SopE2
AV04_06715
hypothetical protein
integrase
AV04_06725
integrase
AV04_06730
exodeoxyribonuclease VIII
AV04_06745
lytic enzyme
Salmonella enterica subsp. enterica serovar Enteritidis str. EC20121825 genome−p.c.VAL54.69%−taxID1412526
AV04_06560
AV04_06565
membrane protein
AV04_06590
hypothetical protein
AV04_06545
exodeoxyribonuclease X
AV04_06550
AV04_06555
DNA polymerase III subunit theta
AV04_06580
integrase
AV04_06585
excisionase
Salmonella enterica subsp. enterica serovar Enteritidis str. EC20121825 genome−p.c.VAL98.02%−taxID1412526
yebU ECRM12581_11565
ECRM12581_11575
ECRM12581_11610
pphA ECRM12581_11580
ECRM12581_11595
ECRM12581_11600
Escherichia coli O145H28 str. RM12581
KPNIH1_16535
KPNIH1_16545
hypothetical protein
KPNIH1_16575
exodeoxyribonuclease X
KPNIH1_16540
serine/threonine protein phosphatase
KPNIH1_16560
membrane protein
KPNIH1_16565
hypothetical protein
KPNIH1_16580
protease 2
Klebsiella pneumoniae subsp. pneumoniae KPNIH1
rsmF B21_01794
16S rRNA m[5]C1407 methyltransferase
B21_01796
yobB B21_01802
conserved protein
polymerase III,holoenzyme
pphA B21_01797
protein phosphatase 1 modulates phosphoproteins,sig
yebZ B21_01799
putative resistance protein
conserved protein
Escherichia coli BL21(DE3)

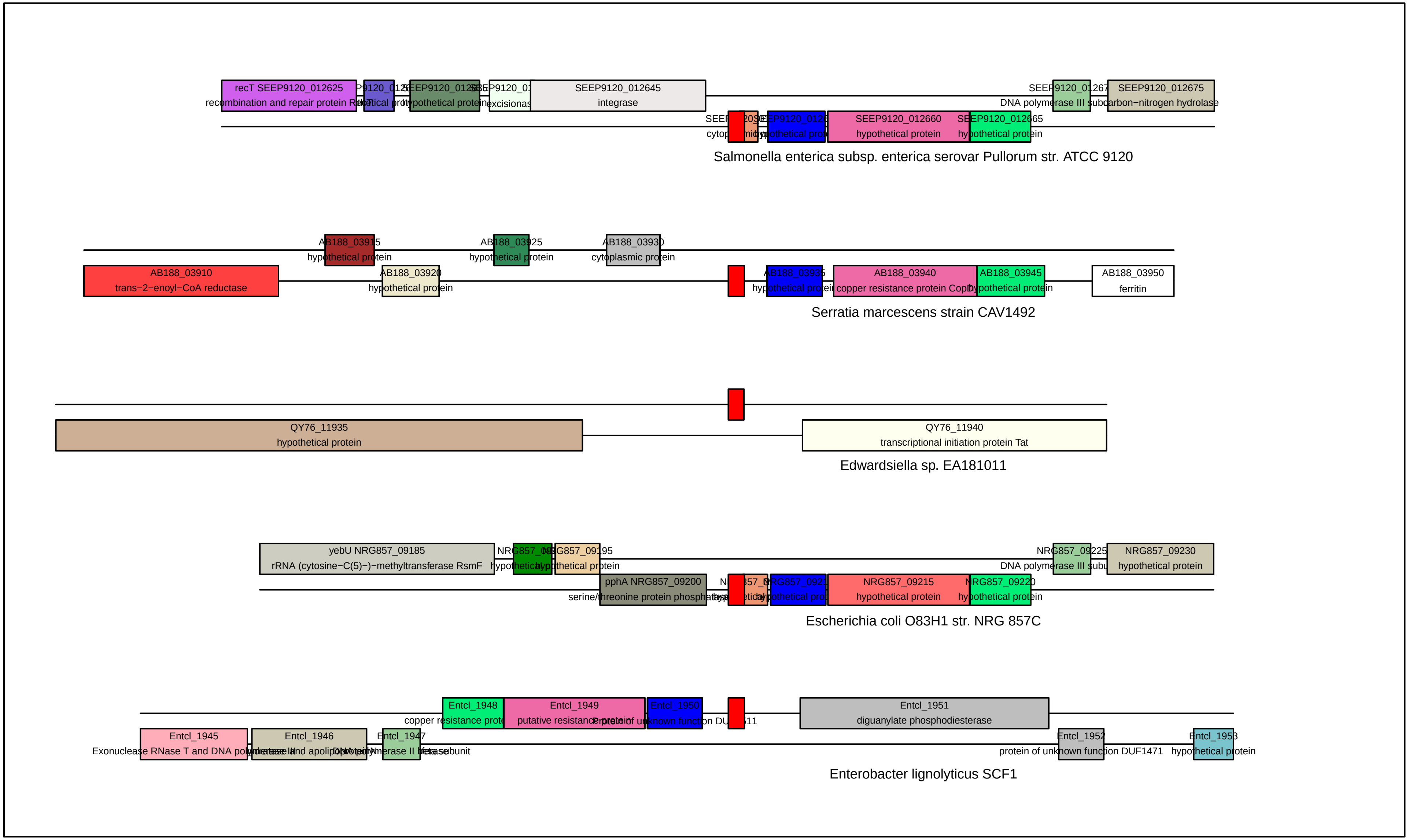
recT SEEP9120_012625
SEEP9120_012645
integrase
SEEP9120_012660
hypothetical protein
SEEP9120_012675
carbon−nitrogen hydrolase
Salmonella enterica subsp. enterica serovar Pullorum str. ATCC 9120
AB188_03915
hypothetical protein
AB188_03925
hypothetical protein
AB188_03930
cytoplasmic protein
AB188_03910
trans−2−enoyl−CoA reductase
AB188_03920
hypothetical protein
AB188_03940
AB188_03945
AB188_03950
ferritin
Serratia marcescens strain CAV1492
QY76_11935
hypothetical protein
QY76_11940
transcriptional initiation protein Tat
Edwardsiella sp. EA181011
yebU NRG857_09185
rRNA (cytosine−C(5)−)−methyltransferase RsmF
NRG857_09195
NRG857_09230
hypothetical protein
pphA NRG857_09200
NRG857_09215
hypothetical protein
NRG857_09220
hypothetical protein
Escherichia coli O83H1 str. NRG 857C
Entcl_1948
Entcl_1949
Entcl_1950
Entcl_1951
diguanylate phosphodiesterase
Entcl_1945
Entcl_1946
Entcl_1947
Entcl_1952
protein of unknown function DUF1471
Entcl_1953
hypothetical protein
Enterobacter lignolyticus SCF1

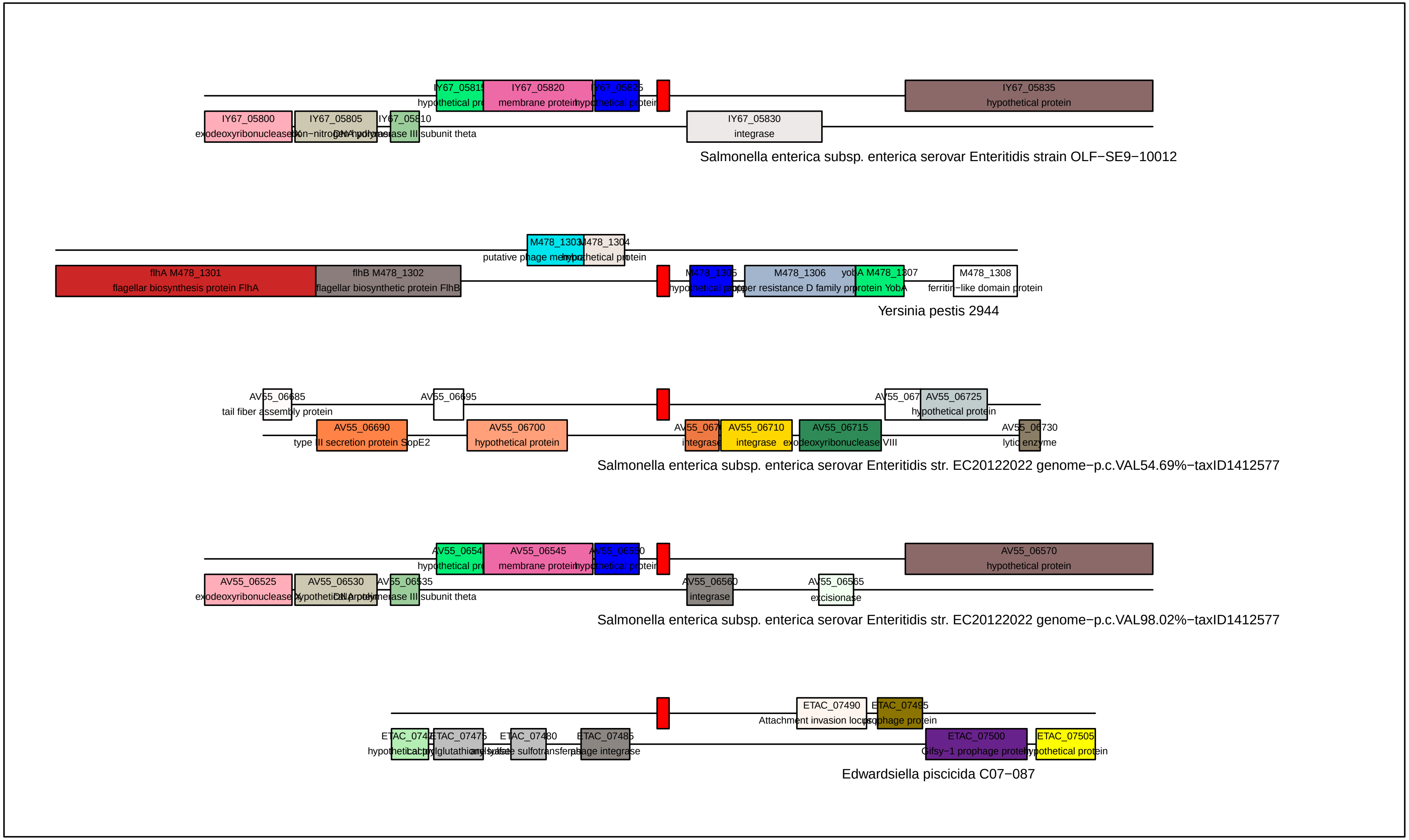
IY67_05820
membrane protein
IY67_05835
hypothetical protein
IY67_05800
IY67_05805
IY67_05830
integrase
Salmonella enterica subsp. enterica serovar Enteritidis strain OLF−SE9−10012
M478_1303
M478_1304
flhA M478_1301
flagellar biosynthesis protein FlhA
flhB M478_1302
flagellar biosynthetic protein FlhB
M478_1306
M478_1308
ferritin−like domain protein
Yersinia pestis 2944
AV55_06685
tail fiber assembly protein
AV55_06725
hypothetical protein
AV55_06690
AV55_06700
hypothetical protein
AV55_06710
integrase
AV55_06715
exodeoxyribonuclease VIII
AV55_06730
lytic enzyme
Salmonella enterica subsp. enterica serovar Enteritidis str. EC20122022 genome−p.c.VAL54.69%−taxID1412577
AV55_06545
membrane protein
AV55_06570
hypothetical protein
AV55_06525
AV55_06530
AV55_06560
integrase
AV55_06565
excisionase
Salmonella enterica subsp. enterica serovar Enteritidis str. EC20122022 genome−p.c.VAL98.02%−taxID1412577
ETAC_07490
ETAC_07495
ETAC_07475
ETAC_07480
ETAC_07485
phage integrase
ETAC_07500
ETAC_07505
hypothetical protein
Edwardsiella piscicida C07−087

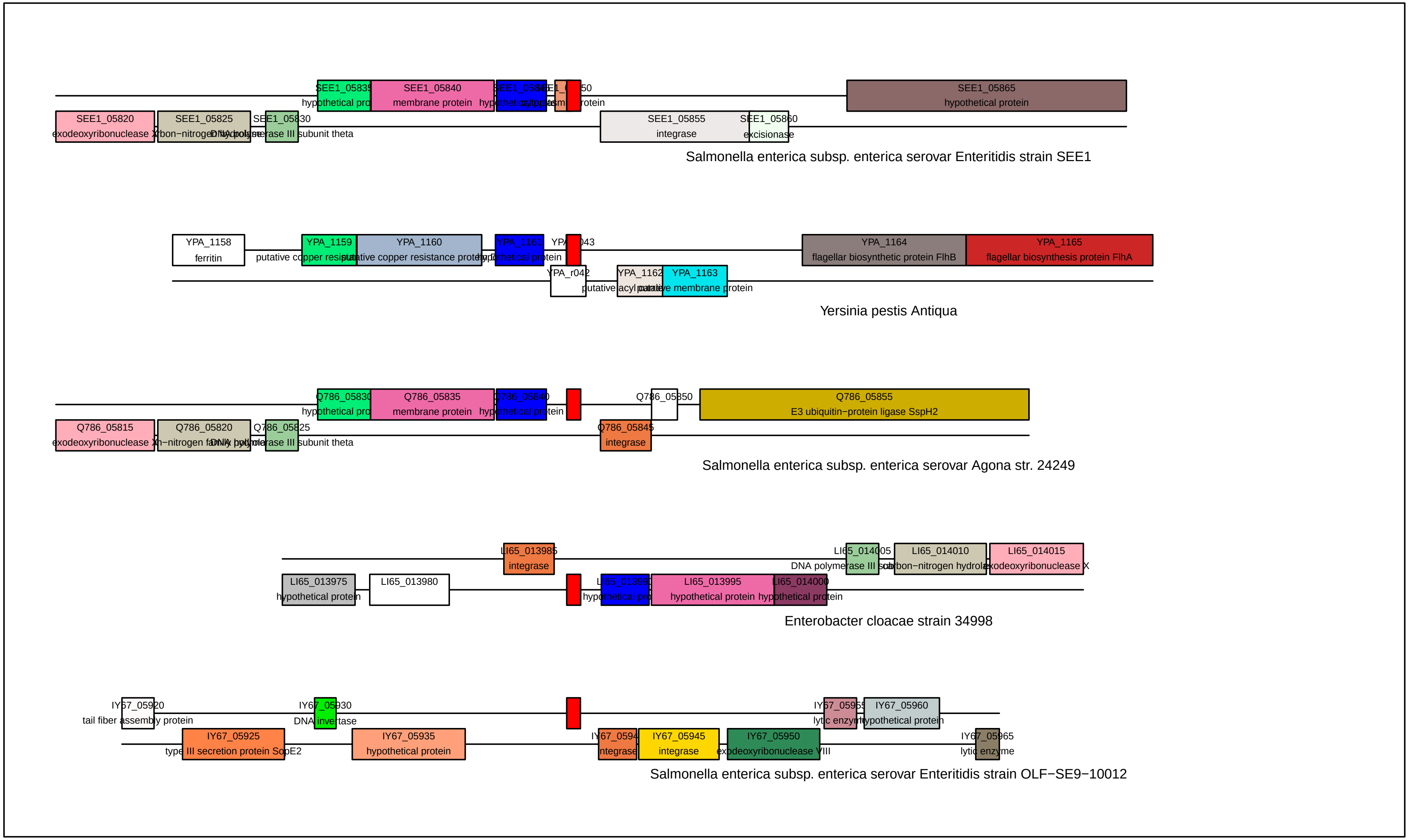

SEE1_05820
exodeoxyribonuclease X
SEE1_05825
SEE1_05830
subunit theta
SEE1_05840
membrane protein
SEE1_05855
integrase
excisionase
SEE1_05865
hypothetical protein
Salmonella enterica subsp. enterica serovar Enteritidis strain SEE1
YPA_1158
ferritin
YPA_1159
YPA_1160
YPA_1162
YPA_1163
YPA_1164
flagellar biosynthetic protein FlhB
YPA_1165
flagellar biosynthesis protein FlhA
Yersinia pestis Antiqua
Q786_05815
exodeoxyribonuclease X
Q786_05820
Q786_05835
membrane protein
Q786_05850
Q786_05855
E3 ubiquitin−protein ligase SspH2
integrase
subunit theta
Salmonella enterica subsp. enterica serovar Agona str. 24249
LI65_013975
hypothetical protein
LI65_013980
LI65_013985
integrase
LI65_013995
hypothetical protein
LI65_014010
LI65_014015
exodeoxyribonuclease X
Enterobacter cloacae strain 34998
IY67_05920
tail fiber assembly protein
IY67_05925
type III secretion protein SopE2
DNA invertase
IY67_05935
hypothetical protein
IY67_05945
integrase
IY67_05950
IY67_05960
hypothetical protein
lytic enzyme
Salmonella enterica subsp. enterica serovar Enteritidis strain OLF−SE9−10012

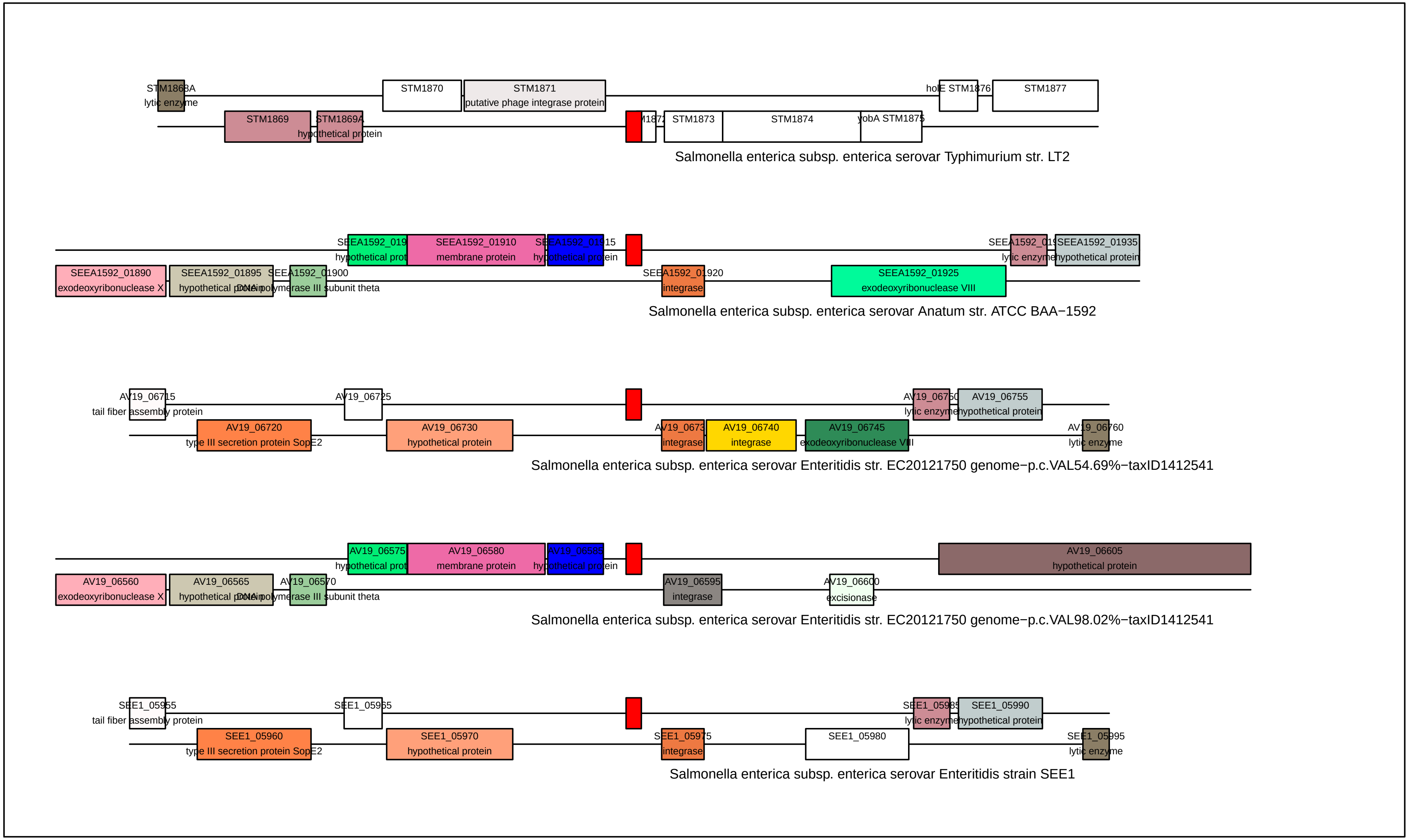
STM1868A
lytic enzyme
STM1870
STM1871
putative phage integrase protein
holE STM1876
STM1877
STM1869
STM1869A
hypothetical protein
STM1873
STM1874
yobA STM1875
Salmonella enterica subsp. enterica serovar Typhimurium str. LT2
SEEA1592_01910
membrane protein
hypothetical protein
SEEA1592_01935
hypothetical protein
lytic enzyme
SEEA1592_01890
exodeoxyribonuclease X
SEEA1592_01895
hypothetical protein
DNA polymerase III subunit theta
integrase
SEEA1592_01925
exodeoxyribonuclease VIII
Salmonella enterica subsp. enterica serovar Anatum str. ATCC BAA−1592
AV19_06715
tail fiber assembly protein
AV19_06725
AV19_06755
hypothetical protein
lytic enzyme
AV19_06720
type III secretion protein SopE2
AV19_06730
hypothetical protein
integrase
AV19_06740
integrase
AV19_06745
exodeoxyribonuclease VIII
AV19_06760
lytic enzyme
Salmonella enterica subsp. enterica serovar Enteritidis str. EC20121750 genome−p.c.VAL54.69%−taxID1412541
AV19_06575
AV19_06580
membrane protein
AV19_06605
hypothetical protein
AV19_06560
exodeoxyribonuclease X
AV19_06565
hypothetical protein
DNA polymerase III subunit theta
AV19_06570
AV19_06595
integrase
AV19_06600
excisionase
Salmonella enterica subsp. enterica serovar Enteritidis str. EC20121750 genome−p.c.VAL98.02%−taxID1412541
SEE1_05955
tail fiber assembly protein
SEE1_05965
SEE1_05990
hypothetical protein
lytic enzyme
SEE1_05960
type III secretion protein SopE2
SEE1_05970
hypothetical protein
SEE1_05975
integrase
SEE1_05980
SEE1_05995
lytic enzyme
Salmonella enterica subsp. enterica serovar Enteritidis strain SEE1

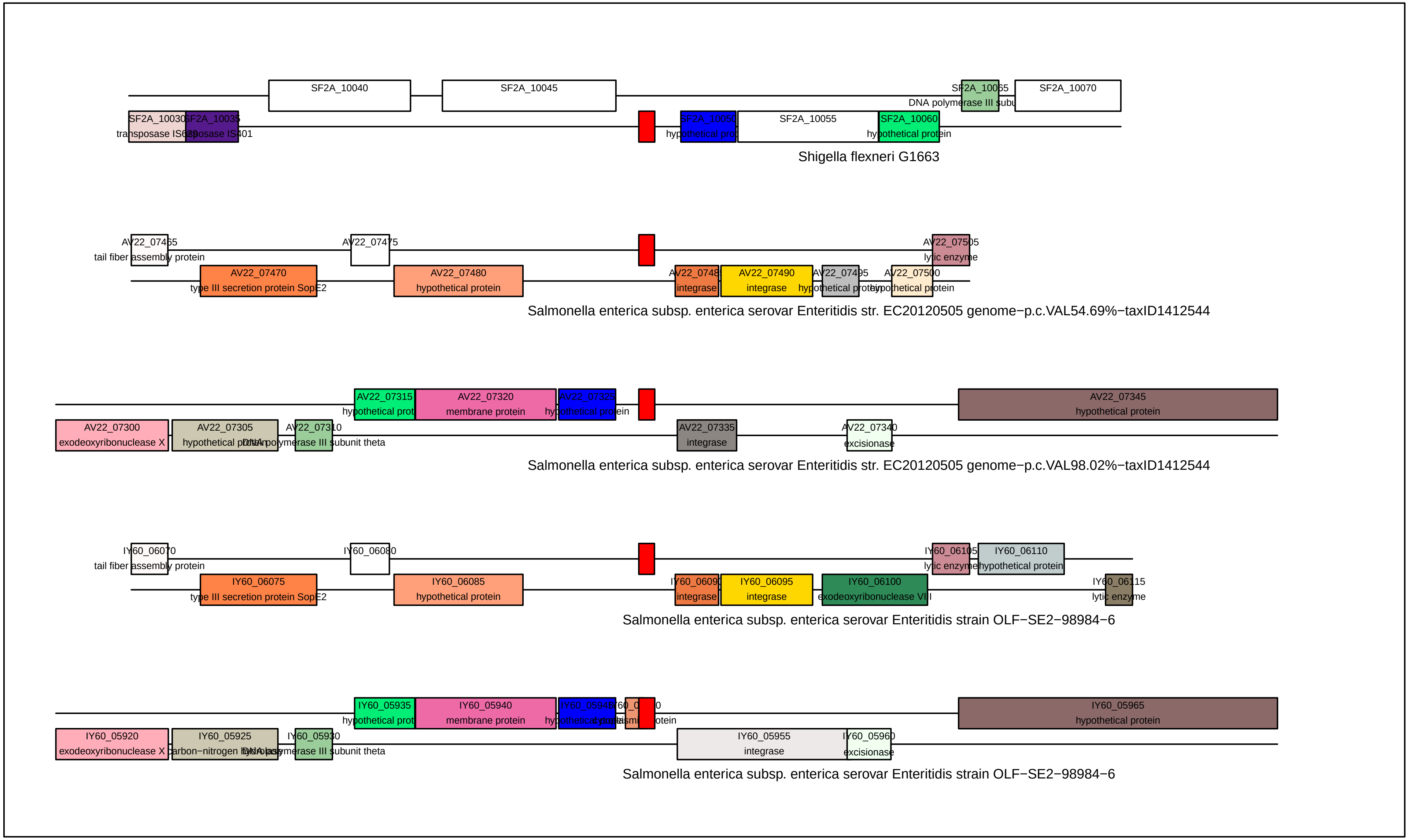

SF2A_10040
SF2A_10045
SF2A_10065
DNA polymerase III subu
SF2A_10070
SF2A_10030
transposase IS
SF2A_10035
transposase IS401
SF2A_10050
hypothetical prot
SF2A_10055
SF2A_10060
hypothetical protein
Shigella flexneri G1663
AV22_07465
tail fiber assembly protein
AV22_07475
AV22_07505
lytic enzyme
AV22_07470
type III secretion protein SopE2
AV22_07480
hypothetical protein
integrase
AV22_07490
integrase
AV22_07495
hypothetical protein
AV22_07500
hypothetical protein
Salmonella enterica subsp. enterica serovar Enteritidis str. EC20120505 genome−p.c.VAL54.69%−taxID1412544
AV22_07315
hypothetical prot
AV22_07320
membrane protein
AV22_07325
hypothetical protein
AV22_07345
hypothetical protein
AV22_07300
exodeoxyribonuclease X
AV22_07305
hypothetical protein
AV22_07310
DNA polymerase III subunit theta
AV22_07335
integrase
AV22_07340
excisionase
Salmonella enterica subsp. enterica serovar Enteritidis str. EC20120505 genome−p.c.VAL98.02%−taxID1412544
IY60_06070
tail fiber assembly protein
IY60_06080
IY60_06105
lytic enzyme
IY60_06110
hypothetical protein
IY60_06075
type III secretion protein SopE2
IY60_06085
hypothetical protein
integrase
IY60_06095
integrase
IY60_06100
exodeoxyribonuclease VIII
IY60_06115
lytic enzyme
Salmonella enterica subsp. enterica serovar Enteritidis strain OLF−SE2−98984−6
IY60_05935
hypothetical prot
IY60_05940
membrane protein
IY60_05965
hypothetical protein
IY60_05920
exodeoxyribonuclease X
IY60_05925
IY60_05930
DNA polymerase III subunit theta
IY60_05955
integrase
IY60_05960
excisionase
Salmonella enterica subsp. enterica serovar Enteritidis strain OLF−SE2−98984−6

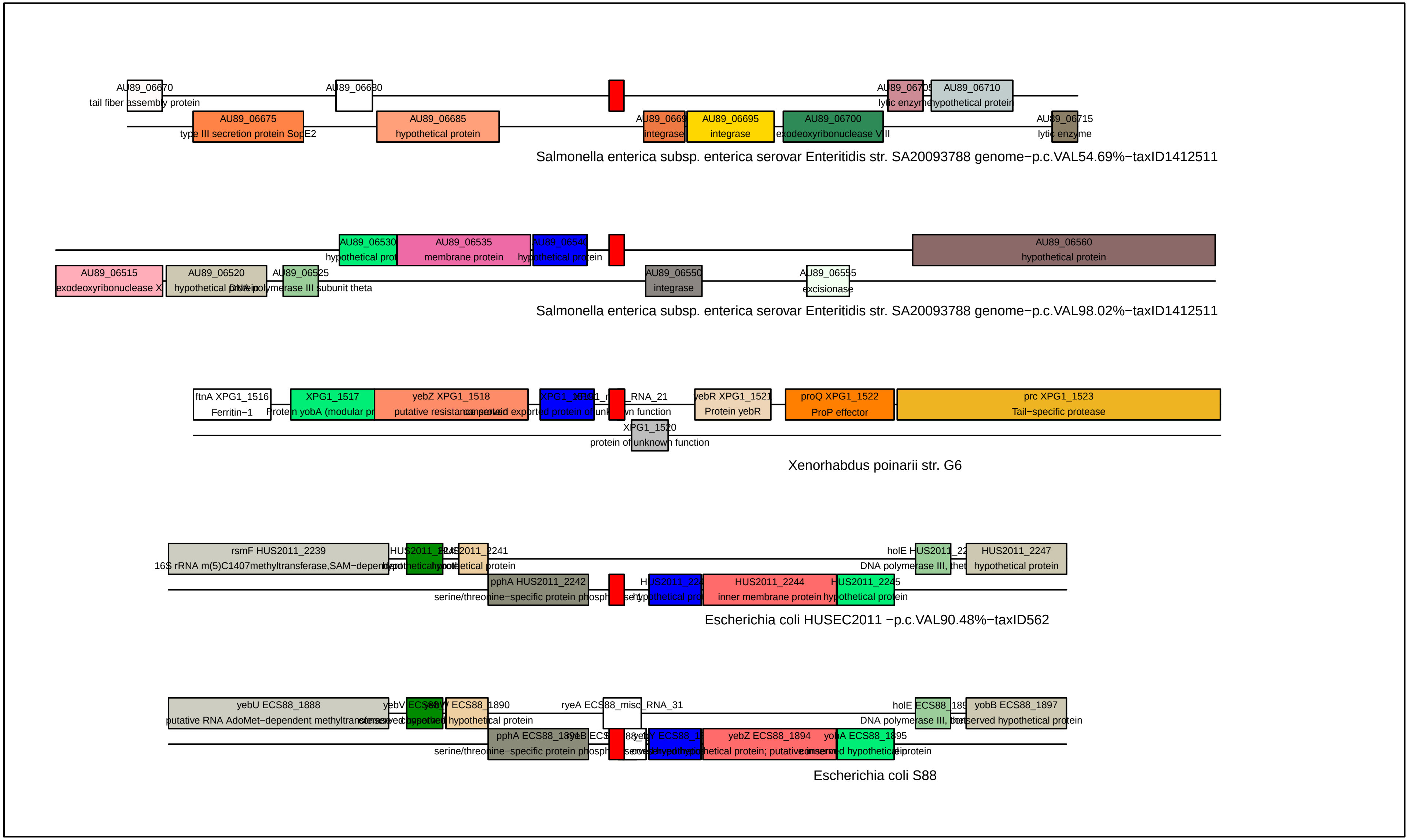

AU89_06670
tail fiber assembly protein
AU89_06680
AU89_06710
hypothetical protein
AU89_06675
type III secretion protein SopE2
AU89_06685
hypothetical protein
integrase
AU89_06695
integrase
AU89_06700
exodeoxyribonuclease VIII
AU89_06715
lytic enzyme
Salmonella enterica subsp. enterica serovar Enteritidis str. SA20093788 genome−p.c.VAL54.69%−taxID1412511
AU89_06535
membrane protein
AU89_06560
hypothetical protein
AU89_06515
exodeoxyribonuclease X
AU89_06520
AU89_06550
integrase
AU89_06555
excisionase
Salmonella enterica subsp. enterica serovar Enteritidis str. SA20093788 genome−p.c.VAL98.02%−taxID1412511
ftnA XPG1_1516
Ferritin−1
XPG1_1517
yebZ XPG1_1518
yebR XPG1_1521
Protein yebR
proQ XPG1_1522
ProP effector
prc XPG1_1523
Tail−specific protease
XPG1_1520
protein of unknown function
Xenorhabdus poinarii str. G6
rsmF HUS2011_2239
hypothetical protein
HUS2011_2247
hypothetical protein
pphA HUS2011_2242
HUS2011_2244
inner membrane protein
Escherichia coli HUSEC2011 −p.c.VAL90.48%−taxID562
yebU ECS88_1888
yobB ECS88_1897
pphA ECS88_1893
yebZ ECS88_1894
yobA ECS88_1895
Escherichia coli S88

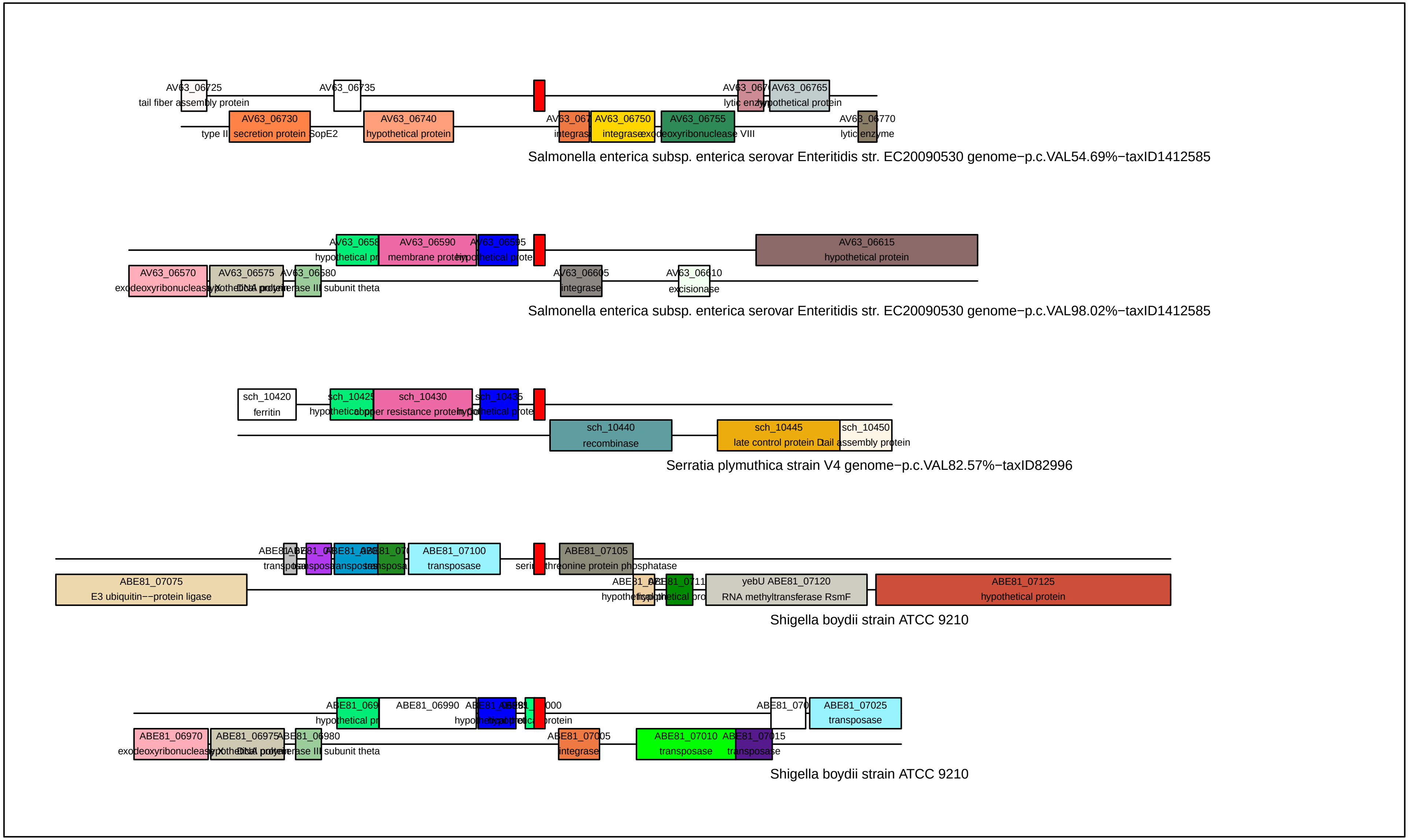

AV63_06725
tail fiber assembly protein
AV63_06735
AV63_06765
hypothetical protein
AV63_06730
secretion protein
AV63_06740
hypothetical protein
AV63_06750
integrase
AV63_06755
AV63_06770
lytic enzyme
Salmonella enterica subsp. enterica serovar Enteritidis str. EC20090530 genome−p.c.VAL54.69%−taxID1412585
AV63_06590
membrane protein
AV63_06615
hypothetical protein
AV63_06570
AV63_06575
AV63_06605
integrase
AV63_06610
excisionase
subunit theta
Salmonella enterica subsp. enterica serovar Enteritidis str. EC20090530 genome−p.c.VAL98.02%−taxID1412585
sch_10420
ferritin
sch_10430
sch_10440
recombinase
sch_10445
late control protein D
sch_10450
tail assembly protein
Serratia plymuthica strain V4 genome−p.c.VAL82.57%−taxID82996
ABE81_07100
transposase
ABE81_07105
threonine protein phosphatase
ABE81_07075
E3 ubiquitin−−protein ligase
yebU ABE81_07120
RNA methyltransferase RsmF
ABE81_07125
hypothetical protein
Shigella boydii strain ATCC 9210
ABE81_06990
ABE81_07025
transposase
ABE81_06970
ABE81_06975
ABE81_06980
subunit theta
ABE81_07005
integrase
ABE81_07010
transposase
ABE81_07015
transposase
Shigella boydii strain ATCC 9210

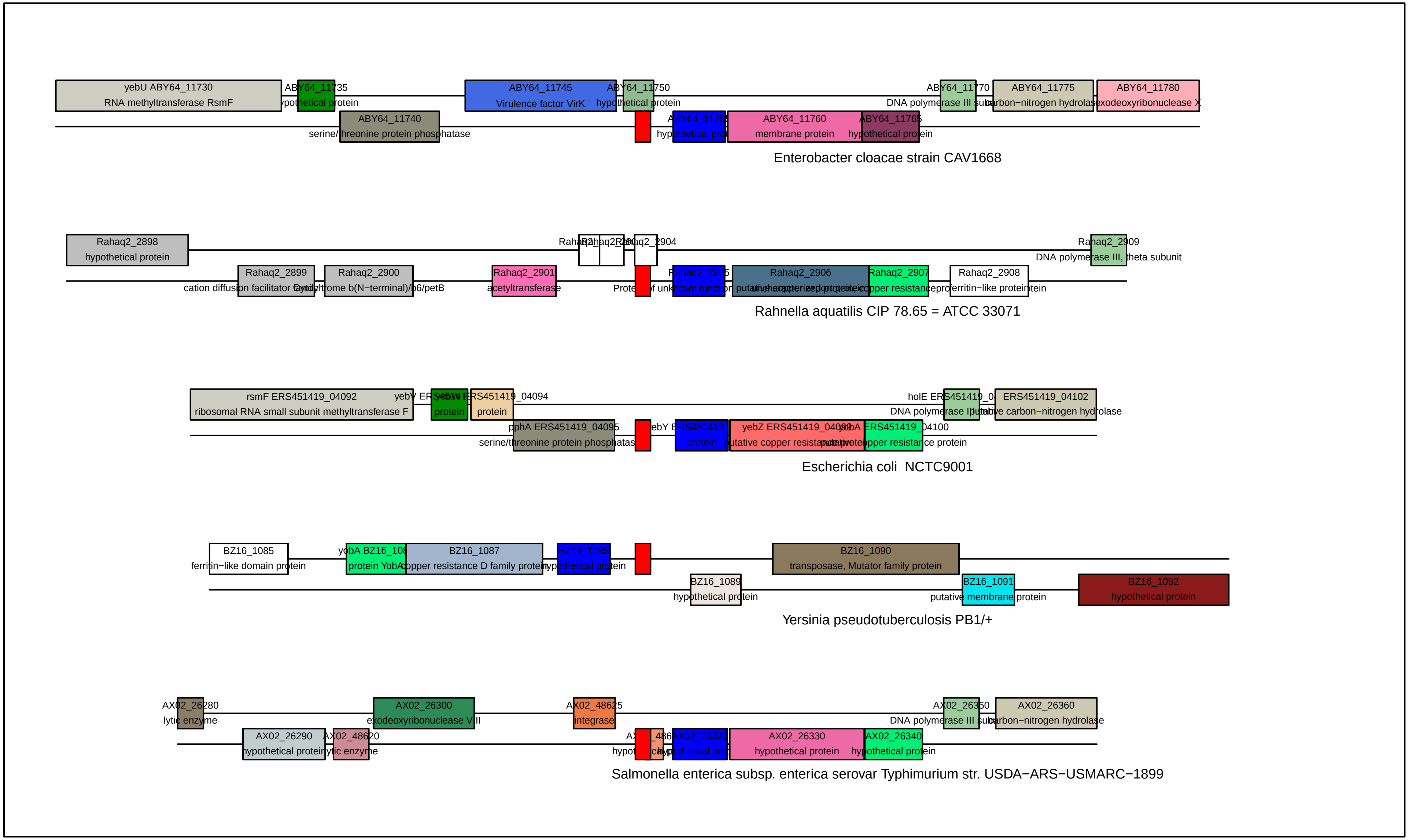
yebU ABY64_11730
RNA methyltransferase RsmF
ABY64_11735
ABY64_11740
serine/threonine protein phosphatase
ABY64_11745
Virulence factor VirK
ABY64_11750
ABY64_11760
membrane protein
ABY64_11765
ABY64_11770
ABY64_11775
ABY64_11780
Enterobacter cloacae strain CAV1668
Rahaq2_2898
hypothetical protein
Rahaq2_2899
Rahaq2_2900
Rahaq2_2901
acetyltransferase
Rahaq2_2904
Rahaq2_2906
Rahaq2_2907
Rahaq2_2908
Rahaq2_2909
DNA polymerase III, theta subunit
Rahnella aquatilis CIP 78.65 = ATCC 33071
rsmF ERS451419_04092
ribosomal RNA small subunit methyltransferase F
protein
ERS451419_04094
pphA ERS451419_04095
serine/threonine protein phosphatase
yebZ ERS451419_04099
ERS451419_04100
ERS451419_04102
putative carbon−nitrogen hydrolase
Escherichia coli NCTC9001
BZ16_1085
ferritin−like domain protein
BZ16_1087
copper resistance D family protein
BZ16_1088
hypothetical protein
BZ16_1089
hypothetical protein
BZ16_1090
transposase, Mutator family protein
BZ16_1091
putative membrane protein
BZ16_1092
hypothetical protein
Yersinia pseudotuberculosis PB1/+
AX02_26280
lytic enzyme
AX02_26290
hypothetical protein
AX02_48620
lytic enzyme
AX02_26300
exodeoxyribonuclease VII
AX02_48625
integrase
AX02_26330
hypothetical protein
AX02_26340
hypothetical protein
AX02_26350
AX02_26360
carbon−nitrogen hydrolase
Salmonella enterica subsp. enterica serovar Typhimurium str. USDA−ARS−USMARC−1899

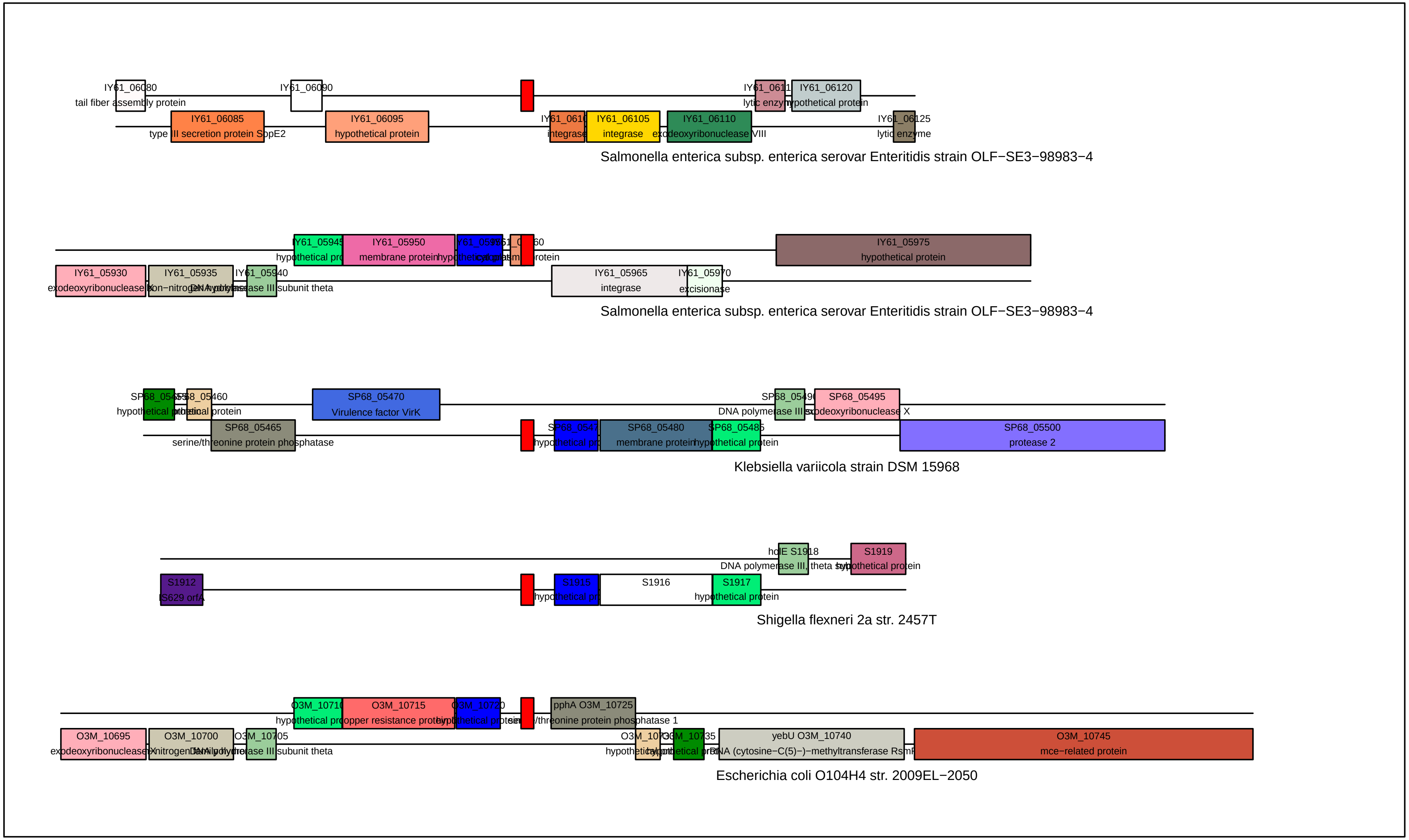
IY61_06080
tail fiber assembly protein
IY61_06090
IY61_06120
hypothetical protein
IY61_06085
type III secretion protein SopE2
IY61_06095
hypothetical protein
integrase
IY61_06105
integrase
IY61_06110
exodeoxyribonuclease VIII
IY61_06125
lytic enzyme
Salmonella enterica subsp. enterica serovar Enteritidis strain OLF−SE3−98983−4
IY61_05950
membrane protein
IY61_05975
hypothetical protein
IY61_05930
exodeoxyribonuclease X
IY61_05935
IY61_05940
DNA polymerase III subunit theta
IY61_05965
integrase
IY61_05970
excisionase
Salmonella enterica subsp. enterica serovar Enteritidis strain OLF−SE3−98983−4
SP68_05460
hypothetical protein
SP68_05470
Virulence factor VirK
SP68_05495
exodeoxyribonuclease X
DNA polymerase III
SP68_05465
serine/threonine protein phosphatase
SP68_05480
membrane protein
hypothetical protein
SP68_05500
protease 2
Klebsiella variicola strain DSM 15968
holE S1918
DNA polymerase III, theta subunit
S1919
hypothetical protein
S1912
IS629 orfA
S1915
S1916
S1917
hypothetical protein
Shigella flexneri 2a str. 2457T
O3M_10715
copper resistance protein
hypothetical protein
pphA O3M_10725
serine/threonine protein phosphatase 1
O3M_10695
exodeoxyribonuclease X
O3M_10700
O3M_10705
DNA polymerase III subunit theta
hypothetical protein
yebU O3M_10740
rRNA (cytosine−C(5)−)−methyltransferase RsmF
O3M_10745
mce−related protein
Escherichia coli O104H4 str. 2009EL−2050

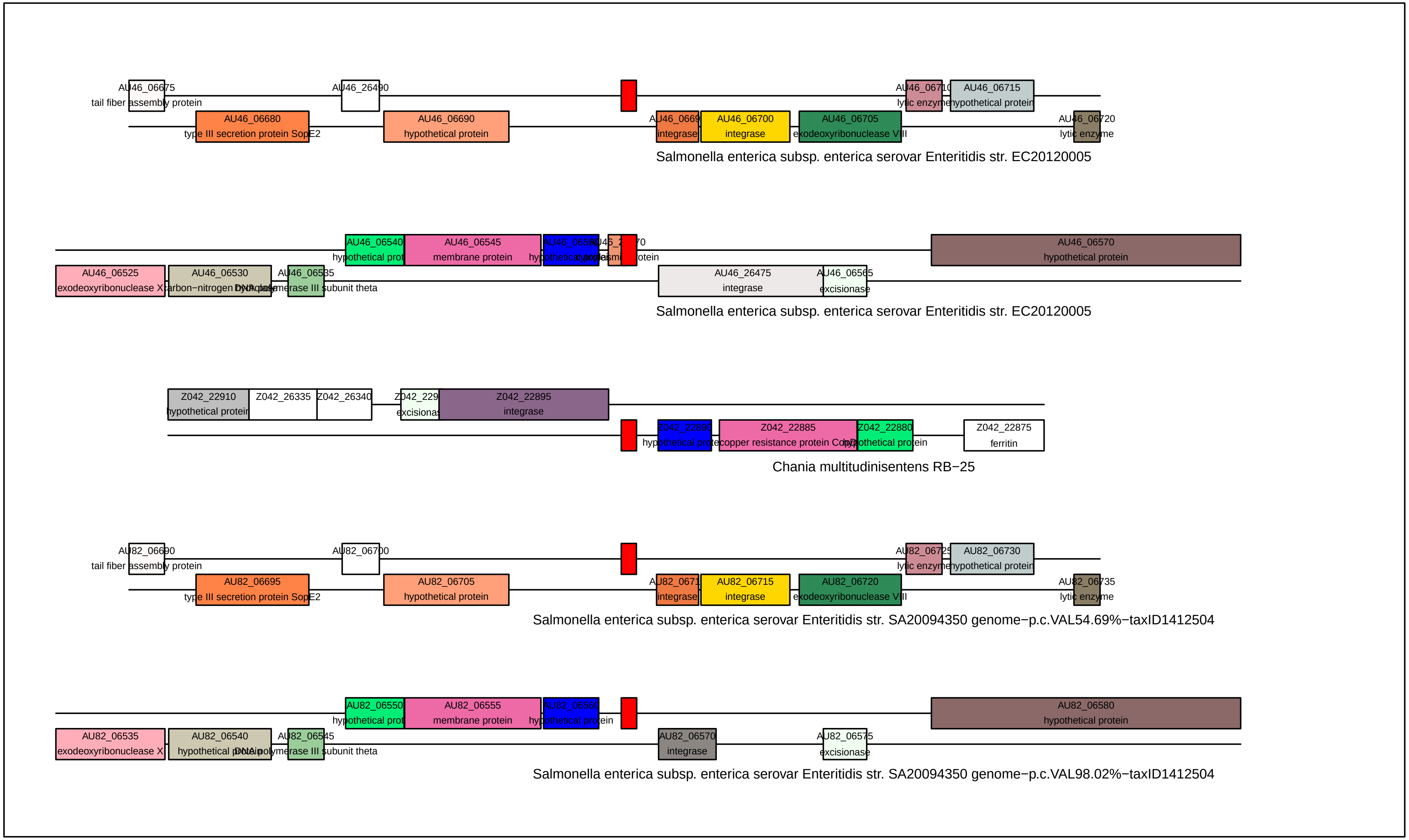

AU46_06675
tail fiber assembly protein
AU46_26490
AU46_06715
hypothetical protein
AU46_06680
type III secretion protein SopE2
AU46_06690
hypothetical protein
integrase
AU46_06700
integrase
AU46_06705
exodeoxyribonuclease VIII
lytic enzyme
AU46_06720
lytic enzyme
Salmonella enterica subsp. enterica serovar Enteritidis str. EC20120005
AU46_06540
AU46_06545
membrane protein
AU46_06570
hypothetical protein
AU46_06525
exodeoxyribonuclease X
AU46_06530
DNA polymerase III subunit theta
AU46_26475
integrase
AU46_06565
excisionase
Salmonella enterica subsp. enterica serovar Enteritidis str. EC20120005
Z042_22910
hypothetical protein
Z042_26335
Z042_26340
Z042_22895
integrase
Z042_22885
copper resistance protein CopC
Z042_22880
hypothetical protein
Z042_22875
ferritin
Chania multitudinisentens RB−25
AU82_06690
tail fiber assembly protein
AU82_06700
AU82_06725
AU82_06730
hypothetical protein
AU82_06695
type III secretion protein SopE2
AU82_06705
hypothetical protein
integrase
AU82_06715
integrase
AU82_06720
exodeoxyribonuclease VIII
lytic enzyme
AU82_06735
lytic enzyme
Salmonella enterica subsp. enterica serovar Enteritidis str. SA20094350 genome−p.c.VAL54.69%−taxID1412504
AU82_06550
AU82_06555
membrane protein
hypothetical protein
AU82_06580
hypothetical protein
AU82_06535
exodeoxyribonuclease X
AU82_06540
hypothetical protein
AU82_06545
DNA polymerase III subunit theta
AU82_06570
integrase
AU82_06575
excisionase
Salmonella enterica subsp. enterica serovar Enteritidis str. SA20094350 genome−p.c.VAL98.02%−taxID1412504

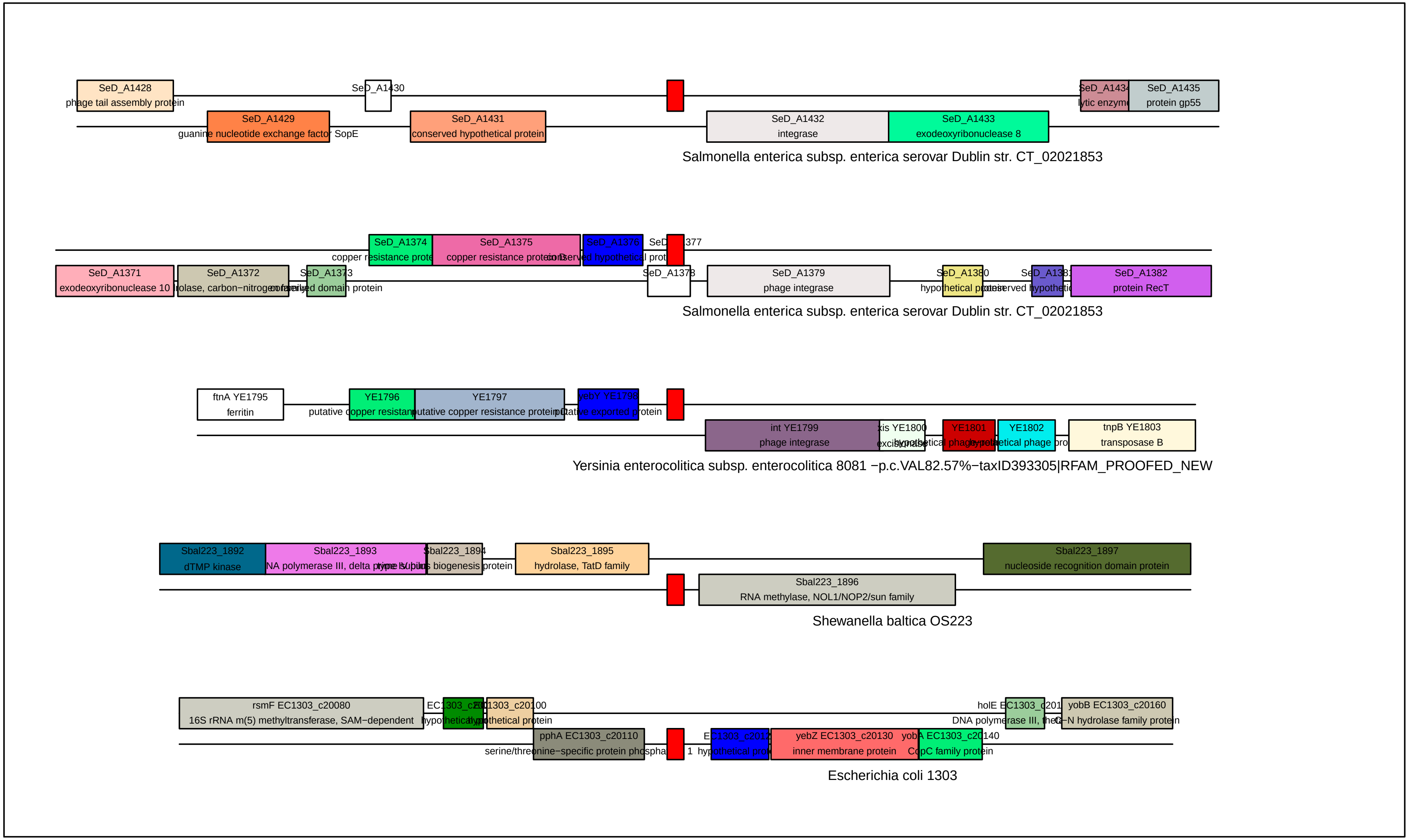
SeD_A1428
phage tail assembly protein
SeD_A1430
SeD_A1435
protein gp55
SeD_A1429
guanine nucleotide exchange factor SopE
SeD_A1431
conserved hypothetical protein
SeD_A1432
integrase
SeD_A1433
exodeoxyribonuclease 8
Salmonella enterica subsp. enterica serovar Dublin str. CT_02021853
SeD_A1374
SeD_A1375
copper resistance protein
SeD_A1371
exodeoxyribonuclease 10
SeD_A1372
SeD_A1373
SeD_A1378
SeD_A1379
phage integrase
SeD_A1382
protein RecT
Salmonella enterica subsp. enterica serovar Dublin str. CT_02021853
ftnA YE1795
ferritin
YE1796
YE1797
putative copper resistance protein
int YE1799
phage integrase
YE1801
YE1802
tnpB YE1803
transposase B
Yersinia enterocolitica subsp. enterocolitica 8081 −p.c.VAL82.57%−taxID393305|RFAM_PROOFED_NEW
Sbal223_1892
dTMP kinase
Sbal223_1893
Sbal223_1895
hydrolase, TatD family
Sbal223_1897
nucleoside recognition domain protein
Sbal223_1896
RNA methylase, NOL1/NOP2/sun family
Shewanella baltica OS223
rsmF EC1303_c20080
16S rRNA m(5) methyltransferase, SAM−dependent
yobB EC1303_c20160
C−N hydrolase family protein
pphA EC1303_c20110
yebZ EC1303_c20130
inner membrane protein
CopC family protein
Escherichia coli 1303

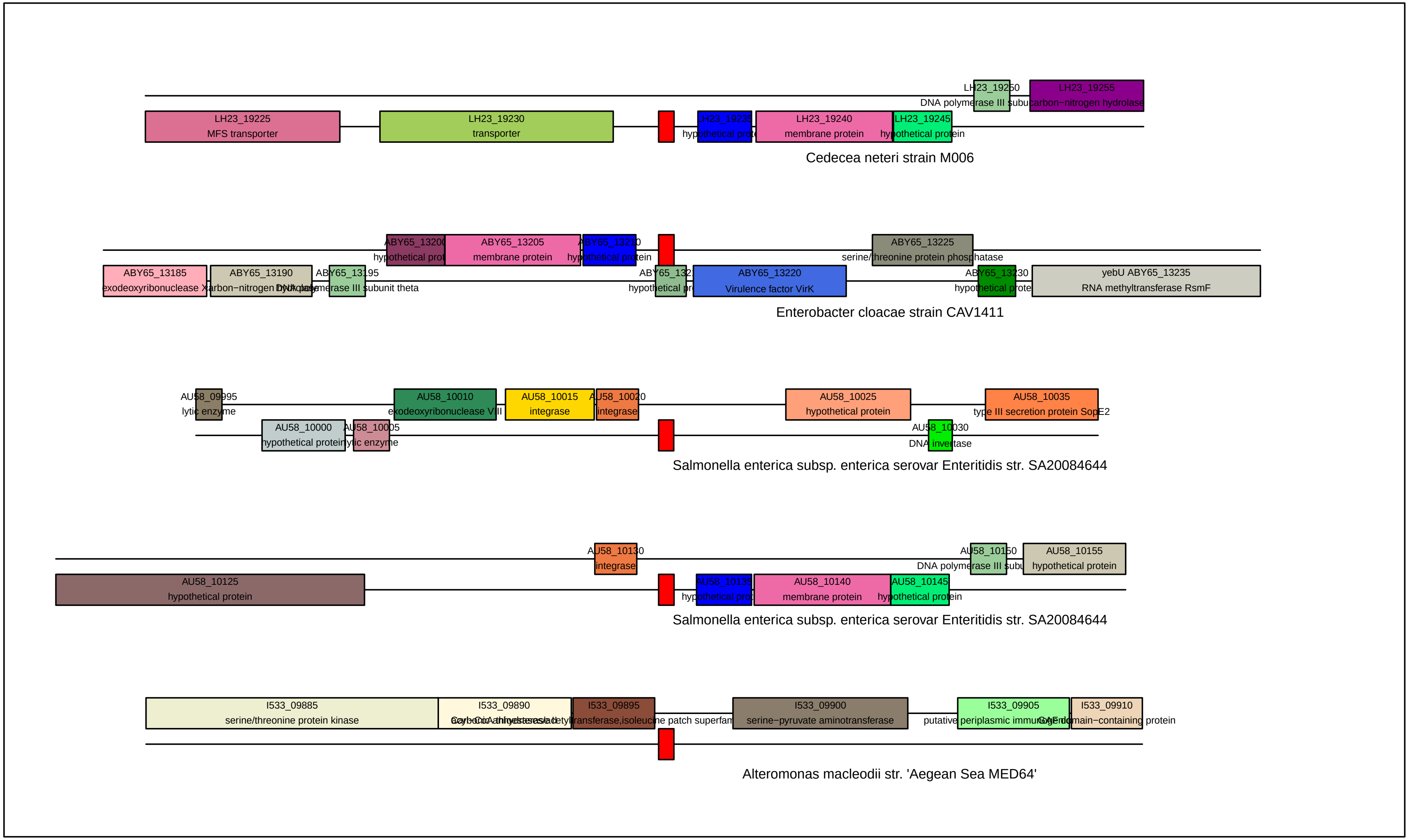

LH23_19225
MFS transporter
LH23_19230
transporter
LH23_19240
membrane protein
LH23_19245
hypothetical protein
LH23_19255
carbon−nitrogen hydrolase
DNA polymerase III subu
Cedecea neteri strain M006
ABY65_13185
exodeoxyribonuclease X
ABY65_13190
ABY65_13195
DNA polymerase III subunit theta
ABY65_13205
membrane protein
ABY65_13210
hypothetical protein
ABY65_13220
Virulence factor VirK
ABY65_13225
serine/threonine protein phosphatase
ABY65_13230
yebU ABY65_13235
RNA methyltransferase RsmF
Enterobacter cloacae strain CAV1411
AU58_09995
lytic enzyme
AU58_10000
hypothetical protein
AU58_10005
AU58_10010
exodeoxyribonuclease VIII
AU58_10015
integrase
AU58_10020
integrase
AU58_10025
hypothetical protein
AU58_10030
DNA invertase
AU58_10035
type III secretion protein SopE2
Salmonella enterica subsp. enterica serovar Enteritidis str. SA20084644
AU58_10125
hypothetical protein
AU58_10130
integrase
AU58_10140
membrane protein
AU58_10145
hypothetical protein
AU58_10150
DNA polymerase III subu
AU58_10155
hypothetical protein
Salmonella enterica subsp. enterica serovar Enteritidis str. SA20084644
I533_09885
serine/threonine protein kinase
I533_09890
I533_09895
I533_09900
serine−pyruvate aminotransferase
I533_09905
I533_09910
Alteromonas macleodii str. 'Aegean Sea MED64'

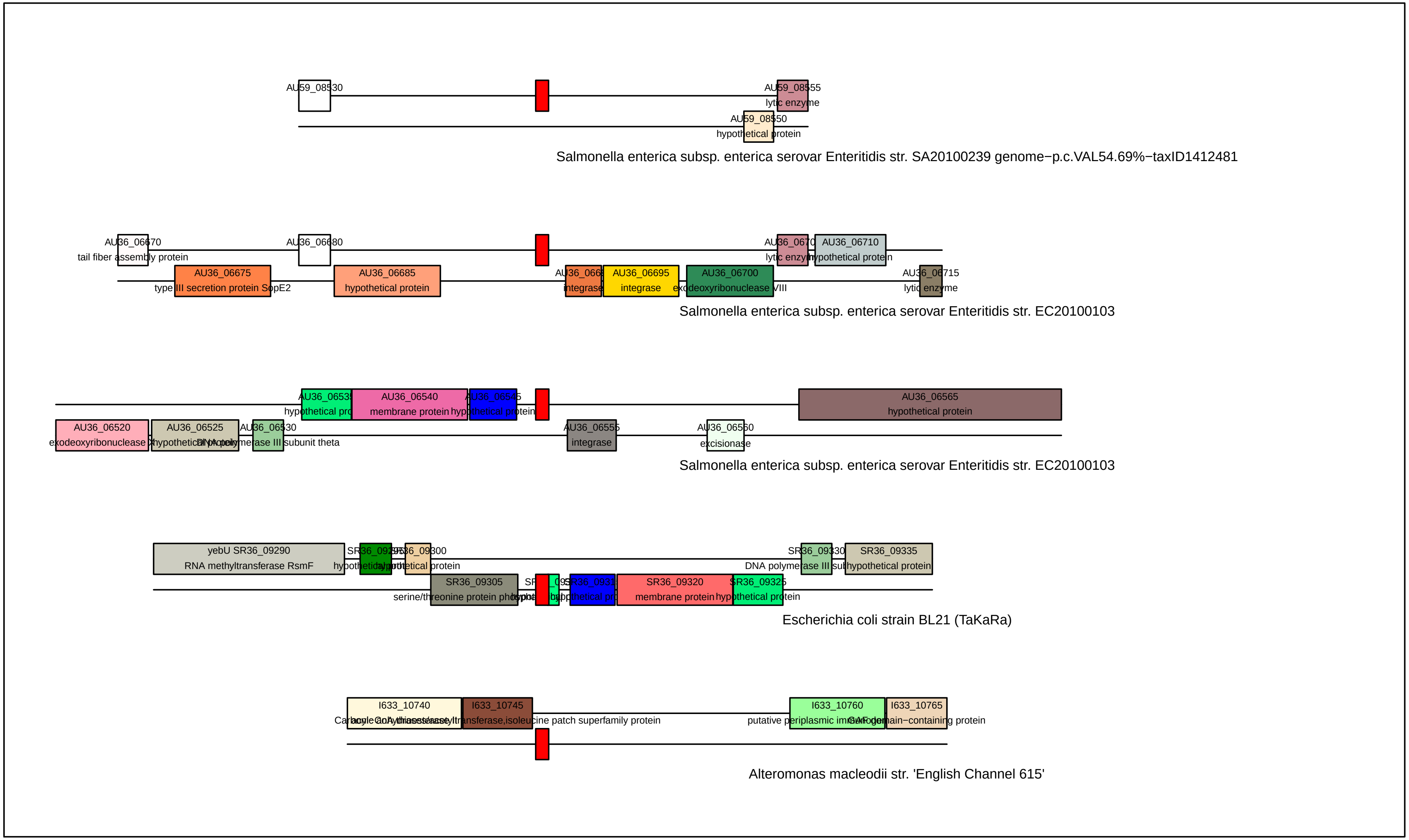

AU59_08530
AU59_08555
lytic enzyme
AU59_08550
hypothetical protein
Salmonella enterica subsp. enterica serovar Enteritidis str. SA20100239 genome−p.c.VAL54.69%−taxID1412481
AU36_06670
tail fiber assembly protein
AU36_06680
AU36_06710
hypothetical protein
AU36_06675
type III secretion protein SopE2
AU36_06685
hypothetical protein
integrase
AU36_06695
integrase
AU36_06700
exodeoxyribonuclease VIII
AU36_06715
lytic enzyme
Salmonella enterica subsp. enterica serovar Enteritidis str. EC20100103
AU36_06540
membrane protein
AU36_06545
AU36_06565
hypothetical protein
AU36_06520
exodeoxyribonuclease X
AU36_06525
AU36_06530
DNA polymerase III subunit theta
AU36_06555
integrase
AU36_06560
excisionase
Salmonella enterica subsp. enterica serovar Enteritidis str. EC20100103
yebU SR36_09290
RNA methyltransferase RsmF
SR36_09300
hypothetical protein
SR36_09330
SR36_09335
hypothetical protein
SR36_09305
SR36_09320
membrane protein
SR36_09325
hypothetical protein
Escherichia coli strain BL21 (TaKaRa)
I633_10740
I633_10745
isoleucine patch superfamily protein
I633_10760
putative periplasmic
I633_10765
domain−containing protein
Alteromonas macleodii str. 'English Channel 615'

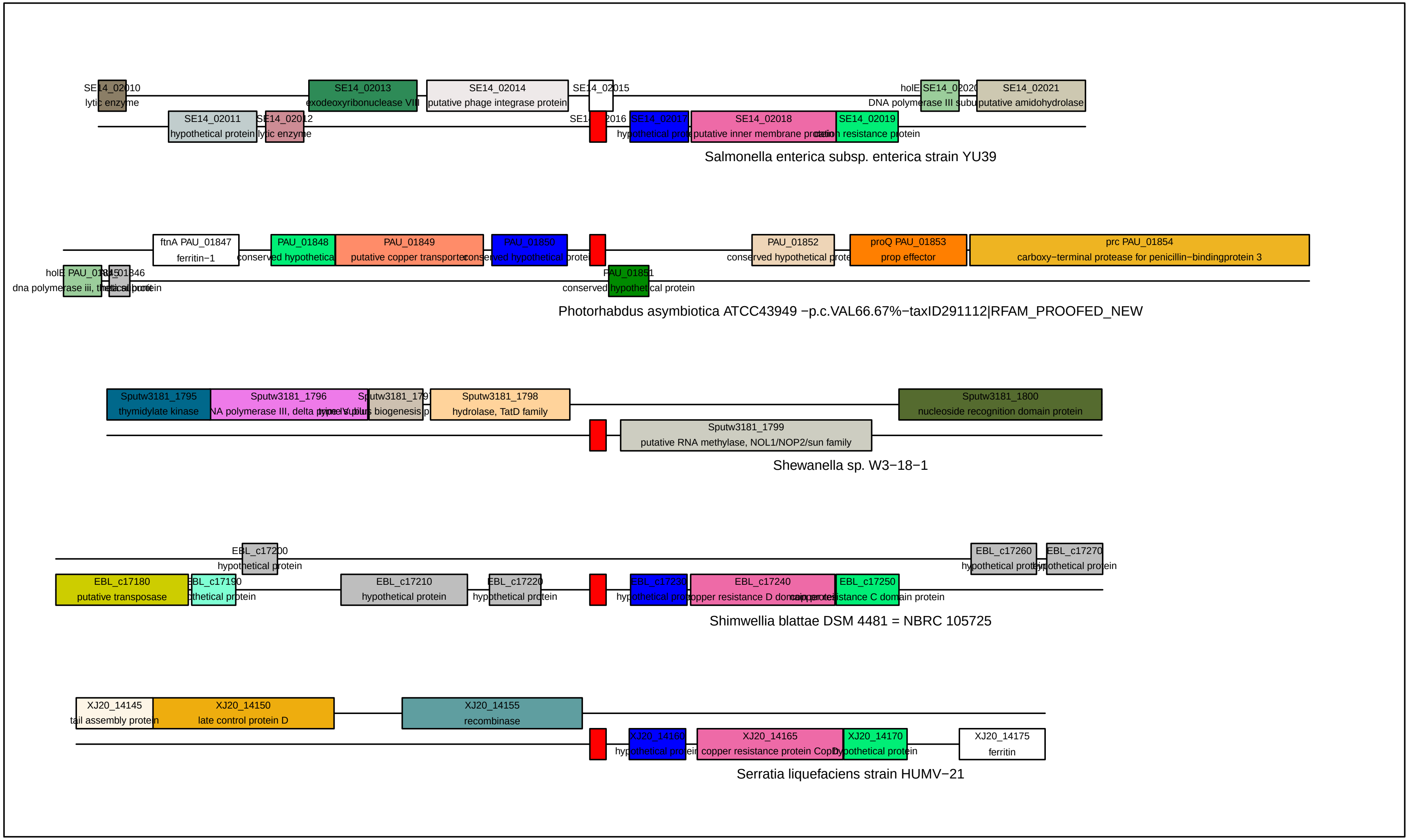

SE14_02010
lytic enzyme
SE14_02011
hypothetical protein
SE14_02013
exodeoxyribonuclease VIII
SE14_02014
putative phage integrase protein
SE14_02015
SE14_02017
SE14_02018
putative inner membrane protein
SE14_02019
resistance protein
holE
DNA polymerase III
SE14_02021
putative amidohydrolase
Salmonella enterica subsp. enterica strain YU39
ftnA PAU_01847
ferritin−1
PAU_01848
PAU_01849
putative copper transporter
PAU_01850
PAU_01851
conserved hypothetical protein
PAU_01852
proQ PAU_01853
prop effector
prc PAU_01854
carboxy−terminal protease for penicillin−bindingprotein 3
holE
dna polymerase iii, theta subunit
Photorhabdus asymbiotica ATCC43949 −p.c.VAL66.67%−taxID291112|RFAM_PROOFED_NEW
Sputw3181_1795
thymidylate kinase
Sputw3181_1796
Sputw3181_1798
hydrolase, TatD family
Sputw3181_1800
nucleoside recognition domain protein
Sputw3181_1799
putative RNA methylase, NOL1/NOP2/sun family
Shewanella sp. W3−18−1
EBL_c17200
hypothetical protein
EBL_c17260
EBL_c17270
hypothetical protein
EBL_c17180
putative transposase
EBL_c17210
hypothetical protein
EBL_c17220
hypothetical protein
EBL_c17240
EBL_c17250
Shimwellia blattae DSM 4481 = NBRC 105725
XJ20_14145
tail assembly protein
XJ20_14150
late control protein D
XJ20_14155
recombinase
XJ20_14165
XJ20_14170
hypothetical protein
XJ20_14175
ferritin
Serratia liquefaciens strain HUMV−21

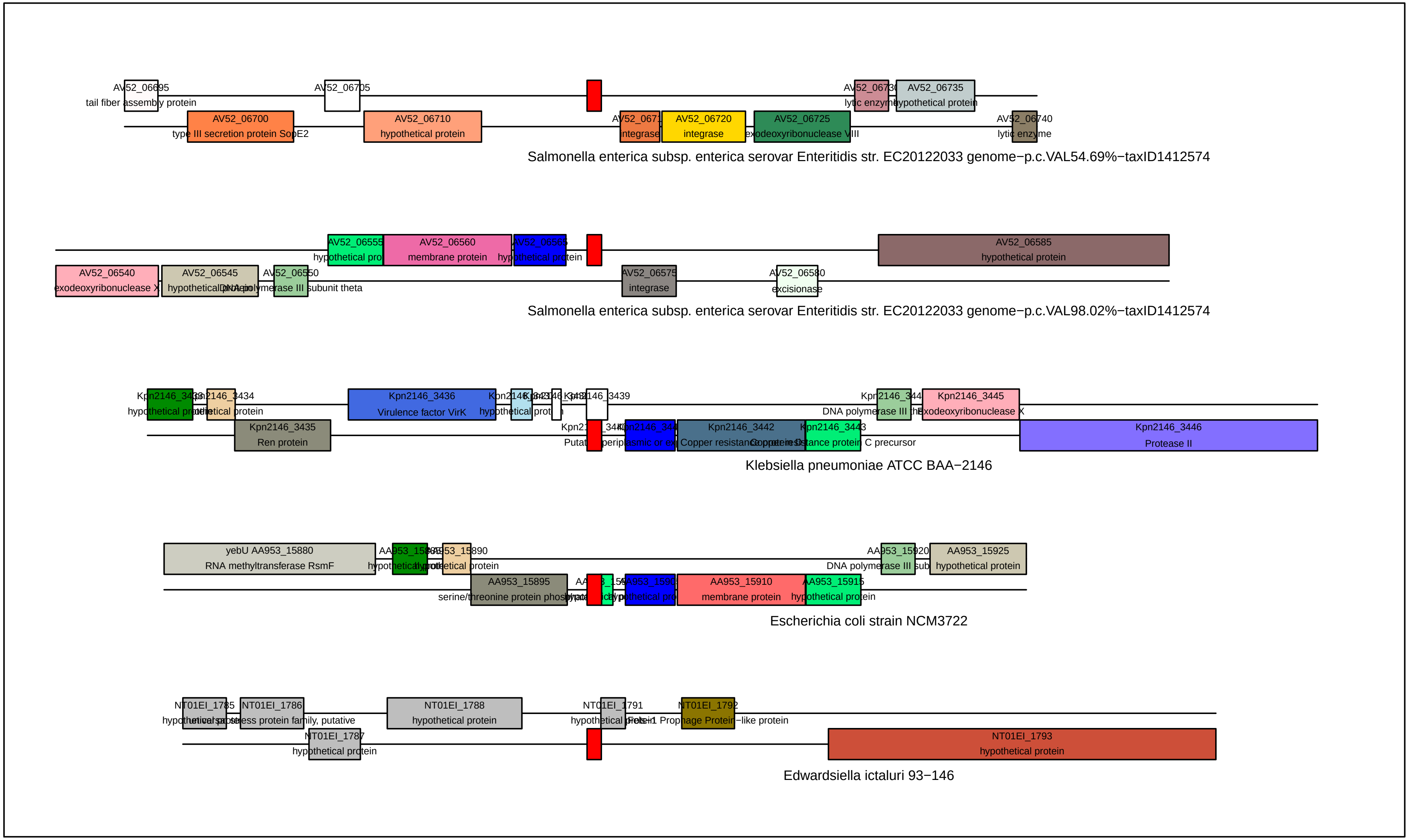

AV52_06695
tail fiber assembly protein
AV52_06705
AV52_06735
hypothetical protein
AV52_06700
type III secretion protein SopE2
AV52_06710
hypothetical protein
AV52_06720
integrase
AV52_06725
exodeoxyribonuclease VIII
AV52_06740
lytic enzyme
Salmonella enterica subsp. enterica serovar Enteritidis str. EC20122033 genome−p.c.VAL54.69%−taxID1412574
AV52_06555
AV52_06560
membrane protein
AV52_06585
hypothetical protein
AV52_06540
exodeoxyribonuclease X
AV52_06545
AV52_06550
DNA polymerase III subunit theta
AV52_06575
integrase
AV52_06580
excisionase
Salmonella enterica subsp. enterica serovar Enteritidis str. EC20122033 genome−p.c.VAL98.02%−taxID1412574
Kpn2146_3434
Kpn2146_3436
Virulence factor VirK
Kpn2146_3439
Kpn2146_3445
Exodeoxyribonuclease X
DNA polymerase III
Kpn2146_3435
Ren protein
Kpn2146_3442
Kpn2146_3443
C precursor
Kpn2146_3446
Protease II
Klebsiella pneumoniae ATCC BAA−2146
yebU AA953_15880
RNA methyltransferase RsmF
AA953_15890
AA953_15920
AA953_15925
hypothetical protein
AA953_15895
serine/threonine protein phosphatase
AA953_15910
membrane protein
AA953_15915
hypothetical protein
Escherichia coli strain NCM3722
NT01EI_1785
NT01EI_1786
NT01EI_1788
hypothetical protein
NT01EI_1791
NT01EI_1792
NT01EI_1787
hypothetical protein
NT01EI_1793
hypothetical protein
Edwardsiella ictaluri 93−146

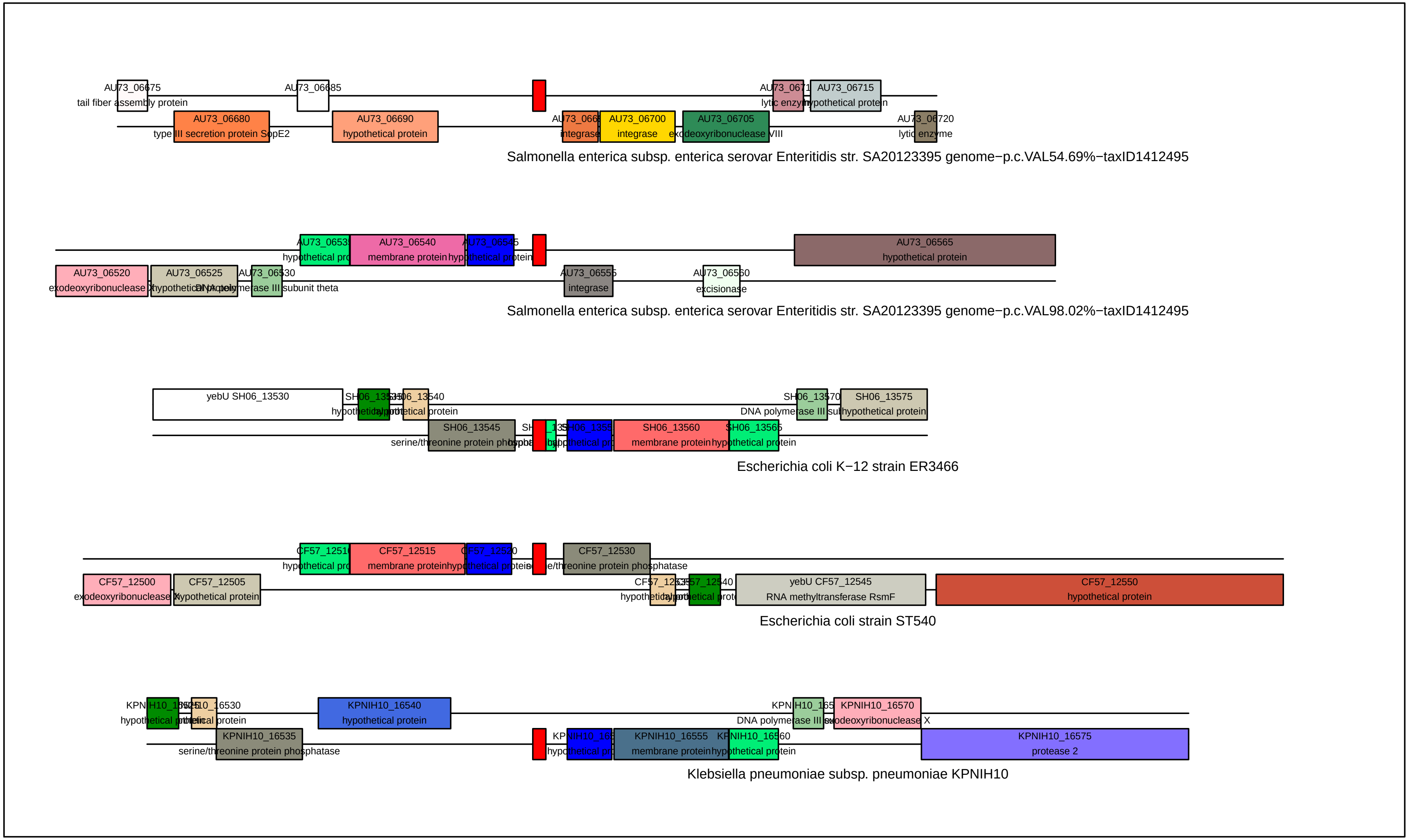

AU73_06675
tail fiber assembly protein
AU73_06685
AU73_06715
hypothetical protein
AU73_06680
type III secretion protein SopE2
AU73_06690
hypothetical protein
integrase
AU73_06700
integrase
AU73_06705
exodeoxyribonuclease VIII
AU73_06720
lytic enzyme
Salmonella enterica subsp. enterica serovar Enteritidis str. SA20123395 genome−p.c.VAL54.69%−taxID1412495
AU73_06540
membrane protein
AU73_06565
hypothetical protein
AU73_06520
exodeoxyribonuclease X
AU73_06525
AU73_06530
subunit theta
AU73_06555
integrase
AU73_06560
excisionase
Salmonella enterica subsp. enterica serovar Enteritidis str. SA20123395 genome−p.c.VAL98.02%−taxID1412495
yebU SH06_13530
SH06_13570
DNA polymerase III
SH06_13575
hypothetical protein
SH06_13545
SH06_13560
membrane protein
SH06_13565
hypothetical protein
Escherichia coli K−12 strain ER3466
CF57_12515
membrane protein
CF57_12530
serine/threonine protein phosphatase
CF57_12500
exodeoxyribonuclease X
CF57_12505
hypothetical protein
CF57_12540
yebU CF57_12545
RNA methyltransferase RsmF
CF57_12550
hypothetical protein
Escherichia coli strain ST540
KPNIH10_16540
hypothetical protein
KPNIH10_16570
exodeoxyribonuclease X
DNA polymerase III
KPNIH10_16535
serine/threonine protein phosphatase
KPNIH10_16555
membrane protein
KPNIH10_16560
hypothetical protein
KPNIH10_16575
protease 2
Klebsiella pneumoniae subsp. pneumoniae KPNIH10

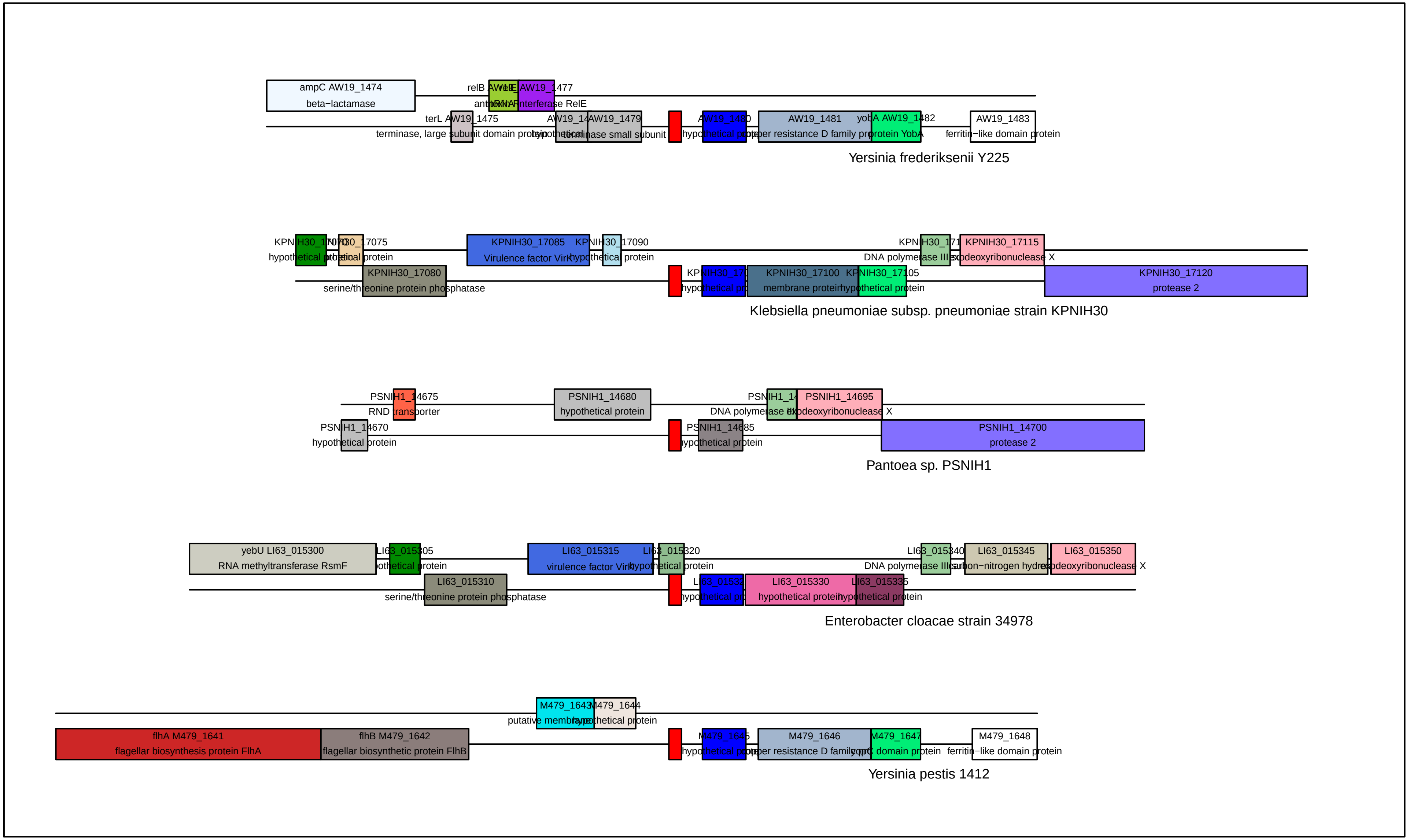

ampC AW19_1474
beta−lactamase
terL AW19_1475
AW19_1479
AW19_1481
AW19_1483
ferritin−like domain protein
Yersinia frederiksenii Y225
KPNIH30_17075
KPNIH30_17080
serine/threonine protein phosphatase
KPNIH30_17085
KPNIH30_17100
membrane protein
KPNIH30_17105
KPNIH30_17115
KPNIH30_17120
protease 2
Klebsiella pneumoniae subsp. pneumoniae strain KPNIH30
PSNIH1_14670
PSNIH1_14675
RND transporter
PSNIH1_14680
hypothetical protein
PSNIH1_14685
PSNIH1_14695
PSNIH1_14700
protease 2
Pantoea sp. PSNIH1
yebU LI63_015300
RNA methyltransferase RsmF
LI63_015305
LI63_015310
serine/threonine protein phosphatase
LI63_015315
LI63_015320
LI63_015330
LI63_015335
LI63_015340
LI63_015345
LI63_015350
Enterobacter cloacae strain 34978
M479_1643
M479_1644
flhA M479_1641
flagellar biosynthesis protein FlhA
flhB M479_1642
flagellar biosynthetic protein FlhB
M479_1646
M479_1647
M479_1648
ferritin−like domain protein
Yersinia pestis 1412

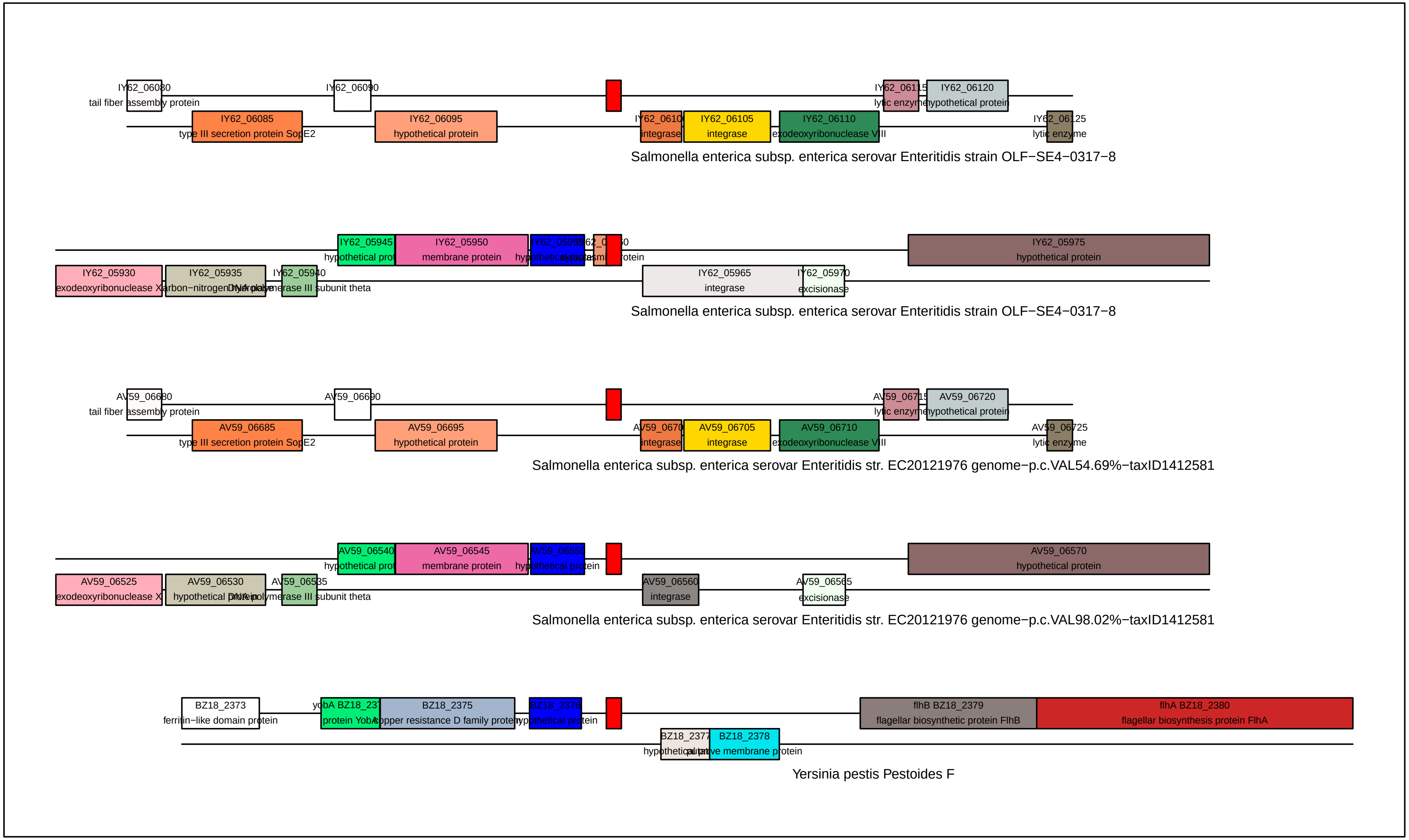
IY62_06080
tail fiber assembly protein
IY62_06085
type III secretion protein SopE2
IY62_06090
IY62_06095
hypothetical protein
integrase
IY62_06105
integrase
IY62_06110
exodeoxyribonuclease VIII
lytic enzyme
IY62_06120
hypothetical protein
IY62_06125
lytic enzyme
Salmonella enterica subsp. enterica serovar Enteritidis strain OLF−SE4−0317−8
IY62_05930
exodeoxyribonuclease X
IY62_05935
IY62_05940
DNA polymerase III subunit theta
IY62_05945
IY62_05950
membrane protein
IY62_05965
integrase
IY62_05970
excisionase
IY62_05975
hypothetical protein
Salmonella enterica subsp. enterica serovar Enteritidis strain OLF−SE4−0317−8
AV59_06680
tail fiber assembly protein
AV59_06685
type III secretion protein SopE2
AV59_06690
AV59_06695
hypothetical protein
integrase
AV59_06705
integrase
AV59_06710
exodeoxyribonuclease VIII
lytic enzyme
AV59_06720
hypothetical protein
AV59_06725
lytic enzyme
Salmonella enterica subsp. enterica serovar Enteritidis str. EC20121976 genome−p.c.VAL54.69%−taxID1412581
AV59_06525
exodeoxyribonuclease X
AV59_06530
AV59_06535
DNA polymerase III subunit theta
AV59_06540
AV59_06545
membrane protein
AV59_06550
hypothetical protein
AV59_06560
integrase
AV59_06565
excisionase
AV59_06570
hypothetical protein
Salmonella enterica subsp. enterica serovar Enteritidis str. EC20121976 genome−p.c.VAL98.02%−taxID1412581
BZ18_2373
ferritin−like domain protein
BZ18_2375
copper resistance D family protein
hypothetical protein
flhB BZ18_2379
flagellar biosynthetic protein FlhB
flhA BZ18_2380
flagellar biosynthesis protein FlhA
BZ18_2378
putative membrane protein
Yersinia pestis Pestoides F

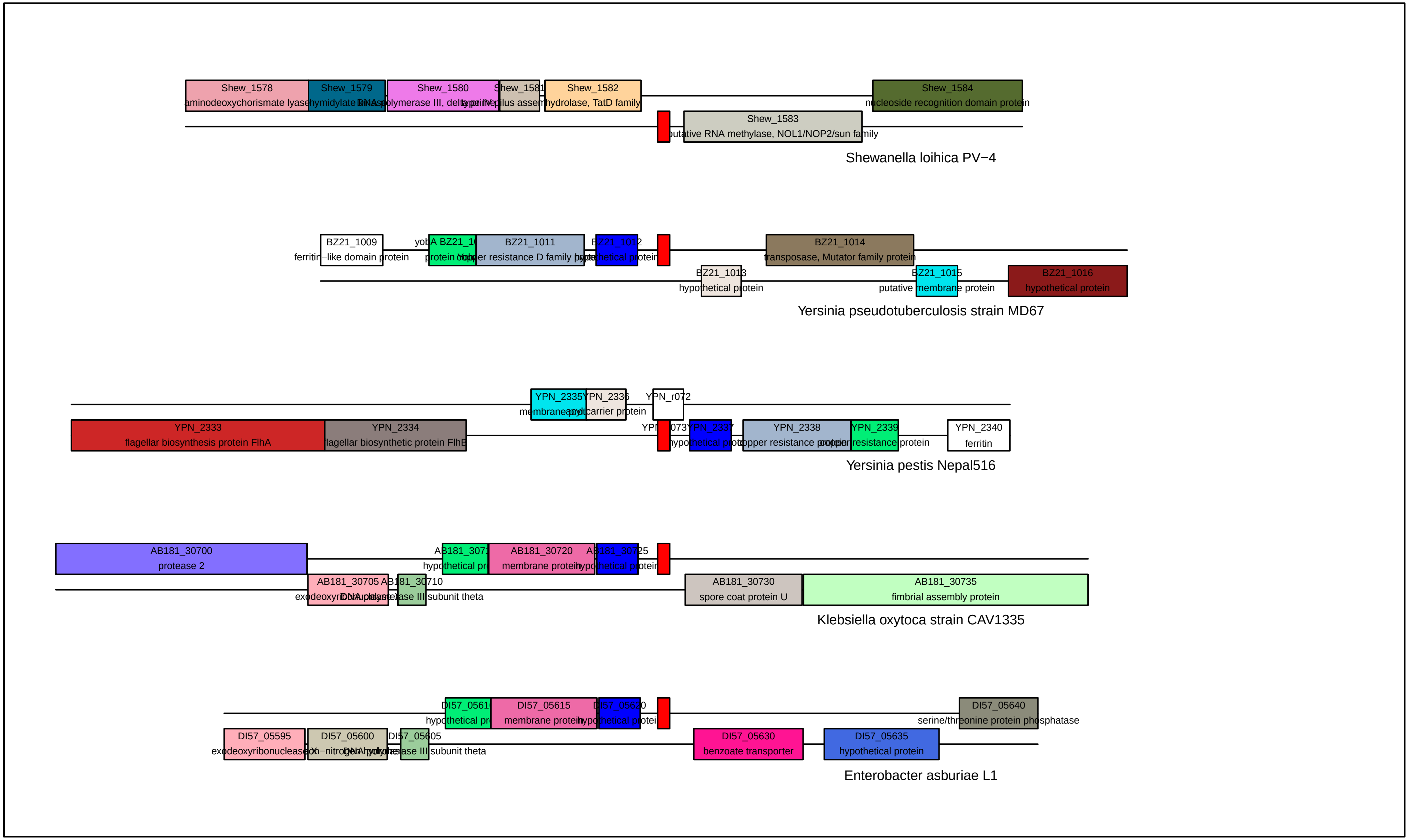

Shew_1578
Shew_1579
Shew_1580
Shew_1582
hydrolase, TatD family
Shew_1583
putative RNA methylase, NOL1/NOP2/sun family
Shew_1584
nucleoside recognition domain protein
Shewanella loihica PV−4
BZ21_1009
ferritin−like domain protein
BZ21_1011
BZ21_1014
transposase, Mutator family protein
BZ21_1016
hypothetical protein
Yersinia pseudotuberculosis strain MD67
YPN_2333
flagellar biosynthesis protein FlhA
YPN_2334
YPN_2338
YPN_2340
ferritin
Yersinia pestis Nepal516
AB181_30700
protease 2
AB181_30720
membrane protein
AB181_30730
spore coat protein U
AB181_30735
fimbrial assembly protein
Klebsiella oxytoca strain CAV1335
DI57_05595
DI57_05600
DI57_05615
membrane protein
DI57_05630
benzoate transporter
DI57_05635
hypothetical protein
DI57_05640
Enterobacter asburiae L1

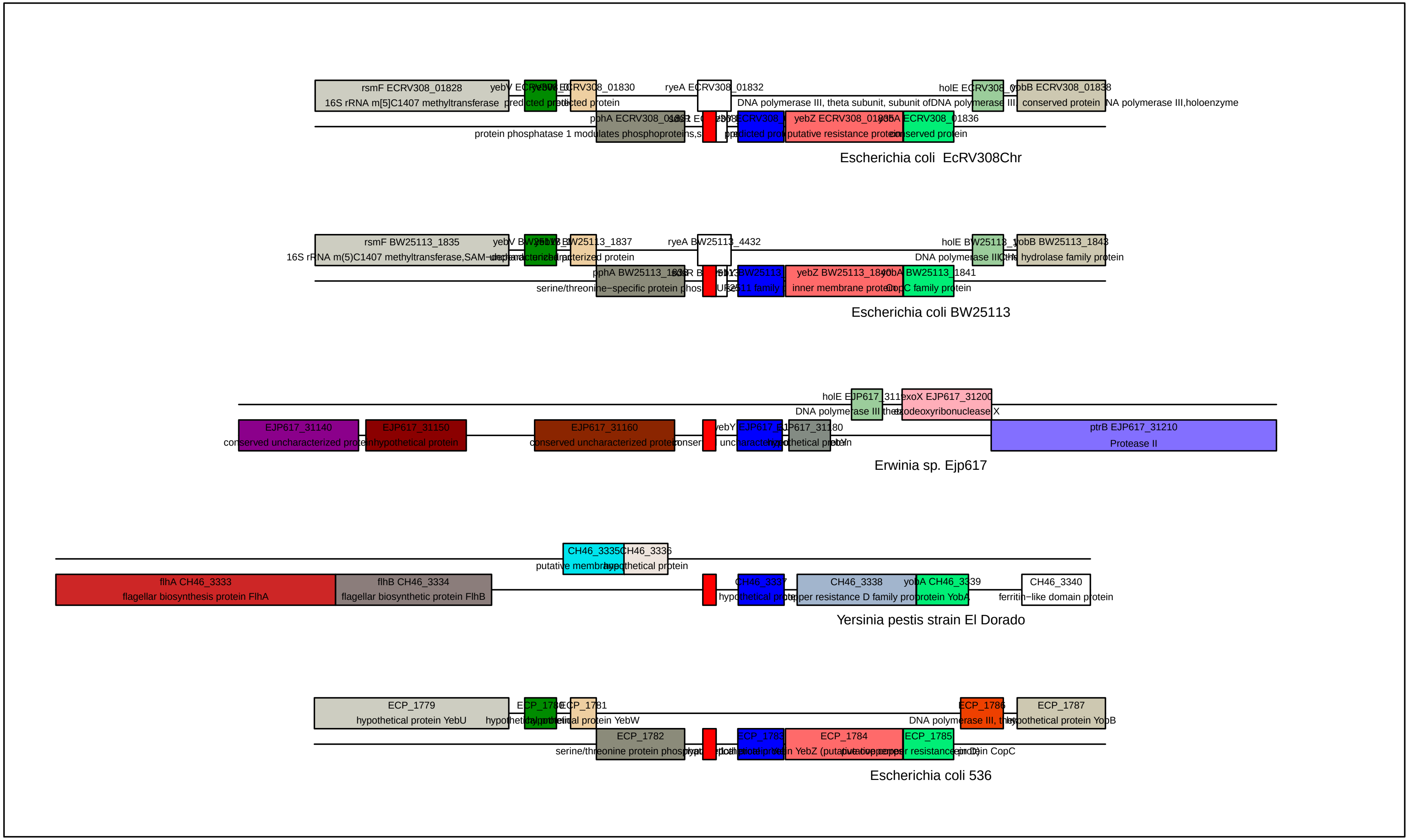
rsmF ECRV308_01828
16S rRNA m[5]C1407 methyltransferase
ryeA ECRV308_01832
DNA polymerase III, theta subunit
conserved protein
Escherichia coli EcRV308Chr
rsmF BW25113_1835
ryeA BW25113_4432
yebZ BW25113_1840
inner membrane protein
Escherichia coli BW25113
exoX EJP617_31200
EJP617_31140
EJP617_31150
hypothetical protein
EJP617_31160
ptrB EJP617_31210
Protease II
Erwinia sp. Ejp617
flhA CH46_3333
flagellar biosynthesis protein FlhA
flhB CH46_3334
flagellar biosynthetic protein FlhB
CH46_3338
CH46_3340
ferritin−like domain protein
Yersinia pestis strain El Dorado
ECP_1779
hypothetical protein YebU
ECP_1782
ECP_1784
ECP_1785
ECP_1787
Escherichia coli 536

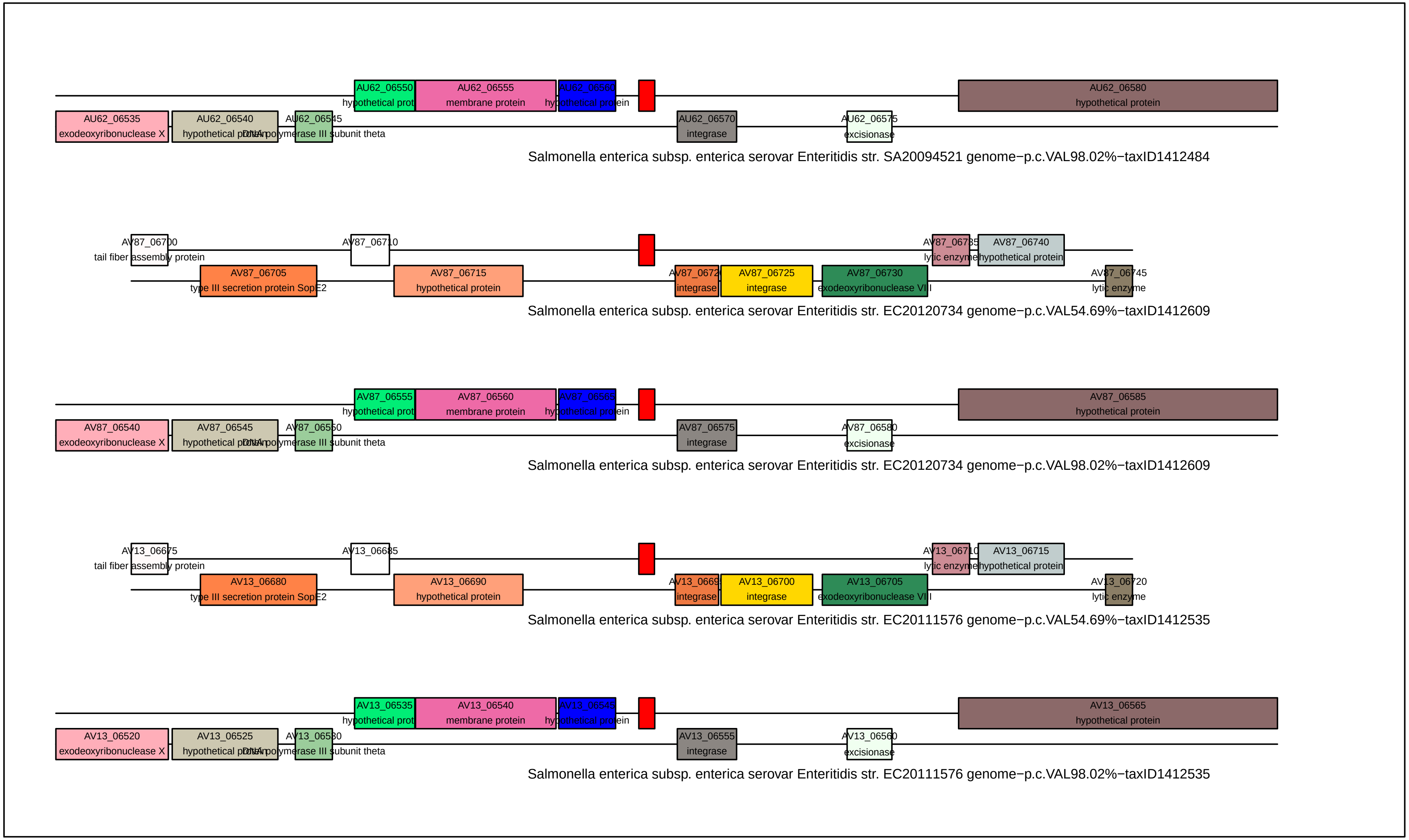
AU62_06550
hypothetical prot
AU62_06555
membrane protein
AU62_06560
hypothetical protein
AU62_06580
hypothetical protein
AU62_06535
exodeoxyribonuclease X
AU62_06540
hypothetical protein
AU62_06545
DNA polymerase III subunit theta
AU62_06570
integrase
AU62_06575
excisionase
Salmonella enterica subsp. enterica serovar Enteritidis str. SA20094521 genome−p.c.VAL98.02%−taxID1412484
AV87_06700
tail fiber assembly protein
AV87_06710
AV87_06735
lytic enzyme
AV87_06740
hypothetical protein
AV87_06705
type III secretion protein SopE2
AV87_06715
hypothetical protein
AV87_0672
integrase
AV87_06725
integrase
AV87_06730
exodeoxyribonuclease VIII
AV87_06745
lytic enzyme
Salmonella enterica subsp. enterica serovar Enteritidis str. EC20120734 genome−p.c.VAL54.69%−taxID1412609
AV87_06555
hypothetical prot
AV87_06560
membrane protein
AV87_06565
hypothetical protein
AV87_06585
hypothetical protein
AV87_06540
exodeoxyribonuclease X
AV87_06545
hypothetical protein
AV87_06550
DNA polymerase III subunit theta
AV87_06575
integrase
AV87_06580
excisionase
Salmonella enterica subsp. enterica serovar Enteritidis str. EC20120734 genome−p.c.VAL98.02%−taxID1412609
AV13_06675
tail fiber assembly protein
AV13_06685
AV13_06710
lytic enzyme
AV13_06715
hypothetical protein
AV13_06680
type III secretion protein SopE2
AV13_06690
hypothetical protein
AV13_0669
integrase
AV13_06700
integrase
AV13_06705
exodeoxyribonuclease VIII
AV13_06720
lytic enzyme
Salmonella enterica subsp. enterica serovar Enteritidis str. EC20111576 genome−p.c.VAL54.69%−taxID1412535
AV13_06535
hypothetical prot
AV13_06540
membrane protein
AV13_06545
hypothetical protein
AV13_06565
hypothetical protein
AV13_06520
exodeoxyribonuclease X
AV13_06525
hypothetical protein
AV13_06530
DNA polymerase III subunit theta
AV13_06555
integrase
AV13_06560
excisionase
Salmonella enterica subsp. enterica serovar Enteritidis str. EC20111576 genome−p.c.VAL98.02%−taxID1412535

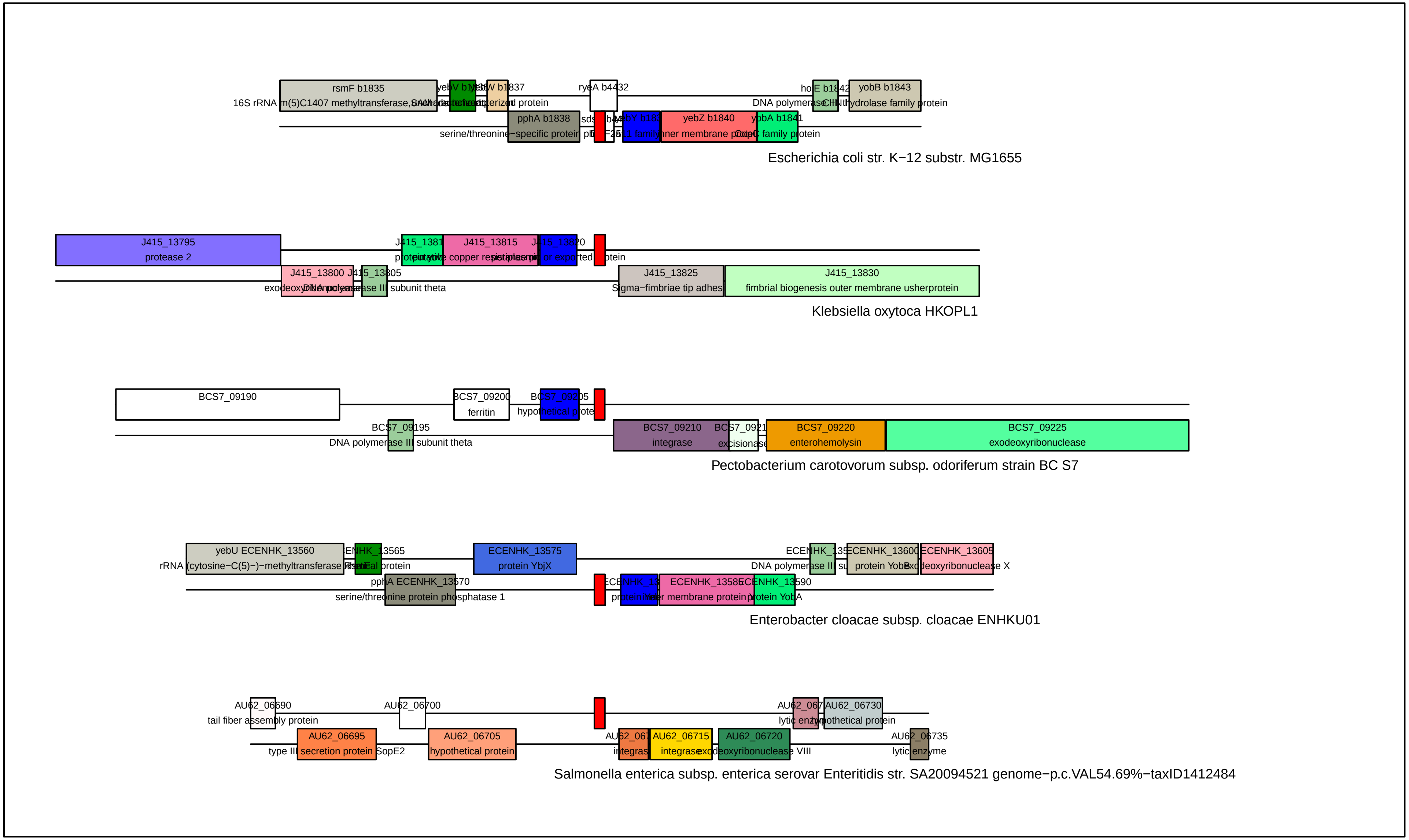

rsmF b1835
16S rRNA m(5)C1407 methyltransferase, SAM-dependent
ryeA b4432
yobB b1843
pphA b1838
serine/threonine-specific protein
yebZ b1840
yobA b1841
Escherichia coli str. K−12 substr. MG1655
J415_13795
protease 2
J415_13815
J415_13800
J415_13825
J415_13830
fimbrial biogenesis outer membrane usherprotein
Klebsiella oxytoca HKOPL1
BCS7_09190
BCS7_09200
ferritin
BCS7_09195
DNA polymerase III subunit theta
BCS7_09210
integrase
BCS7_09220
enterohemolysin
BCS7_09225
exodeoxyribonuclease
Pectobacterium carotovorum subsp. odoriferum strain BC S7
yebU ECENHK_13560
rRNA (cytosine−C(5)−)−methyltransferase RsmF
ECENHK_13575
protein YbjX
pphA ECENHK_13570
serine/threonine protein phosphatase 1
ECENHK_13605
Enterobacter cloacae subsp. cloacae ENHKU01
AU62_06690
tail fiber assembly protein
AU62_06700
AU62_06695
type III secretion protein SopE2
AU62_06705
hypothetical protein
AU62_06715
integrase
AU62_06720
exodeoxyribonuclease VIII
AU62_06730
hypothetical protein
AU62_06735
lytic enzyme
Salmonella enterica subsp. enterica serovar Enteritidis str. SA20094521 genome−p.c.VAL54.69%−taxID1412484

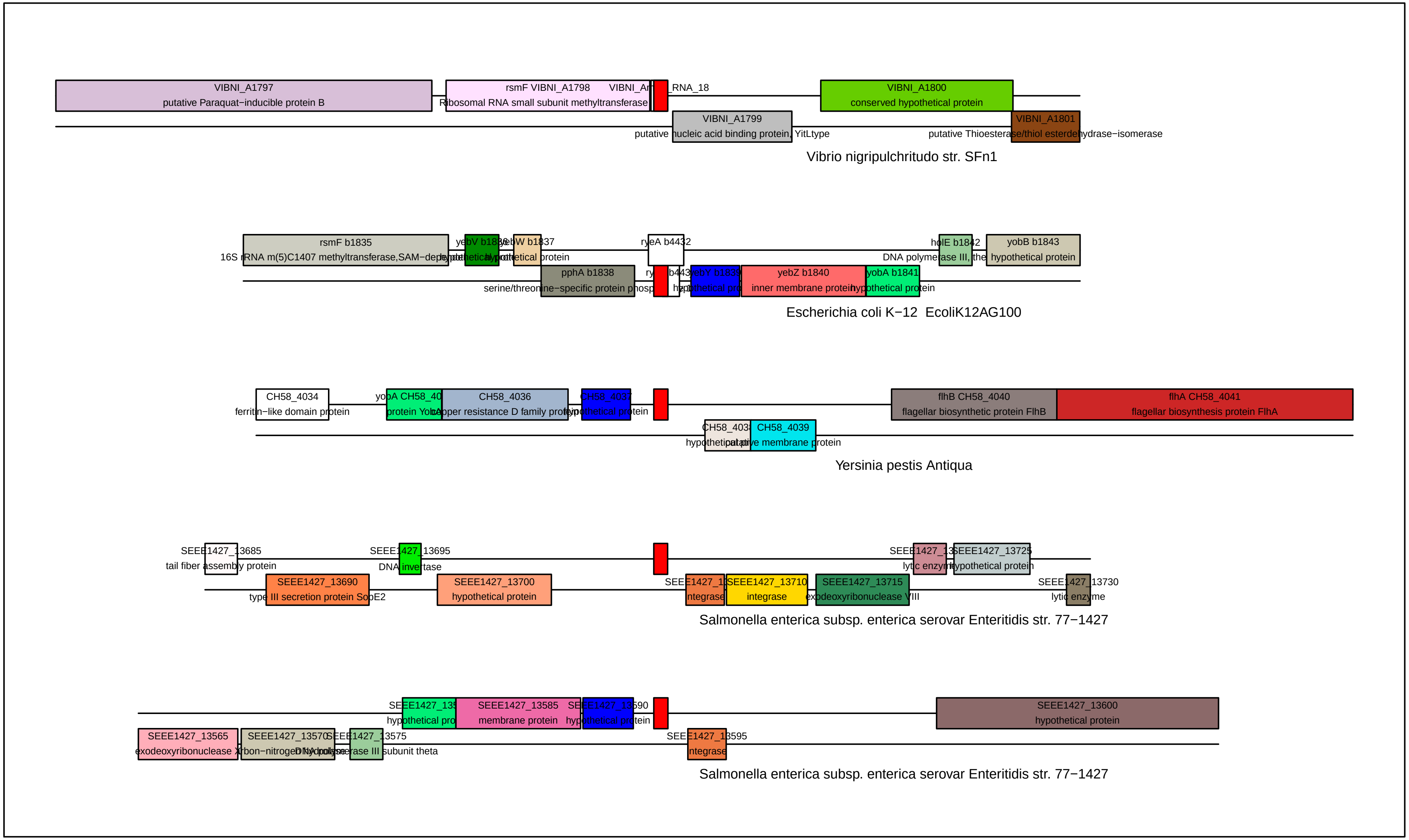

VIBNI_A1797
putative Paraquat−inducible protein B
rsmF VIBNI_A1798
Ribosomal RNA small subunit methyltransferase
VIBNI_A1800
conserved hypothetical protein
VIBNI_A1799
VIBNI_A1801
Vibrio nigripulchritudo str. SFn1
rsmF b1835
pphA b1838
yebZ b1840
inner membrane protein
yobA b1841
yobB b1843
hypothetical protein
Escherichia coli K−12 EcoliK12AG100
CH58_4034
CH58_4036
flhB CH58_4040
flagellar biosynthetic protein FlhB
flhA CH58_4041
flagellar biosynthesis protein FlhA
CH58_4039
Yersinia pestis Antiqua
SEEE1427_13690
SEEE1427_13700
hypothetical protein
SEEE1427_13710
integrase
SEEE1427_13715
Salmonella enterica subsp. enterica serovar Enteritidis str. 77−1427
SEEE1427_13565
exodeoxyribonuclease
SEEE1427_13585
membrane protein
SEEE1427_13600
hypothetical protein
Salmonella enterica subsp. enterica serovar Enteritidis str. 77−1427

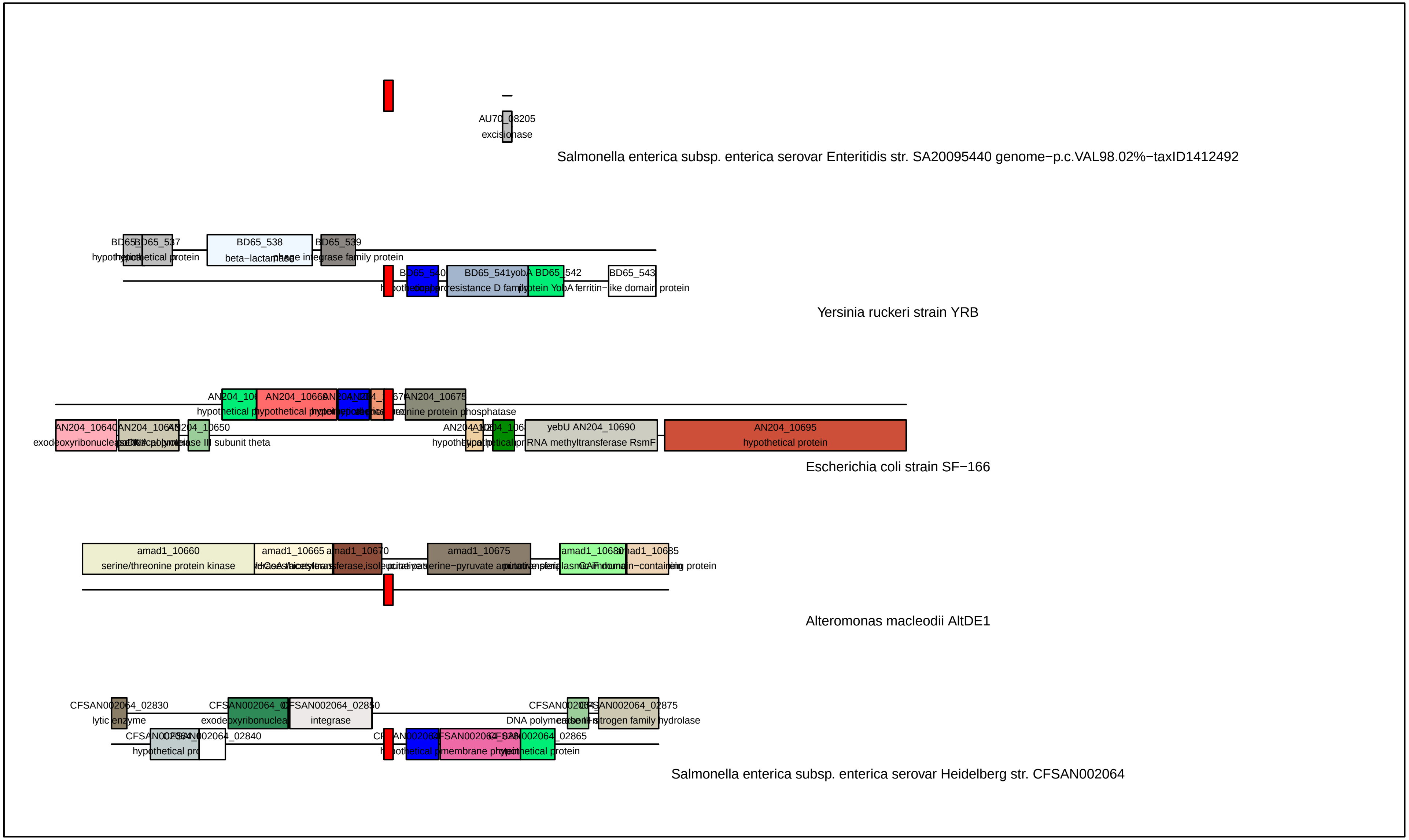

AU70_08205
excisionase
Salmonella enterica subsp. enterica serovar Enteritidis str. SA20095440 genome−p.c.VAL98.02%−taxID1412492
BD65_537
BD65_538
beta−lactamase
BD65_539
phage integrase family protein
BD65_541yobA
BD65_542
BD65_543
protein YobA
ferritin−like domain protein
Yersinia ruckeri strain YRB
AN204_10675
hypothetical protein
phosphatase
AN204_10650
DNA polymerase III subunit theta
yebU AN204_10690
rRNA methyltransferase RsmF
AN204_10695
hypothetical protein
Escherichia coli strain SF−166
amad1_10660
serine/threonine protein kinase
amad1_10665
amad1_10670
amad1_10675
amad1_10685
Alteromonas macleodii AltDE1
CFSAN002064_02830
lytic enzyme
CFSAN002064_02850
integrase
CFSAN002064_02875
DNA polymerase III
estrogen family hydrolase
CFSAN002064_02840
hypothetical protein
CFSAN002064_02865
membrane protein
Salmonella enterica subsp. enterica serovar Heidelberg str. CFSAN002064

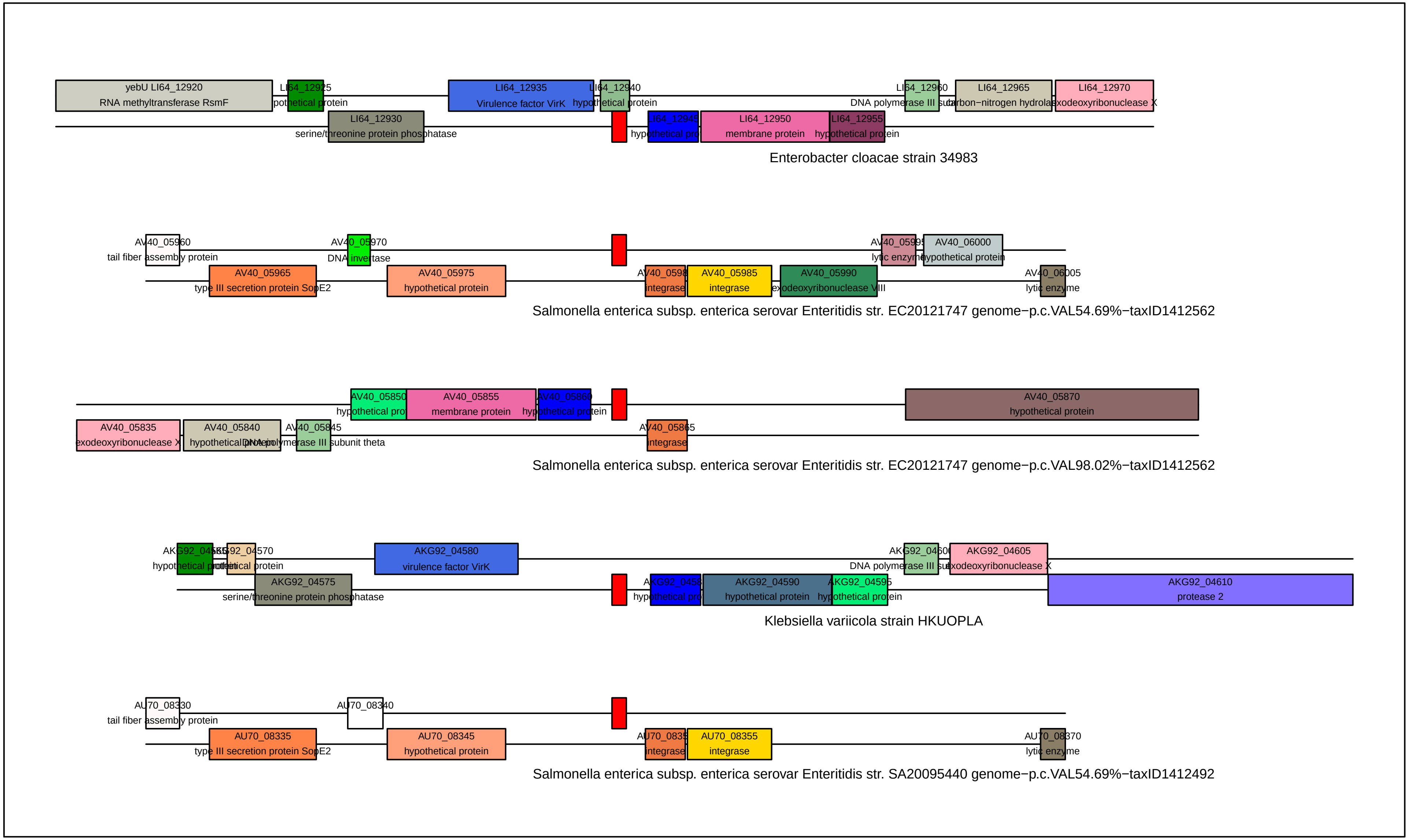
yebU LI64_12920
RNA methyltransferase RsmF
LI64_12930
serine/threonine protein phosphatase
LI64_12935
Virulence factor VirK
LI64_12950
membrane protein
LI64_12955
LI64_12960
LI64_12965
LI64_12970
exodeoxyribonuclease X
Enterobacter cloacae strain 34983
AV40_05960
tail fiber assembly protein
AV40_05965
type III secretion protein SopE2
AV40_05970
AV40_05975
hypothetical protein
AV40_05985
integrase
AV40_05990
exodeoxyribonuclease VIII
AV40_06000
hypothetical protein
AV40_06005
lytic enzyme
Salmonella enterica subsp. enterica serovar Enteritidis str. EC20121747 genome−p.c.VAL54.69%−taxID1412562
AV40_05835
exodeoxyribonuclease X
AV40_05840
AV40_05845
AV40_05850
AV40_05855
membrane protein
AV40_05865
integrase
AV40_05870
hypothetical protein
Salmonella enterica subsp. enterica serovar Enteritidis str. EC20121747 genome−p.c.VAL98.02%−taxID1412562
AKG92_04570
AKG92_04575
serine/threonine protein phosphatase
AKG92_04580
virulence factor VirK
AKG92_04590
hypothetical protein
AKG92_04595
AKG92_04605
exodeoxyribonuclease X
AKG92_04610
protease 2
Klebsiella variicola strain HKUOPLA
AU70_08330
tail fiber assembly protein
AU70_08335
type III secretion protein SopE2
AU70_08340
AU70_08345
hypothetical protein
AU70_08355
integrase
AU70_08370
lytic enzyme
Salmonella enterica subsp. enterica serovar Enteritidis str. SA20095440 genome−p.c.VAL54.69%−taxID1412492

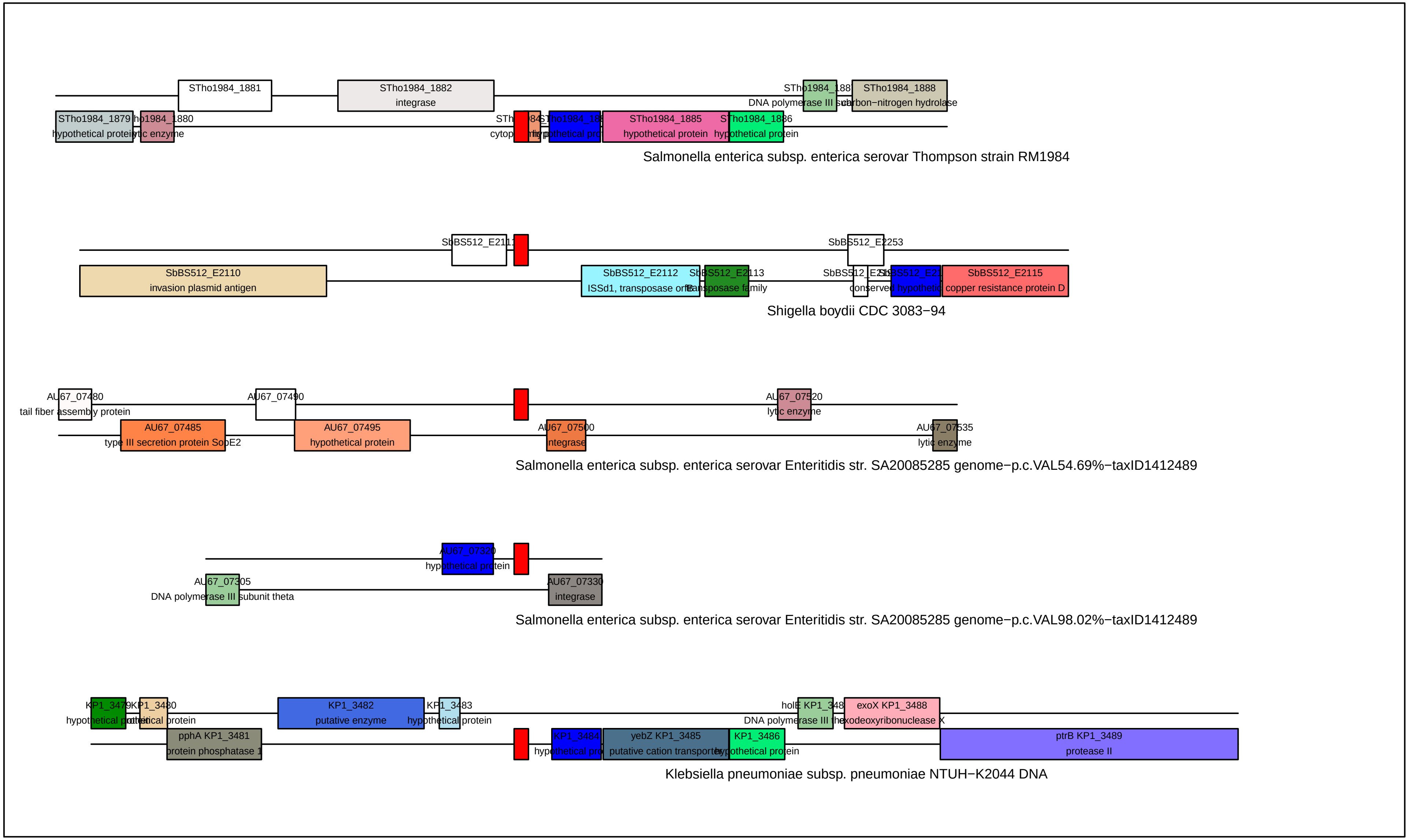

STho1984_1881
STho1984_1882
integrase
STho1984_1888
carbon−nitrogen hydrolase
STho1984_1879
hypothetical protein
STho1984_1885
hypothetical protein
Salmonella enterica subsp. enterica serovar Thompson strain RM1984
SbBS512_E2253
SbBS512_E2110
invasion plasmid antigen
SbBS512_E2112
ISSd1, transposase orfB
SbBS512_E2115
copper resistance protein D
Shigella boydii CDC 3083−94
AU67_07480
tail fiber assembly protein
AU67_07490
AU67_07520
lytic enzyme
AU67_07485
type III secretion protein SopE2
AU67_07495
hypothetical protein
AU67_07500
integrase
AU67_07535
lytic enzyme
Salmonella enterica subsp. enterica serovar Enteritidis str. SA20085285 genome−p.c.VAL54.69%−taxID1412489
AU67_07320
hypothetical protein
AU67_07305
DNA polymerase III subunit theta
AU67_07330
integrase
Salmonella enterica subsp. enterica serovar Enteritidis str. SA20085285 genome−p.c.VAL98.02%−taxID1412489
KP1_3482
putative enzyme
KP1_3483
hypothetical protein
exoX KP1_3488
exodeoxyribonuclease X
pphA KP1_3481
protein phosphatase 1
yebZ KP1_3485
putative cation transporter
KP1_3486
hypothetical protein
ptrB KP1_3489
protease II
Klebsiella pneumoniae subsp. pneumoniae NTUH−K2044 DNA

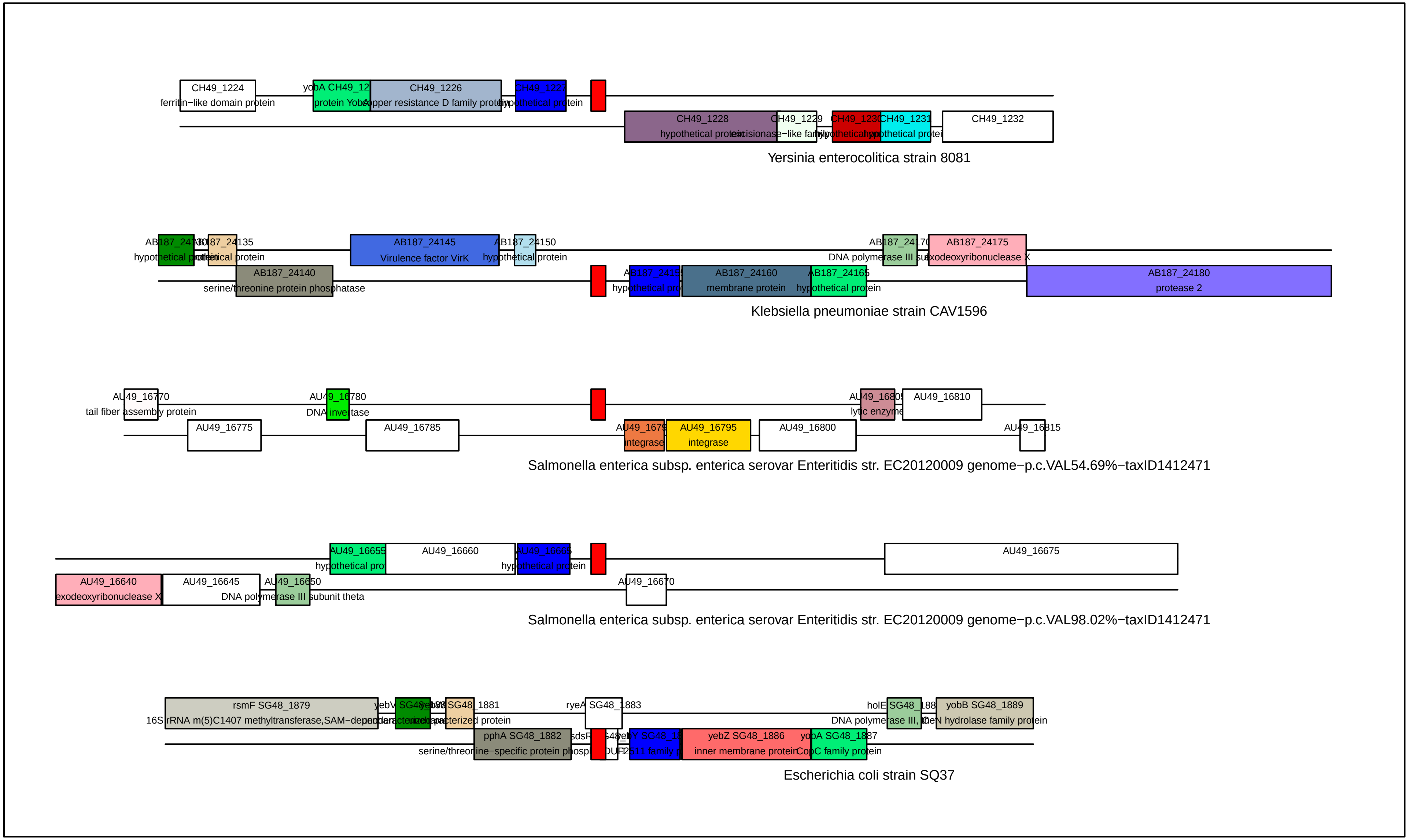
CH49_1224
ferritin−like domain protein
CH49_1226
CH49_1228
CH49_1229
CH49_1231
CH49_1232
Yersinia enterocolitica strain 8081
AB187_24135
AB187_24145
Virulence factor VirK
AB187_24150
hypothetical protein
AB187_24140
serine/threonine protein phosphatase
AB187_24160
membrane protein
hypothetical protein
AB187_24175
AB187_24180
protease 2
Klebsiella pneumoniae strain CAV1596
AU49_16770
tail fiber assembly protein
AU49_16780
DNA invertase
AU49_16775
AU49_16785
AU49_16795
integrase
AU49_16800
AU49_16810
AU49_16815
lytic enzyme
Salmonella enterica subsp. enterica serovar Enteritidis str. EC20120009 genome−p.c.VAL54.69%−taxID1412471
AU49_16660
hypothetical protein
AU49_16675
AU49_16640
exodeoxyribonuclease X
AU49_16645
AU49_16650
DNA polymerase III subunit theta
AU49_16670
Salmonella enterica subsp. enterica serovar Enteritidis str. EC20120009 genome−p.c.VAL98.02%−taxID1412471
rsmF SG48_1879
pphA SG48_1882
serine/threonine−specific protein phosph
yebZ SG48_1886
inner membrane protein
yobB SG48_1889
Escherichia coli strain SQ37

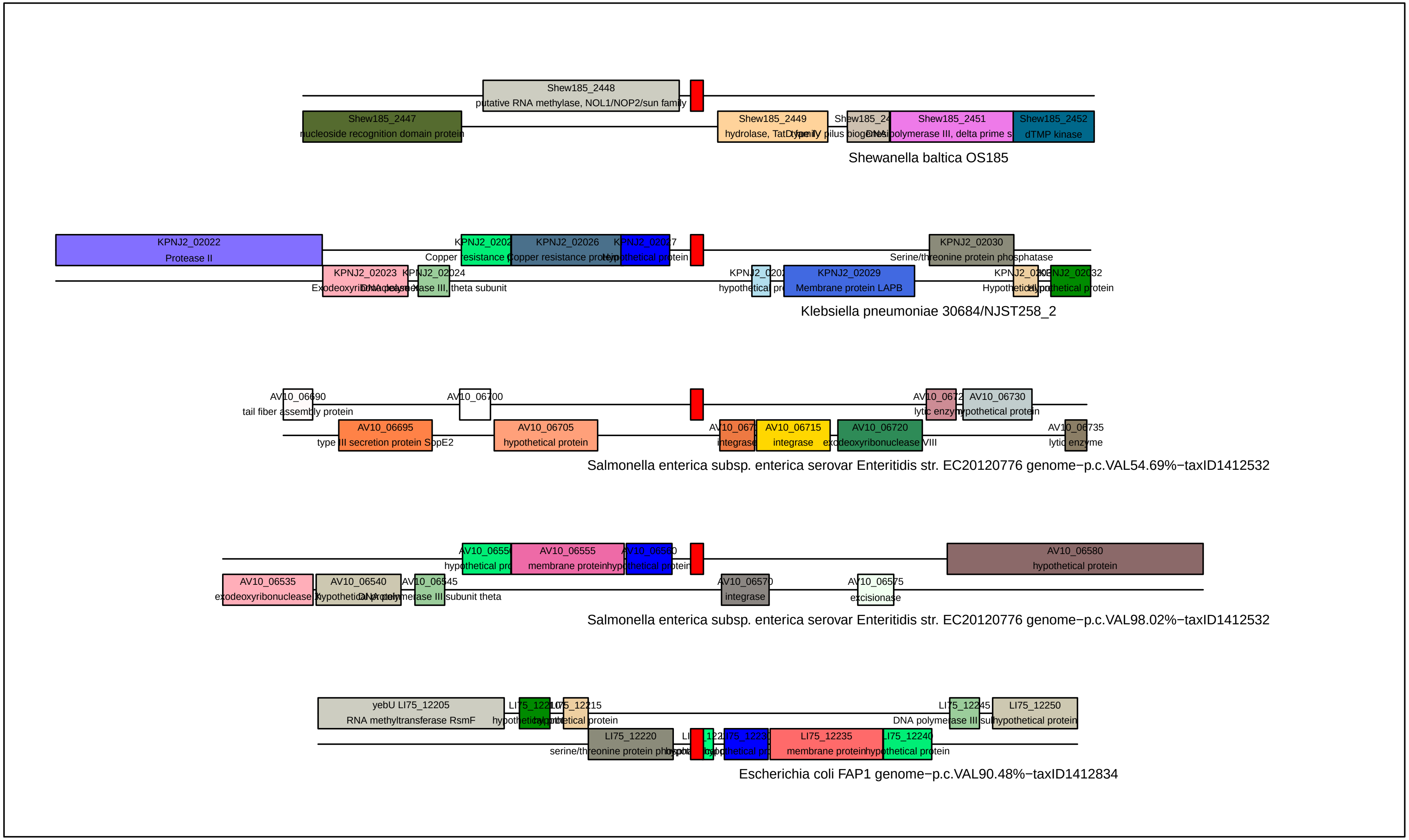
Shew185_2448
putative RNA methylase, NOL1/NOP2/sun family
Shew185_2447
nucleoside recognition domain protein
Shew185_2449
Shew185_2451
Shew185_2452
dTMP kinase
Shewanella baltica OS185
KPNJ2_02022
Protease II
KPNJ2_02026
KPNJ2_02023
KPNJ2_02029
Membrane protein LAPB
KPNJ2_02030
Serine/threonine protein phosphatase
Klebsiella pneumoniae 30684/NJST258_2
AV10_06690
tail fiber assembly protein
AV10_06700
AV10_06695
type III secretion protein SopE2
AV10_06705
hypothetical protein
AV10_06715
integrase
AV10_06720
exodeoxyribonuclease VIII
AV10_06730
AV10_06735
lytic enzyme
Salmonella enterica subsp. enterica serovar Enteritidis str. EC20120776 genome−p.c.VAL54.69%−taxID1412532
AV10_06555
membrane protein
AV10_06580
hypothetical protein
AV10_06535
AV10_06540
AV10_06545
AV10_06570
integrase
AV10_06575
excisionase
Salmonella enterica subsp. enterica serovar Enteritidis str. EC20120776 genome−p.c.VAL98.02%−taxID1412532
yebU LI75_12205
RNA methyltransferase RsmF
LI75_12215
LI75_12245
LI75_12250
hypothetical protein
LI75_12220
LI75_12235
membrane protein
LI75_12240
Escherichia coli FAP1 genome−p.c.VAL90.48%−taxID1412834

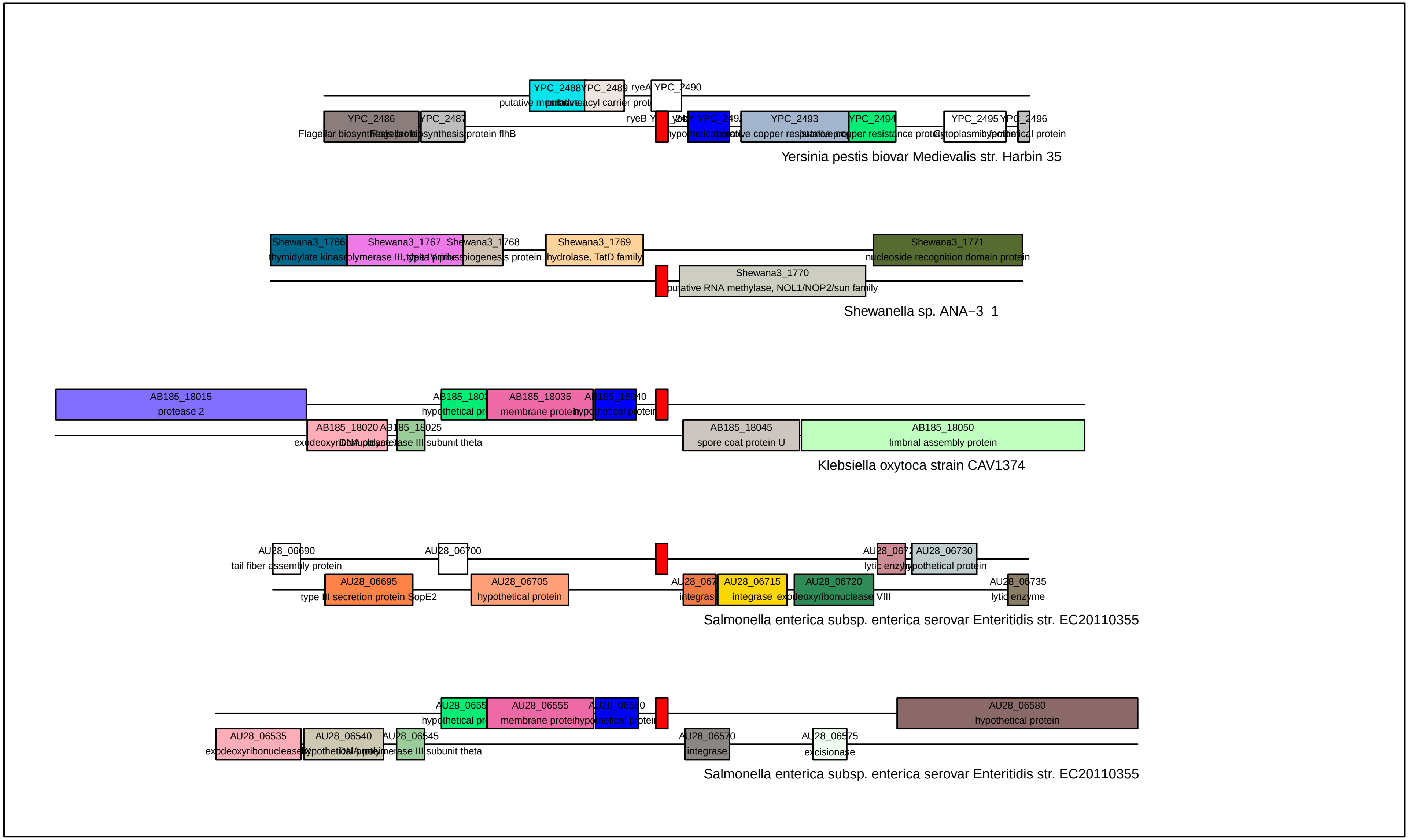

YPC_2488
YPC_2490
YPC_2486
YPC_2487
YPC_2493
YPC_2494
YPC_2495
Yersinia pestis biovar Medievalis str. Harbin 35
Shewana3_1767
Shewana3_1769
hydrolase, TatD family
Shewana3_1771
Shewana3_1770
putative RNA methylase, NOL1/NOP2/sun family
Shewanella sp. ANA−3 1
AB185_18015
protease 2
AB185_18035
AB185_18020
AB185_18045
spore coat protein U
AB185_18050
fimbrial assembly protein
Klebsiella oxytoca strain CAV1374
AU28_06730
AU28_06695
type III secretion protein SopE2
AU28_06705
hypothetical protein
AU28_06715
integrase
AU28_06720
AU28_06735
lytic enzyme
Salmonella enterica subsp. enterica serovar Enteritidis str. EC20110355
AU28_06555
AU28_06580
hypothetical protein
AU28_06535
AU28_06540
AU28_06545
AU28_06570
integrase
AU28_06575
excisionase
Salmonella enterica subsp. enterica serovar Enteritidis str. EC20110355

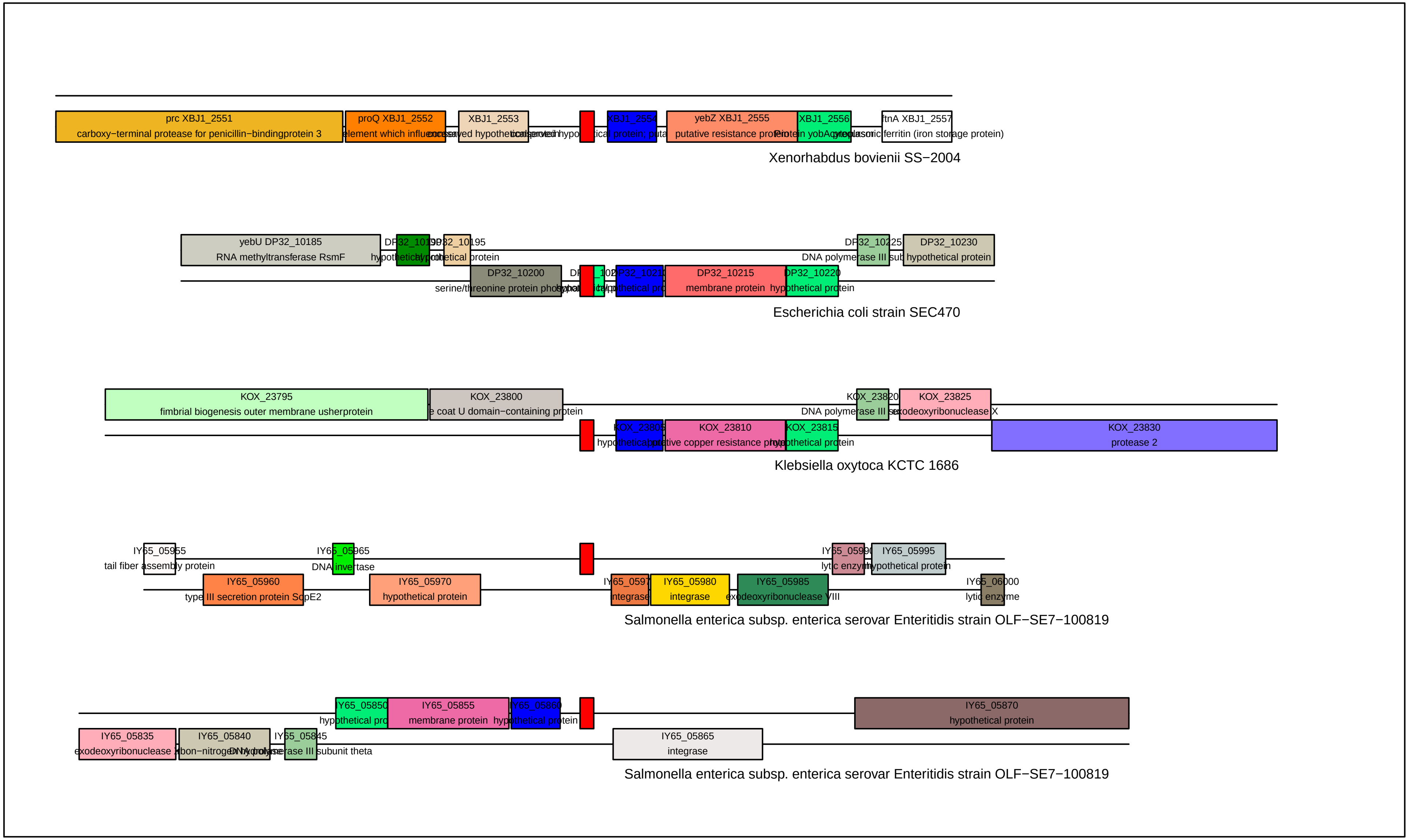
prc XBJ1_2551
carboxy−terminal protease for penicillin−bindingprotein 3
proQ XBJ1_2552
XBJ1_2553
XBJ1_2554
yebZ XBJ1_2555
XBJ1_2556
ftnA XBJ1_2557
ferritin (iron storage protein)
Xenorhabdus bovienii SS−2004
yebU DP32_10185
RNA methyltransferase RsmF
DP32_10200
DP32_10215
membrane protein
DP32_10230
hypothetical protein
Escherichia coli strain SEC470
KOX_23795
fimbrial biogenesis outer membrane usherprotein
KOX_23800
KOX_23810
KOX_23825
KOX_23830
protease 2
Klebsiella oxytoca KCTC 1686
IY65_05960
type III secretion protein SopE2
IY65_05970
hypothetical protein
IY65_05980
integrase
IY65_05985
IY65_05995
Salmonella enterica subsp. enterica serovar Enteritidis strain OLF−SE7−100819
IY65_05835
exodeoxyribonuclease
IY65_05840
IY65_05855
membrane protein
IY65_05865
integrase
IY65_05870
hypothetical protein
Salmonella enterica subsp. enterica serovar Enteritidis strain OLF−SE7−100819

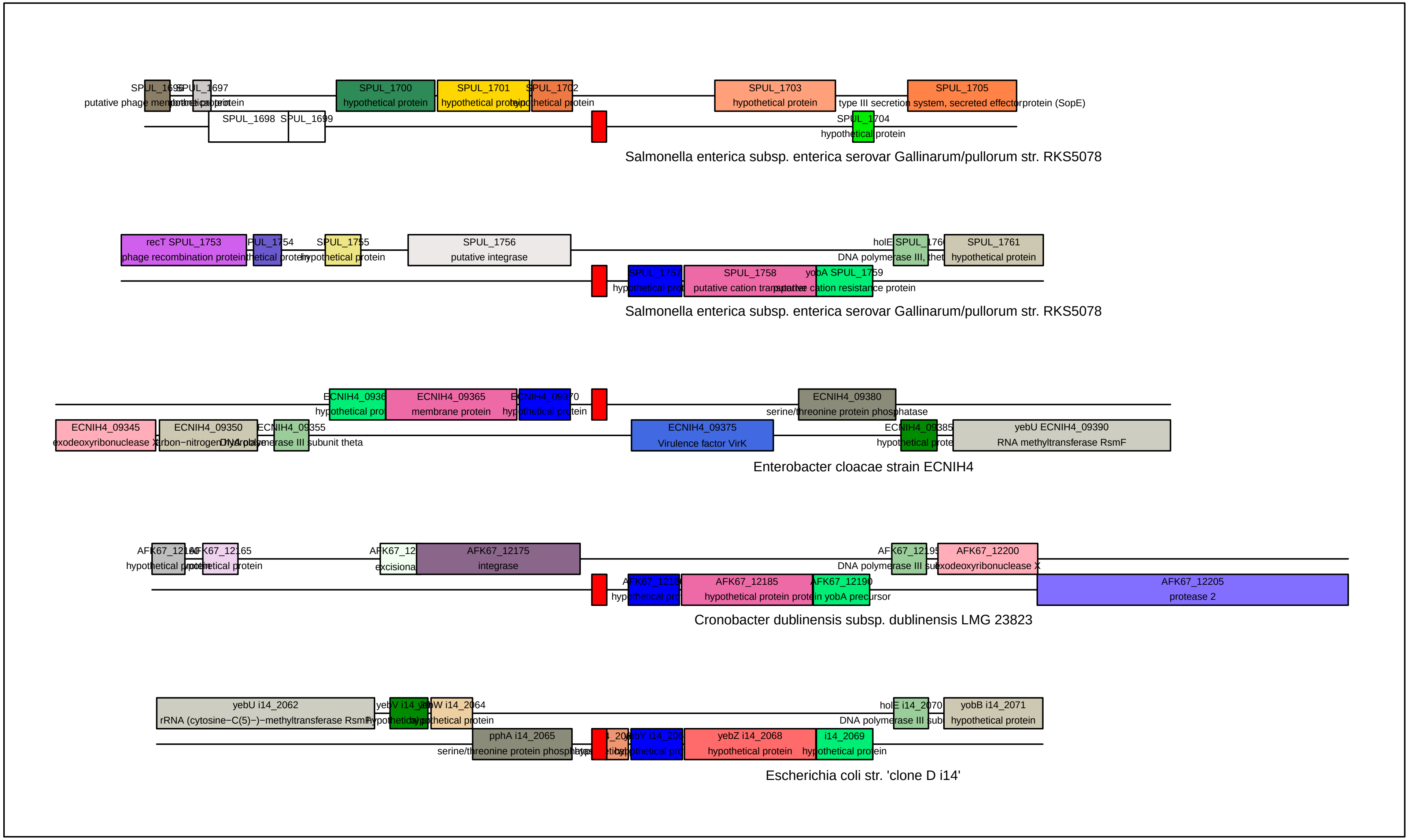
SPUL_1700
hypothetical protein
SPUL_1701
SPUL_1702
SPUL_1703
hypothetical protein
SPUL_1705
type III secretion system, secreted effector protein (SopE)
SPUL_1698
SPUL_1699
SPUL_1704
hypothetical protein
Salmonella enterica subsp. enterica serovar Gallinarum/pullorum str. RKS5078
recT SPUL_1753
phage recombination protein
SPUL_1756
putative integrase
SPUL_1761
hypothetical protein
SPUL_1758
yobA SPUL_1759
cation resistance protein
Salmonella enterica subsp. enterica serovar Gallinarum/pullorum str. RKS5078
ECNIH4_09365
membrane protein
ECNIH4_09380
serine/threonine protein phosphatase
ECNIH4_09345
ECNIH4_09350
ECNIH4_09375
Virulence factor VirK
yebU ECNIH4_09390
RNA methyltransferase RsmF
Enterobacter cloacae strain ECNIH4
AFK67_12165
AFK67_12175
integrase
AFK67_12200
AFK67_12185
hypothetical protein
AFK67_12190
AFK67_12205
protease 2
Cronobacter dublinensis subsp. dublinensis LMG 23823
yebU i14_2062
rRNA (cytosine−C(5)−)−methyltransferase RsmF
yobB i14_2071
hypothetical protein
pphA i14_2065
yebZ i14_2068
hypothetical protein
i14_2069
Escherichia coli str. 'clone D i14'

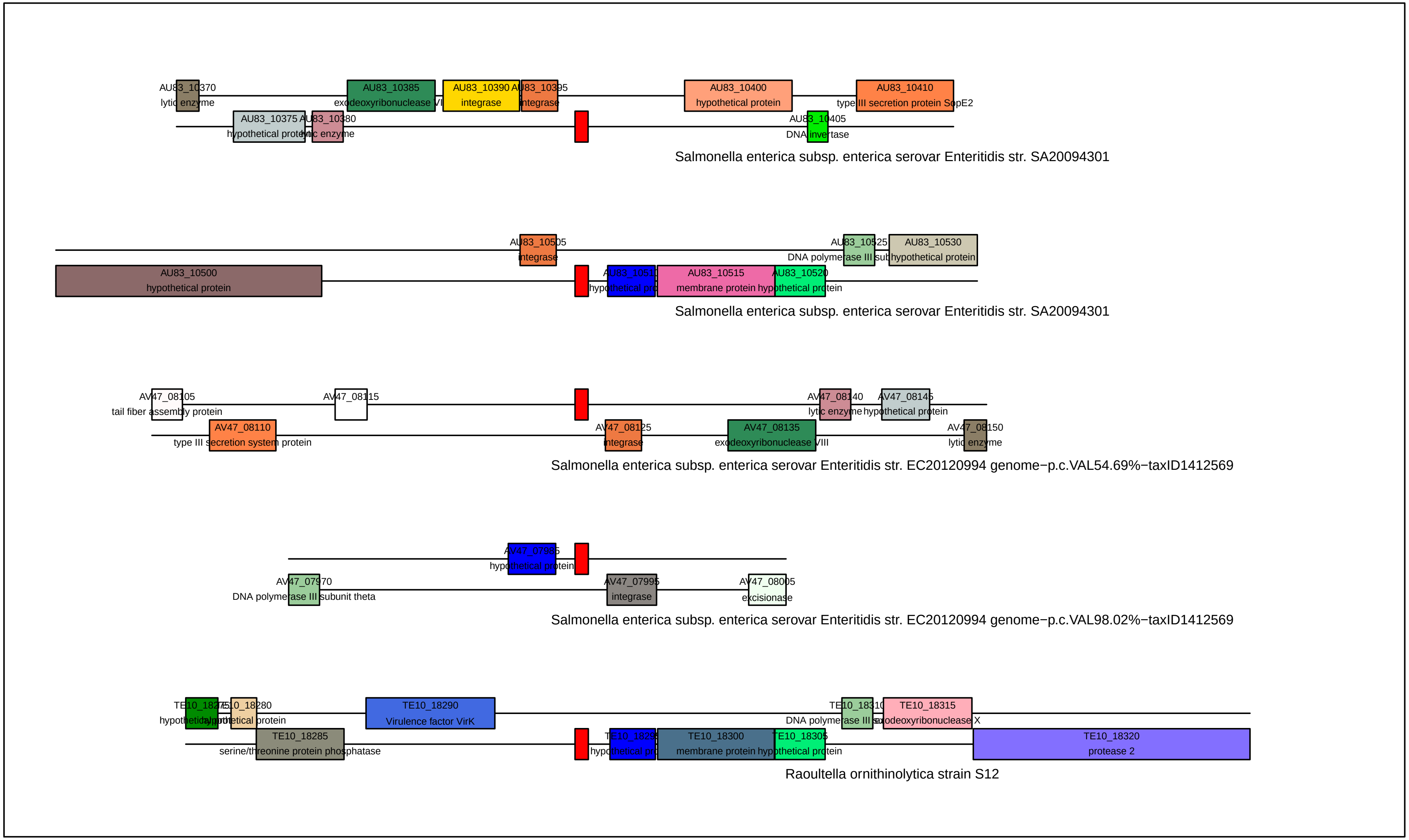

AU83_10370
lytic enzyme
AU83_10385
exodeoxyribonuclease VII
AU83_10390
integrase
AU83_10395
integrase
AU83_10400
hypothetical protein
AU83_10410
type III secretion protein SopE2
AU83_10375
hypothetical protein
AU83_10380
lytic enzyme
AU83_10405
DNA invertase
Salmonella enterica subsp. enterica serovar Enteritidis str. SA20094301
AU83_10505
integrase
AU83_10525
DNA polymerase III sub
AU83_10530
hypothetical protein
AU83_10500
hypothetical protein
AU83_10515
membrane protein
AU83_10520
hypothetical protein
Salmonella enterica subsp. enterica serovar Enteritidis str. SA20094301
AV47_08105
tail fiber assembly protein
AV47_08115
AV47_08140
lytic enzyme
AV47_08145
hypothetical protein
AV47_08110
type III secretion system protein
AV47_08125
integrase
AV47_08135
exodeoxyribonuclease VIII
AV47_08150
lytic enzyme
Salmonella enterica subsp. enterica serovar Enteritidis str. EC20120994 genome−p.c.VAL54.69%−taxID1412569
AV47_07985
hypothetical protein
AV47_07970
DNA polymerase III subunit theta
AV47_07995
integrase
AV47_08005
excisionase
Salmonella enterica subsp. enterica serovar Enteritidis str. EC20120994 genome−p.c.VAL98.02%−taxID1412569
TE10_18280
hypothetical protein
TE10_18290
Virulence factor VirK
TE10_18310
DNA polymerase III
TE10_18315
exodeoxyribonuclease X
TE10_18285
serine/threonine protein phosphatase
TE10_18300
membrane protein
TE10_18305
hypothetical protein
TE10_18320
protease 2
Raoultella ornithinolytica strain S12

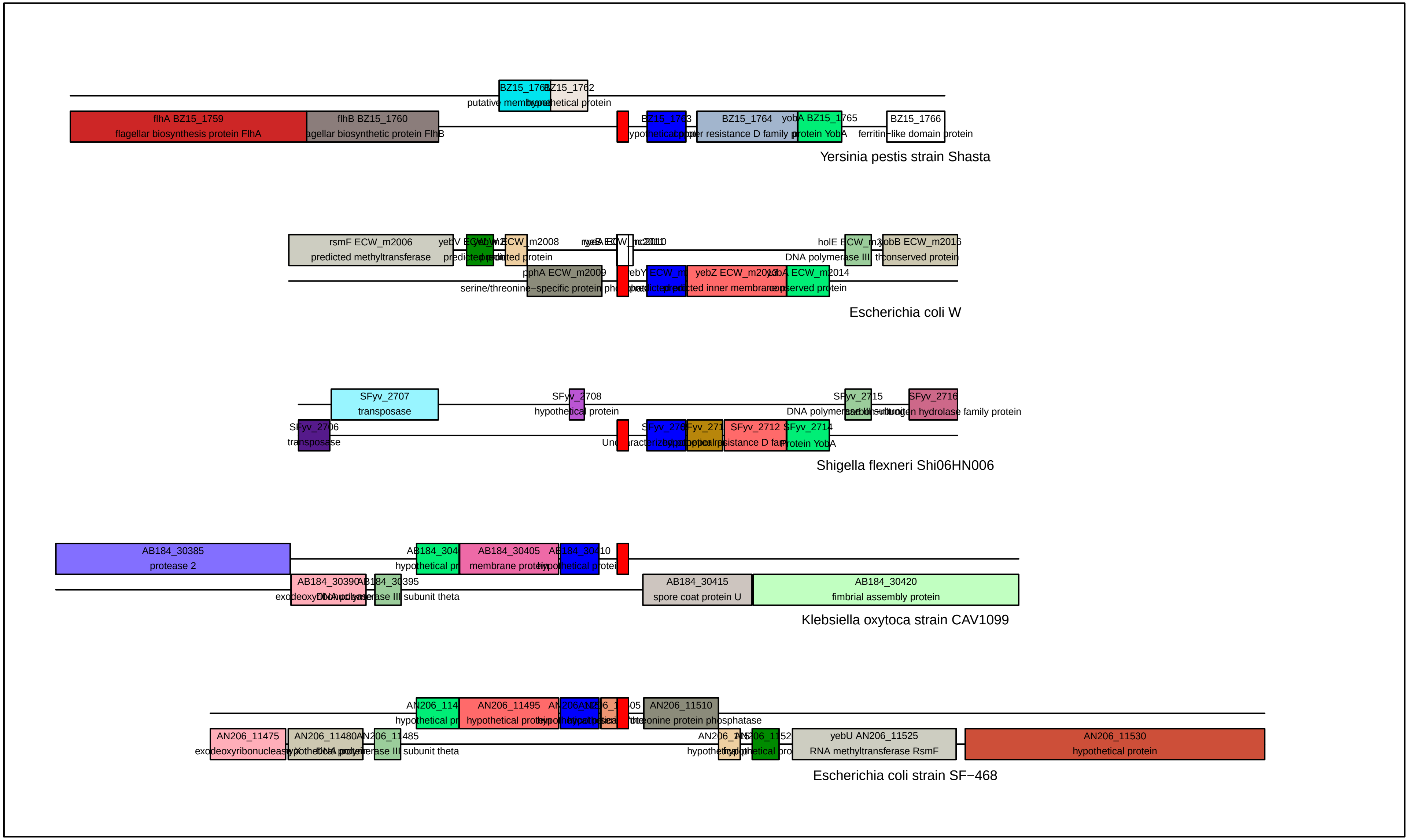

flhA BZ15_1759
flagellar biosynthesis protein FlhA
flhB BZ15_1760
BZ15_1762
BZ15_1764
BZ15_1766
ferritin-like domain protein
Yersinia pestis strain Shasta
rsmF ECW_m2006
predicted methyltransferase
pphA ECW_m2009
yebZ ECW_m2013
conserved protein
Escherichia coli W
SFyv_2707
transposase
hypothetical protein
SFyv_2712
Shigella flexneri Shi06HN006
AB184_30385
protease 2
AB184_30405
AB184_30395
AB184_30415
spore coat protein U
AB184_30420
fimbrial assembly protein
Klebsiella oxytoca strain CAV1099
AN206_11495
AN206_11510
AN206_11475
yebU AN206_11525
RNA methyltransferase RsmF
AN206_11530
hypothetical protein
Escherichia coli strain SF−468

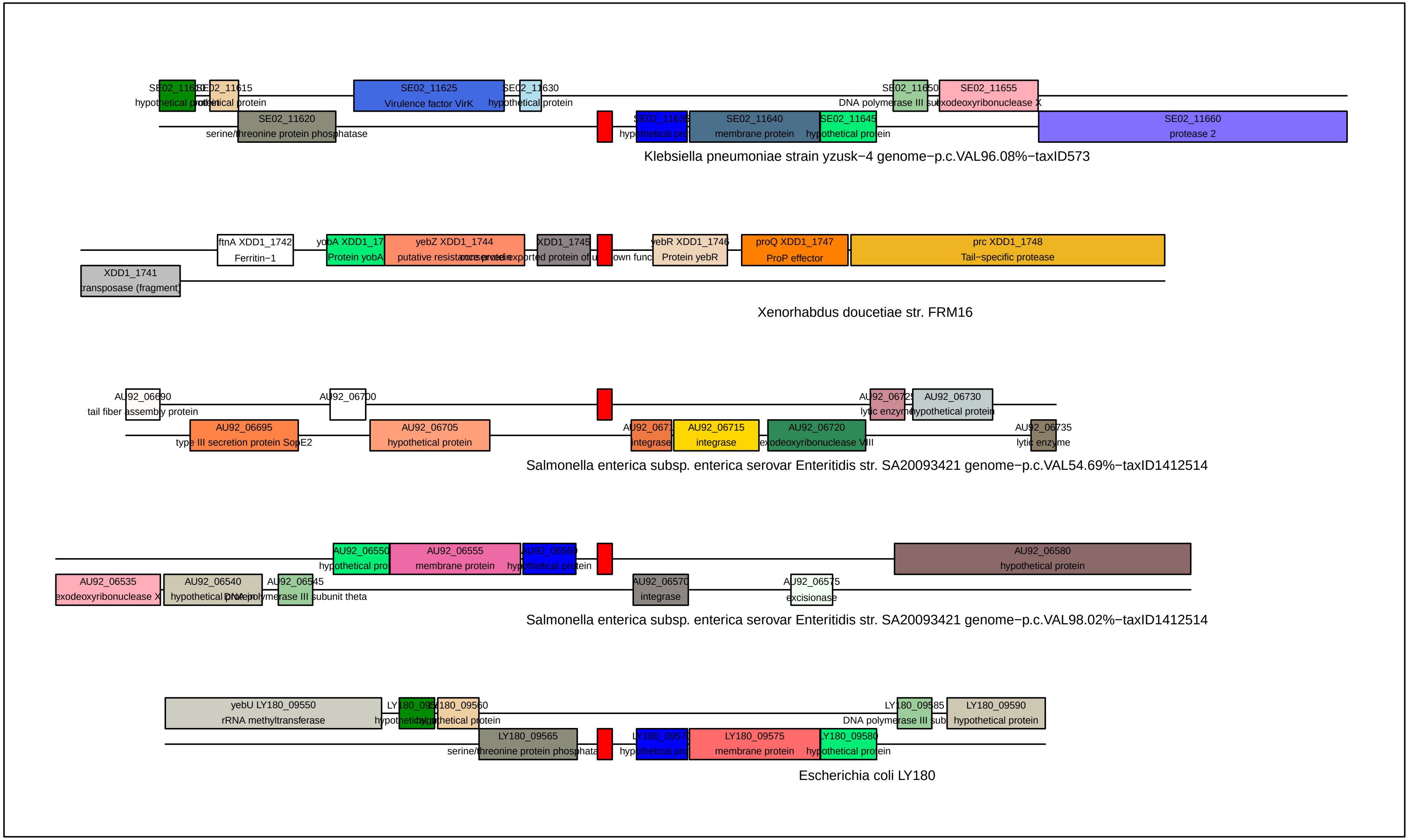
Klebsiella pneumoniae strain yzusk−4 genome−p.c.VAL96.08%−taxID573
Xenorhabdus doucetiae str. FRM16
Salmonella enterica subsp. enterica serovar Enteritidis str. SA20093421 genome−p.c.VAL54.69%−taxID1412514
Salmonella enterica subsp. enterica serovar Enteritidis str. SA20093421 genome−p.c.VAL98.02%−taxID1412514
Escherichia coli LY180

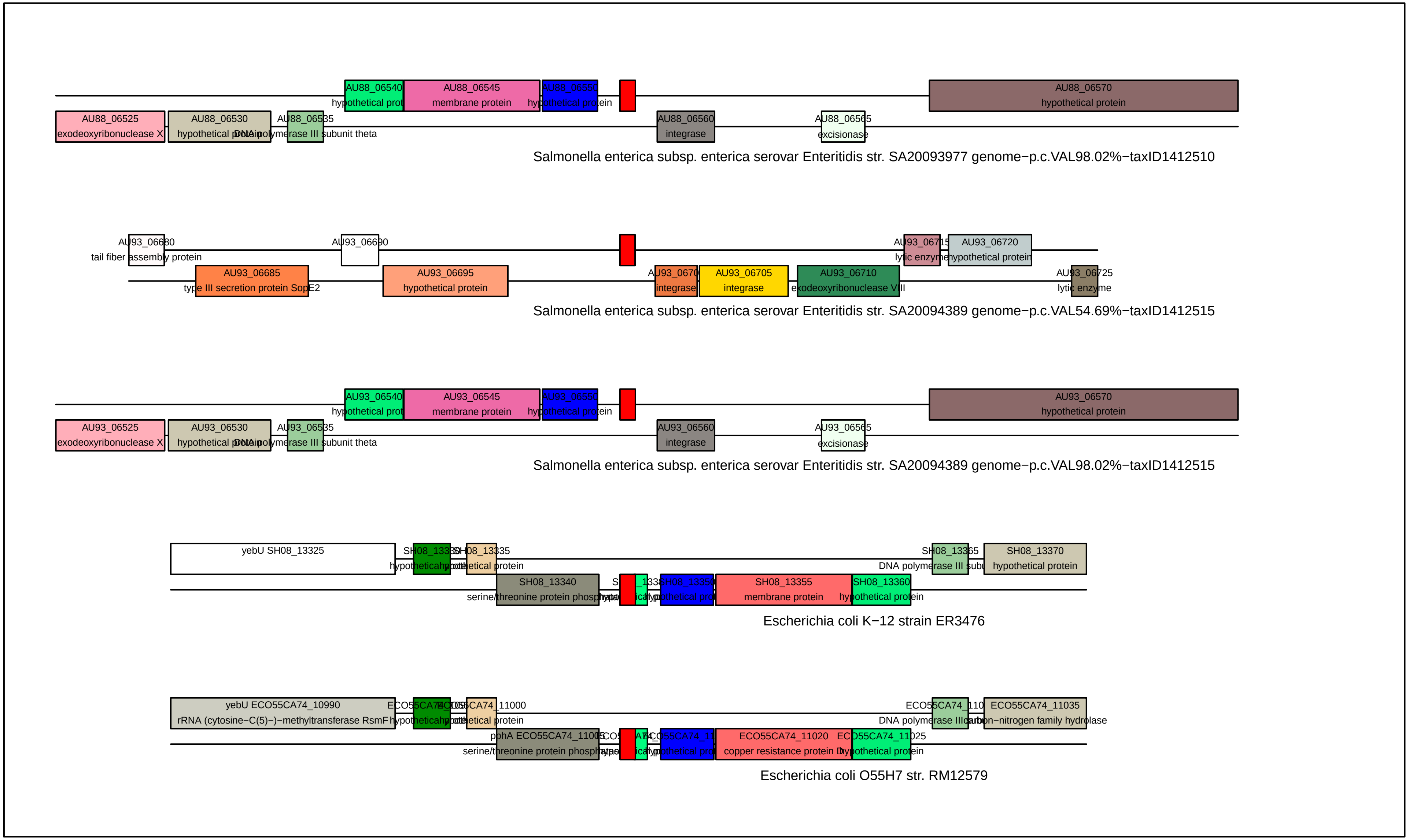
AU88_06540
hypothetical prot
AU88_06545
membrane protein
AU88_06550
hypothetical protein
AU88_06570
hypothetical protein
AU88_06525
exodeoxyribonuclease X
AU88_06530
hypothetical protein
AU88_06535
DNA polymerase III subunit theta
AU88_06560
integrase
AU88_06565
excisionase
Salmonella enterica subsp. enterica serovar Enteritidis str. SA20093977 genome−p.c.VAL98.02%−taxID1412510
AU93_06680
tail fiber assembly protein
AU93_06690
AU93_06715
lytic enzyme
AU93_06720
hypothetical protein
AU93_06685
type III secretion protein SopE2
AU93_06695
hypothetical protein
integrase
AU93_06705
integrase
AU93_06710
exodeoxyribonuclease VIII
AU93_06725
lytic enzyme
Salmonella enterica subsp. enterica serovar Enteritidis str. SA20094389 genome−p.c.VAL54.69%−taxID1412515
AU93_06540
hypothetical prot
AU93_06545
membrane protein
AU93_06550
hypothetical protein
AU93_06570
hypothetical protein
AU93_06525
exodeoxyribonuclease X
AU93_06530
hypothetical protein
AU93_06535
DNA polymerase III subunit theta
AU93_06560
integrase
AU93_06565
excisionase
Salmonella enterica subsp. enterica serovar Enteritidis str. SA20094389 genome−p.c.VAL98.02%−taxID1412515
yebU SH08_13325
SH08_13335
hypothetical protein
SH08_13365
DNA polymerase III
SH08_13370
hypothetical protein
SH08_13340
serine/threonine protein phosphatase
SH08_13355
membrane protein
SH08_13360
hypothetical protein
Escherichia coli K−12 strain ER3476
yebU ECO55CA74_10990
rRNA (cytosine−C(5)−)−methyltransferase RsmF
ECO55CA74_11000
hypothetical protein
DNA polymerase III
ECO55CA74_11035
carbon−nitrogen family hydrolase
pphA ECO55CA74_11005
serine/threonine protein phosphatase
ECO55CA74_11020
copper resistance protein D
ECO55CA74_11025
hypothetical protein
Escherichia coli O55H7 str. RM12579

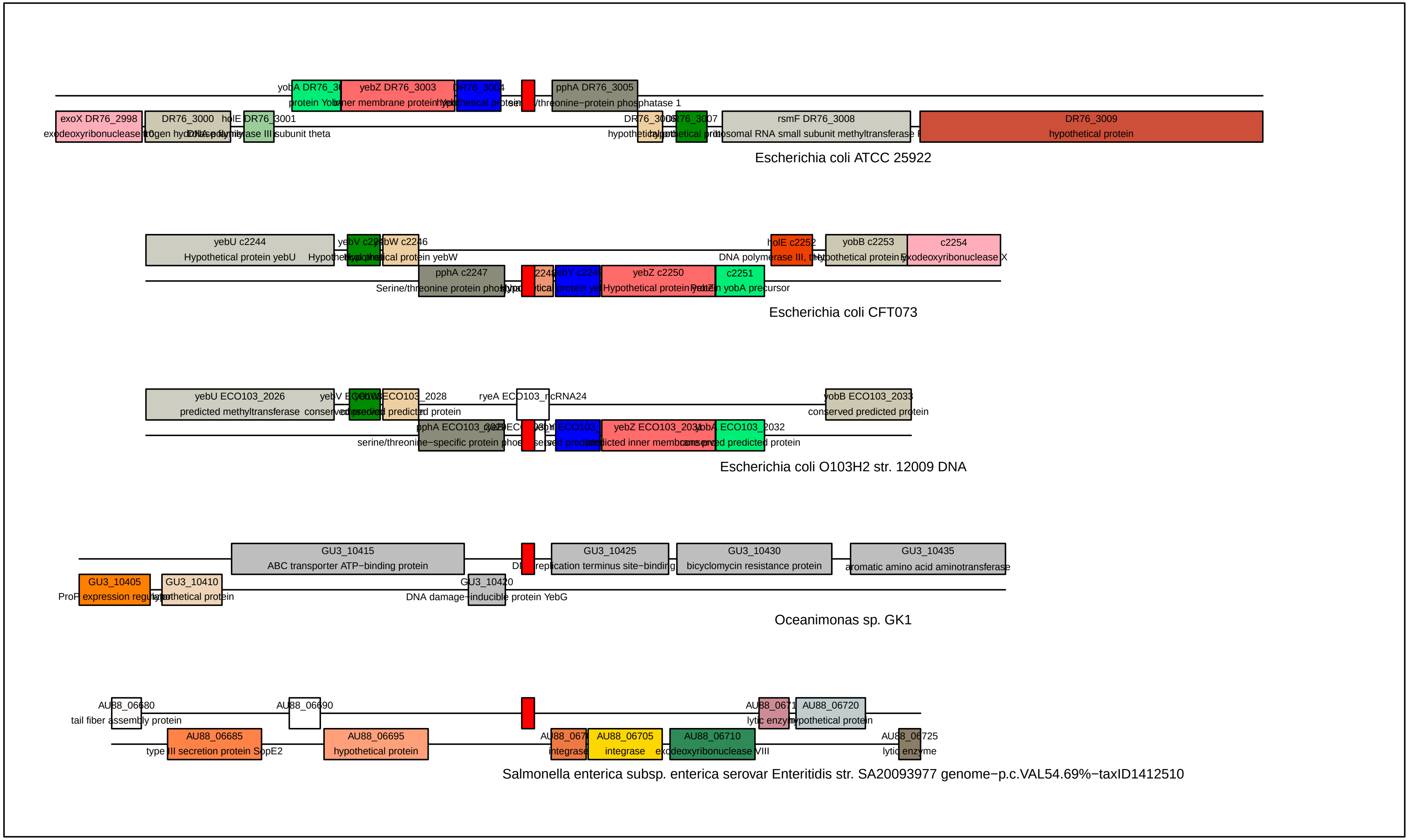

exoX DR76_2998
DR76_3000
yebZ DR76_3003
pphA DR76_3005
rsmF DR76_3008
DR76_3009
hypothetical protein
Escherichia coli ATCC 25922
yebU c2244
Hypothetical protein yebU
pphA c2247
yebZ c2250
c2251
yobB c2253
c2254
Escherichia coli CFT073
yebU ECO103_2026
predicted methyltransferase
yebZ ECO103_2031
yobB ECO103_2033
conserved predicted protein
Escherichia coli O103H2 str. 12009 DNA
GU3_10415
ABC transporter ATP−binding protein
GU3_10425
GU3_10430
bicyclomycin resistance protein
GU3_10435
aromatic amino acid aminotransferase
GU3_10405
GU3_10410
GU3_10420
DNA damage−inducible protein YebG
Oceanimonas sp. GK1
AU88_06685
AU88_06695
hypothetical protein
AU88_06705
integrase
AU88_06710
AU88_06720
Salmonella enterica subsp. enterica serovar Enteritidis str. SA20093977 genome−p.c.VAL54.69%−taxID1412510

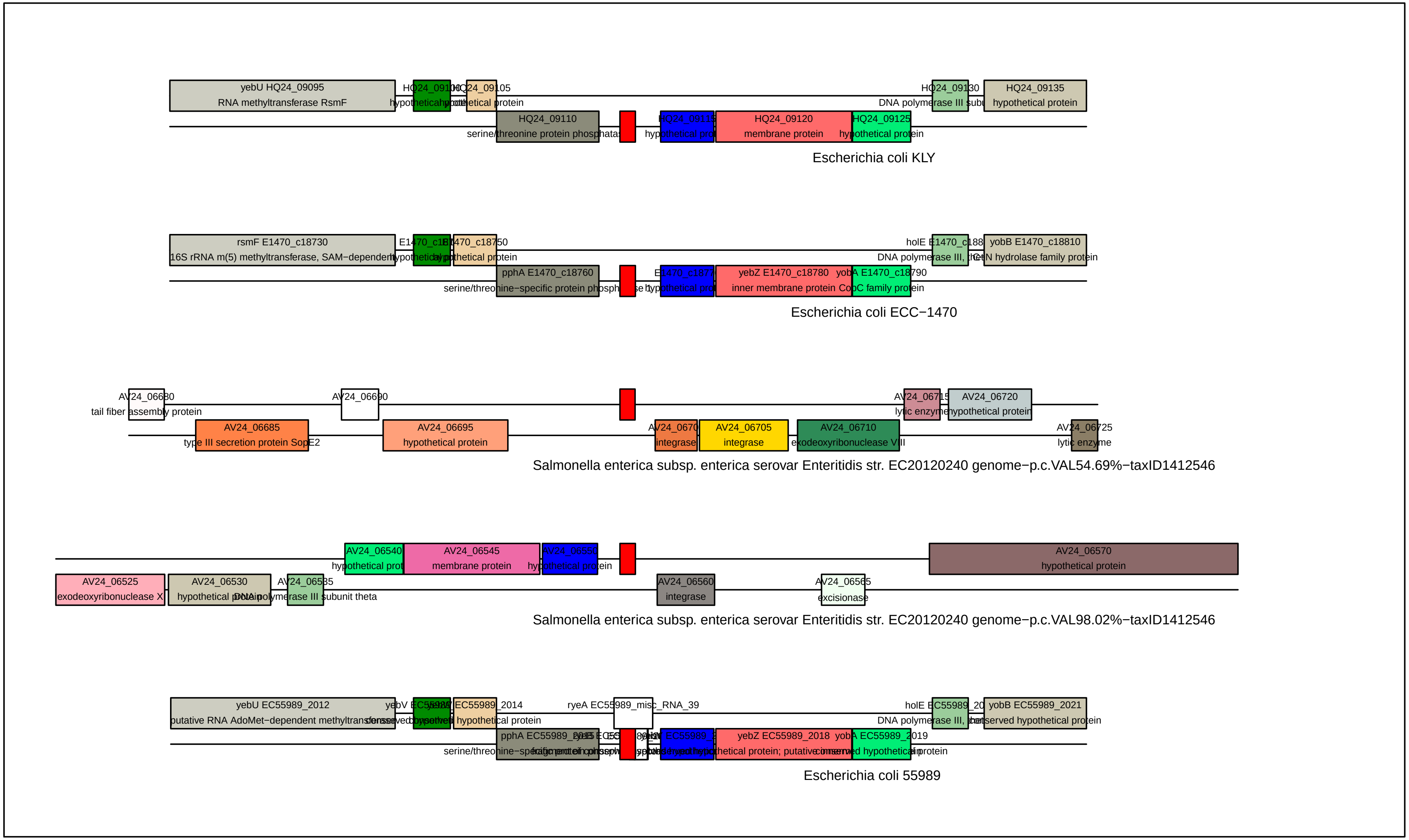

yebU HQ24_09095
RNA methyltransferase RsmF
HQ24_09110
serine/threonine protein phosphatas
HQ24_09120
membrane protein
HQ24_09125
hypothetical protein
HQ24_09135
hypothetical protein
Escherichia coli KLY
rsmF E1470_c18730
16S rRNA m(5) methyltransferase, SAM−dependen
pphA E1470_c18760
serine/threonine−specific protein phosph
yebZ E1470_c18780
inner membrane protein CopC family protein
holE
yobB E1470_c18810
Escherichia coli ECC−1470
AV24_06680
tail fiber assembly protein
AV24_06685
type III secretion protein SopE2
AV24_06690
AV24_06695
hypothetical protein
AV24_06705
integrase
AV24_06710
exodeoxyribonuclease VIII
AV24_06720
hypothetical protein
AV24_06725
lytic enzyme
Salmonella enterica subsp. enterica serovar Enteritidis str. EC20120240 genome−p.c.VAL54.69%−taxID1412546
AV24_06525
exodeoxyribonuclease X
AV24_06530
AV24_06540
AV24_06545
membrane protein
AV24_06560
integrase
AV24_06565
excisionase
AV24_06570
hypothetical protein
Salmonella enterica subsp. enterica serovar Enteritidis str. EC20120240 genome−p.c.VAL98.02%−taxID1412546
yebU EC55989_2012
putative RNA AdoMet−dependent methyltransferase
ryeA EC55989_misc_RNA_39
yebZ EC55989_2018
yobB EC55989_2021
Escherichia coli 55989

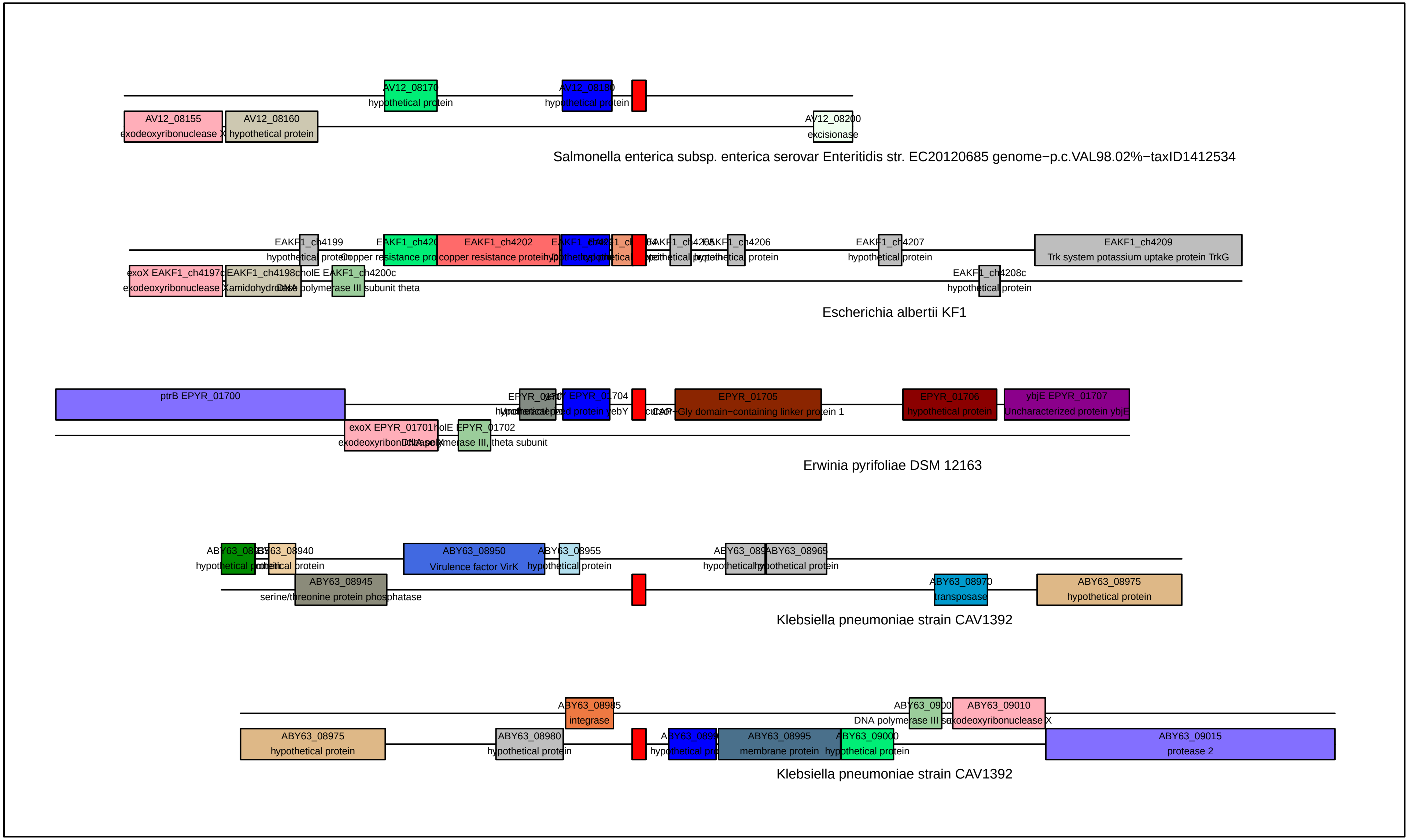

AV12_08170
hypothetical protein
AV12_08155
exodeoxyribonuclease X
AV12_08160
hypothetical protein
AV12_08200
excisionase
Salmonella enterica subsp. enterica serovar Enteritidis str. EC20120685 genome−p.c.VAL98.02%−taxID1412534
EAKF1_ch4199
EAKF1_ch4202
copper resistance protein D
EAKF1_ch4206
EAKF1_ch4207
hypothetical protein
EAKF1_ch4209
Trk system potassium uptake protein TrkG
exoX EAKF1_ch4197c
exodeoxyribonuclease X
EAKF1_ch4198c
holE EAKF1_ch4200c
DNA polymerase III subunit theta
EAKF1_ch4208c
hypothetical protein
Escherichia albertii KF1
ptrB EPYR_01700
EPYR_01704
EPYR_01705
CAP−Gly domain−containing linker protein 1
EPYR_01706
hypothetical protein
ybjE EPYR_01707
Uncharacterized protein ybjE
exoX EPYR_01701
holE EPYR_01702
DNA polymerase III, theta subunit
Erwinia pyrifoliae DSM 12163
ABY63_08940
hypothetical protein
ABY63_08950
Virulence factor VirK
ABY63_08955
hypothetical protein
ABY63_08965
hypothetical protein
ABY63_08945
serine/threonine protein phosphatase
ABY63_08970
transposase
ABY63_08975
hypothetical protein
Klebsiella pneumoniae strain CAV1392
ABY63_08985
integrase
ABY63_09010
exodeoxyribonuclease X
ABY63_08975
hypothetical protein
ABY63_08980
hypothetical protein
ABY63_08995
membrane protein
ABY63_09000
hypothetical protein
ABY63_09015
protease 2
Klebsiella pneumoniae strain CAV1392

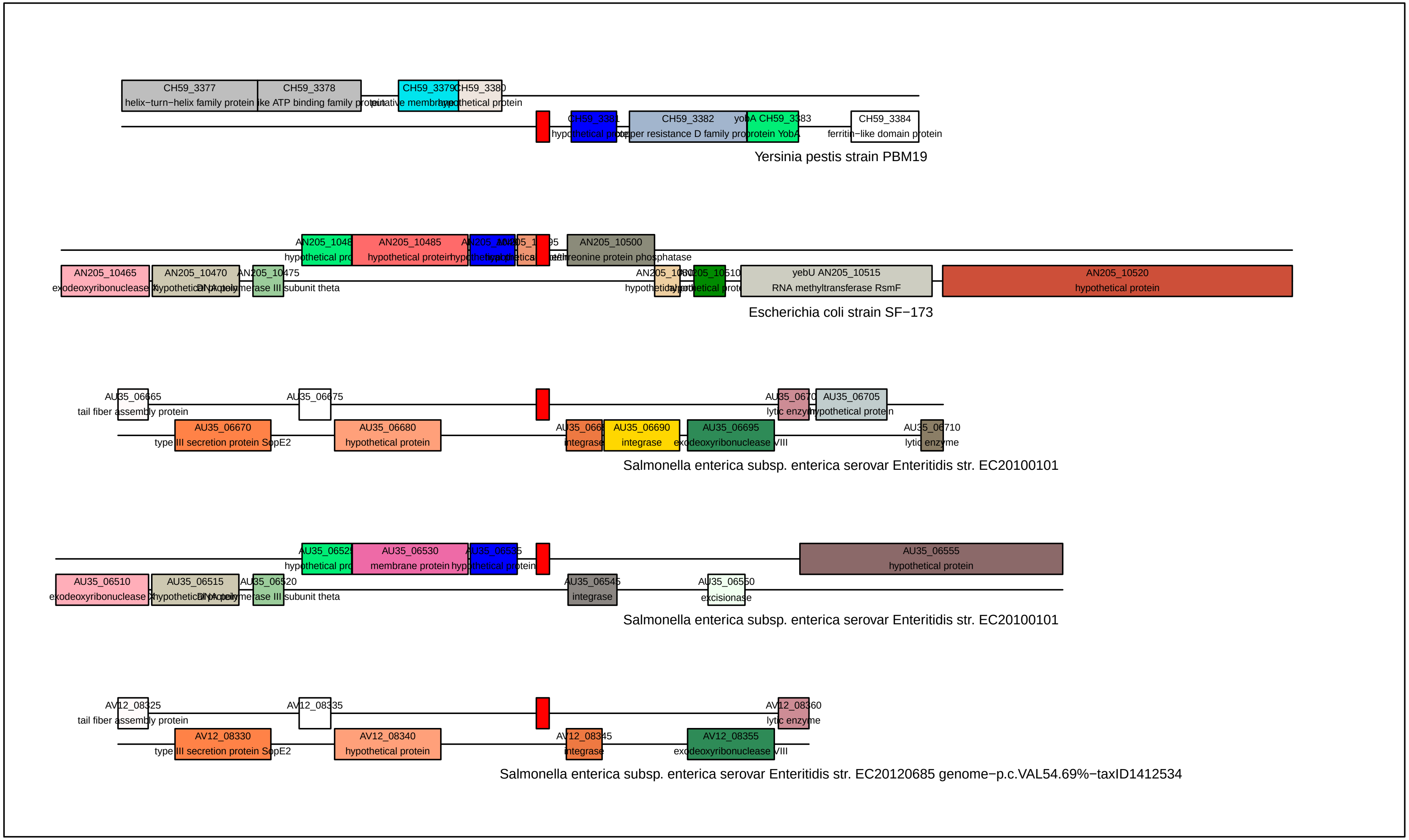

CH59_3377
helix−turn−helix family protein
CH59_3378
CH59_3382
CH59_3384
ferritin−like domain protein
Yersinia pestis strain PBM19
AN205_10485
hypothetical protein
AN205_10500
AN205_10465
AN205_10470
yebU AN205_10515
RNA methyltransferase RsmF
AN205_10520
hypothetical protein
Escherichia coli strain SF−173
tail fiber assembly protein
AU35_06670
type III secretion protein SopE2
AU35_06680
hypothetical protein
AU35_06690
integrase
AU35_06695
AU35_06705
Salmonella enterica subsp. enterica serovar Enteritidis str. EC20100101
AU35_06530
membrane protein
AU35_06555
hypothetical protein
AU35_06510
AU35_06515
integrase
excisionase
Salmonella enterica subsp. enterica serovar Enteritidis str. EC20100101
tail fiber assembly protein
AV12_08330
type III secretion protein SopE2
AV12_08340
hypothetical protein
integrase
AV12_08355
Salmonella enterica subsp. enterica serovar Enteritidis str. EC20120685 genome−p.c.VAL54.69%−taxID1412534

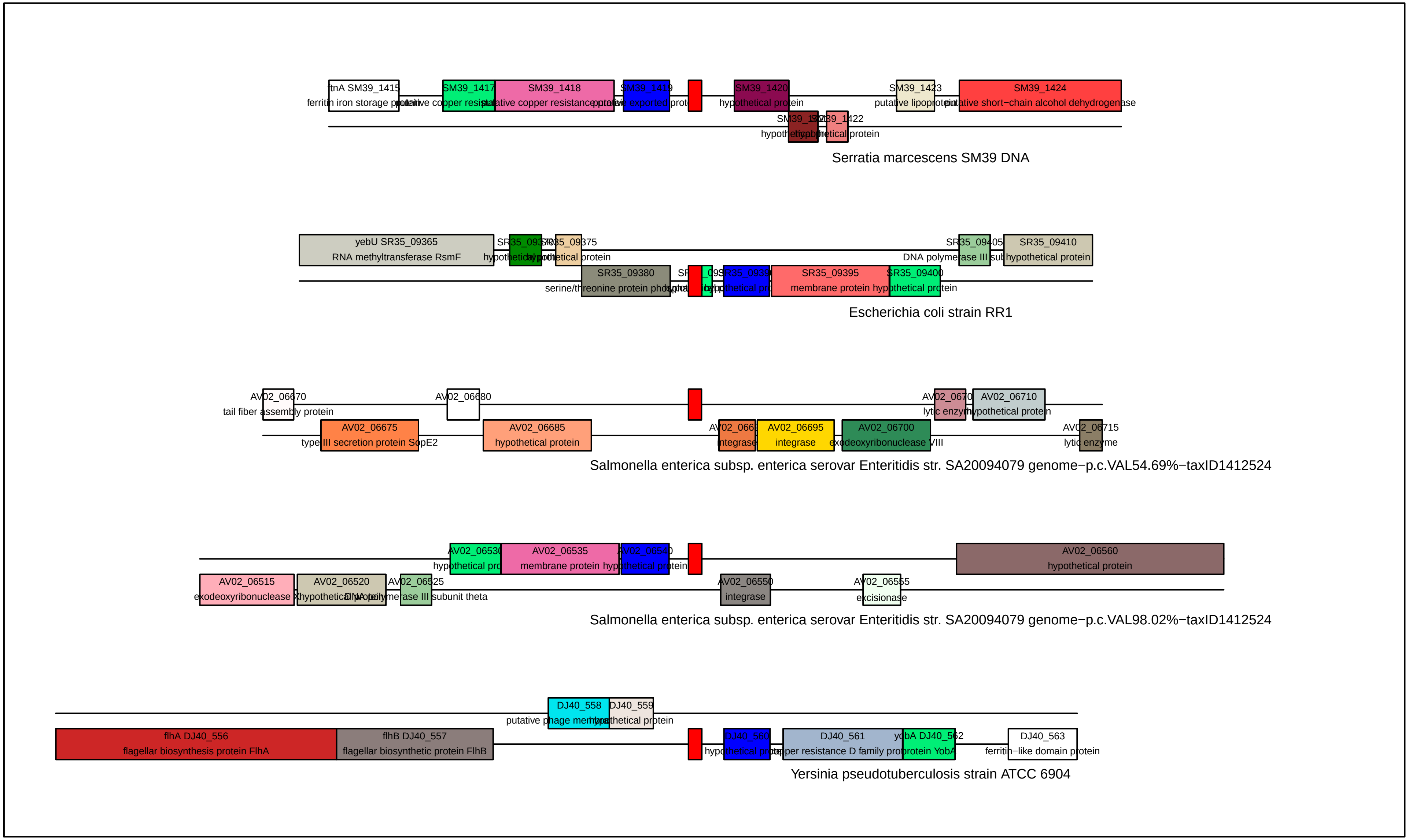

SM39_1418
putative copper resistance protein
SM39_1420
hypothetical protein
SM39_1423
SM39_1424
putative short−chain alcohol dehydrogenase
SM39_1422
Serratia marcescens SM39 DNA
yebU SR35_09365
RNA methyltransferase RsmF
SR35_09375
SR35_09380
SR35_09395
membrane protein
SR35_09400
SR35_09405
SR35_09410
hypothetical protein
Escherichia coli strain RR1
AV02_06670
tail fiber assembly protein
AV02_06680
AV02_06675
type III secretion protein SopE2
AV02_06685
hypothetical protein
AV02_06695
integrase
AV02_06700
exodeoxyribonuclease VIII
AV02_06710
hypothetical protein
AV02_06715
lytic enzyme
Salmonella enterica subsp. enterica serovar Enteritidis str. SA20094079 genome−p.c.VAL54.69%−taxID1412524
AV02_06535
membrane protein
AV02_06540
hypothetical protein
AV02_06560
hypothetical protein
AV02_06515
exodeoxyribonuclease
AV02_06520
AV02_06525
DNA polymerase III subunit theta
AV02_06550
integrase
AV02_06555
excisionase
Salmonella enterica subsp. enterica serovar Enteritidis str. SA20094079 genome−p.c.VAL98.02%−taxID1412524
DJ40_558
DJ40_559
hypothetical protein
flhA DJ40_556
flagellar biosynthesis protein FlhA
flhB DJ40_557
flagellar biosynthetic protein FlhB
DJ40_561
DJ40_563
ferritin−like domain protein
Yersinia pseudotuberculosis strain ATCC 6904

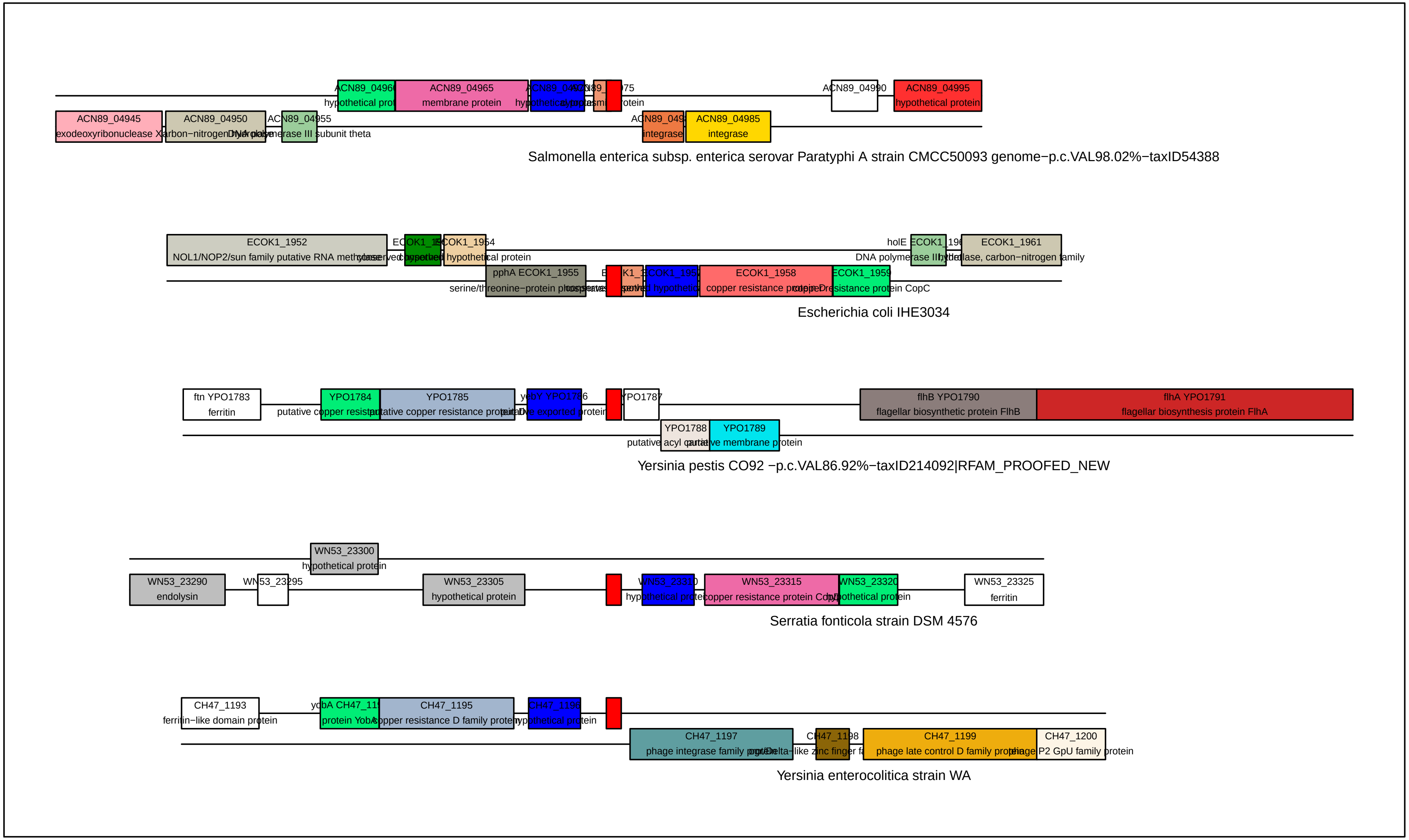
ACN89_04945
exodeoxyribonuclease X
ACN89_04950
ACN89_04955
ACN89_04965
membrane protein
ACN89_04985
integrase
ACN89_04990
ACN89_04995
hypothetical protein
Salmonella enterica subsp. enterica serovar Paratyphi A strain CMCC50093 genome−p.c.VAL98.02%−taxID54388
ECOK1_1952
NOL1/NOP2/sun family putative RNA methylase
pphA ECOK1_1955
ECOK1_1958
ECOK1_1959
copper resistance protein CopC
holE
ECOK1_1961
Escherichia coli IHE3034
ftn YPO1783
ferritin
YPO1784
YPO1785
YPO1788
YPO1789
flhB YPO1790
flagellar biosynthetic protein FlhB
flhA YPO1791
flagellar biosynthesis protein FlhA
Yersinia pestis CO92 −p.c.VAL86.92%−taxID214092|RFAM_PROOFED_NEW
WN53_23290
endolysin
WN53_23295
WN53_23300
hypothetical protein
WN53_23305
hypothetical protein
WN53_23315
WN53_23325
ferritin
Serratia fonticola strain DSM 4576
CH47_1193
ferritin−like domain protein
CH47_1195
CH47_1197
CH47_1199
phage late control D family protein
CH47_1200
P2 GpU family protein
Yersinia enterocolitica strain WA

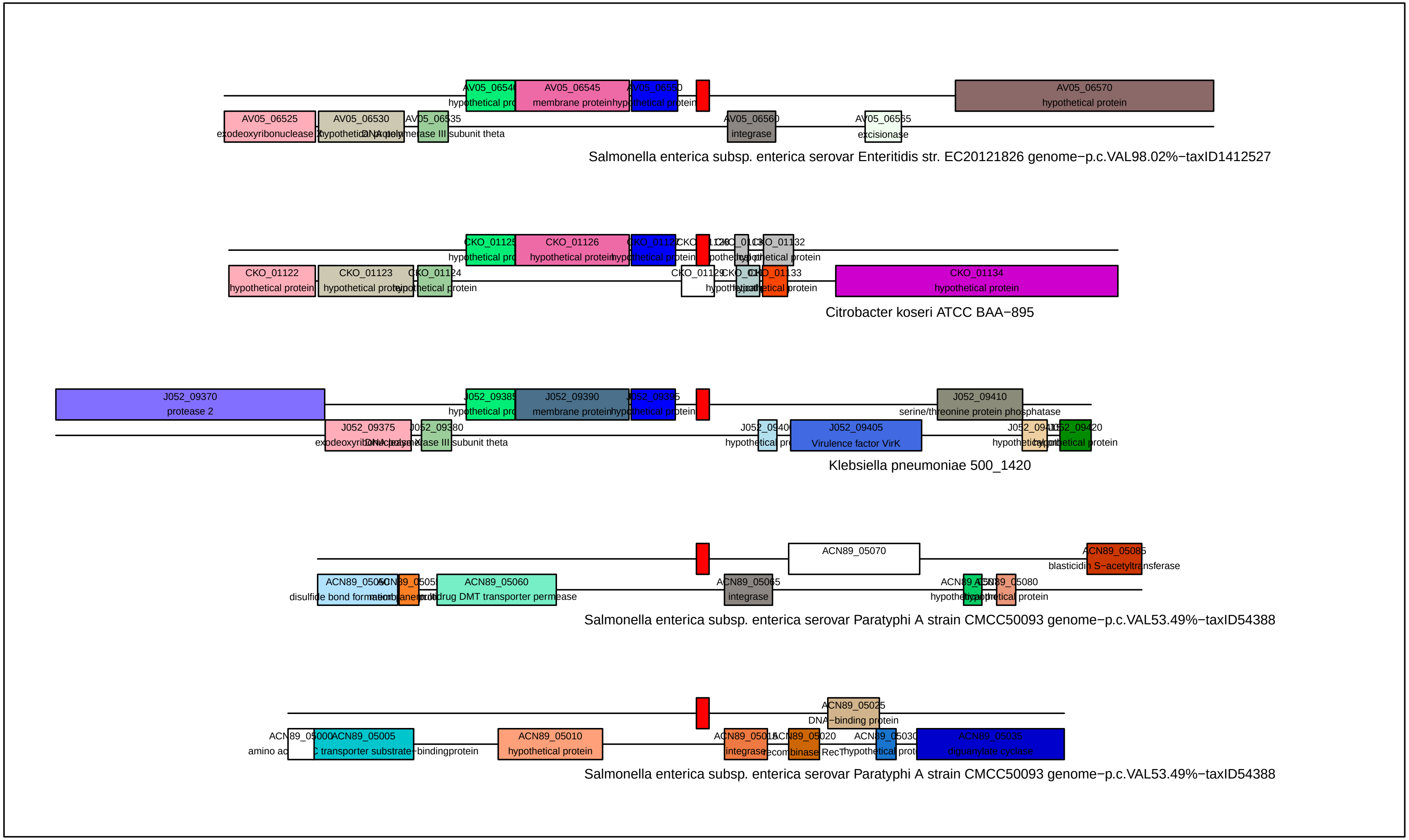

AV05_06545
membrane protein
AV05_06570
hypothetical protein
AV05_06525
AV05_06530
AV05_06560
integrase
excisionase
Salmonella enterica subsp. enterica serovar Enteritidis str. EC20121826 genome−p.c.VAL98.02%−taxID1412527
CKO_01126
hypothetical protein
CKO_01122
hypothetical protein
CKO_01123
CKO_01134
hypothetical protein
Citrobacter koseri ATCC BAA−895
J052_09370
protease 2
J052_09390
membrane protein
J052_09410
serine/threonine protein phosphatase
J052_09375
J052_09405
Virulence factor VirK
Klebsiella pneumoniae 500_1420
ACN89_05070
ACN89_05060
ACN89_05065
integrase
Salmonella enterica subsp. enterica serovar Paratyphi A strain CMCC50093 genome−p.c.VAL53.49%−taxID54388
ACN89_05025
DNA−binding protein
ACN89_05010
hypothetical protein
diguanylate cyclase
Salmonella enterica subsp. enterica serovar Paratyphi A strain CMCC50093 genome−p.c.VAL53.49%−taxID54388

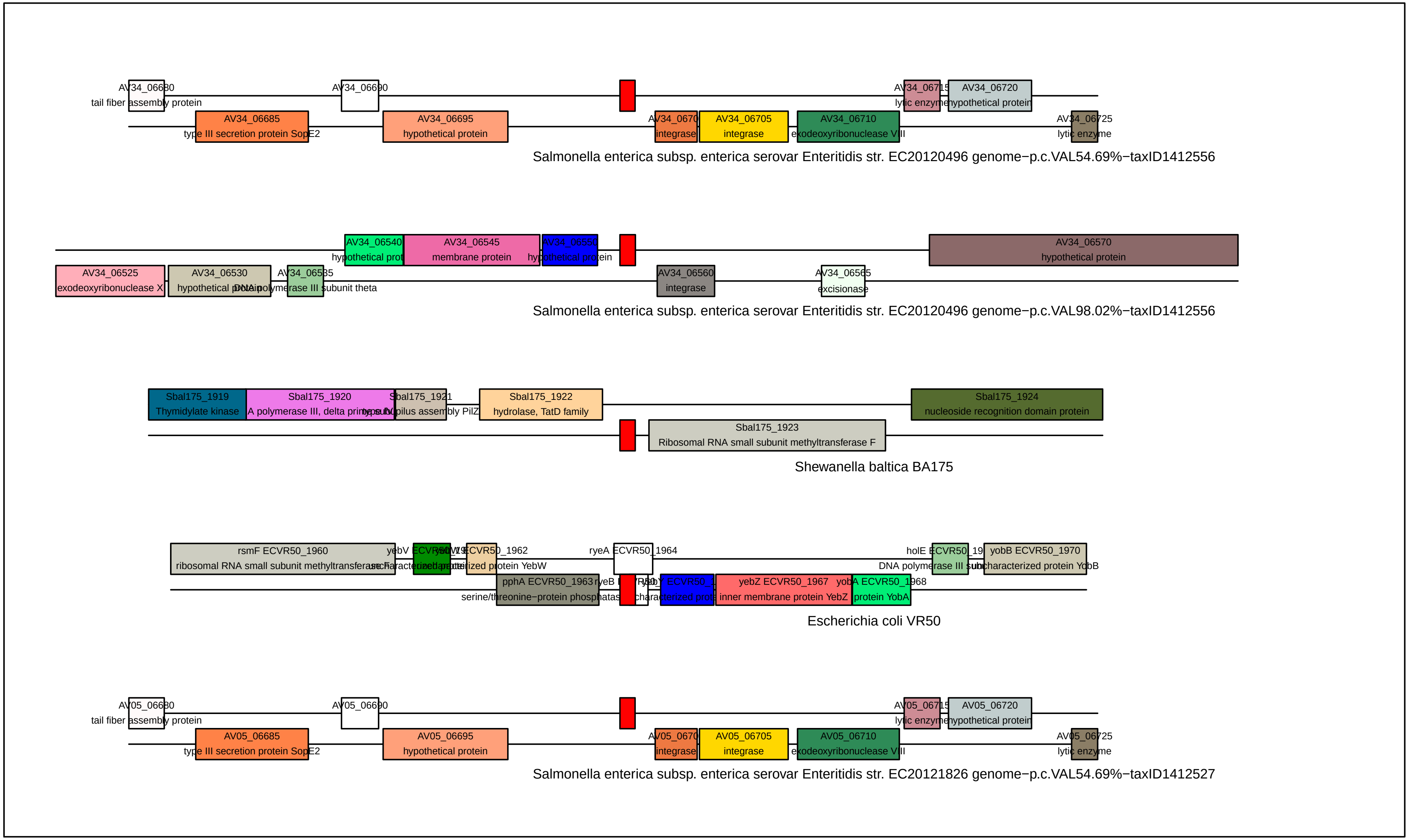
AV34_06680
tail fiber assembly protein
AV34_06690
AV34_06715
lytic enzyme
AV34_06720
hypothetical protein
AV34_06685
type III secretion protein SopE2
AV34_06695
hypothetical protein
integrase
AV34_06705
integrase
AV34_06710
exodeoxyribonuclease VIII
AV34_06725
lytic enzyme
Salmonella enterica subsp. enterica serovar Enteritidis str. EC20120496 genome−p.c.VAL54.69%−taxID1412556
AV34_06540
AV34_06545
membrane protein
AV34_06570
hypothetical protein
AV34_06525
exodeoxyribonuclease X
AV34_06530
AV34_06535
AV34_06560
integrase
AV34_06565
excisionase
Salmonella enterica subsp. enterica serovar Enteritidis str. EC20120496 genome−p.c.VAL98.02%−taxID1412556
Sbal175_1919
Thymidylate kinase
Sbal175_1920
Sbal175_1921
Sbal175_1922
hydrolase, TatD family
Sbal175_1924
nucleoside recognition domain protein
Sbal175_1923
Ribosomal RNA small subunit methyltransferase F
Shewanella baltica BA175
rsmF ECVR50_1960
ryeA ECVR50_1964
yobB ECVR50_1970
pphA ECVR50_1963
yebZ ECVR50_1967
inner membrane protein YebZ
protein YobA
Escherichia coli VR50
AV05_06680
tail fiber assembly protein
AV05_06690
AV05_06715
lytic enzyme
AV05_06720
hypothetical protein
AV05_06685
type III secretion protein SopE2
AV05_06695
hypothetical protein
integrase
AV05_06705
integrase
AV05_06710
exodeoxyribonuclease VIII
AV05_06725
lytic enzyme
Salmonella enterica subsp. enterica serovar Enteritidis str. EC20121826 genome−p.c.VAL54.69%−taxID1412527

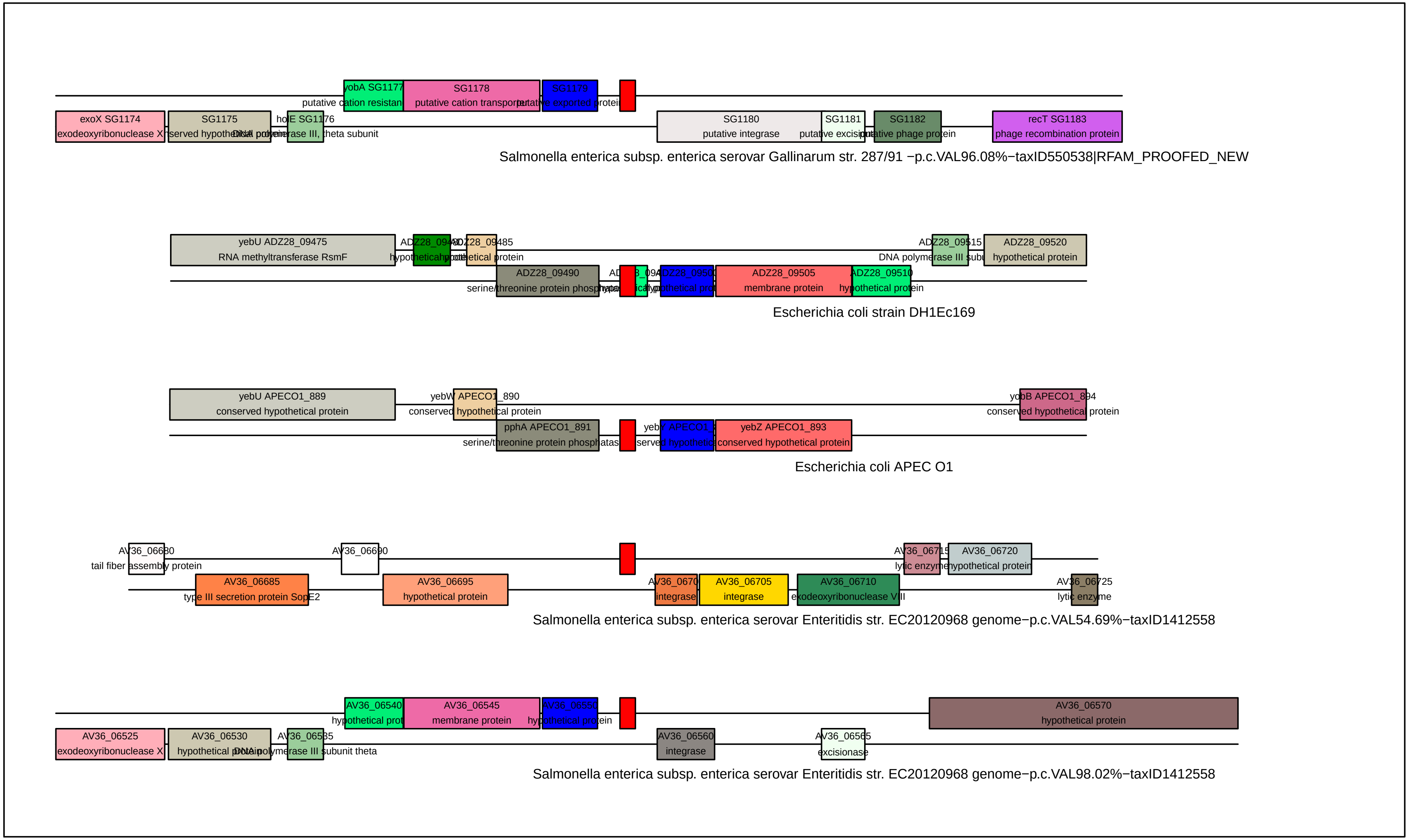
SG1178
putative cation transporter
SG1179
exoX SG1174
exodeoxyribonuclease X
SG1175
SG1180
putative integrase
SG1181
SG1182
recT SG1183
phage recombination protein
Salmonella enterica subsp. enterica serovar Gallinarum str. 287/91 −p.c.VAL96.08%−taxID550538|RFAM_PROOFED_NEW
yebU ADZ28_09475
RNA methyltransferase RsmF
ADZ28_09520
hypothetical protein
ADZ28_09490
ADZ28_09505
membrane protein
ADZ28_09510
Escherichia coli strain DH1Ec169
yebU APECO1_889
conserved hypothetical protein
pphA APECO1_891
yebZ APECO1_893
conserved hypothetical protein
Escherichia coli APEC O1
AV36_06685
AV36_06695
hypothetical protein
AV36_06705
integrase
AV36_06710
AV36_06720
Salmonella enterica subsp. enterica serovar Enteritidis str. EC20120968 genome−p.c.VAL54.69%−taxID1412558
AV36_06540
AV36_06545
membrane protein
AV36_06570
hypothetical protein
AV36_06525
exodeoxyribonuclease X
AV36_06530
AV36_06560
integrase
AV36_06565
excisionase
Salmonella enterica subsp. enterica serovar Enteritidis str. EC20120968 genome−p.c.VAL98.02%−taxID1412558

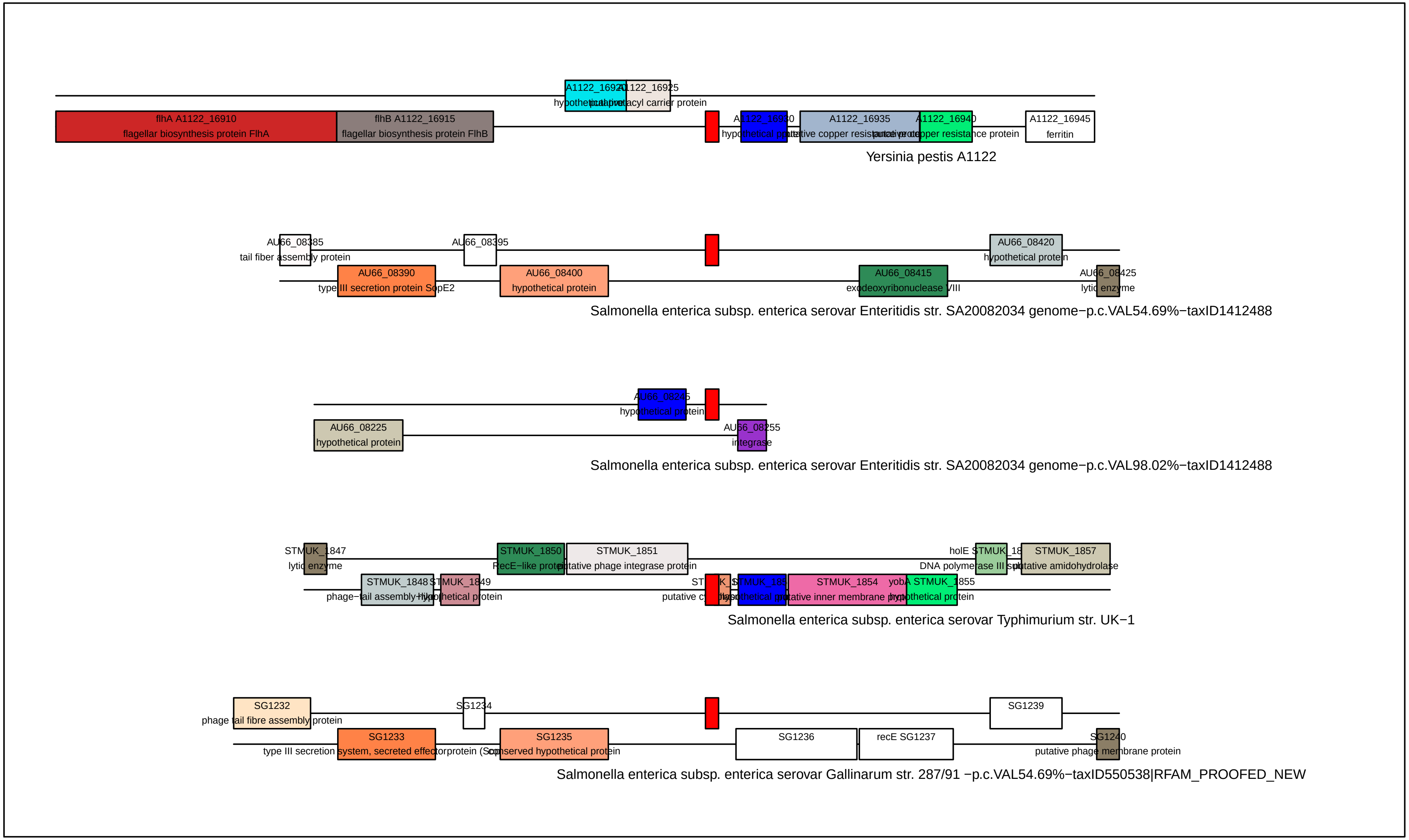

flhA A1122_16910
flagellar biosynthesis protein FlhA
flhB A1122_16915
flagellar biosynthesis protein FlhB
A1122_16935
A1122_16945
ferritin
Yersinia pestis A1122
tail fiber assembly protein
AU66_08390
type III secretion protein SopE2
AU66_08400
hypothetical protein
AU66_08415
exodeoxyribonuclease VIII
AU66_08420
lytic enzyme
Salmonella enterica subsp. enterica serovar Enteritidis str. SA20082034 genome−p.c.VAL54.69%−taxID1412488
AU66_08225
hypothetical protein
integrase
Salmonella enterica subsp. enterica serovar Enteritidis str. SA20082034 genome−p.c.VAL98.02%−taxID1412488
STMUK_1850
STMUK_1851
putative phage integrase protein
STMUK_1857
putative amidohydrolase
STMUK_1848
STMUK_1854
putative inner membrane protein
Salmonella enterica subsp. enterica serovar Typhimurium str. UK−1
SG1232
phage tail fibre assembly protein
SG1239
SG1233
SG1235
conserved hypothetical protein
SG1236
recE SG1237
putative phage membrane protein
Salmonella enterica subsp. enterica serovar Gallinarum str. 287/91 −p.c.VAL54.69%−taxID550538|RFAM_PROOFED_NEW

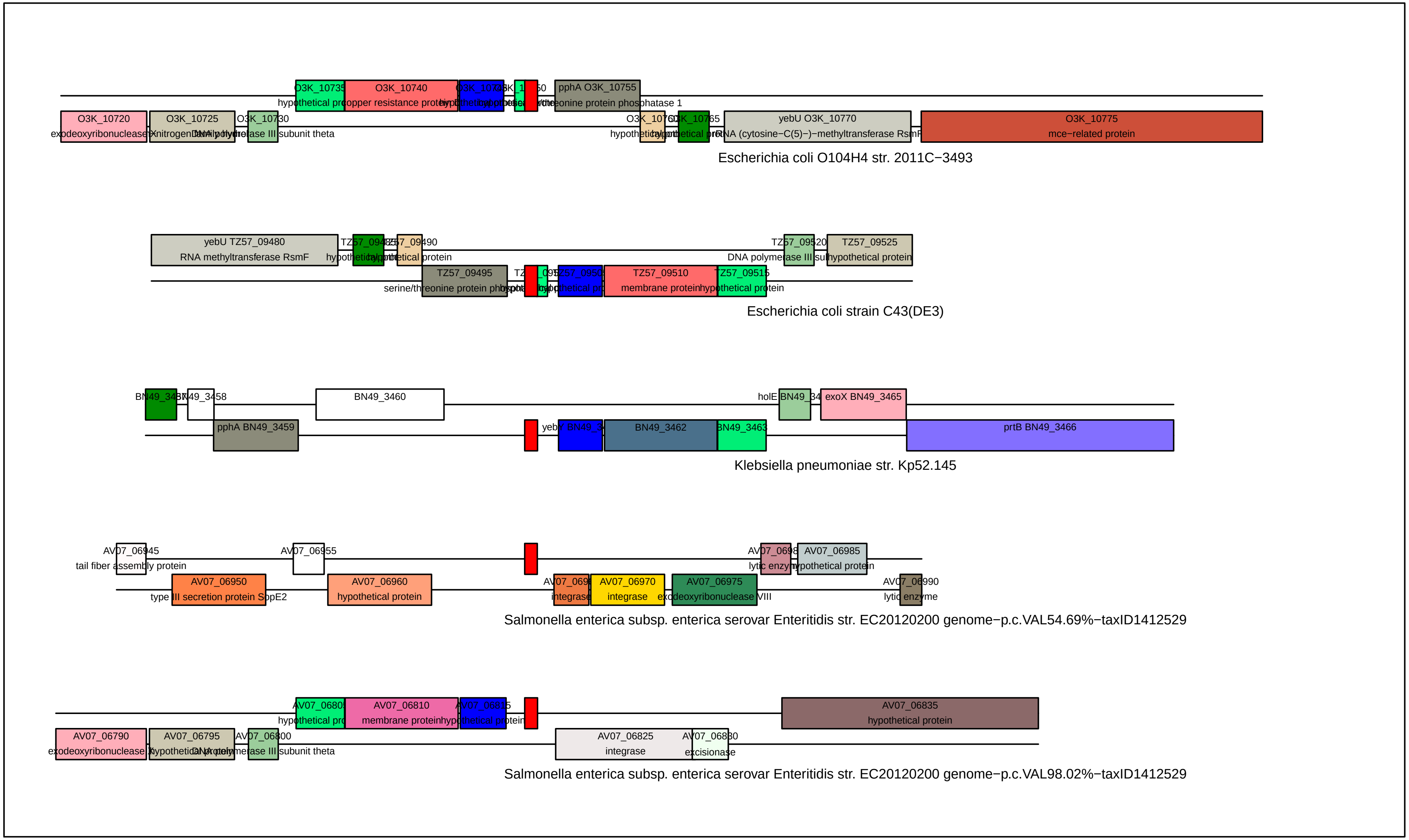
O3K_10740
pphA O3K_10755
O3K_10720
O3K_10725
yebU O3K_10770
O3K_10775
mce−related protein
Escherichia coli O104H4 str. 2011C−3493
yebU TZ57_09480
RNA methyltransferase RsmF
TZ57_09525
TZ57_09495
TZ57_09510
membrane protein
Escherichia coli strain C43(DE3)
BN49_3460
pphA BN49_3459
BN49_3462
exoX BN49_3465
prtB BN49_3466
Klebsiella pneumoniae str. Kp52.145
AV07_06945
tail fiber assembly protein
AV07_06955
AV07_06985
AV07_06950
type III secretion protein SopE2
AV07_06960
hypothetical protein
AV07_06970
integrase
AV07_06975
Salmonella enterica subsp. enterica serovar Enteritidis str. EC20120200 genome−p.c.VAL54.69%−taxID1412529
AV07_06810
membrane protein
AV07_06835
hypothetical protein
AV07_06790
AV07_06795
AV07_06825
integrase
excisionase
Salmonella enterica subsp. enterica serovar Enteritidis str. EC20120200 genome−p.c.VAL98.02%−taxID1412529

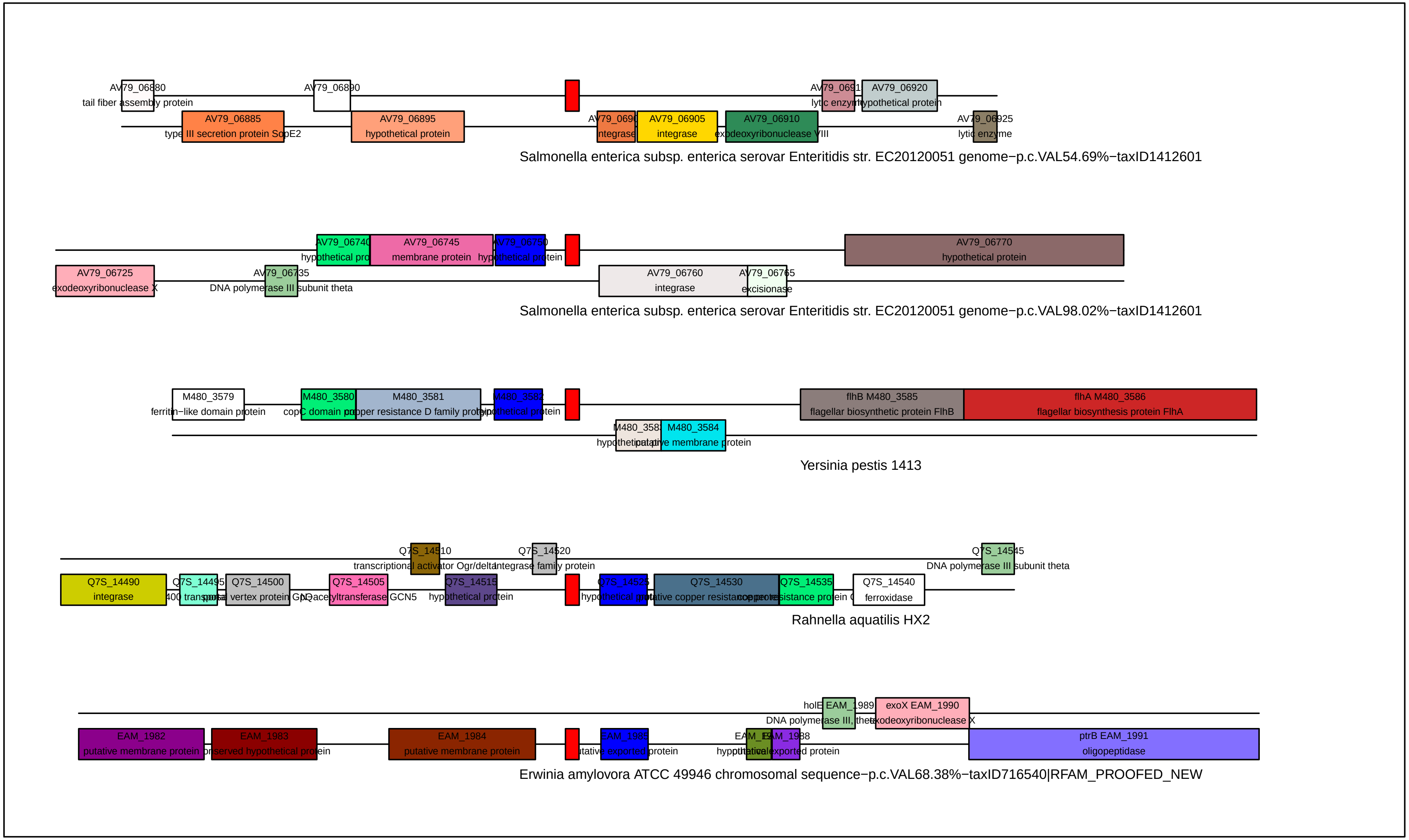
AV79_06880
tail fiber assembly protein
AV79_06890
AV79_06920
hypothetical protein
AV79_06885
type III secretion protein SopE2
AV79_06895
hypothetical protein
integrase
AV79_06905
integrase
AV79_06910
exodeoxyribonuclease VIII
AV79_06925
lytic enzyme
Salmonella enterica subsp. enterica serovar Enteritidis str. EC20120051 genome−p.c.VAL54.69%−taxID1412601
AV79_06745
membrane protein
AV79_06770
hypothetical protein
AV79_06725
exodeoxyribonuclease X
AV79_06735
DNA polymerase III subunit theta
AV79_06760
integrase
AV79_06765
excisionase
Salmonella enterica subsp. enterica serovar Enteritidis str. EC20120051 genome−p.c.VAL98.02%−taxID1412601
M480_3579
ferritin−like domain protein
M480_3580
M480_3581
flhB M480_3585
flagellar biosynthetic protein FlhB
flhA M480_3586
flagellar biosynthesis protein FlhA
M480_3584
Yersinia pestis 1413
Q7S_14510
Q7S_14520
Q7S_14545
DNA polymerase III subunit theta
Q7S_14490
integrase
Q7S_14500
Q7S_14505
Q7S_14515
Q7S_14530
Q7S_14535
Q7S_14540
ferroxidase
Rahnella aquatilis HX2
exoX EAM_1990
EAM_1982
putative membrane protein
EAM_1983
EAM_1984
putative membrane protein
ptrB EAM_1991
oligopeptidase
Erwinia amylovora ATCC 49946 chromosomal sequence−p.c.VAL68.38%−taxID716540|RFAM_PROOFED_NEW

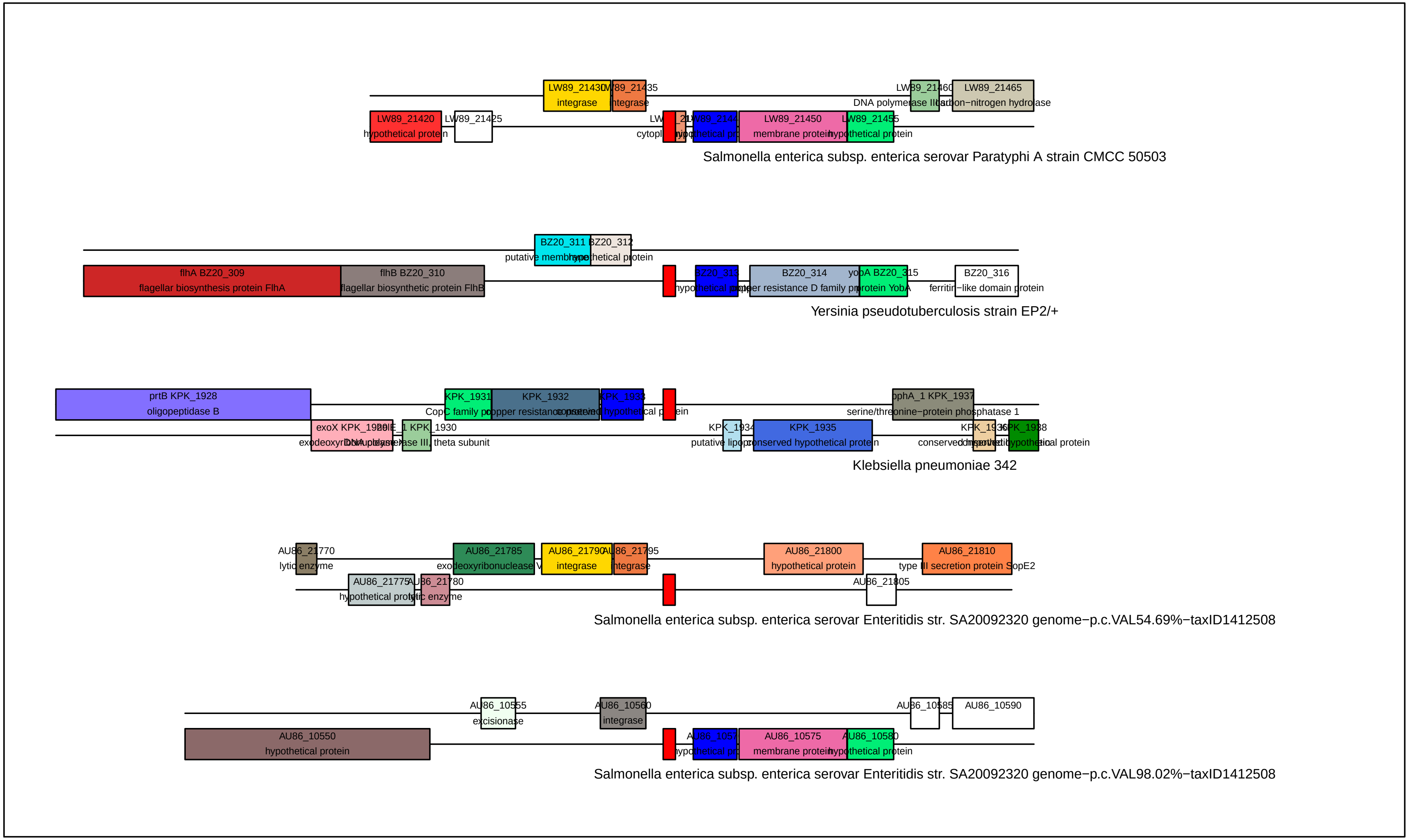
LW89_21420
hypothetical protein
LW89_21425
LW89_21430
integrase
LW89_21450
membrane protein
LW89_21465
Salmonella enterica subsp. enterica serovar Paratyphi A strain CMCC 50503
flhA BZ20_309
flagellar biosynthesis protein FlhA
flhB BZ20_310
flagellar biosynthetic protein FlhB
BZ20_311
BZ20_314
BZ20_316
ferritin−like domain protein
Yersinia pseudotuberculosis strain EP2/+
prtB KPK_1928
oligopeptidase B
KPK_1932
KPK_1935
conserved hypothetical protein
serine/threonine−protein phosphatase 1
Klebsiella pneumoniae 342
AU86_21785
exodeoxyribonuclease
AU86_21800
hypothetical protein
AU86_21810
type III secretion protein SopE2
Salmonella enterica subsp. enterica serovar Enteritidis str. SA20092320 genome−p.c.VAL54.69%−taxID1412508
AU86_10550
hypothetical protein
AU86_10560
integrase
AU86_10575
membrane protein
AU86_10590
Salmonella enterica subsp. enterica serovar Enteritidis str. SA20092320 genome−p.c.VAL98.02%−taxID1412508

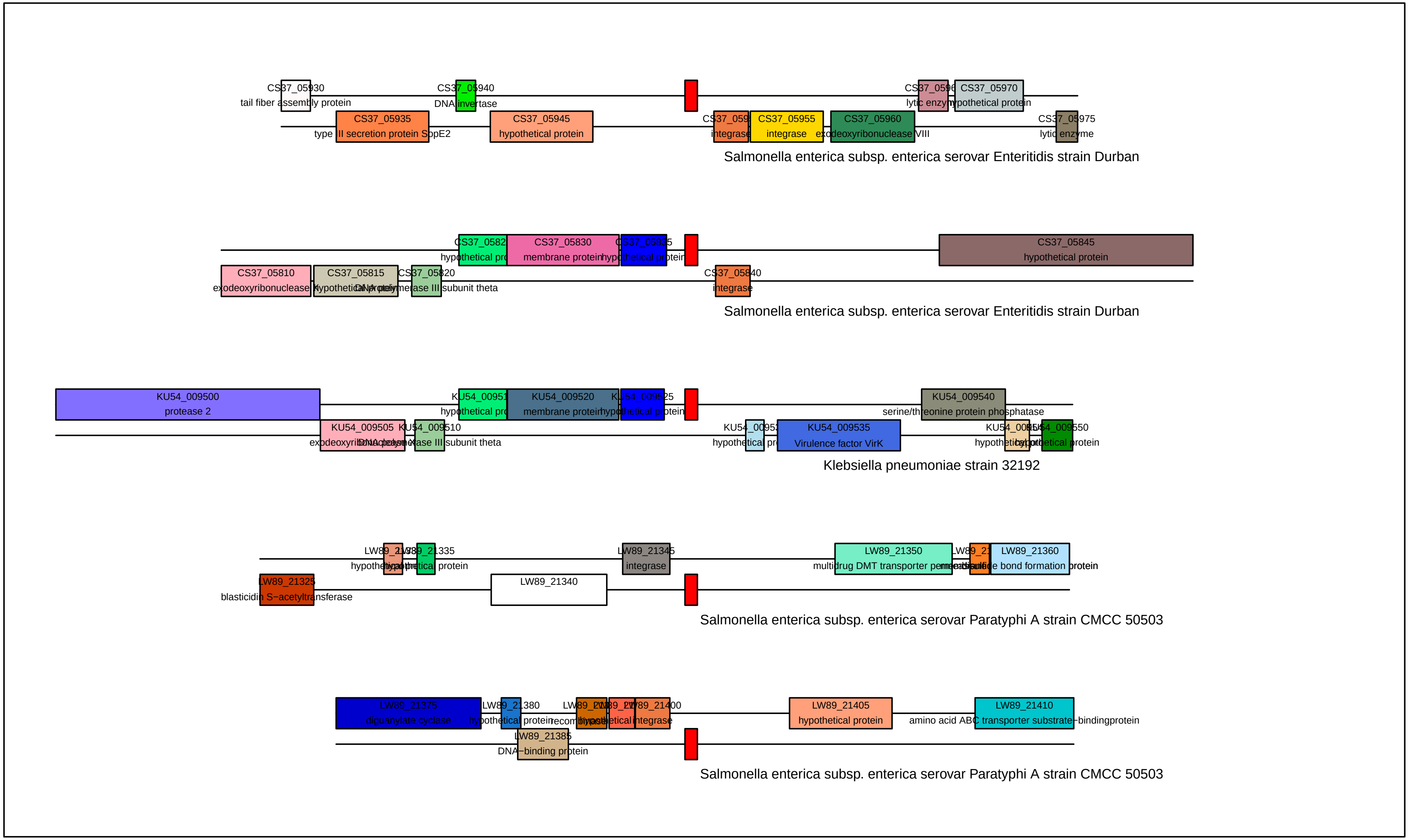
CS37_05930
tail fiber assembly protein
CS37_05935
CS37_05940
CS37_05945
hypothetical protein
CS37_05955
integrase
CS37_05960
CS37_05970
CS37_05975
Salmonella enterica subsp. enterica serovar Enteritidis strain Durban
CS37_05810
CS37_05815
CS37_05830
membrane protein
CS37_05840
integrase
CS37_05845
hypothetical protein
Salmonella enterica subsp. enterica serovar Enteritidis strain Durban
KU54_009500
protease 2
KU54_009505
KU54_009520
membrane protein
KU54_009535
Virulence factor VirK
KU54_009540
Klebsiella pneumoniae strain 32192
LW89_21325
LW89_21335
LW89_21340
LW89_21345
integrase
LW89_21350
LW89_21360
Salmonella enterica subsp. enterica serovar Paratyphi A strain CMCC 50503
LW89_21375
diguanylate cyclase
LW89_21380
LW89_21385
LW89_21400
integrase
LW89_21405
hypothetical protein
LW89_21410
amino acid ABC transporter substrate−bindingprotein
Salmonella enterica subsp. enterica serovar Paratyphi A strain CMCC 50503

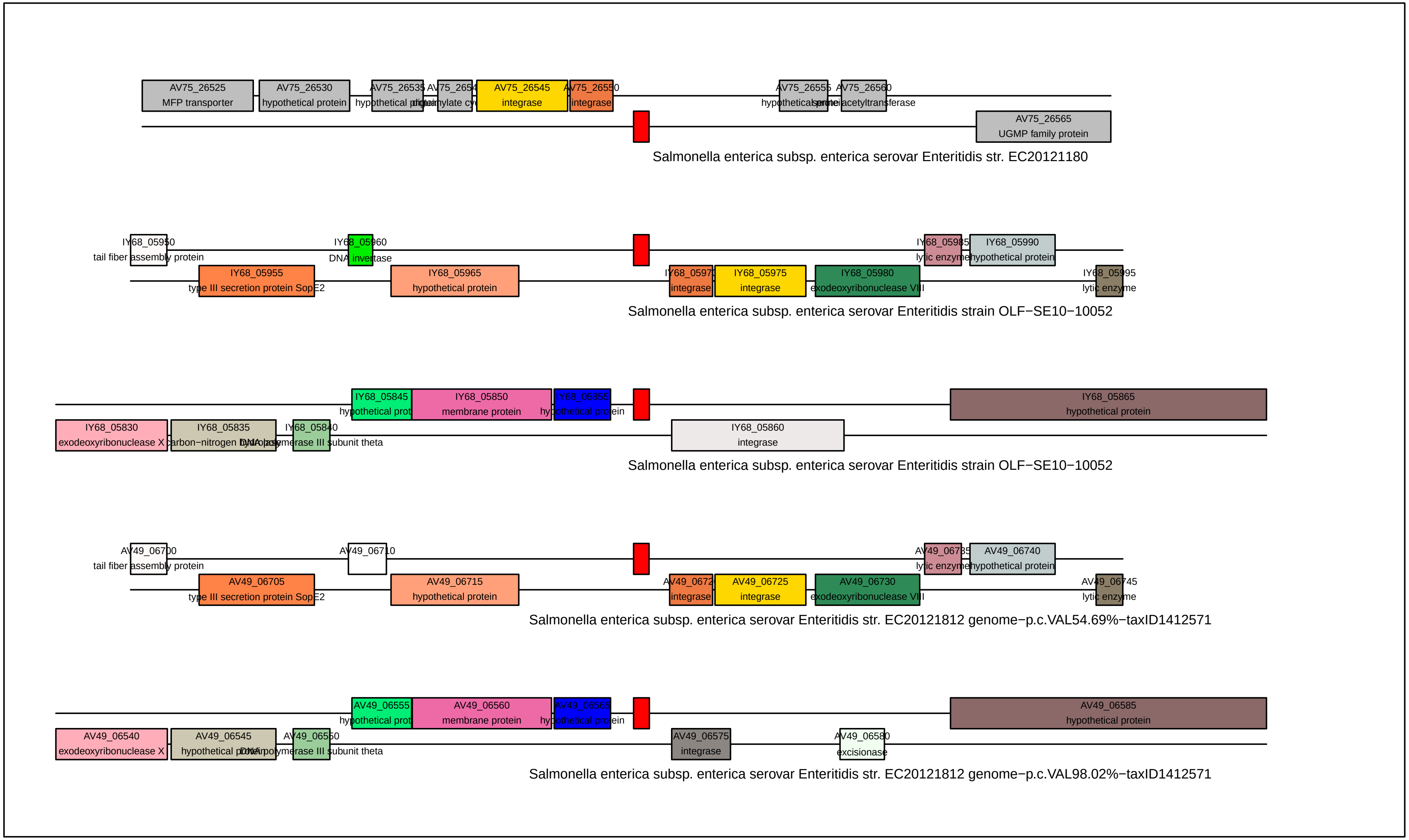

AV75_26525
MFP transporter
AV75_26530
hypothetical protein
AV75_26535
AV75_26545
integrase
AV75_26550
integrase
AV75_26555
AV75_26560
AV75_26565
UGMP family protein
Salmonella enterica subsp. enterica serovar Enteritidis str. EC20121180
IY68_05950
tail fiber assembly protein
IY68_05960
DNA invertase
IY68_05955
type III secretion protein SopE2
IY68_05965
hypothetical protein
integrase
IY68_05975
integrase
IY68_05980
exodeoxyribonuclease VIII
IY68_05985
lytic enzyme
IY68_05990
hypothetical protein
IY68_05995
lytic enzyme
Salmonella enterica subsp. enterica serovar Enteritidis strain OLF−SE10−10052
IY68_05845
IY68_05850
membrane protein
IY68_05865
hypothetical protein
IY68_05830
exodeoxyribonuclease X
IY68_05835
IY68_05840
IY68_05860
integrase
Salmonella enterica subsp. enterica serovar Enteritidis strain OLF−SE10−10052
AV49_06700
tail fiber assembly protein
AV49_06710
AV49_06705
type III secretion protein SopE2
AV49_06715
hypothetical protein
integrase
AV49_06725
integrase
AV49_06730
exodeoxyribonuclease VIII
AV49_06735
lytic enzyme
AV49_06740
hypothetical protein
AV49_06745
lytic enzyme
Salmonella enterica subsp. enterica serovar Enteritidis str. EC20121812 genome−p.c.VAL54.69%−taxID1412571
AV49_06555
AV49_06560
membrane protein
AV49_06585
hypothetical protein
AV49_06540
exodeoxyribonuclease X
AV49_06545
AV49_06550
AV49_06575
integrase
AV49_06580
excisionase
Salmonella enterica subsp. enterica serovar Enteritidis str. EC20121812 genome−p.c.VAL98.02%−taxID1412571

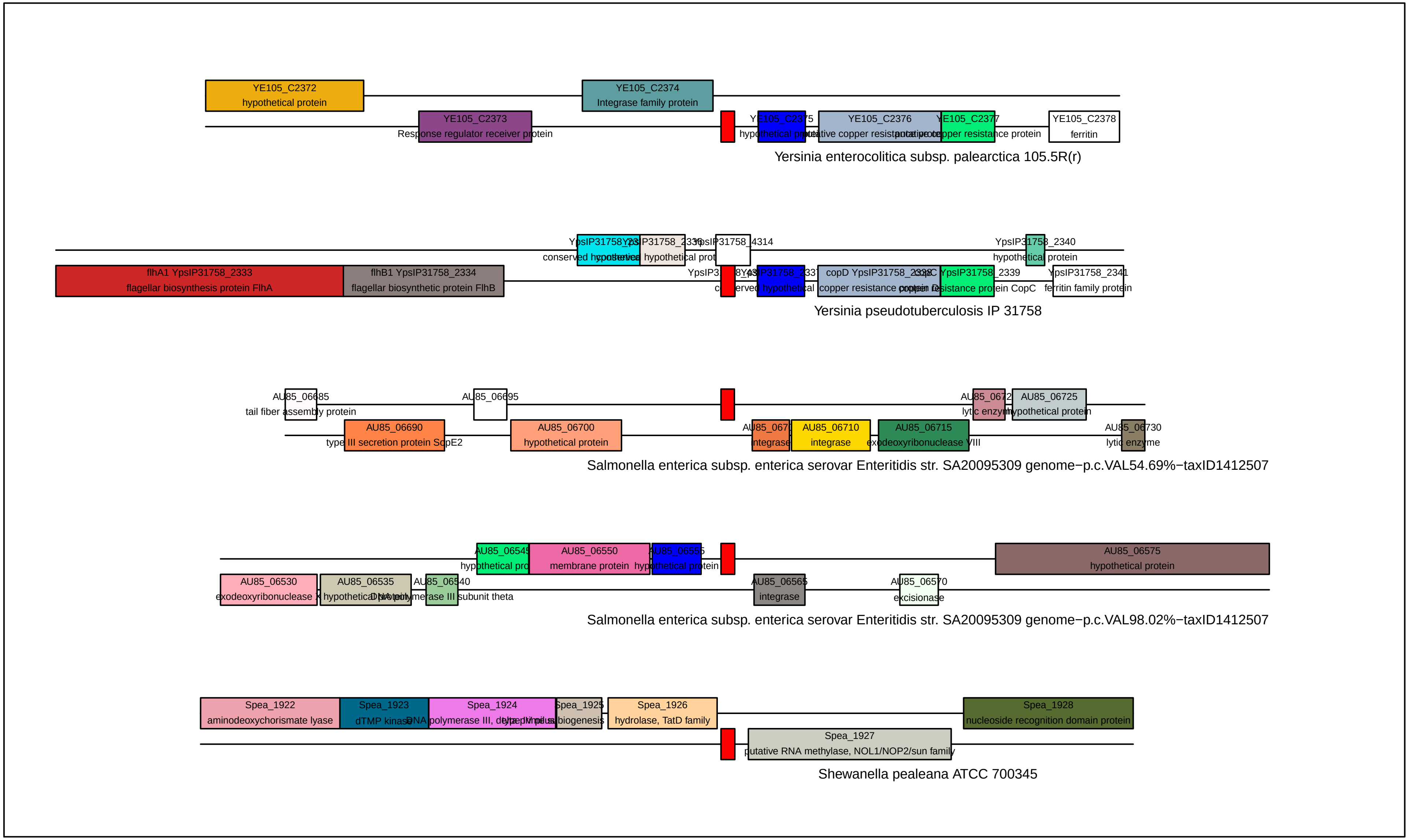
YE105_C2372
hypothetical protein
YE105_C2373
Response regulator receiver protein
YE105_C2374
Integrase family protein
YE105_C2376
YE105_C2378
ferritin
Yersinia enterocolitica subsp. palearctica 105.5R(r)
flhA1 YpsIP31758_2333
flagellar biosynthesis protein FlhA
flhB1 YpsIP31758_2334
flagellar biosynthetic protein FlhB
hypothetical protein
Yersinia pseudotuberculosis IP 31758
tail fiber assembly protein
AU85_06690
type III secretion protein SopE2
AU85_06700
hypothetical protein
AU85_06710
integrase
AU85_06715
exodeoxyribonuclease VIII
AU85_06725
hypothetical protein
lytic enzyme
Salmonella enterica subsp. enterica serovar Enteritidis str. SA20095309 genome−p.c.VAL54.69%−taxID1412507
AU85_06530
exodeoxyribonuclease
AU85_06535
AU85_06550
membrane protein
AU85_06565
integrase
AU85_06570
excisionase
AU85_06575
hypothetical protein
Salmonella enterica subsp. enterica serovar Enteritidis str. SA20095309 genome−p.c.VAL98.02%−taxID1412507
Spea_1922
aminodeoxychorismate lyase
Spea_1923
Spea_1924
Spea_1926
hydrolase, TatD family
Spea_1927
putative RNA methylase, NOL1/NOP2/sun family
Spea_1928
nucleoside recognition domain protein
Shewanella pealeana ATCC 700345

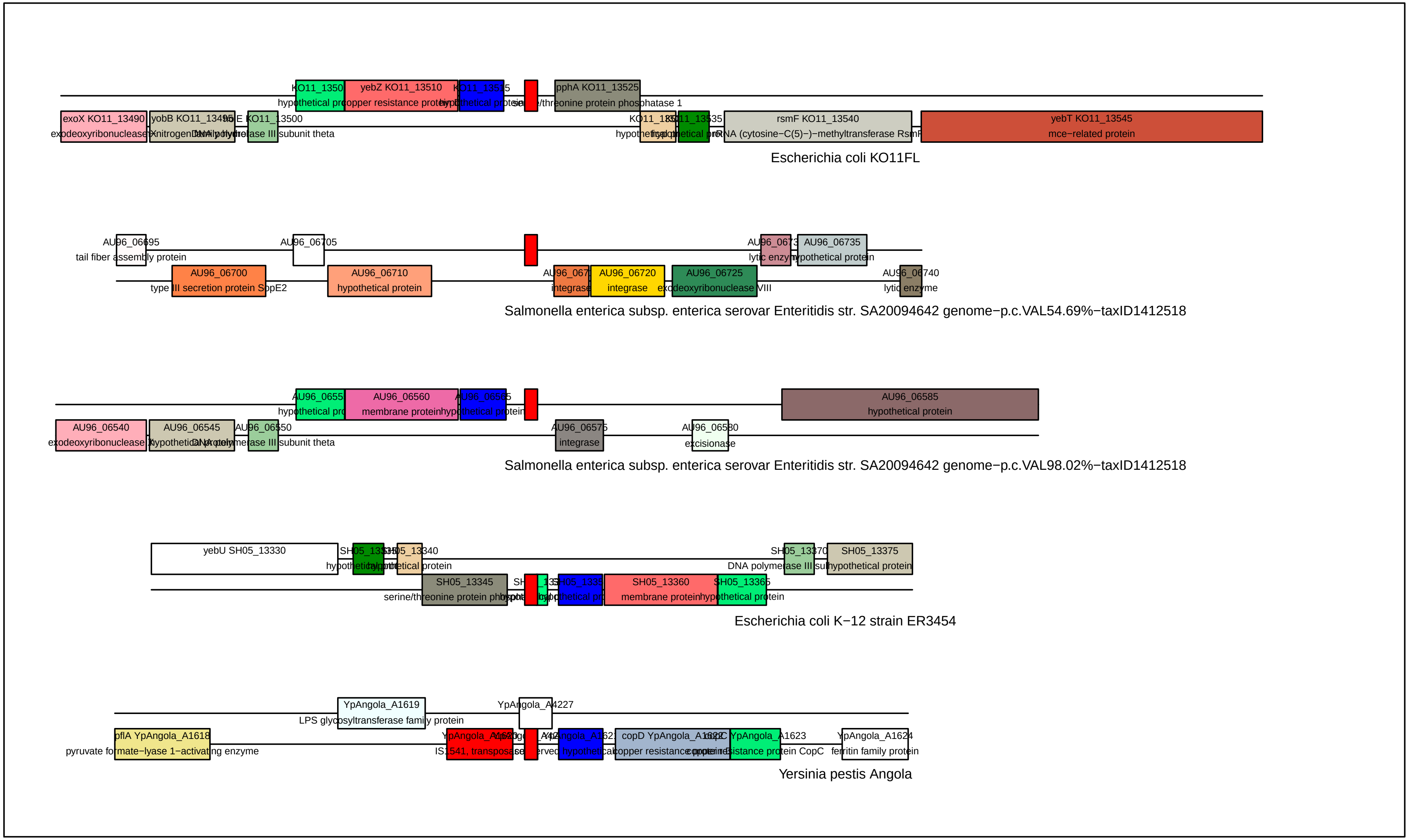

yebZ KO11_13510
pphA KO11_13525
exoX KO11_13490
exodeoxyribonuclease X
rsmF KO11_13540
yebT KO11_13545
mce−related protein
Escherichia coli KO11FL
AU96_06695
tail fiber assembly protein
AU96_06705
AU96_06735
hypothetical protein
AU96_06700
type III secretion protein SopE2
AU96_06710
hypothetical protein
AU96_06720
integrase
AU96_06725
exodeoxyribonuclease VIII
AU96_06740
lytic enzyme
Salmonella enterica subsp. enterica serovar Enteritidis str. SA20094642 genome−p.c.VAL54.69%−taxID1412518
AU96_06560
membrane protein
AU96_06585
hypothetical protein
AU96_06540
exodeoxyribonuclease X
AU96_06545
AU96_06550
DNA polymerase III subunit theta
AU96_06575
integrase
AU96_06580
excisionase
Salmonella enterica subsp. enterica serovar Enteritidis str. SA20094642 genome−p.c.VAL98.02%−taxID1412518
yebU SH05_13330
SH05_13340
hypothetical protein
SH05_13375
hypothetical protein
SH05_13345
serine/threonine protein phosphatase
SH05_13360
membrane protein
SH05_13365
hypothetical protein
Escherichia coli K−12 strain ER3454
YpAngola_A1619
LPS glycosyltransferase family protein
YpAngola_A4227
pflA YpAngola_A1618
pyruvate formate−lyase 1−activating enzyme
IS1541, transposase
copD YpAngola_A1622
YpAngola_A1623
copper resistance protein CopC
YpAngola_A1624
ferritin family protein
Yersinia pestis Angola

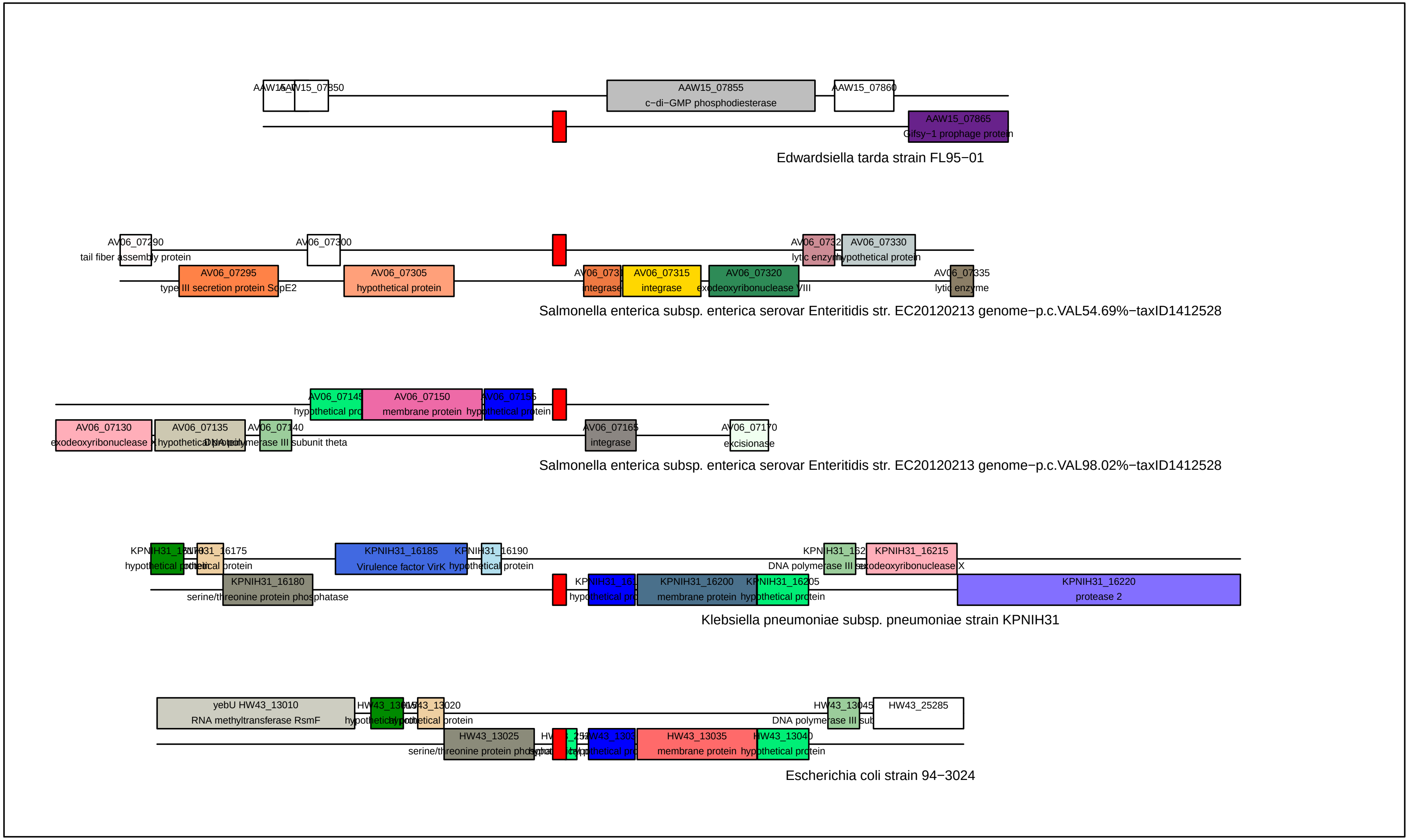
AAW15_07850
AAW15_07855
c−di−GMP phosphodiesterase
AAW15_07860
AAW15_07865
Gifsy−1 prophage protein
Edwardsiella tarda strain FL95−01
AV06_07290
tail fiber assembly protein
AV06_07300
AV06_07330
hypothetical protein
AV06_07295
type III secretion protein SopE2
AV06_07305
hypothetical protein
integrase
AV06_07315
integrase
AV06_07320
exodeoxyribonuclease VIII
AV06_07335
lytic enzyme
Salmonella enterica subsp. enterica serovar Enteritidis str. EC20120213 genome−p.c.VAL54.69%−taxID1412528
AV06_07150
membrane protein
AV06_07130
exodeoxyribonuclease
AV06_07135
AV06_07140
subunit theta
AV06_07165
integrase
AV06_07170
excisionase
Salmonella enterica subsp. enterica serovar Enteritidis str. EC20120213 genome−p.c.VAL98.02%−taxID1412528
KPNIH31_16175
KPNIH31_16185
Virulence factor VirK
KPNIH31_16190
hypothetical protein
KPNIH31_16215
exodeoxyribonuclease X
KPNIH31_16180
serine/threonine protein phosphatase
KPNIH31_16200
membrane protein
KPNIH31_16205
hypothetical protein
KPNIH31_16220
protease 2
Klebsiella pneumoniae subsp. pneumoniae strain KPNIH31
yebU HW43_13010
RNA methyltransferase RsmF
HW43_13020
HW43_13045
HW43_25285
HW43_13025
HW43_13035
membrane protein
HW43_13040
hypothetical protein
Escherichia coli strain 94−3024

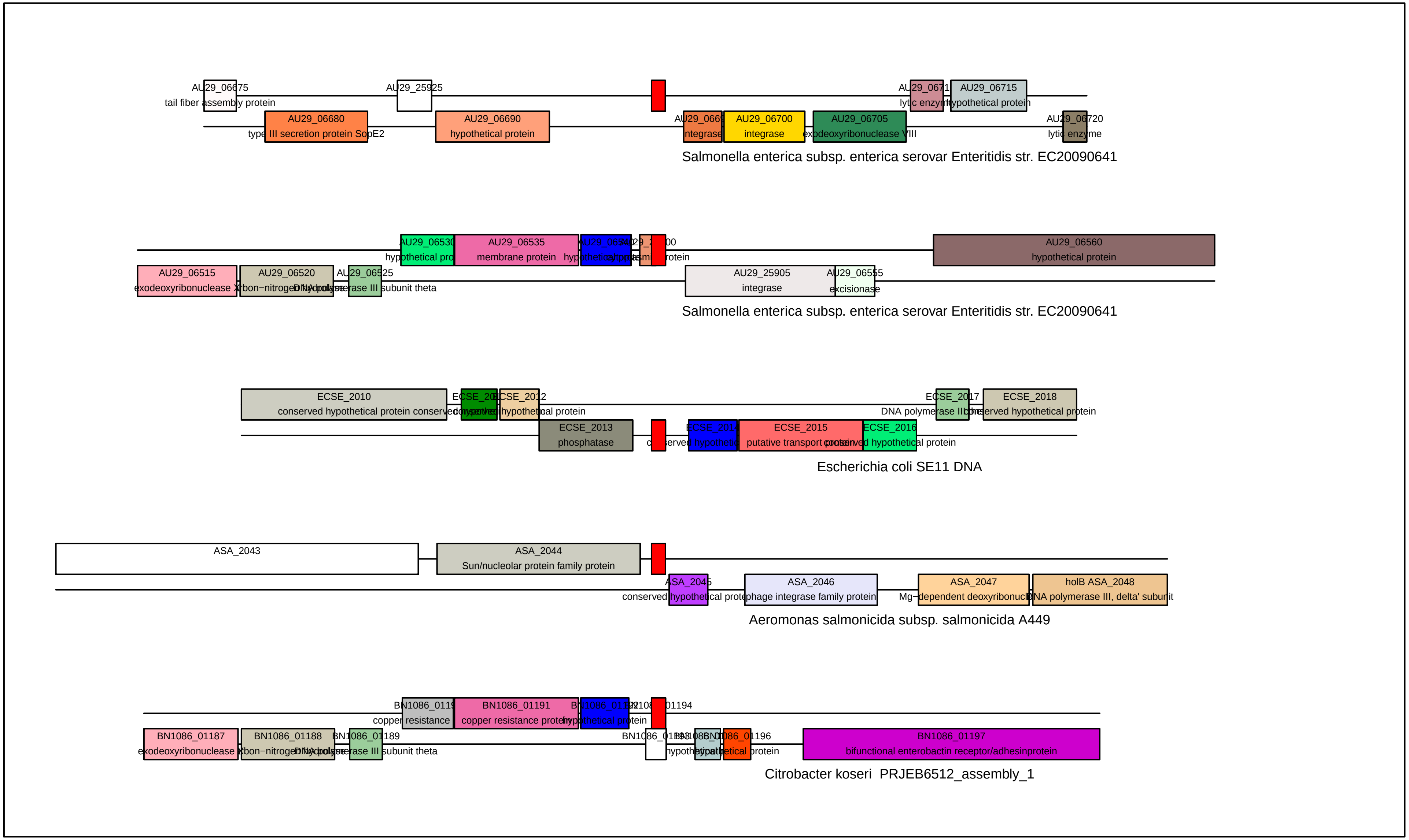

AU29_06675
tail fiber assembly protein
AU29_25925
AU29_06715
hypothetical protein
AU29_06680
type III secretion protein SopE2
AU29_06690
hypothetical protein
integrase
AU29_06700
integrase
AU29_06705
exodeoxyribonuclease VIII
AU29_06720
lytic enzyme
Salmonella enterica subsp. enterica serovar Enteritidis str. EC20090641
AU29_06535
membrane protein
AU29_06560
hypothetical protein
AU29_06515
exodeoxyribonuclease X
AU29_06520
AU29_06525
DNA polymerase III subunit theta
AU29_25905
integrase
AU29_06555
excisionase
Salmonella enterica subsp. enterica serovar Enteritidis str. EC20090641
ECSE_2010
conserved hypothetical protein
ECSE_2012
hypothetical protein
ECSE_2017
ECSE_2018
conserved hypothetical protein
ECSE_2013
phosphatase
ECSE_2015
putative transport protein
ECSE_2016
conserved hypothetical protein
Escherichia coli SE11 DNA
ASA_2043
ASA_2044
Sun/nucleolar protein family protein
ASA_2045
ASA_2046
phage integrase family protein
ASA_2047
holB ASA_2048
Aeromonas salmonicida subsp. salmonicida A449
BN1086_01191
copper resistance protein
hypothetical protein
BN1086_01187
exodeoxyribonuclease X
BN1086_01188
DNA polymerase III subunit theta
BN1086_01196
hypothetical protein
BN1086_01197
bifunctional enterobactin receptor/adhesinprotein
Citrobacter koseri PRJEB6512_assembly_1

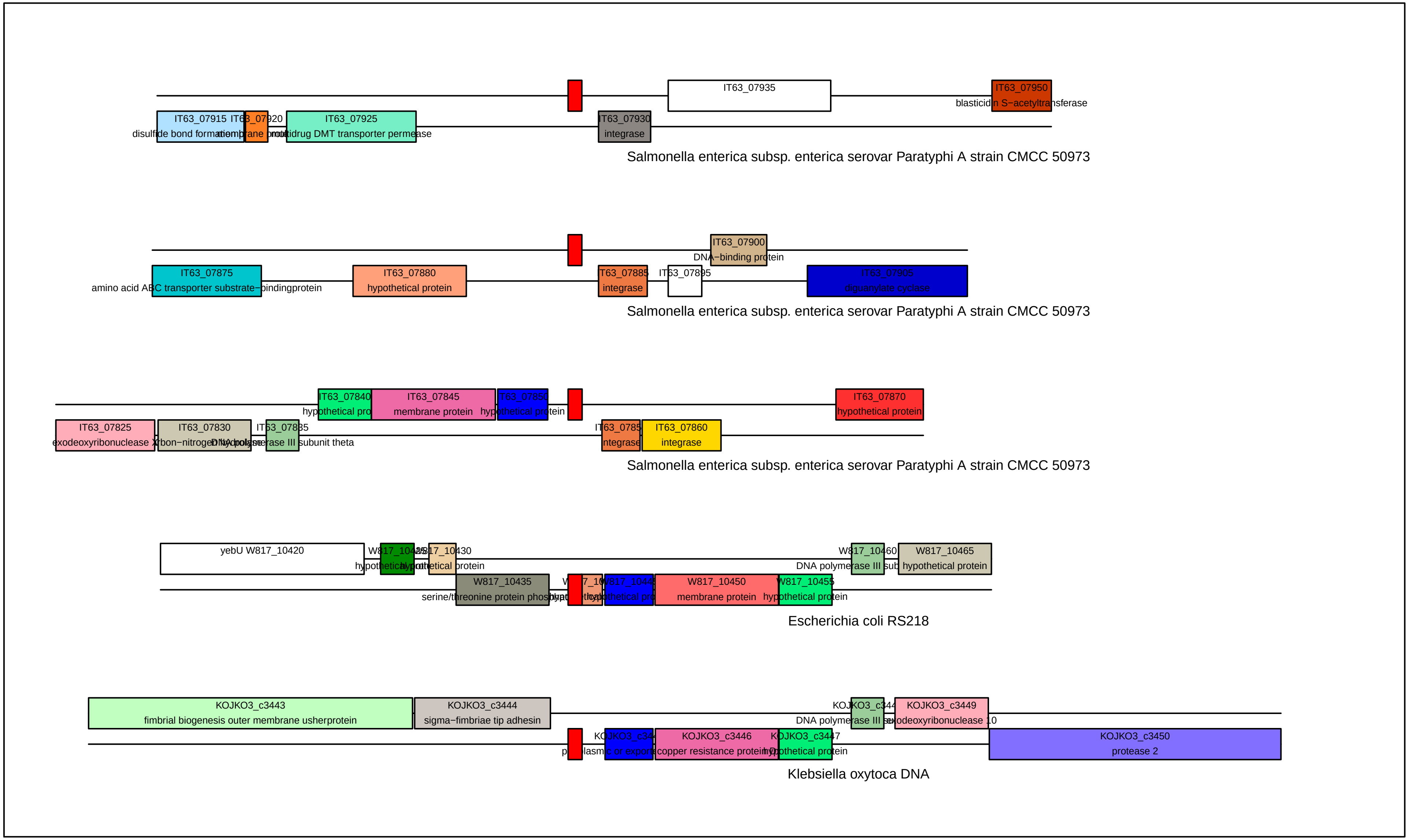
IT63_07935
IT63_07950
blasticidin S−acetyltransferase
IT63_07915
disulfide bond formation
IT63_07925
multidrug DMT transporter permease
IT63_07930
integrase
Salmonella enterica subsp. enterica serovar Paratyphi A strain CMCC 50973
IT63_07900
DNA−binding protein
IT63_07875
amino acid ABC transporter substrate−bindingprotein
IT63_07880
hypothetical protein
IT63_07885
integrase
IT63_07905
diguanylate cyclase
Salmonella enterica subsp. enterica serovar Paratyphi A strain CMCC 50973
IT63_07840
IT63_07845
membrane protein
IT63_07870
hypothetical protein
IT63_07825
exodeoxyribonuclease X
IT63_07830
IT63_07860
integrase
Salmonella enterica subsp. enterica serovar Paratyphi A strain CMCC 50973
yebU W817_10420
W817_10430
hypothetical protein
W817_10460
DNA polymerase III
W817_10465
hypothetical protein
W817_10435
W817_10450
membrane protein
W817_10455
hypothetical protein
Escherichia coli RS218
KOJKO3_c3443
fimbrial biogenesis outer membrane usherprotein
KOJKO3_c3444
sigma−fimbriae tip adhesin
KOJKO3_c3449
KOJKO3_c3446
copper resistance protein
KOJKO3_c3450
protease 2
Klebsiella oxytoca DNA

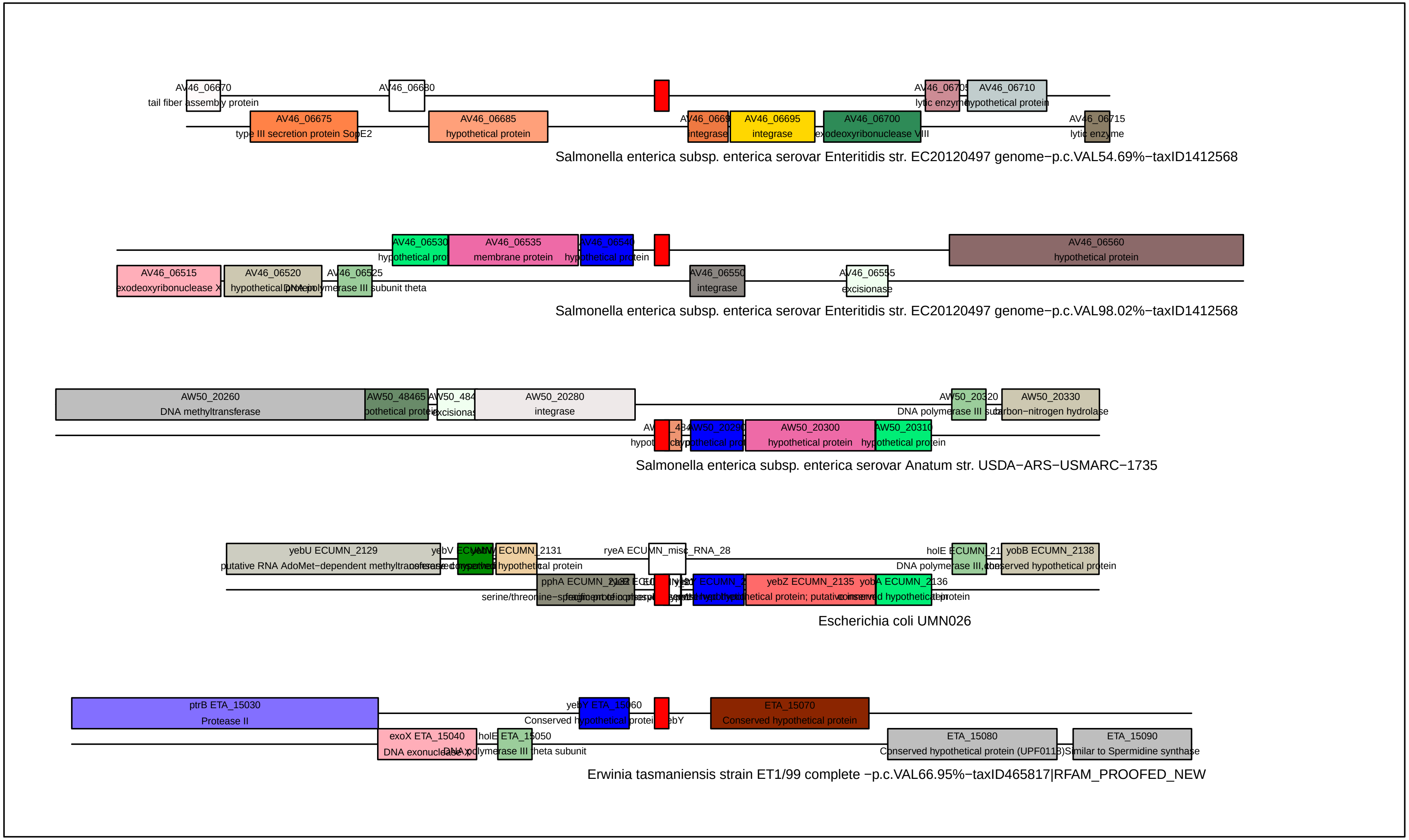

AV46_06670
tail fiber assembly protein
AV46_06680
AV46_06675
type III secretion protein SopE2
AV46_06685
hypothetical protein
integrase
AV46_06695
integrase
AV46_06700
exodeoxyribonuclease VIII
AV46_06710
hypothetical protein
AV46_06715
lytic enzyme
Salmonella enterica subsp. enterica serovar Enteritidis str. EC20120497 genome−p.c.VAL54.69%−taxID1412568
AV46_06530
AV46_06535
membrane protein
AV46_06560
hypothetical protein
AV46_06515
exodeoxyribonuclease X
AV46_06520
AV46_06550
integrase
excisionase
Salmonella enterica subsp. enterica serovar Enteritidis str. EC20120497 genome−p.c.VAL98.02%−taxID1412568
AW50_20260
DNA methyltransferase
AW50_48465
AW50_20280
integrase
AW50_20330
carbon−nitrogen hydrolase
AW50_20300
hypothetical protein
AW50_20310
Salmonella enterica subsp. enterica serovar Anatum str. USDA−ARS−USMARC−1735
yebU ECUMN_2129
putative RNA AdoMet−dependent methyltransferase
ryeA ECUMN_misc_RNA_28
yobB ECUMN_2138
pphA ECUMN_2132
yebZ ECUMN_2135
Escherichia coli UMN026
ptrB ETA_15030
Protease II
exoX ETA_15040
ETA_15070
Conserved hypothetical protein
ETA_15080
ETA_15090
Erwinia tasmaniensis strain ET1/99 complete −p.c.VAL66.95%−taxID465817|RFAM_PROOFED_NEW

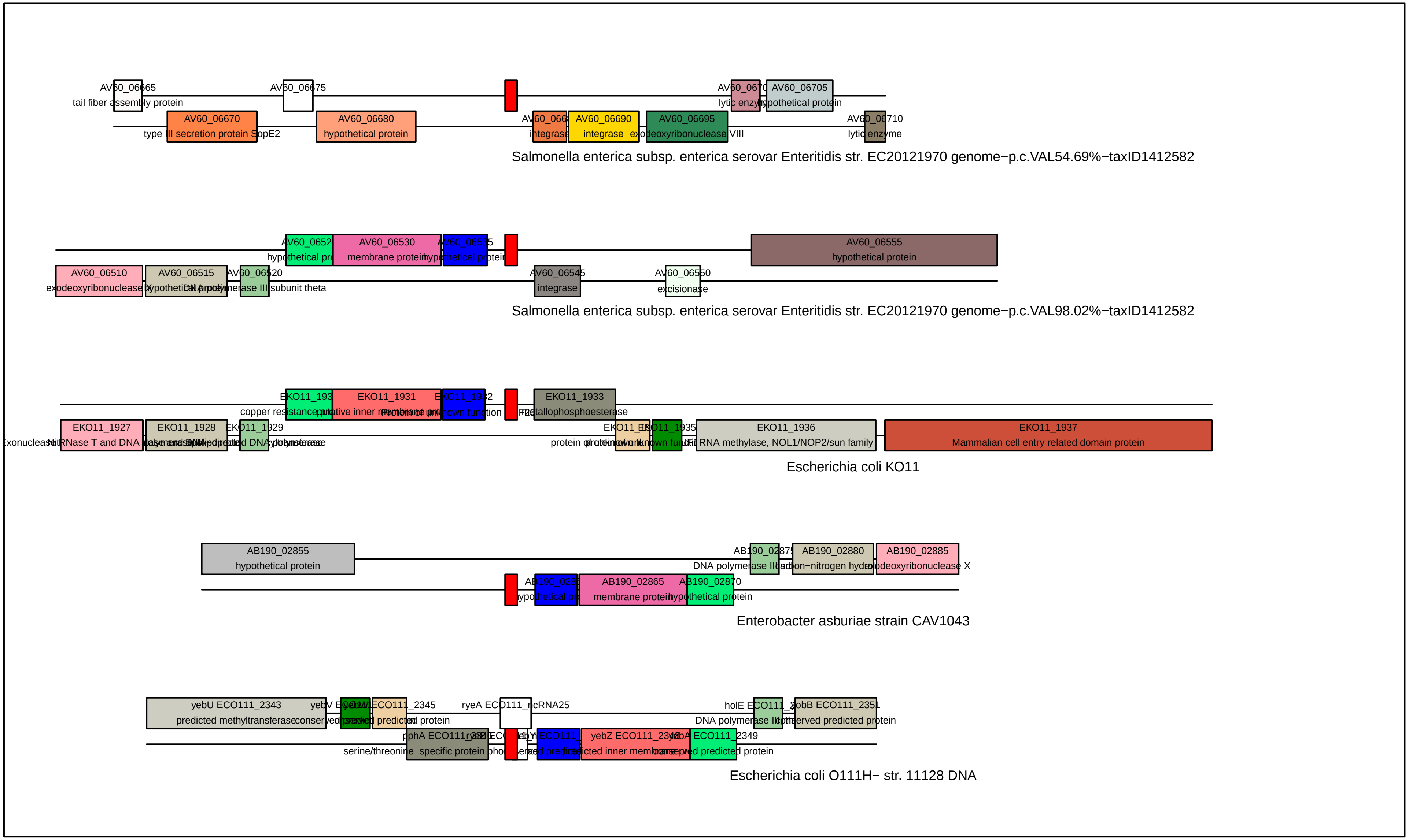
AV60_06665
tail fiber assembly protein
AV60_06675
AV60_06705
hypothetical protein
AV60_06670
type III secretion protein SopE2
AV60_06680
hypothetical protein
AV60_06690
integrase
AV60_06695
exodeoxyribonuclease VIII
AV60_06710
lytic enzyme
Salmonella enterica subsp. enterica serovar Enteritidis str. EC20121970 genome−p.c.VAL54.69%−taxID1412582
AV60_06530
membrane protein
AV60_06555
hypothetical protein
AV60_06510
exodeoxyribonuclease X
AV60_06515
AV60_06520
AV60_06545
integrase
AV60_06550
excisionase
Salmonella enterica subsp. enterica serovar Enteritidis str. EC20121970 genome−p.c.VAL98.02%−taxID1412582
EKO11_1931
EKO11_1933
metallophosphoesterase
EKO11_1927
EKO11_1928
EKO11_1929
EKO11_1936
RNA methylase, NOL1/NOP2/sun family
EKO11_1937
Mammalian cell entry related domain protein
Escherichia coli KO11
AB190_02855
hypothetical protein
AB190_02880
AB190_02885
AB190_02865
membrane protein
AB190_02870
hypothetical protein
Enterobacter asburiae strain CAV1043
yebU ECO111_2343
predicted methyltransferase
ryeA ECO111_ncRNA25
yobB ECO111_2351
conserved predicted protein
yebZ ECO111_2348
ECO111_2349
Escherichia coli O111H− str. 11128 DNA

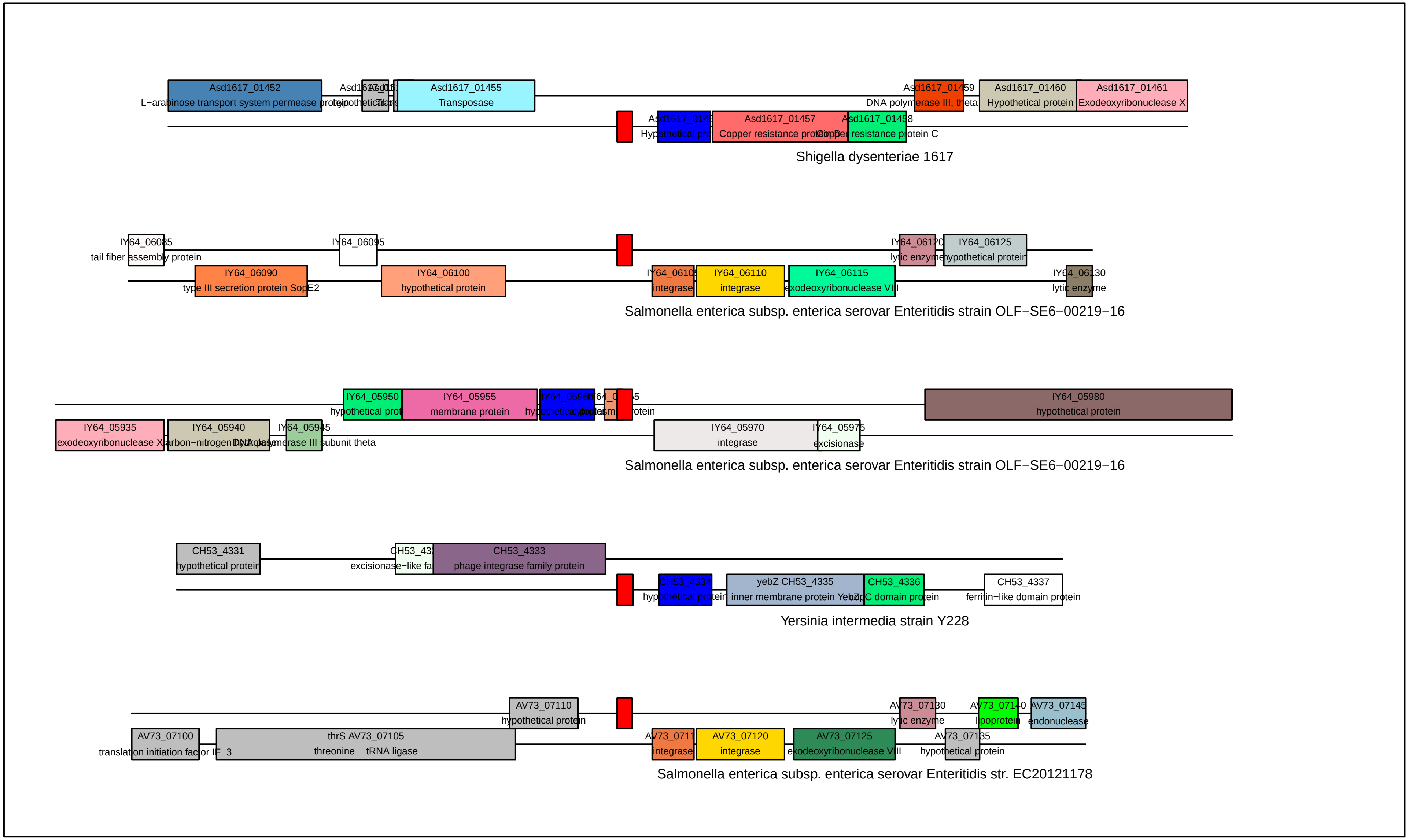

Asd1617_01452
L−arabinose transport system permease protein
Asd1617_01455
Transposase
Asd1617_01459
DNA polymerase III, theta
Asd1617_01460
Hypothetical protein
Asd1617_01461
Exodeoxyribonuclease X
Asd1617_01457
Copper resistance protein
Asd1617_01458
resistance protein C
Shigella dysenteriae 1617
IY64_06085
tail fiber assembly protein
IY64_06095
IY64_06120
lytic enzyme
IY64_06125
hypothetical protein
IY64_06090
type III secretion protein SopE2
IY64_06100
hypothetical protein
IY64_06110
integrase
IY64_06115
exodeoxyribonuclease VIII
IY64_06130
lytic enzyme
Salmonella enterica subsp. enterica serovar Enteritidis strain OLF−SE6−00219−16
IY64_05950
IY64_05955
membrane protein
IY64_05980
hypothetical protein
IY64_05935
exodeoxyribonuclease X
IY64_05940
IY64_05945
IY64_05970
integrase
IY64_05975
excisionase
Salmonella enterica subsp. enterica serovar Enteritidis strain OLF−SE6−00219−16
CH53_4331
hypothetical protein
CH53_4333
phage integrase family protein
yebZ CH53_4335
inner membrane protein YebZ
CH53_4336
CH53_4337
ferritin−like domain protein
Yersinia intermedia strain Y228
AV73_07110
hypothetical protein
AV73_07130
lytic enzyme
AV73_07140
lipoprotein
AV73_07145
endonuclease
AV73_07100
translation initiation factor IF−3
thrS AV73_07105
threonine−−tRNA ligase
integrase
AV73_07120
integrase
AV73_07125
exodeoxyribonuclease VIII
AV73_07135
hypothetical protein
Salmonella enterica subsp. enterica serovar Enteritidis str. EC20121178

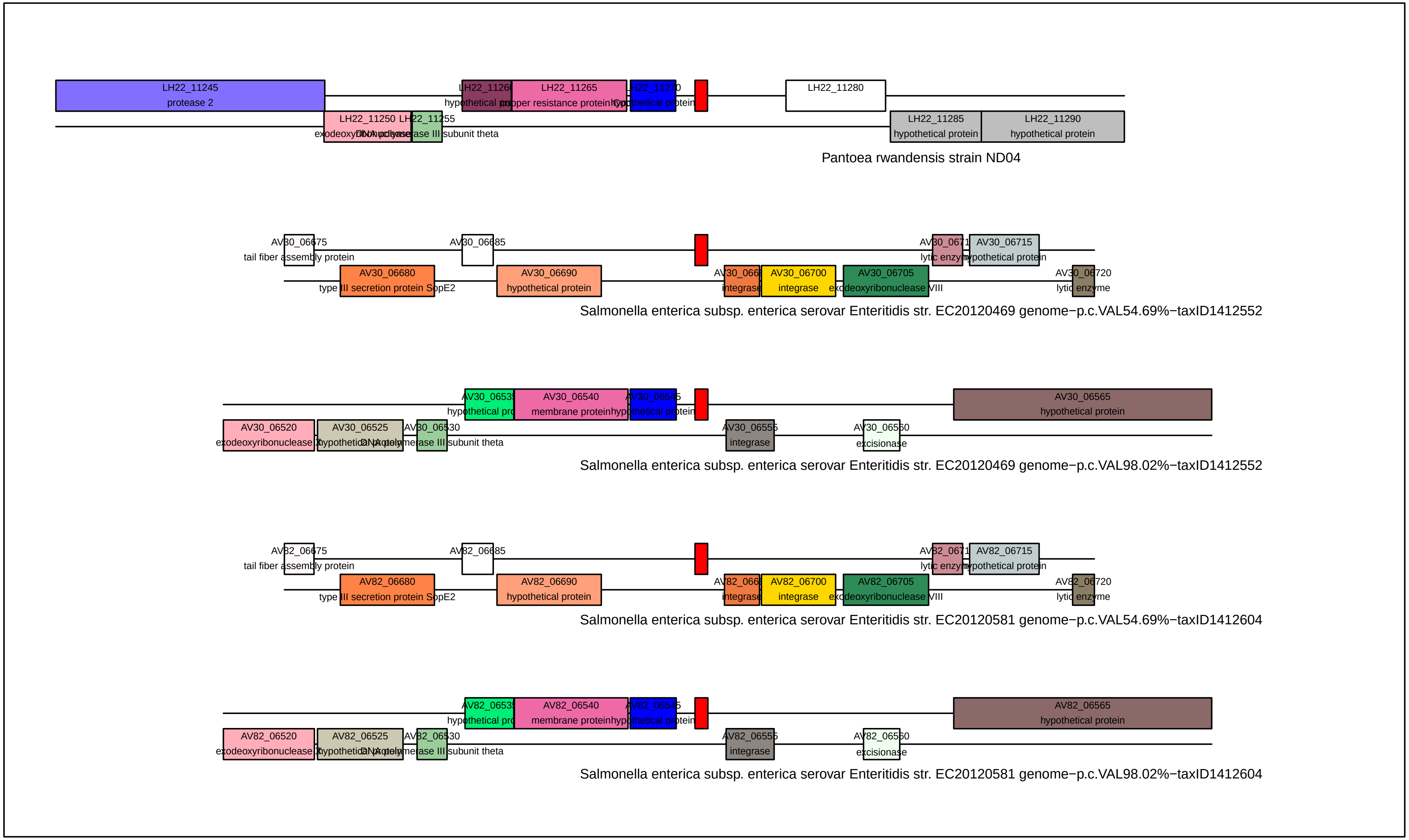
LH22_11245
protease 2
LH22_11265
LH22_11280
LH22_11250
LH22_11285
hypothetical protein
LH22_11290
hypothetical protein
Pantoea rwandensis strain ND04
AV30_06675
tail fiber assembly protein
AV30_06685
AV30_06715
AV30_06680
type III secretion protein SopE2
AV30_06690
hypothetical protein
AV30_06700
integrase
AV30_06705
exodeoxyribonuclease VIII
AV30_06720
lytic enzyme
Salmonella enterica subsp. enterica serovar Enteritidis str. EC20120469 genome−p.c.VAL54.69%−taxID1412552
AV30_06540
membrane protein
AV30_06565
hypothetical protein
AV30_06520
exodeoxyribonuclease X
AV30_06525
AV30_06555
integrase
AV30_06560
excisionase
Salmonella enterica subsp. enterica serovar Enteritidis str. EC20120469 genome−p.c.VAL98.02%−taxID1412552
AV82_06675
tail fiber assembly protein
AV82_06685
AV82_06715
AV82_06680
type III secretion protein SopE2
AV82_06690
hypothetical protein
AV82_06700
integrase
AV82_06705
exodeoxyribonuclease VIII
AV82_06720
lytic enzyme
Salmonella enterica subsp. enterica serovar Enteritidis str. EC20120581 genome−p.c.VAL54.69%−taxID1412604
AV82_06540
membrane protein
AV82_06565
hypothetical protein
AV82_06520
exodeoxyribonuclease X
AV82_06525
AV82_06555
integrase
AV82_06560
excisionase
Salmonella enterica subsp. enterica serovar Enteritidis str. EC20120581 genome−p.c.VAL98.02%−taxID1412604

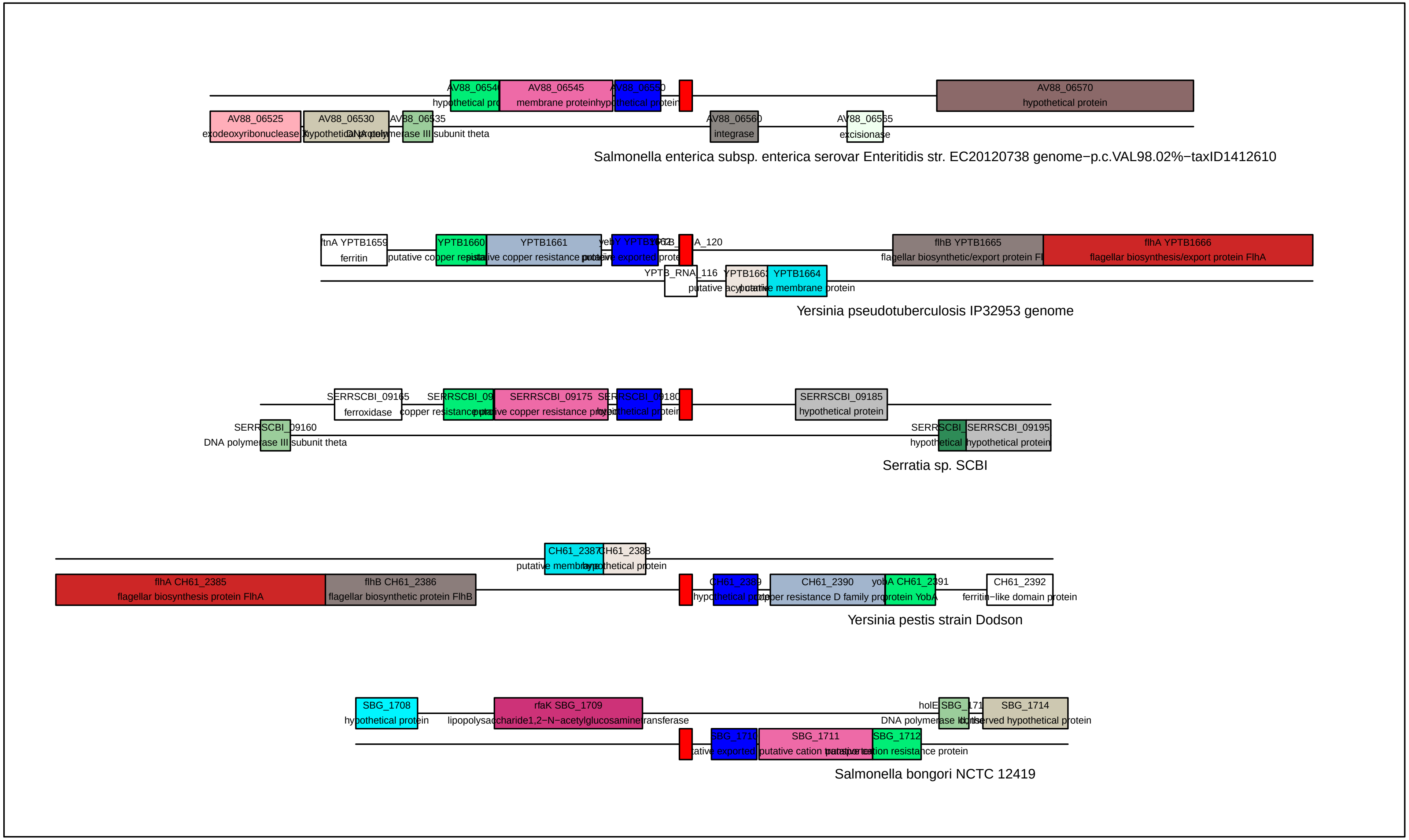
Salmonella enterica subsp. enterica serovar Enteritidis str. EC20120738 genome−p.c.VAL98.02%−taxID1412610
AV88_06545
membrane protein
AV88_06570
hypothetical protein
AV88_06525
AV88_06530
AV88_06560
integrase
subunit theta
Yersinia pseudotuberculosis IP32953 genome
YPTB1660
YPTB1661
YPTB1664
ferritin
flhB YPTB1665
flhA YPTB1666
flagellar biosynthesis/export protein FlhA
Serratia sp. SCBI
SERRSCBI_09175
SERRSCBI_09185
hypothetical protein
ferroxidase
SERRSCBI_09195
Yersinia pestis strain Dodson
flhA CH61_2385
flagellar biosynthesis protein FlhA
flhB CH61_2386
flagellar biosynthetic protein FlhB
CH61_2390
CH61_2392
Salmonella bongori NCTC 12419
SBG_1708
hypothetical protein
rfaK SBG_1709
SBG_1711
SBG_1714

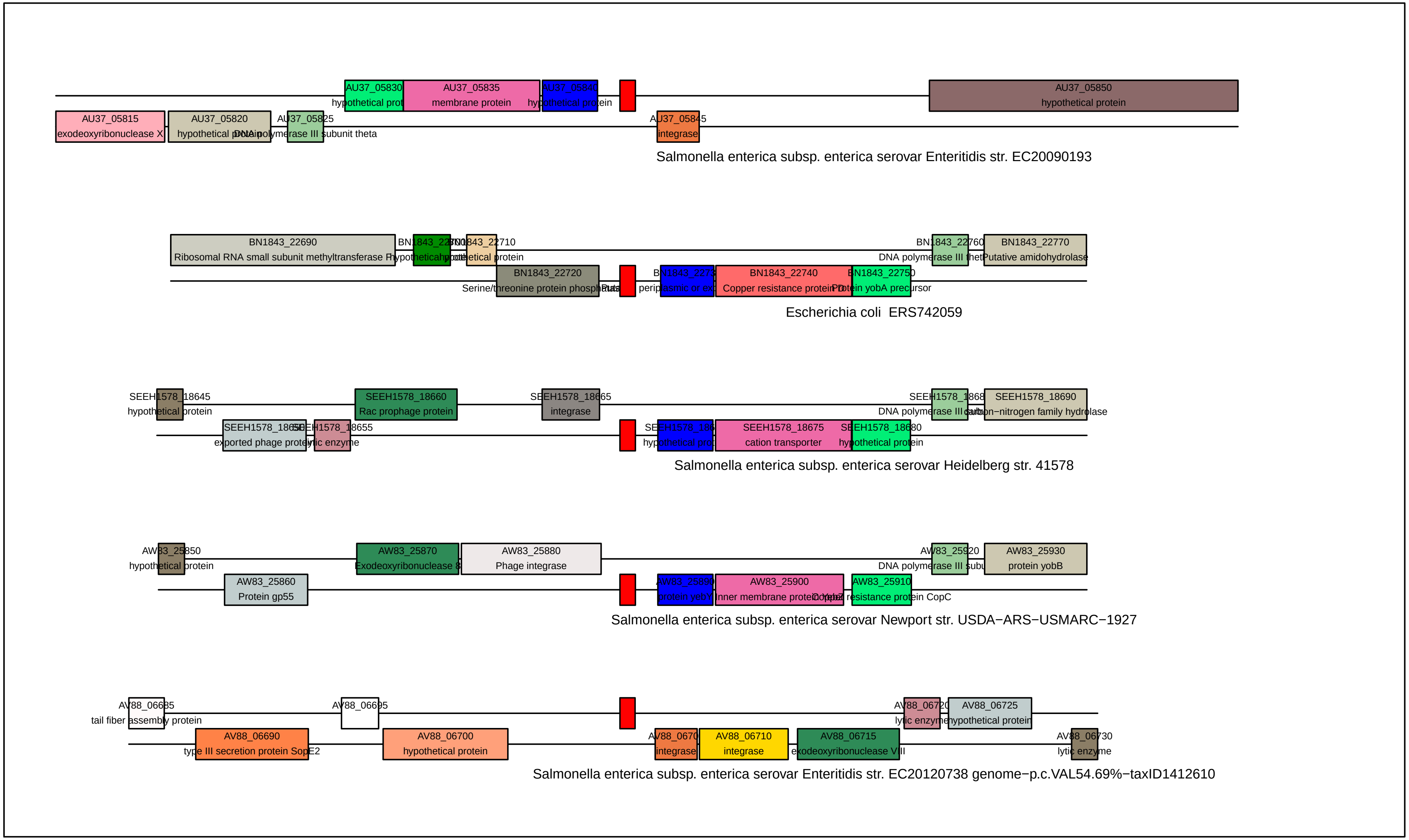

AU37_05830
hypothetical prot
AU37_05835
membrane protein
AU37_05840
hypothetical protein
AU37_05850
hypothetical protein
AU37_05815
exodeoxyribonuclease X
AU37_05820
hypothetical protein
AU37_05825
DNA polymerase III subunit theta
AU37_05845
integrase
Salmonella enterica subsp. enterica serovar Enteritidis str. EC20090193
BN1843_22690
Ribosomal RNA small subunit methyltransferase F
BN1843_22710
hypothetical protein
BN1843_22760
DNA polymerase III the
BN1843_22770
Putative amidohydrolase
BN1843_22720
Serine/threonine protein phosphatase
BN1843_22740
Copper resistance protein D
BN1843_22750
Protein yobA precursor
Escherichia coli ERS742059
SEEH1578_18645
hypothetical protein
SEEH1578_18660
Rac prophage protein
SEEH1578_18665
integrase
SEEH1578_18690
carbon−nitrogen family hydrolase
DNA polymerase III
SEEH1578_18655
exported phage prote
lytic enzyme
SEEH1578_18675
cation transporter
SEEH1578_18680
hypothetical protein
Salmonella enterica subsp. enterica serovar Heidelberg str. 41578
AW83_25850
hypothetical protein
AW83_25870
Exodeoxyribonuclease 8
AW83_25880
Phage integrase
AW83_25920
DNA polymerase III subu
AW83_25930
protein yobB
AW83_25860
Protein gp55
AW83_25890
protein yobY
AW83_25900
Inner membrane protein
AW83_25910
Copper resistance protein CopC
Salmonella enterica subsp. enterica serovar Newport str. USDA−ARS−USMARC−1927
AV88_06685
tail fiber assembly protein
AV88_06695
AV88_06720
lytic enzyme
AV88_06725
hypothetical protein
AV88_06690
type III secretion protein SopE2
AV88_06700
hypothetical protein
integrase
AV88_06710
integrase
AV88_06715
exodeoxyribonuclease VIII
AV88_06730
lytic enzyme
Salmonella enterica subsp. enterica serovar Enteritidis str. EC20120738 genome−p.c.VAL54.69%−taxID1412610

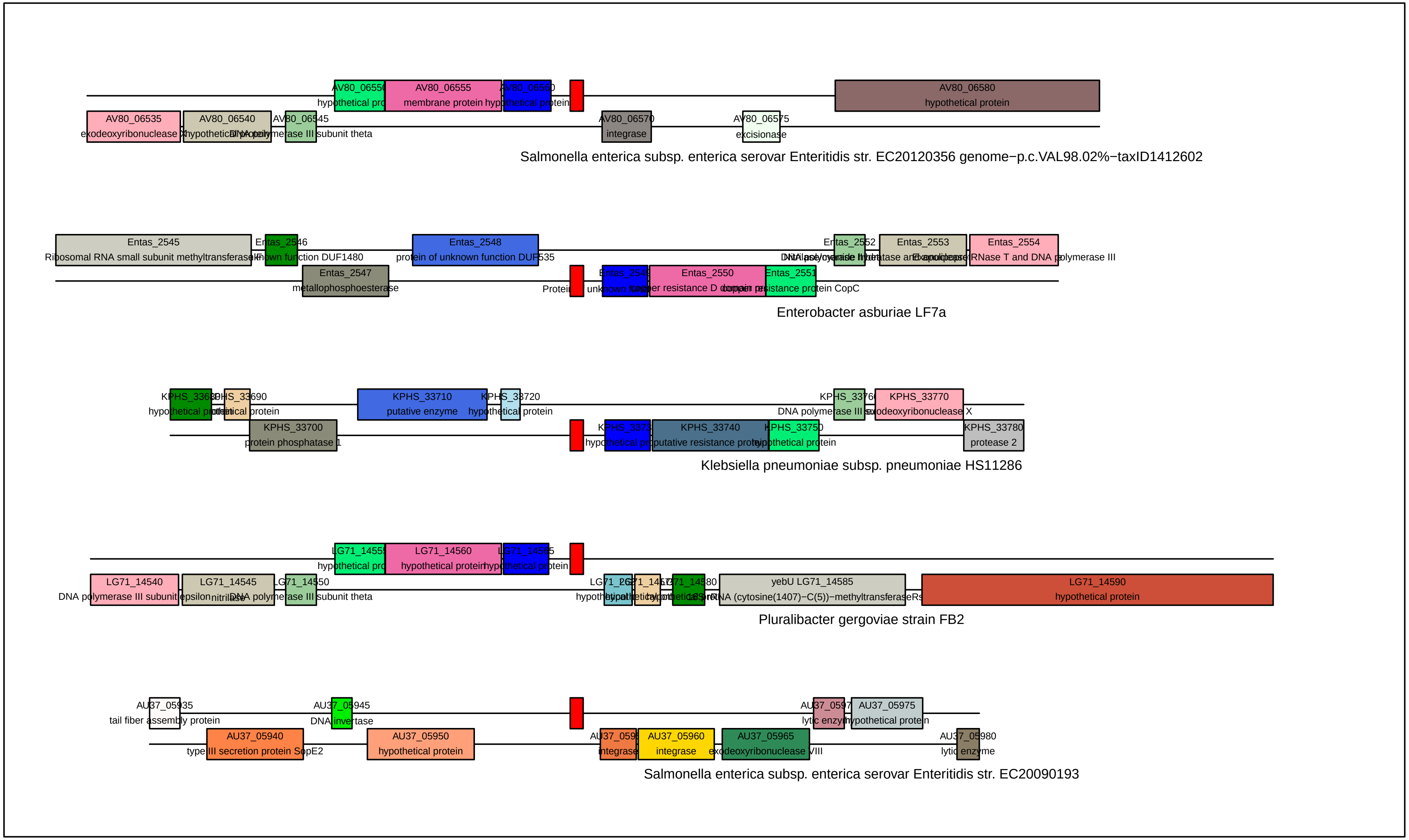

AV80_06555
membrane protein
AV80_06580
hypothetical protein
AV80_06535
exodeoxyribonuclease X
AV80_06540
AV80_06545
subunit theta
AV80_06570
integrase
AV80_06575
excisionase
Salmonella enterica subsp. enterica serovar Enteritidis str. EC20120356 genome−p.c.VAL98.02%−taxID1412602
Entas_2545
Ribosomal RNA small subunit methyltransferase
Entas_2546
Entas_2548
protein of unknown function DUF535
Entas_2552
Entas_2553
Entas_2554
RNase T and DNA polymerase III
Entas_2547
metallophosphoesterase
Entas_2550
Entas_2551
Enterobacter asburiae LF7a
KPHS_33690
KPHS_33710
putative enzyme
KPHS_33720
hypothetical protein
KPHS_33770
exodeoxyribonuclease X
KPHS_33700
protein phosphatase 1
KPHS_33740
KPHS_33750
KPHS_33780
protease 2
Klebsiella pneumoniae subsp. pneumoniae HS11286
LG71_14560
hypothetical protein
LG71_14565
LG71_14540
DNA polymerase III subunit epsilon
LG71_14545
LG71_14550
subunit theta
LG71_14580
yebU LG71_14585
rRNA (cytosine(1407)−C(5))−methyltransferase
LG71_14590
hypothetical protein
Pluralibacter gergoviae strain FB2
AU37_05935
tail fiber assembly protein
AU37_05945
DNA invertase
AU37_05975
hypothetical protein
AU37_05940
type III secretion protein SopE2
AU37_05950
hypothetical protein
AU37_05960
integrase
AU37_05965
exodeoxyribonuclease VIII
AU37_05980
lytic enzyme
Salmonella enterica subsp. enterica serovar Enteritidis str. EC20090193

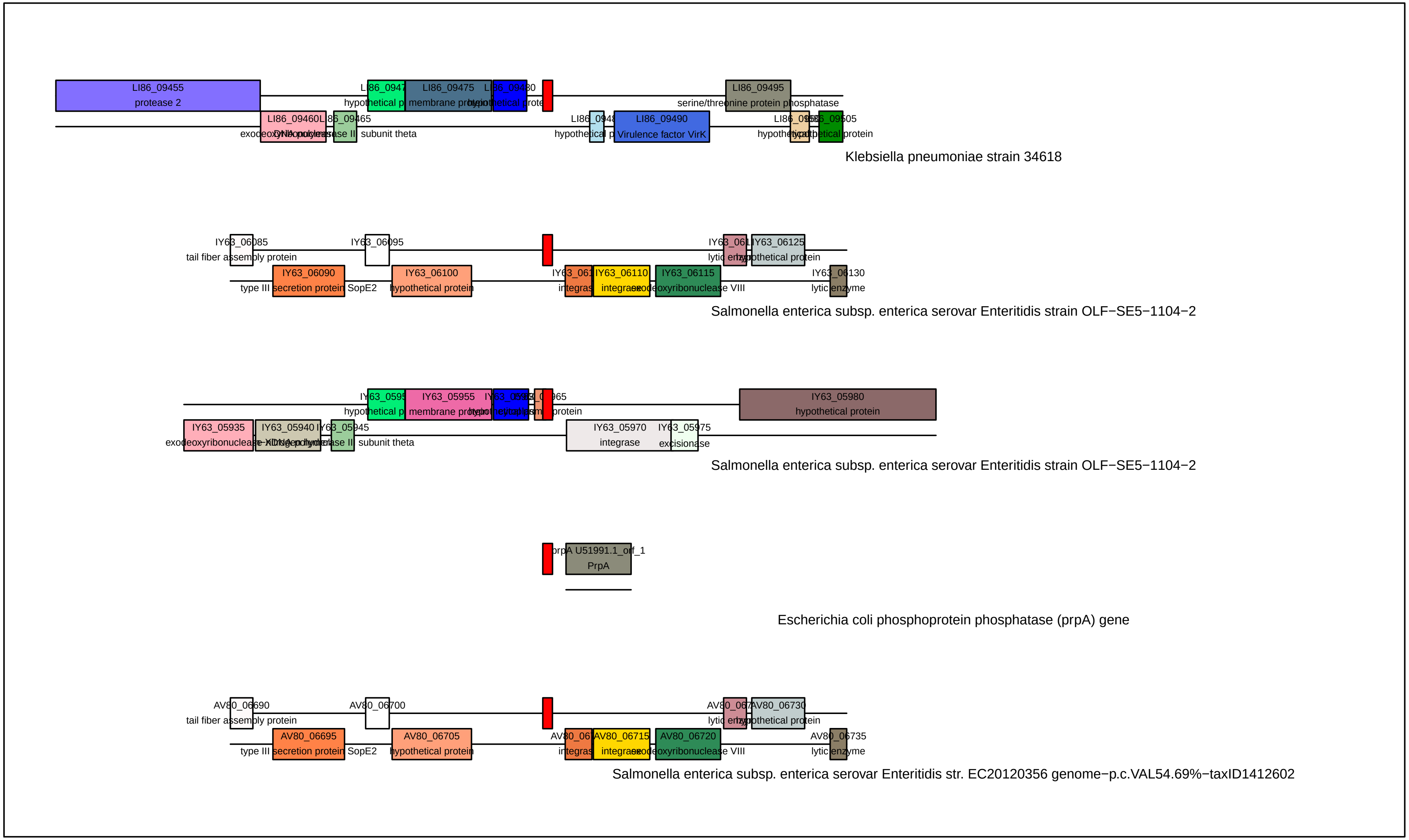

LI86_09455
protease 2
LI86_09475
LI86_09480
LI86_09495
serine/threonine protein phosphatase
LI86_09465
subunit theta
LI86_09490
Virulence factor VirK
LI86_09505
Klebsiella pneumoniae strain 34618
IY63_06085
tail fiber assembly protein
IY63_06095
IY63_06125
hypothetical protein
IY63_06090
type III secretion protein SopE2
IY63_06100
hypothetical protein
IY63_06110
integrase
IY63_06115
exodeoxyribonuclease VIII
IY63_06130
lytic enzyme
Salmonella enterica subsp. enterica serovar Enteritidis strain OLF−SE5−1104−2
IY63_05955
membrane protein
IY63_05980
hypothetical protein
IY63_05935
IY63_05940
IY63_05945
subunit theta
IY63_05970
integrase
IY63_05975
excisionase
Salmonella enterica subsp. enterica serovar Enteritidis strain OLF−SE5−1104−2
prpA U51991.1_orf_1
PrpA
Escherichia coli phosphoprotein phosphatase (prpA) gene
AV80_06690
tail fiber assembly protein
AV80_06700
AV80_06730
hypothetical protein
AV80_06695
type III secretion protein SopE2
AV80_06705
hypothetical protein
AV80_06715
integrase
AV80_06720
exodeoxyribonuclease VIII
AV80_06735
lytic enzyme
Salmonella enterica subsp. enterica serovar Enteritidis str. EC20120356 genome−p.c.VAL54.69%−taxID1412602

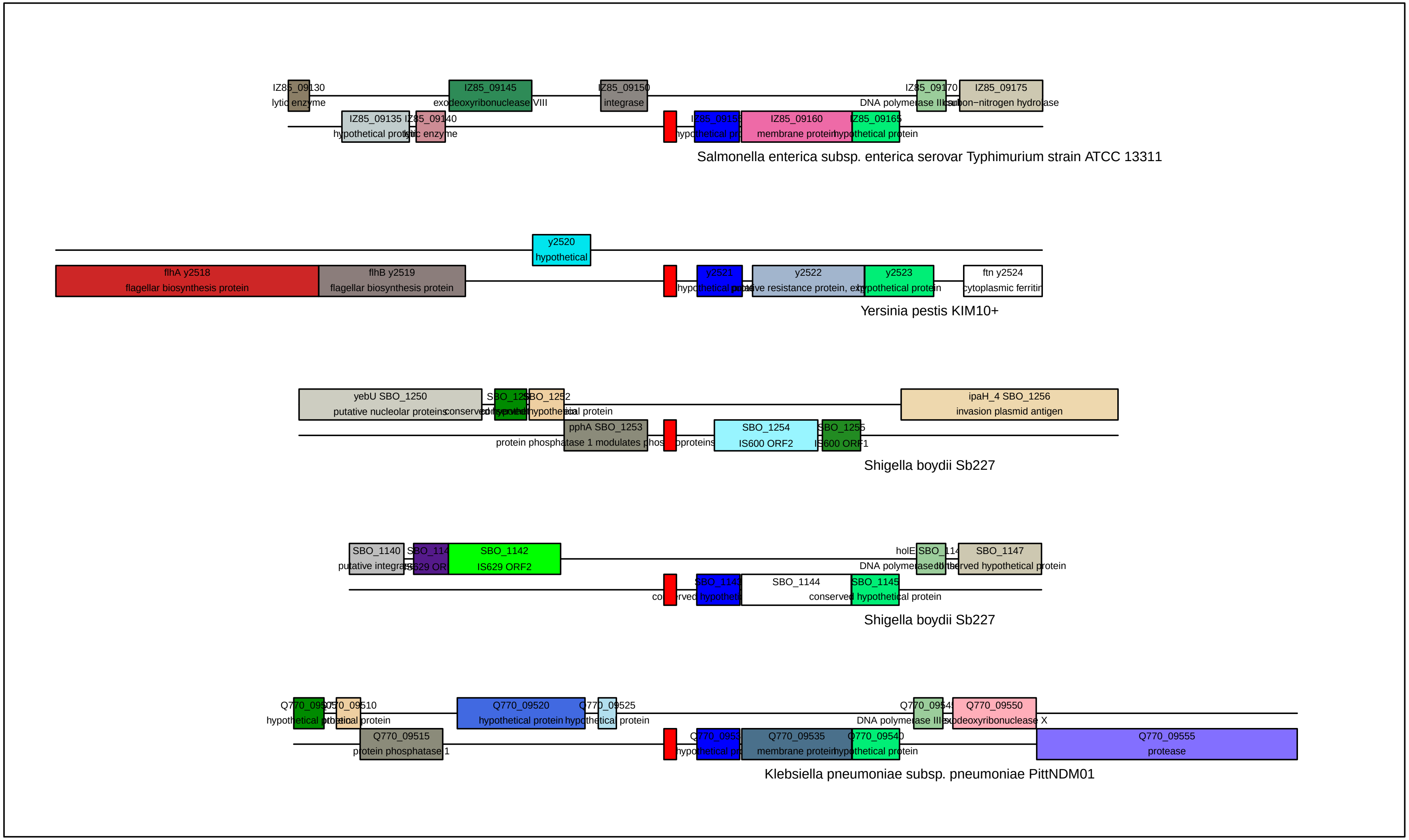

lytic enzyme
IZ85_09145
IZ85_09150
integrase
IZ85_09175
IZ85_09135
IZ85_09160
membrane protein
Salmonella enterica subsp. enterica serovar Typhimurium strain ATCC 13311
y2520
hypothetical
flhA y2518
flagellar biosynthesis protein
flhB y2519
flagellar biosynthesis protein
y2521
y2522
y2523
ftn y2524
Yersinia pestis KIM10+
yebU SBO_1250
putative nucleolar proteins
ipaH_4 SBO_1256
invasion plasmid antigen
pphA SBO_1253
SBO_1254
IS600 ORF2
Shigella boydii Sb227
SBO_1140
SBO_1142
IS629 ORF2
SBO_1147
SBO_1144
Shigella boydii Sb227
Q770_09520
hypothetical protein
Q770_09550
Q770_09515
Q770_09535
membrane protein
Q770_09555
protease
Klebsiella pneumoniae subsp. pneumoniae PittNDM01

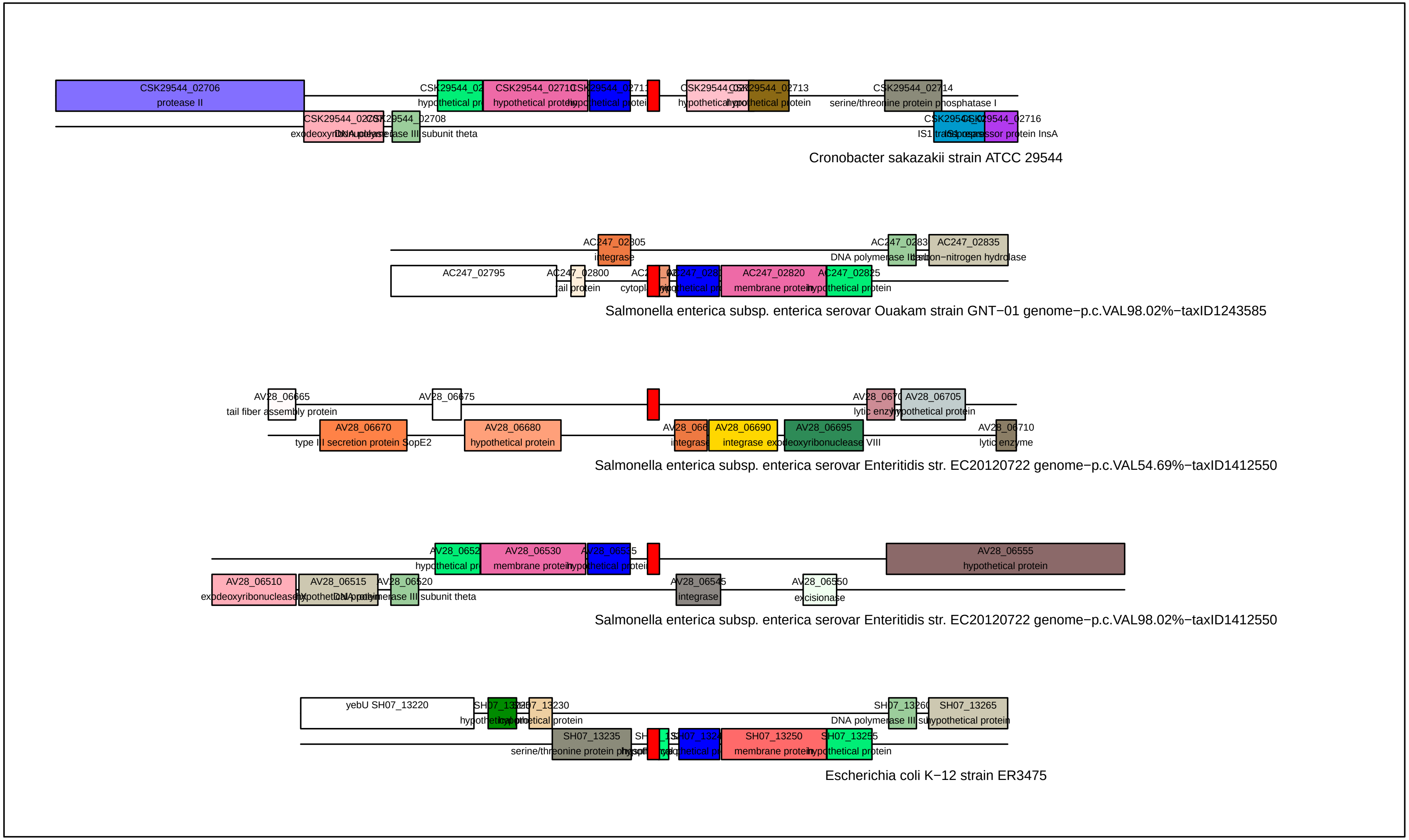

CSK29544_02706
protease II
DNA polymerase III subunit theta
serine/threonine protein phosphatase I
IS1 repressor protein InsA
Cronobacter sakazakii strain ATCC 29544
AC247_02795
AC247_02805
integrase
AC247_02820
membrane protein
AC247_02835
carbon−nitrogen hydrolase
Salmonella enterica subsp. enterica serovar Ouakam strain GNT−01 genome−p.c.VAL98.02%−taxID1243585
AV28_06665
tail fiber assembly protein
AV28_06670
type III secretion protein SopE2
AV28_06675
AV28_06680
hypothetical protein
AV28_06690
integrase
AV28_06695
exodeoxyribonuclease VIII
AV28_06705
hypothetical protein
AV28_06710
lytic enzyme
Salmonella enterica subsp. enterica serovar Enteritidis str. EC20120722 genome−p.c.VAL54.69%−taxID1412550
AV28_06510
AV28_06515
AV28_06520
AV28_06530
membrane protein
AV28_06545
integrase
AV28_06550
excisionase
AV28_06555
hypothetical protein
Salmonella enterica subsp. enterica serovar Enteritidis str. EC20120722 genome−p.c.VAL98.02%−taxID1412550
yebU SH07_13220
SH07_13230
SH07_13235
SH07_13250
membrane protein
SH07_13255
hypothetical protein
SH07_13265
hypothetical protein
DNA polymerase III
Escherichia coli K−12 strain ER3475

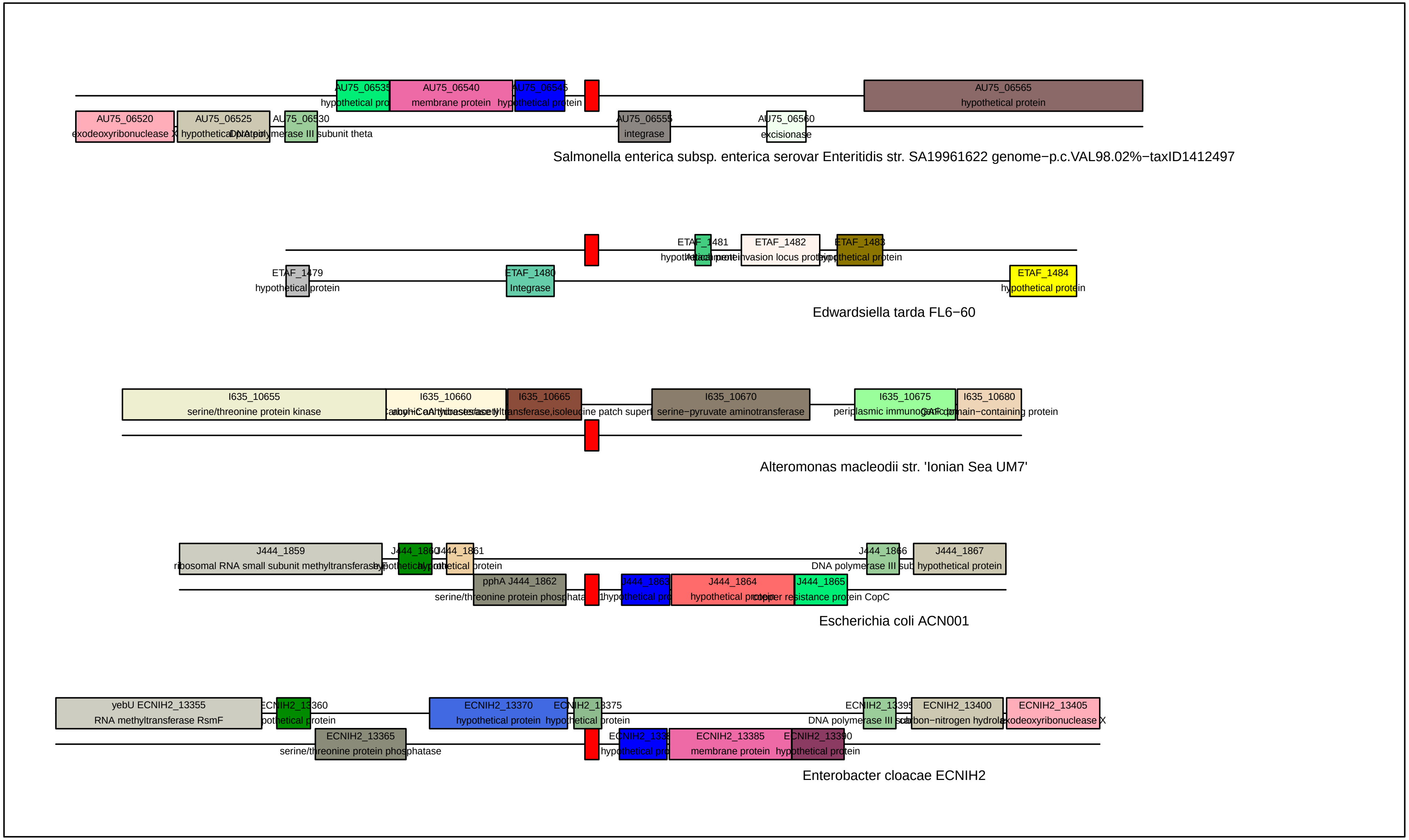
Salmonella enterica subsp. enterica serovar Enteritidis str. SA19961622 genome−p.c.VAL98.02%−taxID1412497
Edwardsiella tarda FL6−60
Alteromonas macleodii str. 'Ionian Sea UM7'
Escherichia coli ACN001
Enterobacter cloacae ECNIH2

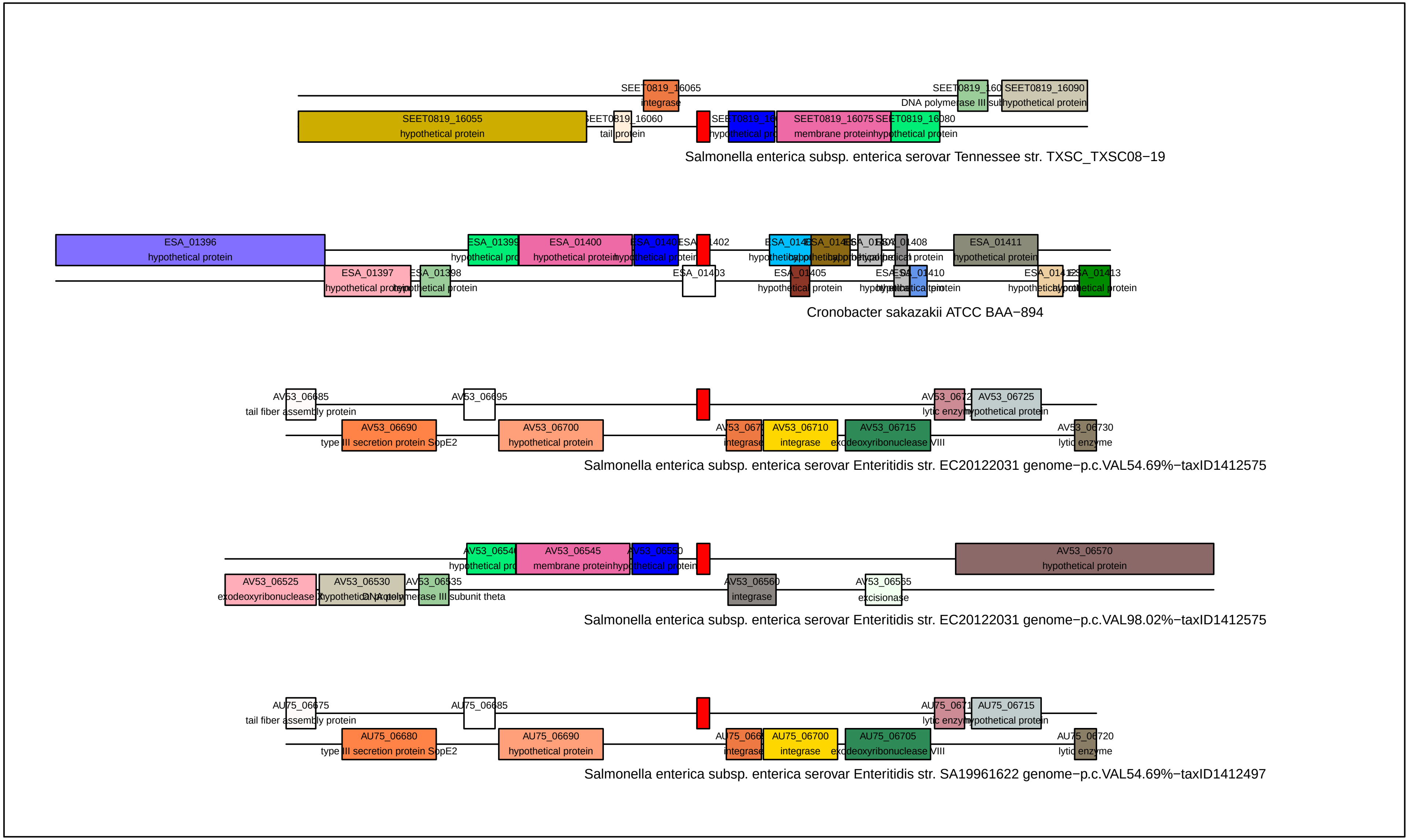

SEET0819_16065
integrase
SEET0819_16090
hypothetical protein
SEET0819_16055
hypothetical protein
tail protein
SEET0819_16075
membrane protein
SEET0819_16080
Salmonella enterica subsp. enterica serovar Tennessee str. TXSC_TXSC08−19
ESA_01396
hypothetical protein
ESA_01400
ESA_01411
ESA_01397
ESA_01403
ESA_01405
Cronobacter sakazakii ATCC BAA−894
AV53_06685
tail fiber assembly protein
AV53_06695
AV53_06725
AV53_06690
type III secretion protein SopE2
AV53_06700
hypothetical protein
AV53_06710
integrase
AV53_06715
exodeoxyribonuclease VIII
AV53_06730
lytic enzyme
Salmonella enterica subsp. enterica serovar Enteritidis str. EC20122031 genome−p.c.VAL54.69%−taxID1412575
AV53_06545
membrane protein
AV53_06570
hypothetical protein
AV53_06525
AV53_06530
subunit theta
AV53_06560
integrase
AV53_06565
excisionase
Salmonella enterica subsp. enterica serovar Enteritidis str. EC20122031 genome−p.c.VAL98.02%−taxID1412575
AU75_06675
tail fiber assembly protein
AU75_06685
AU75_06715
AU75_06680
type III secretion protein SopE2
AU75_06690
hypothetical protein
AU75_06700
integrase
AU75_06705
exodeoxyribonuclease VIII
AU75_06720
lytic enzyme
Salmonella enterica subsp. enterica serovar Enteritidis str. SA19961622 genome−p.c.VAL54.69%−taxID1412497

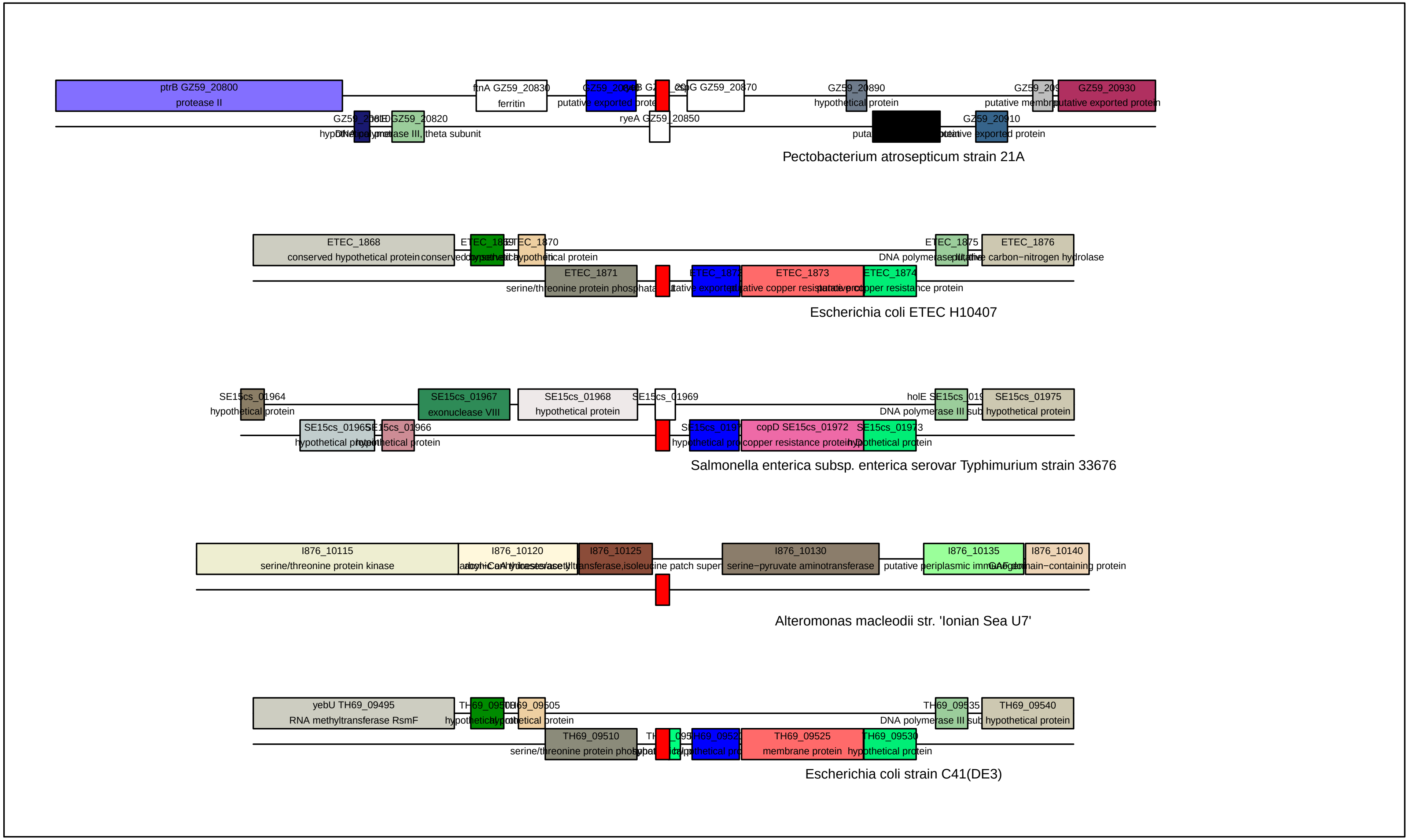

ptrB GZ59_20800
protease II
ftnA GZ59_20830
ferritin
cpoG GZ59_20870
GZ59_20890
hypothetical protein
GZ59_20930
putative exported protein
GZ59_20820
ryeA GZ59_20850
GZ59_20910
putative exported protein
Pectobacterium atrosepticum strain 21A
ETEC_1868
conserved hypothetical protein
ETEC_1870
ETEC_1875
ETEC_1876
putative carbon−nitrogen hydrolase
ETEC_1871
ETEC_1873
ETEC_1874
Escherichia coli ETEC H10407
SE15cs_01964
hypothetical protein
SE15cs_01967
exonuclease VIII
SE15cs_01968
hypothetical protein
holE
SE15cs_01975
hypothetical protein
SE15cs_01966
copD SE15cs_01972
copper resistance protein D
Salmonella enterica subsp. enterica serovar Typhimurium strain 33676
I876_10115
serine/threonine protein kinase
I876_10120
I876_10125
I876_10130
serine−pyruvate aminotransferase
I876_10135
I876_10140
Alteromonas macleodii str. 'Ionian Sea U7'
yebU TH69_09495
RNA methyltransferase RsmF
TH69_09540
hypothetical protein
TH69_09510
TH69_09525
membrane protein
TH69_09530
Escherichia coli strain C41(DE3)

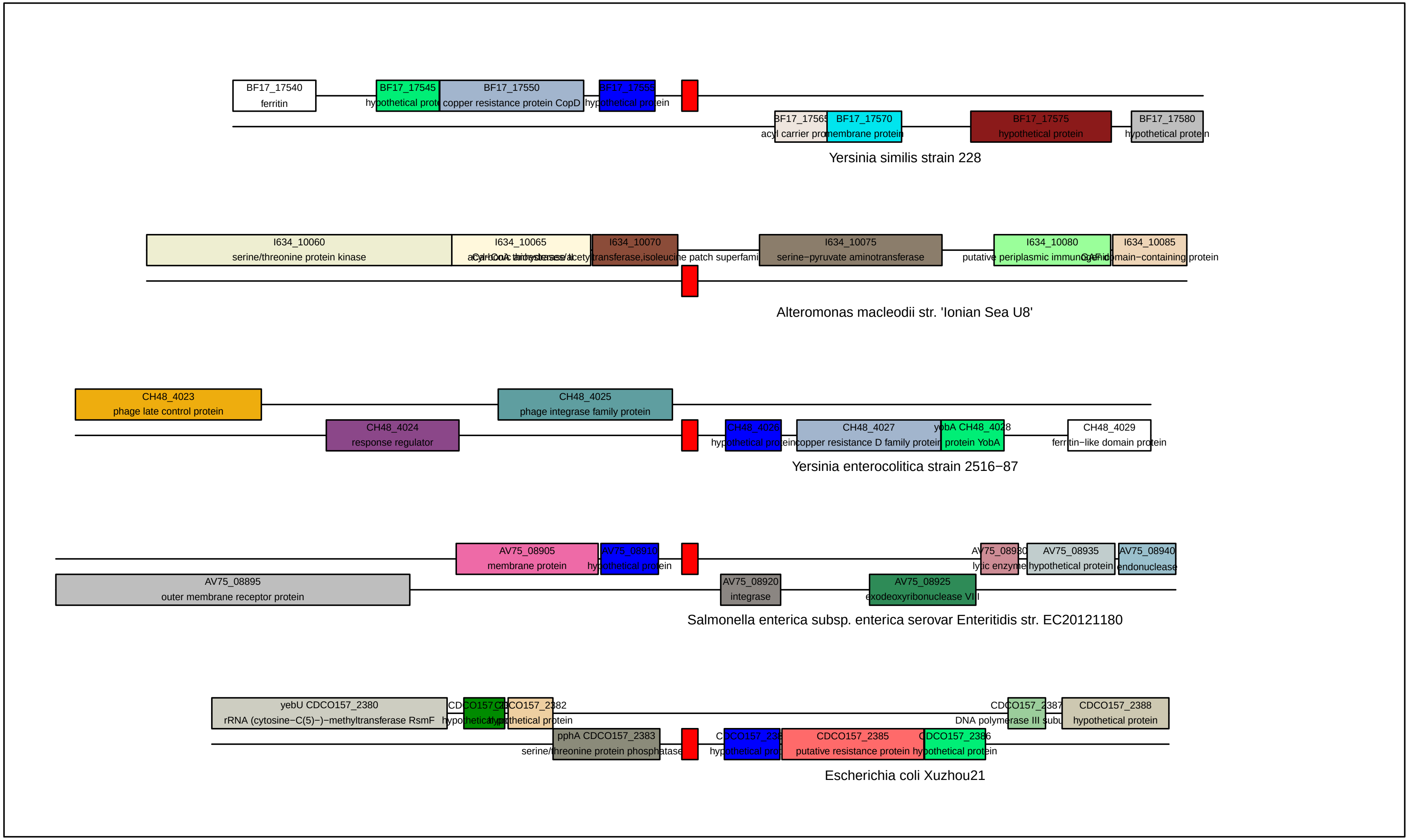
BF17_17540
ferritin
BF17_17545
BF17_17550
copper resistance protein CopD
BF17_17570
BF17_17575
hypothetical protein
BF17_17580
Yersinia similis strain 228
I634_10060
serine/threonine protein kinase
I634_10065
I634_10070
I634_10075
serine−pyruvate aminotransferase
I634_10080
I634_10085
Alteromonas macleodii str. 'Ionian Sea U8'
CH48_4023
phage late control protein
CH48_4024
response regulator
CH48_4025
phage integrase family protein
CH48_4027
copper resistance D family protein
protein YobA
CH48_4029
Yersinia enterocolitica strain 2516−87
AV75_08895
outer membrane receptor protein
AV75_08905
membrane protein
AV75_08920
integrase
AV75_08925
AV75_08935
hypothetical protein
AV75_08940
endonuclease
Salmonella enterica subsp. enterica serovar Enteritidis str. EC20121180
yebU CDCO157_2380
rRNA (cytosine−C(5)−)−methyltransferase RsmF
pphA CDCO157_2383
CDCO157_2385
putative resistance protein
CDCO157_2388
hypothetical protein
Escherichia coli Xuzhou21

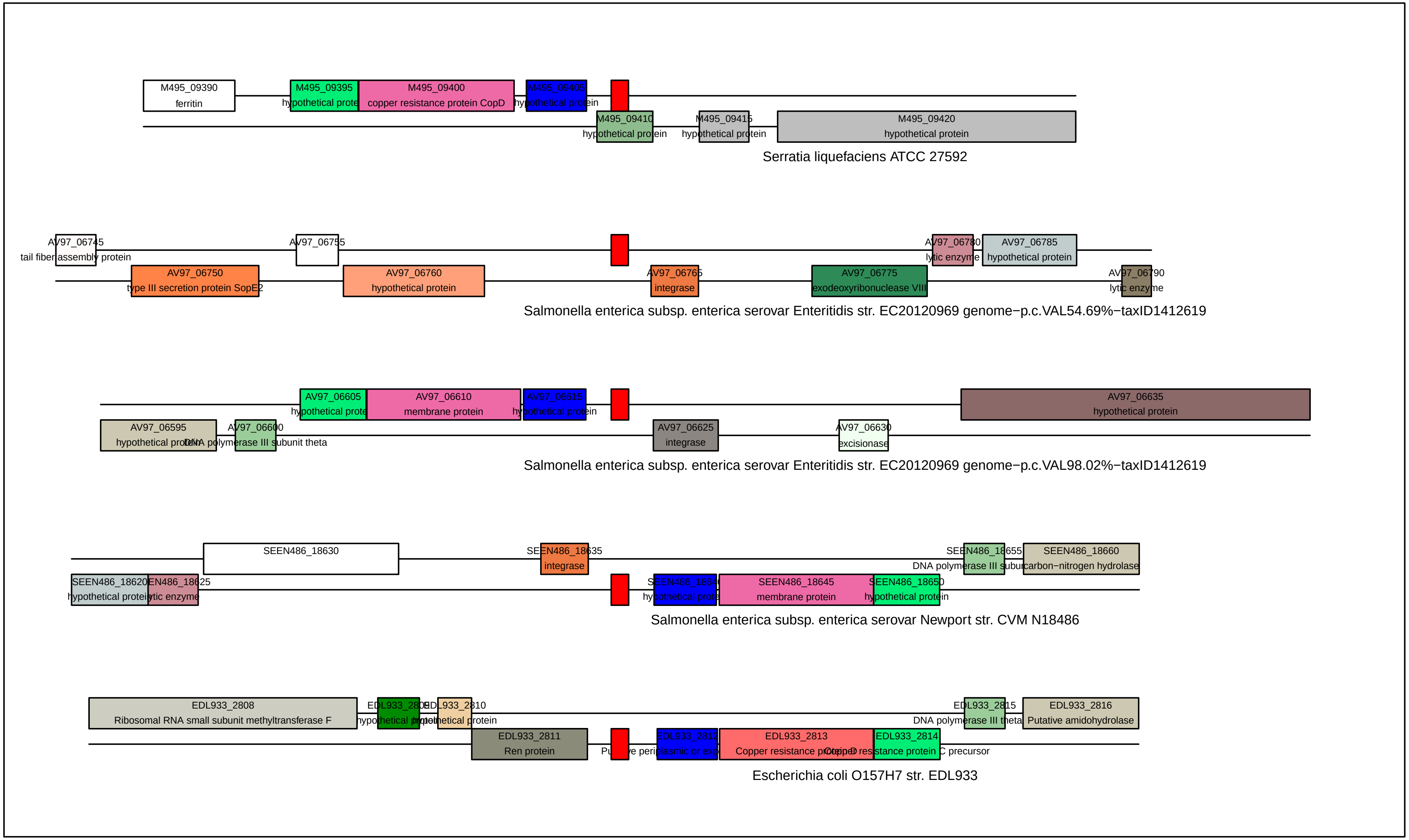

M495_09390
ferritin
M495_09395
M495_09400
copper resistance protein CopD
M495_09405
M495_09410
hypothetical protein
M495_09415
M495_09420
hypothetical protein
Serratia liquefaciens ATCC 27592
AV97_06745
tail fiber assembly protein
AV97_06755
AV97_06780
lytic enzyme
AV97_06785
hypothetical protein
AV97_06750
type III secretion protein SopE2
AV97_06760
hypothetical protein
AV97_06765
integrase
AV97_06775
exodeoxyribonuclease VIII
AV97_06790
lytic enzyme
Salmonella enterica subsp. enterica serovar Enteritidis str. EC20120969 genome−p.c.VAL54.69%−taxID1412619
AV97_06605
AV97_06610
membrane protein
AV97_06615
hypothetical protein
AV97_06635
hypothetical protein
AV97_06595
AV97_06600
DNA polymerase III subunit theta
AV97_06625
integrase
AV97_06630
excisionase
Salmonella enterica subsp. enterica serovar Enteritidis str. EC20120969 genome−p.c.VAL98.02%−taxID1412619
SEEN486_18630
SEEN486_18635
integrase
SEEN486_18655
SEEN486_18660
carbon−nitrogen hydrolase
SEEN486_18620
lytic enzyme
SEEN486_18645
membrane protein
SEEN486_18650
hypothetical protein
Salmonella enterica subsp. enterica serovar Newport str. CVM N18486
EDL933_2808
Ribosomal RNA small subunit methyltransferase F
EDL933_2810
EDL933_2815
EDL933_2816
Putative amidohydrolase
EDL933_2811
Ren protein
EDL933_2813
EDL933_2814
C precursor
Escherichia coli O157H7 str. EDL933

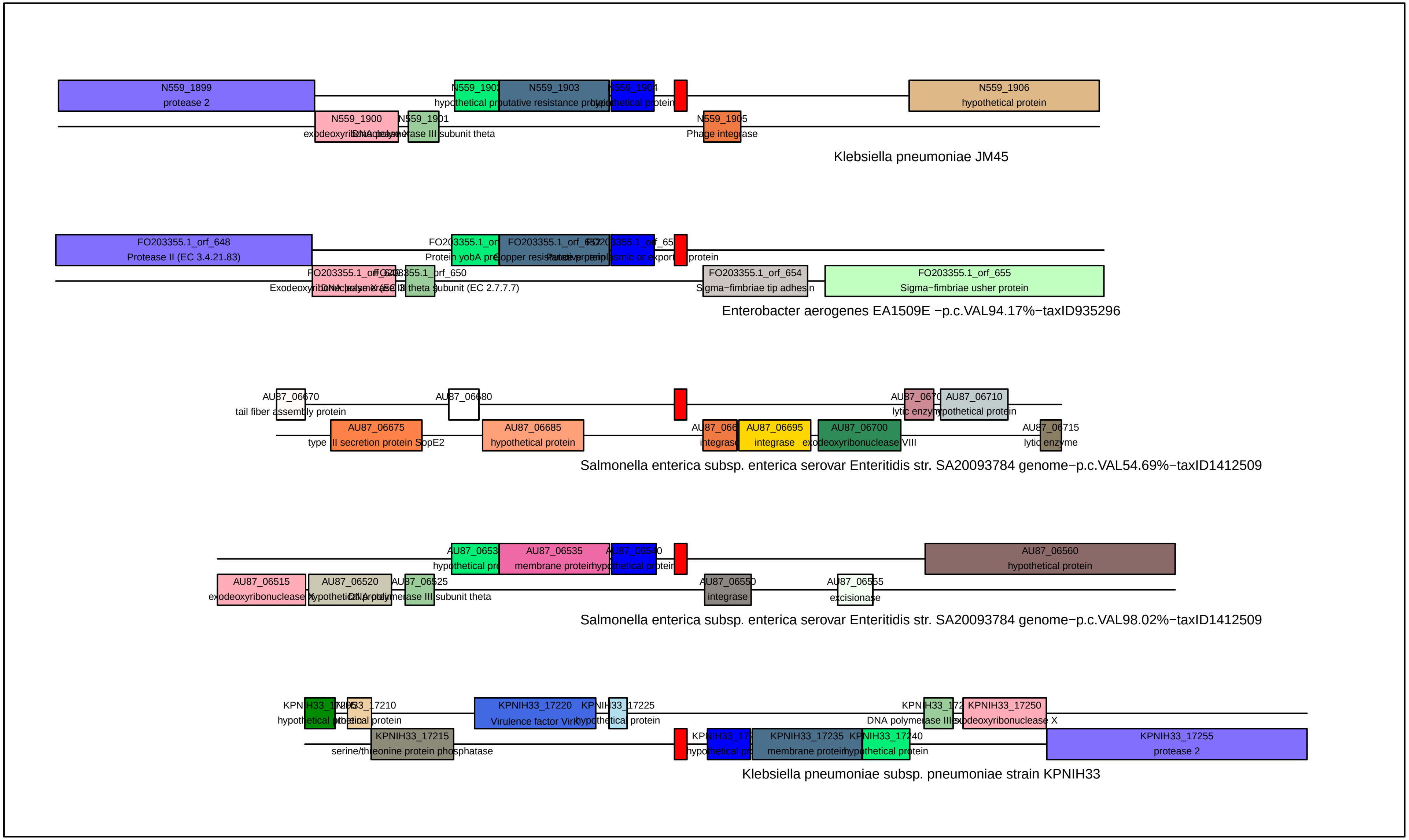

N559_1899
protease 2
N559_1903
N559_1906
hypothetical protein
N559_1900
N559_1901
subunit theta
N559_1905
Phage integrase
Klebsiella pneumoniae JM45
FO203355.1_orf_648
Protease II (EC 3.4.21.83)
FO203355.1_orf_654
FO203355.1_orf_655
Sigma−fimbriae usher protein
Enterobacter aerogenes EA1509E −p.c.VAL94.17%−taxID935296
tail fiber assembly protein
AU87_06710
AU87_06675
AU87_06685
hypothetical protein
AU87_06695
integrase
AU87_06700
AU87_06715
lytic enzyme
Salmonella enterica subsp. enterica serovar Enteritidis str. SA20093784 genome−p.c.VAL54.69%−taxID1412509
AU87_06535
AU87_06560
hypothetical protein
AU87_06515
AU87_06520
AU87_06550
integrase
subunit theta
Salmonella enterica subsp. enterica serovar Enteritidis str. SA20093784 genome−p.c.VAL98.02%−taxID1412509
KPNIH33_17220
KPNIH33_17250
KPNIH33_17215
serine/threonine protein phosphatase
KPNIH33_17235
KPNIH33_17240
KPNIH33_17255
protease 2
Klebsiella pneumoniae subsp. pneumoniae strain KPNIH33

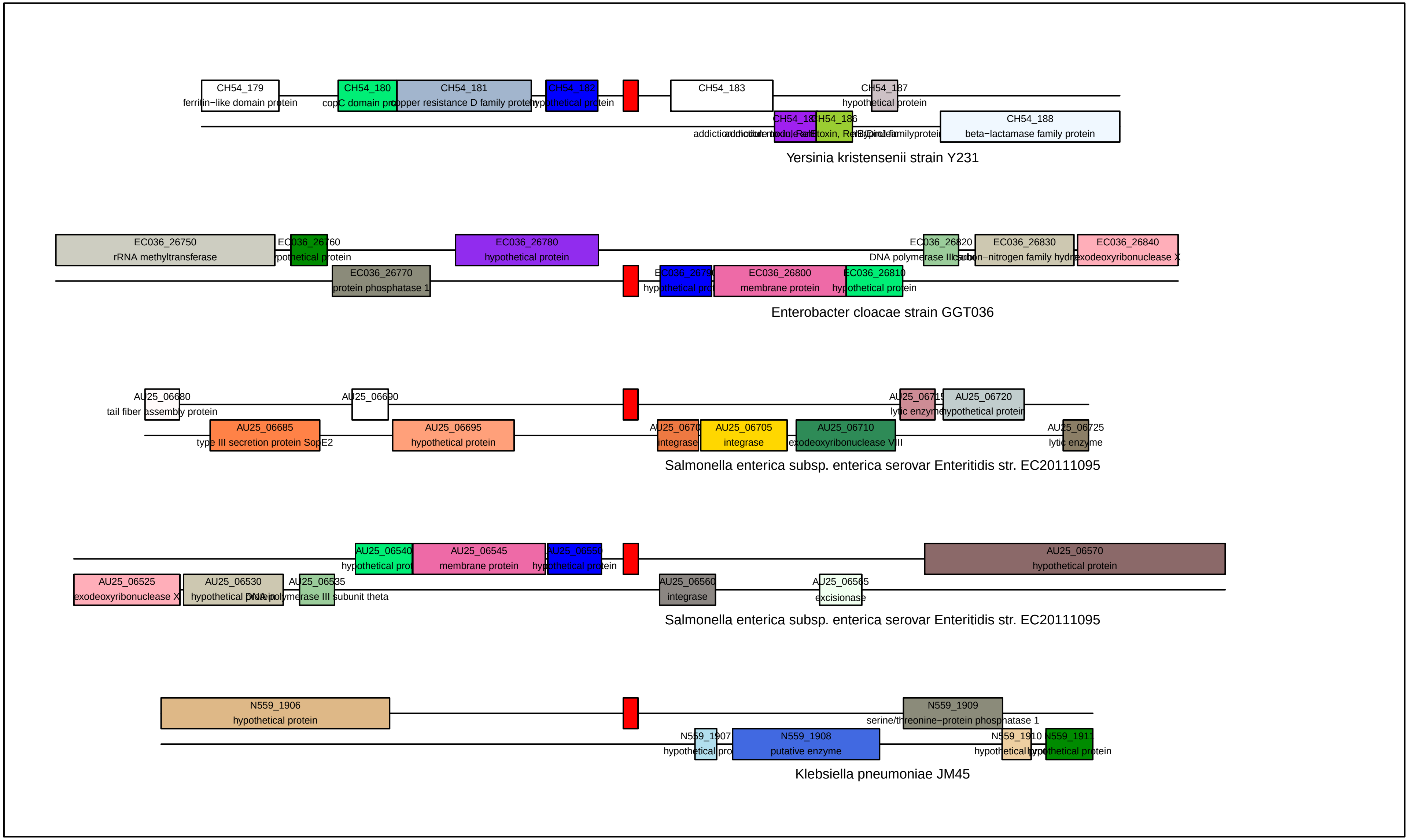
CH54_179
ferritin−like domain protein
CH54_180
CH54_181
copper resistance D family protein
CH54_182
hypothetical protein
CH54_183
CH54_187
hypothetical protein
CH54_188
beta−lactamase family protein
Yersinia kristensenii strain Y231
EC036_26750
rRNA methyltransferase
EC036_26760
EC036_26770
protein phosphatase 1
EC036_26780
hypothetical protein
EC036_26800
membrane protein
EC036_26810
hypothetical protein
EC036_26820
EC036_26830
EC036_26840
exodeoxyribonuclease X
Enterobacter cloacae strain GGT036
AU25_06680
tail fiber assembly protein
AU25_06685
type III secretion protein SopE2
AU25_06690
AU25_06695
hypothetical protein
AU25_06705
integrase
AU25_06710
exodeoxyribonuclease VIII
AU25_06720
hypothetical protein
AU25_06725
lytic enzyme
Salmonella enterica subsp. enterica serovar Enteritidis str. EC20111095
AU25_06525
exodeoxyribonuclease X
AU25_06530
AU25_06535
DNA polymerase III subunit theta
AU25_06540
AU25_06545
membrane protein
AU25_06550
hypothetical protein
AU25_06560
integrase
AU25_06565
excisionase
AU25_06570
hypothetical protein
Salmonella enterica subsp. enterica serovar Enteritidis str. EC20111095
N559_1906
hypothetical protein
N559_1907
N559_1908
putative enzyme
N559_1909
serine/threonine−protein phosphatase 1
N559_1910
N559_1911
hypothetical protein
Klebsiella pneumoniae JM45

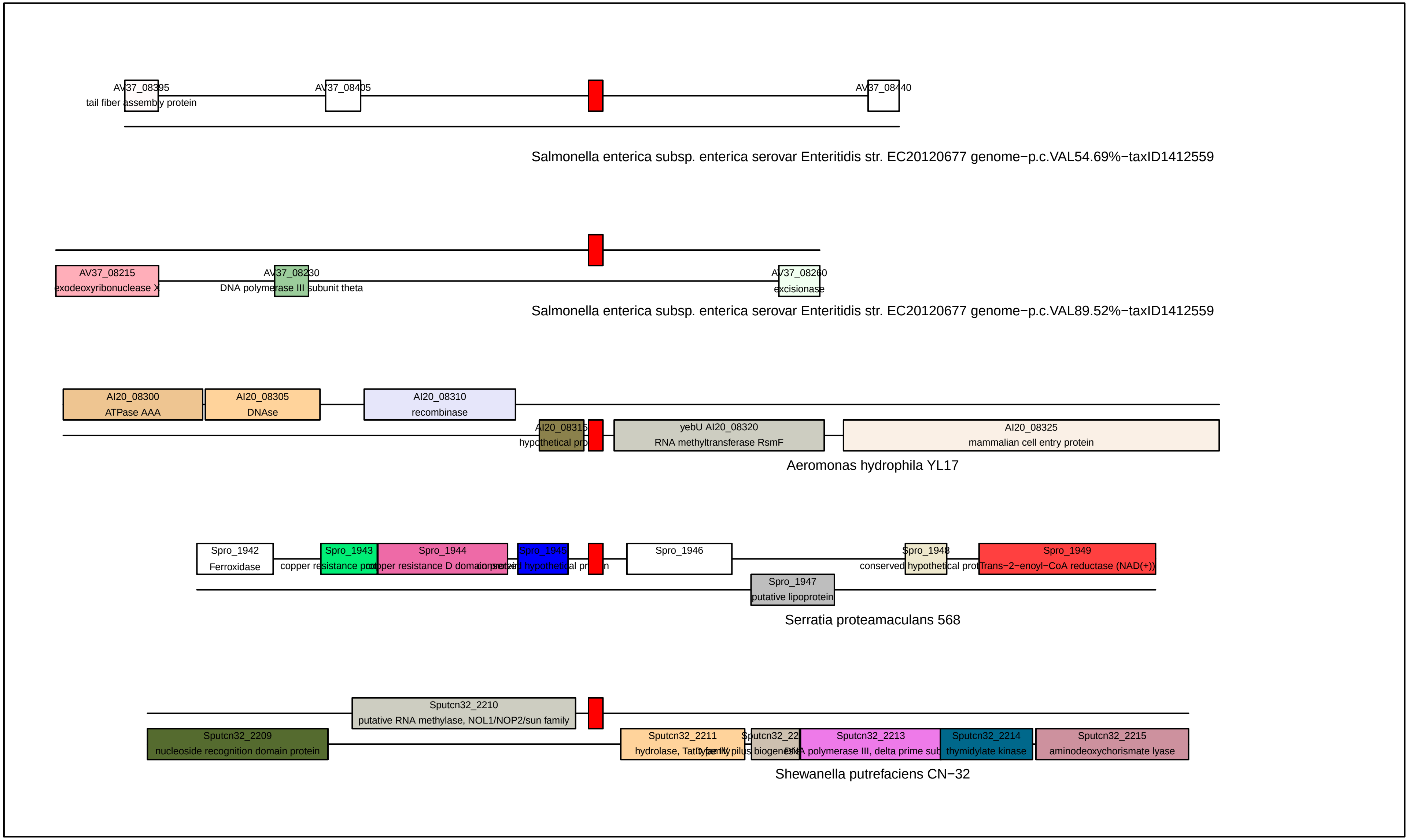
AV37_08395
tail fiber assembly protein
AV37_08405
AV37_08440
Salmonella enterica subsp. enterica serovar Enteritidis str. EC20120677 genome−p.c.VAL54.69%−taxID1412559
AV37_08215
exodeoxyribonuclease X
AV37_08230
DNA polymerase III subunit theta
AV37_08260
excisionase
Salmonella enterica subsp. enterica serovar Enteritidis str. EC20120677 genome−p.c.VAL89.52%−taxID1412559
AI20_08300
ATPase AAA
AI20_08305
DNAse
AI20_08310
recombinase
AI20_08315
yebU AI20_08320
RNA methyltransferase RsmF
AI20_08325
mammalian cell entry protein
Aeromonas hydrophila YL17
Spro_1942
Ferroxidase
Spro_1943
Spro_1944
Spro_1946
Spro_1948
Spro_1949
Trans−2−enoyl−CoA reductase (NAD(+))
Spro_1947
putative lipoprotein
Serratia proteamaculans 568
Sputcn32_2210
putative RNA methylase, NOL1/NOP2/sun family
Sputcn32_2209
nucleoside recognition domain protein
Sputcn32_2211
Sputcn32_2213
Sputcn32_2214
thymidylate kinase
Sputcn32_2215
aminodeoxychorismate lyase
Shewanella putrefaciens CN−32

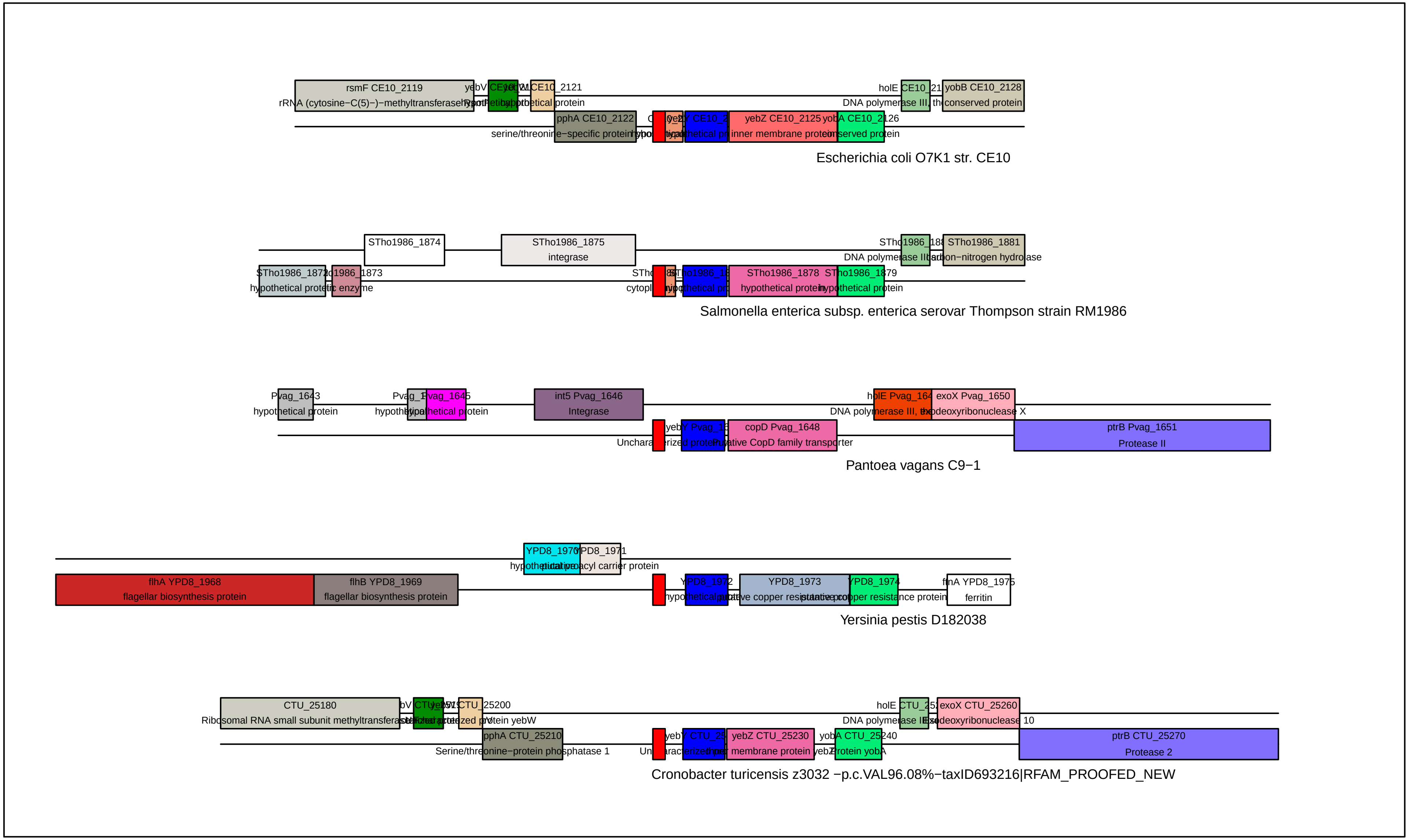
Escherichia coli O7K1 str. CE10
Salmonella enterica subsp. enterica serovar Thompson strain RM1986
Pantoea vagans C9−1
Yersinia pestis D182038
Cronobacter turicensis z3032 −p.c.VAL96.08%−taxID693216|RFAM_PROOFED_NEW
rsmF CE10_2119
pphA CE10_2122
yebZ CE10_2125
yobB CE10_2128
STho1986_1874
STho1986_1875
integrase
STho1986_1878
STho1986_1879
STho1986_1881
int5 Pvag_1646
Integrase
copD Pvag_1648
exoX Pvag_1650
ptrB Pvag_1651
Protease II
flhA YPD8_1968
flagellar biosynthesis protein
flhB YPD8_1969
flagellar biosynthesis protein
YPD8_1973
ferritin
CTU_25180
pphA CTU_25210
yebZ CTU_25230
exoX CTU_25260
ptrB CTU_25270
Protease 2

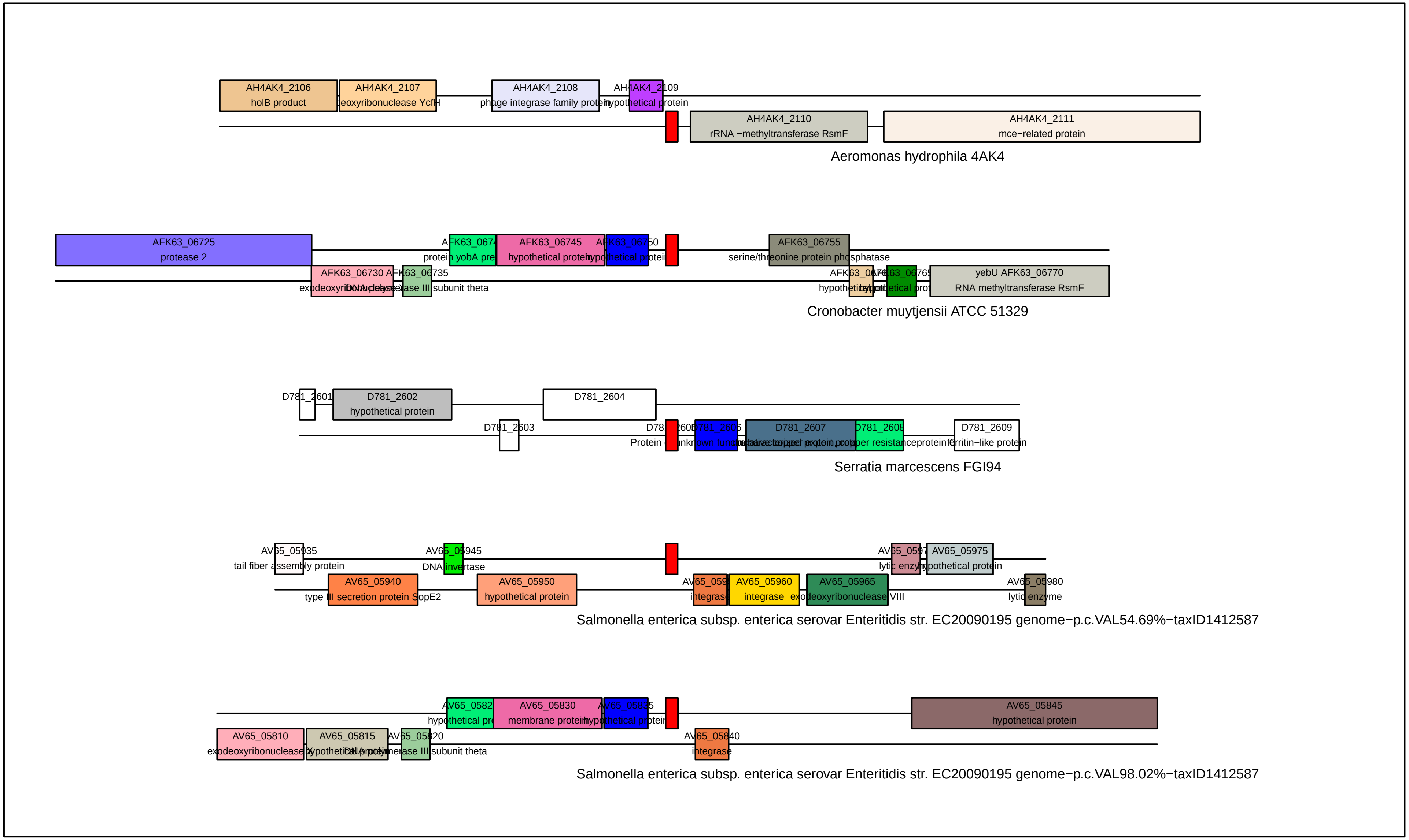
AH4AK4_2106
holB product
AH4AK4_2107
AH4AK4_2108
phage integrase family protein
AH4AK4_2109
hypothetical protein
AH4AK4_2110
rRNA −methyltransferase RsmF
AH4AK4_2111
mce−related protein
Aeromonas hydrophila 4AK4
AFK63_06725
protease 2
AFK63_06745
hypothetical protein
AFK63_06755
serine/threonine protein phosphatase
AFK63_06730
AFK63_06735
subunit theta
yebU AFK63_06770
RNA methyltransferase RsmF
Cronobacter muytjensii ATCC 51329
D781_2602
hypothetical protein
D781_2604
D781_2603
D781_2607
D781_2609
Protein
ferritin−like protein
Serratia marcescens FGI94
AV65_05935
tail fiber assembly protein
AV65_05945
DNA invertase
AV65_05975
hypothetical protein
AV65_05940
type III secretion protein SopE2
AV65_05950
hypothetical protein
AV65_05960
integrase
AV65_05965
exodeoxyribonuclease VIII
AV65_05980
lytic enzyme
Salmonella enterica subsp. enterica serovar Enteritidis str. EC20090195 genome−p.c.VAL54.69%−taxID1412587
AV65_05830
membrane protein
AV65_05845
hypothetical protein
AV65_05810
exodeoxyribonuclease X
AV65_05815
AV65_05820
subunit theta
AV65_05840
integrase
Salmonella enterica subsp. enterica serovar Enteritidis str. EC20090195 genome−p.c.VAL98.02%−taxID1412587

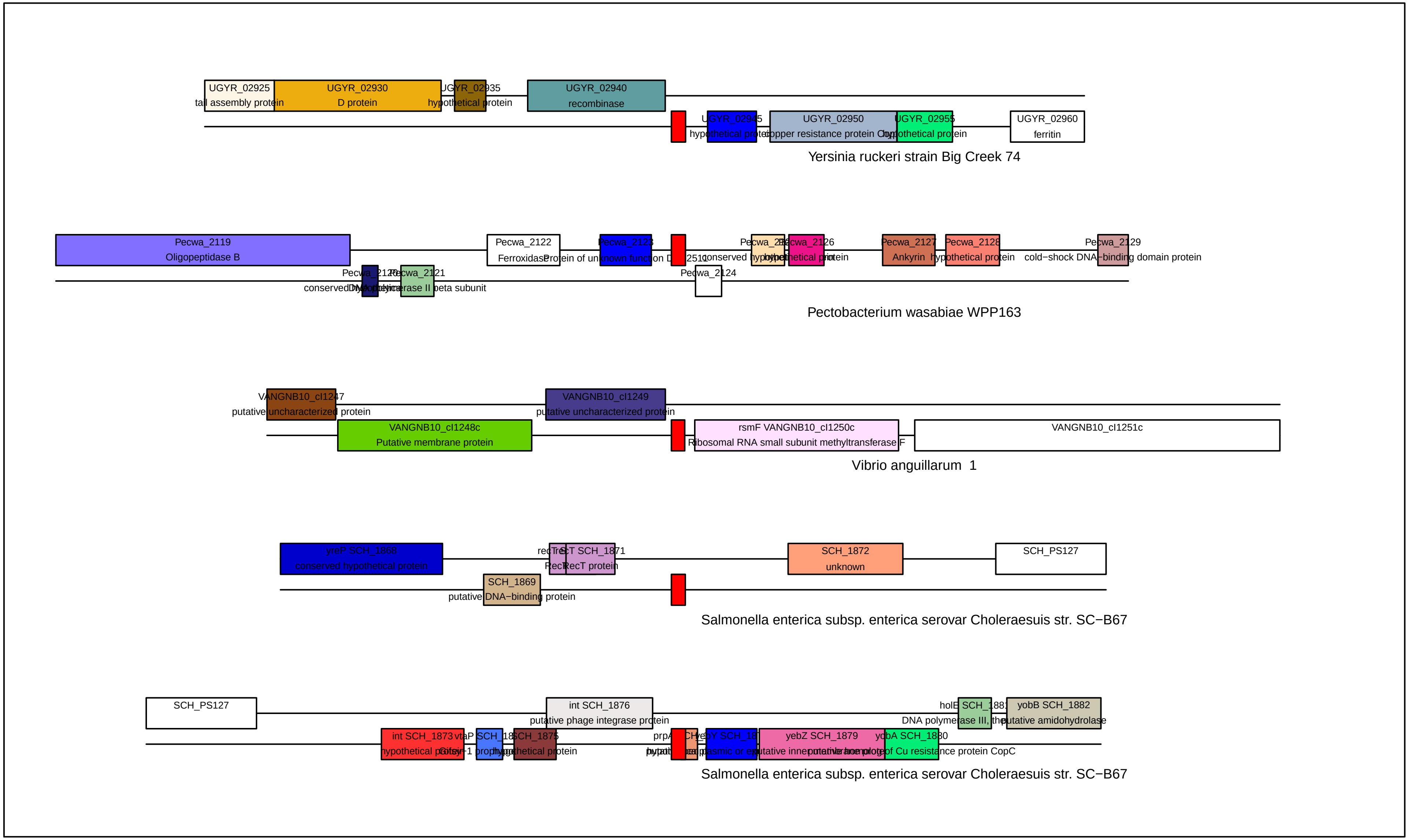

UGYR_02925
UGYR_02930
D protein
UGYR_02940
recombinase
UGYR_02950
UGYR_02960
ferritin
Yersinia ruckeri strain Big Creek 74
Pecwa_2119
Oligopeptidase B
Pecwa_2122
Pecwa_2124
Pecwa_2127
Ankyrin
cold−shock DNA−binding domain protein
Pectobacterium wasabiae WPP163
VANGNB10_cI1249
VANGNB10_cI1248c
Putative membrane protein
rsmF VANGNB10_cI1250c
VANGNB10_cI1251c
Vibrio anguillarum 1
SCH_1872
unknown
SCH_PS127
SCH_1869
Salmonella enterica subsp. enterica serovar Choleraesuis str. SC−B67
SCH_PS127
int SCH_1876
putative phage integrase protein
yobB SCH_1882
yebZ SCH_1879
Salmonella enterica subsp. enterica serovar Choleraesuis str. SC−B67

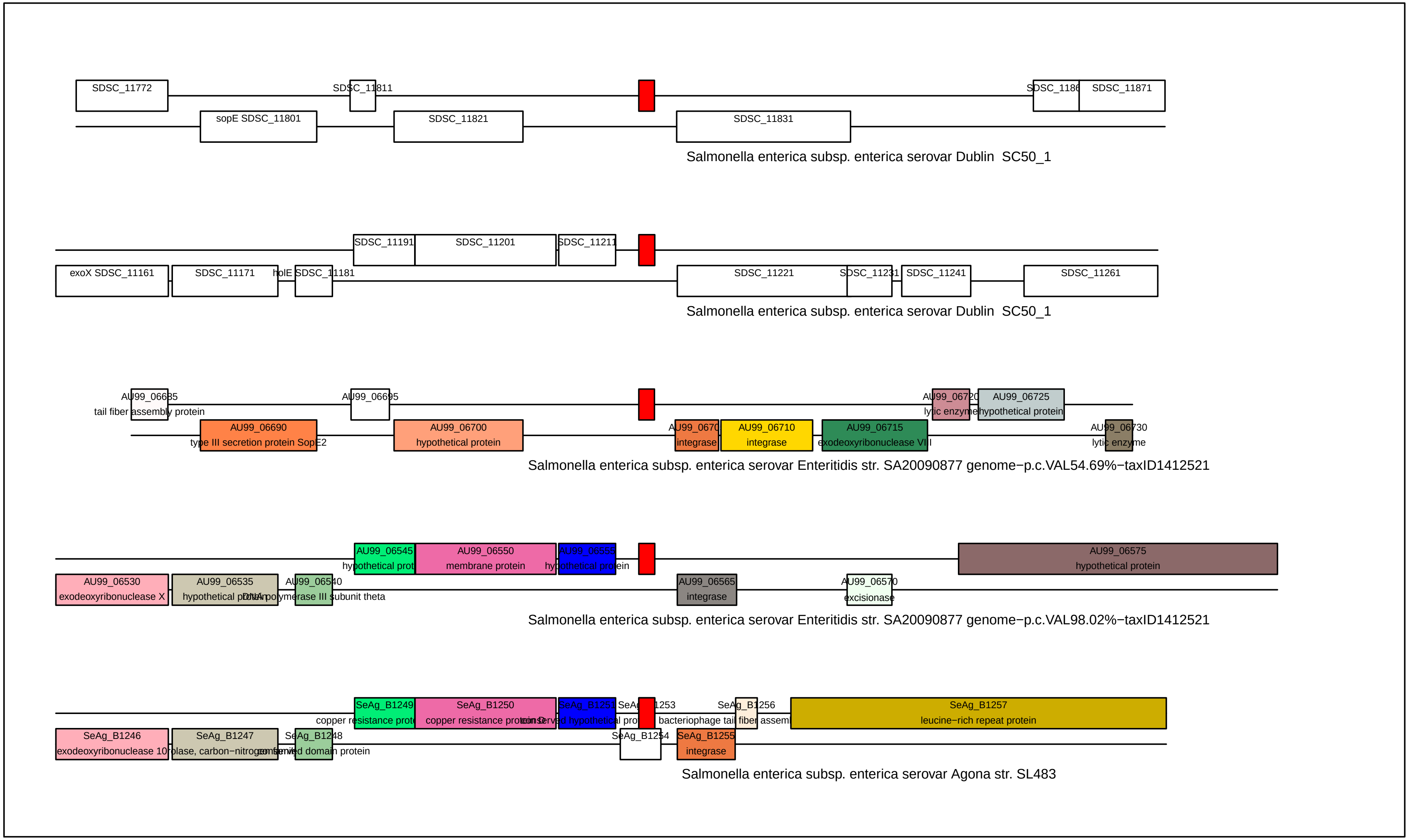

SDSC_11772
SDSC_11811
SDSC_11871
sopE SDSC_11801
SDSC_11821
SDSC_11831
Salmonella enterica subsp. enterica serovar Dublin SC50_1
SDSC_11191
SDSC_11201
SDSC_11211
exoX SDSC_11161
SDSC_11171
holE SDSC_11181
SDSC_11221
SDSC_11231
SDSC_11241
SDSC_11261
Salmonella enterica subsp. enterica serovar Dublin SC50_1
AU99_06685
tail fiber assembly protein
AU99_06695
AU99_06725
hypothetical protein
AU99_06690
type III secretion protein SopE2
AU99_06700
hypothetical protein
integrase
AU99_06710
integrase
AU99_06715
exodeoxyribonuclease VIII
AU99_06730
lytic enzyme
Salmonella enterica subsp. enterica serovar Enteritidis str. SA20090877 genome−p.c.VAL54.69%−taxID1412521
AU99_06545
AU99_06550
membrane protein
AU99_06555
AU99_06575
hypothetical protein
AU99_06530
exodeoxyribonuclease X
AU99_06535
AU99_06540
DNA polymerase III subunit theta
AU99_06565
integrase
AU99_06570
excisionase
Salmonella enterica subsp. enterica serovar Enteritidis str. SA20090877 genome−p.c.VAL98.02%−taxID1412521
SeAg_B1249
SeAg_B1250
copper resistance protein D
SeAg_B1256
SeAg_B1257
leucine−rich repeat protein
SeAg_B1246
SeAg_B1247
SeAg_B1248
SeAg_B1254
SeAg_B1255
integrase
Salmonella enterica subsp. enterica serovar Agona str. SL483

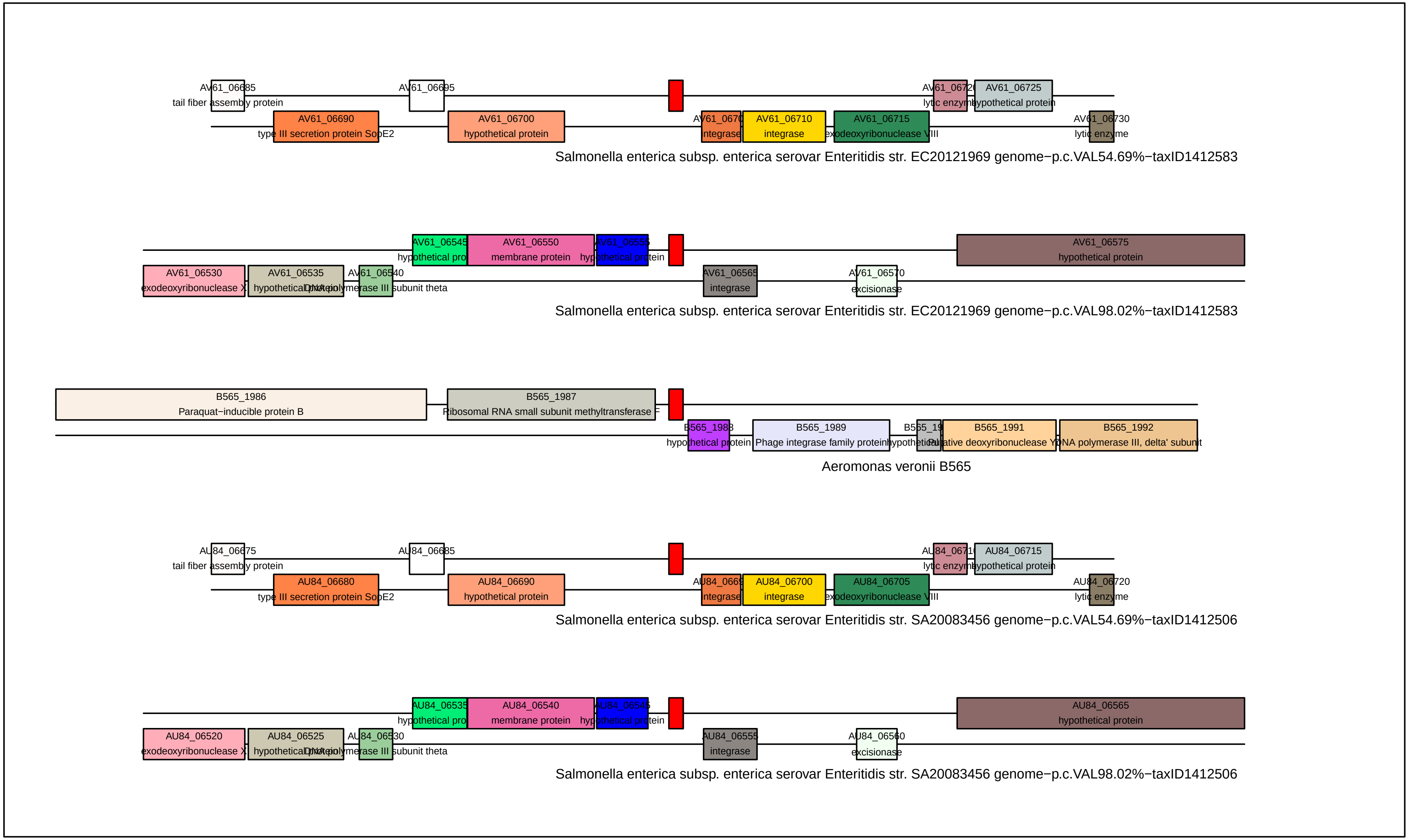

AV61_06685
tail fiber assembly protein
AV61_06690
type III secretion protein SopE2
AV61_06695
AV61_06700
hypothetical protein
integrase
AV61_06710
integrase
AV61_06715
exodeoxyribonuclease VIII
lytic enzyme
AV61_06725
hypothetical protein
AV61_06730
lytic enzyme
Salmonella enterica subsp. enterica serovar Enteritidis str. EC20121969 genome−p.c.VAL54.69%−taxID1412583
AV61_06530
exodeoxyribonuclease X
AV61_06535
hypothetical protein
DNA polymerase III subunit theta
AV61_06540
AV61_06545
AV61_06550
membrane protein
hypothetical protein
AV61_06565
integrase
AV61_06570
excisionase
AV61_06575
hypothetical protein
Salmonella enterica subsp. enterica serovar Enteritidis str. EC20121969 genome−p.c.VAL98.02%−taxID1412583
B565_1986
Paraquat−inducible protein B
B565_1987
Ribosomal RNA small subunit methyltransferase F
B565_1988
hypothetical protein
B565_1989
Phage integrase family protein
B565_1991
Putative deoxyribonuclease YjjV
B565_1992
DNA polymerase III, delta' subunit
Aeromonas veronii B565
AU84_06675
tail fiber assembly protein
AU84_06680
type III secretion protein SopE2
AU84_06685
AU84_06690
hypothetical protein
integrase
AU84_06700
integrase
AU84_06705
exodeoxyribonuclease VIII
lytic enzyme
AU84_06715
hypothetical protein
AU84_06720
lytic enzyme
Salmonella enterica subsp. enterica serovar Enteritidis str. SA20083456 genome−p.c.VAL54.69%−taxID1412506
AU84_06520
exodeoxyribonuclease X
AU84_06525
hypothetical protein
DNA polymerase III subunit theta
AU84_06530
AU84_06535
AU84_06540
membrane protein
hypothetical protein
AU84_06555
integrase
AU84_06560
excisionase
AU84_06565
hypothetical protein
Salmonella enterica subsp. enterica serovar Enteritidis str. SA20083456 genome−p.c.VAL98.02%−taxID1412506

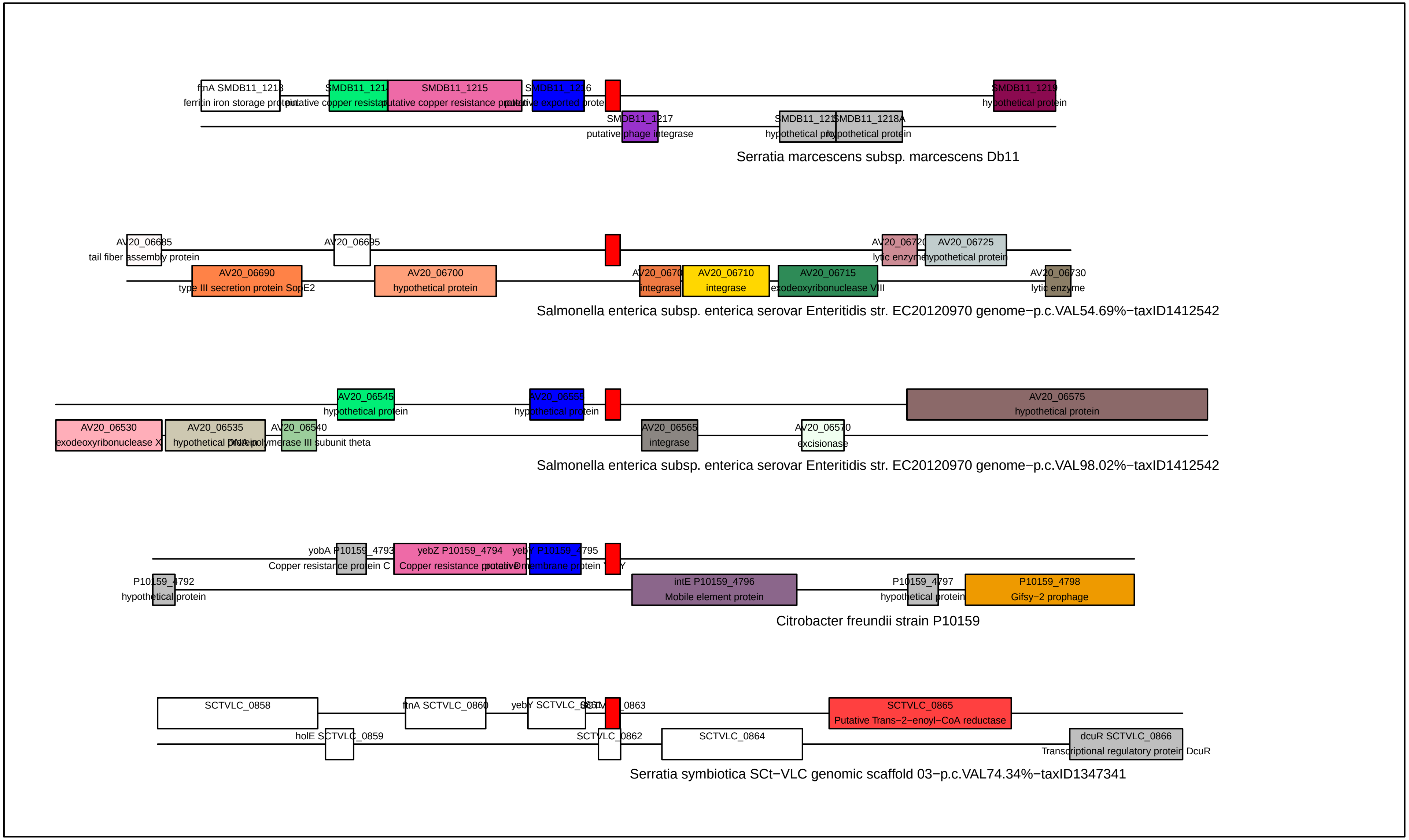

ftnA SMDB11_1213
ferritin iron storage protein
SMDB11_1215
putative copper resistance protein
SMDB11_1217
putative phage integrase
SMDB11_1218A
hypothetical protein
SMDB11_1219
hypothetical protein
Serratia marcescens subsp. marcescens Db11
AV20_06685
tail fiber assembly protein
AV20_06695
AV20_06690
type III secretion protein SopE2
AV20_06700
hypothetical protein
integrase
AV20_06710
integrase
AV20_06715
exodeoxyribonuclease VIII
lytic enzyme
AV20_06725
hypothetical protein
AV20_06730
lytic enzyme
Salmonella enterica subsp. enterica serovar Enteritidis str. EC20120970 genome−p.c.VAL54.69%−taxID1412542
AV20_06545
hypothetical protein
AV20_06575
hypothetical protein
AV20_06530
exodeoxyribonuclease X
AV20_06535
AV20_06540
subunit theta
AV20_06565
integrase
AV20_06570
excisionase
Salmonella enterica subsp. enterica serovar Enteritidis str. EC20120970 genome−p.c.VAL98.02%−taxID1412542
yebZ P10159_4794
P10159_4792
hypothetical protein
intE P10159_4796
Mobile element protein
P10159_4797
hypothetical protein
P10159_4798
Gifsy−2 prophage
Citrobacter freundii strain P10159
SCTVLC_0858
ftnA SCTVLC_0860
holE SCTVLC_0859
SCTVLC_0864
SCTVLC_0865
Putative Trans−2−enoyl−CoA reductase
dcuR SCTVLC_0866
Transcriptional regulatory protein DcuR
Serratia symbiotica SCt−VLC genomic scaffold 03−p.c.VAL74.34%−taxID1347341

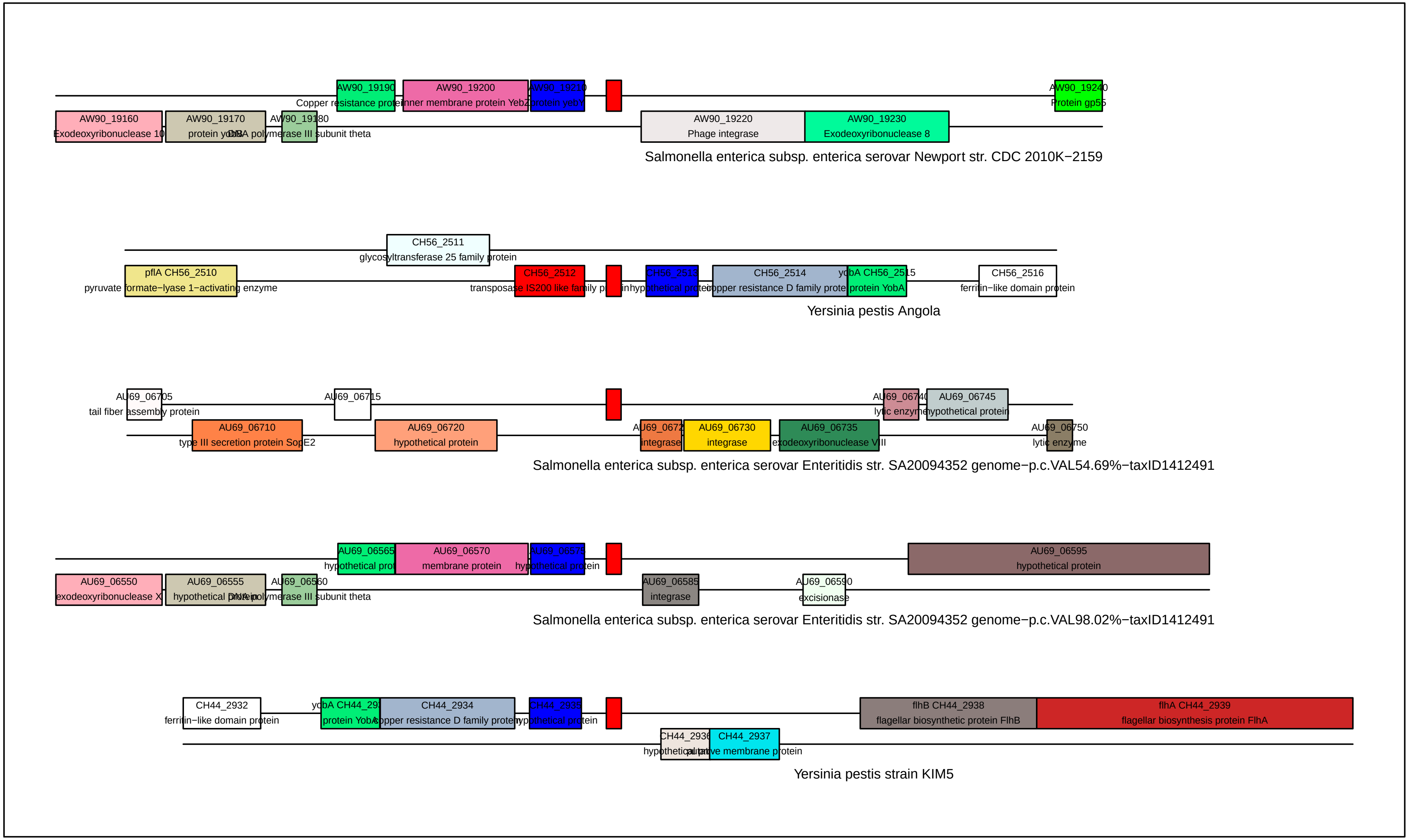

AW90_19160
AW90_19170
AW90_19190
AW90_19200
AW90_19220
Phage integrase
AW90_19230
Exodeoxyribonuclease 8
AW90_19240
Salmonella enterica subsp. enterica serovar Newport str. CDC 2010K−2159
CH56_2511
glycosyltransferase 25 family protein
pflA CH56_2510
pyruvate formate−lyase 1−activating enzyme
CH56_2512
CH56_2514
CH56_2516
ferritin−like domain protein
Yersinia pestis Angola
AU69_06705
tail fiber assembly protein
AU69_06715
AU69_06745
AU69_06710
type III secretion protein SopE2
AU69_06720
hypothetical protein
AU69_06730
integrase
AU69_06735
exodeoxyribonuclease VIII
AU69_06750
lytic enzyme
Salmonella enterica subsp. enterica serovar Enteritidis str. SA20094352 genome−p.c.VAL54.69%−taxID1412491
AU69_06570
membrane protein
AU69_06595
hypothetical protein
AU69_06550
AU69_06555
AU69_06585
integrase
AU69_06590
excisionase
Salmonella enterica subsp. enterica serovar Enteritidis str. SA20094352 genome−p.c.VAL98.02%−taxID1412491
CH44_2932
ferritin−like domain protein
CH44_2934
flhB CH44_2938
flagellar biosynthetic protein FlhB
flhA CH44_2939
flagellar biosynthesis protein FlhA
CH44_2937
Yersinia pestis strain KIM5

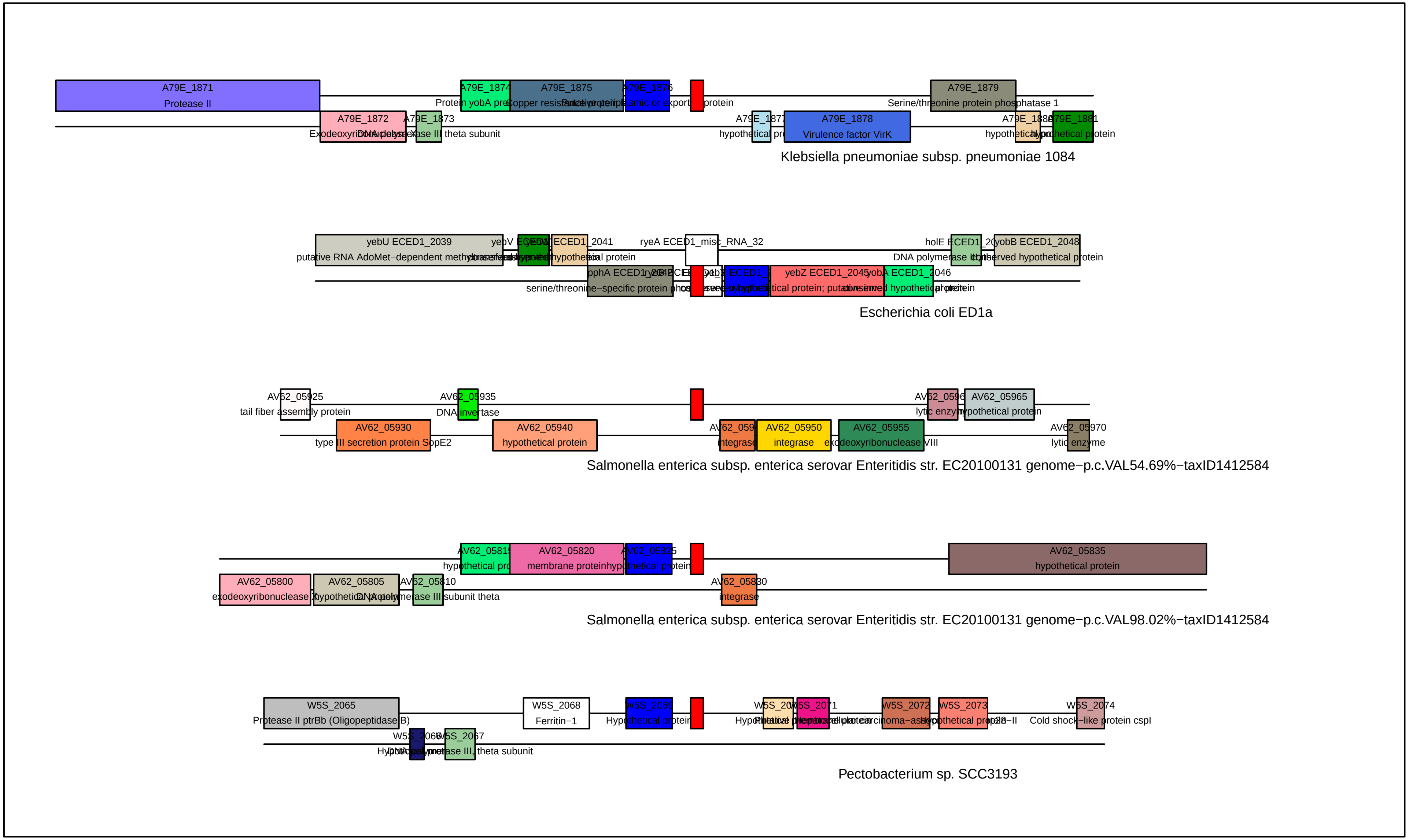
A79E_1871
Protease II
A79E_1872
A79E_1873
A79E_1874
A79E_1875
A79E_1876
A79E_1877
A79E_1878
Virulence factor VirK
A79E_1879
Serine/threonine protein phosphatase 1
Klebsiella pneumoniae subsp. pneumoniae 1084
yebU ECED1_2039
ryeA ECED1_misc_RNA_32
yebZ ECED1_2045
yobB ECED1_2048
Escherichia coli ED1a
AV62_05925
tail fiber assembly protein
AV62_05930
type III secretion protein SopE2
AV62_05935
DNA invertase
AV62_05940
hypothetical protein
AV62_05950
integrase
AV62_05955
exodeoxyribonuclease VIII
AV62_05965
hypothetical protein
AV62_05970
lytic enzyme
Salmonella enterica subsp. enterica serovar Enteritidis str. EC20100131 genome−p.c.VAL54.69%−taxID1412584
AV62_05800
exodeoxyribonuclease I
AV62_05805
AV62_05820
membrane protein
AV62_05835
hypothetical protein
AV62_05830
integrase
Salmonella enterica subsp. enterica serovar Enteritidis str. EC20100131 genome−p.c.VAL98.02%−taxID1412584
W5S_2065
Protease II ptrBb (Oligopeptidase B)
W5S_2067
W5S_2068
Ferritin−1
W5S_2071
W5S_2072
W5S_2073
W5S_2074
Cold shock−like protein cspI
Pectobacterium sp. SCC3193

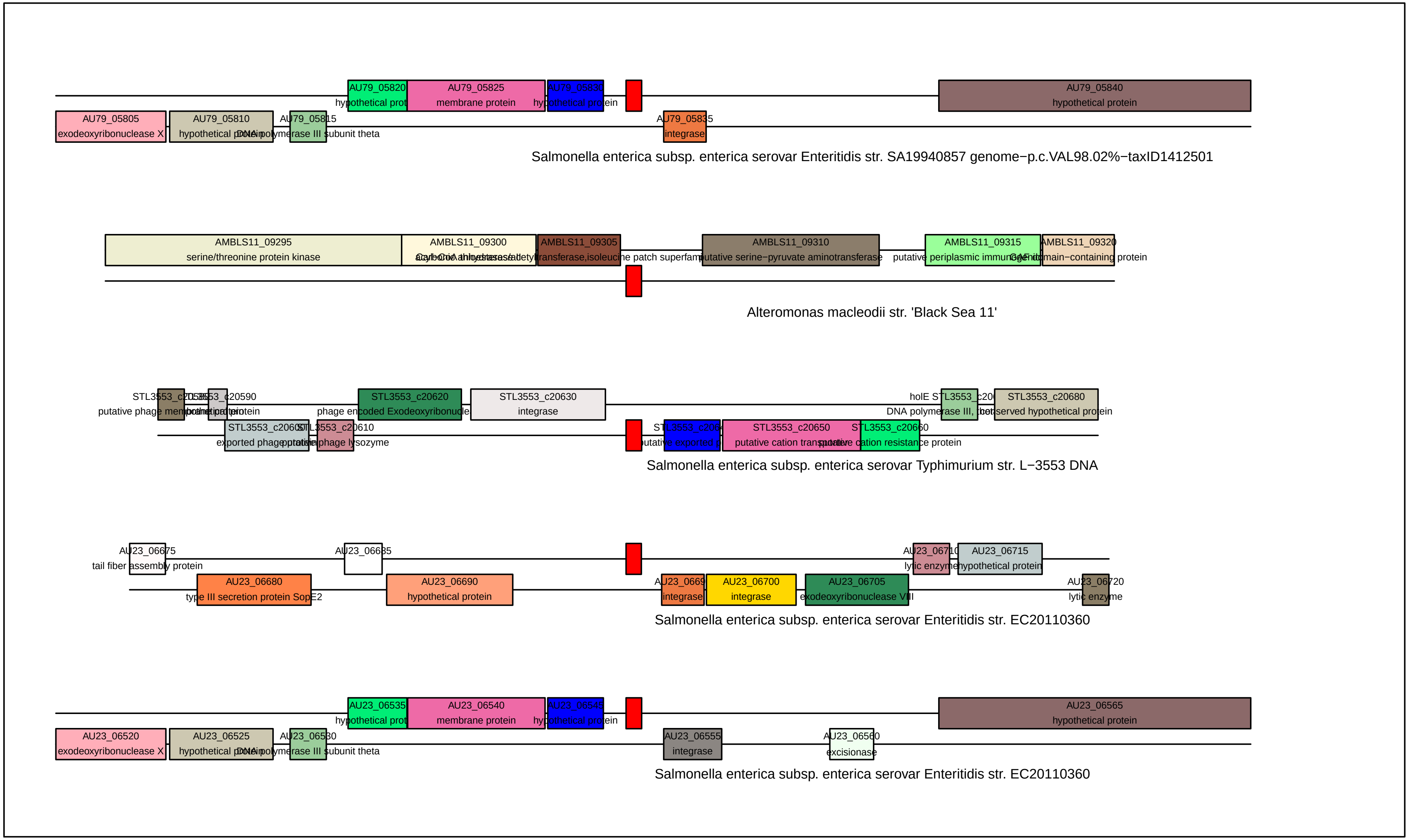

AU79_05840
hypothetical protein
AU79_05825
membrane protein
AU79_05805
exodeoxyribonuclease X
AU79_05810
AU79_05835
integrase
Salmonella enterica subsp. enterica serovar Enteritidis str. SA19940857 genome−p.c.VAL98.02%−taxID1412501
AMBLS11_09295
serine/threonine protein kinase
AMBLS11_09300
AMBLS11_09305
AMBLS11_09310
putative serine−pyruvate aminotransferase
AMBLS11_09315
Alteromonas macleodii str. 'Black Sea 11'
STL3553_c20620
STL3553_c20630
integrase
STL3553_c20680
STL3553_c20650
putative cation transporter
phage lysozyme
Salmonella enterica subsp. enterica serovar Typhimurium str. L−3553 DNA
AU23_06675
tail fiber assembly protein
AU23_06680
type III secretion protein SopE2
AU23_06690
hypothetical protein
AU23_06700
integrase
AU23_06705
exodeoxyribonuclease VIII
AU23_06715
hypothetical protein
lytic enzyme
Salmonella enterica subsp. enterica serovar Enteritidis str. EC20110360
AU23_06565
hypothetical protein
AU23_06540
membrane protein
AU23_06520
exodeoxyribonuclease X
AU23_06525
AU23_06555
integrase
excisionase
Salmonella enterica subsp. enterica serovar Enteritidis str. EC20110360

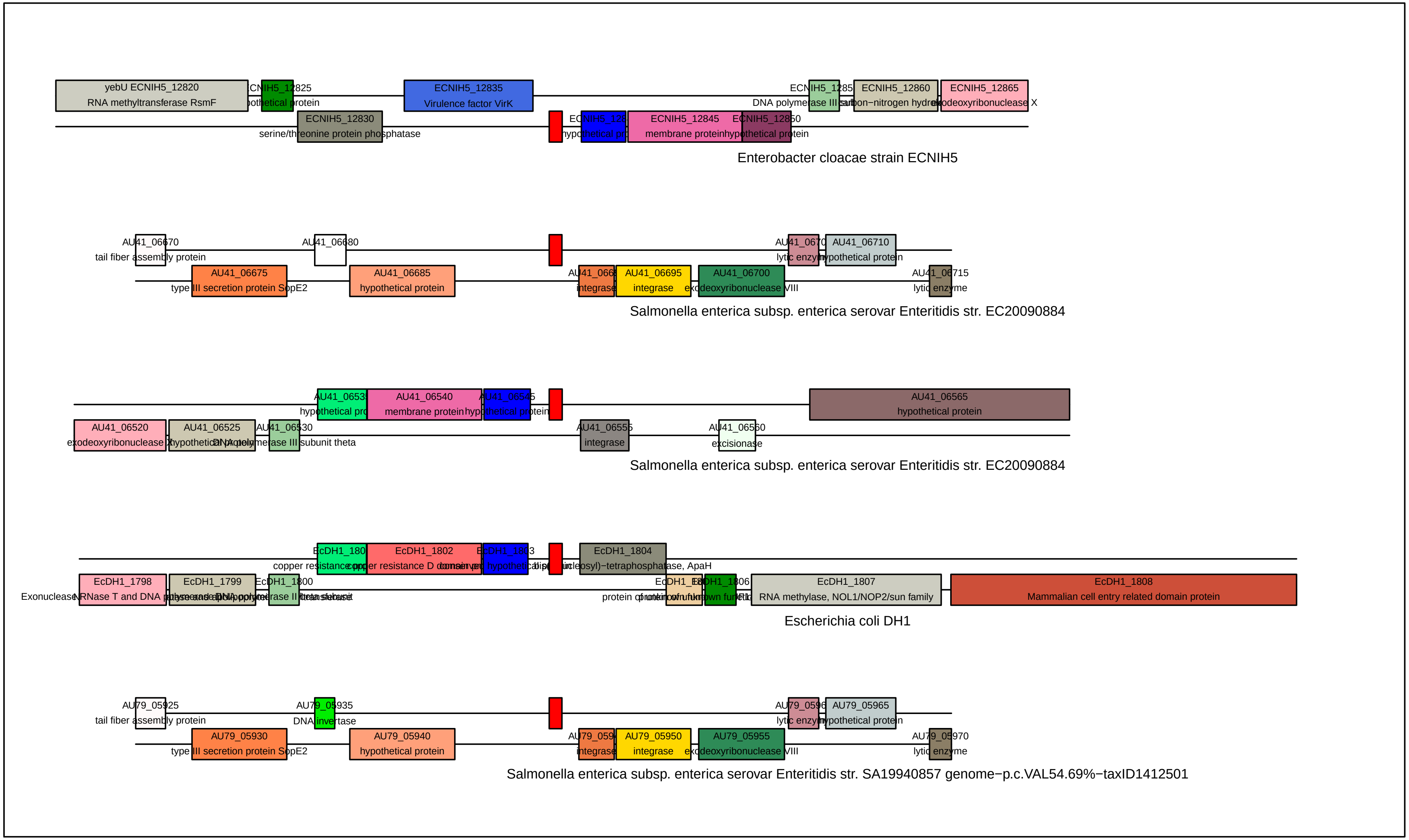

yebU ECNIH5_12820
RNA methyltransferase RsmF
ECNIH5_12830
serine/threonine protein phosphatase
ECNIH5_12835
Virulence factor VirK
ECNIH5_12845
membrane protein
ECNIH5_12860
ECNIH5_12865
exodeoxyribonuclease X
Enterobacter cloacae strain ECNIH5
tail fiber assembly protein
AU41_06675
type III secretion protein SopE2
AU41_06680
AU41_06685
hypothetical protein
AU41_06695
integrase
AU41_06700
exodeoxyribonuclease VIII
AU41_06710
hypothetical protein
AU41_06715
lytic enzyme
Salmonella enterica subsp. enterica serovar Enteritidis str. EC20090884
AU41_06520
exodeoxyribonuclease X
AU41_06525
AU41_06540
membrane protein
AU41_06555
integrase
AU41_06560
excisionase
AU41_06565
hypothetical protein
Salmonella enterica subsp. enterica serovar Enteritidis str. EC20090884
EcDH1_1798
EcDH1_1799
EcDH1_1800
EcDH1_1802
EcDH1_1804
EcDH1_1807
RNA methylase, NOL1/NOP2/sun family
EcDH1_1808
Mammalian cell entry related domain protein
Escherichia coli DH1
AU79_05925
tail fiber assembly protein
AU79_05930
type III secretion protein SopE2
AU79_05935
DNA invertase
AU79_05940
hypothetical protein
AU79_05950
integrase
AU79_05955
exodeoxyribonuclease VIII
AU79_05965
hypothetical protein
AU79_05970
lytic enzyme
Salmonella enterica subsp. enterica serovar Enteritidis str. SA19940857 genome−p.c.VAL54.69%−taxID1412501

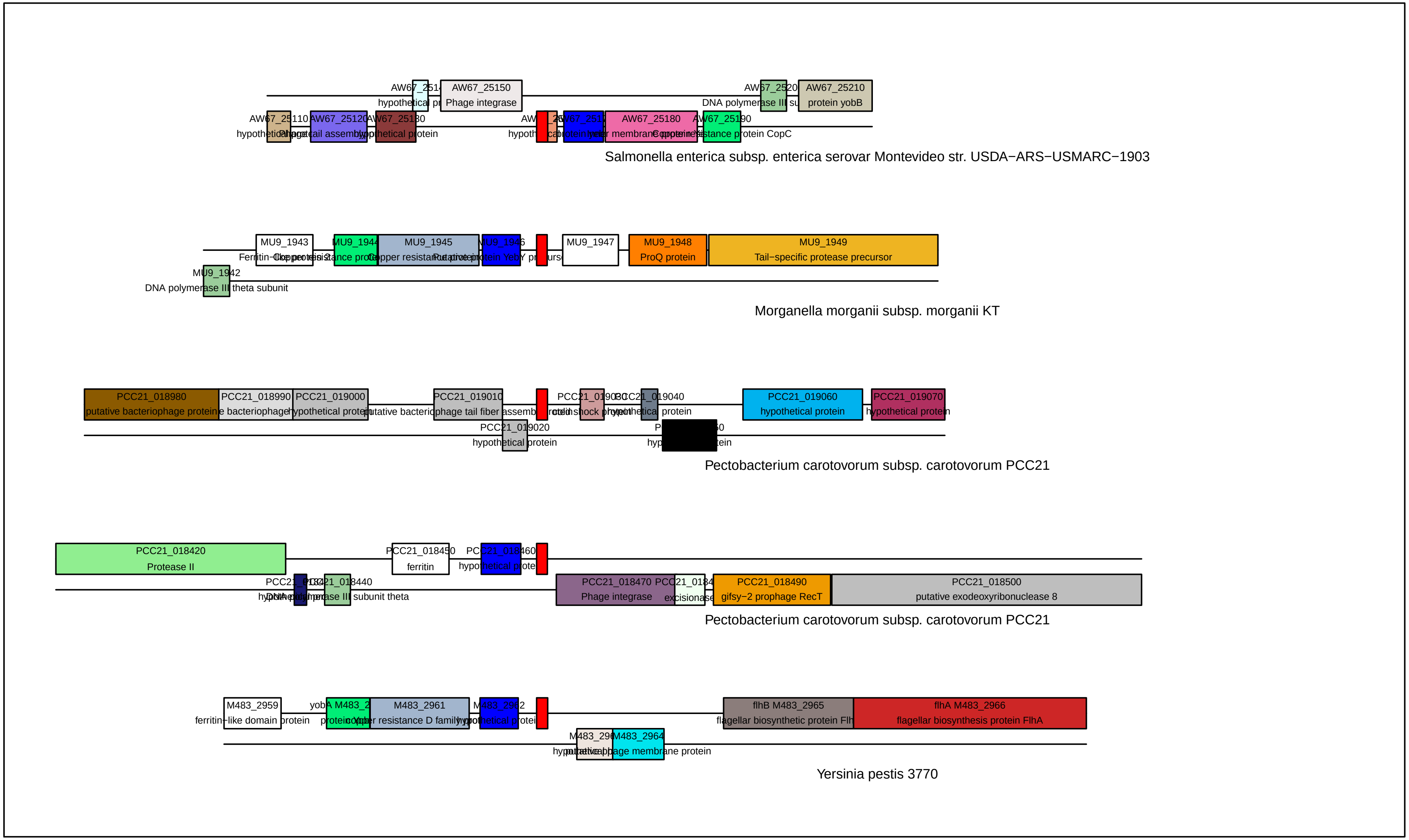

AW67_25150
Phage integrase
AW67_25210
protein yobB
AW67_25120
AW67_25180
Salmonella enterica subsp. enterica serovar Montevideo str. USDA−ARS−USMARC−1903
MU9_1943
MU9_1945
MU9_1947
MU9_1948
ProQ protein
MU9_1949
Tail−specific protease precursor
MU9_1942
DNA polymerase III theta subunit
Morganella morganii subsp. morganii KT
PCC21_018980
putative bacteriophage protein
PCC21_018990
PCC21_019000
PCC21_019010
PCC21_019040
PCC21_019060
hypothetical protein
PCC21_019070
hypothetical protein
PCC21_019020
hypothetical protein
Pectobacterium carotovorum subsp. carotovorum PCC21
PCC21_018420
Protease II
PCC21_018450
ferritin
PCC21_018460
PCC21_018440
PCC21_018470
Phage integrase
PCC21_018490
gifsy−2 prophage RecT
PCC21_018500
putative exodeoxyribonuclease 8
Pectobacterium carotovorum subsp. carotovorum PCC21
M483_2959
ferritin−like domain protein
M483_2961
M483_2962
flhB M483_2965
flhA M483_2966
flagellar biosynthesis protein FlhA
M483_2964
Yersinia pestis 3770

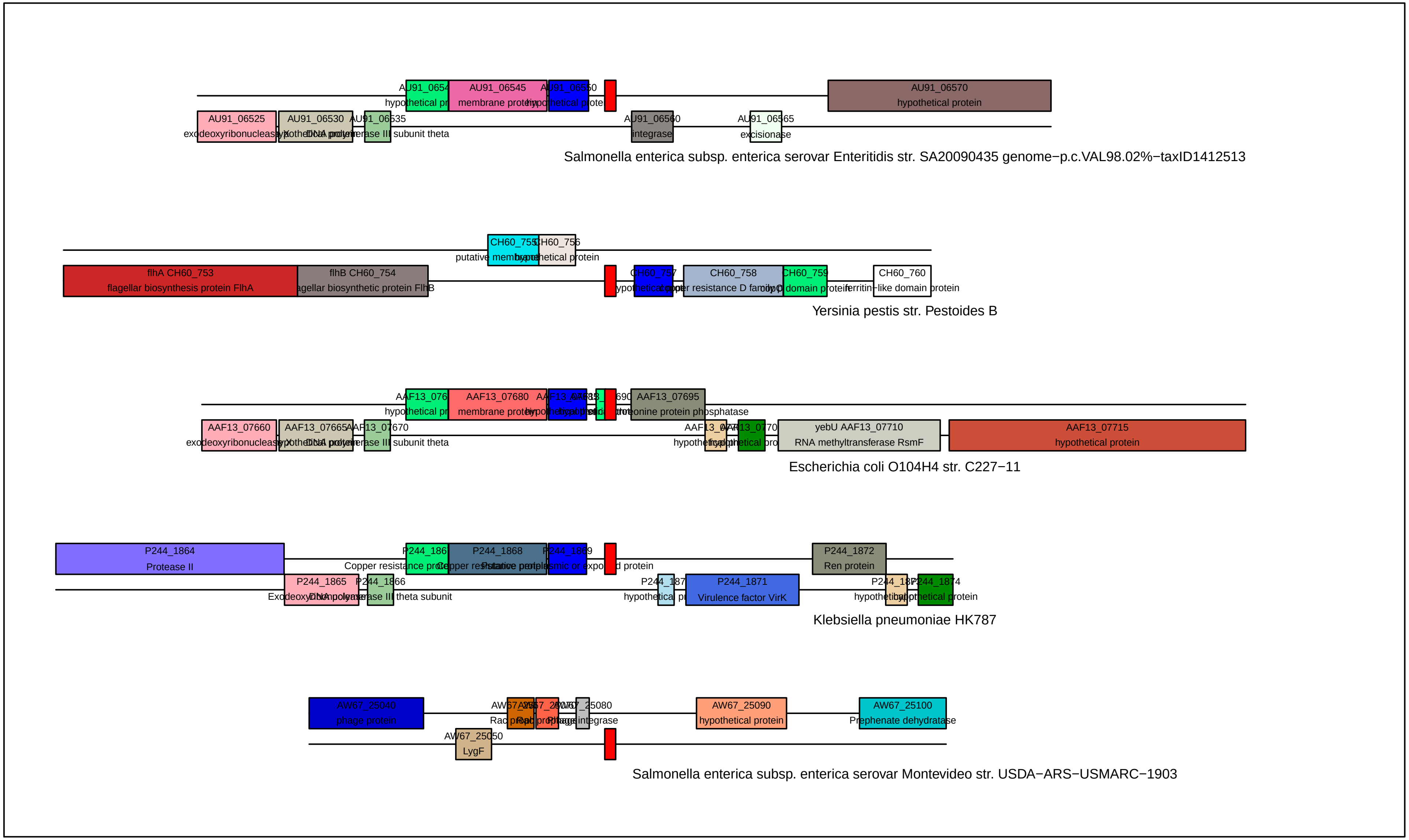
AU91_06545
membrane protein
AU91_06570
hypothetical protein
AU91_06525
AU91_06530
AU91_06560
integrase
AU91_06565
excisionase
Salmonella enterica subsp. enterica serovar Enteritidis str. SA20090435 genome−p.c.VAL98.02%−taxID1412513
CH60_756
flhA CH60_753
flagellar biosynthesis protein FlhA
flhB CH60_754
CH60_758
CH60_760
Yersinia pestis str. Pestoides B
AAF13_07680
AAF13_07695
AAF13_07660
AAF13_07665
AAF13_07670
yebU AAF13_07710
RNA methyltransferase RsmF
AAF13_07715
hypothetical protein
Escherichia coli O104H4 str. C227−11
P244_1864
Protease II
P244_1868
P244_1869
P244_1872
Ren protein
P244_1865
P244_1866
P244_1871
Virulence factor VirK
Klebsiella pneumoniae HK787
AW67_25040
phage protein
AW67_25090
hypothetical protein
AW67_25100
Prephenate dehydratase
AW67_25050
LygF
Salmonella enterica subsp. enterica serovar Montevideo str. USDA−ARS−USMARC−1903

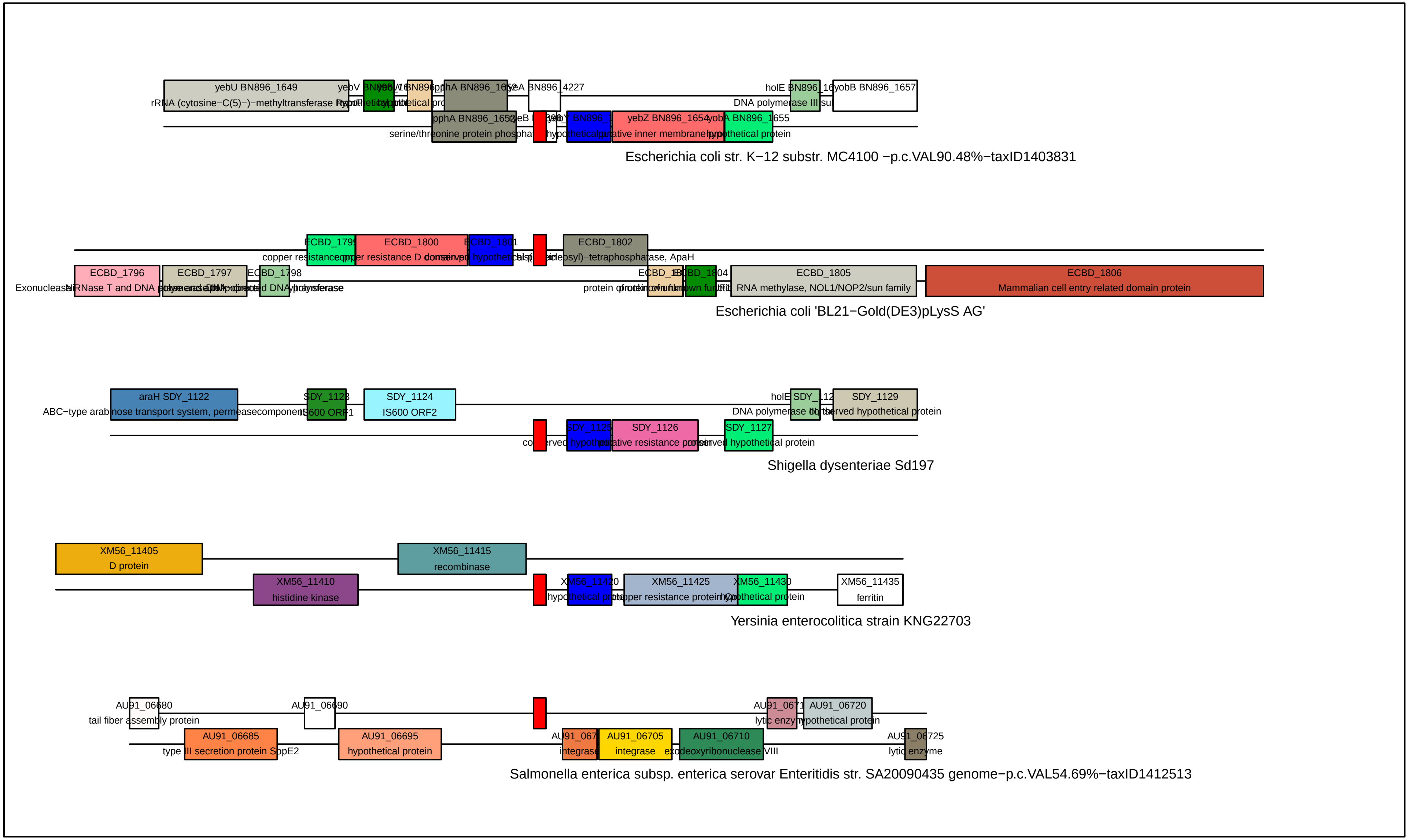
yebU BN896_1649
rRNA (cytosine−C(5)−)−methyltransferase
BN896_4227
holE
yobB BN896_1657
DNA polymerase III
pphA BN896_1652
serine/threonine protein phospha
yebZ BN896_1654
yobA BN896_1655
hypothetical protein
Escherichia coli str. K−12 substr. MC4100 −p.c.VAL90.48%−taxID1403831
ECBD_1800
ECBD_1802
copper resistance D domain
ECBD_1796
ECBD_1797
ECBD_1798
ECBD_1805
ECBD_1806
RNA methylase, NOL1/NOP2/sun family
Mammalian cell entry related domain protein
Escherichia coli 'BL21−Gold(DE3)pLysS AG'
araH SDY_1122
ABC−type arabinose transport system, permease component
SDY_1124
IS600 ORF2
SDY_1129
SDY_1126
SDY_1127
Shigella dysenteriae Sd197
XM56_11405
D protein
XM56_11415
recombinase
XM56_11410
histidine kinase
XM56_11425
XM56_11430
XM56_11435
ferritin
Yersinia enterocolitica strain KNG22703
tail fiber assembly protein
AU91_06685
AU91_06695
hypothetical protein
AU91_06720
AU91_06705
integrase
AU91_06710
lytic enzyme
Salmonella enterica subsp. enterica serovar Enteritidis str. SA20090435 genome−p.c.VAL54.69%−taxID1412513

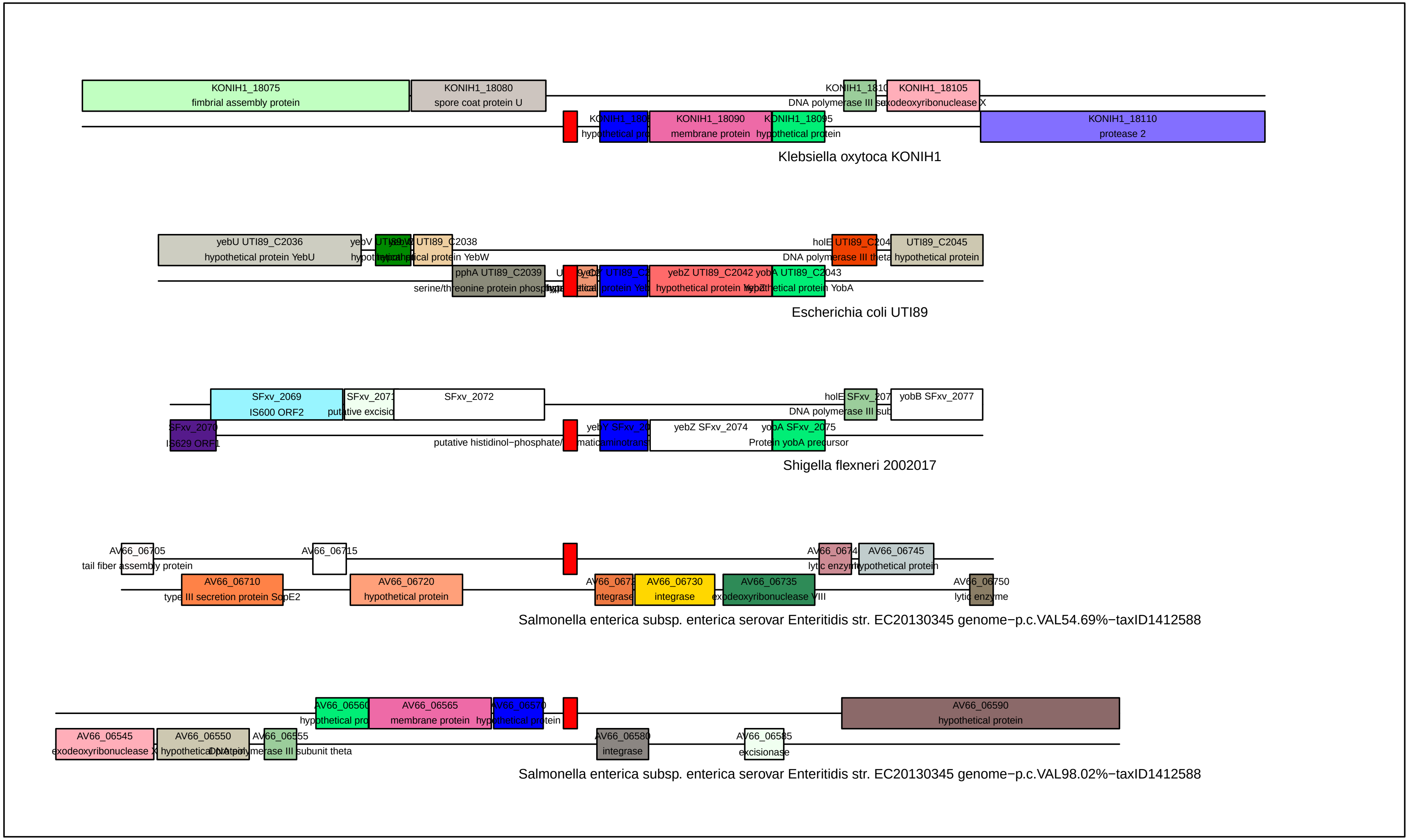
KONIH1_18075
fimbrial assembly protein
KONIH1_18080
spore coat protein U
KONIH1_18105
exodeoxyribonuclease X
KONIH1_18090
membrane protein
KONIH1_18095
hypothetical protein
KONIH1_18110
protease 2
Klebsiella oxytoca KONIH1
yebU UTI89_C2036
hypothetical protein YebU
UTI89_C2038
hypothetical protein YebW
UTI89_C2045
hypothetical protein
pphA UTI89_C2039
serine/threonine protein phosphatase
yebZ UTI89_C2042
hypothetical protein YebZ
yobA UTI89_C2043
hypothetical protein YobA
DNA polymerase III theta
Escherichia coli UTI89
SFxv_2069
IS600 ORF2
SFxv_2072
yobB SFxv_2077
SFxv_2070
IS629 ORF1
putative histidinol−phosphate/aromatic aminotransferase
yebZ SFxv_2074
yobA SFxv_2075
Protein yobA precursor
Shigella flexneri 2002017
AV66_06705
tail fiber assembly protein
AV66_06715
AV66_06745
hypothetical protein
AV66_06710
type III secretion protein SopE2
AV66_06720
hypothetical protein
AV66_06730
integrase
AV66_06735
exodeoxyribonuclease VIII
AV66_06750
lytic enzyme
Salmonella enterica subsp. enterica serovar Enteritidis str. EC20130345 genome−p.c.VAL54.69%−taxID1412588
AV66_06565
membrane protein
AV66_06590
hypothetical protein
AV66_06545
exodeoxyribonuclease X
AV66_06550
AV66_06555
DNA polymerase III subunit theta
AV66_06580
integrase
AV66_06585
excisionase
Salmonella enterica subsp. enterica serovar Enteritidis str. EC20130345 genome−p.c.VAL98.02%−taxID1412588

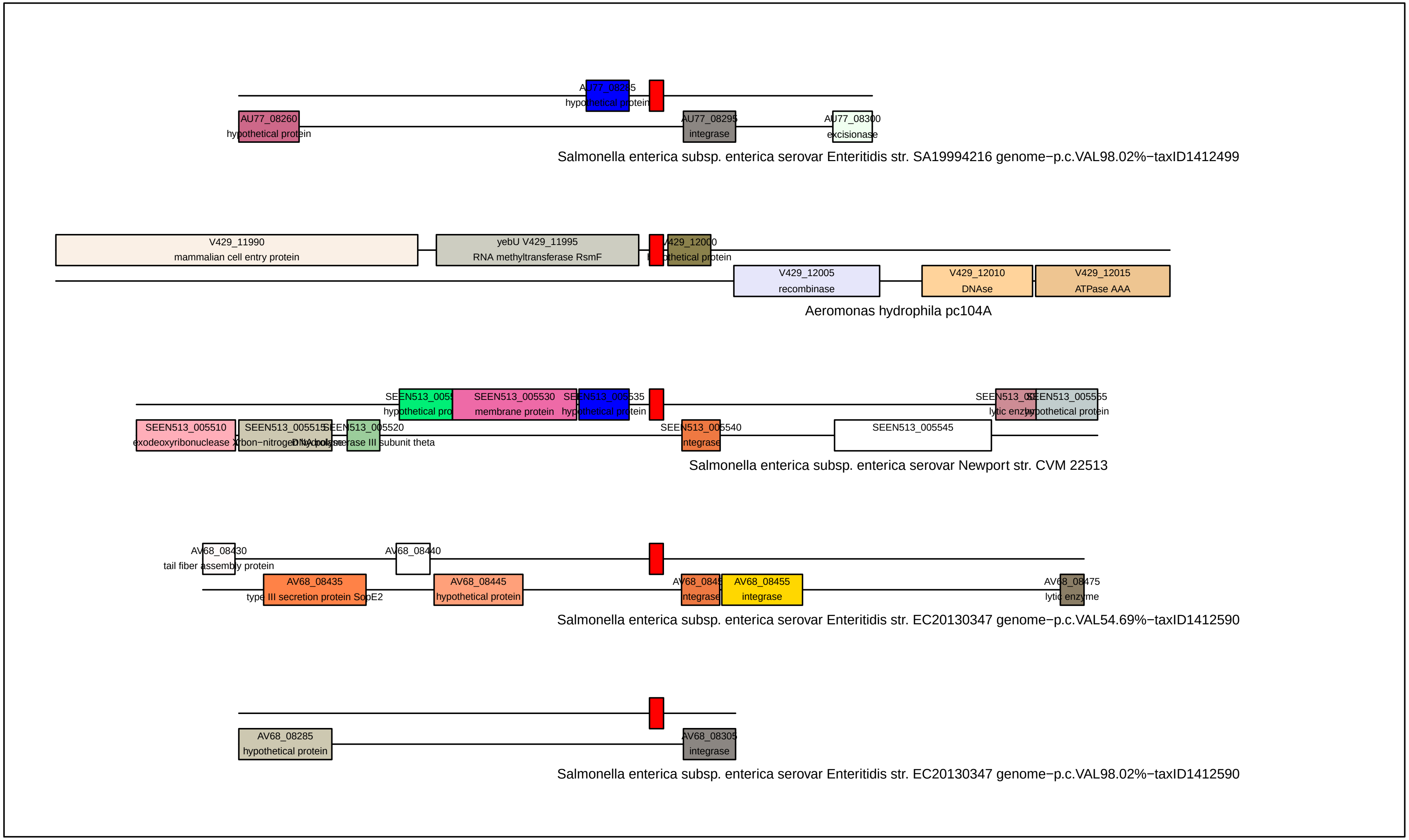

AU77_08285
hypothetical protein
AU77_08260
hypothetical protein
AU77_08295
integrase
AU77_08300
excisionase
Salmonella enterica subsp. enterica serovar Enteritidis str. SA19994216 genome−p.c.VAL98.02%−taxID1412499
V429_11990
mammalian cell entry protein
yebU V429_11995
RNA methyltransferase RsmF
V429_12000
hypothetical protein
V429_12005
recombinase
V429_12010
DNAse
V429_12015
ATPase AAA
Aeromonas hydrophila pc104A
SEEN513_005530
membrane protein
SEEN513_005535
hypothetical protein
SEEN513_005555
hypothetical protein
SEEN513_005510
exodeoxyribonuclease X
SEEN513_005515
SEEN513_005520
DNA polymerase III subunit theta
SEEN513_005540
integrase
SEEN513_005545
Salmonella enterica subsp. enterica serovar Newport str. CVM 22513
AV68_08430
tail fiber assembly protein
AV68_08440
AV68_08435
type III secretion protein SopE2
AV68_08445
hypothetical protein
integrase
AV68_08455
integrase
AV68_08475
lytic enzyme
Salmonella enterica subsp. enterica serovar Enteritidis str. EC20130347 genome−p.c.VAL54.69%−taxID1412590
AV68_08285
hypothetical protein
AV68_08305
integrase
Salmonella enterica subsp. enterica serovar Enteritidis str. EC20130347 genome−p.c.VAL98.02%−taxID1412590

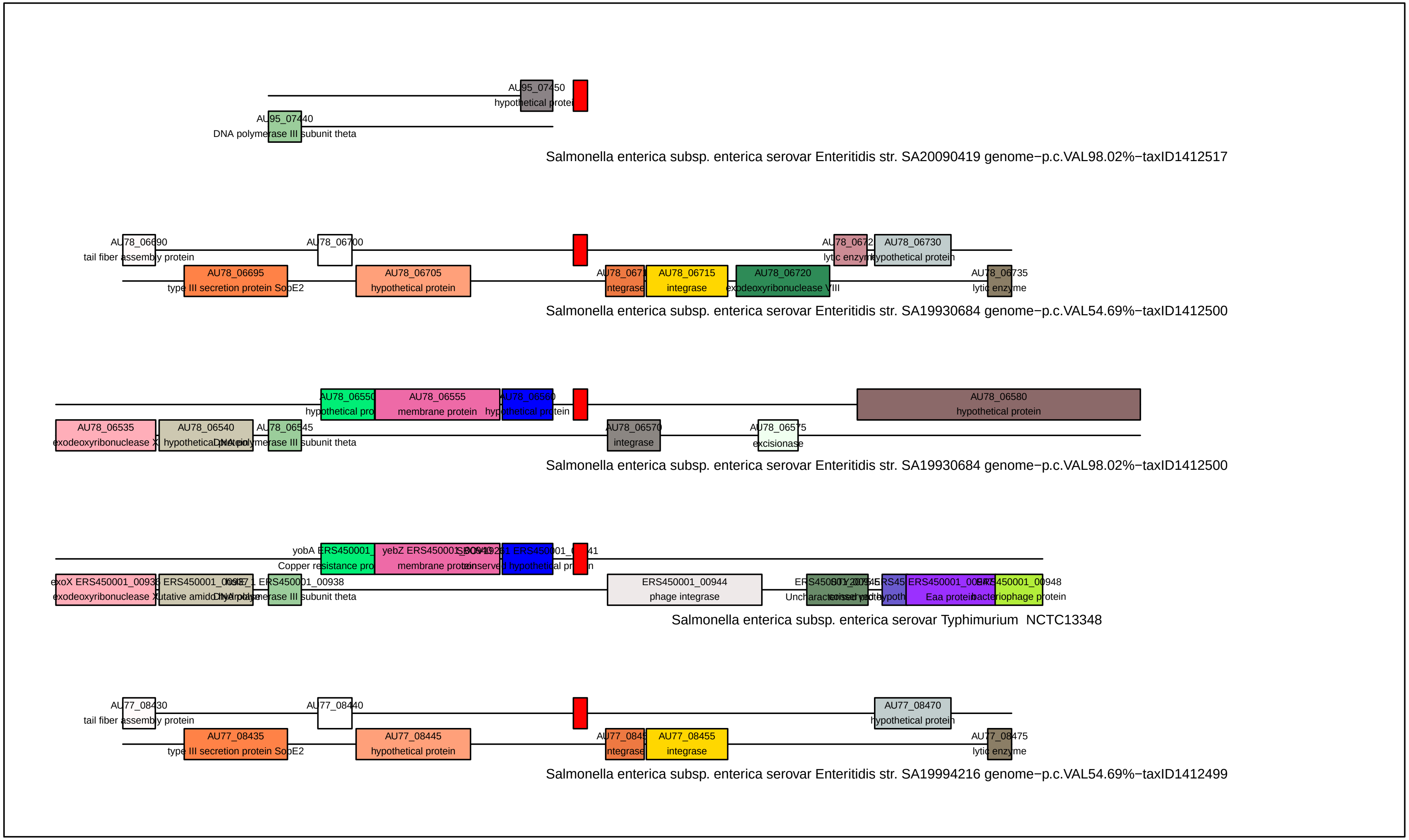

AU95_07450
AU95_07440
DNA polymerase III subunit theta
Salmonella enterica subsp. enterica serovar Enteritidis str. SA20090419 genome−p.c.VAL98.02%−taxID1412517
AU78_06690
tail fiber assembly protein
AU78_06700
AU78_06730
hypothetical protein
AU78_06695
type III secretion protein SopE2
AU78_06705
hypothetical protein
integrase
AU78_06715
integrase
AU78_06720
exodeoxyribonuclease VIII
AU78_06735
lytic enzyme
Salmonella enterica subsp. enterica serovar Enteritidis str. SA19930684 genome−p.c.VAL54.69%−taxID1412500
AU78_06555
membrane protein
AU78_06580
hypothetical protein
AU78_06535
exodeoxyribonuclease X
AU78_06540
AU78_06545
subunit theta
AU78_06570
integrase
AU78_06575
excisionase
Salmonella enterica subsp. enterica serovar Enteritidis str. SA19930684 genome−p.c.VAL98.02%−taxID1412500
yobA
membrane protein
exoX ERS450001_00936
exodeoxyribonuclease X
ERS450001_00938
subunit theta
ERS450001_00944
phage integrase
Eaa protein
ERS450001_00948
bacteriophage protein
Salmonella enterica subsp. enterica serovar Typhimurium NCTC13348
AU77_08430
tail fiber assembly protein
AU77_08440
AU77_08470
hypothetical protein
AU77_08435
type III secretion protein SopE2
AU77_08445
hypothetical protein
integrase
AU77_08455
integrase
AU77_08475
lytic enzyme
Salmonella enterica subsp. enterica serovar Enteritidis str. SA19994216 genome−p.c.VAL54.69%−taxID1412499

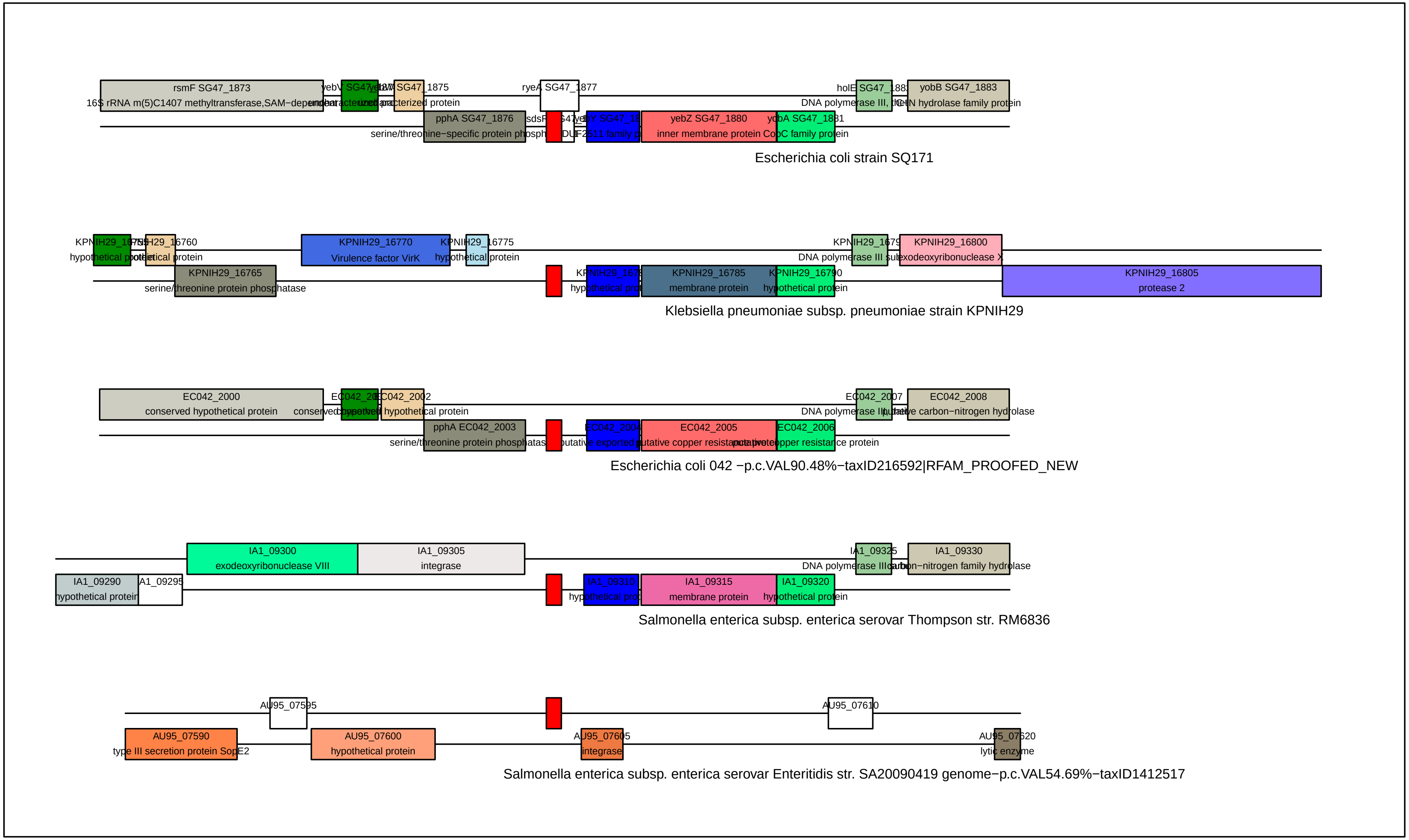
rsmF SG47_1873
yobB SG47_1883
pphA SG47_1876
yebZ SG47_1880
inner membrane protein CopC family protein
Escherichia coli strain SQ171
KPNIH29_16770
Virulence factor VirK
KPNIH29_16800
KPNIH29_16765
serine/threonine protein phosphatase
KPNIH29_16785
membrane protein
KPNIH29_16805
protease 2
Klebsiella pneumoniae subsp. pneumoniae strain KPNIH29
EC042_2000
conserved hypothetical protein
EC042_2008
pphA EC042_2003
EC042_2005
Escherichia coli 042 −p.c.VAL90.48%−taxID216592|RFAM_PROOFED_NEW
IA1_09300
exodeoxyribonuclease VIII
IA1_09305
integrase
IA1_09330
IA1_09290
IA1_09315
membrane protein
Salmonella enterica subsp. enterica serovar Thompson str. RM6836
AU95_07590
AU95_07600
hypothetical protein
AU95_07605
integrase
Salmonella enterica subsp. enterica serovar Enteritidis str. SA20090419 genome−p.c.VAL54.69%−taxID1412517

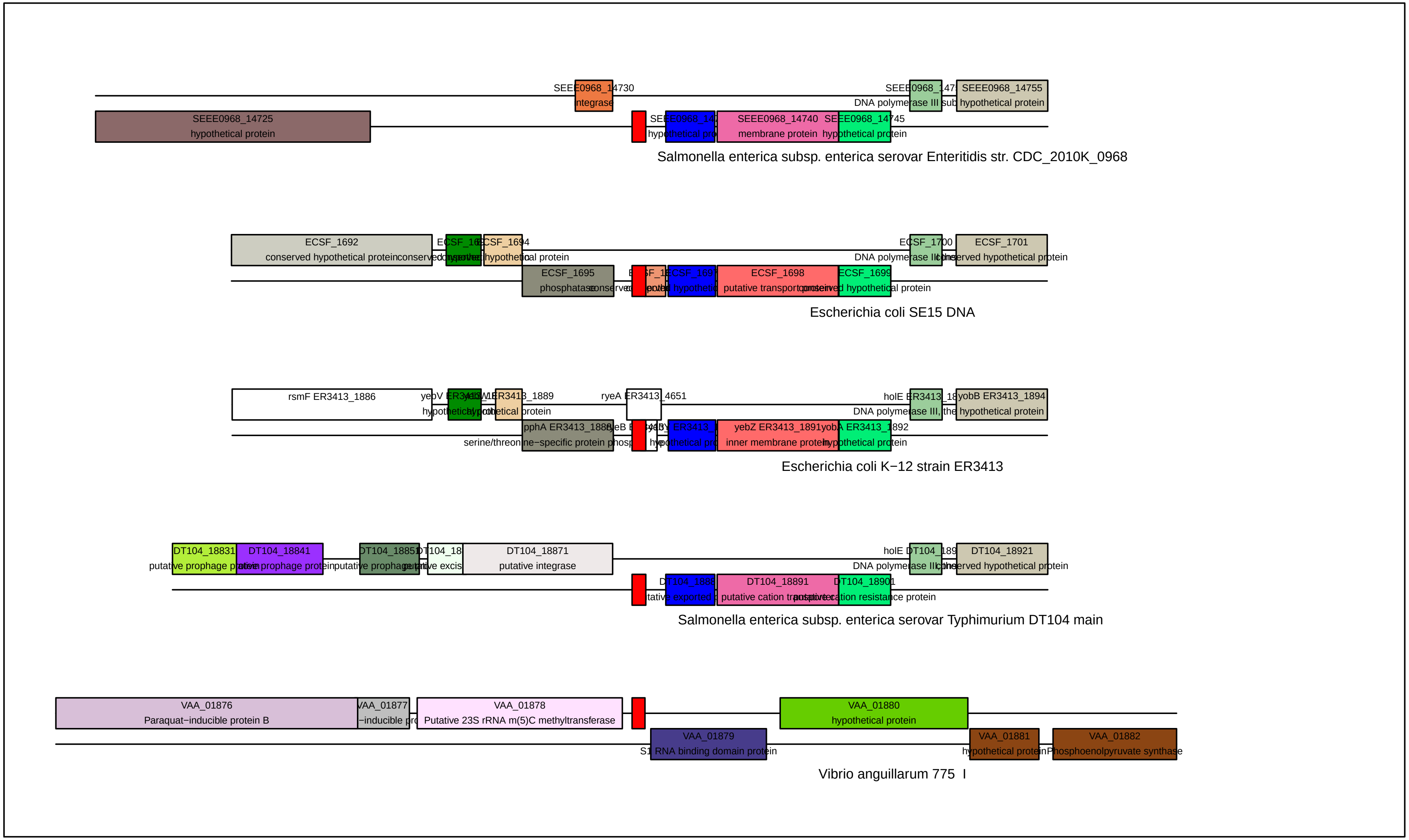

SEEE0968_14730
integrase
SEEE0968_14755
hypothetical protein
SEEE0968_14725
hypothetical protein
SEEE0968_14740
membrane protein
SEEE0968_14745
hypothetical protein
Salmonella enterica subsp. enterica serovar Enteritidis str. CDC_2010K_0968
ECSF_1692
conserved hypothetical protein
ECSF_1694
ECSF_1700
ECSF_1701
conserved hypothetical protein
ECSF_1695
phosphatase
ECSF_1698
putative transport protein
ECSF_1699
Escherichia coli SE15 DNA
rsmF ER3413_1886
ER3413_1889
hypothetical protein
ryeA ER3413_4651
yobB ER3413_1894
hypothetical protein
pphA ER3413_1888
serine/threonine−specific protein phosp
yebZ ER3413_1891
inner membrane protein
yobA ER3413_1892
hypothetical protein
Escherichia coli K−12 strain ER3413
DT104_18831
DT104_18841
putative prophage protein
DT104_18871
putative integrase
DT104_18921
conserved hypothetical protein
DT104_18891
DT104_18901
putative cation resistance protein
Salmonella enterica subsp. enterica serovar Typhimurium DT104 main
VAA_01876
Paraquat−inducible protein B
VAA_01877
VAA_01878
Putative 23S rRNA m(5)C methyltransferase
VAA_01880
hypothetical protein
VAA_01879
S1 RNA binding domain protein
VAA_01881
hypothetical protein
VAA_01882
Phosphoenolpyruvate synthase
Vibrio anguillarum 775 I

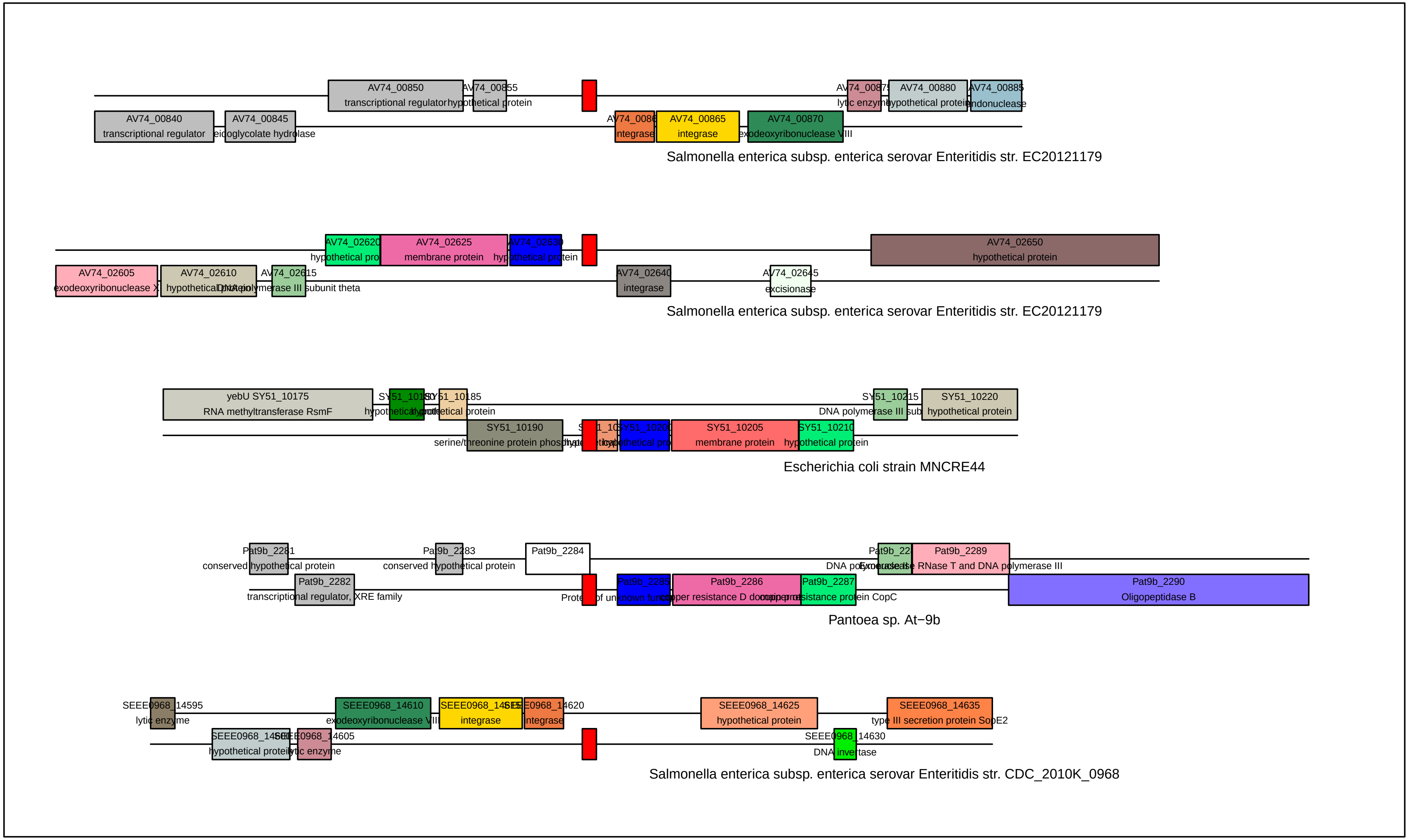
AV74_00850
transcriptional regulator
AV74_00855
hypothetical protein
AV74_00840
transcriptional regulator
AV74_00845
integrase
AV74_00865
integrase
AV74_00870
exodeoxyribonuclease VIII
lytic enzyme
AV74_00880
hypothetical protein
AV74_00885
endonuclease
Salmonella enterica subsp. enterica serovar Enteritidis str. EC20121179
AV74_02620
AV74_02625
membrane protein
AV74_02630
hypothetical protein
AV74_02650
hypothetical protein
AV74_02605
exodeoxyribonuclease X
AV74_02610
AV74_02615
DNA polymerase III subunit theta
AV74_02640
integrase
AV74_02645
excisionase
Salmonella enterica subsp. enterica serovar Enteritidis str. EC20121179
yebU SY51_10175
RNA methyltransferase RsmF
SY51_10185
SY51_10215
SY51_10220
hypothetical protein
SY51_10190
serine/threonine protein phosphatase
SY51_10205
membrane protein
SY51_10210
hypothetical protein
Escherichia coli strain MNCRE44
Pat9b_2281
conserved hypothetical protein
Pat9b_2283
conserved hypothetical protein
Pat9b_2284
Pat9b_2289
RNase T and DNA polymerase III
Pat9b_2282
transcriptional regulator, XRE family
Pat9b_2286
Pat9b_2287
copper resistance protein CopC
Pat9b_2290
Oligopeptidase B
Pantoea sp. At−9b
SEEE0968_14595
lytic enzyme
SEEE0968_14610
exodeoxyribonuclease VIII
integrase
SEEE0968_14620
integrase
SEEE0968_14625
hypothetical protein
SEEE0968_14635
type III secretion protein SopE2
SEEE0968_14605
lytic enzyme
SEEE0968_14630
DNA invertase
Salmonella enterica subsp. enterica serovar Enteritidis str. CDC_2010K_0968

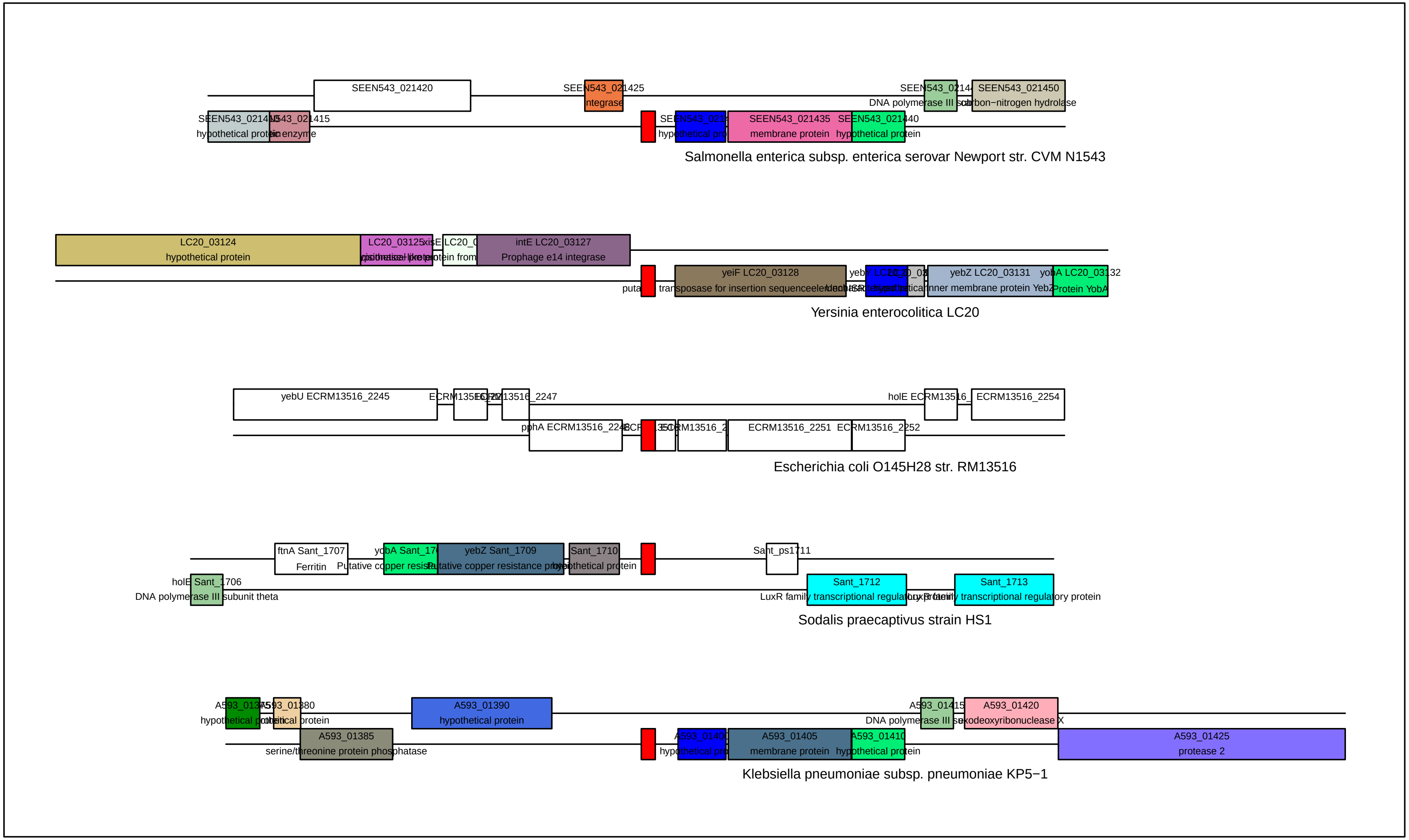

SEEN543_021420
SEEN543_021425
integrase
SEEN543_021450
carbon−nitrogen hydrolase
SEEN543_021435
membrane protein
SEEN543_021440
hypothetical protein
Salmonella enterica subsp. enterica serovar Newport str. CVM N1543
LC20_03124
hypothetical protein
intE LC20_03127
Prophage e14 integrase
yeiF LC20_03128
yebZ LC20_03131
Inner membrane protein YebZ
Protein YobA
Yersinia enterocolitica LC20
yebU ECRM13516_2245
ECRM13516_2254
ECRM13516_2251
ECRM13516_2252
Escherichia coli O145H28 str. RM13516
ftnA Sant_1707
Ferritin
yebZ Sant_1709
Sant_1710
Sant_ps1711
Sant_1712
Sant_1713
holE Sant_1706
DNA polymerase III subunit theta
Sodalis praecaptivus strain HS1
A593_01390
hypothetical protein
A593_01420
A593_01385
serine/threonine protein phosphatase
A593_01405
membrane protein
A593_01425
protease 2
Klebsiella pneumoniae subsp. pneumoniae KP5−1

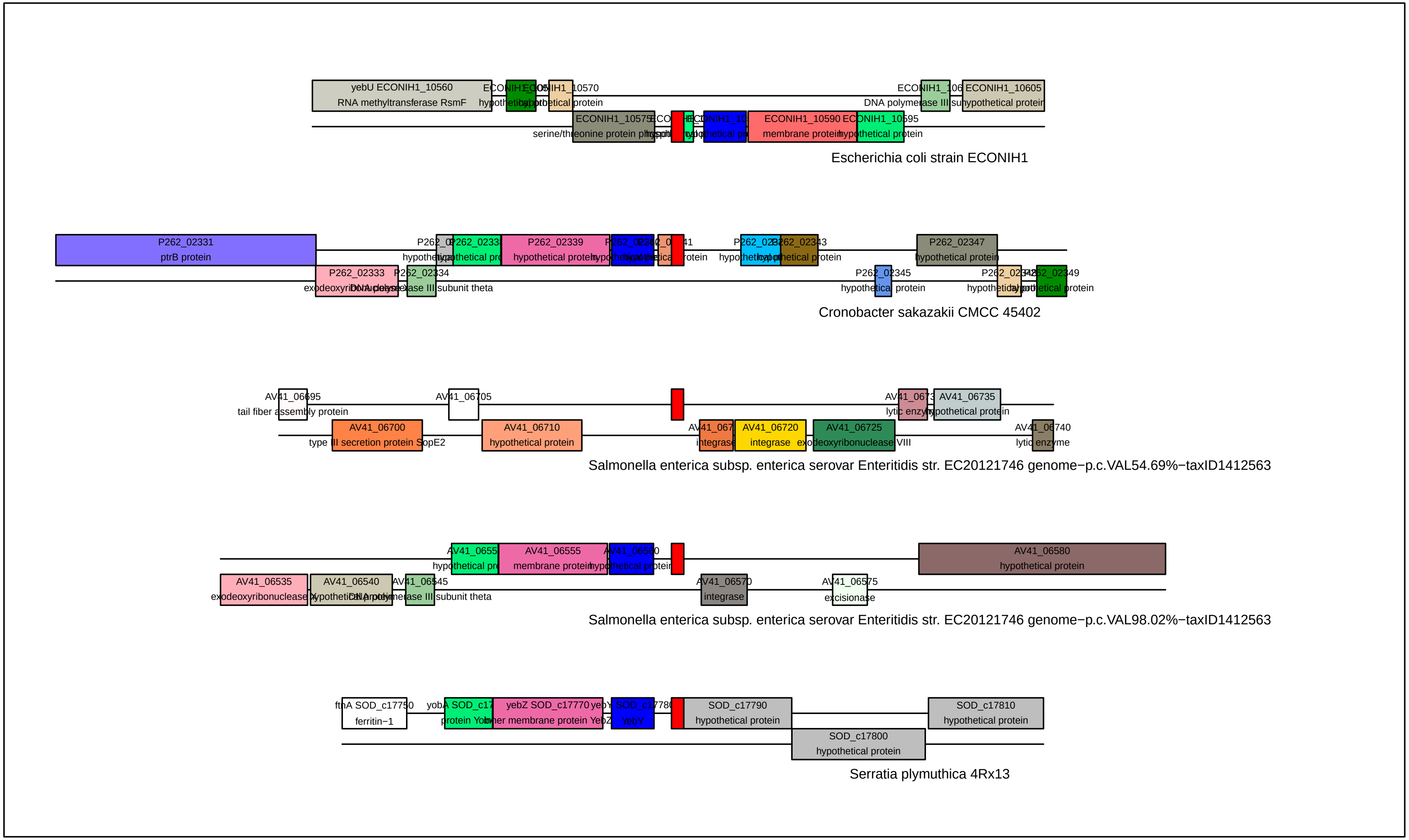
yebU ECONIH1_10560
RNA methyltransferase RsmF
ECONIH1_10605
hypothetical protein
ECONIH1_10575
ECONIH1_10590
membrane protein
ECONIH1_10595
hypothetical protein
Escherichia coli strain ECONIH1
P262_02331
ptrB protein
P262_02339
hypothetical protein
P262_02347
hypothetical protein
P262_02333
P262_02334
subunit theta
P262_02345
hypothetical protein
Cronobacter sakazakii CMCC 45402
AV41_06695
tail fiber assembly protein
AV41_06705
AV41_06735
hypothetical protein
AV41_06700
type III secretion protein SopE2
AV41_06710
hypothetical protein
AV41_06720
integrase
AV41_06725
exodeoxyribonuclease VIII
AV41_06740
lytic enzyme
Salmonella enterica subsp. enterica serovar Enteritidis str. EC20121746 genome−p.c.VAL54.69%−taxID1412563
AV41_06555
membrane protein
AV41_06580
hypothetical protein
AV41_06535
AV41_06540
AV41_06545
subunit theta
AV41_06570
integrase
AV41_06575
excisionase
Salmonella enterica subsp. enterica serovar Enteritidis str. EC20121746 genome−p.c.VAL98.02%−taxID1412563
ftnA SOD_c17750
ferritin−1
yebZ SOD_c17770
SOD_c17790
hypothetical protein
SOD_c17810
hypothetical protein
SOD_c17800
hypothetical protein
Serratia plymuthica 4Rx13

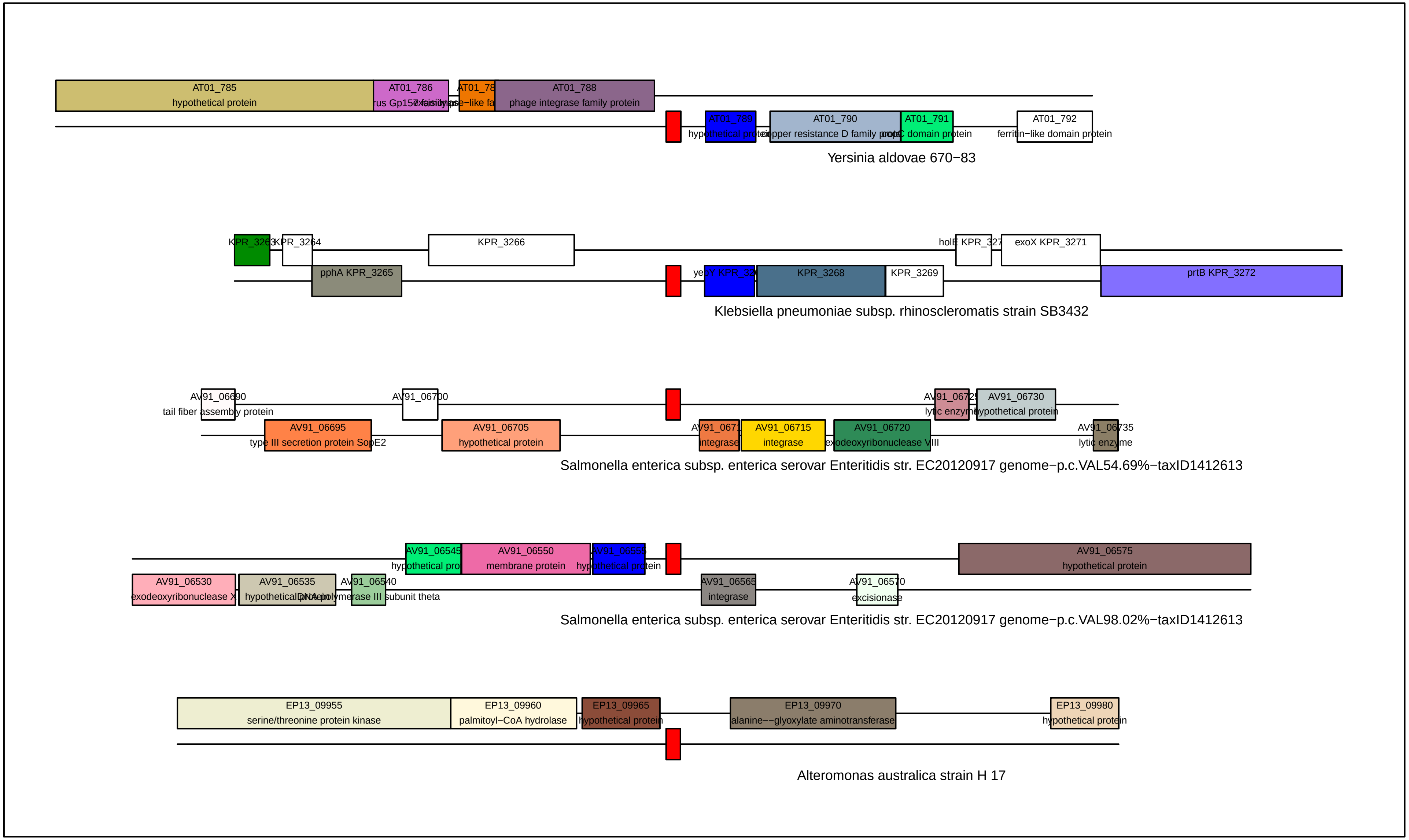
AT01_785
hypothetical protein
AT01_786
AT01_788
phage integrase family protein
AT01_789
AT01_790
copper resistance D family protein
AT01_791
AT01_792
ferritin−like domain protein
Yersinia aldovae 670−83
KPR_3264
KPR_3266
exoX KPR_3271
pphA KPR_3265
KPR_3268
KPR_3269
prtB KPR_3272
Klebsiella pneumoniae subsp. rhinoscleromatis strain SB3432
AV91_06690
tail fiber assembly protein
AV91_06700
AV91_06730
hypothetical protein
AV91_06695
type III secretion protein SopE2
AV91_06705
hypothetical protein
integrase
AV91_06715
integrase
AV91_06720
exodeoxyribonuclease VIII
AV91_06735
lytic enzyme
Salmonella enterica subsp. enterica serovar Enteritidis str. EC20120917 genome−p.c.VAL54.69%−taxID1412613
AV91_06545
AV91_06550
membrane protein
AV91_06575
hypothetical protein
AV91_06530
exodeoxyribonuclease X
AV91_06535
AV91_06540
AV91_06565
integrase
AV91_06570
excisionase
Salmonella enterica subsp. enterica serovar Enteritidis str. EC20120917 genome−p.c.VAL98.02%−taxID1412613
EP13_09955
serine/threonine protein kinase
EP13_09960
palmitoyl−CoA hydrolase
EP13_09965
hypothetical protein
EP13_09970
alanine−−glyoxylate aminotransferase
EP13_09980
hypothetical protein
Alteromonas australica strain H 17

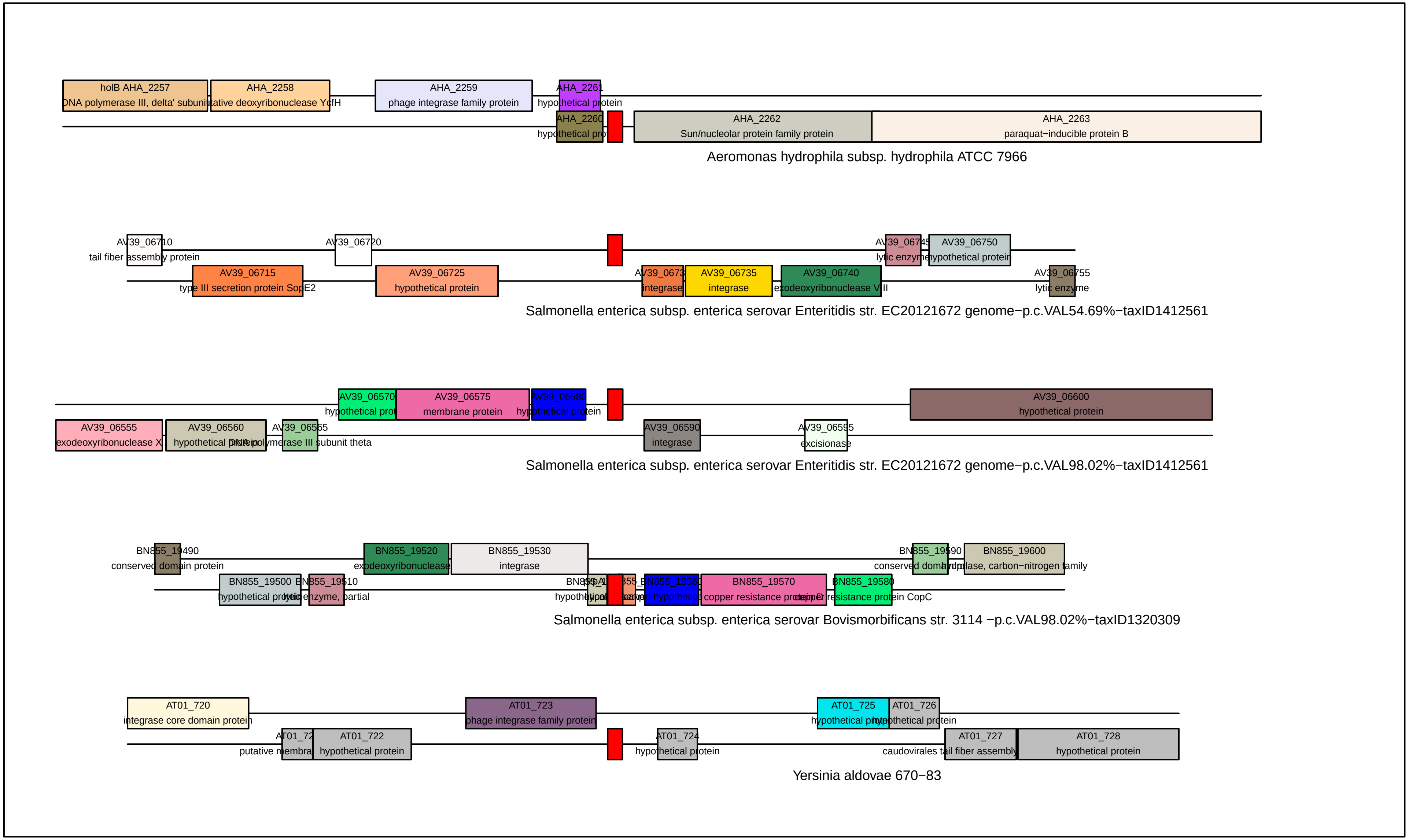

holB AHA_2257
AHA_2258
AHA_2259
phage integrase family protein
AHA_2261
AHA_2260
AHA_2262
Sun/nucleolar protein family protein
AHA_2263
paraquat−inducible protein B
Aeromonas hydrophila subsp. hydrophila ATCC 7966
tail fiber assembly protein
AV39_06715
AV39_06725
hypothetical protein
AV39_06735
integrase
AV39_06740
AV39_06750
AV39_06755
lytic enzyme
Salmonella enterica subsp. enterica serovar Enteritidis str. EC20121672 genome−p.c.VAL54.69%−taxID1412561
AV39_06570
AV39_06575
membrane protein
AV39_06600
hypothetical protein
AV39_06555
AV39_06560
AV39_06565
subunit theta
AV39_06590
integrase
AV39_06595
excisionase
Salmonella enterica subsp. enterica serovar Enteritidis str. EC20121672 genome−p.c.VAL98.02%−taxID1412561
BN855_19490
conserved domain protein
BN855_19520
BN855_19530
integrase
BN855_19600
BN855_19500
BN855_19510
lytic enzyme, partial
BN855_19570
BN855_19580
Salmonella enterica subsp. enterica serovar Bovismorbificans str. 3114 −p.c.VAL98.02%−taxID1320309
AT01_720
integrase core domain protein
AT01_723
phage integrase family protein
AT01_725
AT01_726
hypothetical protein
AT01_722
hypothetical protein
AT01_724
hypothetical protein
AT01_727
AT01_728
hypothetical protein
Yersinia aldovae 670−83

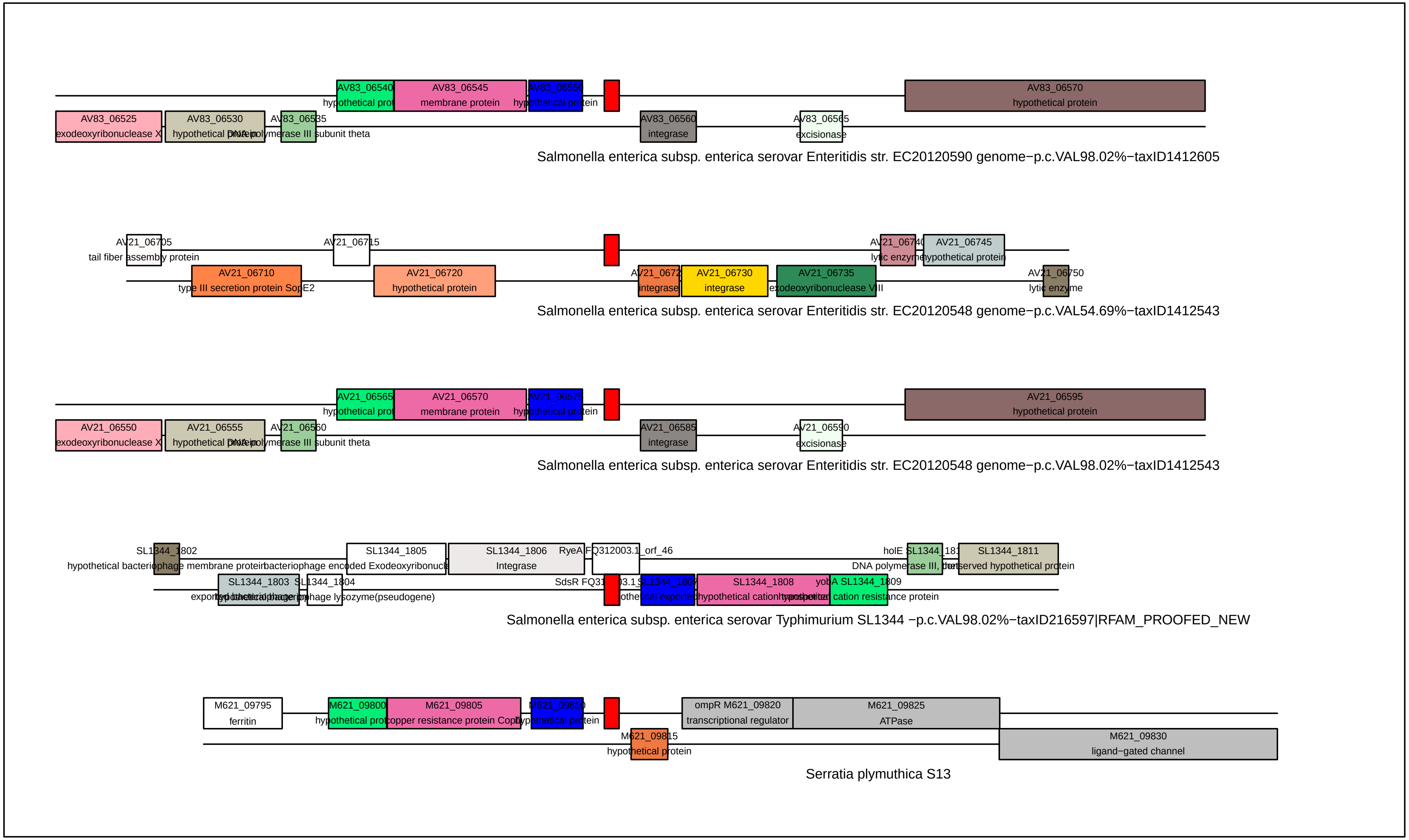

AV83_06540
AV83_06545
membrane protein
AV83_06570
hypothetical protein
AV83_06525
exodeoxyribonuclease X
AV83_06530
AV83_06535
DNA polymerase III subunit theta
AV83_06560
integrase
AV83_06565
excisionase
Salmonella enterica subsp. enterica serovar Enteritidis str. EC20120590 genome−p.c.VAL98.02%−taxID1412605
AV21_06705
tail fiber assembly protein
AV21_06715
AV21_06745
hypothetical protein
AV21_06710
type III secretion protein SopE2
AV21_06720
hypothetical protein
integrase
AV21_06730
integrase
AV21_06735
exodeoxyribonuclease VIII
AV21_06750
lytic enzyme
Salmonella enterica subsp. enterica serovar Enteritidis str. EC20120548 genome−p.c.VAL54.69%−taxID1412543
AV21_06570
membrane protein
AV21_06595
hypothetical protein
AV21_06550
exodeoxyribonuclease X
AV21_06555
AV21_06560
DNA polymerase III subunit theta
AV21_06585
integrase
AV21_06590
excisionase
Salmonella enterica subsp. enterica serovar Enteritidis str. EC20120548 genome−p.c.VAL98.02%−taxID1412543
SL1344_1802
SL1344_1805
SL1344_1806
Integrase
RyeA
FQ312003.1_orf_46
holE
SL1344_1811
SL1344_1803
SL1344_1804
lysozyme(pseudogene)
SdsR
SL1344_1808
SL1344_1809
cation resistance protein
Salmonella enterica subsp. enterica serovar Typhimurium SL1344 −p.c.VAL98.02%−taxID216597|RFAM_PROOFED_NEW
M621_09795
ferritin
M621_09800
M621_09805
M621_09810
hypothetical protein
ompR M621_09820
transcriptional regulator
M621_09825
ATPase
M621_09815
hypothetical protein
M621_09830
ligand−gated channel
Serratia plymuthica S13

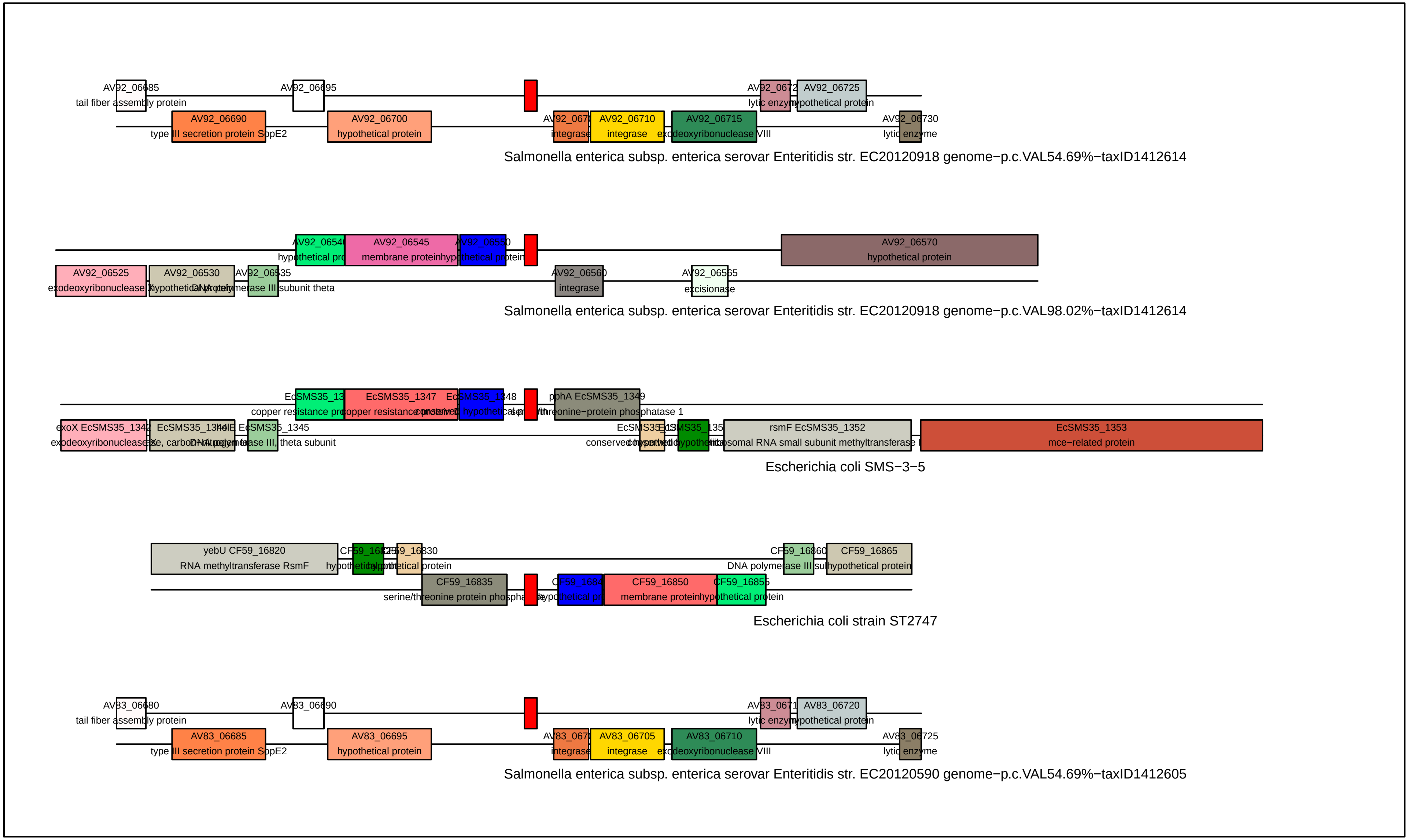

AV92_06685
tail fiber assembly protein
AV92_06695
AV92_06690
type III secretion protein SopE2
AV92_06700
hypothetical protein
AV92_06710
integrase
AV92_06715
exodeoxyribonuclease VIII
AV92_06725
hypothetical protein
AV92_06730
lytic enzyme
Salmonella enterica subsp. enterica serovar Enteritidis str. EC20120918 genome−p.c.VAL54.69%−taxID1412614
AV92_06545
membrane protein
AV92_06570
hypothetical protein
AV92_06525
exodeoxyribonuclease X
AV92_06530
AV92_06535
AV92_06560
integrase
AV92_06565
excisionase
Salmonella enterica subsp. enterica serovar Enteritidis str. EC20120918 genome−p.c.VAL98.02%−taxID1412614
EcSMS35_1347
pphA EcSMS35_1349
threonine−protein phosphatase 1
EcSMS35_1345
rsmF EcSMS35_1352
ribosomal RNA small subunit methyltransferase
EcSMS35_1353
mce−related protein
Escherichia coli SMS−3−5
yebU CF59_16820
RNA methyltransferase RsmF
CF59_16830
CF59_16865
hypothetical protein
CF59_16835
CF59_16850
membrane protein
CF59_16855
Escherichia coli strain ST2747
AV83_06680
tail fiber assembly protein
AV83_06690
AV83_06685
type III secretion protein SopE2
AV83_06695
hypothetical protein
AV83_06705
integrase
AV83_06710
exodeoxyribonuclease VIII
AV83_06720
hypothetical protein
AV83_06725
lytic enzyme
Salmonella enterica subsp. enterica serovar Enteritidis str. EC20120590 genome−p.c.VAL54.69%−taxID1412605

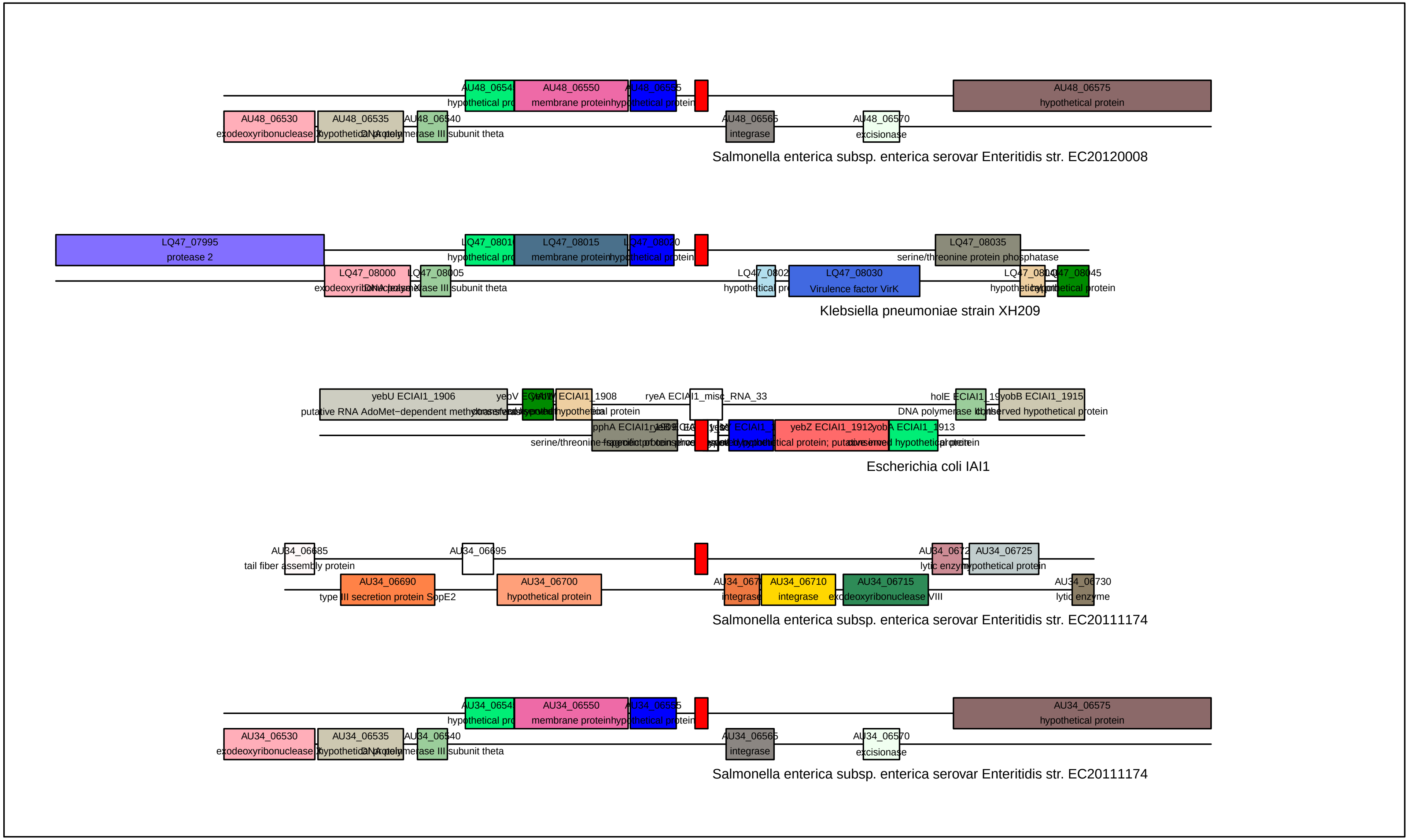

AU48_06550
membrane protein
AU48_06575
hypothetical protein
AU48_06530
exodeoxyribonuclease X
AU48_06535
AU48_06540
DNA polymerase III subunit theta
AU48_06565
integrase
AU48_06570
excisionase
Salmonella enterica subsp. enterica serovar Enteritidis str. EC20120008
LQ47_07995
protease 2
LQ47_08015
membrane protein
LQ47_08035
serine/threonine protein phosphatase
LQ47_08000
exodeoxyribonuclease X
LQ47_08005
DNA polymerase III subunit theta
LQ47_08030
Virulence factor VirK
hypothetical protein
Klebsiella pneumoniae strain XH209
yebU ECIAI1_1906
putative RNA AdoMet-dependent methyltransferase
ryeA ECIAI1_misc_RNA_33
yobB ECIAI1_1915
conserved hypothetical protein
DNA polymerase
yebZ ECIAI1_1912
Escherichia coli IAI1
AU34_06685
tail fiber assembly protein
AU34_06695
AU34_06725
AU34_06690
type III secretion protein SopE2
AU34_06700
hypothetical protein
AU34_06710
integrase
AU34_06715
exodeoxyribonuclease VIII
AU34_06730
lytic enzyme
Salmonella enterica subsp. enterica serovar Enteritidis str. EC20111174
AU34_06550
membrane protein
AU34_06575
hypothetical protein
AU34_06530
exodeoxyribonuclease X
AU34_06535
AU34_06540
DNA polymerase III subunit theta
AU34_06565
integrase
AU34_06570
excisionase
Salmonella enterica subsp. enterica serovar Enteritidis str. EC20111174

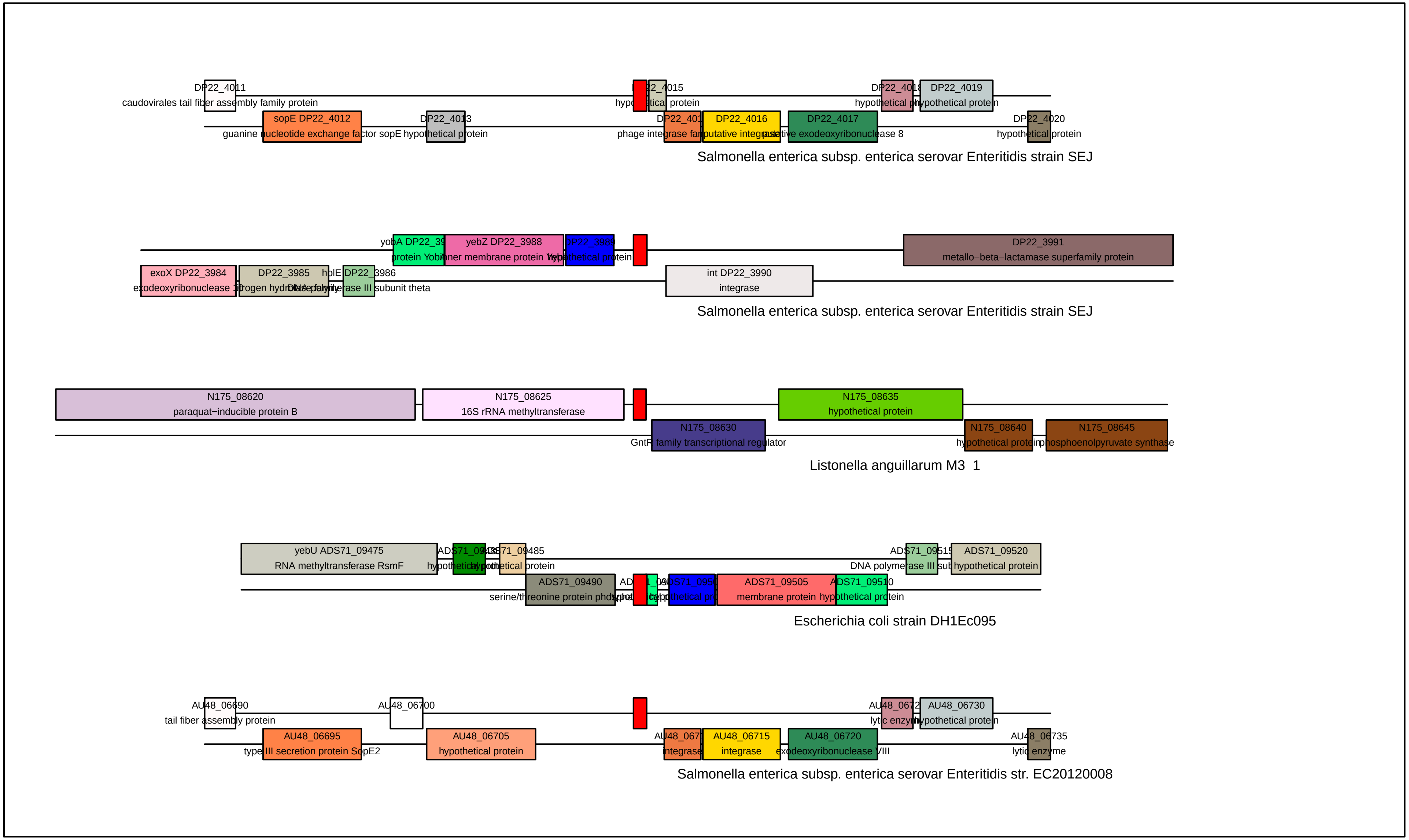

DP22_4011
caudovirales tail fiber assembly family protein
sopE DP22_4012
DP22_4013
DP22_4016
DP22_4017
DP22_4019
hypothetical protein
Salmonella enterica subsp. enterica serovar Enteritidis strain SEJ
yebZ DP22_3988
DP22_3991
metallo−beta−lactamase superfamily protein
exoX DP22_3984
DP22_3985
int DP22_3990
integrase
Salmonella enterica subsp. enterica serovar Enteritidis strain SEJ
N175_08620
paraquat−inducible protein B
N175_08625
16S rRNA methyltransferase
N175_08635
hypothetical protein
N175_08630
N175_08640
N175_08645
Listonella anguillarum M3 1
yebU ADS71_09475
RNA methyltransferase RsmF
ADS71_09520
ADS71_09490
ADS71_09505
Escherichia coli strain DH1Ec095
AU48_06730
AU48_06695
AU48_06705
hypothetical protein
AU48_06715
integrase
AU48_06720
Salmonella enterica subsp. enterica serovar Enteritidis str. EC20120008

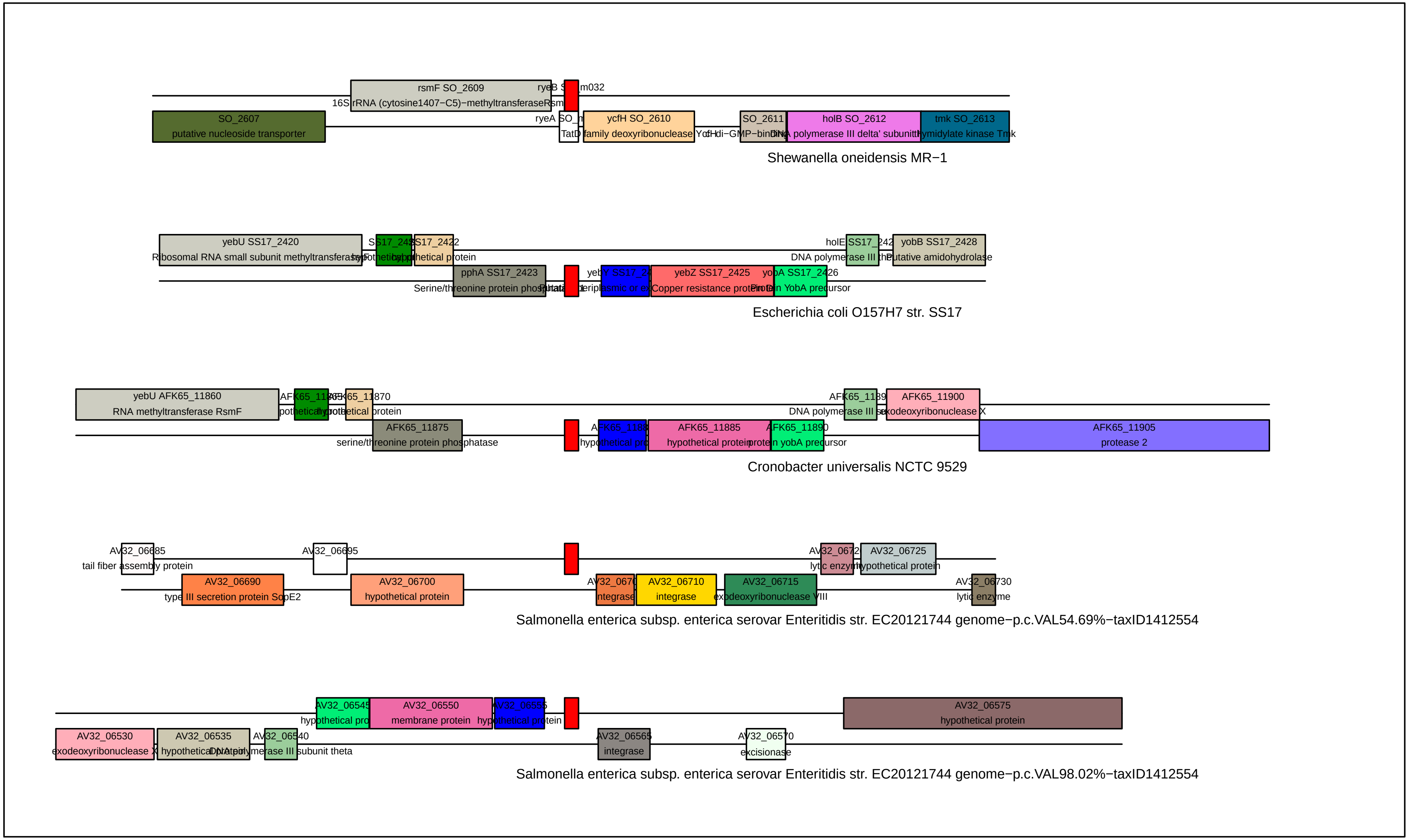
rsmF SO_2609
SO_2607
putative nucleoside transporter
ycfH SO_2610
SO_2611
holB SO_2612
tmk SO_2613
Shewanella oneidensis MR−1
yebU SS17_2420
pphA SS17_2423
yebZ SS17_2425
Copper resistance protein D
yobB SS17_2428
Putative amidohydrolase
Serine/threonine protein phosphatase
Escherichia coli O157H7 str. SS17
yebU AFK65_11860
RNA methyltransferase RsmF
AFK65_11875
serine/threonine protein phosphatase
AFK65_11885
hypothetical protein
AFK65_11900
AFK65_11905
protease 2
Cronobacter universalis NCTC 9529
AV32_06685
tail fiber assembly protein
AV32_06690
type III secretion protein SopE2
AV32_06695
AV32_06700
hypothetical protein
AV32_06710
integrase
AV32_06715
exodeoxyribonuclease VIII
AV32_06725
hypothetical protein
AV32_06730
lytic enzyme
Salmonella enterica subsp. enterica serovar Enteritidis str. EC20121744 genome−p.c.VAL54.69%−taxID1412554
AV32_06545
AV32_06550
membrane protein
AV32_06575
hypothetical protein
AV32_06530
exodeoxyribonuclease X
AV32_06535
AV32_06540
AV32_06565
integrase
AV32_06570
excisionase
Salmonella enterica subsp. enterica serovar Enteritidis str. EC20121744 genome−p.c.VAL98.02%−taxID1412554

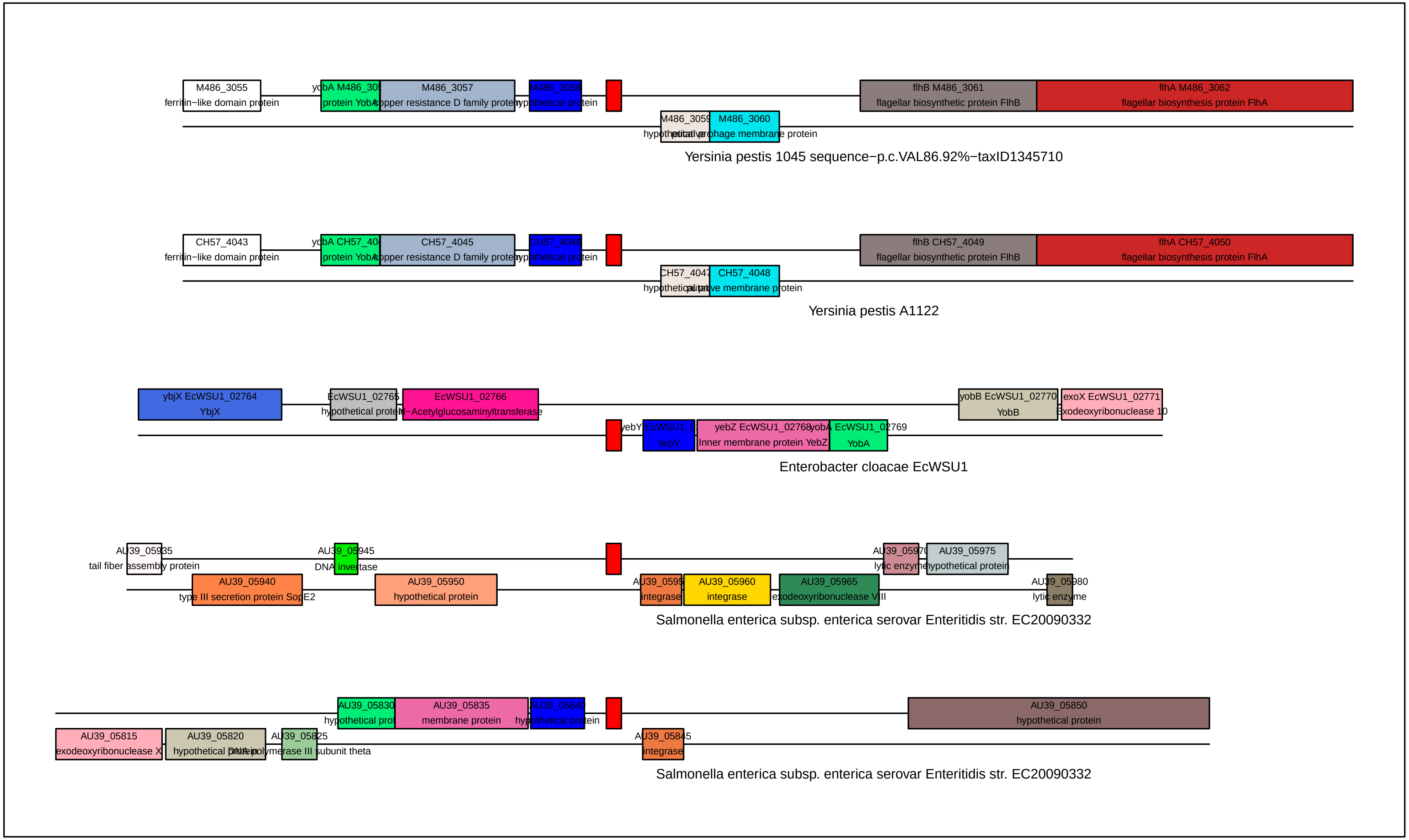

M486_3055
ferritin−like domain protein
M486_3057
copper resistance D family protein
flhB M486_3061
flagellar biosynthetic protein FlhB
flhA M486_3062
flagellar biosynthesis protein FlhA
M486_3060
Yersinia pestis 1045 sequence−p.c.VAL86.92%−taxID1345710
CH57_4043
ferritin−like domain protein
CH57_4045
flhB CH57_4049
flagellar biosynthetic protein FlhB
flhA CH57_4050
flagellar biosynthesis protein FlhA
CH57_4048
Yersinia pestis A1122
ybjX EcWSU1_02764
YbjX
EcWSU1_02766
yobB EcWSU1_02770
YobB
exoX EcWSU1_02771
Exodeoxyribonuclease 10
yebZ EcWSU1_02768
Inner membrane protein YebZ
YobA
Enterobacter cloacae EcWSU1
AU39_05940
type III secretion protein SopE2
AU39_05950
hypothetical protein
AU39_05960
integrase
AU39_05965
AU39_05975
Salmonella enterica subsp. enterica serovar Enteritidis str. EC20090332
AU39_05835
membrane protein
AU39_05850
hypothetical protein
AU39_05815
AU39_05820
Salmonella enterica subsp. enterica serovar Enteritidis str. EC20090332

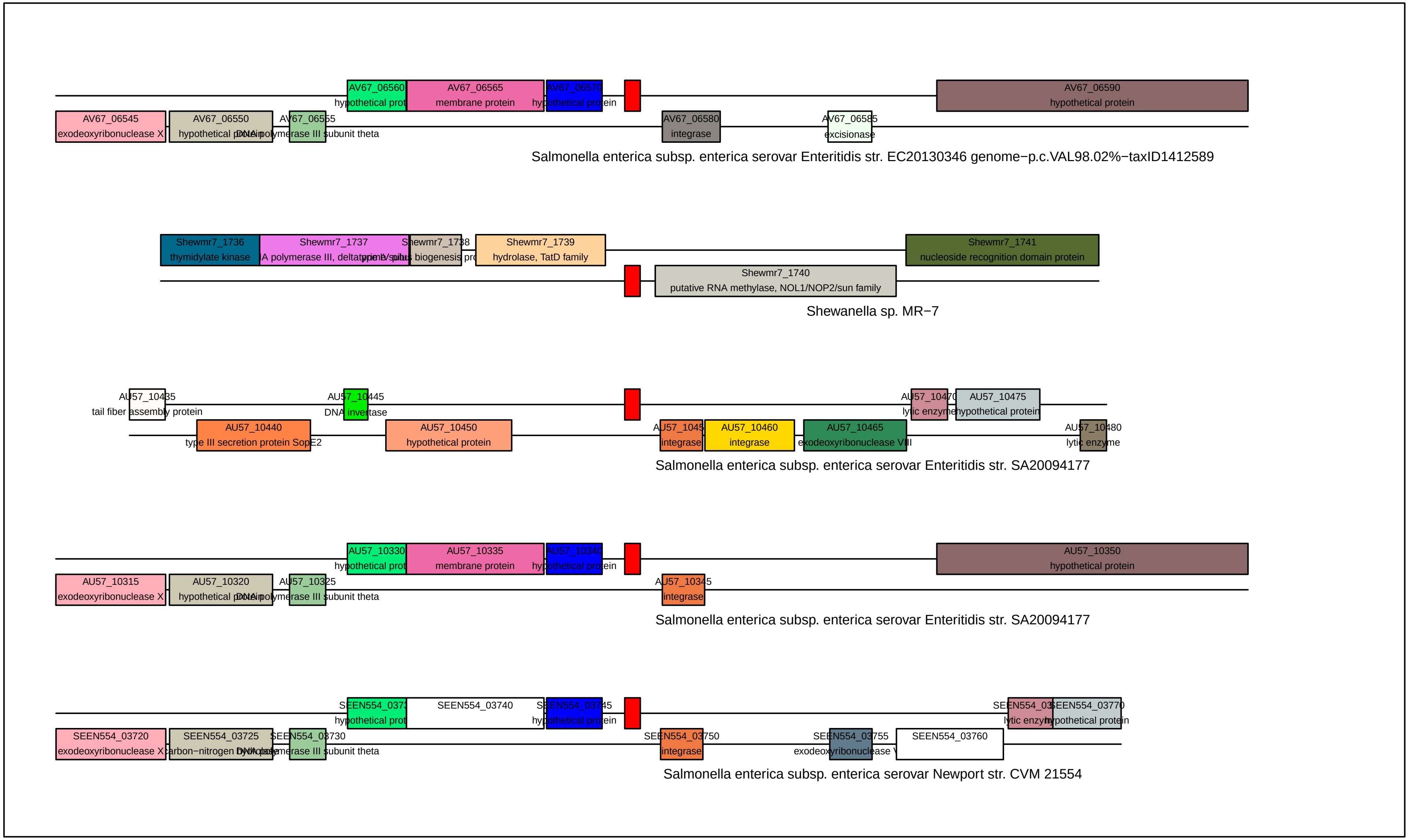

Salmonella enterica subsp. enterica serovar Enteritidis str. EC20130346 genome−p.c.VAL98.02%−taxID1412589
AV67_06545 exodeoxyribonuclease X
AV67_06550 hypothetical protein
AV67_06555 DNA polymerase III subunit theta
AV67_06560 hypothetical protein
AV67_06565 membrane protein
AV67_06570 hypothetical protein
AV67_06580 integrase
AV67_06585 excisionase
AV67_06590 hypothetical protein
Shewanella sp. MR−7
Shewmr7_1736 thymidylate kinase
Shewmr7_1737
Shewmr7_1738
Shewmr7_1739 hydrolase, TatD family
Shewmr7_1740 putative RNA methylase, NOL1/NOP2/sun family
Shewmr7_1741 nucleoside recognition domain protein
Salmonella enterica subsp. enterica serovar Enteritidis str. SA20094177
AU57_10435 tail fiber assembly protein
AU57_10440 type III secretion protein SopE2
AU57_10445 DNA invertase
AU57_10450 hypothetical protein
AU57_10460 integrase
AU57_10465 exodeoxyribonuclease VIII
AU57_10475 hypothetical protein
AU57_10480 lytic enzyme
Salmonella enterica subsp. enterica serovar Enteritidis str. SA20094177
AU57_10315 exodeoxyribonuclease X
AU57_10320 hypothetical protein
AU57_10325 DNA polymerase III subunit theta
AU57_10330 hypothetical protein
AU57_10335 membrane protein
AU57_10340 hypothetical protein
AU57_10345 integrase
AU57_10350 hypothetical protein
Salmonella enterica subsp. enterica serovar Newport str. CVM 21554
SEEN554_03720 exodeoxyribonuclease X
SEEN554_03725
SEEN554_03730 DNA polymerase III subunit theta
SEEN554_03740
SEEN554_03745 hypothetical protein
SEEN554_03750 integrase
SEEN554_03755
SEEN554_03760
SEEN554_03770 hypothetical protein

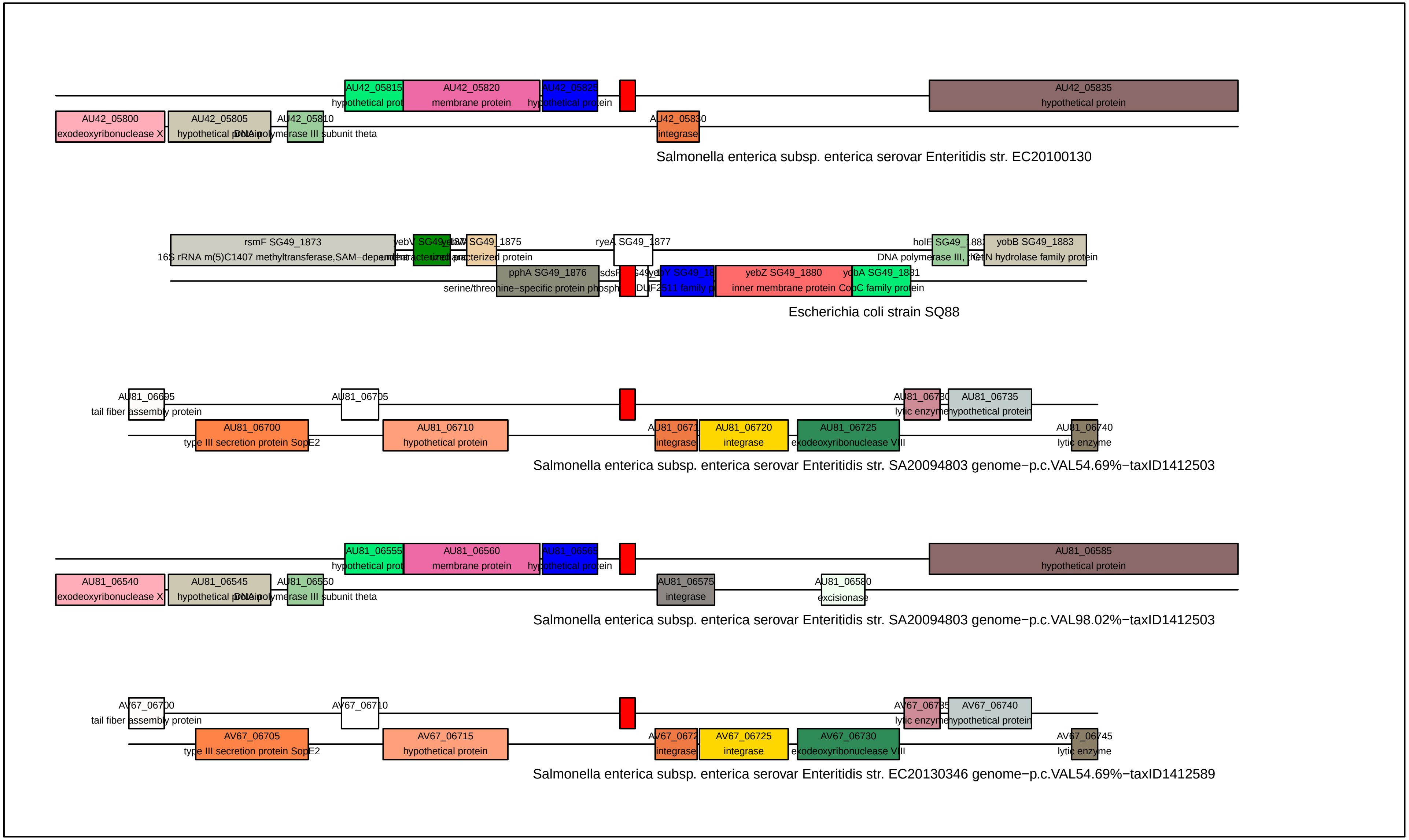
AU42_05815
AU42_05820
membrane protein
AU42_05825
AU42_05835
hypothetical protein
AU42_05800
exodeoxyribonuclease X
AU42_05805
AU42_05810
DNA polymerase III subunit theta
AU42_05830
integrase
Salmonella enterica subsp. enterica serovar Enteritidis str. EC20100130
rsmF SG49_1873
16S rRNA m(5)C1407 methyltransferase,SAM−dependent
SG49_1875
uncharacterized protein
ryeA SG49_1877
holE
yobB SG49_1883
CN hydrolase family protein
pphA SG49_1876
yebZ SG49_1880
inner membrane protein CopC family protein
Escherichia coli strain SQ88
AU81_06695
tail fiber assembly protein
AU81_06705
AU81_06735
hypothetical protein
AU81_06700
type III secretion protein SopE2
AU81_06710
hypothetical protein
AU81_06720
integrase
AU81_06725
exodeoxyribonuclease VIII
AU81_06740
lytic enzyme
Salmonella enterica subsp. enterica serovar Enteritidis str. SA20094803 genome−p.c.VAL54.69%−taxID1412503
AU81_06555
AU81_06560
membrane protein
AU81_06585
hypothetical protein
AU81_06540
exodeoxyribonuclease X
AU81_06545
AU81_06550
DNA polymerase III subunit theta
AU81_06575
integrase
AU81_06580
excisionase
Salmonella enterica subsp. enterica serovar Enteritidis str. SA20094803 genome−p.c.VAL98.02%−taxID1412503
AV67_06700
tail fiber assembly protein
AV67_06710
AV67_06740
hypothetical protein
AV67_06705
type III secretion protein SopE2
AV67_06715
hypothetical protein
AV67_06725
integrase
AV67_06730
exodeoxyribonuclease VIII
AV67_06745
lytic enzyme
Salmonella enterica subsp. enterica serovar Enteritidis str. EC20130346 genome−p.c.VAL54.69%−taxID1412589

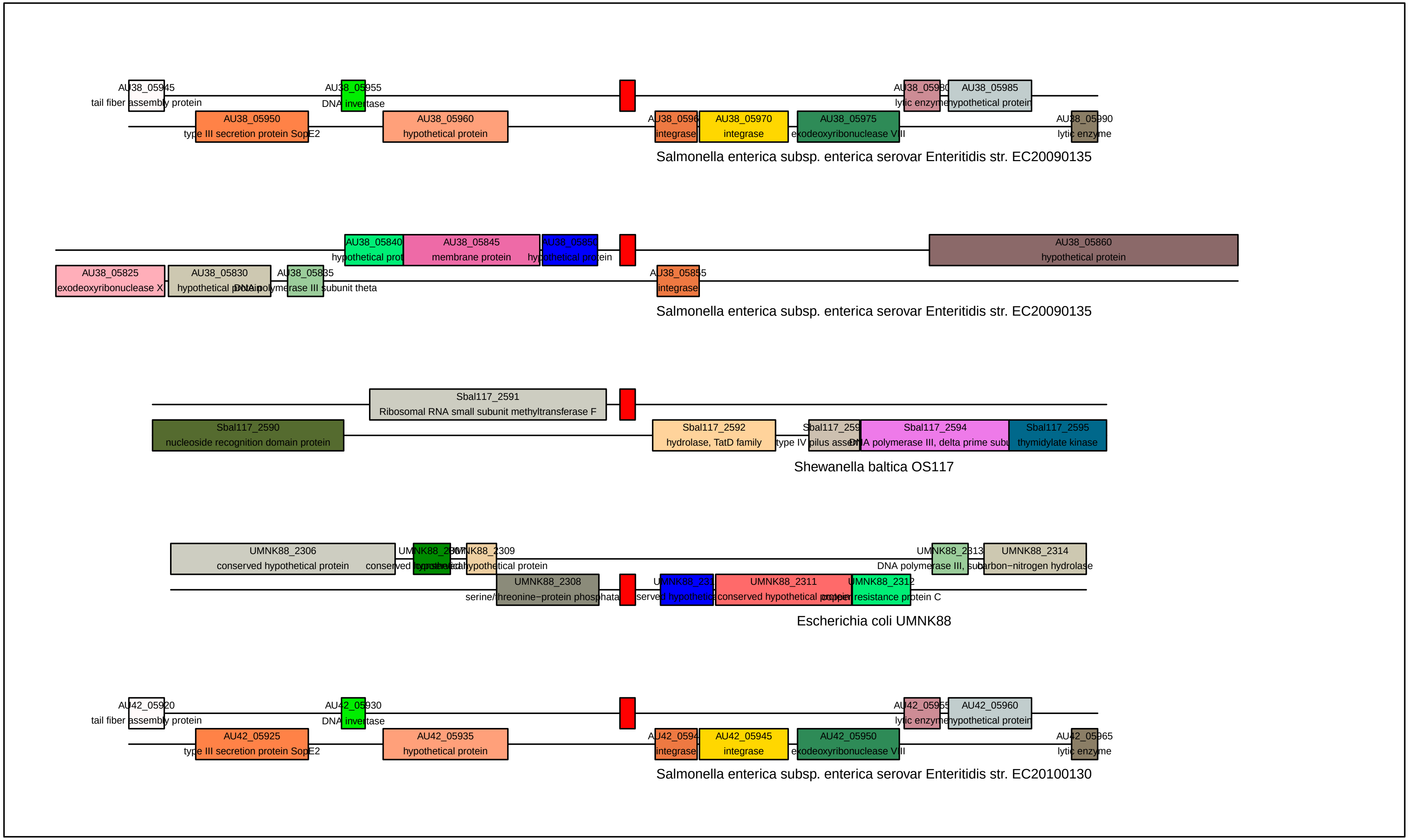

AU38_05945
tail fiber assembly protein
AU38_05955
DNA invertase
AU38_05950
type III secretion protein SopE2
AU38_05960
hypothetical protein
integrase
AU38_05970
integrase
AU38_05975
exodeoxyribonuclease VIII
lytic enzyme
AU38_05985
hypothetical protein
lytic enzyme
Salmonella enterica subsp. enterica serovar Enteritidis str. EC20090135
AU38_05840
AU38_05845
membrane protein
AU38_05860
hypothetical protein
AU38_05825
exodeoxyribonuclease X
AU38_05830
integrase
Salmonella enterica subsp. enterica serovar Enteritidis str. EC20090135
Sbal117_2591
Ribosomal RNA small subunit methyltransferase F
Sbal117_2590
nucleoside recognition domain protein
Sbal117_2592
hydrolase, TatD family
Sbal117_2594
Sbal117_2595
thymidylate kinase
Shewanella baltica OS117
UMNK88_2306
conserved hypothetical protein
UMNK88_2314
UMNK88_2308
UMNK88_2311
Escherichia coli UMNK88
AU42_05920
tail fiber assembly protein
AU42_05930
DNA invertase
AU42_05925
type III secretion protein SopE2
AU42_05935
hypothetical protein
integrase
AU42_05945
integrase
AU42_05950
exodeoxyribonuclease VIII
lytic enzyme
AU42_05960
hypothetical protein
AU42_05965
lytic enzyme
Salmonella enterica subsp. enterica serovar Enteritidis str. EC20100130

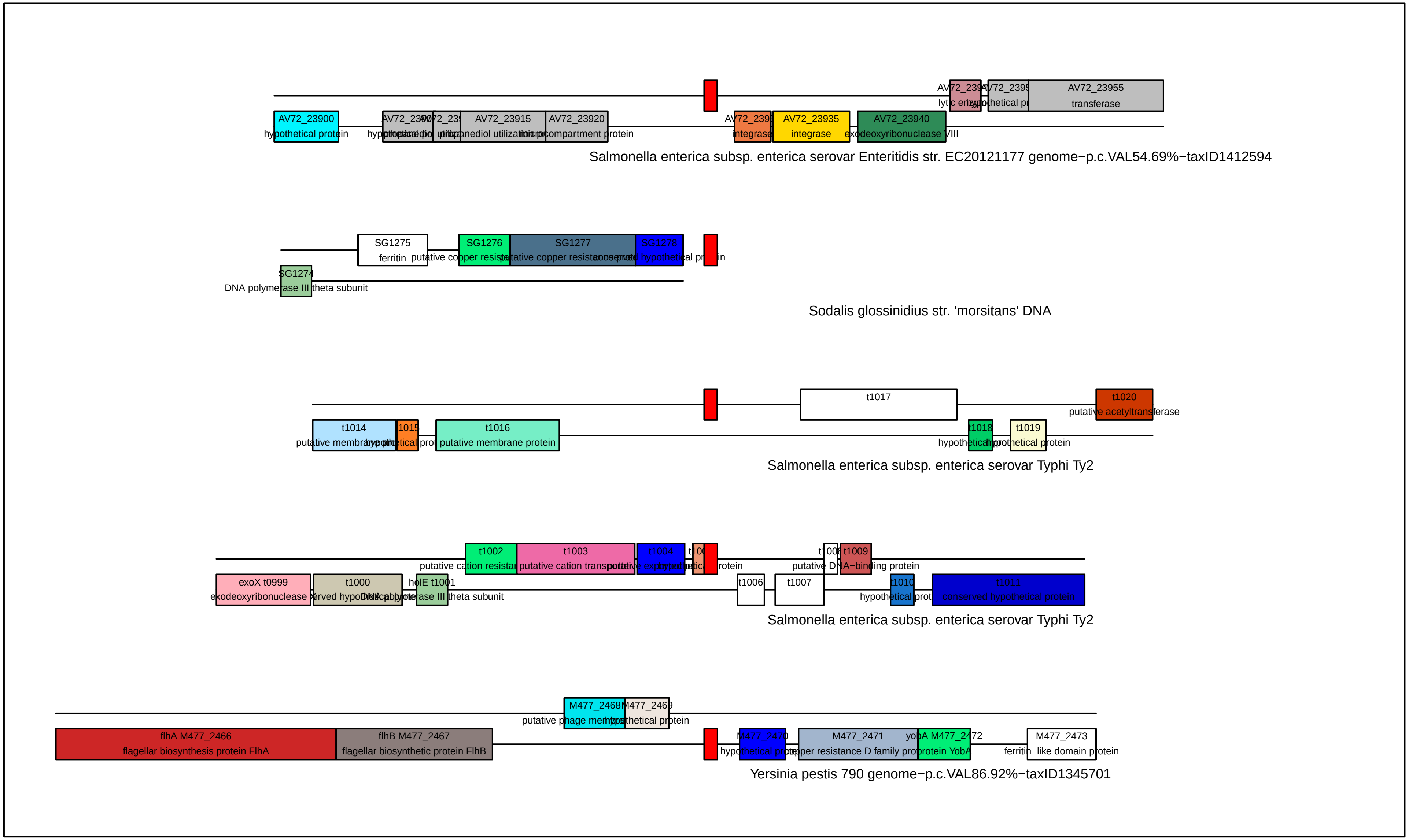

AV72_23900
hypothetical protein
AV72_23915
AV72_23920
AV72_23935
integrase
AV72_23940
AV72_23955
transferase
Salmonella enterica subsp. enterica serovar Enteritidis str. EC20121177 genome−p.c.VAL54.69%−taxID1412594
SG1274
SG1275
ferritin
SG1276
SG1277
SG1278
Sodalis glossinidius str. 'morsitans' DNA
t1014
t1016
putative membrane protein
t1017
t1018
t1019
t1020
putative acetyltransferase
Salmonella enterica subsp. enterica serovar Typhi Ty2
exoX t0999
t1000
holE t1001
t1002
t1003
t1004
t1006
t1007
t1009
t1010
t1011
conserved hypothetical protein
Salmonella enterica subsp. enterica serovar Typhi Ty2
flhA M477_2466
flagellar biosynthesis protein FlhA
flhB M477_2467
flagellar biosynthetic protein FlhB
M477_2471
M477_2473
ferritin−like domain protein
Yersinia pestis 790 genome−p.c.VAL86.92%−taxID1345701

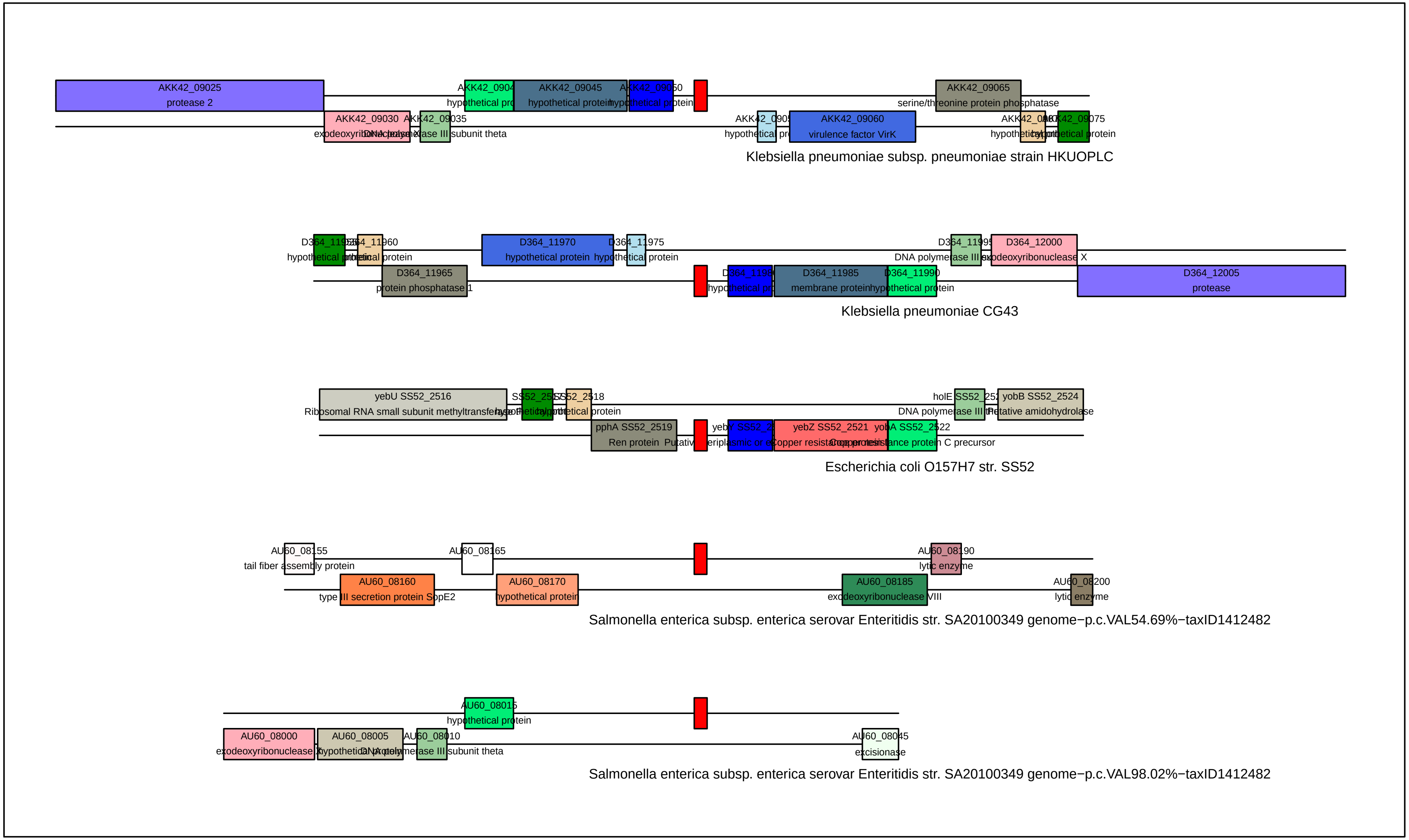
AKK42_09025
protease 2
AKK42_09045
hypothetical protein
AKK42_09065
serine/threonine protein phosphatase
AKK42_09030
AKK42_09035
subunit theta
AKK42_09060
virulence factor VirK
Klebsiella pneumoniae subsp. pneumoniae strain HKUOPLC
D364_11970
hypothetical protein
D364_11975
D364_12000
exodeoxyribonuclease X
D364_11965
protein phosphatase 1
D364_11985
membrane protein
D364_12005
protease
Klebsiella pneumoniae CG43
yebU SS52_2516
SS52_2518
yobB SS52_2524
pphA SS52_2519
yebZ SS52_2521
Escherichia coli O157H7 str. SS52
AU60_08155
tail fiber assembly protein
AU60_08165
AU60_08190
lytic enzyme
AU60_08160
AU60_08170
AU60_08185
AU60_08200
lytic enzyme
Salmonella enterica subsp. enterica serovar Enteritidis str. SA20100349 genome−p.c.VAL54.69%−taxID1412482
AU60_08015
hypothetical protein
AU60_08000
AU60_08005
AU60_08010
subunit theta
AU60_08045
excisionase
Salmonella enterica subsp. enterica serovar Enteritidis str. SA20100349 genome−p.c.VAL98.02%−taxID1412482

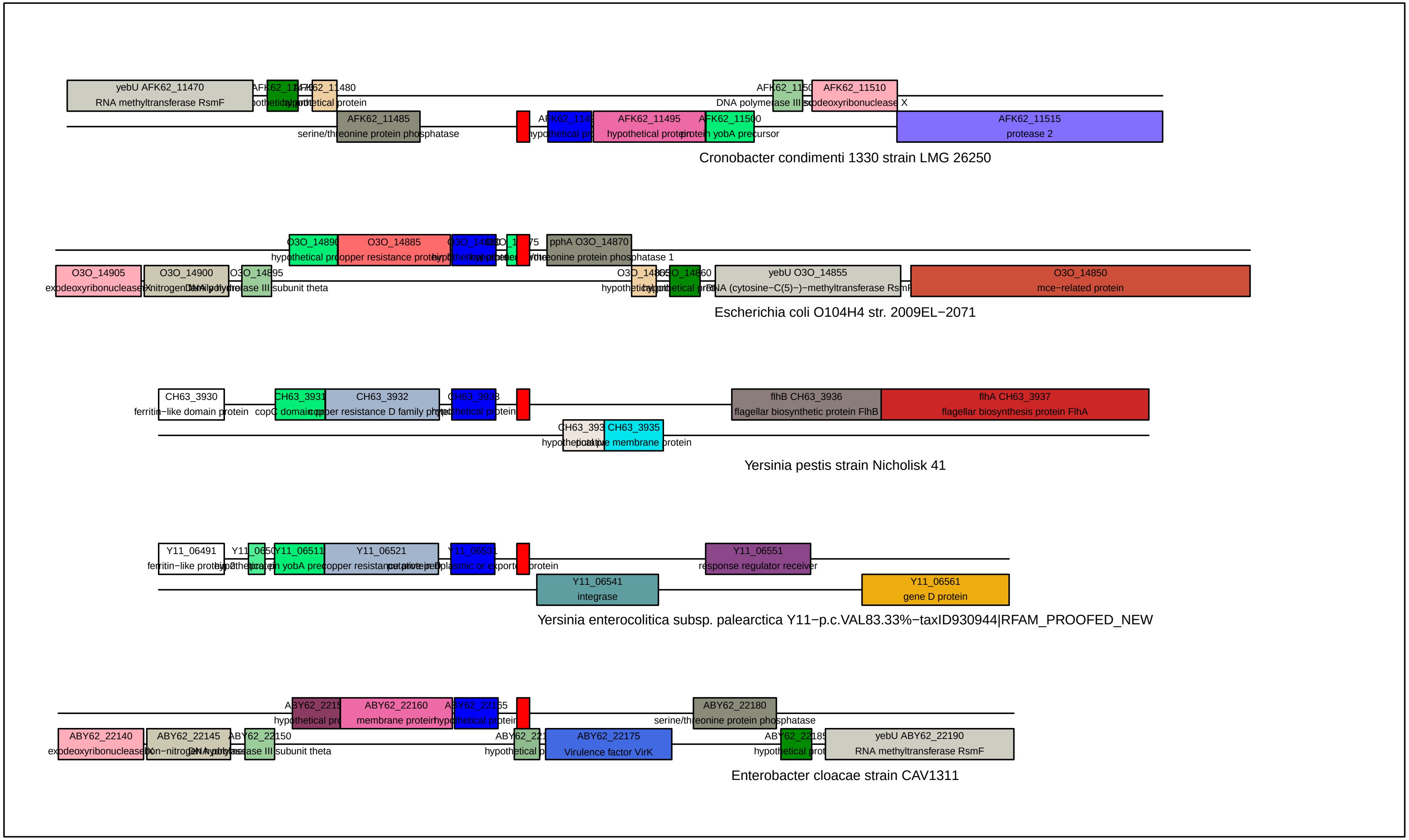

yebU AFK62_11470
RNA methyltransferase RsmF
AFK62_11485
serine/threonine protein phosphatase
AFK62_11495
AFK62_11510
exodeoxyribonuclease X
AFK62_11515
protease 2
Cronobacter condimenti 1330 strain LMG 26250
O3O_14885
pphA O3O_14870
O3O_14905
O3O_14900
yebU O3O_14855
O3O_14850
mce−related protein
Escherichia coli O104H4 str. 2009EL−2071
CH63_3930
ferritin−like domain protein
CH63_3932
flhB CH63_3936
flagellar biosynthetic protein FlhB
flhA CH63_3937
flagellar biosynthesis protein FlhA
CH63_3935
Yersinia pestis strain Nicholisk 41
Y11_06491
Y11_06521
Y11_06551
response regulator receiver
Y11_06541
integrase
Y11_06561
gene D protein
Yersinia enterocolitica subsp. palearctica Y11−p.c.VAL83.33%−taxID930944|RFAM_PROOFED_NEW
ABY62_22160
ABY62_22180
serine/threonine protein phosphatase
ABY62_22140
ABY62_22145
ABY62_22175
Virulence factor VirK
yebU ABY62_22190
RNA methyltransferase RsmF
Enterobacter cloacae strain CAV1311

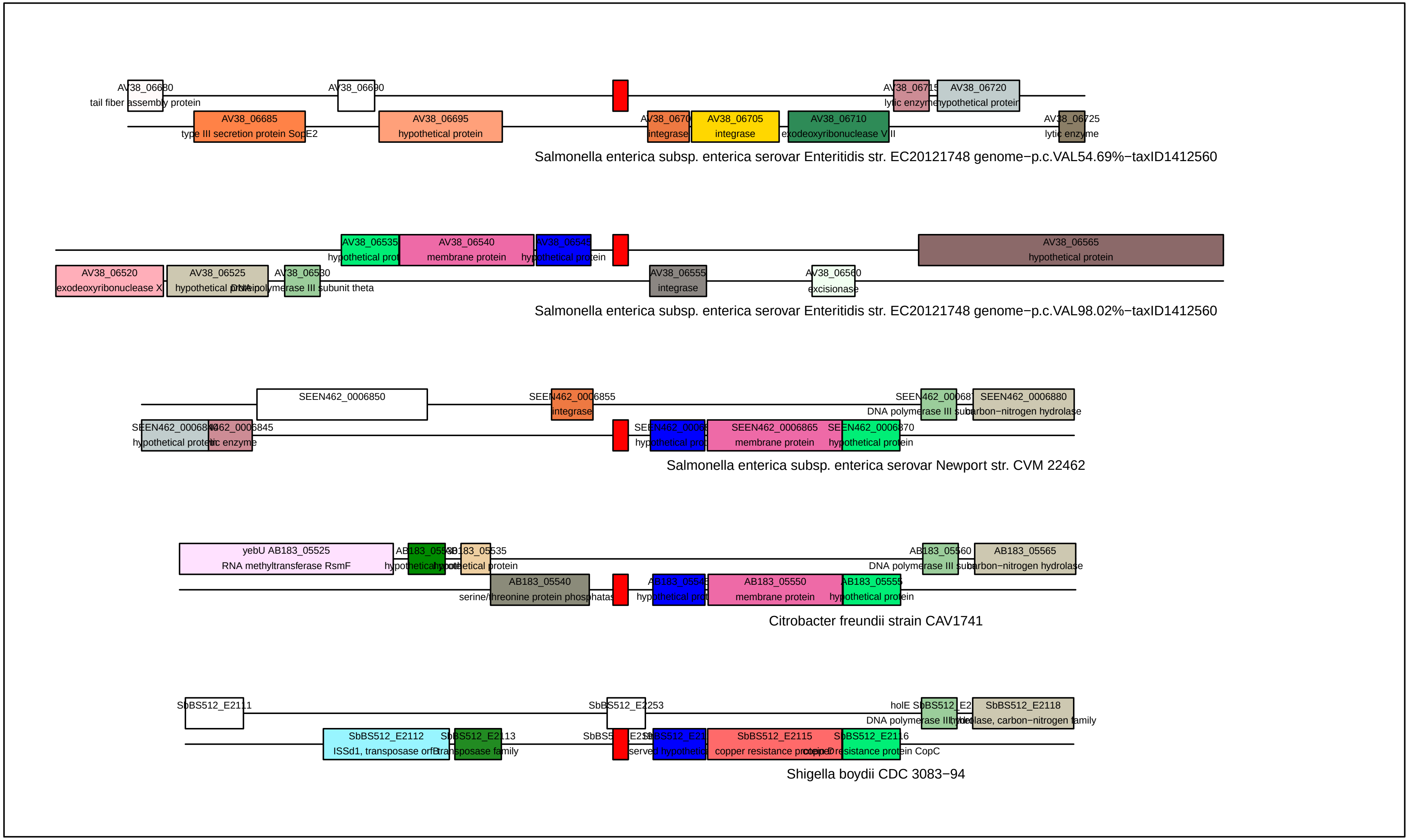
AV38_06680
tail fiber assembly protein
AV38_06690
AV38_06715
lytic enzyme
AV38_06720
hypothetical protein
AV38_06685
type III secretion protein SopE2
AV38_06695
hypothetical protein
integrase
AV38_06705
integrase
AV38_06710
exodeoxyribonuclease VIII
AV38_06725
lytic enzyme
Salmonella enterica subsp. enterica serovar Enteritidis str. EC20121748 genome−p.c.VAL54.69%−taxID1412560
AV38_06535
AV38_06540
membrane protein
AV38_06565
hypothetical protein
AV38_06520
exodeoxyribonuclease X
AV38_06525
AV38_06530
DNA polymerase III subunit theta
AV38_06555
integrase
AV38_06560
excisionase
Salmonella enterica subsp. enterica serovar Enteritidis str. EC20121748 genome−p.c.VAL98.02%−taxID1412560
SEEN462_0006850
SEEN462_0006855
integrase
SEEN462_0006880
carbon−nitrogen hydrolase
SEEN462_0006845
SEEN462_0006865
membrane protein
SEEN462_0006870
hypothetical protein
Salmonella enterica subsp. enterica serovar Newport str. CVM 22462
yebU AB183_05525
RNA methyltransferase RsmF
AB183_05535
AB183_05560
AB183_05565
carbon−nitrogen hydrolase
AB183_05540
AB183_05550
membrane protein
AB183_05555
hypothetical protein
Citrobacter freundii strain CAV1741
SbBS512_E2111
SbBS512_E2253
SbBS512_E2118
SbBS512_E2112
ISSd1, transposase orfB
SbBS512_E2113
transposase family
SbBS512_E2115
copper resistance protein
SbBS512_E2116
Shigella boydii CDC 3083−94

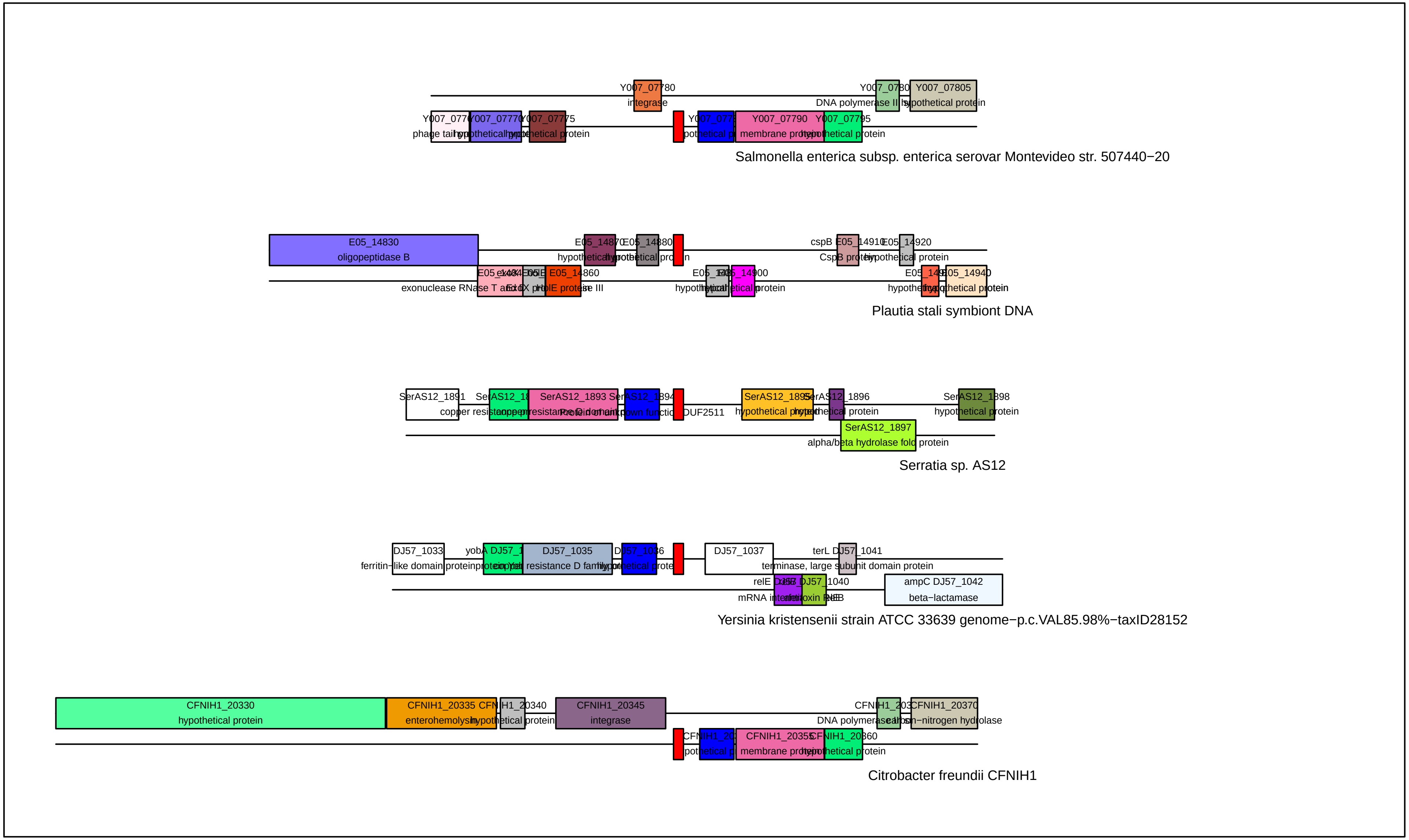
Y007_07780
integrase
Y007_07805
Y007_07790
membrane protein
Salmonella enterica subsp. enterica serovar Montevideo str. 507440−20
E05_14830
oligopeptidase B
E05_14860
cspB
Plautia stali symbiont DNA
SerAS12_1891
SerAS12_1893
SerAS12_1896
SerAS12_1897
alpha/beta hydrolase fold protein
SerAS12_1898
DUF2511
Serratia sp. AS12
DJ57_1033
DJ57_1035
DJ57_1037
ampC DJ57_1042
beta−lactamase
terminase, large subunit domain protein
Yersinia kristensenii strain ATCC 33639 genome−p.c.VAL85.98%−taxID28152
CFNIH1_20330
hypothetical protein
CFNIH1_20345
integrase
CFNIH1_20370
membrane protein
Citrobacter freundii CFNIH1

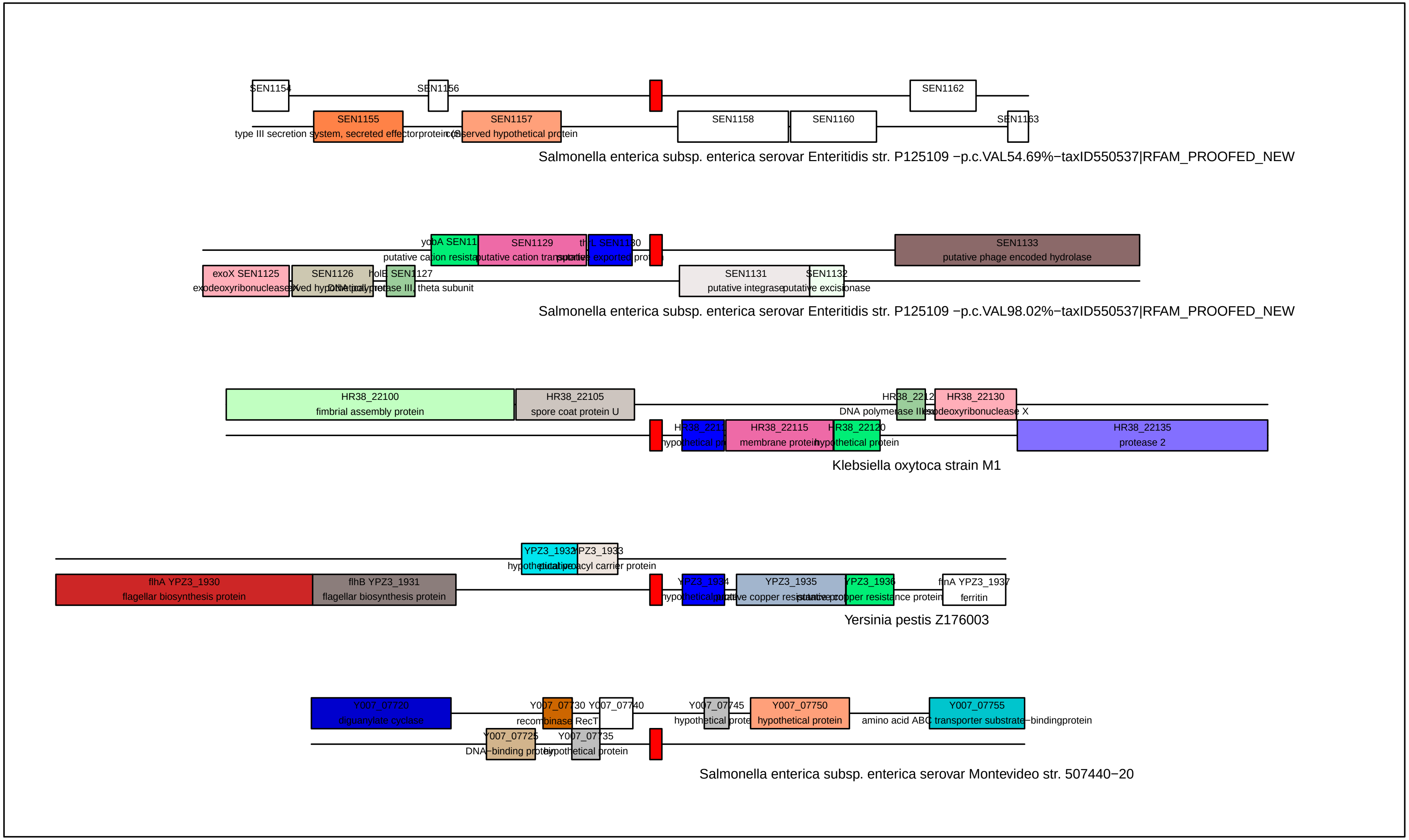

SEN1154
SEN1156
SEN1162
SEN1155
SEN1157
SEN1158
SEN1160
SEN1163
Salmonella enterica subsp. enterica serovar Enteritidis str. P125109 −p.c.VAL54.69%−taxID550537|RFAM_PROOFED_NEW
SEN1129
SEN1133
putative phage encoded hydrolase
exoX SEN1125
SEN1126
SEN1131
putative integrase
Salmonella enterica subsp. enterica serovar Enteritidis str. P125109 −p.c.VAL98.02%−taxID550537|RFAM_PROOFED_NEW
HR38_22100
fimbrial assembly protein
HR38_22105
spore coat protein U
HR38_22130
HR38_22115
membrane protein
HR38_22135
protease 2
Klebsiella oxytoca strain M1
YPZ3_1933
flhA YPZ3_1930
flagellar biosynthesis protein
flhB YPZ3_1931
flagellar biosynthesis protein
YPZ3_1935
ferritin
Yersinia pestis Z176003
Y007_07720
diguanylate cyclase
Y007_07750
hypothetical protein
Y007_07755
Salmonella enterica subsp. enterica serovar Montevideo str. 507440−20

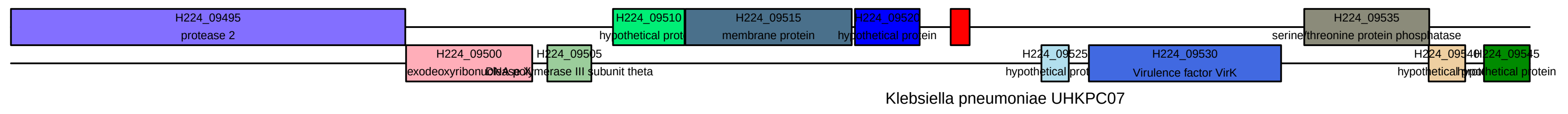
H224_09495
protease 2
H224_09500
exodeoxyribonuclease X
H224_09505
DNA polymerase III subunit theta
H224_09510
H224_09515
membrane protein
H224_09520
hypothetical protein
H224_09525
H224_09530
Virulence factor VirK
H224_09535
serine/threonine protein phosphatase
H224_09545
hypothetical protein
Klebsiella pneumoniae UHKPC07
